# The different routes of parallel evolution in epiarenic growth in a hyperarid desert environment

**DOI:** 10.3389/fpls.2026.1822909

**Published:** 2026-07-07

**Authors:** Marcus A. Koch, Christiane Kiefer, Elena Bergmann, Ron Eric Stein, Michael H. J. Barfuss

**Affiliations:** 1 Centre for Organismal Studies (COS) Heidelberg, Department of Biodiversity and Plant Systematics, Heidelberg University, Heidelberg, Germany; 2 Cluster of Excellence GreenRobust, Heidelberg University, Heidelberg, Germany; 3 Heidelberg Center for the Environment (HCE), Heidelberg University, Heidelberg, Germany; 4 Department of Botany and Biodiversity Research, Faculty of Life Sciences, University of Vienna, Vienna, Austria

**Keywords:** Atacama desert, convergent evolution, divergence time estimates, epiarenic growth, phylogenomics, plastome, *Tillandsia*, *Tillandsia landbeckii*

## Abstract

The Atacama Desert, one of the driest and oldest regions on Earth, represents an extreme environment that has driven remarkable adaptive evolution over millions of years. Within this setting, the genus *Tillandsia* comprises specialists capable of surviving at the dry limit of plant life. These species exhibit crassulacean acid metabolism (CAM), lack functional roots, and possess trichomes adapted for water and nutrient absorption. Of the more c. 750 known *Tillandsia* species, nine occur in the Chilean–Peruvian Atacama Desert, where they colonize bare sand surfaces (epiarenic growth) under hyperarid conditions without significant rainfall, relying solely on nocturnal fog for moisture. Despite their striking adaptations, the evolutionary mechanisms and timing underlying the emergence of epiarenic growth remain poorly understood. Here, we reconstructed a maximum-likelihood phylogeny based on 278 plastome sequences of Tillandsioideae and other Bromeliaceae subfamilies to study respective sister species relationships. Further, divergence time estimates for the origin of epiarenic *Tillandsia* were estimated using Bayesian inference in *BEAST2*. In addition, we analyzed variation in orthologous copies of the nuclear-encoded *Agt1* gene to test for interspecific and interploidal gene flow among epiarenic taxa and their closest relatives. This gene has been previously established as a barcoding marker in bromeliads. The results indicate that epiarenic *Tillandsia* evolved multiple times independently from the Late Pliocene through the Pleistocene, consistent with a long-term hyperarid evolutionary arena promoting extreme adaptations. Moreover, evidence for frequent interspecific hybridization and gene flow suggests that hybridization may have also contributed to the long-term success of epiarenic *Tillandsia* species and is reflecting also the spatio-temporal dynamics of the Atacama’s hyperarid landscapes during the Pleistocene.

## 1 Introduction

The Chilean-Peruvian Atacama Desert is one of the harshest environments on Earth not only for vascular plant species to live in. The desert system forms a nearly continuous arid and hyper-arid region from northern Chile (30°S) to southern Peru (18°S) stretching 1,600 km along the Pacific coast (Rundel et al., 1991). The Atacama Desert area covers c. 100,000 km² ranging in altitude from near sea level to 3,500 m a.s.l. (Bull et al., 2018) and is wedged between two mountain chains, the Chilean Coast Range and the Andes, creating a two-sides rain shadow (Ritter et al., 2019; Houston and Hartley, 2003).

It is widely regarded as the oldest and driest non-polar desert on Earth, with parts of its core experiencing essentially no rain for centuries. Hyper-arid conditions may have persisted for tens of millions of years (Dunai et al., 2005), while plant colonization over millions of years of desert history in the wider region has also been discussed (Luebert and Wen, 2008; Luebert et al., 2011; Böhnert et al., 2020, Böhnert et al., 2022).

The long-term climatic evolution of the Atacama Desert is best described as a protracted transition to extreme aridity rather than an abrupt shift (Sáez et al., 2012; Ritter et al., 2018, Ritter et al., 2022). It has been argued that hyperarid conditions persisted since approximately 20–25 million years ago (Mya) in parts of the region (Dunai et al., 2005). Together, these studies support the view that the Atacama Desert evolved gradually into its modern hyperarid state during the Miocene. However, Pleistocene records from the hyperarid core of the Atacama Desert indicate that hyperaridity was already firmly established but was modulated by episodic phases (Ritter et al., 2019). Fluvial archives from the Late Pleistocene (ca. 17.8–13.8 kya, 11.8 kya, and 1.1–0.7 kya BP) indicate a collapse of hyperaridity in the desert lowlands (Nester et al., 2007). Taken together, these independent archives suggest that Pleistocene climate change in the Atacama involved glacial–interglacial modulation of an already hyperarid system with short-lived hydrological pulses superimposed on long-standing extreme aridity rather than a stepwise onset or termination of hyperarid conditions (Nester et al., 2007; Ritter et al., 2019).

Climatically, its environment is controlled by the cold Humboldt Current offshore, the persistent Pacific high-pressure system, and the double rain-shadow from both mountain chains, all of which suppress cloud formation and precipitation while keeping temperatures relatively mild rather than extremely hot with yearly average day and night temperature of about 18 °C and 14 °C, respectively, in the hyperarid core (Schweikert et al., 2026). A peculiar feature is the thermal inversion layer, which occurs at about 1000 m a.s.l (Del Río et al., 2021). This layer prevents clouds from rising above this threshold; however, they may penetrate inland along orographic corridors for more than 20 km, advancing and regressing in daily cycles (Schween et al., 2022). This phenomenon very rarely brings rainfall, but under certain circumstances it results in regular fog events which represent the only source of water and enable life (Cereceda et al., 2008).

Overall, these conditions create an extreme landscape of salt flats, stony plateaus, alluvial fans, and thick salt playas, with some basins so dry and saline that visible life is nearly absent at the surface. Yet where fog from the Pacific reaches hillsides (the lomas zones) or where groundwater and rare rains are available in valleys and high Andean basins, distinctive plant life appears (Luis, 1986; Jones et al., 2023) and key studies document sparse, highly specialized plant vegetation in the hyperarid core of the Atacama Desert, often limited to cryptogams, endoliths, and ephemeral annuals triggered by rare precipitation (Ruhm et al., 2020). Also microbial biodiversity hotspots have been described accordingly (Alfaro et al., 2021; Hakobyan et al., 2023; Jaeschke et al., 2024). But, generally, the hyperarid core lacks perennial vascular plants (Ruhm et al., 2020).

The most impressive and notable exception among vascular plants is the occurrence of mono- or oligospecific *Tillandsia* vegetation in the hyperarid core of the Atacama Desert covering hundreds of km² of terrain covered by sand from distant sources (Rundel et al., 1997; Rundel and Dillon, 1998; Pauca-Tanco et al., 2020). By taking advantage of remote sensing technologies total distribution size of *Tillandsia* fog oases in Chile and Peru is calculated to be c. 1,900 km² (Moat et al., 2021), which covers less than 5% of the arid and hyperarid coastal area between 100 and 1500 m a.s.l. in Peru (Hesse, 2012). Earlier calculations for northern Chile revealed c. 69 km² of *Tillandsia* fog oases reflecting the extreme conditions in the hyperarid core of the Atacama Desert in Chile (Pinto et al., 2006) compared to Peru (Hesse, 2012). The Atacama Desert is also home to other type of fog oases. In total there are 17,093 km^2^ of verdant fog oases which include ephemeral fog oases (8,414 km^2^) and herbaceous and woody fog oases (8,678 km^2^) (Moat et al., 2021).

The mono- or oligospecific *Tillandsia* vegetation is built up by nine different *Tillandsia* species sharing some peculiar commonalities. They all grow on bare sand, and vegetation develops into highly structured (banded) patterns. Biologically, *Tillandsia* spp. are extreme atmospheric plants that lack functional roots for water uptake, relying instead on leaf trichomes and CAM photosynthesis to harvest and efficiently utilize fog moisture under large daily temperature and radiation fluctuations (Raux et al., 2020). Epiarenic *Tillandsia* species as exemplified by *T. landbeckii* have a long live-span of decades (Koch et al., 2020), propagate mostly clonally but show high spatial genetic structure on population and species´ distribution scale over tens to hundreds of kilometers, which is indicating both, strong local adaptation (Koch et al., 2020; Jabbusch and Koch, 2025) and historical connectivity in fragmented desert landscapes (Latorre et al., 2011; Koch et al., 2022; Stein et al., 2023). Generally, research on banded vegetation has focused on pattern formation theory (Gandhi et al., 2018; Kästner et al., 2025), hydrological parameters (Paschalis et al., 2016; Wang and Zhang, 2019) or catastrophic responses (Rietkerk et al., 2004). For *Tillandsia* vegetation pattern formation there is only little conceptual and modelling information available exemplifying nonreciprocal feedback among plants and environmental factors as drivers for banded pattern formation as a self-organizing response (Hidalgo-Ogalde et al., 2024).

The number of these environmental factors may be low according to the extreme environment *Tillandsia* lomas are exposed to (Koch et al., 2019). Topological parameters have been shown to play a major role (Aponte and Flores, 2013; Pinto-Ramos et al., 2023). This includes for example slope, aspect, elevation and distance from the coast (Wolf et al., 2016), with most of these parameters modulating occurrence and frequency of fog events (Lobos-Roco et al., 2024). Sand availability and movement plays an important role for integrity of plant architecture lacking any root system. Acrotonous and branching shoot growth toward incoming fog is organized to trap sand and progressive dieback and burial of older ramets stabilize the plant and also otherwise mobile substrates (Rundel et al., 1997). Lower night and moderate day temperature support CAM photosynthesis (Lin et al., 2006; Yamori et al., 2014; Heyduk, 2022) and minimize carbon leakage at night (Friemert et al., 1988; Lüttge, 2004). Although it has been calculated that 72% of *Tillandsia* species perform C3 photosynthesis among the 28% performing CAM are also two of the mono-species loma forming species (*Tillandsia landbeckii*, *Tillandsia purpurea*; Crayn et al., 2015). Genetically structured populations show a fine-scaled modulated genotype/phenotype mosaic (Stein et al., 2023; Jabbusch and Koch, 2025). And finally, nutrition responses and nutrient supply from fog (González et al., 2011) and microbial communities (Jaeschke et al., 2024) play a fundamental role in species´ survival. All these adaptations described, however, are not unique to the genus *Tillandsia*. For example, *Cistanthe* (Montiaceae) which also occurs in the Atacama Desert, displays facultative or constitutive CAM photosynthesis, depending on the species (Holtum et al., 2021). Drought tolerance enhancing root microbial communities have been described for *Alhagi sparsifolia* (Zhang et al., 2020). Further, morphological adaptation such as narrow leaves with dense growth are commonly found in plants living in fog oases as they are more efficient at intercepting fog (Martorell and Ezcurra, 2007).

The nine epiarenic – growing on bare sand – *Tillandsia* species belong to the subfamily Tillandsioideae, tribe Tillandsieae and subgenera *Diaphoranthema*, *Aerobia*, *Phytarrhiza* and *T. biflora* and *T. purpurea* species complexes (Möbus et al., 2021; Vera-Paz et al., 2023) with a tribal crown group age of c. 8.8 My (Vera-Paz et al., 2023). This tribe comprises c. 750 *Tillandsia* species (Barfuss et al., 2016; Vera-Paz et al., 2022) and current subgeneric classification is still characterized by arbitrary splitting based on almost exclusively maternally inherited DNA sequences from the plastid genome (Barfuss et al., 2005, Barfuss et al., 2016; Vera-Paz et al., 2023). In all these previous studies single epiarenic species have been included (Barfuss et al., 2016; Möbus et al., 2021; Vera-Paz et al., 2022, Vera-Paz et al., 2023; Kiefer et al., 2024a), which contributes only 1.2% to the genus species diversity. Accordingly, they can be considered as exceptional growth forms among the otherwise most often epiphytically growing species. These species are *T. purpurea*, *T. landbeckii*, *T. virescens* and *T. capillaris* (with the latter two closely related to each other), *T. recurvata*, *T. werdermannii*, *T. paleacea*, *T. marconae*, and *T. latifolia*. They all have their own peculiarities. *Tillandsia purpurea* and *T. landbeckii* are vicariants in Peru and Chile, respectively, with a range overlap in northern Chile/southern Peru. These two endemic species are the most prominent loma forming *Tillandsia*s with a dynamic biogeographic history (Koch et al., 2022; Villasante Benavides et al., 2021). The *T. capillaris*/*T. virescens* species complex has a wider distribution extending from central Peru and Chile across Bolivia to central Argentina (Castello et al., 2016), and outside the hyperarid distribution range the species also grow epiphytically and epilithically (Castello et al., 2016). *Tillandsia recurvata* is a highly diverse species distributed from the southern United States to Peru, northern Argentina and Brazil (GBIF.org, 2026) and it is mostly epiphytic. *Tillandsia recurvata* occurs as epiarenic species in Peru only (Pinto et al., 2006), and does not occur in Chile (Zizka et al., 2009). Endemic and only locally distributed loma forming species in Peru are *T. werdermannii*, *T. latifolia*, and *T. paleacea* (Pauca-Tanco et al., 2020). Finally, the local endemic *T. marconae* occurs in southern Peru (Till and Vitek, 1985) and also in northern Chile (Zizka and Munoz-Schick, 1993) within the sympatric range of *T. landbeckii* and *T. purpurea. Tillandsia marconae* has been proven to be a homoploid hybrid between *T. purpurea* and *T. landbeckii* (Bratzel et al., 2020) with a reticulate evolutionary history (Möbus et al., 2021). Generally, there is poor information on chromosome number and ploidal level variation in the genus *Tillandsia*. It has long been accepted that within the entire subfamily Tillandsioideae only some members of *Tillandsia* subgenus *Diaphoranthema* are reported as tetraploid (Till, 1992) with the majority of species being diploid (2n = 2x = 50). However, Stein et al. (2023) provided first evidence that also cytogenetic evolutionary processes are more complex and involve interspecies and interploidal geneflow with diploids, triploids and tetraploids.

Introgression and hybridization in *Tillandsia* have not been well studied to date. Although Till (2000); Loiseau et al. (2021) and Vera-Paz et al. (2023) put forward hybridization as a topic to be further studied in *Diaphoranthema* and across the entire subfamily Tillandsioideae, there are only few studies on species and population level demonstrating hybridization (Gouda, 2020; Bárrios and Hamilton, 2021; Rossado et al., 2024) or showing the adaptive potential of hybridization and introgression (Stein et al., 2023). Using a phylogenomic approach, high rates of hybridization within and among subclades of subgenus *Tillandsia* suggest that pervasive hybridization may be a major driver of species diversification, range shifts and expansion (Yardeni et al., 2026). Accordingly, one might have to consider that also pervasive hybridization has played a role in the formation of the nine epiarenic *Tillandsia* species in an extreme environment. In the Atacama Desert, the hyperarid conditions driving the evolution of the epiarenic growth form predate the crown-group age of tribe Tillandsieae, providing a long-term evolutionary arena for parallel evolution, with major hyperaridification pulses during the Miocene and Pliocene. Extensive Pleistocene range shifts of species with spatio-temporally variable distributional overlap may have promoted adaptation, but may have also fostered hybridization, giving rise to new epiarenic species via adaptive introgression over the past 2.5 million years.

As *Tillandsia* lomas are a very unique type of vegetation which makes up a prominent part of the vegetation in the most arid parts of the Atacama Desert, we investigate the timing of the origin of exclusively or partly epiarenic species in a phylogenomic framework by analysing a plastome phylogeny that includes roughly 40% of known *Tillandsia* species and all putative sister species of the nine epiarenic taxa. Divergence-time analyses address whether major hyperaridification pulses correlate with the emergence of epiarenic species. We ask the question, how often epiarenic growth form on species level evolved independently; and we also considered hybridization and reticulation as processes through which the epiarenic life style eventually may have been “introgressed” into other lineages. Therefore we used additional sequence data from the nuclear-encoded low-copy gene glyoxylate aminotransferase, which has been introduced as Bromeliaceae-specific DNA barcode marker (*Agt1*; Bratzel et al., 2020), and which we consider valuabe to detect hybrids. Additional evidence for cytogenetic dynamics resulting from introgression and hybridization is studied by genome size estimates. All results are discussed in the context of the emergence and evolution of potential key traits, adaptive features, and biogeographical patterns.

## 2 Materials and methods

### 2.1 Study region, plant material and research strategy

The nine *Tillandsia* species (tribe Tillandsieae, subfamily Tillandsioideae) growing on bare sand are found only in the arid and hyperarid coastal Desert systems of Peru and Chile. Those are considered “epiarenic species” herein. Few of these species have a wider distribution and occur (more) often also as epiphyte (*T. recurvata*, *T. virescens*, and *T. capillaris*). *Tillandsia landbeckii* grows very rarely epiphytically on cacti in Chile or in Peru at higher altitudes (*T. landbeckii* subsp. *andina*), which may eventually represent the ancestral lineage. All other species (*T. latifolia*, *T. marconae*, *T. paleacea*, *T. purpurea*, and *T. werdermannii*) are terrestrial/epiarenic species forming monospecific or oligospecific *Tillandsia* loma vegetation.

Accordingly, the taxon sampling focused on tribe Tillandsiodeae and its various subgenera and species complexes. Epiarenic *Tillandsia* species are found in *T*. subg. *Diaphoranthema*, *T.* subg. *Aerobia*, *T.* subg. *Phytarrhiza*, the *T. biflora* complex and the *T. purpurea* complex. Overall, taxon sampling of subfamiliy Tillandsioideae tribe Tillandsieae is representative and includes the majority of subgenera and species groups recently characterized in comprehensive phylogenetic analyses (e.g. Barfuss et al., 2005; Vera-Paz et al., 2023; Lyu et al., 2024).

Additonal representatives are included from the two other tribes of subfamily Tillandsiodeae, namely Vrieseae and Catopsideae, and from the subfamilies Puyoideae, Bromelioideae, Pitcairnioideae, Hechtioideae and Brocchinoideae. The most basal genus in Bromeliaceae is considered *Brocchinia* (e.g. Kessous et al., 2025), hence *Brocchinia micrantha* is used herein as outgroup.

Newly analysed plant material was collected in the wild or obtained from botanical collections with respective metadata available. In total, 34 plastomes including the epiarenic taxa were newly analyzed in this study and complemented by material from previous studies [Möbus et al. (2021), 44 accessions; Vera-Paz et al. (2023), 199 accessions; Chávez-Galarza et al. (2021), 2 accessions; **Supplementary Material 1**)]. The study of Vera-Paz et al. (2023) focused on *Tillandsia* subg. *Tillandsia* but also included many other *Tillandsia* subgenera and species complexes. Accordingly, this study provided a robust plastome-based backbone phylogeny representing the maternal lineage for tribe Tillandsieae and its sister tribe Vrieseeae, which diverged from each other c. 12.5 Mya (Vera-Paz et al., 2023). For the purpose of this study all epiarenic *Tillandsia* species respective non-epiarenic sister species have been identified and added to the analyses according to a comprehensive barcode phylogeny based on plastid *trn*K-*mat*K (Large Single-Copy region, alignment length of 1887 bp.), *rpo*B-*trn*C-*pet*N (LSC-Region,alignment length of 3225 bp.) and *ycf*1 (Small Single-Copy Region, alignment length of 4722 bp.) presented earlier (Barfuss et al., 2016; Möbus et al., 2021). Sometimes sister relationships for taxon selection were not obvious and additional species had to be considered: e.g., within a broadly defined *T. purpurea* complex, *T. straminea* is considered as epiphytic sister. The taxon occurs in southern Ecuador and central Peru growing at altitudes up to 2,500 m a.s.l., and it is argued that *T. straminea* is probably the epiphytic ancestor of *T. purpurea* (Smith and Downs, 1977). *Tillandsia kuehhasii* is believed to be closely related to *T. capillaris* (Till, 1984, Till, 1992, Till, 1995), and in an earlier study it grouped within the *T. virescens* clade (Castello et al., 2016). Additionally, we also consulted the available larger phylogenies based on plastid genes and entire plastomes for sister taxa collection (Granados Mendoza et al., 2017; Vera-Paz et al., 2022, Vera-Paz et al., 2023).

Accordingly, all identified closest relatives and putative non-epiarenic sister taxa have been included in our plastome analyses. The “pairs” of epiarenic and non-epiarenic sister taxa were examined for DNA sequence variation of the nuclear encoded *Agt*1 gene, which has been introduced as a suitable barcode marker system to discriminate Bromeliaceae and in particular Tillandsioideae species on species-level (Bratzel et al., 2020). The *Agt*1 sequencing approach has been chosen to include a reliable nuclear encoded marker gene which would contribute to unravelling hybridization and reticulate evolution by adding a biparentally inherited DNA marker contrasting the plastome phylogeny. The respective taxon selection is provided in **Supplementary Material 2** and studied accessions are also listed in **Supplementary Material 1**.

In the case of available living plant material for the epiarenic and non-epiarenic sister taxa we added genome size data to differentiate between diploids, triploids and tetraploids.

Our study therefore includes three datasets: (1) for phylogenetic reconstruction and divergence time estimates which includes all samples from Vera-Paz et al. (2022, 2023) as well as from Möbus et al. (2021) and Chávez-Galarza et al. (2021) and 34 newly assembled plastomes (reference based assemblies), (2) for hybrid identification which includes the epiarenic taxa as well as their sister taxa, and if the sister taxon was unclear some more closely related taxa and (3) for genome size estimates we included all respective living material available from our collection.

### 2.2 DNA extraction and plastome short read sequencing

Tissue material was either obtained from freshly collected plants from the Botanical Garden Heidelberg or silical-gel dried specimens. DNA was extracted using a modified CTAB Protocol for Bromeliad DNA extraction (Matuszak-Renger et al., 2018) or with the DNeasy Plant Mini Kit (QIAGEN, Hilden, Germany) using a modified protocol with slightly increased volumes and a chloroform/isoamylalcohol step included. DNA concentration was measured using the Qubit^®^ dsDNA HS Assay kit on a NanoDrop photometer (Thermo Fisher Scientific, Dreieich, Germany). The integrity of the DNA was checked on a 1% agarose gel. Sequencing libraries for Illumina sequencing were prepared by Novogene GmbH (Munich, Germany) with an insert size of 350 bp. Illumina sequencing (Genome skimming) was performed on a NovaSeq X Plus, targeting 2 Gbp raw data per sample (150bp paired end reads). Sequencing data have been deposited at ENA (ENA codes are provided in **Supplementary Material 1**).

### 2.3 Reference based assembly of plastomes and annotations

Raw reads were trimmed using *Trimmomatic* v. 0.40-rc1 (Bolger et al., 2014). On average, 89% of input read pairs (forward and reverse) survived trimming (**Supplementary Material 1**). Plastomes were assembled based on a reference (reference-based assembly). In a first step sequencing reads were mapped using *bwa* and the *mem* algorithm (Li, 2013) and a *T. landbeckii* plastome as reference (OR520905; Inverted Repeat B removed to avoid secondary alignments; Kiefer et al., 2024a) resulting into bam files. In a second step ambiguously mapped or unmapped reads were removed from the bam files using *SAMtools* v. 1.16.1-35-g969d449 (Li et al., 2009) and the option -q 1; *picard-tools* v. 1.138 (Broad Institute) was used for removing duplicates.

Next, the *GATK* v. 4.4.0.0 *HaplotypeCaller* (Poplin et al., 2018) was used for variant calling setting ploidy to 1. Indels were removed from the vcf file by *BCFtools* v. 1.16-21-gc9aed47 (Danecek et al., 2021). New sequences including the detected SNPs were generated using the *GATK* v. 4.4.0.0 *FastaAlternateReferenceMaker*. To ensure good quality, regions of low mapping quality or coverage were masked using *GATK* v. 3.8-1-0-gf15c1c3ef *CallableLoci* and subsequently *BEDtools* v. 2.27.1 *maskfasta* (Quinlan and Hall, 2010). As a last step, the *cpanno* script v. 0.9.2. (Kiefer et al., 2024b) was used to annotate the newly generated sequences by alignment. Plastid genomes presented in Möbus et al. (2021) referred to a different reference accession, accordingly we reanalyzed the original raw data using the workflow as presented above.

### 2.4 Alignments and phylogenetic reconstruction of plastome coding sequences

In total 116 sequences encoding proteins, tRNA or rRNA, 21 introns and 116 intergenic sequences were extracted separately using *BEDtools* v. 2.27.1 *getfasta* (Quinlan and Hall, 2010) from the newly generated and annotated sequences as well as from the sequences obtained from Vera-Paz et al. (2023) and Chávez-Galarza et al. (2021). The sequences of multiple exon genes were merged into a single sequence per gene. One of the *rps*12 exons was annotated twice in the bed file due to alternative splicing and only one of the variants was kept. All extracted sequences were aligned using *MAFFT* v. 7.520 (Katoh and Standley, 2013) with the option -adjustdirection. All alignments were curated manually in *AliView* (Larsson, 2014), meaning that sequences of single accessions were removed from an alignment when there was an excess number of SNPs indicating bad sequence quality, very long gaps also indicating problems in sequencing rather than a true deletion or very long (>200bp), non-alignable inversions. After the revision of the alignments, five were removed completely, including two tRNA-coding sequences (*trn*M- CAU, *trn*I-CAU) and four intergenic sequences (*ycf*1-pseudo-intergenic, *ndh*A-intergenic, *psb*F-intergenic, *trn*M-intergenic) because of rearrangements or different annotation compared to the accessions sequenced by Vera-Paz et al. (2022) and Vera-Paz et al. (2023).

All gaps were removed from the alignments using *trimAl* v. 1.2rev59 (Capella-Gutiérrez et al., 2009) to ensure consistency of the data. This was particularly necessary, because indels were only removed from the accessions assembled in this study and not from the accessions retrieved from GenBank. The individual alignments of coding and non-coding sequences were concatenated using the *catfasta2phyml* script (Nylander, 2024) while in the process stretches of N were inserted if a sequence was not available for an accession for one of the alignments. The final alignment used for phylogenetic reconstruction included 114 sequences encoding proteins, tRNA and rRNA (*trn*M-CAU, *trn*I-CAU removed), 108 intergenic sequences (*yc*f1-pseudo-intergenic, *ndh*A-intergenic, *psb*F-intergenic, *trn*M-CAU-intergenic, *atp*E-intergenic, *ndh*K-intergenic, *psb*D-intergenic, *ycf*1-intergenic removed) and 21 introns (none removed). The final alignment is found with **Supplementary Material 3**.

### 2.5 Maximum-Likelihood phylogenetic tree analysis

Phylogenetic inference in our study is based on maximum-likehood criteria. Accordingly, a maximum likelihood plastome phylogeny was generated using *IQ-TREE* v. 2.2.2.4. First, partitions and the best molecular evolutionary model were determined by *ModelFinder* (Kalyaanamoorthy et al., 2017) with the option “-m MFP+MERGE”. *ModelFinder* determined 19 partitions with different substitution models (**Supplementary Material 4**). Second, a maximum likelihood tree as well as 1000 bootstrap replicates were generated in *IQ-TREE*, and *Brocchinia micrantha* from the subfamily Brocchinioideae served as an outgroup congruent to Vera-Paz et al. (2023). *FigTree* v. 1.4.4 was used to display the tree for final editing (Rambaut, 2018).

### 2.6 Divergence time estimates

Divergence time estimates were calculated based on Bayesian inference running *BEAST2* (Bouckaert et al., 2019), relying on an ultrametric tree and using secondary node calibration. For calibration three nodes were selected from Vera-Paz et al. (2023), whose time estimates fall well within the same range as in our previous study (Möbus et al., 2021). The calibration points were defined as follows: crown group age of Bromeliaceae (*Brocchinia micrantha* versus *Alcantarea odorata*; 95% HPD = 21.06-37.58 Mya), crown age of subfamily Tillandsioideae (*Catopsis paniculata* versus *Werauhia gladioflora*; 95% HPD = 13.72-19.34 Mya) and crown age of Tribe Tillandsieae (*Guzmania osyana* versus *Tillandsia landbeckii*; 95% HPD = 5.94-12.06 Mya). For details on fossil calibration refer to Vera-Paz et al. (2023), who also obtained calibrations from a relaxed, conservative fossil set calibration of Ramírez-Barahona et al. (2020).

Due to the large dataset, data were assigned to four partitions: CDS, RNAs, intergenic spacers and introns and best molecular evolutionary models were selected using *ModelFinder* (Kalyaanamoorthy et al., 2017) embedded in *IQ-TREE* v. 2.2.2.4. The XML configuration file for *BEAST2* was generated with *BEAUti* v. 2.6.7. As some of the selected substitution models are not embedded in *BEAST2*, they needed to be manually set up in *BEAUti* for every partition (**Supplementary Material 4**). Clock Models and Tree Models were linked for all partitions. Moreover, the Relaxed Clock Log Normal and Birth Death Tree Model were selected. The chain length of Markov-Chain-Monte-Carlo (MCMC) was set to 200 million generations and sampling every 20,000 generations, whereas every 20.000th logfile was saved. Eight parallel runs were started in *BEAST2* and monitored with *Tracer* v. 1.7.2 (Rambaut et al., 2018). Results were combined by *LogCombiner* v. 2.6.7, summarized in *TreeAnnotator* v. 2.6.7 (settings: 10% burnin, median node heights) and visualized in *FigTree* v.1.4.4 (Rambaut, 2018).

### 2.7 Amplification, cloning and sequencing of *Agt*1

For identifying potential reticulate origin and hybridisation in epiarenic *Tillandsia* species the nuclear encoded barcoding marker *Agt1* (Bratzel et al., 2020) was amplified for 31 species from 65 accessions with 142 sequences in total, of which 83 sequences have been newly generated herein (**Supplementary Material 1**). Taxon selection focused on all epirarenic species and their closest sister taxa (**Supplementary Material 2**). The targeted DNA fragment comprises approximately three-quarters of exon IV (264 nt.), the entire intron IV, as well as a very short portion of exon V (14 nt.). This highly variable DNA marker has been developed and optimized in particular to identify and characterize taxa on species level. The fragment is less suitable for reconstructing gene trees with high node support. Accordingly, the exploration of allelic variation is focusing on defining groups of alleles and their charcteristic distribution pattern across epiarenic taxa.

PCR amplification of the *Agt*1 marker region was performed using 10–20 ng DNA template, 5x Taq reaction buffer (5 mM dNTPs and 15 mM MgCl_2_), 10 pmol of each primer (AGT1-SP6-Fw: 5’-ATTTAGGTGACACTATAGATTGATGTCGCATTAACCGGC-3’ and AGT1-M13-Rev: 5’-AACAGCTATGACCATGGCAGTTCTTCAGTCCCCATG-3’). The cycling conditions were the following: Initial 95 °C 3 min., followed by 30 cycles [95 °C 30 s.; 56 °C 20 s.; 72 °C 20 s.] and a final step by 72 °C 5 min. and hold at 4 °C. PCR products were visualized on a 1% agarose gel, cut out and purified using the PureLink Quick Gel Extraction Kit (Thermo-Fischer Scientific, Waltham, MA, USA). PCR products were sent out for custom Sanger sequencing (Eurofins Genomics, Ebersberg, Germany).

Electropherograms were then inspected for double peaks or sharp drops in readability of the sequence indicating the presence of more than one allele. If more than one allele was expected, PCR products were cloned into *pJET 1.2*/blunt vector using the CloneJET PCR Cloning Kit (Thermo-Fischer Scientific, Waltham, MA, USA) according to the supplier recommendations. Up to five clones were sequenced per accession using custom Sanger sequencing and SP6 and M13 sequencing primers. All different sequences obtained in this study and all *Tillandsia* sequences from an earlier study (Bratzel et al., 2020) were aligned manually in *Aliview* (Larsson, 2014) to optimize in particular the placing of gaps.

A gene tree was calculated with all alleles, accordingly also multiple alleles per individual were included. We used *raxml-ng*, set GTR as the best fitting molecular evolutionary model and run 1000 bootstrap replicates. The tree was visualized in *FigTree* v. 1.4.4 (Rambaut, 2018) and rooted according to the plastome phylogeny. All *Agt*1 sequences have been deposited in Genbank (**Supplementary Material 1**) and the alignment (611 bp. in length) is provided in **Supplementary Material 5**. Following our experimental approach, we expect three different categories of results: (1) direct sequencing provides unambiguous results providing no evidence for hybridization and introgression, (2) direct sequencing provides good results, but faint PCR products may result in additional *Agt1* copies after cloning and which are placed at uncertain position in the Agt1 allele diversity tree. This result may be seen as evidence of paralogue copies being remnants of a reticulate past history involving other species carrying divergent alleles, and (3) direct sequencing fails completely and cloning strategy reveals highly divergent alleles and indicates this as a strong signature of a past reticulate history. These results, of course, can be validated further by comparing the genetic signature of the maternally inherited plastome.

### 2.8 Genome size estimates as a proxy for ploidy level determination

Genome size estimates were performed on selected individuals for the epiarenic and non-epiarenic sister taxa where fresh leaf material was available. We aimed at analyzing potential ploidal level variation among the studied species as an additional indicator for potential reticulate evolutionary processes (e.g. homo- or polyploid speciation) (**Supplementary Material 6**). Furthermore, selected three examples suitable for testing potential inter-species geneflow in sympatric populations. We included population-level sampling of epiarenic *Tillandsia landbeckii* from three large populations from the southern (Caldera, 69 individuals studied), central (Oyarbide, 82 individuals studied) and northern (Arica, 60 individuals studied) Chilean distribution range (Stein et al., 2023; Schweikert et al., 2026). The sampled population areas cover in all three cases c. 4 km². In the northern population single *T. marconae* individuals are present, and, accordingly, two respective samples have been analysed, too. In the southern population, non-epiarenic *T. geisii* grows on few dispersed cacti, and has been also sampled and studied together with *T. landbeckii*. At Oyarbide there are no *Tillandsia* species nor other vascular plants present.

Nuclear DNA content was determined using flow cytometry following a simplified protocol (Doležel et al., 2007). To release nuclei from the tissue, approximately, 10 mm^2^ of fresh and young leaf tissue from each sampled plant was chopped together with approximately 15 mm^2^ of *Zea mays* cv. CE-777, which served as an internal standard (5.43 pg/2C; Doležel et al., 1989). Chopping was done using a sharp razor blade in a petri dish containing 0.5 mL of ice-cold Nuclei Extraction Buffer from the CyStain™ PI Absolute P kit (ref 05-5022, Sysmec-Partec GmbH, Münster/Görlitz, Germany). The suspension containing the nuclei was filtered through a 30-μm CellTrics^®^ filter (Sysmec-Partec GmbH, Münster/Görlitz, Germany). 2.0 mL of Staining Buffer from the CyStain™ PI Absolute P kit (ref 05-5022, Sysmec-Partec GmbH, Münster/Görlitz, Germany), containing propidium iodide and RNase, were added for staining the nuclei and removing the RNA which could disturb the signal. After 90 min incubation at room temperature in the dark, the relative fluorescence intensity of 10.000 particles was recorded using a flow cytometer (Sysmex RP-310; Sysmex-Partec GmbH, Münster/Görlitz, Germany) equipped with a green (532-nm) solid state laser. We applied the following stringent criteria to get precise and stable flow cytometric results: (i) only analyses where the coefficient of variation of the sample peak was below 5% were taken into account, (ii) each sample was measured twice on different days to minimize potential random instrumental drift (Doležel and Bartoš, 2005), and (iii) if the between-day variation exceeded a 5% threshold another measurement was done and the most remote value was discarded when the sample was re-analyzed. The histograms were recorded with the *CyView* software version 1.9.0.1057.5d66c74 (Sysmex-Partec GmbH, Münster/Görlitz, Germany) using the following parameters: pump speed was set to 4 µL/sec., particle threshold was adjusted with min. 10000 nuclei, cleaning trigger was selectd with “all events – no gates” and set to “DNA 6,7486%, and linear scaling was used. The software *CyBatch* version 1.0.4.24702 (Sysmex-Partec GmbH, Münster/Görlitz, Germany) was used for data analysis using peak analysis set to “automatic” and CV limits of the G1-peaks adjusted with < 5%.

We also used our earlier studied plant material as additional internal reference: triploid *T. landbeckii* X *T. capillaris* (Stein et al., 2023); tetraploid *T. capillaris* (Stein et al., 2023); diploid *T. landbeckii* with 2.23–2.61 pg (n = 10, mean 2.41 pg, SD 0.11) and diploid *T. purpurea* with 2.11–2.96 pg (n = 3, mean 2.40 pg, SD 0.39) and *T. marconae* with a mean of 2.28 pg (n = 2, SD 0.01) (Möbus et al., 2021). In total we added 300 new genome size estimates to previous reports.

Genome sizes were assigned to ploidal level following comparisons and estimates presented earlier by Gitaí et al. (2014), and using ploidy level estimates comparing genome sizes and allele frequency distribution spectra based on genetic data and separating diploid, triploids and tetraploids (ddRAD) (Stein et al., 2023). It should be noted that generally genome size was used as a proxy to infer ploidal level due to a lack of knowledge regarding ploidy levels of Bromeliaceae.

### 2.9 Generation of an Atacama Desert precipitation map and distribution maps of epiarenic *Tillandsia* taxa

Distribution data for all nine epiarenic *Tillandsia* species were downloaded from https://www.gbif.org/(https://doi.org/10.15468/dl.adcwhc). Data were cleaned using the R-package *CoordinateCleaner* (v3.0.1, Zizka et al., 2019), filtered for observations centered on capitals and institutions, on centroids, rounded coordinates and seas. Geographically and ecologically unplausible records outside the known distribution ranges for *Tillandsia purpurea* and *Tillandsia werdermannii* according to Villasante Benavides et al. (2021) and Pauca-Tanco et al. (2020) were removed. Range polygons were drawn around clusters of at least three observations within 250 km of each occurrence reference other using the package *concaveman* (https://github.com/mapbox/concaveman). The remaining observations and range polygons were plotted in QGIS (QGIS QGIS Developmental Team, 2026) using the CartoDB Positron basemap (https://github.com/CartoDB/basemap-styles; ^©^ CARTO, ^©^ OpenStreetMap contributors). Additionally, a map showing the mean annual precipitation of hyperarid (<25 mm) and arid (<50 mm) regions in the Atacama and Sechura coastal deserts was generated based on high-spatial resolution historical climate data from Fick and Hijmans (2017).

### 2.10 Re-evaluation of the evolution of CAM in Bromeliaceae

CAM mechanism is a perfect adaptation to Desert environments allowing to optimize water use efficiency and take up CO_2_ at night and use it during the day, so their stomata can stay closed when it is hot and dry during the day. Air temperature decrease and cooling at night increase relative humidity thereby lowering transpiration and water loss, and this also reduce carbon leakage from the vacuoles. Accordingly, the combination of hyperaridity and nocturnal fog strongly favours CAM photosynthesis. However, up to date the distribution of CAM photosynthesis was never analysed in a phylogenetic context on a broader scale summarizing all knowledge currently available. Therefore, we surveyed the literature for available δ^13^C values for our taxon sampling, as δ^13^C values are widely accepted as informative indicators of prevalent photosynthetic pathways (Farquhar et al., 1989). Values less negative than −20‰ indicate predominantly nocturnal carbon fixation via CAM, whereas more negative values (below −20‰) are typical of predominantly daytime carbon fixation via the C_3_ pathway (Osmond, 1978; O'Leary, 1988). Data for taxa investigated herein have been compiled from a comprehensive survey of Bromeliaceae (1,893 species) published by Crayn et al. (2015). Further information has been collected from Lyu et al. (2024) and De La Harpe et al. (2020). Occurrence of CAM has been plotted on the time calibrated plastome phylogeny using Bayesian inferences (*BEAST2*).

## 3 Results

### 3.1 Phylogenetic inferences based on plastome phylogeny

The alignment of the complete plastomes resulted in a 117,324 bp matrix. As described in the Material and Method section one inverted repeat region, small inversions, and various ambiguously aligned regions were excluded. The final alignment is highly in agreement with an earlier large-scale data matrix provided by Vera-Paz et al. (2023) consisting of 132,726 bp, and finally our presented Maximum-likelihood and Bayesian inference analyses are highly congruent with recent results from Vera-Paz et al. (2022, 2023), which indicates reliable and robust datasets.

**Figure 1:**
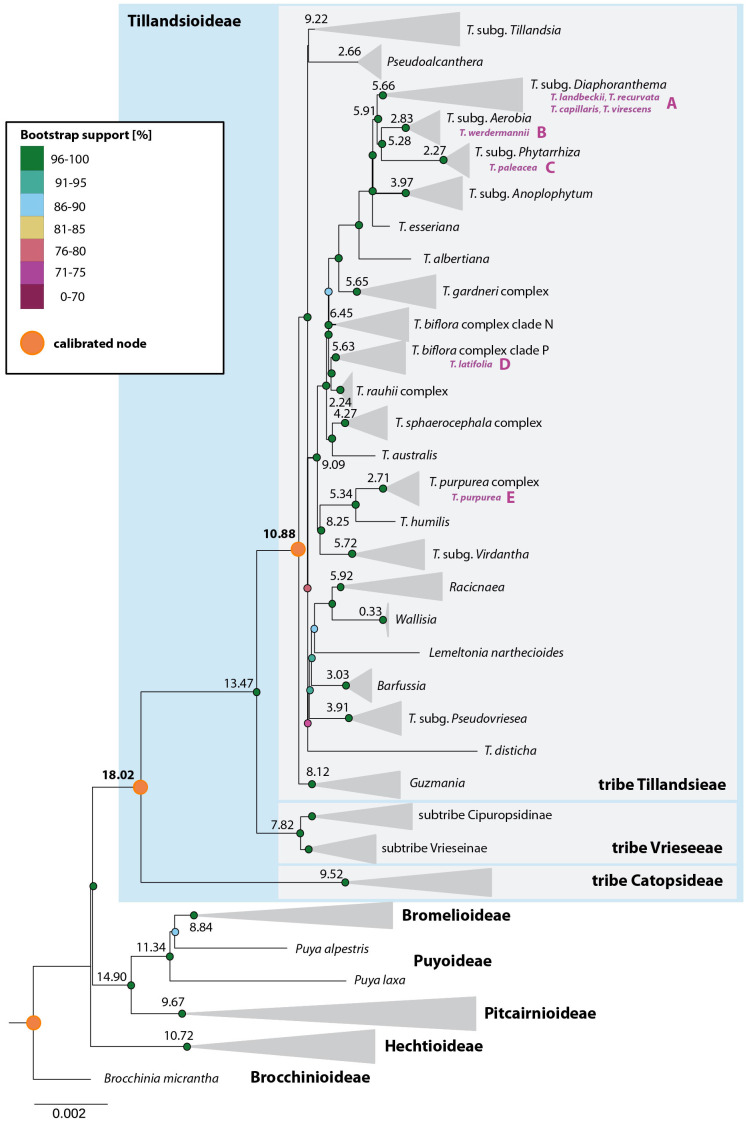
Condensed plastome Maximum Likelihood tree of tribe Tillandsioideae and outgroup clade. The detailed tree is provided with **Supplementary Material 7**. Bootstrap values are indicated by colour code. Clades with epiarenic *Tillandsia* species are denoted **(A–E)** and point to respective **Figures 2A** and **4B–E**. Divergence time estimates are redrawn from *BEAST2* analysis (**Supplementary Material 8**).

Phylogenetic relationships for all major groups and clades of the *ML* analysis received strong statistical bootstrap support (≥ 85%). The detailed *ML* tree is available in **Supplementary Material 7**. The respective ultrametric Bayesian tree, estimated using *BEAST2*, showed congruent results with the ML tree and is available in **Supplementary Material 8**. Divergence time estimates from *BEAST2* have been shown along the respective nodes in ML analyses. Subfamilies Brochinoideae (outgroup), Hechtioideae, Pitcairnoideae, Bromelioideae were placed sister to subfamily Tillandsioideae with consistent high bootstrap support > 95% (**Figure 1**). Only representatives of subfamily Puyoideae remain paraphyletic close to subfamily Bromeliodieae. Within subfamily Tillandsiodeae all three tribes Catopsideae, Vrieseae and Tillandsieae are well-supported by bootstrap values > 95%. Within tribe Tillandsieae subgenera and species complexes are also defined by high bootstrap support > 95%, and only few inner nodes remain with lower bootstrap values.

**Figure 2:**
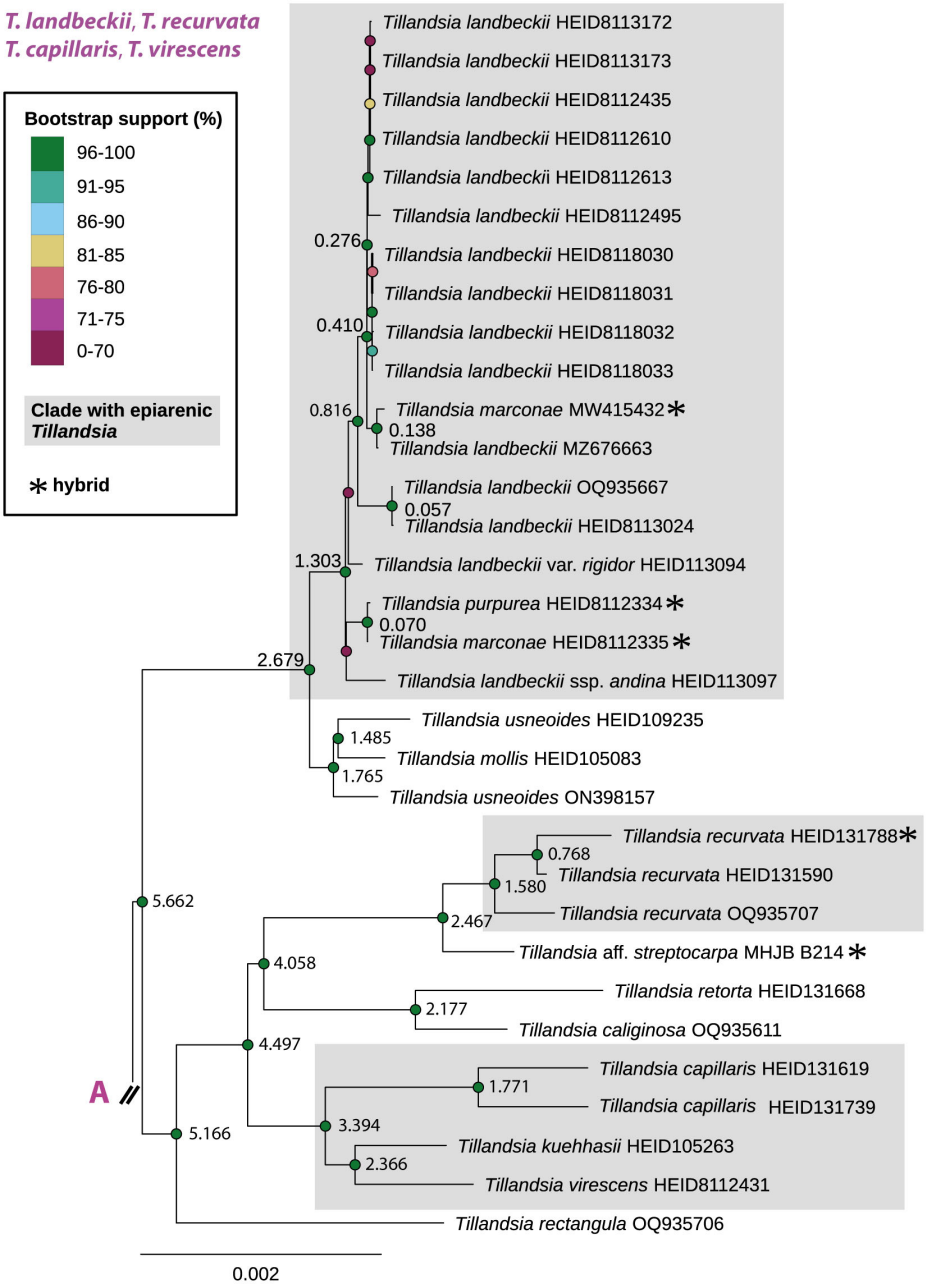
Cutout of the plastome maximum likelihood tree showing *Tillandsia* subg. *Diaphoranthema* (clade A in **Figure 1**). Bootstrap values are indicated by colour code. Clades with epiarenic *Tillandsia* species are underlaid in grey. Divergence time estimates are redrawn from *BEAST2* analysis (**Supplementary Material 8**). Hybrids or introgressed accession are indicated (*).

**Figure 3:**
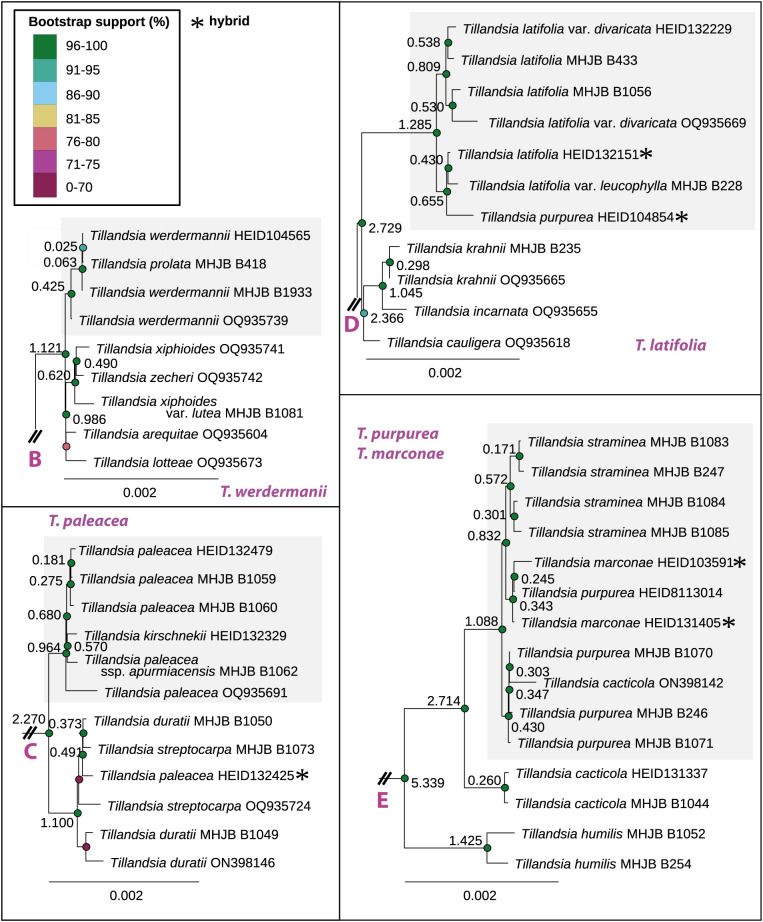
Cutout of the plastome maximum likelihood tree showing clades **(B–E)** from **Figure 1**. Bootstrap values are indicated by colour code. Clades with epiarenic *Tillandsia* species are underlaid in grey. Divergence time estimates are redrawn from *BEAST2* analysis (**Supplementary Material 8**). Hybrids or introgressed accession are indicated (*).

**Figure 4:**
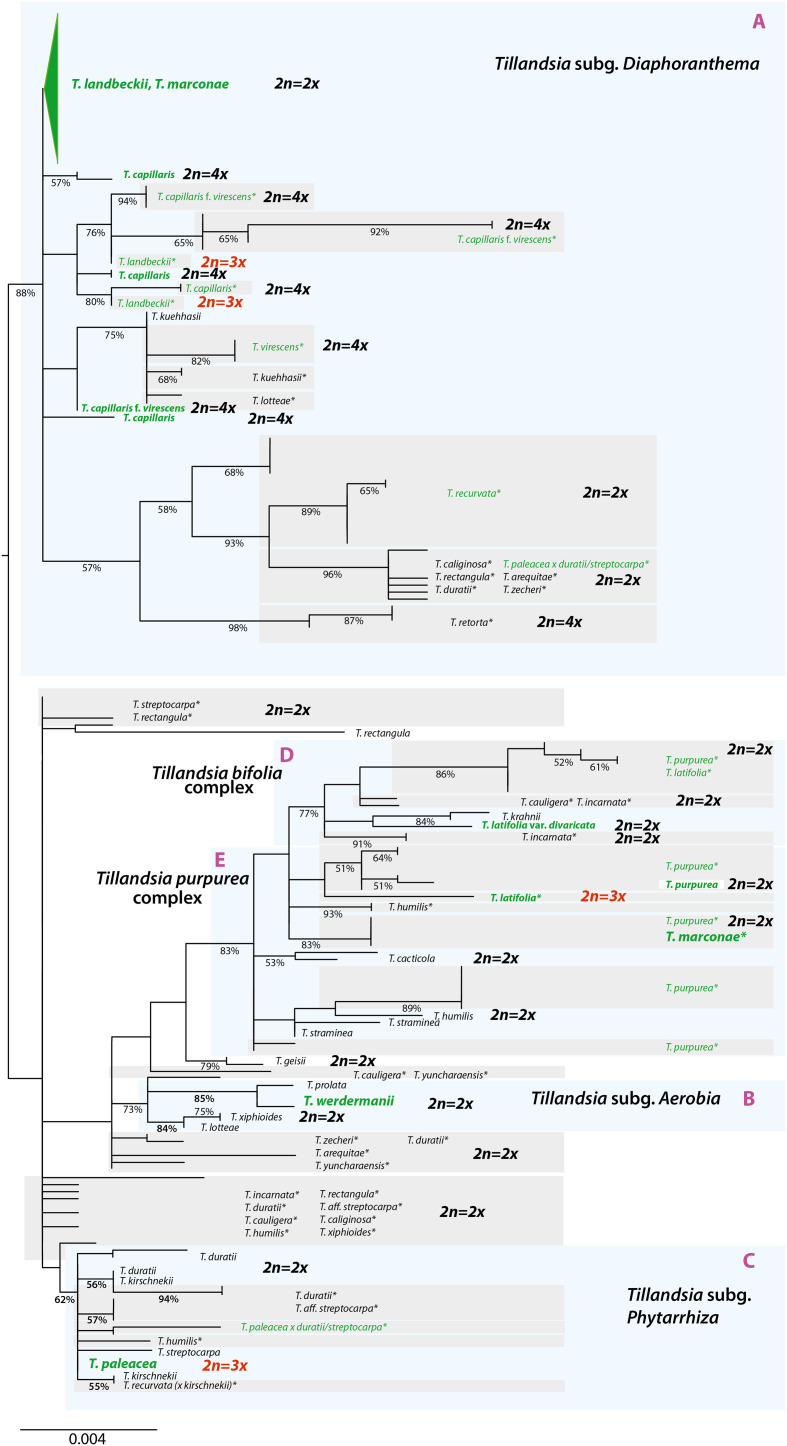
*Agt1* allelic diversity and genetic relationships of epiarenic *Tillandsia* spec. and their closest relatives computed as Maximum-likelihood tree. The tree is a simplification of the original output tree (**Supplementary Material 9**). The five phylogenetic groups which include epiarenic *Tillandsia* species (species names in green) are underlaid in blue and marked (**(A–E)**; see **Figure 1**). All *agt1* alleles obtained from cloned PCR products are underlaid in grey and taxon names are supplemented with an asterisk (*). If available, ploidal level based on genome size estimates (**Supplementary Material 6**) is placed near to respective species or groups of taxa.

The nine epiarenic *Tillandsia* species are distributed among five clearly defined phylogenetic clades (**Figure 1**, details in **Figures 2**, **3** and **4**): (A) Four species, *T. landbeckii*, *T. capillaris*, *T. virescens* and *T. recurvata*, are found in *Tillandsia* subg. *Diaphoranthema*. The respective zoom into the ML tree shows that *Tillandsia landbeckii* is well separated and forms a phylogenetically well-defined taxon sister to *T. usneoides* and *T. mollis*. Nested taxa are found within *T. landbeckii* accessions (*T. purpurea*, *T. marconae*). The two closely related epiarenic species *T. capillaris* and *T. virescens* also form a distinct group with *T. retorta, T. caliginosa* and *T. recurvata* forming a sister clade. Nesting of *T. kuehhasii* within *T. virescens* was expected, because this has been described earlier within a phylogeographic study (Castello et al., 2016). Accordingly, *T. recurvata* is a sister species of *T. retorta* and *T. caliginosa*. The accession which is positioned closest to *T. recurvata*, *T*. aff. *streptocarpa*, is later introduced herein as an accession of hybrid origin. The same accession (*T*. aff. *streptocarpa*) has been identified to be related to *T. bandensis* (Hromadnik, 2026; pers. communication), and this taxon has been already shown to be sister to *T. recurvata* (Donadío et al., 2022). All bootstrap values among species are high and ≥95%. In summary we can consider at least three independent origins of epiarenic growth form among the four species. (B) *Tillandsia werdermanni* is found to belong to *Tillandsia* subg. *Aerobia* (**Figure 1**). Its sister species are *T. lotteae*, *T. arequitae*, *T. xiphioides* and *T. zecheri* (**Figure 3**). Bootstrap values are also consistently high. (C) *Tillandsia paleacea* is a member of *T.* subg. *Phytarrhiza*. Inhere the species is closely related to *T. kirschnekii*, which is nesting within *T. paleacea*, and a sister clade is built up by *T. duratii* and *T. streptocarpa* with >95% bootstrap support (**Figure 3**). (D) *Tillandsia latifolia* from the *Tillandsia bifolia* complex clade P as defined in Vera-Paz et al. (2023) (**Figure 1**) forms a well-defined sister-relationship (> 95% bootstrap support) to *T. krahnii*, *T. incarnata* and *T. cauligera*. Within the *T. latifolia* plastome cluster we also find two hybrid accessions (**Figure 3**). (E) *Tillandsia purpurea* belongs to its own species complex (Vera-Paz et al., 2023), and earlier subgeneric assignment (*T*. subg. *Phytarrhiza*) are misleading (see Donadío et al., 2022 for details). The closest species to *T. purpurea* is *T. straminea*, nesting as a group within *T. purpurea* plastomes. The second well separated sister species is shown to be *T. cacticola* with bootstrap support >95%.

In summary, plastome data favour 6–8 independent origins of *Tillandsia* species evolving epiarenic growth. The case of *T. marconae* is the only obvious hybrid species, which arose from the two epiarenic taxa *T. landbeckii* and *T. purpurea*. And, at least based on plastome data epiarenic growth in *T. capillaris* and *T. virescens* may be considered to have evolved only once.

### 3.2 An evolutionary timeline from plastome BEAST2 analysis

The complete *BEAST2* chronogram is presented in **Supplementary Material 8**. Relevant divergence time estimates are extracted and shown in the *ML* trees (**Figures 1**-**3**). For all nine epiarenic species stem and crown group ages and respective intervals are shown in **Table 1**. The topology of the Bayesian tree obtained with *BEAST2* is largely congruent with *ML* analysis (**Figure 1**). *BEAST2* analysis did not place *Brocchinia* as sistergroup to the rest of the family although the deepest divergence time split (crown group age of Bromeliaceae) was set as calibration point with *Brocchinia micrantha.* However, basal tree topology has no statistical support. Such divergent tree topologies, particularly at the base of the phylogenetic trees, are common when comparing Maximum Likelihood (ML) trees with Bayesian trees (from *BEAST2*), often due to differences in how the methods handle time, substitution models, and incomplete lineage sorting (ILS) (e.g., Fourment et al., 2025).

**Table 1:** Stem and crown group ages [Mya] from plastome derived *BEAST2* analysis (**Supplementary Material 6**) and respective 95% highest posterior density (HPD) are provided.

Epiarenic taxon	Stem group age [Mya] (95% HPD)	Crown group age [Mya] (95% HPD)	Ploidy	Interspecies hybrid
*T. capillaris*	3.393 (2.462-4.427)	1.771 (0.922-2.714)	4x	no
*T. paleacea*	2.270 (1.337-3.281)	0.964 (0.471-1.541)	2x	no
*T. landbeckii*	2.678 (1.666-3.771)	1.303 (0.779-1.879)	2x	no
*T. latifolia*	2.729 (1.832-3.756)	1.285 (0.775-1.851)	2x/3x	no
*T. marconae*	n.a.	0.343 (0.138-0.589)	2x	yes
*T. purpurea*	2.714 (1.460-4.084)	1.088 (0.643-1.592)	2x	no
*T. recurvata*	2.467 (1.599-3.371)	1.580 (0.885-2.306)	4x	no
*T. virescens*	3.393 (2.462-4.427)	2.366 (1.334-3.451)	4x	no
*T. werdermannii*	1.121 (0.654-1.630)	0.425 (0.131-0.791)	2x	no

Ploidy level information refers to genome size estimates (**Supplementary Material 6**) for the nine epiarenic *Tillandsia* taxa. Evidence for interspecies hybrid constitution consider evidence from plastome and *agt1* phylogenetic trees. n.a., not applicable.

In all nine cases of epiarenic taxa crown group ages, the onset of diversification, are placed into the Pleistocene less than 2.58 Ma (Gibbard and Head, 2010). Stem group ages—particularly for species with a wide desert distribution (*T. landbeckii*, *T. purpurea*, *T. capillaris*, *T. virescens*)— are set into the Late Pliocene (**Table 1**). Also the wider and outside the arid-hyperarid deserts distributed *T. paleacea* and *T. recurvata* show stem group ages > 2 Mya. The two narrowly distributed desert species *T. werdermannii* and *T. marconae* are much younger, and diversification is placed within the second half of the Pleistocene (**Table 1**).

### 3.3 Genetic signature of biparentally inherited and nuclear encoded *Agt1*

For identifying taxa with a past reticulate history the *Agt1* barcoding marker was amplified and sequenced either directly or after cloning - if direct sequencing failed - with a focus on epiarenic taxa and their putative sister taxa. A summary of *Ag*t1 allelic sequences generated is provided in **Supplementary Material 1**.

The alignment generated from the obtained sequences had a length of 611 bp. Of these 503 characters were constant, 35 were variable but uninformative while 73 characters were informative.

**Figure 5:**
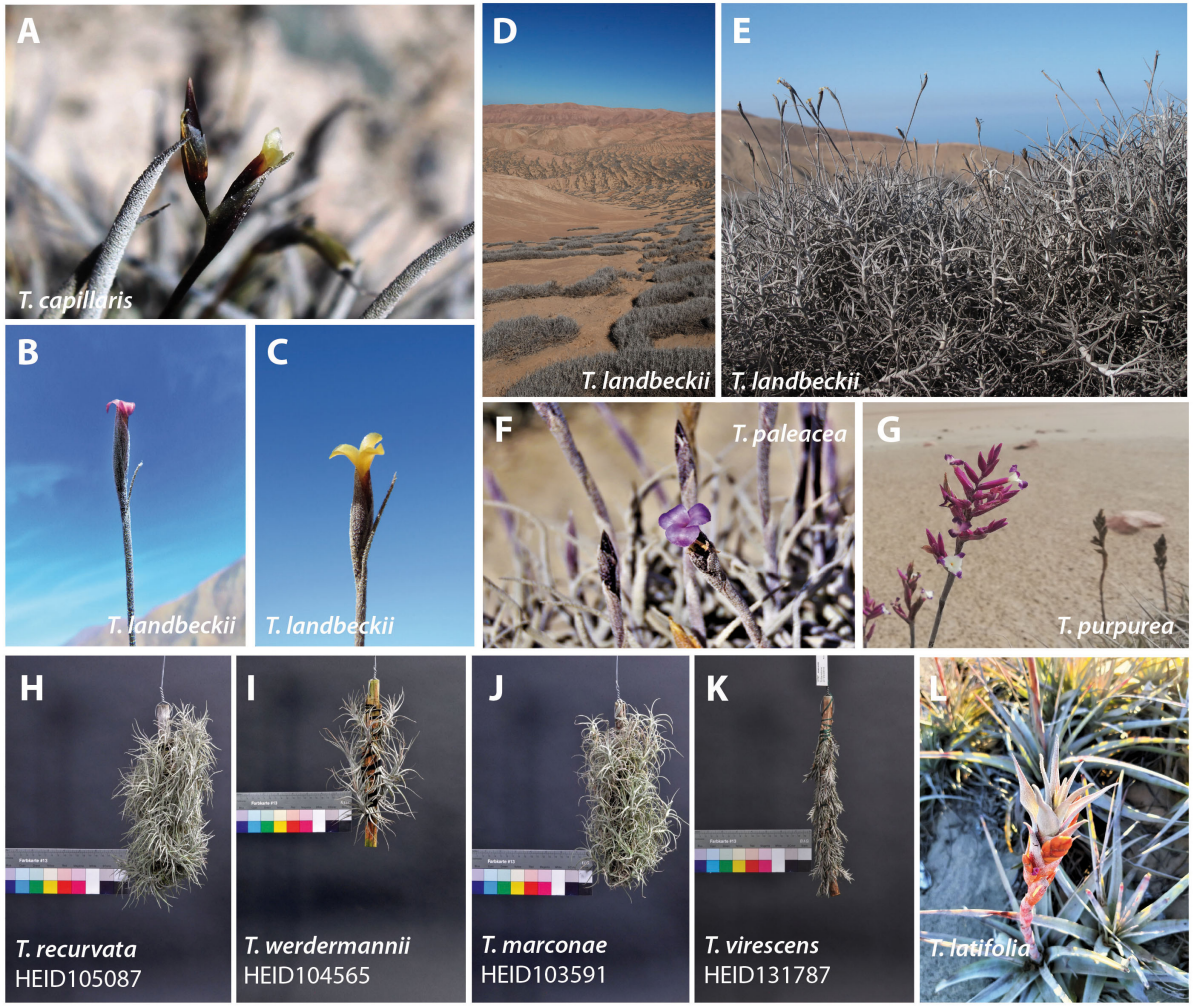
Illustration of the epiarenic species in Chile and Peru. Credits and origins: **(A)** *Tillandsia capillaris*, Camaná (Arequipa, Peru, 2017), **(B)** *Tillandsia landbecki*, Camaná (Arequipa, Peru, 2019), **(C)** *Tillandsia landbecki*, Pachia (Tacna, Peru, 2018), **(D)** *T. landbeckii* typical monospecific vegetation, Cerro Oyarbide (Iquique, Chile, 1999), **(E)** *T. landbeckii*, Cerro Oyarbide (Iquique, Chile, 2001), **(F)** *Tillandsia paleacea*, Caravelí (Arequipa, Peru, 2019), **(G)** *Tillandsia purpurea*, Camaná (Arequipa, Peru, 2018), **(H–K)** various species selected from collections at Heidelberg Botanical Garden. Accession information is accessible via https://gartenbank.cos.uni-heidelberg.de/public/. **(L)** *Tillandsia latifolia*, Marcona (Ica, Peru, 2019) ([^©^G. Anthony Pauca-Tanco: **(A-C, F, G, L)**, ^©^Bot. Garden Heidelberg: **(H–K)**, ^©^Marcus **(A)** Koch: **(D, E)**].

The mid-point rooted *Agt*1 allele tree is shown as ML analysis in **Supplementary Material 9**, and a summary of the tree including bootstrap support (> 50%) is presented in **Figure 4**. Because of the limited size of the *Agt1* marker the number of SNPs is too low to provide high bootstrap support for single alleles. However, this has to be expected and can be considered as normal outcome of such an analysis. The ML tree ignores coding of insertions/deletions, which increase the possibility to identify species-specific alleles (Bratzel et al., 2020), but this of mutations do not contribute to increase node support among clades, because of increased homoplasy and is not considered herein. The *Agt1* allele tree recognize and distinguish the five major clades comprising epiarenic *Tillandsia* species well. Alleles from *Tillandsia* subg. *Diaphoranthema* (**Figure 5**, clade A) are separated with 88% bootstrap support. Alleles from *Tillandsia* subg. *Aerobia* (**Figure 5**, clade B) are defined by a bootstrap support value of 73%. *Tillandsia* subg. *Phytarrhiza* (**Figure 5**, clade C) is characterized by a bootstrap support of 62%. *Agt1* alleles from *Tillandsia bifolia* complex (**Figure 5**, clade D) and alleles from *Tillandsia purpurea* complex (**Figure 5**, clade E) are also characterized by basal bootstrap node support of 77% and 83%, respectively. However, here clade D is separated from clade E, but both are not placed as sister to each other. Considering low bootstrap support among clades A-E and an uncertain position of alleles from cloning procedure we cannot define tree incongruencies among subgenera and species complexes comparing the plastome and *Agt1* tree as significant.

We could identify three different categories of outcomes in our sequencing approach. The first category contained *Agt*1 sequences where direct sequencing was unambiguous. This was true for *T. cacticola*, *T. straminea*, *T. kirschnekii*, *T. prolata*, *T. krahnii* and *T. geissii*. For all of these taxa and accessions we found no evidence for hybridization and introgression between species. The second category contained sequences which looked unambiguous in direct sequencing. However, the cloning of faint PCR products revealed additional alleles, often with uncertain position in the *Agt*1 tree. This was true for sequences generated for *T. capillaris* and *T. virescens*, *T. landbeckii*, *T. rectangula*, *T. purpurea*, *T. yuncharaensis*, *T. humilis*, *T. duratii*, *T. streptocarpa*, *T. lotteae* and *T. xiphioides.* We interpret this outcome as first evidence for putative past interspecies geneflow. The third category contained sequences where direct sequencing did not yield any readable sequences and the cloning approach revealed highly divergent alleles. This was true for *T. kuehhasii*, *T. recurvata*, *T. retorta*, *T. caliginosa*, *T. rectangula*, *T. marconae*, *T arequitae*, *T. zecheri*, *T. cauligera* and *T. incarnata* (**Figure 4**), and we consider this as evidence for a past reticulate history.

Combining both lines of evidence, incongruent species-specific plastome data and *agt*1 allelic composition, we identified the following inter-species hybrids, which are also indicated in **Figures 2** and **3** with asterisks: *Tillandsia recurvata* HEID131788: *T. recurvata* x *T. kirschnekii*; *Tillandsia* aff. *streptocarpa* MHJB B214: *T. bandensis* x *T. streptocarpa*; *Tillandsia marconae* HEID8112335/MW415432: *T. landbeckii* x *T. purpurea*; *Tillandsia purpurea* HEID8112334: *T. landbeckii* x *T. purpurea*; *T. paleacea* HEID132425: *T. paleacea* x *T. streptocarpa*; *T. latifolia* HEID132151: *T. latifolia* x *T. purpurea*; *T. purpurea* HEID104854: *T. purpurea* x *T. latifolia*; *T. marconae* HEID103591/HEID131405: *T. purpurea* x *T. landbeckii*. Accordingly, we identified signals in eight *Tillandsia* species to be involved in interspecies geneflow. With 31 species studied for *Agt1* diversity, this equals 26%.

We can also conclude that *T. marconae* is the only obvious interspecific and reticulate epiarenic hybrid, which evolved from two wide-spread epiarenic parental species. And both of the epiarenic parental taxa, *T. landbeckii* and *T. purpurea*, are involved also in various other secondary hybridization events. In summary, we found direct evidence for secondary interspecies geneflow for six out of nine epiarenic taxa (67%) while considering also formation of triploid hybrids between *T. capillaris* and *T. landbeckii* as described recently by Stein et al. (2023) or detected herein (one accession of *T. latifolia*).

### 3.4 Genome size based ploidy estimates

Genome size estimates were used for obtaining an insight into ploidy level variation which may be used as an indicator for reticulation (**Supplementary Material 6**). The majority of estimates are provided for *T. landbeckii* (n=211 for population sampling, n=238 individuals in total). For this particular species the mean genome size over all accessions is 2.55 pg (2C-value) with a small standard deviation of 0.11 pg. The three individual populations fit also well and showed no unusual results (2C mean values [min./max.] for Caldera, Oyarbide and Arica are 2.49 [2.37-2.57], 2.61 [2.50-2.80] and 2.65 [2.36-2.73] pg respectively). At best, one could consider a slight increase of mean genome size in *T. landbeckii* from South to North.

Another 72 genome size estimates are distributed across 26 species. A mean 2C genome size estimate of 1.97 pg (range: 1.04-3.34 pg) has been reported for diploid taxa (2n = 2x = 48/50) by Gitaí et al. (2014). Accordingly, we sorted and grouped our genome size estimates with three exceptions: For *Tillandsia paleacea* we revealed two very different estimates: 2.42 pg for HEID132425 and 3.46 pg for HEID 132425. Accordingly, the latter accession is considered as triploid. For *T. purpurea* we also identified one individual (HEID131388-2) with a unusual high 2C value of 3.38 pg compared to all the other *T. purpurea* individuals. In the third case, *T. latifolia*, we also observed to contrasting genome size estimates of 2.09 pg (HEID132229) versus 2.99 pg (HEID132151), considering the latter accession as triploid, too. Our 2C genome sizes for diploid individuals ranges from 1.77-3.02 from a mean of 2.4 pg. In summary we can state that the majority of accessions and taxa were shown to be diploid. Tetraploid species were *T. capillaris*, *T. virescens*, *T. recurvata* and *T. retorta*. We found triploid individuals in epiarenic *T. latifolia*, *T. paleacea* and *T.purpurea*. *Tillandsia paleacea* requires more accessions for full characterization. The closely related *T. kirschnekii* is diploid, and we may take this as independent evidence that *T. paleacea* is involved in interspecies hybridization with species such as *T. streptocarpa* (s. above).

For the three population-level examples in the South (Caldera), Center (Oyarbide) and North (Arica) of the Chilean distribution range populations with mixed species showed very distinctive genome sizes of *T. landbeckii* (2.65 pg) versus *T. marconae* (2.32 pg) (Arica) and *T. landbeckii* (2.49 pg) versus *T. geisii* (1.77 pg) (Caldera) without any evidence for contemporary cytogenetic variation caused by hybridization and introgression, which is consistent with our genetic results.

### 3.5 Distribution ranges of epiarenic *Tillandsia* species

**Figure 6:**
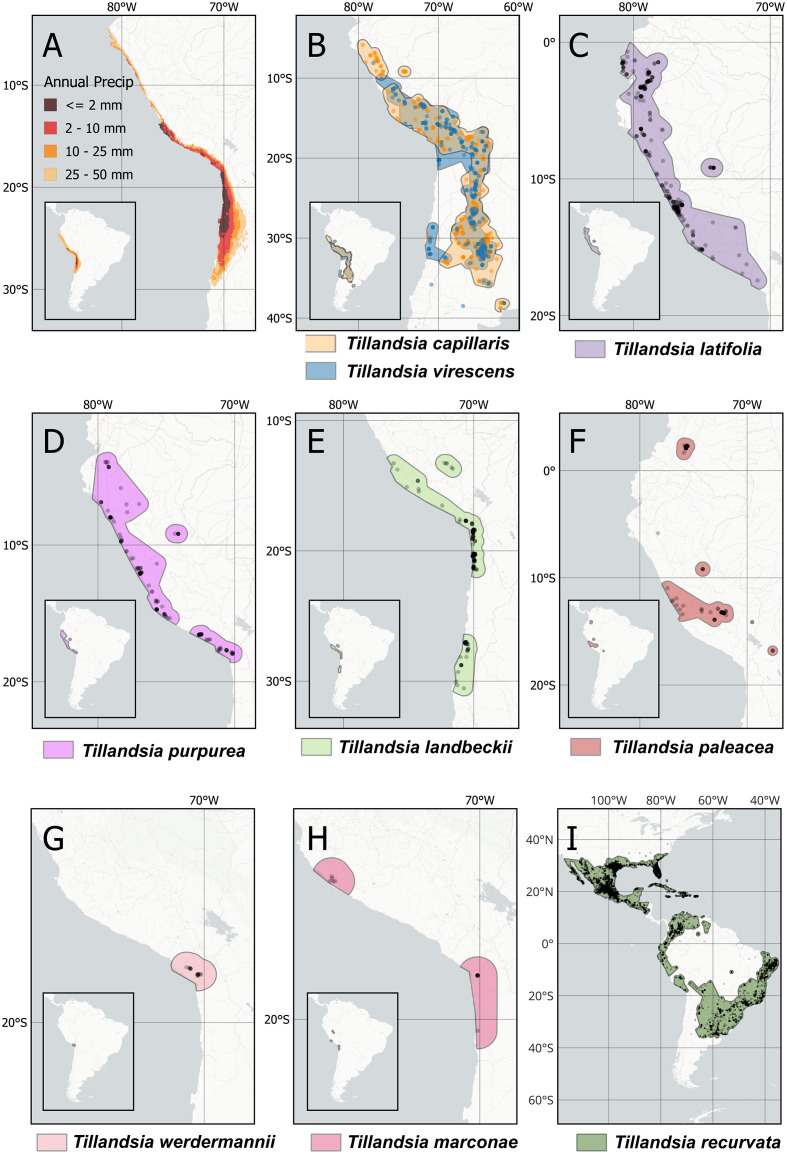
**(A)** Illustration of the arid (2–50 mm annual precipation) and hyperarid (< 2 mm annual precipitation) regions of the Chilean-Peruvian coastal desert system. The distribution maps of the nine *Tillandsia* species showing epiarenic growth are shown with with **(B)** *T. capillaris* and *T. virenscens*, **(C)** *T. latifolia*, **(D)** *T. purpurea*, **(E)** *T. landbeckii*, **(F)** *T. paleaceae*, **(G)** *T. werdermannii*, **(H)** *T. marconae*, and **(I)** *T. recurvata*.

The reconstruction of GBIF-based and further curated and corrected distribution range of the various species showing epiarenic growth are shown with (**Figures 6B–I**). The range of the Chilean-Peruvian desert system is shown with **Figure 6A**. The hyperarid part of the desert is indicated with annual precipitation < 2 mm rainfall per year, and the arid part is highlighted with the prepipitation range from 2 to 10 mm rainfall per year. Here the species show epiarenic growth. Accordingly, wider distribution of *T. capillaris*, *T. virescens*, and *T. recurvata* also indicates their more prevalent epiphytic growth form. In the case of *T. latifolia* and *T. paleacea* there might be additional taxonomic issues, e.g. unrecognized and misplaced taxa, or taxa of hybrid origin assigned to one or the other parent.

### 3.6 Distribution of CAM across Bromeliaceae

**Figure 7:**
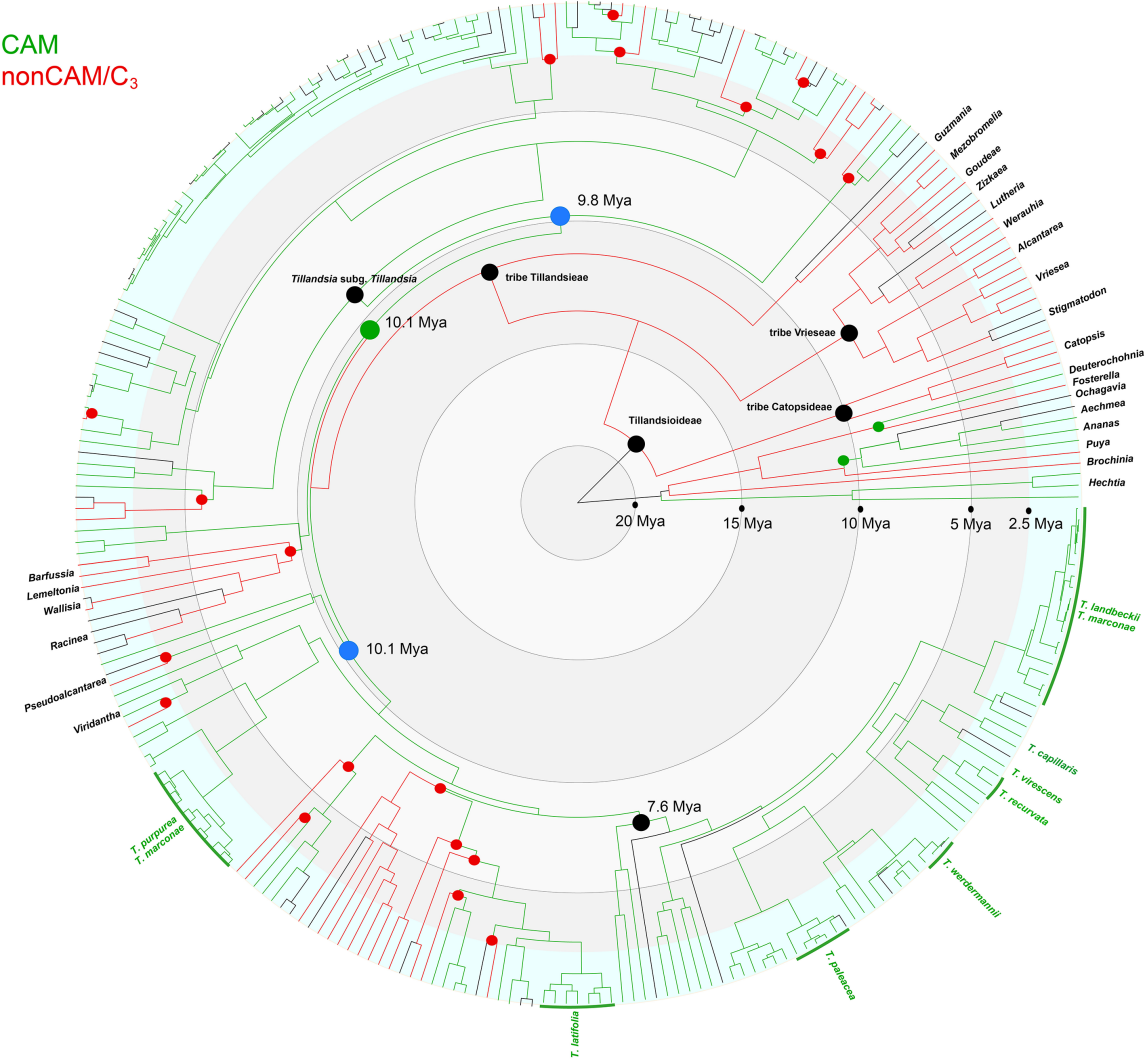
Evolution of CAM in Bromeliaceae with a particular focus on subfamily Tillandsioideae. Taxa with CAM photosynthesis are defined based on δ^13^C values and indicated with green terminal lines. NonCAM taxa are kept in red. Black branches are referring to missing literature data. Green dots indicate the origin of CAM. Red dots show putative and parallel reversals to C_3_ photosynthesis. Blue dots indicate parallel acquisition of specialised phyllospheric bacterial communities in atmospheric *Tillandsia* spp (Lyu et al., 2024). Epiarenic *Tillandsia* accessions are named accordingly. The *BEAST2* tree with detailed taxon designation can be found with **Supplementary Material 8**.

The results from plotting CAM phytosynthesis onto the time-calibrated *BEAST* phylogeny is shown in **Figure 7**. Considering uncertainty of placement of the root (*Brochinia*) in *BEAST* analysis the evolution of CAM in subfamilies other than subfamily Tillandsioideae is not possible. In subfamily Tillandsioideae CAM photosynthesis appears first in tribe Tillandsieae at approximately 10.1 Mya, shortly after the split from *Guzmania*. Numerous reversals to C_3_ are evident, but such reversals are rare and often found in clades not assigned to genus *Tillandsia* (e.g. *Barfussia*, *Lemeltonia*, *Wallisia*, *Pseudoalcantarea* or *Viridantha*). Such reversals are not found in clades containing epiarenic species.

## 4 Discussion

Over the past few years, mainly four milestone contributions have advanced our knowledge and understanding of Tillandsioideae and *Tillandsia* taxonomy, systematics, and evolution. As a starting point one might consider the conceptual work on adaptive radiation and diversification in Bromeliaceae as a whole (Givnish et al., 2014). As a first milestone a reliable and comprehensive taxonomic framework with well-defined taxa was presented by Barfuss et al. (2016), which laid the foundation for spatial and temporal frameworks to study principles of species diversification while assessing geographic origins and dispersal history (Vera-Paz et al., 2023). Although not fully relying on the first two contributions, the attempt by Lyu et al. (2024) to trace evolutionary signatures of major shifts towards epiphytic growth forms can be considered as a third major advancement in studying Tillandsioideae evolution. Finally, only recently our understanding of pervasive gene flow in *Tillandsia* was significantly broadened introducing a robust gene-capture dataset based on nuclear genomic information (Yardeni et al., 2026). Earlier findings estimated evidence for hybridization in ca. 11% of 147 species from 11 genera in the subfamily Tillandsioideae (Loiseau et al., 2021). And it was Gardner (1984), who also claimed very early that natural hybridization appears to be responsible for some portion of the morphological variation. Indeed, interspecific gene flow among various taxa can also be confirmed by the data presented herein. E.g., nested taxa are found within *T. landbeckii* accessions (*T. purpurea*, *T. marconae*) as the result of introgression and hybridization (**Figure 2**), the analysed *Tillandsia prolata* plastome is nesting within *T. werdermannii*, which may be considered here as an example of incomplete lineage sorting as consolidated by *Agt1* results (**Figures 3**, **4**), or two herein analysed *T. latifolia* accessions showing also footprints of secondary geneflow with *T. purpurea* (**Figures 3**, **4**). In summary, our herein presenting findings exemplified secondary geneflow across species in eight out of 31 cases (26%). And if we consider epiarenic taxa only, even six out of nine species (67%) (**Figure 6**) showed secondary inter-species geneflow. This increased percentage in epiarenic species may be best explained by shared biogeographic constrain and distribution (**Figure 6**) in the Atacama Desert and past migration dynamics and range shifts throughtout the Pleistocene (e.g. Stein et al., 2023): Additional temporal information on genetic interspecies connectivity among epiarenic taxa can be collected. As evidenced from our data we can exemplify introgression of *T. purpurea* into a female *T. latifolia* background (< 0.65 Mya), introgression of *T. paleacea* into a female *T. streptocarpa* background (< 491 kya), *T. marconae* with multiple (maybe polytopic; Bratzel et al., 2020) hybridization events in both parental genetic backgrounds (female *T. purpurea*: < 0.25-0.35 Mya; female *T. landbeckii*: < 138 Ma), and *T. capillaris* into a female *T. landbeckii* background (< 0.07 Mya). It should be noted here, that evidence from *Agt1* sequence variation may be biased by sample size (no. of accessions studied) and low sequence divergence (considering single nucleotide polymorphisms only). However, our results allow to define *Agt1* alleles with the respective clades containing epiarenic taxa (clades A-E; **Figure 3**); and with the chosen sequencing and cloning strategy we also got some additional information of potentially chimeric alleles (sequences obtained only via the cloning procedure), which we most often found as those alleles not placed into the correct clade (**Figure 3**). Of course, a single gene will provide only limited information, and it is also impossible with the given data to further explore putative shared ancestral variation and incomplete lineage sorting.

With the habitat and ecological transition from terrestrial, tank-forming Bromeliaceae to atmospheric and epiphytic Tillandsioideae (Lyu et al., 2024; Kessous et al., 2025), epiarenic *Tillandsia* can be regarded as the most extreme endpoint of this evolutionary trajectory. Arid, and in particular hyperarid, environments constrain virtually all resources to the limits of terrestrial plant life. This applies not only to water and nutrient supply and landscape structural diversity but also to substrate and phyllosphere microbiomes, as well as other biotic interactions such as plant–pollinator relationships. This raises the question of which key innovations among epiarenic taxa were already in place and which major environmental and biogeographical factors may have driven the onset of epiarenic growth in the Chilean–Peruvian desert system. Traits such as specialized trichomes, non-functional roots for water absorption, and CAM photosynthesis likely were already present in the tribe and likely facilitated colonization of these hyper-arid regions.

In a comprehensive survey of Bromeliaceae (1,893 species), Crayn et al. (2015) showed that the six almost exclusive terrestrial subfamilies consist either entirely of C_3_ species (Brocchinioideae, Lindmanioideae, Navioideae), whereas Hechtioideae is composed of CAM plants; in Pitcairnioideae, CAM representatives belong to a xeric clade comprising *Deuterocohnia*, *Dyckia*, and *Encholirium*, and in Puyoideae approximately 21% of *Puya* species studied exhibited CAM. In the subfamily Tillandsioideae, 28% of species possessed CAM photosynthesis, whereas in Bromelioideae 90% of taxa showed CAM (Crayn et al., 2015). Crayn et al. (2015) proposed that CAM tends to be the dominant photosynthetic type towards more extreme epiphytic and lithophytic “atmospheric” life forms, which has been also concluded by De La Harpe et al. (2020) and Lyu et al. (2024) and dates back to the idea presented by Pittendrigh (1948). However, CAM photosynthesis has been established very early in the evolution of tribe Tillandsieae long before the emergence of epiphytic and epiarenic growth form (**Figure 7**), and it has been shown that CAM evolution is associated with gene family expansion in early bromeliad evolution (Crego et al., 2024).

Epiphytic growth as a key innovation evolved early, at the base of Tillandsieae (Lyu et al., 2024), and transitions from epiphytic to lithophytic growth have occurred repeatedly throughout the evolutionary history of *Tillandsia* (Möbus et al., 2021; Lyu et al., 2024). Epiarenic growth therefore builds on pre-existing growth forms. Accordingly, another important key innovation is the exploitation of alternative nutrient sources. Evidence for the importance of microbiota for the fitness and survival of epiarenic *Tillandsia* in hyperarid, nutrient-poor environments has been provided by several studies demonstrating non-random microbiome composition in sand substrates and on *Tillandsia* leaf surfaces (Alfaro et al., 2021; Hakobyan et al., 2023; Jaeschke et al., 2024). A highly specialized phyllospheric bacterial community has been shown to occur in atmospheric *Tillandsia* and is capable of contributing to N_2_ fixation and nitrate reduction pathways (Lyu et al., 2024). The occurrence pattern of these specialized bacterial communities indicates that they evolved in parallel with independent transitions to atmospheric *Tillandsia* (**Figure 7**), with an inferred timing of around 10 Mya that coincides with the early radiation of tribe Tillandsieae after its split from *Guzmania*. Estimates in Lyu et al. (2024) are slightly younger (approximately 8 Ma), but calibration of their tree is based on consensus estimates and we consider these differences as insignificant. However, this discrepancy does not affect our conclusion that nutrient acquisition under extreme environmental conditions was secured well before the radiation of epiphytic and epiarenic *Tillandsia* species.

Plastome and *Agt1* results demonstrate parallel (independent) evolution of the epiarenic lifestyle in seven out of nine known epiarenic species when the hybrid origin of *T. marconae* from two epiarenic parental species (*T. landbeckii* and *T. purpurea*) and the sister relationship of *T. virescens* and *T. capillaris* are considered (**Table 1**). This highlights strong environmental pressure and evolutionary selection to establish this growth form and foster parallel evolution.

Stem-group ages of clades containing epiarenic taxa are placed either into the Late Pliocene and the Piacenzian stage, which extended from approximately 3.6 to 2.58 Mya, or around the Mid-Pleistocene Transition (1.25–0.7 Mya) in the case of *T. werdermanii*. The former interval marks severe aridification towards the end of the Pliocene in the Atacama Desert (Amundson et al., 2012; Ritter et al., 2018; Arens et al., 2024) and marks the transition from a warmer, more stable climate towards the colder conditions that characterized the subsequent Pleistocene. Whereas the latter coincides with a major change in Pleistocene climate that likely fostered aridity and, in particular, coastal fog systems (e.g., ENSO effects; Wilcox, 2025). At the same time substantial environmental change may have triggered migration and range shifts resulting in potential secondary contact (Stein et al., 2023). Therefore, we may conclude that major environmental changes likely triggered the emergence of epiarenic growth as marked by mean stem group ages (**Table 1**). If we consider crown group ages as minimum age of the realisation of epiarenic growth, then the majority of species diversified during the Mid-Pleistocene transition. Exceptions are wide-spread *T. recurvata*, *T. virescens* and *T. capillaris*, which also occur outside the arid/hyperarid Desert system, and here we may consider a shift towards epiarenic growth after the herein defined crown group ages. Accordingly, also in these three species the shift may have occurred later, e.g. during the Mid-Pleistocene transition. The clade containing all epiarenic species, excluding *T. purpurea* and *T. latifolia*, originated around 7.6 Mya. This lineage includes a well-supported assemblage of clades and taxon groups (*T. gardner* complex, “Incerta sedis”, *T.* subg. *Anoplophytum* s.str., *T.* subg. *Diaphoranthema*, *T.* subg. *Phytarrhiza* s.str., *T*. subg. *Aerobia*; Vera-Paz et al., 2023) with basal biogeographic associations to the Chacoan subregion and an even stronger association with the South American transition zone along the Peruvian–Chilean distribution range for all epiarenic taxa and their sister species. Hence, we expect a biogeographical signal for the evolution of epiarenic growth, where predominant shifts of lifestyle in sister species occurr in close biogeographic proximity to arid–hyperarid habitats.

In summary, we conclude that sister taxa and progenitors already shared crucial key traits. Additional insights into epiarenic growth and relevant characters may be gained by examining species’ biology and biotic–abiotic interactions. A first striking feature is longevity: under hyperarid conditions at the limits of plant life, *T. landbeckii*, for example, is estimated to reach ages of at least 50–100 years (Koch et al., 2020), and monospecific populations and vegetation may have persisted for thousands of years, as indicated by fossil *Tillandsia* dune systems (Latorre et al., 2011). Second, epiarenic *Tillandsia* species exhibit growth rates that are tightly coupled to regional and local sand dynamics, ensuring a steady state of sand trapping and oversanding that allows plants to grow in coppice dune systems without a root system and to anchor themselves via old, dead shoot–leaf structures in otherwise volatile sand substrates (Schweikert et al., 2026). This indicates that epiarenic *Tillandsia* species must modulate their interactions with abiotic factors such as wind, sand, and fog (as a water source) at both individual and (mono- or oligospecific) vegetation levels. Longevity maintains vegetation integrity and compensates for high seedling mortality and unreliable pollination (Koch et al., 2019), but at the cost of reduced gene flow and consequently lower heterozygosity and genetic diversity, which may limit phenotypic variation and plasticity which is needed to cope with environmental variability under extreme conditions (Koch et al., 2019). This apparent dilemma is resolved through predominant vegetative propagation and local selection on genotypes with higher phenotypic plasticity that are distributed non-randomly across populations, thereby contributing to long-term population and vegetation integrity (Jabbusch and Koch, 2025).

In this context, past gene flow is illustrated by interspecific gene flow among epiarenic *Tillandsia* species in various combinations, particularly in species with wide distributions, overlapping ranges, and occasional co-occurrence in metapopulations as illustrated here by *Agt1*. Most prominently, this involves the geographically vicariant *T. purpurea* in Peru and *T. landbeckii* in Chile, whose latitudinal ranges have shifted substantially since the Last Glacial Maximum (Stein et al., 2023). Other species, such as *T. capillaris* and *T. paleacea*, also contribute to a pervasive gene pool, a phenomenon recently documented for *Tillandsia* subg. *Tillandsia* (Yardeni et al., 2026) and interpreted as evidence that interspecific gene flow facilitates explosive diversification. In our case, we propose that this genetic configuration confers adaptive capacity by maintaining genetic diversity and phenotypic plasticity required to cope with environmental change in an arid–hyperarid desert system with narrowly defined ecological niches. Phylogeographic analyses of *T. landbeckii* also revealed genetic structure along the entire latitudinal range that reflects past gene flow and range dynamics rather than ongoing gene flow (Merklinger et al., 2019; Koch et al., 2022). These characteristics collectively render the epiarenic species unique and particularly remarkable. In our study we found only rare evidence for ploidal level variation in hybrids, and, accordingly, we may tentatively conclude that homoploid evolution is the prevailing mode of gene flow across species.

## 5 Conclusion

This study characterises the parallel evolution of epiarenic growth in Tillandsia species restricted to the arid–hyperarid desert system along the Peruvian–Chilean Pacific coast. Phylogenetic and genotypic evidence reveals multiple parallel and convergent evolutionary events, in which major key innovations such as the transition to CAM photosynthesis and the establishment of a specialized phyllospheric bacterial community predate the onset of epiarenic growth. Accordingly, biogeographic features and traits including longevity, vegetative propagation, dispersal, and a distinctive genetic population structure are discussed as major factors underlying the emergence of epiarenic growth and its long-term persistence.

## Data Availability

The raw chloroplast genome sequences were deposited in GenBank under accession numbers ERS23821205-ERS23821238. Agt1 sequence data are found with accession numbers PX713484-PX713568 and PX987240.
