# Supplementary material for: The different routes of parallel evolution in epiarenic growth in a hyperarid desert environment
Source: Front Plant Sci. 2026 Jul 7;17:1822909. doi: 10.3389/fpls.2026.1822909 (PMC13392990; doi:10.3389/fpls.2026.1822909)
Supplement: Supplementary file 3 [file SupplementaryFile3.docx]

**Supplementary Material 003:**

**Nexus file of the plastome alignment used for phylogenetic reconstructions.**

# Alignment of plastome sequences for 278 taxa

#NEXUS

BEGIN DATA;

DIMENSIONS NTAX=278 NCHAR=117324;

FORMAT DATATYPE=DNA GAP=- MISSING=n;

MATRIX

8113172 atggaaaaatggtggttcaattcgatgttgtctaacgagagaacataggtatggtttaagtaaatcaatggaaagtcttgatgctattggccataccagtggaagtgatgaaccccttctaaatgatacggagaaaaattggagtgatagtttcagtaatgttgattatttatttggtatcagggatatttggagtttgatctctgatgacacttttttagttagggatagtaatggtgacagttattccgtatattttgatattgaaaatcatagttttgagattgacattatcattacatgtatgatactcaatctagttggactaatcaattaatagttccatttcaggtggtactgacaatttctgaaagttttagtataagaactcgtaataatggcagtgatttcaatataagaggaagatctaatgatttcgatataaataaaaaatacagacatttatgggttcaatgcgaaaattgttatggattaaattataaaaaacttcttaggtcaaaaatgaatatttgtgaacagtgtggatatcatttgaaaatgagtagttcagatagaatcgaactttcgattgattcgggcacttgggatcctatggatgaagatatggtttctatggaccccattcaatttcattcagaagaggaaccttataaagatcgtatcgattcttatcaaagaaagacaggtttaactgaagctgttcaaacaggcataggtcaactaaacggtattcccacagcaattggggttatggattttcagttcatgggaggtagtatgggatctgtagtaggtgagaaaatcactcgtttgattgagtatgctactaatcgatctctacctgtcattattgtgtgtgcttctggaggagcacgcatgcaagaaggaagtttgagcttgatgcaaatggctaaaatatcttctgcttcatatgattaccaatccactaaaaagttattctatgtaccagtccttacatctcctacaaccggtggagtaacagccagttttggtatgttgggagatatcattattgctgaacctaatgcgtacattgcatttgcgggtaaaagagtaattgaacaaacattgaataagacagtacccgatggttcacaagtggctgagtatttattccataaaggcttattcgacccaatcgtaccacgtaatcctttaaaaggtgttctaagtgagttatttcagctccatggtttctttcccttgaatcaaaattcaATATTATTTTTATATATACTTAGTAGTTAATATTACTTATAATAGATATACTTATCATAAGATATCTTTCTAATATACAAATATTAAATCGAGGCACCCATTCTttatggttgttcctgaagtagaaattgtttgatctgttcctgaatagcttccttcaaaacggcttctgcttgctcggtgaatgtcttggtagaagatataatttcttggaattgaggtttattcttttttaagtaagtacgtaactgaacaagaaatttctttacctgtccaatttctaacggatcaagatatccattcgctccggtataaatagtagctatctgctcttccaccgtgagagggtctgattgggattgtttaagcaactcacgtaatcgttgacctcttgccaattgattctgagtagctttatcgagatcagaagcaaattgtgcaaaggcttctaactctgcgaattgcgctagttccaattttgatttgccagctacctgtttcatggctttaatttgagctgcggatcctactctggaaacagaaatacccacattaatagcaggtcggattccagcattgaatagatcggcggataagaatatttgtccatctgtaatggaaattacattagtaggaatataagctgaaacgtctccagattgagtctcaactattggtaaagcagtcatacttccttcacctaaactagaacttgatttagcggctctttccaaaaggcgtgaatgcaaataaaaaacatctcctggataagcttcacgaccgggaggtcttcttaatagaagagacatttggcgataagcttgtgcctgtttggagagatcatcataaattattaaagtatgtcgttcacggtacataaaatattcagccaaagtcgctcccgtataaggagcgaggtattgtaatgtagcaggtgaatccgccgtttcagctacgacaatagtgtattccatggcccctcgctcctggaaagtagtcactacctgagccacagaagatgctttttgaccgatagctacataaacacatattacattttgccctttttgattgagaatcgtatctgtggctactgctgttttgccggtctgtctgtccccaataattaattctcgttgaccgcgtcctataggaatcatcgaatcaatagcaataagccctgtttgaaggggctcatatacagaacgtctcaaaataataccaggggcaggggattcaattaaccgagattcataagctgaaatttcccctctcccatcaataggtgtagccaaagcatttataacacgacccaaataagcctcactcacaggtatctgagcaattcttcctgttgcttttacagaacttccctcttgtatcatcaaaccatcacccattaatacaacgccaacattattggattccaagttcagagcaatgcctattgtaccctcttcaaattctactaattcacctgccattacttcatcaagaccatgaatacgagcaatgccgtcgcctacttgaagtacggtaccggtattcacaatctttacttctatattatattgttcaatacgttcacggataatattactaatttcgtcggctcgaagggttaccattagtgtttctttattctttttcgaaagcaaaggtaaaaataatgcctaaattgtaaactaaagtaaaagtgtcatttcttcaaattgctctccacttctaagttcatagctttcgcggtagcttcatcgatgttacccaccaaataaaaagcctgctcgggcagaccatctaattctccggaaaggatcagttgaaaccccctaattgtttctgcaagaccaacatattttcctggagaaccagtaaatacttctgccacgaagaagggttgtgataagaaacgctcaatttttcgtgctcttgctacagttaaacgatcctcctcggataattcatccaacccaagaatagctataatgtcctgaagttctttgtaacgttgtgaagtttgcttaactctttgcgcagtttcataatgttcctcaccaacgatccgaggttgtaacatagttgacgttgaatctaaaggatctactgctggataaatacccttggcagctaatcctcttgatagtacggtagtagcatctaaatgtgcaaatgtagtggcaggagcagggtctgtcaaatcgtccgcaggtacataaactgcttggatcgaagttatagatccctctttggtagaagtaattctttcttgcaaagaacccatttctgtactaagggtaggttgataacccactgcagaaggcattctccctaataatgcggatacttctgatcctgcttggacaaaacgaaagatattgtcgatgaatagaagcacatcttgttcattaacatcccggaaatattctgccatagttagggcagtcaaaccaactctcatacgagctcccggcggttcattcatttgaccatagactaaagctacttttgattctgcaagatttttttcattaattactccggattctttcatttccatgtaaagatcatttccttcacgagtacgttctcctactccgccaaatacggatacgcctccatgagctttggcaatgttgttgatcaattccatgatgagtactgttttacctactccagctcccccaaatagtccgatttttcctccacggcgataaggagctaaaagatctaccaccttaatacctgtttcaaagattgataatttcgtatctaactgtataaaggcgggcgcagatctatgaataggagatgttgtgcgagtatctacaggacctaaattatcaacaggctctccaagaacgttgaagattcgaccgagagtagctccgccgactggaacacttagaggagctcccgtgtcaatcacttccattcctctcatcagcccatctgtagcactcatagctacagctctaactcgattatttcctaataattgttgtacctcgcaagtcacattaatttgttgaccgactcgacccttaactaccaaagcgttataaatattaggcattttgcccgggggaaaaacgacatccagtactgggccaataatttgagcgatacgccctagttttttttcttcaagtgtggaaaccgcagggctagaagtagtaggattgattctcataattataataaagtgaaatatgtcgaaatctttttggaataataccaaatcaaaataaatgtccgatagcaagttgatcagttaattcaataagagataaatgggagatagctttttcatatagatttccgtctttatattataccctttcgtagatgaattatgcttattttcacatctaggatttacatatacaacatatattactgtcaagagggaattttctcagtatttagattcaaaataagaaagggttcaattataaacccgcaaaagattaggattgggttgcgctatatatatcaaagagtatacaataatgatgtatttggtgaatcaaatacatggtctaataacaaaccattttaacataacattttgttgataatattaattgaatattttttgaaagatttttgttaaaggtttcattcatgcctaatccatatcgagtagaccttgttgttgtgagaattcttaattcctagtaggaaataacattgacagcctctactcgtgtcctagctcgtctgagagctagattcgcttcaatcacttgtctcttaccctcagctctactcaagttagcttcagctattttaagagtttgttgagcttcttgcggatcaatgtcagtactcatctccgcatcatttcctaaaatggtgatctcattattccctattctagcaaaaccacccatcagagccaccgttaaccattggtcgttgaggcgtattctcaaaagacctatatctacagccgtggcaataggggcgtggtttggtaatacaccaatttggccactatttgtagataaaatgatttctttcacttctgaatcccaaataattcgattaggagtcagtacacaaagatttaaggtcatctaatcagttatttcttccatggccccaagaatgccaatattagcacggatcgtacggaaatgtaactcggtattcaaacaactattcagagttcctagagctccttgtaaggcttgttggaaaactcgttgtcggacttgatttatcgctctttgttgttcaaaatgaagggtttcatttttgaaattttctaatcgttccaaactatcacaagtagctttaatcaaattttctttttctcgttctatctcagagtatccattcattcgatactcatctgcttccagttcgactttctgtaagcgaacccgggctttttcgagctgctcaatggcccttctacgtaattcttccgaatttcgaatagtactcaagatcctctgttttcgattatctaataaatcatttaacactccctttccaaaaaaaatcaatacaccaatcactacacttagatttattggatttgttgctaaaatatcggtattaaacccaaaacccccggcggatggccaatggcgtgagaaaacgaaagaatcggttacatttttcatatgctttcctcttatagataggactgacaaagaacaaaatttttttctattacttcgctcttttttgatcaatttatttttaattgaatttcccctttttatgggaatagattaaatctagtaatttcatttaggttaggtcttaggtctcatttcaattgtgaaatatatcgtttgtggaaacgtttcctaaaaggaaaaagtttccattgtactaagctaaggacgggaaggaagaaagcgagtgatctggtaattcctcatcctcaaagcagtccttcctgtagtctcaacaaataagtaattataggagtaattgatataattcgaagaagcaaacgattcaagttaataaaaaggtacttttttacattttgaaagtagattatcttaccattaatttcacaactttcatgatctcttcccgaaccaaacatgaatctttcgattcatttggctctcacgctcaattatttattttattttttgttatgggtattcccatattttttatgtaatgagcctgccctctcttttctgtttgtattcaaagatatctaaactgatacaagaccagaataaatattaggaggactcttccgaccagataaaaatcaataattgtcagcaaagttgtttctttatttgtatccaaaaattcctcttttttatacataggtcgtcgattcggcattggataaaaaaggcagagtgccttttctttctagtaaatggttcaaatccttttatcgatatgagtgttctatatcagataaattaccaactattcattttgaaaacatttcagtactaatgtagtcgtagaaagagtaccatgttttgcctggacttcaaacagtttagctttaaccatggtaatggtctcacattattggttgatagagaatcaaagttgatttaccaataagtcacgaaatgctatggttcttacatatgatttttgaatttattcagaagtaattcgtcgagatcgtgcaccttttttcctatttatcctaataactataaataaagtaaagtgcagccggatggatccaacctattcttgaaatacacaacccgcacttaaacaaaaggattcgcaaataaaagcgctaatgccacaaccagtccgtaaattgttaaagcttccataaaagctagactaagcaataaagtacctcgtattttaccctctgcttctggttgtctcgcaataccctctacagcttggcctgcagcagtaccttgaccaactccgggtccaatagaagcaagtcctacagccaatccagcagcaataacggaagcggcagaaatcagtggattcatctcacaaaaagaaatggttaatgatacaatcaaccaatgaattattacttaattttatcattaagatctattgggtcggagtaactaaaaattaatgataatattactgaatcgtcagaactacttcgatatctcattttttgtttctacccatgatgaagtttttgtaaatccatatcggctctagttattgcatttctttccaaccattctttcattccatccttcgttctttactcttctatatccttgagttcatctacacaatcacaaatgaaacagaagaaaggacttgacttatcttgtaatccatctaatctaaatgcagtaaactgcaatcaaatcaatatatgcatatgatcgatatctatatatagataggactatataactagtgaatatctattacatttctttcttccataacgtcacattttatattgaatcggattataaatcattcctcgaaacccacacaaaaagtggctggacttatagacattacatacatctagtgtgacctccccaacctttttttaattccgtaatatcgttcatcttctctcctacgactctaggtcatatattcatatttatatctattatgttctccaaccaagcagattatcttgaaccatgctgggataagctaaaaaaaagactatttagaaaactagtcaatgatgaccctccatggattcgcctatataagccgcggctaacgttgcaaaaataagagcttgaataccacttgtaaataatccaagaaacatgacaggtataggaactactgaagggactaaagaaacaagaacaacaactactaattcatcagccaatatattcccgaaaagtcgaaaactaagagataagggttttgtgaaatcttctaggatgttaattggtaaaagtattggagttggtttgatgtatttctcgaaataacccaacccttttttggtaagacctgcataaaaatatgccactgacgtgggtaaagctaaagcaacagtagtatttatatcattcgtgggcgcagctaactccccatgaggtaactgtatgattttccaaggtaaaagggcacctgaccaattagaaacaaaaataaataggaacatagttccaataaagggaacccaaggtccatattcttctccaatctgggttttgctcaagtctcgaataaattcaaggacatattcaaagaaattctgaccgtcggtcggaatggtttgtggattccgaacagctatgatggctgaacctaacaagatagcaattacgacccaagaagtgataagtacttgggcatggatttgtaaacctcctatttgccaatagaaatgttggcctacttctacacccgatatatcgtataaccccttgagtgttttaatgtaacatggtataacattcatattgtcctctgatagaaattgaacttcaaaaaaggaattagtttgattcaaccttctcaactcaccaacttgaatcatttatttaggataccaagaaatcacataacatcataatatatatcaatatccccagttttttttatcaaaaaaagtaaccgatccaaaattaacattaactaattttattattcttaaatgatatttgcgactttagaacatatattaactcatatctctttttcgatcatttcaattgtgattatgattcatttgatgaccttattagtccacaaaattgtaggattacgcgattcatcagaaaaagggatgatagctacctttttctctataacaggattattagtttctcgttggatttcttcgggacattttccattaagtaatttatacgagtcattaatcttcctttcgtgtagtttcttcattattcatatgattcccaagatacgtaacgatttaagcacaataattgcaccaagtaccatttttactcaaggctttgccatgtcgggtctttcaactgaaatgcatcaatccgcaatattagtacctgctctacaatctcagtggttaatgatgcacgtaagtatgatgttattgagctatgcagctcttttatgcggatcattattatcagtcgctcttctagtcattacatttcgaaaaaacatcaaaatttttttaaaaagcaataattttttaattaagtcatttttcattgaatatttgaatgaaaaaagaagtgtttttaaaaacacttcatttcgaaattattacaaatatcaattaactgagcgtttggattattggagttatcgtgtcattagtctagggtttacctttttaaccataggtattctttgtggagcagtatgggctaatgaggcatgggggtcctattggaattgggaccccaaggaaacttgggcatttattacttggaccatattcgcgatttatttacatactagaacaaatataaattggaaaggtacgaattcggcacttgtagcttctataggatttattataatttggatatgttattttgggatcaatctattaggaataggtctacatagttatggttcattcacataaacatctaattgaataaactacatgaagaatacataagataaaaaatcatcccatatataacgaaagtttttatgagtttttgagaaccatttaaattaatgattccttgaaatggttctcaaaaactcgagatgtatctaattacaattattattcattttatttcttttttcattatacagtacagcaaacgattttccaaatattaaattcaaagaattatatttccttccgtttcattaatgaaacaatgaaaaaaaagaaagcattgccttctttcccatatcttgtatctatagtatttttgccctggtgggtttctctttcatttaataaatgtctggaactttgggttacaaattggtggaataccgggcaatccaaaactctcttgaatatcattcaagagaaaaacgttctagaaagattcatagaattagaagaactctttctgttggacgaaatgataaaggagtacccggagacacatatacaaaaacttcgtataggaatacacaaggaaacgatacaattggtcaaaacacacaatgaatatcatctccatatcattttgcatttctcgacaaatataatctctttcgctattctaagtggttattttattttgggtaatgaggaacttgtcattcttaattcttgggttcaggaattcctctataacttaagtgacacaataaaagctttttcgattcttttagttactgatttatggattggatttcactcgactcatggttgggaactaataattggttcgttctacaatgattttggattggctcataacgatcaaattatatctggtcttgtttccacctttccagttattctagatacaattgtgaaatattggattttccattatttaaatcgtgtatctccttcgcttgtagtcatttatcattcaatgaatgaatgaagaactcatttgatctcctgatatcaatcaaatcagaatctttctttataaaccattttgaatcttacttaattctttatatttctaccagttcaaggtattcgtcctatattacagtacaactattccagtacaatgacagactcgtgcatagggaactttactagttctctatctaatttattgtagaaattccgagatccatgattggacttcgaccgctacaagatcaacaatgccatgagcttgggcttctgttgctgacataaaaacatccctttccatatcctcggatacaacccataaagggttgcccgttctttgtacataaacctttgttatggtttcgcgaagtttcagtagttcttccgcttccaggataaattcccctgcttgtgcctcataaaaagaactagcaggttggtgaatcataaccctagcgtgagggaatgctagacgtttggtaatttctcctccgaccagaataaaagatcccattgacgcggctaatcccatgcatatcgtatgcacattaggtgacacaaattgcatagtatcataaatggctattcctggtattacccatccgccgggagagtttataaacaaataaagatccctagtaccatcttctatactgagatataccatgagaccaacaagttgattcgagatctcgctatcaaccacttgccctaaaaaaagtaatctttctcgataaagtcggtttacgtcaatccaaaccgcatcttcctctccaggactccgaaaaggtacttttggaacaccaatgggcattaaattaaataaaaattgagtactatacttcactttaatatggaaacgtaacaatcaatggtttattgtcttcatcccttttttatctgattggaaagtttatagagtaagacagagaaatcgatcgttcgaatgataaacaagtatctatccattcgtttcccaaaaatgggaccaatcctcccattgcgtattggtacttatcgggtatagaatagatctgcttctctttgttcttacgaacaaaattgttctttcaatggaatggaataaatattaatcctttctgatacggaatctactgaaattaggtacatggtatatatagtcttttccaatgcgataaaataaagcggcatagtgtctatttttctttgataaagaggtatttccgatgatattgatataacggttcttctatctcgcatgattgggctaaactaaagtaggggataaaaaataacaagaaaaaaaatagaattgaataaccgtacaggcatcttttgttcattgcatacggctccgcaatggaattgactttttcttctcttctattctatcgaagaaagaaatagaatccatctaatccagatcgttgaatgatccatttaccacccttcctttcatagaagtaaaaaagaatactatgatggttctgttactttatatatttatctcgtctgtgatttagcaatcccaaagtttctttttgatacgatcaaataagatttttcgtactctttctttcatagcataagtatcagaaagagacttctggtgtggaagaaaaatggtttgtgacactgaaatgtactcccgacacataagtcaaatcggaaataacctttatttcatactactatctcgatacaaaatctcatgttatgaaaaacaataatggtttgttcatatcgaacccgaagtgccatgctattattacttataattttcttttataatcaatgtggcgaaggcatagtctttttttcaaataaaaactcattggcgccagaaccgtacgtgcacctttggatacatacggttcaaaaaagaaaaagaatcaatgtgtcgattccagttttattttnnnnnnnnnnnnnnnnnnnnnnnnnnnnnnnaaaaaaaagagattactgaacttattaaactaacctatcattgatgtattgtttcatcgatattaaaatcacgatgtcattgtcttgttcctgaatgggccctttcaattcttttaggttcatggtctaccccgggaaaagatatgtccgaattctatttgcacatataggacaaatggtctcagtaccatatcactacagtaatagtaggtataagaatcatttatgatacaagtggtaatcatacatatcaatttgttatcgatttggtattttctgaacggagcctggatactttattttatcagtccaagtaaaccataaattcttctaaattgataatattgatcccaaataaattgatctaattgcacttcacgctccgaatgattgattgatgcctcactcaatacaatatctcttgggcgaaacagaggatatctcgatcaggggagagaacgggtaaatcccatatgacccaatatgtatgacaagtcgcacctaatcgttcgaatctttgttgcgaagtcgataaattatacgtcccctggttgaatcataacgacttacttcaatttttactctatctcccggtagtatccgtataaaactgcggcggatcctccctgaaacatatcctagaattagatcttcattatctaaacggacccggaacataccgttgggaagtgattcagtaattaaaccctcatgaatcaatttttgttctttcattccaggtaacctccttgaagtatcaactaatggaggaagagttatataactcacttttcctctctattttacaaataggaagttagaaaattcggataccagaggtggaagatcaatgattcaccaggttattgatacggataatatccaaataccaaatacgttctcgacgtgatccatgtaaagaaaaaggggttggtttggggaagatcaaagaaagaacttgttcttcttccgtaaaaaattcttctaataataccgaacctaatctctgcaaaaaagagcgtaccgtactcttatgtttacgggccaaagttctagcacacgaaagtctaagtatatactttactcgatacaaactccgtttttcggaggatccactatgataatgagaaagatttctacatatccgaccaaatcgatcaataatatcacaatctgataaatcggtccagattggcttactaataggatgtcccgatacggtacaaaatttagctttagacaatgatccaatgagaggaataactgggactatggtatcgaatttcttaataccagtttctattagaaatgaattctccaacatttgattccttaccaccaaaggattttttagtacacttgaaagataacccagaaaatagaaggaatagtttgataattggtttatatgaatcctgtgtggttgagaccaaaaattaaaataatattgccatagattgacaaggtaacatttccacttcttcatcaaaagatgagtcccttttgaagccagaattgcttttccttgatatcgaacataatgcatgaaaggatccgtaaaaaaccataaagttttctgaaaatagttatggtacactactataagatgttccatttttccatagaactgtattcgctcaagaaaggttccagaagatgttaatcgtaaataagaagattgtttacgaagaaaaacgaataaaaattcgcattcagatacataagaattatataggaattgaaatagtcttttattttcttttgaaaaaacataaattgatttcttcggagtaatgagactattccaattatgatattcgtggagaaagaatcgcaataaatgcaaagatggaacatcttcgatccgacattgaaggatttgaaccaagatttccaaatggataggatagggtattagtatatctgacacataatttaaatgtaataatttgtcctctaaaaagggaaatattgaatgaatagatcgtaaattatgacattttggtatttctttttcttcgggaaaaggtactaatcgcagcgagaatggaatttccacaatgaccgcaaaaccttctgatatcatctgagaaaaaaaatgagaataaaaataattgttgtgcccaacgaatcgattttggttagaataattaaccgaataaattaaaaaattctgttgatacattcgaataattaaacgtttcacaagtactgaactagatttattgtcataaccaaaaatttccacgggttcgtaaaaaatcgaatcgtttaaaccatgatcatgagcaaacgcataaatatactcctgaaaaagaaacggatataggaagtgttgttgccgagatctatctttttctaaatatccttgtaattcttccatttacatttctacttgagccagaggcagtaggttttcttggtttatcaaatgatacatagtgcaatatggccagaacaggtaaagaaaaaatatataccccggaaaagaaagagggagtccaatcaacagatctttttaccttttctatccaattggtttatgttcgttataattacaacaggaaaaatcctttatttttgcaacccaatcgctcttttgactttggaataaattctctttatcaatatactgtttcttctacacatctgtctacaatccataataaagaataattaggattctggttcactcacaggagaacccactctttcccgcattaggcactaattcatttttaacatctaattagatcgggtaatcattccaattaagaacataagctcgttgctttttattttaccagaattggagccatagagctctatccatttattcactagacccaactgtaaatgtgaatttattttgttcccttccaaaaatcataattgattttattacgacatgctgttttttccattcattacccttgaggatcagtcgtggtcttatagactataccaatagtctggacgaattcgttgcttcatccaaatgtgtaaaagatcatagtcgcacttataatgaaagaagttgggaagaagttgttaataacagattacctagagaaataggtaaaagaaatttccatccaagatttaataactggtccattctcattctaggtaaagtccatcttgttgtgatagaaatgaagagaaacaaataagctttagttaatgtaataaggatacccattgtcattccaaaaatgctggcgattttttttattccgaaaagctcagaaatggatatatacggaataggtaaattccacccacctaagtaaagaactgttacaaataatgaagaaactaataaatttaggtaagaagcaagataaaataaaccatatttgatacccgaatactcggtttgataacctgctactaattcctcctccgcttctggtaaatcaaagggcaatctctcacattcggctagagaagaaattagaaaaacaataaatcctataggctgacgccacagattccacccccaaaaaccatattttgactgtgcttcaactatatcaactgtacttgaactgttagataatagagatattgataacacacatagagttaatggtatttcataactaatagattgagcagcagctcgtagaccacctaaaaaggaatatttattattcgatccatatcctgacataagaagcccaataggagcaatactagaaatggcgatccataaaaaaacacctatactgagatcggctaaaacaagacgatacccaaaaggaattactaaataacttagtagaattgatataaccgctatagacggtcccacactaaacaaacgaatatctcctcgagatgggaggaggtcctctttaaaaagtagtttagtcccatccgctatagcttgaagaattcccaacgggccagcatattcaggcccaatacgttgttgtatcgctgcagatatttctctttctaaccacacaattactagtacccccattgttattcccaatataagggtcaaaatgggtacaatccatattagtccatacacttcttttaaaaattccgatgtataaaaagaattgatagtttgtacttctgtcgtatcaattatcattcatagtcgataataacatcactgttcccatcgctatttcaaaaccgtacgtgagacctcagcttcatacggctcctcaatggccacaaaaataaggatgggttcggtattatcaccatctttggatggatagaataaagatacatcggaaagtcccaaattagaccaaggaattctgtctgctagaataagaaaaaagtgcttccgaattgatctcatcctttacaataaaaatttctctttgttcggtaataacttaatatttcaataaaatactccttatatcaatcaatacaaaataaaaagaattagtcattagttcatgaatcgtgataagaattcaatatgtatgaatatggatagagaaaagaataaataaggatccctttttttattattcattcaattactacattccatttcttttttcctgttcttctgtctcagaggaggatactaaaataaagaattaattcgttcttgatagtcatttctttaaccagtgaataggagcatactctagatcggaatcgtagggaagtactacttgatcatttctaccaatttaaagtccttattatgattcgttttatgaagaaatacctcttttttgataccctacattatctccattactaatcctttgtgtaccttggtgttcctaaccgtccactggcttttgattgatccccggtatagtaatacatatgagacagtagtagaaactccatacagttgatcttttgtttgcgcccgcttcaagatatgatgactaatcaaaaaatcttaatcttggggtaagcagtttacactgcttatttttacttcaactttattcttgtacatagggaatgagattgtttcttcttactacaaattgggaagctgtttagtttcactcatataactatctggtttaactcatcaacccgaatgctgaataaaaaagaaaacaatacattgaacctctttcttttttcttttatttctagaagagttcatattccaacgaatcacacctaaaagagggtatcctgagcaattgcaagaattgggttcattgatattcctggtatagtagatgctatcacacatacagtcatactcaattcgatggaattgtttgatcttaaaggggatcttctataatttcgcacgtaaggggttatttcttggtttcgtccagtcattaataacttgattatttttagataatagtagatagaaacaacgctcgtaaggagtcctattgaaaccaagaaatataggcctgcctgccatccacaccagaatagatagagttttccgaaaaaacctgctagtggaggaagacctcctagggataagagacatagggctgaagagagagccaaaaaaggatctttcgtgtataatcctgcataatctcgaatgttatcagttccggtacgtagaccaaataatacaatgcgagcaaaagttcctagattcatggagatatagaacagcatataagttatcatgcttgcatatccatcatttgagtctccaacaattattccaataattacatatccgatttgaccgatggacgaatatgcaagcatacgtttcatgcttgtttgagtaatagcaatgagattccccaatatcatgctaagaatagctaggatttccagaagaagatgccattcgtttgatgagaaataaaaaggaatatcgaaaattcgcgtggctgaagctgaagcagctacttttgaagtaacagaaagaaaagcaacgactggagtgggagatccttcgtatacgtcaggagtccattgatgaaaaggggctggggaaagcttgaacccaattcctacagtgatgaatataagcgcaattgaaattcctggggagttatacatttgtgtattgataagaccattcactatttcttgaagctcgatctcccccccagatgaaccatatagccaagagaaaccatgaaccagaatagaagagcttgccccacccataagtaaatatttcatagtagcctcattagaccgtacatctctcttggtatatccggataataggtaggaacataaactgaaacattctggagctacaaagatagttattaaatcgttagcaccacataaaaacattcctcctagagtagctgttaatacgaataacagaaactctgttatagccatttctgtacattcaatgtactctacggatagaggaatacatagagttgaacatagtaaaataagaaattgaaagatttcgttgaaattgttcgtttggaaatttcccgaaaagctaattataggttcttctctccatcggaacaatagggccgttatgcttattactaaacttgttgaagagatgaaatagaaccaaggtctatctttttgatcagaggttgaatcgatcatcagaagaagaattagaccaaaaattaggatacattctgggaaaatgaaacttccatggaagagaagcaaatgaaacgctttcataaaaattctcgtagaatcgagaatgaagttttcattctgtacatgccagatcatgaattagtaactgcatccaatctccgaaaaagtcccaattgtttcgaactttctatttttggaatgggatatttacggaatcccatgaataggatcaaaccttattccatgatatttccataagattcctctttcttattcttaagcaagcccagggcttagttgatcatgatttatgttttatctttcttttcctttttgtttgtttcgagaatcgtccgattctttttcttgtgtctacatagatcctgttcatggagtcagagtcgaaaagaggattcctcacttctttctctcattcaaaaccgtgcatgagactttcatctcgcacggctcctaagtgataaaagaaagaagaactcatcttctttcttttttgattaccttcctcgcgtatgtataagaccgaatccattcgatttctcgattactaatccttaacttttcgaggaatccttcatcagtggttgtgaatgactgactttttcaatcctttcgaccttggttccgtaggagcaagtcagaaagattgagaaatagaaccatctgatttgattcgttctcaatagccatgagatgatcatcttagggtgatccttttgtcaacggatgctcctattacactcgtagtctctgaaggatgagaaccaactatgtagcatctacatcgataattcaagcattgtatacgtcattagtccgatcctttgtaggaactacccgtaataacgaacttgtaaaatggatctgtttatcataaagagattcgttgttcctgaccctgcttcaccttaattgttatttgaacaaaaagatcaccttttggtaaaagttatgtcttggtccgagtggggatagcatttctcttctgcatgtctatggagttttgaaaaatccaaacatctcagagatagatatagaggtaggaatttgtcgaacgaaccgcacttaagaccattccaacgctccctttcgccatgcataaactgaacccacaattgggataagcacgaaaattaaagcttcgataaatacagatacacccaatacatcgaaactcattgcccatggataaagaaagaccgtttcaacatcaaaaacaacaaaaactagagcaaacatgtaatagcggattcggaattgtaaccaagcgtcccccataggttctatgcccgattcataactagagagcttctctggtcctttactaaccggggctaaaactcctgaaattacaaatgccaagataggaataacgcttgatattattagaaatgcccataaaatatcatattcgtgaagcagaaacataaatgtactcctattaatgttggcattgtcaattcatccagaacttgttttcctaggtgaaacaagaatttattttaatcaaagtgtatagttcagagtttgtttgctgcgtggcatgtcttgtttagagattcatccaaggaatctcgattcctttttgatttcgattctatttagatatggtgtagaggcgttcttacacctctattctctattttctttttcttgtcctagatttgacacatactgatctgattagatccattggaatgaattattgtttttattttcactatgtatttacatgaatatgtggtaatgctcatttctatgaaaaaccaatggtctgtccagtctataaacaagtactaatgaggaaatgaaaactatactaaaaatagaatgtctctatctatgataataattggataagatagcgtgtaccttgtcaactgataacgagagaacaaaatcgggataaataccaatacttattacgggtagaaagatacagattgaaagaaatagttctcgtagtccagaatcccaaaaattagagtttggaatattaaataacttgtatccataaaacatctgacgtaacatagataataaataaataggagttaatatcattccaattgccattacaaaagtaattagcatttttgacattaaaagatattttgtactagtaattagtccaaaaaatactacaaattccgcaacaaaaccactcattcctggcaatgcaagagaagccattgagaagctactgaacatggtaaatgtttttggcattgggatagatatccctcccatttcttcgagataaacaagacgtgttctatcacaactcgttcccgccaagaaaaaaagtgcagcaccaataaatccatgagagagcatttgtaaaatagctccattgagtcccatgtcggttatagaaccaattcctataattgtgaaacccatgtgagatacagaggaataggctattctcttttttaaattacgttgaccaagagaagttgaagctgcatagattatttgcattgttcctattatcaccaaccaaggagaaaatatagaatgagcgtggggtagtaattccatattgatccgaatcaatccatatgcgcccatttttaataggattccagctaaaagcatacatgtactgtaatgtgcttctccatgggtatcaggtaaccatgtatgtaggggtataatcggcaatttgacagcataagcaataaggaagccaaaatagaatattatttccaatgccgcaggatatgattgattaattaatctttcaaaatctaatgttggttcattggaaccatataaacccatacctagaactcctattaagaaaaaaatggaaccccctgcagtgtacaaaataaactttgtagccgagtagagacgtttctttcccccccacatggataaaagtaagtaaacaggaattaattctaactcccacatgatgaaaaaaagtaaaaagtctcgagaggaaaataaccctatttgaccgctgtacattgctagcatcaggaaatagaacaaccgcgaatttcgagtaattggccaagctgctaaagttgctaaagtagtgataaatcctgtcagtaaaatgggtcctatggaaagtccatcgattcctagtctccagtggaaatcaaaaacatctatccatttagaatcttccttcaattgcattaatggatcatccaattggaaatgataacagaatgcataagtcgttagaaggagttctaataagcatatacacatagtataccacctaaacatcttattccctctatgagggaaaaagaaaattgaggaacccgcgaatatcggtaaaacaacaagtattgttaaccaaggaaaataactcgtgataaagactagatacataaagcccgtcctcaaaataatatattttgtcgagcacgggcttttgtcggtaaagaggaatcacaaacgattcaagtggatttttgtaacgtactaattattcaataaattcgattgattaatacgagttgattttctgttacgatggatcgacgaaacaatagcaagtccaatagctgcttcagcagctgcaatggctataccaaagattgagaaaatgtctccctttaattggcgactatcaaatatatcagaaaatgttacaagattgatattaaccgaatttagtataagctcaagacacataagcgctctaaccatgtttcgacttgtgatcaatccatagataccgatagaaaataaataaacactcaaaaaaaggacatgctcaaacatcattgactaactccttatcaatctcgattcatttcaatatgaacaacaattcaaccgattcaattgattagaatagaacaattacacaacaaaagaagatattgacggtagatagatttaacttagaatttagttagaattctaagaattgggaatcattataagttaagtatttttattgcttttattagaaataaaaaaataaataaaaatattgatacataagataaatacaagaatagataagacgatattcgtccacctcccatatatttaatcccttcccctataaaaaaacttgcaacaccaacaccatttgtaattccatcaattatacgtctatcaaaaaactgagttagtttggttaatcctcttatccccacggtaaaaactctagtataaaaaatatctatgtaaccacgattatatgaccaattgtatatcacattttttattcggtccacaagaattcttttaggacccctctttacaaatgaattgattaaatccaaattctggaaaaatgaataagcagatccatataaaatatatgctatgaatagtccgaaaattgctatacttaccgaaaaaatggcatttgtaaaaaattcattcaaatttttagaacttgaatgtaaaagatttgttgatggggttaaccattttgataatatatcaaattgatcaaaagaaattcctattgatccaaccaacaaagtaaatagtactaatacaagaagaggaaatagcatagtattgtccgattcgtgaggatacatggaagtgtatttatttccaaagtaagtactaaagtatcgtatcctatttcttacattattatcaattttcgatctttcttttgcaaaaaaagaaacctttttattcattgttgataaaagtaaatttggattgactcctcttggagttccttttccccatagagatatggaatagaacgaaccatttttcgtgttactgtaatttttaaaatgaacgcggaaatacccatcaaaggtaagtaaatatacccgaaacatataaaatgcggttaatcctgctgtgaaataagctattactgcgaaaattggtgaatacaaccaactatcattaagaatgtcatctttggaccaaaaacaagcaagaggtggaataccacaaagagaaagtgtacccaataaaaaagtagttcttgtaattggaacatatcttgttaaaccacccataagaaccatattctgacttttatctggtgaatatccaacaataggttccattgaatgaataatagatccggatcccaaaaacaataaagcttttgaataggcatgagtgatcaaatggaataaagcagctcgataagaacctatacctagagctaacataatataacccaattgagacattgtagaataggctaagctttttttaatgtctctttgagcaagggccaaagtagcccctaataatagcgttattacacctattaaagaaatgaaattcattatataaggtatgactatgaaaagaggaagaagccgagctacaagaaaaattcctgctgctaccatagtagcagcgtgtataagagccgaaataggagtgggtccttccatagcatcaggtaaccatacgtgaagggggaattgtgcggatttagcaactgcaccgacgaataataaagaagcacacaaggtagcaaataaagaattgacctcattattctggattaagttattagttatttcaagcaaataccgaaattctaaactacctgttatccaataaaaacctaagattcctaacaataaaccaaaatcccctacacgattagttacaaaagctttttgacaagcacttgctgcaattggtcgtgtgaaccaaaaacctattaataaataagaacacattcccacaagttcccaaaaaatataaatttgtatcaaatttgaactagtaactaatcccagcatagaagtattaaaaaaactcatataagcaaaaaatctcaaatatccttgatcgtgatacatatacttgtcactataaataagaaccatgattccaacagtagtaattagtattgacataatagaagtaagtggatcgatcaagtatccaaactctaaggaaaaatcattattgatggtccaagaccatagatattgatagataaaacttccatttatttgttgaatagacagattggctgaaaacaccatagctatacttaagagtaaaacaataggaaaagcccacatgcgacgaatattttttgttgctgtcggaataagtagaagtccaaatcctattgacatagtaactggaagtggaagaaaaggtattatccacgcatattgatatgtatgttccataagaaaagaacctttttttcttataattacaaattgttatcgattcaccttagaaaagatacaaaaaaagaacgatttgaagaattattgtcgagccatagtaattgcacctatcaaggaaactaaaagaattattgaaatgagttcgaatggaagataaaaatctgttgataaatgaatcccaatttgttgaacgttatttattaggtcctgttccataatctggtttgaccttgcagtccaaataattccataccatgacgtatctgggatagtagtaattagtgaaaaaagaatagttgtacaaaccatcaaagtgaccccatctccaatggtccaaaaattggaattattggaatattctgaaccattcatgaacattacagcaaatatgatcaagatatttatggctcccacataaataaggagctgtgcggcagctacaaaataggagttcgatgaaatatagaataaggatatacaaacaagaaccaatcccaatgaaaaggcagaataaattggattggtaaataatactacccccagactccctaatacaagaattgaccccagaaatacaacaagaatatcatgtattggtccaggtaaatccattatgtataaaatacaaaataaataactttttcatgaccttaactttaaatggtccaggaaaggaaaaggggttaccctatttttgttttcttgtatataatattgtatatgatacatttataattgaatatggattaatgtagatatagataaaattatcgggccaaccttaaaaaagtagttgaaatttgctatttcgaaatcatcactaaaaatatatttttagtttaaatcaaagagtgattctcaacaatcattagtttttatttgtagttactgactacccaaaataaatggatcctttatatcaaaacaaaatctcaacgatcaacttctcccataatgatatctatactacccaatatcgtcatgatatcagccaatttcattcttttaactagctgaggaagaatttgcaaattgataaaaccaggtggacgaattttccatctccaggggaaaacactattatctcctatcaaataaattcccaattctccttttggggcttccactctcacataaagttcttgtttcgacaattctaaattgggtgaaggttttttactaataaatctatattcaaaatcattccattcggaattctttgctctatcaaagcgtcgtacttctaaattttcataaggtcctccaggaattccttctagagcttgttgaataatttttatggattccttcatttcaccgattcgtactaaataacgagctaatgaatctccttctttttgccattggacttcccaatcgaattcattgtaacactcataatgatcaactttacgaagatcccattggattccagaagctcgtaacatcggtcctgataaaccccaatttacagcttcttctccaccaatgatgcccactccttcaactcgttccaaaaaaatgggattccacgtaataagtttttgatattcaacaactcctgttaaagaataatcgcagaaatccaaacatttatctatccatccataaggtagatcagcagctactcctccaatacggaaataattatgcatcattcgcatacctgtggcggcttcgaatagatcatatatcaattccctttctctgaaaatatagaaaaagggagtctgtgcaccgatatccgccataaaaggtccaagccataacaaatgagaagctatacggctcaattccagcataattactctgatatagctagctctttgaggtacttgaacattttccaattgttctggcgcatttacggttattgcctctgtgaacatagtagctaaataatcccatcgtgttacataaggtagatattgtataattgttcgattttccgctatcttttccattcctctgtgtaaatagcccaatatgggctcacagtcaataacatcttcaccatcgagagtaacgattagtcggagaacaccatgcattgatgggtggtgaggccccatattgactatcatgagatcttttcttgtaaccggtactgtcatatcttttcttccttaattcattattccataaaaagagactcatcaaaacgaaaaattgaatactaccgattaaattagtaatcggtaattgttcttgaatcaaagggtttatctttgtctatttttatttgagtcgaattcataactgtttgaattgtataatctccaattattgagattggtaatcgacccaaagcaatttgattataattcaattcgtgacgatcataagtagaaagttcatattcttcagtcattgataaacaatttgttggacaatactcgacacagttaccacaaaatatacaaaccccgaaatctatactataattaagtaattgtttctttttaatatctctttcaaatctccaatcaacaacgggtagatctatcgggcatacacgaacacatacttcacaagcaatacatttatcaaattcaaagtggattctaccccggaaacgctctgatgtgatcgatttttcataaggatattgaatagttacaggtaaacgatttgtgtgggataaggtaattatgaaactttgaccaatgtaccttgcagctcgtattgtttgttgaccataattgatggacccaattaccatagggaacatattgtagatatacatgaaaaatttgacgtttctttctcttgtttgatagagattatgaatctaaaatagtgttatcgtattctcttattcaatgagcatcttgtatttcatagaaatttggggtaatatagtccttacgtaagggccagcctatccaactttcaggcattaagatacgtttaagacgtggatgattattataagagattcccaacatatcataagattcgcgttcttgaaaatcagcacttctccaaatccagaaaacagacgggattctaggattatttcttggagcaaatacttttatacatacctcttctggtttatctataccatactgtattcttgtaagatgatacacactagctaaaaatccgcctggtgctacatcataggcacactgagagcgtaaataattgtaaccatatacatatgaaatgacagcaatggagtcccaatcctcggtttttatttgtaaagtctctattcctcggtaatcgaagcccaaagatctatgaaccagctcatgcttgactagccaatcagataagcgattctgatgcattgtcttgatctctcccacatttgtataagtatttcacatttacaatgaaatttttaaagattgacctgctcttttgaaaaatgtttcagaagatatctctgaagtagatggtgattgatagagcaatccttgatcgtaatttccagtatgagtactgtgtcgaacagaaaatttgtgattggtagtaaaatatcgatttttctgttgagatacagctctatcttcaaatatttctcgagatatcttcttacgaagttttgttatagcatctataactgcctctggtttgggtgggcagcccggcaaatagacatccacaggaattagcttatcgactccccgaacagtactataagaatcagtactgaacatccctcctgtaatagtacaggctcccatagcaatgacgtattttggttcaggcatttgctcatataatctcactaaagagggagccattttcattgtgactgtgccggctgttaaaattaggtccgcttgcctaggacttgatcttggtaccaacccataacgatcaaagtcgaatcgcgagcctattaatgaagcaaattcaatgaaacagcaactggtaccatatagaagcggccataaactggagagtcttgaccaattcgaaagatcattcgatgtagttgaaataactgaattgggggttgtttggtcaagtaacggaaactcaatcaaattcataactgtctcaatgtaattttttccttccttttttttgtctgaatactcagatgcaaaatagaaatactttttcttgggtaaaggaacagatgactcgatccatttctgtatcgatcatgatatatgtaataactcgagcatccatttcaaatgcatatcccatttttgcgcagcagggttatgaaaacccacgagaagcaactggacgaattgtatgtgctaattgccatttagctaataagcccgtggatattgaagttccgcaagctgtgcttcctgatactgtatttgaagcagttgttcaaattccttatgatatgcaactgaaacaagttcttgctaatggtaaaaaagggggtttgaatgtgggtgctgttcttattttacccgagggattcgaattagccccccccgatcgtatttctcctgagttgaaagaaaagataggaaatctgtcttttcagagttatcgtcccaataaaaaaaatattcttgtgataggtcctgttccgggtcagaaatatagtgaaatcgtctttcccattctttcccccgaccctgctacaaagaaagacgttcacttcttaaaatatcccatatatgtgggtgggaacagaggaaggggtcagatttatcctgatggtagcaagagtaacaatacagtctataatgctacatcagcaggtatagtaagcaaaatagtacgtaaagaaaagggaggatatgaaataaccatagttgatgcatcggatggacgtcaagcggttgatattatacctccaggaccagaacttcttgtttcagagggtgaatccattaagcttgatcaaccattaacaagcaatcccaatgtaggagggtttggtcagggagatgcagaaatagtgcttcaagatccattacgcgtccaaggccttttgttcttcttcgcatctgttattttggcacaagtttttttggttcttaaaaagaaacagtttgaaaaagttcaattgtacgaaatgaatttttaggtccagagattccttaacatttggtaaaaagtgccgattttttgtccatcgatacaattatgtatgatcaaaaaattctgtaaatccttttgcttgctttgtttatactctttttgttttgcaggacgcctggaattcattacttgtattcctagaggcatacaaacaaatacaaaaaaagcaaggatggtaaaaatgaaaaattttaggaaggattactagtcttcctaatcttctattcgacaggaattttcacccgttttcttgtgtcgtcttgtatcaaaataataattatttttttcctgttcatcaaagattactattcctttccttcttttccgggtctatcggaactcctttgtttagattcataagaagtagtggacaaacaaaggaaaaaaggattatgggcgaagaaaatagataagttaaacttctaacttagagaaattattcaaaaaaaagactgttccggttgaaaggcggatttgattaaggttctattagtttagtctagaaatgatttgcaggatgtctcatccctagaaatcaatataatttatattggattatagataaaatcgattgattcgttctttcttcttgcttccagagagcaaaggatcctttttgtcatttctacaaatataatatctggataactaaccaatttgtaggttatggaatagatcaaagctcattataagaccgcgggccctttccgctctaatcagacaaaggagggtaaggacccgctaagttcttactttttcatgtctacaacccagcatgagtaaagtatatgattggtttgaggaacgtcttgaaattcaggcgattgcagatgatataactagtaaatatgttcctcctcatgtcaacatattttattgtttaggggggatcacacttacttgttttctagtacaagtagctacgggttttgctatgactttttactatcgtccaaccgttacagaggctttttcctctgttcaatacatcatgactgaggccaactttggttggttaatccgatcagttcatcgatggtcagcaagtatgatggttctcatgatgatcctgcatgtatttcgtgtgtatctcacaggtggatttaaaaaacctcgcgaattaacttgggttacgggtgtggttttggctgtattgactgcatcttttggtgtaactggttattccttacctcgggaccaaattggttattgggcagtaaaaattgtgacaggcgtacctgaagctattcctgtaataggatcgcctttagtagagttattacgtggaagtgctagtgtgggccaatccactttaactcgtttttatagtttacacacttttgtattgcctctccttactgccgtatttatgttaatgcactttccaatgatacgtaagcaaggtatttccggtcctttatagagaagacatatctgtaattgatcatatatcggggaggacaatagtatttcattgctacaaatatggattattgaaaaaataagacatgtttttggatacttctcttcaatattgtattccttatttgatacgaatagttgaagtgaattttctgaagagaggatggattgtgtgacttgttataattgatcctattgataatacatagaatggacctgttatctctatcaagatgattctacctcgtcggatatttattctagtatctggggcacggaatatatagaatagatcaagaaaagaaatatttgaactatgattcatacctactattcagacctcgcaaccggactcaaaaaaattcaaataggtatttcctaaatcaaacgaattttattccttcagatttattttgaccgaaggataactctttctctagattttgttgagtcattacatccattcattcaataagtgatcatcaaaggttcttactcgagaacctttgagtttagcttgaggctaaatcatcgtggttctagtatgaatctgaggtttcaattgattcatagggtctcaacaagagaattcctatcaatagtaaaagtaaatccgcaaaaatagggaagagaagattcaagaggcctgtaacgatcaacataaagaaagacagatgagccaacttgatattttttggcattatcatcacaaagaagagattccggatttttttcgtatcttcgaggcaaatcaagtggttaatgaagtttttaactttctattagatatccgttgaaatcagtatttgtgtgtttccgcttgagccgtacgagatgaaattctcatatacggttctcagagggggagttccattgggttacctatctcaatatgggaataacaaagaaacctgacttgaatgatcctgtattaagagcaaaattggctaaagggatgggacataattattatggggaacccgcgtggcccaatgatcttttatatatttttccagtagtaattctaggtactattgcatgtaatgtaggtttagcggttctagaaccgtcaatgattggtgaaccagcagatccatttgcaactcctttggaaatattacccgaatggtacttctttcccgtatttcaaatactccgcacagtacccaataagttattgggcgttcttttaatggtttcagtaccaatgggattattcacagtaccttttttggagaatgtcaataaattccaaaatccatttcgtcgaccagtagctacaacagtttttttgatcggtaccgcagtagctctttggttaggtattggagcaacattacctattgataaatccctaactttaggtctttttcaaattgattcaaccgcgaagtacggaatagttacttcaaagtgaatcttccctagatacattaatttgattatgatctattccgcgaaaatacggatcgtgccaaagatgaaaaatttttttctttagaattagaaaaagaagatgaaaaaattacaatggatgtgtgacttgaactattgatttagccatgcagatatatgaatttgccacgttggaattcacaaccaaacgtgtctccgcatccaaccaaaacgtaagccccctacgtagcagaggataggccggttcgcttgaggagaatattttctatgatcataccgaatcatgttattcacgaataggctccgtaagatccgtagaatagaataagtgatgtggcatgatccaatgttctattccacttatttattatagtgtggaaatgcattcattccctttgcatcgattccgatctatgatactatcggagtgaaagaagggatctaaggaagaacagaggctagactttattagtaacaagtaaatactttttacgtaagaaactcgagatatggtgggtataaacaccaatcaaaagacatgagacaatccaaaaagcacttgatcatgatcaaatttttaagcctacttggatattgagcatttgcctataagaacagaattctttgcaatgaatagtttgcaattctggaaaattgaatgtgataaatctttcttacatagagttattaactatgttattgtatgaaataacgattttttattttatatgggtttatttctgttattcctttgattcttgctcgagccggatgatgaaaaattatcggatccataagaattcacctatcccaatgattgaagtttttctatttggaatcgtcttaggtctaattcctattactttggcaggattatttgtgactgcatatttacaatacagacgtggtgatcagttggacctttgattgagtaacatttctttttttgattgacctcctacgggaggaggtcaaattccagttgcaattcaactttgttaagttattttattgtgattcgacatacataagatagacagaatatgcttactataactagttattttggttttctactggctgctttaactataaccccagctatatttattggtttgaacaagatacgacttatttgaaaatgaattgaataaataattcagaaaaatatttctcgggaattccagatattctatggttctttcccgcaccaattgccaatttttttcttggtcattgagattcacggataattcagattaatatttagggatagatcttacccctttttatcccctcaaacaaaccgaaatggatatagtaagtctcgcttgggctgctttaatggtagtctttacattttccctttcactcgtagtatggggaaggagtggactctaattatattaataattgattgagtaatcgaattgtataaattgtttatttttcgaagcttttttttaaattctttcaaaattatactggaaagacaataatgaataataaccattcgatcaaacaacgaatgattactttagttgtatttcaaaacttacgcatgataggaaattttttacaaccgaattcctcctaaatattctgtttggataaacctgttcttaccagaattttggatattacaatgagaaaaaaccaatccctagttttttctttatttgtttctctctttctttactttcactgttctaagaacaaatcgttctgctattagattacgacgatacttactttatactggaagtgactagtggttgaggttctacacataaaaaaattggatctttcttccgctgagtgatccaccacttagactcctagagatttttttatgcttttttgacatgtcatgtttaagcatgaaaaatggtccgagtcccctttactaattcctttcaaaatctgaagaagttaccatagaacttcgtaaatgatctgttaatggctcaatcaatgacttcttgggatgggaaatcaaaaaaattccttgttttgctatagtatattcctatcttcctctattgattcttttgatggataccggaacctatatataaattataagaaacctaggaattagaagaattggccttcgattattttgggttgatcgaaatcgagtggatgtagcgaaaaaaaatcgaattagactgctatttcatctgtatacaatacaactttatatgatcattgtatacaagataatataaaatactctaaaatgtggtagaaaggactatatatagtcctttctaccacattttatgattccaaccagatcatttcatttaagacttggaattttcttttaatcccttcttcaatcattgaaaaaggattgataagaactatcagattatcctactgcaataattcttgctaagaagaacgcccatgtagtggcaattccacccagaaggtaatgggttactcctacagcacgtccttgtacaatgctcaaggctctaggctgagtagcaggagcaacttttaatttgttatgagcccaaacgatggattcaatgagttcttgccaataaccacggccactgaatagaaacattaaactgaaagcccagacaaaatgagcacctaagaaaaaaagaccatatgcagataatgaagaaccataagactgaataacctgagatgcctgtgcccataagaaatctcggagccacccattaatagtaatggaactttgtgcaaagtttcctcctgtgatatgagttaccaccccttgatcacttatagtaccccaaacatccgactgcattttccaactgaaatggaaaattactacagaaattgcattgtacatccagaatagacctaagaagacatgatcccaggcggatacttgacatgtccccccccgccctggtccatcacaagggaaacgaaaaccaagatttgctttatcaggaatcaaacgggaactgcgagcaaatagaacacctttcagtagtatcaatacagtcacatggatcgtaaatgcatgaatgtgatggactaaaaaatctgcggttcctaatggaataggtaacaaagctactttgccgcctactgctactaactcaccacctccccaggttaagctggtactcgttgttgcaccaggagctgttacgccaggtgctaaagcatgggtattttgtatccattgagcaaagatgggttgtaattgtatagcggtatctgaaaacatatcttggggacgtcctaaagcactcatggtatcattatgaatatacaagccaaaactgtgaaaacctagaaatatgcatacccagttaagatgtgatatgattgcatcgcggtgcctaaggacgcgatctaatagatcgttgtatcgagtagttggatcgtagtctcttaccataaaaatggctgcatgcgcagcagcaccaactatgagaaatccaccaatccacatgtgatgtgtgaacaacgaaagttgtgtaccatagtcagtagctaggtatggataggggggcatggaatacatatgatgagctacaacaatggttaaagagcctaacatagccaggttaagagataattgagcatgccatgacgttgttaggatttcatataggcccttatggccctggcctgtaaatggacctttatgagcctctaaaatgtccttaaggccatgaccaatgccccagttggtcctatacatatgaccagcgatcaggaaaagaatagcaatagctaaatgatggtgtgcaatatcgctcaaccacagaccccctgttattggatctaatcctccacgaaaacttagaaattccgcgtattttgaccaattcaaggtgaaaaagggggttgctccctcggcaaaactgggataaagttgagccaaaaggtcccgattcaagataaattcatgaggaagtggtatctctttaggatcaactccagcgtcgagaaattggttaatcggtaaagatacatggatttggtgtcccgcccaagaaagagacccaagtcctagtaaccccgctaagtggtgattcaacatggattccacatcttggaaccaagccaattttggggcggctttgtgataatggaaccaaccggcaaaaagcattaacgctgcaaagaccaatgcaccaattgcggtacaatagagttgtaattcactagttattccagatgctcgccaaatctgaaaaaaaccggaggttatttgtattcctcggaaacccccgcccacatcaccattcaatatttcttgacctactattggccaaactacctgggcactgggtccaatgtgagtaggatcacttagccatgcttcataattggaaaaacgggcaccatggaagtacatgccactcagccaaagaaagataatggagagttgaccgaaatgagcactaaatacttttcgggagatctcctccaaatcactggtatgactatcaaaatcgtgagcatcagcatgtagattccagatccaagtggtagtatcagggcccttagctattgttcttgagaaatggccgggtctggcccattcctcgaaagacgtttttataggatccctatccacaacaattttcacttctggttccggcgaacgaataatcattaagtcctcctctttccggacaacacatacaaagagacccgccaacagtcaagtttttagtgaacctctgaaagatagatattttatttagttattcactagagcaattatgatattgaagtcgatccgaggcaagtgttcggatctattatgacataatgattaggtgcccaacggacctttaccttggaaaatcctttccggcgtgacaaaaagcaacctagtgtatttatatctgaatgaaggtataagtacctatatggatctacttacatggaacataatatcttttacaaatcacaagatcattcattagaaaagataaaatcattctgatatcttaacaaaatataatgcagaaggaagagatataataaaattcttgattagatcttctcataggaacgatctttttatttgattgatggatccaacaaccaaacctaaattcattaaaaaagagagtggttttaaccaaattttcctgatgtagaggcaatcaagaaagctgcataagtaaatatataacccacggaaaagtgggctaatccaaccaatcttgcttgtacaatggaaagagccaccggcttatctctccatcgaatcaaattagccaaaggtgtgcgttcatgagcccatgctaaagtttcaatcaattcctgccaatatccgcgccatgaaattaagaacataaatccagtagcccaaacaagatgtccaaataagaacatccacgcccataccgataaactattcataccaaaaggattatatccattgataagttgtgaagagtttaaccatagataatctcttaaccatcccatcaaataagtggaggattcattaaattgtgaaacattaccctgccataatgtgatgtgtttccaatgccaataaaaagtaacccatccaatggtatttaacatccagaaaactgccaaataaaatgcgtcccaagcagaaatatcacaagtaccgccgcgtcctgggccgtcacaaggaaaactataaccgaaatcctttttatccggcattaatttagaaccgcgtgcatctaaagcaccttttactaaaatcaatgtagttgtatgcaaacctagagcaatagcatgatgaaccaagaaatccccagggcctattgttaagaaaagtgaattactattctcattaatagcattcaaccatcccggtaaccatatgcttcgacctgcattgaaggctgggccattcgttgaagataagagtacatcgaacccatatgaagtcttaccatgagcagattgtatccattgggcaaatatgggttcgatcaagatttgtttttctggagtaccaaaagcgagcatgacgtcattatgaacataaaggcccaaagtatggaaccccagaaacagactggcccaacttaaatgagatatgatagcttctttatggtctaacattcttgccaatacgttatcctcattctgctccggattgtaatctctaatgaaaaatatagctccatgagcaaaggctcctgtcatgatgaaccctgcaatgtattggtgatgagtatataaggcagcttgagtagtaaagtcttgtgctatgaatgcataagcaggtaaggagtacatatgttgagctactaaggaagtaataacccctaaagaggctagagcaagacctaattgaaaatgaatcgaattattgattgtgtcataaagacccttatgtccacgtcctaatcgaccccccggaggagtatgtgcttctaaaagatctttgatactgtgcccaataccgaagttagttctatacatatgaccagcaatgagaaaaataaatgcaatagctaaatgatgatgagcaatatcggtcagccataaactttgcgtttgtggatggaatcccccaagaagggttagaatggcagttcccgccccttgggcggtcccaaataaatgattactcgaatcagggttttgggcataaagattccactgacccgtaagaaggggtcccaacccttggggatacggtaatacgtctaagaaattattccatctgacgtactctcccctggatccgggaatagcgacatgaactaaatgtcctgtccaagccaaggaacttactccgaaaagtcctgacaaatgatgattgagacgagattcagcatttttgaaccacgaaacgcttggtttccatttgggttgtaggtgtaaccaacccgctattaaagatatagcagaaagaaataatagaaaaagagctcctgtataaagatcttcattggtgcgtaatccgattgtataccaccactgataaacaccggaataagcgatattcactgggccggtagcacctcctcgagtaaaggcttctacagctggttgaccaaaatgaggatcccaaattgcatgagcaattggtcttacatgtaaagggtcctgtatccatgactcaaaatttccttgccaagccacatgaaacagatttcctgacgtccacagaaaaattattgctaactgtccaaagtgagaagcaaaaatgttctgataaagacgttcttcagtaatatcatcatgactctcgaagtcatgtgcggtagcaataccaaaccaaatacgacgagtagtggggtcctgagctaagtcttggctaaacctcggaaatcttaattccataatgcctttcaaatcctcctagccatcaataagatagagccatgctgcgagttgtttcaggccctaaataaacacggacactcaaaaaatctgttgggcaggcagattcacatctcttacaacctacacagtcctcggttcttggcgcggaagctatttgcttggctttacatccgtcccaaggtatcatttccaatacatctgtggggcaagctcgtacacattgagtacaccctatacatgtatcataaatttttactgaatgtgacattggatctataaagttttgaattttgatctggtattatatattgtaatattgtagacaccagacgaaacagtggtttattaaaaacaatcaatatatttcttaaatcaatctgtttatgagaaaaggtcaagacactttgattttgtgtcatcattatttcaatataaataatagtatttcaattctatgatgattatcaatgacagatctcaacttaccctctatttttgtgcctttagtgggcctagtatttccggcaattgcaatggcttctttatctcttcatgtccaaaaaaataagattgtctagatccaatgggaccaaatcctatcaatttatttcaacactgtatcataatacagatattttttagtgcggtacgatatgtggatctttccacacacaaatgaaagaactgttatgtatgcggatacatgctatctgcataaatgtagctggttaaaaacggatcagtaaatatttttaaaagtcaatgtataaaggtaaagtcaaatttgggttattctctcaattccaatcgaatgcaactggatctagtatagtatgcgagatctaaaaacatatctctccgtggcacctgtgttaactactctatggtttgggtctttagcgggtctattgatagaaattaatcgtttattcccagatgccctgtcattcccctttttttaattattgatatgcgaagaaatgaagaaatataattacacatgacgtaactaaaacctcgcctctccctttcaattctttagaatagtaaggaaaaagtgtattgaacctcataaaaaatccggtagatccaagatcgaatttagggcgggctctacgaaatcatagcatagaaagaattaaatgaaatattgggatttaggatagaaataattgatagttagaaagaaattgtattacttaatttctatatatatactatattaatacataattaattagataaaattccaactatttattttttcttcttcttcggttcgaaaatataagacttaagttaagtcgatacaaaatctaaaggaggttcttatccatttgtagatggagcttcaacaacagctaggtctagggggaagttgtgagcattacgttcatgcattacttccataccaaggttagcacgattgatgatatcagcccaagtgttaataacacgaccctgactgtcaactacggattggttgaaattgaaaccatttaggttgaaagccatagtactaatacctaaagcagtgaaccagatacctactacaggccaagcagccaagaagaaatgtaaagaacgagaattgttgaaactagcatattggaagattaatcggccaaaataaccatgagcagctacaatattataagtttcttcctcttgaccgaatctgtaaccttcattagcagactcgttttcagtggtttccctgatcaaactagaggttaccaaggaaccatgcatagcactgaatagggagccgccgaatacaccagctacacctaacatgtgaaatggatgcataaggatgttgtgctctgcctggaatacaatcataaagttgaaagtaccggagattcctaaaggcataccatcagagaaacttccttgaccaataggatagatcaagaaaacagcagtagcagctgcaacaggagctgaatatgcaacagcaatccaaggacgcatacccagacggaaactaagttcccactcacgacccatgtaacaagctacaccaagtaagaagtgtagaacaattagctcataaggaccaccattgtataaccattcatcaacagatgctgcttcccagattgggtaaaaatgcaaacctatagctgcagaagtcggaataatggcaccagagataatattgtttccataaagtaaagaaccagaaacaggttcacgaataccatcaatatctactggaggagcagcaatgaaggcgataataaatacagaagttgcggtcaataaggtagggatcatcaaaacaccgaaccatccaatgtaaagacgattttcagtgctggttacccagttgcagaagcgaccccataggcttgtactttcgcgtctctctaaaattgcagtcatggtaagatcttggtttattcaattttcaaggactcccaagcacacgtattaactatagatgtgatagataatagaaggcttgttatttaacagtataacatggcttatacaccaatgtcaaccaatcttaacaaatgattaaatccacccaaagaatttgtaaatgaaatgagttcaaatattttattttccgtaatgggtttaccttggtatcgtgttcatactgtcgtattgaatgatcccggtcgattgctttctgttcatataatgcatacagctctagtttctggttgggctggttcaatggctttatatgaattagcggtttttgatccctctgatcccgttcttgatccgatgtggagacaaggtatgttcgttatacccttcatgactcgtttaggaataaccaattcgtggggtggttggagtatttcaggaggaactataacaaatccgggtatttggagttatgaaggtgtggcaggggcacacatagtgttttccggtttgtgcttcttggcagctatctggcattgggtatattgggacctagaaatattctgtgatgaacgtacgggaaaaccttctttggatttgcccaagatctttggaattcatttatttctctcaggggtagcttgctttggctttggcgcatttcatgtaacaggtttgtatggtcctggaatatgggtgtccgatccttatggactaactggaaaagtacaatctgtaaatccagcgtggggcgcggaaggttttgatccttttgttccgggaggaatagcctctcatcatattgcagcgggtacattgggcatattagcgggcctattccatcttagtgttcgtccgcctcaacgtctatacaaaggattacgtatgggcaatattgaaactgtactttccagtagtatcgctgctgttttttttgcagcttttgttgttgctggaactatgtggtatggttcagcaactaccccaatcgaattatttggtcctactcgttatcagtgggatcagggatactttcagcaagaaatatatcgaagagttagtgtcgggctagccgaaaatcttagtttatcggaagcttggtctaaaattcccgaaaaattagctttttatgattatattggtaataatccggcaaaggggggattattcagagcaggctcaatggacaatggggatggaattgctgttggatggttaggacaccccgtctttagagataaagacgggcgcgagctttttgtacgtcgtatgcctactttttttgaaacatttccggtagttttggtagatgaagacggaattgtgagagctgatgttccttttagaagggcagaatcaaagtatagtgttgaacaagtaggtgttactgttgagttctatggtggcgaacttaatggagtaagttattctgatcctgctactgtgaaaaaatatgctagacgtgcccaattaggtgaaatttttgaattagatcgggctactttgaaatccgatggtgtttttcgtagcagtccaaggggttggttcacttttgggcatgctacgtttgctttgctcttctttttcggacacatttggcatggcgctagaaccttgttcagagatgtttttgctggtattgatccagatttggatgctcaagtggaatttggagcattccaaaaacttggagatccaactacaaggagacaagtagtctgatacgacattgttgtggtatctttcgcctctatttttttgacattgggtatcagagaaatcttgacttgaatcaccttctttgactttttttctttatatgatatggtaaatgatcccaaatgaataggtgtggaagctataattgtaaaccacgatcgaatccatgaaaaccttatattccctgaggaggttctaccacgtggaaacgctctttaatggaactttagctttagctggtcgtgaccaagaaaccaccggtttcgcttggtgggccggtaatgccagacttatcaatttgtccggtaaactacttggagctcacgtagcccatgccggattaatcgtattctgggccggagcaatgaacctttttgaagtggctcatttcgtaccagagaaacccatgtatgaacaagggttgattttacttccgcacctagcaaccctaggttggggggtaggtccggggggggaagttatagacacctttccatactttgtatctggagtacttcacttaatttcctctgcagtcttaggctttggcggtatttatcatgcgcttctcggacctgagactcttgaagaatcttttccattcttcggttatgtatggaaagatagaaataaaatgactacaattttgggtattcacttaattttgttaggtataggtgcttttcttctagtactcaaggctctttattttgggggcgtatacgatacctgggccccaggggggggagatgtaagaaaaattaccaacttgacccttagccccagtgttatatttggttatttactaaaatctccttttgggggagaaggctggattgttagtgtggacgatttagaagatataattgggggacatgtatggttaggttccatttgtatacttggtggaatttggcatatcttaaccaaacccttcgcatgggctcgacgtgcatttgtatggtctggggaggcttacttgtcttatagtttaggtgctttatctgtctttggtttcatcgcttgttgtttcgtctggttcaataataccgcctatcctagtgagttttacggacccaccgggccagaagcttctcaagctcaagcatttacttttctagttagagaccaacgtcttggagctaatgtgggatccgcccaaggacctactggtttaggtaaatatctaatgcgttccccgactggagaggttatttttggaggagaaactatgcgtttttgggatcttcgtgctccctggttagaacctctaaggggtcccaatggtttggacttgagtagactgaaaaaagacatacaaccttggcaagaacgacgttcagcagaatatatgactcatgctcctttaggttctttaaattccgtgggtggcgtagctactgagatcaatgcggtcaattatgtctctcctagaagttggttagcaacctctcattttgttctaggattcttcttttttgtggggcatttgtggcatgcgggaagggcccgcgcagctgcagcagggtttgaaaaaggaatcgatcgtgatttggaacctgttctttccatgacccctcttaactgagattttcttatttatatctattctattgctcttttatgttctggctcggctaggtggaatagccgagccattcctttttatgaaggataaggggccaggccaaacaaataaagaaaaaattgattcaataaacaaatgactatagcccttggtagatttaccaaagaagaaaatgatctatttgatattatggatgactggttacggagggatcgtttcgtttttgtaggctggtccggcctattgctctttccttgtgcttatttcgctttaggaggttggtttacaggtacaacttttgtaacttcatggtatacccatggattggccagttcctatttagaaggttgcaatttcttaaccgctgcagtttccactcctgcaaatagtttagcacattctttgttgctactatggggccctgaggcacaaggagattttactcgttggtgtcaattaggcggtctatggacttttgtcgctctccatggtgctttcggactaataggtttcatgttacgtcaattcgaacttgctcgatctgttcaattgcgaccttataatgcaatcgcattctccgctccaatcgctgtttttgtttccgtattcctgatttatccactgggtcaatctggttggttctttgcgcccagttttggcgtagcagctatatttcgattcatcctcttcttccaaggatttcataattggacgttgaacccatttcatatgatgggagttgccggagtattaggtgcagctctgctatgcgctattcatggtgctaccgtggaaaacactttattcgaagatggtgacggtgcaaatacattccgtgcttttaacccaactcaagccgaagagacttattcaatggtcactgctaaccgcttttggtcccaaatctttggggttgctttttccaataaacgttggttacatttctttatgttatttgtaccagtaaccggtttatggatgagtgctattggggtagtcggtctggctctgaacctacgtgcctatgacttcgtttcccaagaaatccgtgcagcggaagatcctgaatttgagactttctacaccaaaaatattcttttaaacgaaggtattcgtgcttggatggcggctcaggatcagcctcatgaaaaccttatattccctgaggaggttctaccacgtggaaacgctctttaactaaaaggatctactaaattcatcgagttgttccaaagaatcgaaacggccggttatcaatggaatcccttgtcggctttccgtgaaatactcatttggccgaggacttccaaacacgtcgtaagctaaacccgtactgacgaataaccaacccgcaatgaatagggaaggtatagtaatgctatgaataacccagtatcgaatactggtaataatatcagcaaaagaacgttctcccgtgcttccagacatgctgagctccacaaatttttgtacattcaaaagggggaatcgattccgtgaaagatgggatcagtaaatagaaaactactgatattgcatctttgtgagatcgtcaattttgtaccaaaggtgtatttcgagtataccgaatcagtatagctatccttcctctggcacagcaacgcagccttgatcggtaccgaaatgctacacaattcttcttttttgctccttatctatggataccttatgttatgctattcaatagatcaacctccaatttattataaggtttcctggcttcataaaagtaaggatcttgggaaaatgtaagtcaatgatcaatgggttctaataattcatgaaagatattattgtattgacacaattcaattatatgtgaaatctaacctggttaaattatttgaatcctttcatttcaagtaattggttggatatagtatctactatatcatatttaatgaaagaaagaaaacatagttgtaacaatcaatattcgcgatgcaatcattgttggattaggtccaagacaaagattctttcttgaccaaactacaagtatggaactccatgatatggaaagacagaatatagaacctaaaaggataattgaggcgactcgtcttcgaatcgactttgtacccgaaaaagagaacaataaagtcaaagtttttgtttctcaatagatcctttcgatggatggtggaacacctatgttatgtcactctatctttttttagtattatctataatgaccaatgaatcatgaattttccattggaactaaactaattctcttaattggtttttattaccctcatatctggatggaaacttaggtaagtgttttatcaacatatgtagaaaaaaacatatctaataaagccctttttatcgttggatgaactgcattgctgatattgaccccaagaaagaaacggtaggtacagctagtccgtgaacagccaaccagcgcactgtaaaaattggataagttcgatctatggtcatatggctacacaaaccgttgaggatagttctagatctgggccaagacgaactactgtaggggatttattgaaaccattgaattcggaatatggtaaagtagctccgggatgggggactacaccacttatgggggtcgcaatggctctatttgcgatattcctatctattattttggaaatttataattcttccgttttactggatggaatttcaatgagttaggtttataagaactatgaagtcctagtctttcaatcaaagaaaaattactttagacttggatttctagaccattctattctggtagttcgaccgtggaatttatttgttttggtatttccggaatatgcttactctcaaactctttgtttacacagtagtgatattctttgtttccctttttatctttggattcttatctaatgatccaggacgcaatcctggacgtgaggaataatttatattttgctttttaaaaaattaattcttaataatcttaggatgagattcgaattcaaaagtgatcagagaaagctagagagatgaacccaacccagaatatgaaccgtaaaagaaaacacctattaaaccgatcacaagaataccagttacagtacctatcagccaaagaggaatccttccagtagtatcggccatttcatcaagtggtcatgctagagacaaaaacagtcatggataattatgaggatggtatctatccgaatgggataagagaattactactattctctttctttctcatgcttaatatctttagtttaatcgatatctgtcttaattctgtcctttattcgagtagttttttcttcgccaaattgcccgaagcttatgccattttcaatccaatcgtagatgttatgcctgtcatacctgtactcttttttctcttagcctttgtttggcaagccgctgtaagttttcgatgaaatctttaatactctgctaagatgtatttgataaaaaattctaaaaattgatcagataagtcttacattacgaaccctcgattcaaaaatagaaattcttgtatattgaatgaataaccgcagcgatgaatttggatcagccttttccccgttctgaccttccggtgagtatggactattaggtactccacctaattattgatatgacaagaaatctcgggtaacgaatgaatcaaaatcttattacaaataaatttttatggggtgtcaacaaaaatggtatatgtggtaaaaataggcaatctattccccttaaaaaaaatgatcttggagattgtgtatcaattgaagaaataattggaaaataaaacagcgagtacaaaaatgagtaataaaccccagtatagactggtacgattcaattcaacattttgttcattcgggtttgattgtgtcatagttctctaatttgaattaggtttaatcattttgactgactgtttttacgtagataataagtaaaaaagcagtaggaactagaatgaacagtgcagtagcaataaatgcgagaatattgacttccataatttcattattttttttcttcgcaataactcgggatgtaatcccatagagatgataaatttaactcctgtaaattcattgggatgaattgatcctgatgatactgaataggatcaatattatgaataacaatatctgatctatcaaatcgattcatcgtcgagaattgaatagtataacatgggaagatcctttatccatactaattacgaaatgggattttttattggatcaggaatcccattggatttttcatcctcttacactttcttttctataacctactgtcttccttatcttatctccttcctgtgtattatacttacaattatgaggtattatatgaccggtattctatgggtcacacacagacccaaacgaggtgagatgaaaaatcaaatattccaacaaatttactgaaaagggtccttgctcggtcttttattagtatcctctttcttgtatttatttgtactggaatgcatacatcaaaacgatgtctgcaatttgaatgagatagattgtttacaattagtcattgaggatacacaaactcagaactagaaaaggtgtggtttaacctgaaatctgtcgaggtaattatggtaagttcattgtccatcgtacaataaacgaataccatttttgtatgtactcccggtaaaataggatcactctactccgatcaagaacaatctaaaaagtaagacgaggatgtcttaaaatactgaatatagtaataccaccgattgtactaatgtacatatgatatgtctctcctttatcaatcgggagtagtggaaagatttgaaattcccatatccgaaataaaaaacaaaagaaggatccccactggggattagatcttgcttcctgtccccttttccgtgaaaagagagagatgaattgagaaattcaccggatcctagtctagtccccgtgttcttcgaatggatctcttagttgttgagagggttgcccaaaagcggtatataaggcgtacccagtaaagcttacaagtaaaccagatatggagatggcgactagggttgctgtttccatttttagtcaagatcacaatggatctacgataagatcgtttatttacaactacaacggaatagtatacaaagtcaacagatctcaaccaatcattaaataggatttatggaagcattggtttatacattccttttagtctcgactttagggataatttttttcgctatcttttttcgagaaccacctaaggttccgactaaaaaaatgctttcgaaataatttaattgaagtaatgagcctcccaatatgggaggctcattacttcaaatgactattgctttccaattagctgtttttgcattaattgcgacttcatcagtcttactgattagtgtacccgttgtatttgcttcttctgatggttggtcaagtaacaagaatgttgtattttccggtacatcattatggattggattagtctttttggtagctattcttaattctctcatttcttaaacttatttggtatttccccgatccaaaaacaaaaatgtcactccttaagattcaatctgagttctgaaaaaattctatttcaatgttattaattggatctaataaataagattatcctaatctatcttaattggaatcttactttattctggccctgcacaaatatgatccagccgcatatatgatatatgtcatatatgtgtggacatatacgtgcgtatcaggaagtaagaaaatatgtcaccacaaacagagactaaagcaagtgctggatttaaagctggtgttaaagattacaaattgacttattatactcctgactacgaaaccaaagatactgatatcttggcagcattccgagtaactcctcaacccggggttccgcctgaagaagcaggggctgcggtagcagccgagtcttctactggtacatggacaactgtttggactgatggacttactagtcttgatcgttacaaaggaagatgctaccacatcgagcccgttgtcggggaggaaaatcaatatattgcttatgtagcttatcctttagacctctttgaagaaggttctgttactaacatgtttacttccattgtaggtaatgtatttggtttcaaagccctacgagctctacgtctggaggatctgcgaattcccccttcttattcaaaaactttccaaggcccgcctcatggcatccaagttgaaagagataagttgaacaagtatggtcgtcctctattgggatgtactattaaaccaaaattgggattatccgcaaagaactacggtagagcggtttatgaatgtctacgcggtgggcttgattttaccaaagatgatgaaaacgtgaactcacaaccatttatgcgttggagagaccgtttcgtattttgtgccgaagcaatttataaagcgcaggcggaaacgggtgaaatcaaaggacattacttaaatgctactgcgggtacatgtgaagaaatgatcaaaagggccgtatttgccagagaattgggagttcctatcgtaatgcatgactacttaactgggggattcactgcaaatactagtttgtctcattattgccgcgacaatggcctacttcttcacatccatcgcgcaatgcatgcagttattgatagacagaaaaatcatggtatgcattttcgtgtactagctaaagcattacgtatgtctggtggagatcatattcacgctggtacagtagtaggtaaactggaaggggaacgtgagatgactttgggttttgttgatttattacgtgatgattttattgaaaaagaccgtagtcgtggtatctttttcactcaagattgggtctctatgccaggtgttatacctgtggcttcagggggtattcatgtttggcatatgcctgctcttaccgaaatctttggagatgattccgtactacagtttggtggaggaactttagggcacccttggggaaatgcacctggtgcagtagctaatcgggtggctttagaagcgtgtgtacaagctcgtaatgaaggacgtgatcttgcccgtgaaggtgctgaaattatccgtgaagcttgcaaatggagccctgagctagccgctgcttgtgaagtatggaaagagattaaattcgacttcgaaccggtagataagctagataaagcgaaatagaaagataaaaaaaagcgtgtagaatttagtaattcctctttgttctcctaattgattgcgattaaactcggcccaatcttttactaaaaaaaggattgagccgaataaagaatgagcttcctaacatactatatatttgcatatatctttcatatcatatgtacagacctatatatatatacaagatctaaatagaacgaagacgaaacaactcgatatttctatttttttttggatccaatccataattaatcctcaaagctaagggaagggctccctctatccaatctactcatcctgtatattgtctttttcgttccatgttgcaatatttattatttgattatacgagaacgaattccttatttataaactattttcgatgagaatttgtttttaattaaaaaaagagatctttctcacttattattagttaacaatcctaatcctcatatgcttaattctgataggaaataaaatagtaaaataattattcatcgaatgactattcatctattgtattttcatcaaatagggggcagaagattatttataatacttcgggagctaatgaaactattttagtaaaatttaactgtctcaattctcgagcgatcgcaccaaaaactcgagttccttttggatttccttcttgatcaatgacaactgcagcattgtcgtcatatcgtattatcataccgttttcacgtttgagttctttacatgtacgtacaattacagctctaattacttctgatctttctagaggcatattgggaactgcttctttgattacagcaacaataacatcaccaatatgagcatatcggtgattaccagctcctatgattcgaatacacatcaattttcgagctccgctgttatccgctacattcaaaagggtctgaggttgaatcatatcatttttatttcaatctgttatttcaatgcaaaagtatgaaagaaaaagaaatattgtccgtccagaaataaataaacctgcgttttttcatccccaataccctttttgtttagttctatcctgaaataataaattgagttcgtataggcattttgcgcgcagctattgcgatagctgctctagctacagtttctgatactccacccatttcataaagtattcgaccccgtttaacaacggatacccaatattcaggggatcccttccccgaacccatacgtgtttccgcgggtcttactgtaacaggtttgtcgggaaatatacgtacccatatttttccaccacgacgcacatatcgtgtcattgctcttcgccctgcttctatttgtctagctgtaatccaagcaggttcaagtgcctgaagagcgtatctgccgaaacaaatatgattgcctcgacaagatattcctttcattcttcctctatgctgtttacgaaatctggttcttttgggactaagcatagcaattataccaaatgatcaatcgaatttttatttaaccttttagaattgttcttttttttaactgaaagagtttgtcattttttgttttatcactggacagaatgggaagacaaggttgaggttgtttcttaattccatctctactgcagaaccggacatgagagtttcttctcatccagctcctcgcgaatgaaaggattctaattcaaacacattaatagataatacaccttttattgatattttcttacaaaggaagatgtagatacaagatatacaatacagctagacgtgtgtgtacgtttatgtatctttaatgtaatgtttcttttatttttataacgaatcctttcctttttacctctatcgaataaggattgtaatccaataaatagaggtttcgcgggcgaatatttactctttcctgtttcattcgaaggttcaattcataacctctcagaataaatcaatttttcttggtccgttccgccatcccacccaatgaattattaggatttgttttcaatagaagcctatgcagtcacaggttctgtcgttcccatagcttctccattaatggttaggtccgaactttgcaatggagcttccaacaaaattcgttcccaagtcaatttcctcagtttttattaacctgaaggctctttattattttattctatttttgctttatcacactgccttttatgacgtgattcatagaccatacatattggaatcatatatcattgatatttttgttctttctttctatcatccttccatttatccacatccttttgctttacaacccataatcagatctattttttgttgaaagaatttcagttgctacaaccatatgatagatccactcattcatatagtgactgtttcttgggatctcgacaatacgaagcaataagttggttattagtttattttatataattacaaagtttatagcggggtcagtttttaatcccaacttgaaactataaagaaaaaactaacgagtcacacttatttgataattttattggaaatcatgtaaagacaattcttatttgatatagctatttgcgcaagtattttacgattaagaagcaattgcttcttgtacagattgtgcattaatatactataactatagaataccttattctcacgagttactgcgtttatccgagtgatccacaaacgacgaaaatccctcttttgtctgcctctatctcgatgagaggaaaccaaagctctcattttctgttgagtaatcgttcgagtaagtcttgaatgagcccctctaaaggttgatgcaaataaacgaatttttgttcgacgtctccgagctgtatatcctcgtttaactctggtcattgaatcagattaaagcttaatgaataactaattgatttctcttctttcagtcatccttttccccttccccggtcattaataacaaaacggattattccaatatataaaatatcaattccaatggcttttgctactatgaccttcccaaccacgattttgtattctattccttccatttatttcactggaaataataaattagaatgatactatagtgggttccatcgtttctatggttacctcttaaacggtgaggtcctctctatacaccggagcctcttctttcatttaatcaaatttattgttaacttgtatagttcacactctttggctctacctatctacagtaatagctcttttcacaaaaagagttatccatacagtgacggcatttaattatgaaagttggctaggtagctgaccctgttagtccgtcaagaaaaggagcataacctttttttatgataccatttcctccgcttaatggataaccctttgctaccaatggggaattgcttcttatttcaaatctagatgattggatttgcaccaatggaaaccataaattccatatacagggtatatgatagatcttctctatctatcctagatactgaaaaaattgtctcatttgttcgaacttatgatctgaacgagtcgcacctaaatcttagattgatctaaaaatgatttagatttatcttttaatacaattgttatatgacatgtcggtctttttatcagataactacgtcctcgagcccgcggtcttaactttttcacaatagtactcctattggcttcggctttactaatgaatgaatcagcttccttcaaacccatattatgattagcatttgccgctgcagaataaaccaatttgagaatgggataagatgctcgataaggcatgagttccagtatcataagtgtttcctcataggaacgcccgcgaatctgatcaattactcttcgtgcttttaaaacagacatacatatatgttgagctaaaacttttacttcacctgaacttgagttctttatcatgtttgattcacatctatttttctatttaagttcttttctctataagaggtggaatagaataacccggttgaagcgtaatgatcatacgtctgtaatgcattgtatgtcccattcttctaccctttccgggtagtcgatgactattcatagctattaccttgacaccaaagaagagttcgacccaatgctttatttctgtcctagttgatccggattcgacattagaagtatattgattgttccccaataaccgaatacttttttctgtaaatactgcatatttgattccatccataaatccattttcttccctatgagttccagtatcgataagaattctagttcttactgttcatatgttatggtatgaatataccataccaattcgttatgtatggatgatgagattccattgatacagagtcaattccaatagacttattgaacgttcccattggcgctatttacggcgacgaagaataaaactatcactatattttttccttttcctacttcttcttccaagcgcaggataaccccaaggggttgtgggtttttttctcccaattggggctctcccttcaccgcccccatggggatggtctacagggttcataactacccctcttactacaggacgcttacctagccaacacttagatccggctctacccaaacttttttggttcaccccaacattacccacttgtccgactgttgctaagcagtttttggatatcaaacggacctccccagatggtaatcttaatgtggccgatttaccctcttttgcaatcagtttcgctacagcacctgctgctctagctaattgtccaccctttccacgtgtgatttctatgttatgtatggccgtgcctaagggcatatcggtcaaaggtagggcatttcccattgatataggaacttctgtaccagaaacaatggtatctccaattatagcccctctgggatgtaaaatatatctcttctcaccatccccatagtgtatgagacaaatgtatgcatttcgattagggtcgtattctatggttacgattctaccagatatgtctttttgattccgtcgaaaatcgattttacggtatagacgcttatgacctccccctctatgccttgcggtaatgattcctctggcattacgacctttaccacaacgatgccgtccatagatcaaattatttcgtggattggatttcacttgactgtctacggctccattgcgtgttctcggggtagaagttttgtataaatgtattttgcttgaagtagattcttcttttttatcaataaaaacctcttcccaaactgtacaagcttcttccaaagcatacggctttctagatgtatatgatgatatctagacagatggatcttatatgaatcgtatgatgaagtaccacatgagtggatatataggaatccaaatctgccgaatcactcatgttatgatcttctacatcctaggtctccccgttccgtcatctggcttatgttcttcatgtagcattcagaccgaatgactctatgaaattacgtcgatacttccacatattacgggtaacgtaggagacatctctatttttcccccgggggatcttaattaccactgcttagctttcaattcgcctctgaccatcaaattaaatgtgaataacccgccctcctctctttgaaatttgaaacaaggggcgcttccggttctgtgcgtgcttcaaacaattttgtcttctccatattaccatatctctagagtcaataattttctatgaggaactactgaactcaatcacttgctgccgttactcaacagttttctgttgaggtctatcccgtagaggtactcaaattggatcagtgatcgatttctaggtttcgtcgtaaacctaattggttacttccaattacgtaaatcaatagttcaaaccgcatggcagttccaaaaaaacgtacttctatgtcaaaaaaacgtattcgtagaaatatttggaagaaaaagggatatttagttgcagtaaaaactttttctttagcgaaatcggtttccaccgggcattctaaaagttttttgtgcgacaaacaaataataaatctcaattgatatagcctaaagaacctcaattttgtaagatgaatgttaactaatgtatttttctgtattgaatttctcagatatatagaataagagtgggttctaagtattattttttccctatcgcaattgtacttttcaaaagtacaattgtgatagagattgaaactcttttgttatatgcctatcccaaactaattggttttgtaaatggtaaattatatgcgcaataaagaatattaatactaattatttgtatttaatgatgaaattgtgatagatatgaaatcctagttattttttaaaaaatagagaaaaaactatggagttccaactgtttggggaaaacttagtttctagtacatgaaagttgattgttaaaaaacccacgctaggtaggaggattcaaatatttaaaccacaaaccagtctttattcattgattctaattaatgtttcctaaactatattgaatccttgaatctcgacgattcaacatgaattgactattattatttcaagtaaatggccaagggtaaagatgtcagagtcagagttattttggaatgtaccagttgtgtccgaaatagtgtcaataaggaatcgcggggcatttctagatatattactcaaaagaatcgccacaatacacccagtcgattagaattgcaaaaattttgtcgctattgtcataagcatacgattcatggggaaatcaagaaatagatcgaaggaaacatcatgtgttcgatctttccaaagaacaggacaggaagagtaataacttatataatatacataatatatacaaatcaaacccgattttatgtagatattctttcatgtgtatagatatataatgaaataatatatgaatcaatatttcggttggatccgaaatgaataaataatttagagataaggaataaaccttacccttgtctctgtttatgtttcggattggaacaaatcactataattcgtccacgcctgcgaatcagtcggcatttttcacaaattttacgaacggaagcccttattttcatattttttattccttaattctgaatccacttcttggaagagaataagtctcttgaattttttttaacttcaaattgtatacccatggttaaaaaaataacttaaaatgagaacttattcttgggtaaatcaattgcgaaatgcttatgtagagtgtccaatatttgttttacatcttccatgcgaaaatattcaattctcataagatcttctttactgttactcaaaaggtccaataatgtatgtatattggaccttttgagacaattataggtcctggaaggcaattctaattggtcaataaaaatacaattcaatggaattccttttttgtttttctttagattagttaatctatcttgaaaggttaaaaagggtagagtaaacctgtttttattttcttcgaaattaatgtcctcttcctccgcatatagaaaaggaataaataaatcaatcaaattacgagaagcctcataaagtgcttccttaggagttaaacttccatttgtccatatttctataaaaagtatctcttgtttttcattcccataagaatgaatactatgattcgcatttcgaacaggcatgaatacagcatctatagggtaacttccatcttgagagtcatttatgggttccatacgatatccgcgatctctcttgatttgtaatccaatacacaaatcaattggttccgtcaggttagctatatgttgtgtcgtgtcaactatttctacggaaggtggtgagataatatcttgagcggttacgtatctaggaccccttacgcaaattgacgcgtctctaactccatagagattacttctcaatacaatttctttcaaattttgtaagatttcatgtactgattcctcaatacctactatcgtagaatattcatgtggcaccttcttagattttgcacgtgtgatacatgttccttctatttctccaagtaaggcccttcgcatggcaataccgatggtatcagcttgacctttcataagtggtgacagaatgaaacgaccataataaagacgcttactgtctactcttgattcaacacacttccactgtagtgttcgagtggatcctgctacttcctctcgaaccatactatactagtattattatttgatcatttatttctcttgaaatcggtttaattcttattttcaagcttccttcctattaatctggaagttcttctcagatacaaggaaatgattcagttctagagccaaagatcgtagttctcgaatgagcaatcgaaaagattcgggagcatcctcggggttaggtactcttcctccaataatcgtagcaccaagtacttcttgacgagctctaatatgatcagatttataagtaagcatctcttgtaaaatatgagcaacaccaaatccctctagagcccaaacttccatttctcctactcgttgtcccccctgcttggcccttcctctaaggggttgttgtgtaacaagtgcgtaatgtccactagaacgtccatggattttatcatcaacttgatgaattaattttaggatataggactttcctatttgaacaggttgttcaaacggatctcctgttcttccatcaaatattctgctttttccaggatactcgggttcaaatacccatggatttcttgtttgcttactggcttcatataattcggaaaacactagttttctcgaagcctcttgctcatatctctcatcaaagggtgctattctataatgtctctttagcagattccctgctaacccgagcgagcattcaaatatctgtcccacattcattcgtgagggtactcctaaggggttgaagaccatatcaacgggtgttccatcttgcaaatagggcatatcttgtctaggcaaaattttggaaatgatacctttattcccatgtcttccagctactttatcacctactttgatttcacgtttctgtaaaatatatacacaaatcatttctgaattataactggaaccaccctttttctggatccatctcacatcaataacgcgaccccttccacctataggtagttttagagaagtttcttttgcagtggatacctgcataccaagtatggctcgtaataatctatcctccggggaatacgaggattcgctcgctgtctgaggcgttaatttacctactaaaatatcacctgtttctacccaagatcccagcatcacaattccatttctgtccaaattgaggagtaaatgagcctccagatgcggtatttccttagtaattctttcagggccttggcttgtcacatgagtctgaatttcatattttcggatgtgaaaagaagtataaatatcttcatataccaggcgttcgctaattagtactgcatcttcagaattgtaaccctcccatggcatataagctactaatacgttttttcctaaagcgagttccccaccaactgtagccgcaccgtccgctaaaatttgtccttttttaatgcatttaccccgccgaacccgaggtttttgatgcatacaagtatttttgttggaacgttgatacataactaatggaatacttatagtgttcccattacttgataacatgatcttgtgagtatcagtataaatgacctttccttcgtgttcggctataacagaaacccccgaatctagagccgtttggcgttccagcccagttcctacaatgcacttctcggaccgagaaagcggaactgcttggcgctgcatattagaactcattaaagcccgattcgcatcattatgctcgataaaaggaatgagggaagctccaatagaaaaatattggaaaggaaaaatgcttctaagatgaatctgttcccatgcaatagtcaggaattcttgacggtatcgagctggaacaacctgttcttcctgaataccccgattcaaggccaaagaatttcctgctgctaacatataatattcatctctatttggtgataaataaaccatctgtggctctttttctttttgtttctcagatatttcataaaacggactctctatagatccccaatgaccaatcctcacatgaatagctaaggatccaataagtccaacattgattccttcggacgtgtcaattggacaaatacgtccatagtggctaggatggatatctcgtatacgaaaactagcagttcgccccgtcaatcctccaggacccaaataactcaattttcgtccatgaacaatttgtgtcaatggattagttcgatccaaaacttgagataaagggtgtaggccaaaaaacgattcataagtagttgttaatgaagttgaagttaccaaattttgaggagtcggtatcaatttatgcctaattgctccacatatagttcctctaaccgcattttctaaacgaacaagagccaatccgaattgatcctgtaacagatctgctacagaacgaatacgtttatttttcaagtgattcatatcgtcaaatgtacccattccaaatttcattccgatcaaatgatccgcagcaaccaatacatctcgtggtaacaaaaatgtattgttctgaggtatatcaagattcagtctccggttcatatttcgtcgaccgatccttcctaattcacatctttgttgaaaaaatttcttttgtaattccttacataaagactcagaaaataccggatccccacctacacaagcaaattgttgataaaactccaaaatagcattttcttttgacccaatcttttttttctctttatcattcgggaaagacaagaaaatctcagggtaacaaacattatctagaatttctcttagatttgaacccatagctgatgatagaactagaatagatattttttgtttcctactcacacgggcccatatccttgcttttctatcaatttcgaattccaatcttcctccccaatctgatattatagtgctggtatagacagaaattccgttatggtccaattccgaacggtagtaaatacctggactttgcaatatttgattgatcacaattcggtatattccatttactatagaggttcctagggaattcattagaggaatgtttccaataaaaacggtttgttcttgcatatctttactggttttccaaattaatcccgcgggtacatataattccgaaaaatatgtgagtgattcatacacagcatctctttcttttatcaagggttctaccaattgatatctttccacaaataatttaaattcaatttcttgatctgtatcttcaatttttggaaacttatgaaattcttccgtcaagccttgattaatgaacctacaaaatccctcaaattggatctgactaaatccaggtattgtggacattccctcatttccattccggagcatcttaatcttatttaatcccattattgactcactattcatcgacccgtatggatcgatctagcaatgatggaatgtatattctgtttactgaatcacatgaaattttagtgaactccatatatgtatttcatacgtatgaatggaaatgaattgataaaacattcctgaaacaaattctgtcacttacacttatgaagtcttgtatagatacaatataaaaatggatccaatttccacctataataatgatattacgatcagattgggtaccaaggattcacgattaaatctgctttctatgaatgagataaagacagaataatcaggagcactatccagttttactttactttatttagatttagcacacttaatttaatgaaaattcaaagattttttgatagtagtctaatttatagactaggattgaattgcataatcataaaaggagtagtattttttattcaatatattcaatacaatgaaagattaaagcacggtgattctctatagggtacataagagagagacccgactcggtatctgtgtaccaatttctgttctggggtttacatataatatatattttcttaaaagttaatggttccaatttgccccaaattttgcagtctgtgactggaaatccatttttttttcaatttgaaatagtagaaaagggaagtttttaagcggtgtgtgttttgagatacaatcaatcgaagagactaaactagatcccataagaaacaagaattcatttttggaaagagattgaaaagggataagcacaagagattcaagatcaaaaaaaaataaaaaagaaaaattaatattttcatcgattttggatcggatttttagatagtgtatgagtaggcccgacaaaacccttgtatggcttcttctatttctcgataaaaagaaatatgaccaagagtggttcgaatatatatacaacggatttctttttttacacttcctactattagatagtgcccataaatctcatgataagtacccaaggattcatattgaacttcgatgggaacttctcttgacccaatgacacgttgatctagtctccatcggagccacaaaggactatctaaactgattcgtttccgccgataagctccaagtgcgtcataggaactacaaaaatacggttctttctctttcgtatacttataattattattgtcaactgtttgatatggattatacctatttgcacaaatacctcgacgattcccgatcgttaatacatagagtccgataagtatatcttgggttggtacggaaatgggatctccaatagctggagacaagagattcatatgagaaaacataagtaaacgggcctccgcttgagcttccaaagataaaggtacatgaacagccatttgatccccatcaaagtctgcattgaagcccttacaaactaatgggtgtaaacaaatagcacgtccctccactaaaatgggttggaacgcctgtatgcctaatctatgcagggtgggtgctctattcaacaatacaggatgcccctgcataacttcttgcagtatttcccatacaatcggttctttttcccgaattttacttttagcaatccctatgttagaaacaccatgttgcctgattagaccacgaattacaaatgtttggaaaagttctattgctatttctccaggtaatccgcattgatgtaatgaaagcgaaggacccacaacaatgacggaacgccccgaataatcgacccgtttaccaagcagagtctcacgaaaccttccctctttcccttcaattacatccgaaaatgacttgtaaactttattatgaccatccctcattggttgcccgcggatcccattatcaagaagtgtatccacggcttcttgtactaatttctcctgacacattactaattcccctggtgtagatctacttgttgctaatagatcggtaagagtattgttccgatagataactcgtctatagagttcattaatatccgaactcattagtttccctccatctatctgaatgattggtctcaattcaggaggaagaactggtaataagcacaaaaccatccgttccggttctacttttgttcgaataaaatgtttagctaattccatgcgtctaaccaaaaaatcctttcttcttctaattttgctatcttcccattcatttccagtagacccttcgtctcctaattctttccattctgccaatgaattatctataataattcgaaaatccgaatcagctaattgttctctgatagcacctgctcctgtagagatttctcgatttcgaaatatctcgaagcctgaggtagtaaaaaaaagtgggatactgtatttccgggattggatttcatattcgaatgaacctcgtaatcgtaagaaagtaggttttttagctatgggcctagcaaaagagaaatcgcagtatactaggccttctaattgcttaaggggtttatctaaaagattcgcgatataactaggaagacgtttcaaataccacacatgagtcactggacatgcgagtttgatgtatcccatttgatatcttcgtatccgagagtcaacaaattctaccccgcattgttcacaaaatttagggtcttctttttcagctctgatcgctcgataatttccacaagcacaaattccactttttatgggtccagagattctttcgcaaaacaatccatctttttctggtttattggttttataatgaaaagtatagggttttgtcacctctccaactatctctccattaggtaggattttgttggcccaagcctttatttgttgaggagaaactggtccaattcgaagttgttgatgtttatactggtcgatcatagaatagaaatgatgattcattcagatataggatcggattccccccctttaaaatcggacgtgaaggtttcctctcatccggctcaagtagttacaccaaataaaaaaggaggtgaaactttgttcaaaaccctacaaagagctactccttactcaagttcccagtaagaaccaacctttcattgattcattcttcttttatgacaaaatggaaatgtgaaattattgagtagtctattccccgatgaattttttaaaagaaatactttggaattcgtaagggatttatttgtctatatatcgttccattcgatcttttaggtctctactcacctcgatggttatgccatgatgtcccttgaagcatatatgcgatagatagactcctgtaaccatgctatatttgcttgcttaaacagaatttctctctaaaagaaatggaatggctaattccacgaaaaaatcttttttcacgaggtataactggctattttatacggaatcgaccatggatcaattcccctttcatttggaagtattgaatacacccataattctattctgagtttcatgttattttttccaaaacacatgtcagagtcgggggcatcccaatcggattgaatgggatgacagtttatcaatccgaatctgtaaaatgaaaatcttgatcaaatcaaaattgtttgtcgcgtaagaacaaaataattctctatggtgtaacaaaatatctctcatttccaactcgaatagattttttctttttatttccaaataaatgtttttgtcttgccttgaacggtgcacaaatttttggaatccggtaccaacaggtataatcccccccagaacaacgttctctttcaggcctttcaaccaatcaatacgacctcgtagagcagcttttgctaaaactcgagcggtttcttgaaaacttgcttcggatatgaaactttgagtattcagagacgctcttgttattcccaatgagattgcccgataacagatcgattcgtccaaagcgcgccctgctcgttccgctcgcaacaatccgattaattctccaggcgaaaaaacattagacattccatcttctgaaaccaacacttttgatgttacttgacgtacaataatctctatatgtctattatggatctgtaccccctgggatcgataaaccttttggatcttattaaccaaagagatacgactttgggctatggttagctcagctccaatcaaaaacccccaggggatcccaagaattcttggtatacgctcgttccaaccttcaactctcctttcgaggttcatcgatattgaatcaatcgaacgcacttctaagatttgttctacttttggaagaccttgcgttatgtcaccagatctcgatttttcatatataaatgtaactaatgtatctccttcgtaaaggattttcccataatgaccatgaacagttgctccaggagtagccaaataagacttagccgatcttataactaaaaagtcgacattaacaattaaaatttgaccagatttttttacgtgtgattcgtatttaaatagacatacattttcacaaataaattgtccaaggctaattttgatcgatgtctcttcacaataatcatgatggagaaagcaccaattcaaatggaatgggttcaaaatgatgttactgcatagatcgggattataaatccttctattttcatctattaaacagtatttaagtacttgaagtacttggaaaatctgtttcaaattgtcaagtagcaaatatttctttaacacgatctgattatgagttattaaatggtaagatgaataaaaatttgatattttaggtacaatagcgcctaagggacctaaataatccctaattggaattaggggatccgattcttttgtcacattgttatatttcgaactattgaatggaccaattcgagaacaattggatgatgacaaaattagcaaagattggcattccttatttcgattcaacaacataccaatagttccttgatgttggctaagtgattgaatcttcgccttggaataaaaaggattgatattggtgcaatctaatccattatcgggaatcaatccggaacttgccctatcatacctttttccggtatacgaaatagtggactcaattcttatgaaatcgcgaattagatcatttgcccttacctcaacaaaggaagcatgaacctcttctatagaaccattttgttcttggtcccaattcaatactaagcaagtccgaactaattgaatacttgtgtgataaattcctcgaattgacttgccatttccataaaggatataattgacaactctaaattggacattatccttttcctgcaagatatcacgagggaaaagtgttgctaaatttatcccatcggatatttcatatgtgactacgggtcgaaccaaaacaaaatactttttcttggtaggtgcgatccgttgaacatagatccaatttttccatttttttgattctttagaattttttttttccgtttctggtggtataaaaatgccgctgtgcctggatatcttatctgtctctccaggaaaatgaatatctccagaaaagattttaagttcaatatattttttttttctctccactcgtactaatccgcctactcggcttcttgtatttaaagccagtcgtgtatatactccaatgatactattgttccggaccattattaatgaggatcccggtaagatatgcacttcttcgagaatgaaaaaaaaccgatctactttcatttggtatttcggactaaattcttttgctcctcgatactcaatcaaatcttctttttttatgattgaatccacctctatggtcccatatttagtaattccggaactgcttcttctgtatcgtggatcatcaaaataagcaagaatactatttccacgtaaaataccatttatgggtatttcaatcgaaataccaaaacagggtattagttcttgatcatattgtaatgggatgatgaatctatttcttcgcttttttgccaagaaatcaggattctcttggagaatagaaggatatattaaattccaatgatcattggatatgattcgatcaagtcttgaatagtcaagaatttccctatcttttttaccagaagtatctaacaatttatgtcttacttgatcattagtcattgaggggttatagatatatctttcttcaacagaaagagaataaacattcatttgatcttgatccttgtggagcgaaaaagacactatactggatctgcacgggcctcctgctaatatccataaatggcttgtttttggtaatagatgaacattaccatatgtatattcaggtgcatggtagacatcggtactccagtgcatttctccctctgattcagaataaatatgttttcgtaccctctctttaaaatgaaaagtggacgttccagcacgaatctcagcaatcacttgttctgattctacatattgatcattttgaactaaaatcaaactttttggcggaatagtcacattatatataatatcccgactctcaatagttacatacaagtctatagaacatagaaaagcaggatacccatgacgggtacgtgtgggatgaaccaaatcctcattgaattttatttttccattagaaggagctcgtacatgtttggcagtaccgcctgtgaatactccgccggtatgaaaagttcttaatgttagttgagtccccggttcgccaattgattgacccgcaataatacctacggcttctcccaattcgaccaaatcgccatgagtgggactacgaccataacataattgacagatccaagatgtactcctgcaagtaaagggggttctaatatatattggttgtgctcgaaaggttatgaatcgattgacaagtccaatcccaatatcttgatttcgagtggcaatgcatcgtagaccgatatatatatcgtctgctaatacacgaccaattagtgtttggacaaaaattttttccgtcatctcattttgaggactcacggagatacctcggatagtaccacaatctcttctacgtacaataatgtgttgaactacttcaacaagtctacgtgtaagatatccagcatctgatgttcgtatagcagtatctacaactcctttgcgggctccgtagcaggaaattatatattctgtcaaagaaagtccctcgcgtaaattgctttgaatgggtaaatcaatcatttgtccttggggatctgacattaatcctctcatacctactaattggtgtatctgagatgcatttcccctagctcccgaaaaagacattagatagactggattagagggatcagtcatccgaaaattaggattcatttcttgtctcaaatattcacttgtagcataccatatctcaatagattgacgtaatttttctaccgcatgtacattcccataatgatggtgtttctccaaaataaaactttgttgttcagcgtcttggactaaccatcccttagaaggtattgttaaaagatcatcaattcctaatgaaatagatgtaacagtggcttgatggaaacccaaagtctttacttgatccaggatatgtgatgtatatcccattccgaaatgatctattaatctgctaataagtcgtttcatagcagttccatttatcactttattgtgaaagaccagatcggcccgttctgccataagtacctctatattctgctgagtaggattcgacaatggacctgagtcagtgattcgaaatttcctcaatttttattcacgcagaaagaaaattggaaaacccgatttcttagtctacacacgtctttttttaggaggtcgacatccattatgtggcataggtgttacatcacgtacgaagcttaataatataccacttctacgaatggctcgtaatgctgcatctcttccgagaccaggaccctttatcataacttctgctcgttgcataccctgatcaaccactgtacgaatagcatttaccgctgtggcttgagcagcataaggtgttcctcttcttgtgcctttgaatccagaagtaccggcggaggcccaagaaactacccgacctcgtacatctgtaacagttataatggtattgttgaaactcgcttgaacatgaataacgccttttggtattctacgtccattcttacgtgaaccaatacgcccattcctacgtgaaccaattcttggtatagcttttgtcatattttatcatctcatatttatgagtcagaaatatacaaaaaaaaagatacggggatacccatttcatgttaaaacggatcctttaccttatttttacattttggaaagttctttttagaagaaatacaccctagtacatgttcctcgacgctgaggacatcctctaagagcgggagattttttaacatttcttattggctgtcttgtgtttctaataagttgtttaatagttggcatttattttggctttttaaccccatatttagaacgcccttgttgacgatcctttactccgacagcatctagggttcctcgaacaatgtgatatctcacaccgggtaaatccttaacccttcctcctcttactaatactacagaatgttcttgtaaattatggccaataccaggtatataagcagtgatttcaaatccagaggttaatcgtactctggcaactttacgtaaggcagagtttggttttttgggggtgatagtgtcgtatgtatatatatgtatatatagaataaaatgggttggtttagatcgatcttaacctgatgattgatcatcatgaattatttctattcaatatcaaatcacatttaattatggtttgaaataaaatggatggaaaagttgacagataagtcccccttactgtcactctacagaaccgtacatgagattttcacctcatacggctcctcgttcaattctttcgaagtaattggatccttttcttcgttcgagaatctcctcccttcctccactcaagggtaactaagaccaattcagtcacgttttcatgttccaattgaacactttccatttatgatcaaaggagatctttttaccaaacatatgcggatcaaatcacgatcttataattctaataagaacaagaaatctttctcgatatcaatccctttgcccctcattcttcgagaatcagaagttttcgagtttcaatttgttcattttgaatctgggctcttctatcttctacttatttttgatattgacttattttgctttattctttatttcatttcgattttttcctcttcctctatccctatcccataggtatagcgtttgaatcaatagagaaccttttcttctgtatgaatcgatattattacattccaatttcttcccgatacctcccgaaattgacgggttagtgtgagcttatccatgcggttatgcactcttcaaataggaatcaatttgatgaaagatcctggctttcgtgctttggtgagtcgtccgagatcctttcgatgacctatgttgaagggatatctatatgatccgatcgattgcgtaaggcccgcggtagcaatagaaccggggaaagtatacagaaaagacagttctcttctattatattagtattagttagtgatcccggctctgtgagtcctttcttccgtgatgaactgttggcaccagtcctacattttttctctgtggaccgaggagaaggggggctcagcgggaagagggttgtaccatgagagaagcaaggaggtcaacctgcttcaaatatacaacatggattctggcaatgcaatggagttggaccctcatgtcgatccgaatgaatcagtctttctacagaggtaaatctttgcctgctaggcaagaggatagcaagttacaaattctgtctcggtaggacatgtatttctattactatgaaattcatatttcttgtatgtgttcctaagaaaaggaatttgtccatttcatttttcggggtctcaaaaaaagggcgtggaaacacataggaactcttgaatggaaattgaaaagaaatgtagctccagttccttcggaaatggtaagatctttggcgcaagaagaaggggtgatccatatcatcttgacttggttctgcttcccctctttttttaaccgagtcgggttcttctcctaccagtatcgaatagaacatgctgaacaaaatcttcttcatgtaaaacctgctcgatttagatcgggaaaatcgtacggattttatgaaaccatgtgctatggctcgaatccgtagtcaatcctatttccgataggagcagttgacaattgaatcccattattttactatccataatagtgcgaaaagaaggcccggctccaagttgttcaggaatagtggcgttgagtttctcgaccctttgccttaggatttgttaattctatttctcgatgggatggggatagggtggatctcgaaagatatgaaagatctccctccaagccgtacatacgactttcatcgaatacggctttccacagaattctatatgtatctatgagatcgagtatggaattctgtttactcactttaaattgagtatccgtttccctccttttcctgctaggattggaaatcctgtattttacatatccatacgatcgagtccttaggtttccgaaatagtgtaatgtaaaaagaagtgcttcgaatcattgctatttgactcggacctgttcttaaaaagtcgaggtatttcgaattgtttgttgacacggacaaagtaagggaaaacctctgaattcaatattggaccttggacatataatagttccgaatcgaatctctttagaaagaagatcttttgtctcatggtagcctgctccagtccccttacgaaactttcgttattgggttagccatacacttcacatgtttctagcgattcacatggcatcatcaaatgatacaagtcttggataagaatctacaacgcacttaccaacttgatcttgttgcgcccggtaacaaacatgcataaaccatttctcgaagtatgtgtccggatagaccaaagtctcgatagttagctctaggtcttccagtcaaaaaacaacgtcgatgaagacgtataggtgcactattacgtggtggggattgcaattttccatgaatttcccatttttcactcaacgatgaaactttgcttattttttcttttgaggatcggcgaatcaaatgatatttctgttccaaatttttccgcttgttctctctctgaatcaaactttttcttgccataatgttcagttcctattattatcacagttcggatcctccctctccatcgaaacaaatgaaattgttgctgatacagtacataaaaaaacaataaataactaaattaacgagtttttggctctcgaatatccaactgaccaattaatttcttataacgtactctatttttctttgacaaataagccagcaaccgttgacgttttcctagaatttttcgtagacctctttgtgataaaaaatcttttttgtgcaattccaaatgtgaagtaagtctccgtatcttattggtgaaactgaatacttgaaattcaacagaacccttgttttcttctttttcttcttgtggaataatggaaatgaatgaatttttgaccataaaaaatttttctatccttttttttctttttcatggatttttacgatcaggaattatgccggttattttaatctagtacacacacaaatttggatttattcatatttctttattttatccaaatcaaattttcaatttgatttggatagaaagggataatatgtttccacattaacgaaagaaaagaatttatttctatctaaaaggattcccctaatttatttattcctatatccattcaatctgtcatacatctttcggctttcatataccgaaaatgtcacggaatatagatatgatataggaaatatactattaaactcaaagttctttctttaaagaattctgccttctttaaaatatcataaacagttcctgtaggttgagcacctttttcaaggaaatatagaatagcaggaacatttaaataagtttgattctttatcggatcgtaaaaacctacttttcgaagatctcttccttctcttcggaatcgaacatcaattgcaacgattcgatagatggcacgttgctttctaccacatcgtttcaaacgaagttttaccataacattcctctaattgaaattgcaaagcggtatggaattgattcaatataaaatcatgaatagtcattggttcaaccggtatataatatttctatctacggataaataagtatttatccggataagggtcagcaaggatcaaatttatttaatttaaccccatatcatatgaattgaatgaaaagatattcaattttacccacatttgacacttgattccgtttgtgagaaaccaaacgaattctcagatatgattcttagaaccattctgaaaattaataagatttttcccttaacaaatttttgtttcatgtggaatgcagtgtgatcccatctttcgtttcatgaaaaacgatattgggatagatgtaaataaacaatgccccccctagaaacgtataggaggttttttcctcatacggctcgagaaaaatgattccaatttctgtatataatagtggatccataaaataatgaattttactataatttgaattgagtacttttttttcttacttacagaaaaaaagaaatctcattcatactcataactcaagttgggtaattctgacaagaaaatccttagacatttattgagccgtctctaaactcttttgtttgtctcatttcgaatctatttttattcctcagcctgatccaattgttgagacaattgaaaatagtgtttccttgtttcggaatcctttatccttgctttgtgaaatcattgggtttagacattacctcggggatccttattcctttaaaaatggcagcaacataccttttttgttatttctttctatataaatagaaaatgattgattcccttgtgatacacttttcatcgaaatagttttaccaaaaatttgacttttctctgaatttgaacctttcgatttagaatttatatatacttacagagttggtctaacttattgattttcactaaccctagattctttcccttgaaaaatgaatcaatcctttcttctcgagcttcatcatgtactatttacttataacccaacacaaattaggttccgggcagaacaaactatgtcgagccaagagcatcttcattactatagaagatggcagatgtaaaaatccacagccgaccatgtccttcaagtcgcatggataaatccaagcaaccttttcgtaaatacaagcgatcttttcgtaggcgtttgcccccaattggatcgggggatcgaatcgattatagaaacatgagtttaattagtcgatttattagtgaacaaggaaaaatattatctagacgagtgaatagattgaccttgaaacaacaacgattaattactattgctataaaacaagctcgtattttatctttgttaccttttcttaataatgagaaacaatttgagagagctgagtcgatcccaagaactactggtcctagaaccagaaataaataggcatactcctcaattgactcaaaaatccaattggaactcaagctcagattgatttttgttcgaaaaggcctagaatcctgatttgattcttgtgttataatataagaaacaaaaatgggggagaagaagaaatatttttttattgaaacatgttcgttcatttatttatttttattctatatcttcccggagttcattctccggggaattccctttcaatcattcctgtatattacttttgaattccctttaacgacgagatttattatcgtttcttgcatgtctcgcgaaagtcagagtaggcgcgaattctcccaatttgtgacctaccatacgatctgttatataaataggtaaatgttcctttccattatgaatagcgattgtatggccaatcattgtgggtataatggtagatgcccgagaccaagttactattatttctttctcctccctcatgttgagtttttccatttttcccgctaaatgattagctacaaaaggatttttttttagtgaacgtgtcacagctgattactccttttttttacattttaaagattggcattctatgtccaatatctcgatctaagtatggaggtcagaataaatacagatgaatggaaaaaagagaaatttagctagataagatcaacgatttcttatatagctagaacgaccttcacaaattgcggatactaatttgttaagaatcaatcgaattgaagctatagcgtcatcgttggctggaatcgaaatatctgcaagatctgggtcacaatttgtatcgattaaacaaatcgttggaatccccaaaatggcacattctcgaagagccgtatattcttcttgctgatcaacgatgatcacaatatcaggcaatcccgtcatatatttgatcccaccgagatatgtttgcaaggtagataattttctcttcaacattgctgcatctcttttggggagacggttgagtttccccatcttttcttctgctcttaagtccctgaacttataaagtctcgtttccgtagtggaccaattcgttaacataccaccgagccatttttgattaataaaatgagaccgagcccttattgcagctgatgctactgaatccgatgctttatttttggtaccgacgattaagaagttttttcccctacttgctgcatcaaaaactaaatcacaggcttctgataaaaaacgagcagttctagcgagatttgtaatatgaatacctttacgctttgcagaaatgtaaggggccattctaggattccatttcttagtaccatgaccaaaatgaactcctgcttccatcatctcttccaaattgatgttccaatatcttcttgtcattttttcccacacttcctttttgttttttctttttcgtattattaacaaagagacgaggtaccttgaaataaaagattgttccgatggaaccttctaccggggattgaccattgatacacggcgcaaaccataaatttttttaattacttattttatttattaccaaatcaataatcagaccagtatagttaaaagatagttaaagtgaaaatgaatctgctcttattcctcaaatatccaaatttttatacctaatactccataaatagttcgaattgtataggaacaatgatcaattttagcgcgaattgtttgtaggggaactctaccctctctgatccattcaacacgtgcaatttcttttccgtcgatacggcctgctatttgcacttgaattccttttgtatccgtttgttcagttaattcaatagcttttttcattgcttttcgaaacgaaactctattttttaattgtaaagctatatattctgcaagaatattaggttgtccataaggtttttcaactcttgtgatagcaatgttgagtctccggtttacagaattaaactccttttgtacattcatctgtaattcttcgattcctcgtgtttgcccctctattaataaatttgggaatccaatatagattctgacttggatcaaatcgatttttttttgaattgttatacgtgcaattccttcgaaacctgaggatattttcctatttttttgtacatagttcttgatacaattccgtatttttgcatcttcctgtaggtccccggaataattttttggttgtgcgaaccaaaaggaatgatgactttgagttgtaccaagtctgaaaccaagtggatttattttttgtcccatatttttctattcgattttttcatccatatattttatatgaattcaagtctgacgagaataatattctacgactaacaactcatttatttgcaaaccgatccatttactatctattatttgatttactaatcctttatattggaatgagtcaatagtcaaatgttttggcaattcctcatgggcggatgaagcaatataattttgaatcagaccttttgatctttggttatccttcgcagtaataatatctcggggtttgcaacgataacttggtatatccactatacgaccattaactaaaatatgtctatggttaaccaattgcctggctccggggatggtcgaagccatacccaatcgaaagaggatgttatccaaacgcatttcaagtagttgtagtaaaacctgacccgttgaccctttggcttttccagcgatatgtacatatctaagtaattgtcgctctgtcagaccataatgaaaacgcaatttctgtttttcttctaaacgaatacgatattgcgattttttcccagaacgcaattggtttttaagatcacttccggatctaggtcttttactagttagtcctggtaaagctcccagacggcgtatttttttaaaacgaggtcctcggtaacgagacataaagactccttttttattttattgaaatttttacacaataaatttaaactgaactaaaggataaacaaagcaaaatcgaaaaatgaattgtatcaacatctagattttatatatatagtgaggctccgtttgatgatttgttctgtagagatctaattgctctaatcacgacataatagatcgctgatccagcattttatttgaagaaaaggagagattatcaatctcggaaaaatagatagagaaattaacgaaaatgtgcaaaagctctatttgcctctgccattctatgagtctcttcctttttgcgtatggcatcgccactccctttggcagcatctactaattcggaacttaatttgaaagccatatttcgacccggacgctttcgggatgctcctaataaccaacgaatggcaagtgcttttccttgtgtagatcctatttcaataggaacttgatgagtcgatccgcctacacgtcttgcttttactgctatatcgggagttactccacgtattgcttgacgtaaaacagatagtggatttgtttctgtcttttgttgaatctttttcacggctcgatagataatttgataagccaatgatttttttccgtgtttcataatacggttaaccaacatgttaactaatcgattacgataaattggatcggattttggagttttttcttctgcagtacctcgacgtgacatgagcgtgaaagaggttcaagaatcagttttctttttataagggctaaaaacgaatcacttaccatatataacacaaaatttctcctccaattctttctagtcgagcttctcgatctgttattatacctcgagaagtagaaagaattacaattcccattccacctaaaatcttaggaattcgttgatagttggaataaattcgtaagccgggccggctgatacgctttaaaatgattctagttttatatattccttttctggtttttctatgtcgcagagttgaaaccaagaaatatttgttactctcctgatgtttccgaacgttttcaataaaaccttctcgtagaagtattttaacaatgttttcggtgatatttgtagatgctattcgaacccttccttttttatccgtgtcagcatttcttatagaagttattagatcggcaatagtgtccctacccatgacgaactagaattataggttcccccaaattttgatataatcaacatgtttcctataaagtatatacgtgagacacaatctactacctactatatcatagtctcatctactactattctcatggagagttcgatcctggctcaggatgaacgctggcggcatgcttaacacatgcaagtcggacgggaagtggtgtttccagtggcggacgggtgagtaacgcgtaagaacctgcccttgggaggggaacaacaactggaaacggttgctaataccccgtaggctgaggagcaaaaggaggaatccgcccgaggaggggctcgcgtctgattagctagttggtgaggcaatagcttaccaaggcgatgatcagtagctggtccgagaggatgatcagccacactgggactgagacacggcccagactcctacgggaggcagcagtggggaattttccgcaatgggcgaaagcctgacggagcaatgccgcgtggaggtagaaggcccacgggtcgtgaacttcttttctcggagaagaagcaatgacggtatctgaggaataagcatcggctaactctgtgccagcagccgcggtaagacagaggatgcaagcgttatccggaatgattgggcgtaaagcgtctgtaggtggcttttcaagtccgccgtcaaatcccagggctcaaccctggacaggcggtggaaactaccaagctggagtacggtaggggcagagggaatttccggtggagcggtgaaatgcgtagagatcggaaagaacaccaacggcgaaagcactctgctgggccgacactgacactgagagacgaaagctaggggagcaaatgggattagataccccagtagtcctagccgtaaacgatggatactaggcgctgtgcgtatcgacccgtgcagtgctgtagctaacgcgttaagtatcccgcctggggagtacgttcgcaagaatgaaactcaaaggaattgacgggggcccgcacaagcggtggagcatgtggtttaattcgatgcaaagcgaagaaccttaccagggcttgacatgccgtgaatcctcttgaaagagaggggtgccttcgggaacgcggacacaggtggtgcatggctgtcgtcagctcgtgccgtaaggtgttgggttaagtcccgcaacgagcgcaaccctcgtgtttagttgccaccattgagtttggaaccctgaacagactgccggtgataagccggaggaaggtgaggatgacgtcaagtcatcatgccccttatgccctgggcgacacacgtgctacaatggacgggacaaagggtcgcgatcccgcgagggtgagctaactccaaaaacccgtcctcagttcggattgcaggctgcaactcgcctgcatgaagccggaatcgctagtaatcgccggtcagccatacggcggtgaattcgttcccgggccttgtacacaccgcccgtcacactatgggagctggccatgcccgaagtcgttaccttaaccgcaaggagggggatgccgaaggcggggctagtgactggagtgaagtcgtaacaaggtagccgtactggaaggtgcggctggatcacctccttttcagggagagctaatgcttatgcttgttgggtattttggtttgacactgcttcacacccaaaaagaagcgagctacgtctgagctaagcttggatattgaagtcttctttcgtttctcgacggtgaagtaagaccaagctcatgagcttattatcctaggtcggaacaagttagttgataggatccccttttttacgtccccatgtcgccacacgggagggacatggggacgtaaaaaggaaagagagggatggggtttttctcgcttttggcatagcaggcctcccattgggaggcccacacgactcaaacgaggaaaggcttacggtggatacctaggcacccagagacgaggaagggcgtagcaagcgacgaaatgcttcggggagttgaaaataagcatagatccggagattcccaaataggtcaacctttcgaactgctgctgaatccatgggcaggcaagagacaacctggcgaactgaaacatcttagtagccagaggaaaagaaagcaaaagcgattcccgtagtagcggcgagcgaaatgggagcagcctaaaccgtgaaaacggggttgtgggagagcaatacaagcgtcgtgctgctaggcgaagcggtggagtgctgcaccctagatggctagagtccagtagccgaaagcatcactagcttacgctctgacccgagtagcatggggcacgtggaatcccgtgtgaatcagcaaggaccaccttgcaaggctaaatactcctgggtgaccgatagcgaagtagtaccgtgagggaaaggtgaaaagaacccccatcggggagtgaaatagaacatgaaaccgtgagctcccaagcagtgggaggagaaagtgatctctgaccgcgtgcctgttgaagaatgagccggcgactcataggcagtggcttggttaagggaacccaccggagccgtagcgaaagcgagtcttcatagggcgattgtcactgcttatggacccgaacctgggtgatctatccatgaccaggatgaagcttgggtgaaactaagtggaggtccgaaccgactgatgttgaagaatcagcggatgagttgtggttaggggtgaaatgccactcgaacccagagctagctggttctccccgaaatgcgttgaggcgcagcagttgactggacatctaggggtaaagcactgtttcggtgcgggccgcgagagcggtaccaaatcgaggcaaactctgaatactagatatgacccaaaaataacaggggtcaaggtcagccagtgagacgatgggggataagcttcatcgtcgagagggaaacagcccggatcaccagctaaggcccctaaatgaccgctcagtgataaaggaggtaggggtgcagagacagccaggaggtttgcctagaagcagccacccttgaaagagtgcgtaatagctcactgatcgagcgctcttgcgccgaagatgaacggggctaagcgatctgccgaagctgtgggatgtcaaaatgcatcggtaggggagcgttccgccttagagggaagcaaccgcgaaagcgggggtcgacgaagcggaagcgagaatgtcggcttgagtaacgcaaacattggtgagaatccaatgccccgaaaacctaagggttcctccgcaaggttcgtccacggagggtgagtcagggcctaagatcaggccgaaaggcgtagtcgatggacaacaggtgaatattcctgtactaccccttgttggtcccgagggacggaggaggctaggttagccgaaagatggttatcggttcaaggacgcaaggtgaccttgctttttcagggtaagaaggggtagagaaaatgcctcgagccgatgtccgagtaccaggcgctacggcgctgaagtaacccatgccatactcccaggaaaagctcgaacgaccttcaacaaaagggtacctgtacccgaaaccgacacaggtgggtaggtagagaatacctaggggcgcgagacaactctctctaaggaactcggcaaaatagccccgtaacttcgggagaaggggtgcctcctcacaaagggggtcgcagtgaccaggcccgggcgactgtttaccaaaaacacaggtctccgcaaagtcgtaagaccatgtatgggggctgacgcctgcccagtgccggaaggtcaaggaagttggtgacctgatgacaggggagccggcgaccgaagccccggtgaacggcggccgtaactataacggtcctaaggtagcgaaattccttgtcgggtaagttccgacccgcacgaaaggcgtaacgatctgggcactgtctcggagagaggctcggtgaaatagacatgtctgtgaagatgcggactacctgcacctggacagaaagaccctatgaagctttactgttccctgggattggctttgggcctttcctgcgcagcttaggtggaaggcgaagaaggcccccttccgggggggcccgagccatcagtgagataccactctggaagagctagaattctaaccttgtgtcaggacctacgggccaagggacagtctcaggtagacagtttctatggggcgtaggcctcccaaaaggtaacggaggcgtgcaaaggtttcctcgggccggacggagattggccctcgagtgcaaaggcagaagggagcttgactgcaagactcacccgtcgagcagggacgaaagtcggccttagtgatccgacggtgccgagtggaagggccgtcgctcaacggataaaagttactctagggataacaggctgatcttccccaagagttcacatcgacgggaaggtttggcacctcgatgtcggctcttcgccacctggggctgtagtatgttccaagggttgggctgttcgcccattaaagcggtacgtgagctgggttcagaacgtcgtgagacagttcggtccatatccggtgcgggcgttagagcattgagaggacctttccctagtacgagaggaccgggaaggacgcacctctggtgtaccagttatcgtgcccacggtaaacgctgggtagccaagtgcggagcggataactgctgaaagcatctaagtagtaagcccaccccaagatgagtgctctcctattccgacttccccagagcctccggtagcacagccgagaagcgacgggttctctgcccctgcggggatggagcgacagaagtattgagaatccaagataaggtcacggcgagacgagccgtttatcattacgataggtgtcaagtggaagtgcagtgatgtatgcagctgaggcatcctaacagaccgagagatttgaaccttgttcctacatgacctgatcaattcgatcaggcactcgccatctattttcattgttcaactgtttgacaacatgaaaaaaccaaaagctctgctctccctctctatctatccaagggatggaagggcagaggcctttggtgtcccttccagtcaagaattggggcctcacaatcactagccaatatgcttttctctcatgcctttcttcgttcatggttcgatattctggtgtcctaggcgtagaggaaccacaccaatccatcccgaacttggtggttaaactctactgcggtgacgatactgtaggggaggtcctgcggaaaaatagctcgacgccagaatgataaaaagcttaacacctcttatttgactttgaaaatatttttcaaaaagataaaaatccaaaatgcaaaggtcgtcttattcaaaacctcaattatgcttctctcccacttcacacctcgaaacgcactgttcttgagaaggcgctttcacatcttcttaacccgaatgaggagaggaaaggttcctttttgagggtactcccgggaacagatccagtggagacggggtgggggatatagctcagttggtagagctccgctcttgcaaggcggatgtcagcggttcgagtccgcttatctccagcccgtgaacttagctgatactatgatagcaccgaatttttccaattcggcagttcgatctatgatttctcattcatggacgttgataagatccttccatttagcagcaccttaggatggcatagccttaacgttaatggcgaggttgggtcgttgcgattacgggttggatgtctaattgtccaggcggtaatgatagtatcttgtacctgaaccggtggctcactttttctaagtaatggggaagaggaccgaaacatgccactgaaagactctactgagacaaagggctgtcaagaacgtagaggaggtaggatgggcagttggtcagatctagtatggatcgtacatggacgatagttggagtcggcggctctcctaggattccctcatctgggatccctggggaagaggatcaagtttgcccttgcgaatagcttgatgcactatctcccttcaaccctttgagcgaaatgtggcaaaaggaaggaaaatccatggaccgaccccatcgtctccaccccgtaggaactacgagatcaccccaaggacgccttcggcatccaggggtcactgaccgaccatagaccctgttcaataagtggaacacattagctgtccgctctccggttgggcagtaagggtcggagaagggcaatcactcgttcttaaaaccagcattcttaagaccaaagagtcgggcggaaaaaaggggagagctccccgttcctggttctcctgtagctggattctccggaaccacaagaatccttagaatgggattccaactcagcaccttttgagattttgagaagagttgctctttggagagcacagtacgatgaaagttgtaagctgtgttcgggggggagttattgtctatcgttggcctctatggtagaatcagtcggggaggcctgagaggcggtggtttaccctgggcggcatggccaagcggtaaggcaggggactgcaaatcctttatccccagttcaaatctgggtgtcgcctatgactcgacaaattcttgccctgcataagcaaaggcaaaggacggggcttgtcgatacttgatttatagaatacatagttctataagttgttttctcaaaatctggctcttttgggttcggcccaaacagatataccaatagggatgaggtaaggcttacttattaattccagaactacgaaagtaagaggtcagaaatcttggtatccaaaggttcctaaaattccatttttgctcctaggattgaagaaaagattatacgaaagactatcaagacttcctaattatcttacactacctacactaagggtctactgactctgtctggtggaaaggactgaatttgtatccatacttacgatccgacaaacattcaatcaggatggttggtcggcccttatcttatagaataacagagtaaaaaaaaaaggattcctttgcaatttgcggtgtgtatcatatttttacttaatacttcccaatcagaaatcctataaagaatctgttacgagtggattcattgaattggttcatcaatagccttaagtcagaatcctattttgactctgcgccattgattctactatgattattgatcaataatggaataattccttcatagaaataggagatataattcaccgggactgacggggctcgaacccgcagcttccgccttgacagggcggtgctctgaccaattgaactacaatcccgcgaggtgtatggcacatattcttaatgtaatcataaattctagtcctagtcgtcgtattgtaagaaagacaggaatgatatactgatatcatatccacatataatatgggctatagcgagagtgacacggattactagtaaccaataattattttacggcaaaaaattgatcctttctctttatttctacggattaggcatttccgtttatggaagacaaattggttatattcatggagcggtgaattatacccccaggggaagtcgaatccccgctgcctccttgaaagagagatgtcctgaaccactagacgataggggcatatacgcccgaccatcatcatactatatagtatgagcagttttttggaattgtcaatatagtctaatggtatgaaatttattgttcctgaatgaactcctatgcatatacgtatatattattgcagtagactcataataaaaacggctaattcatgaattgaataaaacggtcgggatagctcagttggtagagcagaggactgaaaatcctcgtgtcaccagttcaaatctggttcctgacaagaaaaaatctattggatagatattcaaattaatagagaaggatcgggatacatattcgttaatagtctagagtatgatacatatttattcatctaggtatatagatatatacatccatttttataggtgggtaaaggtaaaaaagagatatatgtatggtaaagaacaaagtgagattacgttccctttcttcatttttgcttacgttactttccggtccatctaagtgatgtgcgcggtacaaagttcatggtgcaaaactcttttgattcatcctattttttctactcatacgaaagaaattaatatgatattttccaaattgaaattggggaatctcaatgaagtccttttttgcttaagctctccataataaaaatttgagtccagtagttattctgtttcatctagaactctggatataagtaaacatgagtttcttatcatttgcggagacaggatttgaacccgtgacctcaaggttatgagccttgtgagctaccaaactgctctactccgcgctaaagaactaggaactaatggacgaagaaagattggatacgcccctctaccatatctatacaaatagaatagtccatttatacagaatggtaaagaggcccttctatgatcatagatcatagagatctatacaaatatgaaacgatatttttatccgcggatatggtcgaatggtaaaatttctctttgccaaggagaagatgcgggttcgattcccgctatccgcccataattaagtaatgtactatgataaaaaaattaattgactactatactatactatatagttagtatagtagttctatcttacccctttctttgcctaacatccaaaaacaaaagcgggtatagtttagtggtaaaaccctagccttccaagctaacgatgcgggttcgattcccgctacccgctccatattctttattatacatgcgtcatcaatttggatatgcatcctttttcccgaaaagatatattttctgtataatcttatttgagcaagagttaagaatacgaaagaagaaaaatgaaaagtgtgattcgttctattaacaacttaaatagttaaggggtccatcggtttcaaactccgatcaaaaactttatttcttaaaagggtttaatccttttcttctcaatagcatatttgaggaaaaatatacgttctctcgatttgtatccaaaggccaattagaaattgcatcaaaaagttggattatggagtcgcgaagcataatttttgaattggattaactattccataagtataggtaaaggatctatggatgaagatacaaaagtatatttccaatcgtaactggatcttccatttttgtgttgtaaaaggaaatttaagccaaatagctaaaaaagatagttttggtttactagaaccatcagcatattgtttcagctcggtggaaaccaaattcttttcctgaggatctaggagtgaaaatagggaacgaagtaactagactagatagatttggtataatctctctcctctagagggatcatctagaaagcggtttagatgcattcatacagaaaagctgacatagatattatggatctcattttttctctggaaatacatggaccttccataaaggagccgaatgaaaccaaaatttcatgttcggttttgaattagagacgttaaaaatgatcaaccaacgtcgactataacgcggatgtagccaagtggatcaaggcagtggattgtgaatccaccatgcgcgggttcaattcccgtcgttcgccatcacattatttcaaattccaaaaatttgattttccatattcgggctattagctcagtggtagagcgcgcccctgataagggcgaggtctctggttcaagtccaggatggcccgctgcgccagggaaaagaatagaagaagcatctgactctttcatgcatactccacttggctcggtcgttgtgcctgggctgtgagggctctcagccacatggatagttcaatgtgctcatcagcgcctgacccggagatgtggatcatccaaggcacattagctgggttgcccgggactcgaacccggaactagtcggttaaaagccgagtactctaccgttgagttagcaacccggataaataggatatgtagatatgatcgaaatataaaaatcaattgagttgcactgcacgaccctatcaaaacattgaactagcaacacatcgaaaagaaggattttatgatcaattagaaacacaaaataccaaaattatgacagaccacccattttttatcaaattcttttttgattttccctaagaaaatacatcttgtatgagagtaaatgcagcaaggcaaacctttgagtgaaggaaacaaaaaaaccaatatgggtggggataaacagccctatttatctacgatcgaattatatttgttcgatacaccattgtcaatataaatgttgagaaagtaaatcaagtaaataaaatcttgtgttggattggcacaactaagaaattggaatgaaaaattaaggtaaataaaaaatcccggtttattcaatcaataaaagtaagcaagcttaaccccttgtttgttgttaaattcaattcccccaccaaaatatgaataaagcaagaaaaaggaaaatggtgtgtaaatagaaataattacacaaggatagatactagttacccttccctactttattttatatggagtagataatttttccttgttgcaatagaggaaaaagatccctccccaaaccgtgcttgcatttttcattgcacacgactttccctatgtatacatatcaaatttacatcccgagaagaaagtctacgaagactactcagttgcttcaaccactacatgagcatttcagaatataaatttatgaatatatttagagatctattctctatatgggtctcatgaccaattatgccttgaagaggactcgaacctccacgctctttagcacgagattttgagtctcgcgtgtctaccatttcaccatcaaggcatcttgaaagtgaatcgtattccatgaatatgatatctatctaatgtgatatatggaatatatgacaaaggtggagtgttggagtatttctatcgatcggtcatataggcctgagtcagacatcaaattgcttcgatttgaattatccggaggataccttatatatatcaaaaagatgtacaatcaaacctatttctcgattcaatagaagcccaaagaagtgaatatggtacccaaataacgataggatagatatgtcaaaagcgggtctgattacgcctattcctaatcctaaatagaatgtaaggacgtagggatccatatgtaaacatagtctctatttacatacgcttgaatgaccccttctcataataagaatgtacataaccctattccggtctggtccggtatggaatgaacttataatctgatgatcgagtcgattccatgattataagttcataaccccattttgggcggaacagatctactaattcttttattccagttagtaagagggatcttgaactaagaaatagacctagaagggggatatggcgaaattggtagacgctacggacttaaaatccgtcgactttataaatcgtgagggttcaagtccctctatccccaataaaaagccattttactttctaactatttgttttttcatcaatgaaaacaattcactatctttctcattcattctactctttcacaaatggatccaacagaaatctttggatcttatacaaatgaagatatataggcaatctctattattaaataattcacaatccatatatttactaggtcaaattttttgttttagtccctaaagtactctactaggatgatgcacaaaaaatggattggattgagccttggtatggaaacctgctaagtggtaacttccaaattcagagaaaccctggaattaaaaatgggcaatcctgagccaaatcttttgggaaaacaagggtataaaactagaataaaaaaggataggtgcagagactcaatggaagccgttctaacgaatagagttgactacgttgcgtcggtagctggagtccctctatcgaaattagagaaaggccatatatacctgtactatacatactgacatatcaaacgattaatcacgacacgtataatatatttatattatttatgaaatcatgaaattcggaaaaattcagagttattgtgaatccattccaatcgaacaaaaatcgactattcagtaataaaatcattcattccagagtttgatagatcttttgaaaaattgattaatcggacgagaataaagagagagtcccattctacatgtcaataccgacaacaatgaaatttttagtaagagggccgccgtggtgaaatcggtagacacgctgctcttaggaagcagtgctaaagcatctcggttcgagtccgagtggcggcatcttctaaaagagatacaatagatcctaaaatgtattcaattcgcgatttccaattctgtaatgggacccctttctccccaagtaggattcgaacctacgaccagtcagttaacagccaaccgctctaccactgagctactgaggaacaacgggattcgacctcatagagttcaactcccgttctcaacccatgaacaatatgagtccgaagcttccttcgtaactcccggaacttcttcgtagtggctctgttccatgcctcatttcatagggaaccccaaagtggctctatttcattatattccatctatatcccaattacattcatttaatatccctttggtcttattgacataagagatgtcatttatagtctatctctttctatatatggaaagttaagaaatcatcatataataatcgagaaattgcaatagaaaagaaaaagggaggtttgtgtagggatgacaggatttgaacccgtgacattttgtacccaaaacaaacgcgctaccaagctgcgctacatcccttttaaaaattgttgtacagtgtcattgtacaaaatacctgtcttgttttccacatctttattttctcctctatctatatagaactttcttgtcatttcttgtttttggtttcatataataaattatatacatctgaaacccacctaacatataaaaaagaatgaatatttatccgtaatgctcaggtcatctttttcttttttagtgacaggaaaatctcatctattggttcattgtacatatccttgttaggaaatccgcgtaaaaaaaaaatgatcttctgggacggaaggattcgaacctccgaataacgggaccaaaacccgctgccttaccgcttggccacgccccattttgatttctattcgatattaatcaatactaatattggtattggttattcgtcaatcccaacccaaatacacaaaaacatatgggtattttgttgctaggattcaagacatgtagatatagaatcaaaaaaattcattgatcattacataaaattcaattaagatattgtatgaaagttgaattccttatattctcattttagaatgataatgggaggttatttgggaaagtccgaaccgaaaaaaagaaggatttcttgatctgcctttttcatttttccttataaaaataactcaaaattatctaatccacaagaacgaaatgcttgttgggcctgtagctcagaggattagagcacgtggctacgaaccacggtgtcgggggttcgaatccctcctcgcccacagccttcccaaaagggaaggccccttactttacctctgggggtaggaaaatcatgatcgggatagcggacgcaaagctattgaacttgggtcttttgtcgaaatggaatggtcttactttttatttatcgtgaatgattcgatcattacatatagtaaccgaggccggaatcagcatatttgtactccccgtaactcttcctcagccaggcttgggcagaatagcagagcaagcacaagtattattagtagcatagcaaaaatgcgttcctcgtcattaatatgtttgctcgcgtgtggcctatcgggagaatcgatgactgcatcaaagatgcactgctagtacatcatctgagaattcttaattggctatttacaattgacggcgattctcaaatatcgtagaacagaatgtgatacgatgagatagaatgcaatagaaacaaagatagggaacgggttacctactcctaacggtcaaagcgagccctttaattcaattcttcattctttaattaagaattaatcaaatgcgtccattgtctaatggataggacagaggtcttctaaacctttggtataggttcaaatcctattggacgcaatttttttccatctattttgttaaatgtctatactaagaaaccctttttgaatgattcaaatcagaaatttttctcaatgattactctcatgctaagaataagtatgacggaaagagagggattcgaaccctcggtacaaataatttgtacaacggattagcaatccgccgctttagtccactcagccatctctcctaattccaaatttaaaatttgttatgtgatgtaacacgtgaaataaagattgataaaaatccaccttcttcatttattttttcattatcttatttaacaaagcaattgatcaggaattcaatatagataatgactcaaaacggatctcctcaatagaaattctcccggcctgttaagtactggccgggacatttcttctttttctcagtttattacttaatttcttactgttgtcaagtaaggaataaaaaaagacatatgataactaaacaatattaaattacatttaacaattggaaatatttctattctacaagagacaaaatttgaacagttgagattcaattgacccgaattcataagatctgacaccggcagttgaaaagaaggtgaagggggtcgtgtatacagaatttatcatttttatagtctagcttcaatcaccccttcaggcgggaactattagtttagtaatgacttcccagcaaaagcttaacttttatttaaataagctttacatcgcttagccggcgagactaaatacgtgtcaacgctagaattttcatgattccactgctatcttgattttgtctcaccctcttttgttcgacaaatggtccattcatatacaataatgaatatgtaggagagatggccgagtggttcaaggcgtagcattggaactgctatgtaggcttttgtttaccgagggttcgaatccctctctttccgtttctgttaattcaccaatgttatcgaccacaatgtatcaaatcaaataacaatggaaaccattattcccgcaataagacccttatttgatagaaattctctattcctaagcggatggattaaggcccttagatctatttagttcggcgaaaagggggcaaaattctatgaacctttcttaattaggagagagagggattcgaacccccgatagttctttgtttagaactataccggttttcaagaccggagctatcaaccactcggccatctctcccaggggtaatctctattttattcctacgaatagaacatgaccatatgacacactaactatctgtagaaacatcccagatgcaaatccatatttcgatgtatctatgtatactgtatacatagatacatgatctagtatgtctgcttgtaaaataaagactaaactccccccgagttcatgtccgaataaaataaagtggtaataagttctaaagaatcaattgattcatgattaaatccctccatgatggattttattacaattttgactaagtgagggatcaaatgtatagttcatttgttggtagctcggaggattagaaacgcccttttaactcagtggtagagtaacgccatggtaaggcgtaagtcatcggttcaaatccgataaagggcttttttccactaaactcaagttttagccttcgtttttcagccattataacgaattcagattattctgactttaagttaggaagttgaacatttattgattagcaaataattgcacgtattaggagtagtccacctgtagtgacatagtagtcctcctcatgtctcattattcaaaaattgtcctgggacataacagaaatattctataatactctatatatacaattcctattattaatcgacaaaccaaggtgttcttatttctccaatgttcttattctaaataaagaaaaagtaagtggacctgacccattgaatgatgactatatcagctattctgatatttaaattcgatatagattaaattgtataagcggatttttttatttccttagaccgcgcaaagcaagaatttctcaatatttatggtttaatcttcttgttactggatgctccataggaataaatcgctattttttcttctacaaagtttatttcaaaatttttcaattccagagaacgaatgcgagaaaaggactttcattttcagtctaccattatttaatattcaattttgtggcaggaaaaagtaggaaattttattttggttaaaagatatactctgaaatatgagtcataggacaattcaggattcaaatggttatcaaatggttatatagtataagggctaatcaaatcgagctcatggatttacctaggttagtttgtggcccaatagaaaaagagtatattcgaaacccattgtaaaggggcattgaacgagaaattgtctatagataatcgaactatcgtatgccttggaaatgatatgaggtgttcggaaatggttgaagtaattgaataggaggatcactagccggctatcggagtcgaaccgatgaccatcgcattacaaatgcgatgctctaacctctgagctaagcgggctcgcataagaaaaaattgcgtaacaaatagaaatattgtataggaattccggaaaatgtcggtcttagatatgaactaaactaattaatagagttctgtagctaaagttctaagctaagttcttttataaatgaatgaatatcgaacgttacagtattacaaatcacactgtaaaaatgaaagggagagataaaatagatatgggatatatctattcatcttgaattgaaaatacatcaatgatagaattatttctgattgaaataaacaaggtttatccaatagaaatgaactgatataggatggaaaaaaagaaataaatgaaagagatggatgaaattataaatgtatcttgcaaagaaaagggaaaagggatataactcagcggtagagtgtcaccttgacgtggtggaagtcatcagttcgagcctgattatccctaaacccaatgtgagtttttctattttgacttgttcccccgccgtgatcgaacgagaatggataagaggcttgtgggattgacgtgatagggtagggatggctatattgctgggagcgaactccaggctaatatgaagcgcatggatacaaacaagttatgccttggaatgaaagacaattccgatttgtctacgaacaaggaagctataagtaatgcaactatgaatagggctatacggactcgaaccgtagaccttctcggtgtaaacgaggtgctctacctaactgagctatagcccttgtcatagatatcttaacatatagataatttcttgtcaagatggatattccctaatctcacatgataactctttgatccgcttactgttaacagattggtattgcttagaaataatattctatctataatccccgaggtgatgggtcttcttttgcggtgataaattgtaaaacagatcaaactaattattatcgaaatgattcgaactgtttcaaagacccaacatgcattttgttgcattgggctctttcatcaactgatgtaaagatcagttagtccaccatattttttctttacaagaagataatgagctggctccatgtgctctgattcattatttgtattcagatctagtagcaataccaaagtgtttcaaagaagggttaccttgacttaggtctgccttcggcttagatccacctaagttaaatggagtctctatcgttccgctgcaagagtcgaatatgagacttcatacaccttaaagttcataggatgaaaggaggttttttgaagcccttatactcattatgcctagcattgaatgggctgagtatttaccttatcaactatcaaatcaatgatgggttctatttgatttggcacctaaattcgcaccaaaacggaccaaaccaaatatttgtcaggctattgttctctcgaatctatggagtaagacttcaactatgttcattgcataataagctcccttgaaaagcattggcgcaccacgctctgtaggatttgaacctacgacatcgggttttggagacccgcgttctaccgaactgaactaagagcgctttcaaagaatttttttccataacacatctcacataaatatagtatccaaaaaagattatgccccgtcgatcccaattaatctttcgttactgcccataggagaagtaataggtgggccgagctggatttgaaccagcgtagacatattgccaacgaatttacagtccgtccccattaaccactcgggcatcgacccaggaagaattcattctagacttatctaatctatgatcaacttcctttcatagtaccccgtggaaacacttgtttattccatatgtatggatgatatgaactgcctaaacaagaacggcgaacaaccagagtactcactacatcaaacaatttccattaatgaaaccatgtaaatccatcggataatcaaagcatgtctgatgaaatggttgttgttatctgcttcaataacgaatcattggtttaactgaataacaaagaaaatagatagacccttctctttctcttcgtctcaggtcgatggatcttctcaacgggaagatctcccatatgcataatacacattccagttgaccgagcctcattctaattgttttgttccgaagcaaagatatccacggaggccggtttgtcctattcagatattcacgaccaggaggtactggattctctttcggataggccctgaaaggagaagaaaggctggaatgccaacagacgtctgtctattctctaattcacccgacccgatagtacccattttggtaacgtccagtgccaaagtcactgaatgggtaagtcgccaatccctaaaacggactactcattcattagatagagaagatcgccaagatttcgtgatccgctgccgaacctattccaattccaacagctcagactcggatcgtggggatcaccggaatacttcgtatcaacagataagatactcgatcaatattgattagatccgaaatccgttattgaattgctcattcaatgagcattctcaatattatctaaatcgtggatacatatgtatccttgacatactgaaacgactgccattattggtatcaaaccaatagcgattcatacaagctaaatcttctaatcggtaattgggccaaagaacaaatttgcatttaatgaatttatttgtatccgtattaagatgcttgtcctcatccaaaaattttacagagtttcttatgttattttcattgcaaaataatggatttctatccgtaacattccaattatgggaattgaaacaaattaacattctcaattctctgcgacgtctaggagatagaatattttcgggaacaaggaaatcataataatttgtgtccccattcacaagcatattcccatatcgtacaatgggtttatccaaattcctcttatcaatatatcttttttttatgcattttctattagtttggtgtttattgttctcgaccaatgaaatacttatagtttgatatataataaattttccatccctttttatagatagatgaattggttcgataattaatattcccttttttatcaattctgtaagagctagatctttctgaattagcattacatccagatgcatctctcctctttgaatcgaggatatagcaatttcttttggatttatcagtctaagtaagagacaatataccttgatattattgatcattctttggttcaaggagtcatcccatcttaattgaaaaaggaaatattttttcaggaagaaatctagttctgcttccttgtttttcttggattgttttttcttcttaccttttttaatggtaatgtctgatctcgcataattctcttcaatatctttttttttttttcgtagatctgatacaagatttacttggtcctgttgctcatttttttgttggttggaattttctaaaagaagtaatttgattggtataatccatggtttaatcttatatgcatcaaaaagtaagacaaattctgggaagaaccaagattctagatttgatatggtatgataaactctttcttgattcattcccatccaatttaaaaaaatacttttttgattggatgggttgatttcttgatgaatcataagagaaaaaatatctttttttttcaatttgttgttgatttttttttcagtcttagtatttttataaattttggtaccgatatgtgtattggtccaggtctcaatattgatattttttctaagacaaaaggggagaatttgacaatcaaaatattttctatctagattttgatgcgtatcaataatatatccttcttctagataatcactaatagctgtacttaccaatacataaaatgattcggattttggtttattgaaattatatggaattttgtgaaccccatttacttgtaatcttgacccataaatataagaatcctccttatccccatagtttatatatttatgtgataaaagatcatatctgtagtgttttttccgatgaatccgctttaattgagtccttattttgaatcctataacgttgattgattctatttcgccatttttgtggtactaaccgcgaccatttggtttgagataaattgtattgataatgcccctttaaccagtcaatcattccagacttatgaattttcttatgtcttgaattgtaatcaaatattcctcgtgtcatacaataattcttgattttatccttaataaaagggtaagtcccctgatattgaagtagagatttcaattgatacttgttaagtaattgggtttgtgataatttgtaaaatacatatgcttgggacaaggaggataagtcataatacatttttgtactattactattcgaaaaggacttttttatagtgtaaaaaaagttcattgtattttgatctctttcatcaattccttcctgatttgtttgatcgttgtaaacggatttattgattattttttttgttgattcaaagaaaagttggaaatagatcctgtgaatggtaatcatacatagcaagatatctatatatatattttcaatcagagatttcataaaataatgtgatttacgtaggaatatctgccaaatatgtttcttcgatcttttatcatcacaagttagaattattcttttcttgtcttttgtgattcgttctatttgattcctgattgtgattgttctatcagaaagatctttcatctttttttctatgagtgaataatttgtccaattcatggattggatttgaatggttgatttgtgaataatcttattatttatttcggaatcttttccattttcagccgtttcatatactttcaccctgctcaattcaaataagaatattgggtttactttttgaatttccttcattcttgttttttcttttgaaacttttagaagtagaaataaatcctttttaacttttctaacttttttttggagttccttataaatgggttcaaaaaaggaaggtcgttttcggggattcccaaaagggaattccgcttccattccccaaactgttaaaaaacaaaaatttaatttgatccctaggatgagatcgtattttagatcttcgccaaggtttcaaacagaaaggaaatagaatctttatctgaataccgtctgttaaccaatctttgggaaattctgtttctgataattgaacaccattataagtgcatttaacatgcatttctctattccactccttcaaatcctcataccattcggggaattggaataataacatacggccaatatttttagcaattatcaatgaaggcaatacaatgtgttttctaagaaaagattgggttactaacatcaaacctcttattgcttgaacaaatataatgctatcccaagtttctgatattactatacgttcattttcctcttttttttcttcgttttttttctctttttcccttgtctcttcctcctcaaaatcaaaatgtgaaatttctggttctctccccaacgaattcctaaaaatgagattcatcatttcagagatataaaaagaagaaaaaaaaaatgttttgtctattcgatccaaaaaaagcggagaatgcacatttgcttgaaacatttcccaaataaccgttttacgtctttgagcacgcatggatcctttgattatatcccgacgaaaatccgattgttgcgagtaacgtatcaaagccacctcttctgcttgatcactattattagtattatttgtagttataatagggttctgattgttatcagtataaattactacgcgtttagcttttcttgaacgaatttcatgatcttccttagattcttcctcctgttcttccaaatcatcagttaatttgtatgaccaccggggaacccttttacggatttcttctattccaatagatttatttctaattattgtttgatcgtttggatcagttgtaattacatcgaatacccattttaaagcttttgtttgattttctaaatcaattcttgtttgctctctcaatgcaggctcaaaaggaaattctttgattgaggttaaggaattaccaatataattcagtaatgattccctatcaggcggattcttttgatgttccaattttctggaataattagaattattaggaagtagcccataaatcttatttatccaattgatttctatggaatcctccgtatctgtagaagtgattaaatcattcatgatcatatgtgaatagaatttttttattgttccacgatatggtcccctcaaaaaggggtcatacgttttgggcaagtatttttgttcgttttcatcattgcaaaatctggtccttttttcgagcatatctagagcaagaaatcccttttctttttctagagcttttgttcgacttattaattcattgttcaagttgtactttttttgctcattggtataaacccaataatgatactgatccacatgggataatttttctgtcgtgtacaaagacatttttcgttctatcatttccgaaaaagtcgataaactaggtggatatgtaaaagatattatttgttttccatcacttggacatgtataaaaaaaatattgtgccatttcatttcgtacagcattttcaaatcgattattttttatataccgacatgggcgattccatcgtttataatcgaaATGATTTTAAAATCTTTTATACTAGGTAATCTATTATCCTTATGCATGAAGATAATAAATTCGGTCGTTGCGGTCGGACTCTATTATGGATTTCTGACCACATTCTCCATAGGGCCCTCTTATCTCTTCCTTCTCCGAGCTCGGGTTATGGAAGAAGGAACCGAGAAGGAGGTATCAGCAACAACTGGTTTTATTACGGGACAGCTatcaagaattctcactatttcttagattcatggatcaaattcgattcagtgggatctttcactcacatttttttccaccaagaacgttttatgaaactctttgaccccctaatttggagtatcctactttcacgtgattcacagggttcaacaagcaatcgatatttcacgatcaaaggtgtagtactgcttgtagtagcggtccttatatctcgtattaacaatcgaaagatggtcgaaagaaaaaatctctatttgatggggcttcctcctatacctatgaattccattggacccagaaatgagacattggaagaatctttttggtcttccaatatcaataggttgattgtttcgctcctgtatcttccaaaagggaaaaagatttctgagagttgtttcatggatccgcaagagattacttgggttctcccaataaataaaaagtgtatcatgcggagttcgcgatggtggaggaaccggatcggaaaaaagagggattttagttgtaagatatctaatgaaaccgtagctggaattgagatctcattcaaagagaaagatagcaaatatctggagtttctttttttatcctatatggatgatccgatccgcaaggaccatgattgggaattgtttgatcgtctttctccgaggaagaagcgaaacataatcaacttgaattcgggacagctattcgaaatcttagggaaagacttgatttgttatctcatgtctgcttttcgtgaaaaaagaccaattgaagggaagggtttcttcaaacagcaaggagctgaggcaactattcaatcaaatgatattgagcatgtttcccatctcttctcgagaaacaagtggggtatttctttgcaaaattgtgctcaatttcatatgtggcaattccgccaagatctcttcgttagttgggggaagaatcagcacgaattggatttgaacgtatcgagagagaatttgatttggttagacaatgtgtggttgggaaggttttttagcaaggtacggaatgtattgtcaaatattcaatatgattccattagaaatgaggattcagaatatcacacattgatcgatcaaacagagattcagcaactaaaagaaagatcgattctttgggatccttcttttcttcaaacggaacgaacagagatagaatcagatcgattcccgaaatgcctttttggatcttcctcaatgtcccggctattcacggaacgtgagaagcagatgaataatcatctgcttccggaagaaatcgaagaatttcttgggaatcctacaagtcgttcttttttctctgacagatggtcagaacttcatctgggttcgaatcctactgagaggtccactagagatcagagattttggaagaaaaaacaagatgtttcttttgtcccttccaggcgatcggaaaatgttgatatattcaagataattacgtatttacaaaaaaccgtctcaattcatcctatttcatcagatccgggatgtgatatggttccgaaggatgaatcggatatggacagttccaataagatttcattcttgaacaagaatccattttttgatttatttcatctattccatgaccggaacaaagggggatacacgttacaccacgattttgaatcagaagagagatttcaagaaatggcagatctattcactctatcaataaccgagccggatctggtgtatcataggggatttgccttttctattgattcctacgggttggatcaaaaaaaattcttgaatgaggtattcaactccagagatgaatcgaaaaataaatctttattggttctacctcctcttttttatgaagagaatgaatctttttatcgaaggatcagaaaaaaatcggtccggatccactgcgggaatgatttggaagatccaaaactaaaaacagcggtatttgctagcaacaacataatggaggcagtcaatcaatatagattgatccgaaatctgattcaaatccaatatagcacctacgggtacataagaaatgtatcgaatcgattctttttaatgaatagatccgatcgcaacttcgaatatggaattcaaagggatcaaataggaaatgatactctgaatcatataactgtaatgaaatatacgatcaaccaacatttatcgaatttgaaaaagagtcagaagaaatggtttgatcctcttatttctcgaaccgagagatccatgaatcgggatcctgatgcatatagatacaaatggtccaatgggagcaagaatttccaggaacatttggaagatttcgtttctgaacagaagaagcgttttcaagtagtgttcgatcgattccgtattaatcaatattcgattgattggtccgaggctatcgacaaacaagatttgtctaagtcactttgtttctttttgtccaagtcacttctctttttgtccaagtcacttccctttttgtccaagtcacttccccttttctttgtgagtatcgggaatatccccattcataggtccgagatccacatctatgaattgaagggtccgaatgatcaactctgcaatcagttgttagaatcaataggtgttcaaatcgttcatttgaataaattgaaacccttcttattggatgatcatgatacttcccaaagaccgaaattcttgatcaacggaggaacaatattaccatttttgttcaaaaggataccaaagtggatgattgactcattccatactagaaataatcgcaggaaatcctttgataacacggattcctatttctcaatgatatcccatgatcgagacaattggctgaatcccgtgaaaccatttcatagaagttcattgatatcttctttttataaagcaaatcgacttcgattcttgaatgatccacggttctattgtaacaaaagattccctttttatgtggaaaagacccgtatcaataattatgatcttacatatggacaattcctccgcaacaaaatattttctttgtgcgtcggtaaaaaaaaacatatttttttggagagagagactatttcaccaatcgagtcacaggtatctgacatattcatacctaacgattttccacaaagtggtgacgaaacgtataacttgtacaaatctttccattttccaattcgatccgatccattcgttcgtagagctatttactcgatcgcagacatttctgcaacacctctaacagaggaacaaatagtcaatttggaaagaacttattgtcagcctctttcagatatgaatctatctgattcagaagggaagaacttgcatcagtatctcagtttcaattcaaacatgggtttgattcacactccatgttctgagaaatatttaccatccggaaagaggaaaaaacggagtctttgtctaaagaaatgcgttgagaaagggcagatgtatagaacctttcaacgagatagtgctttttcaaatctctcaaaatggaatctgttccaaacatatatgccatggttccttacttcgacagggtgcaaatatctaaatttcacccttttagatactttttcagacccattgccgatactaagtcaaaaatttgtatccatttttcatgatattatgcatggatcagatatatcatggccaattcctcagaggattcttccacaacggactctgataagtgagattttgagtaagtgtttacagaatcttcttctgtccgaagaaatgattcatcgaaataatgagtcaccccttccattgatatggacacatctgagatcaacaaatgctcgggagttcctctattcaatccttttccttcttcttgttgctggatatctcgttcgtatacatcttctcttcgtttcccgagcctctagtgagttacagacagagttagaaaagatcaaatctttgatgattccatcatacatgattgagttgcgaaaacttctggataggtatcctacatctgaactgaattctttctggttaaagaatctctttctagttgctctggaacaattaggagattctctggaagaaatacggggttctgcttctggtggcaacatgctattgggtggtggtcccgcttatggggtcaaatcaatacgttctaagaagaaatatttgaatatcaatctcatcgatctcataagtattataccaaatcccatcaatcgaatcactttttcgagaaatacgagacatctaagtcgtacaagtaaagagatctattcattgataagaaaaagaaaaaacgtgaacggtgattggattgatgataaaatagaatcctgggtcgcgaacagtgattcgattgatgatgaagaaagagaattcttggttcagttctccaccttaacgacagaaaaaaggattgatcaaattctattgagtctgagtcataatgatcatttatcaaagaatgactctggttatcaaatgattgaacaaccgggatcaatttacttacgatacttagttgacattcataaaaagtatctaatgaattatgagttcaatagatcctgtttagcagaaagacggatattccttgctcattatcagacaatcacttattcacaaacctcgtgtggggctaatagttttcatttcccatctcatggaaaacccttttcgctccgcttagccctatccccttctaggggtattttagtgataggttctataggaactggacgatcctatttggtcaaatacctagcgacaaactcctatgttcctttcattacggtatttccgaacaagttcctggatgacaagcctaaaggttatcttattgatgatatcgatgatgatagtgacgatatcgatattgatcttgatacggagctgctaactatgacgaatgtgctaactatgtatatgacgccgaaaatagaccgatttgatatcacccttcaattcgaattagcaaaagcaatgtcttcttgcataatatggattccaaacattcatgatctgtatgtgaatgagtcgaattacttatccctcggtctattagagaactatctctccagggattgtgaaagatgttccactagaaatattcttgttattgcttcgactcatattccccaaaaagtggatcccgctctaatagctccgaataaattaaatacgtgcattaagatacgaaggcttcttattccacaacaacgaaagcactttttcattctttcatatactaggggatttcacttggaaaagaaaatgttccatactaacggattcgggtccataaccatgggttccaatgcacgagatcttgtagcacttatcaatgaggccctatcaattagtattacacagaagaaatcaattatagaaactaatacaattagatcggctcttcatagacaaacttgggatttgcgatcccaggtaagatcggttcaggatcatgggatccttttctatcagataggaagggctgttgcacaaaatgtacttctaagtaattgccccatggatcctatatctatctatatgaagaagaaatcatgtaaggaagggtattcttatttgtacaaatggtacttcgaacttggaacgagcatgaagaaattaacgatacttctttatcttttgagttgttctgccggatcggtcgctcaagatctttggtcttcacccggacccgatgaaaaaaattggatcacttcttatggattcgttgagaatgattctgatctagttcatggcctattagaagtagaaggcgctccggtgggatcctcacggacagagaaagattgcagtcagtttgataataatcgagtgacattacttcttcggtccgaaccaaggaatcagttagatatgatgcaaaatggatcttgttctatcgttgatcagagatttctatatgaaaaatacgaatcggagtttgaagaaggggaaggagccgtcgacccgcaacagatagaggaggatttattcaatcacatagtttgggctcctagaatatggcgcctttgtggcaatctatttgattgtatcgaaaggtccactgaattgggatttccctattgggccgggtcatttcggggcaagcggatcatttatcataaagaggatgagcttcaagagaatgattcggagttcttgcagagtggaaccatgcagtaccagacacgagatagatcttccaaagaacaaggcttttttcgaataagccaattcatttgggaccctgcggatccattctttttcctattcaaggatcagccctttgtctctgtgttttcacgtcgagaattctttgcagatgaagagatgtcaaaggggcttattacttcccaaacaaatcctcctacatctatatataaacgctggttcatcaagaatacgcaagaaaagcacttcgaattgttgattcatcgccagagatggcttagaaccaatagttcattatctaatggatctttccgttctaatactctatccgagagttatcagtatttatcaaatctgtttctatctaagggaacgctattggatcaaatgacaaagacattgttgagaaagagatggcttttcccggatgaaatgaaacatttgattcatgtaacaggagaaagatttcccattccttagccgtaaagatatgtggccatgaaaaagggaagggattaagtggaacaggattggccgggtggtagagtttattcgaagcgcttcgtgattttcaaccaattatgtgcttcaatataattacctggagtaagcgctatagcttgtttccaatactcagcagcttgatcggaccaagcttccgcaatttcagaatcaccctgtagaatggcctgttctccccggtaatgacagatcacggccatattattaaaagcttgtggtaagaatgggtttcgttctagtgcccggaaataatattccaaagcctttgtatgctctccattgcttgtgtgtataaggcctatgttatagagtatataacttcgatcatagggatcaatttctggtcgcgtagcttcataataattctgtaaagcttccgcataatttccttcggattgagccaacataccatctctgtaataggtaaatgcctttttttctcctgaagttgtcggaattattcgtaataagatattggctacaattgaagaggtcttatcaataaaatttccatttatatgagatctaggcataattagcaatccattctagaattcttttcattacccggggaaaatgatcccacaaacaaaggaattatacagtacgaaataacataaaaaactttattctaaaatagatatgggctttccacttaaattgtccccttttgtttggaaagatatgagatattggaaattgatttcattcccatttttgtagtataccaatgagcggaactattactatttcatctaagttaaataaccaaggacttttttactacagattctaataactcgagaagttttgatttgattatgatccaaagagaaaaaagaatggaataatcattccatgaaaaaatagagtagagtaaccataccttctgtttgcataagtgtatacaccacgccatacaatcgaaatataaaaatccatgggacgatcataaatttggaatagatccacggggtagctgatgaatgagagaagtttttgttgagaaatattaaatgggaaaggaattcttcctatgtaactagtgatcggtcgtacctgtactgcagtaatatgaataactcgctattcactcagtttctggtcaataataagttatgtacggaataggtggttccttcccttagaaccgtacttgagagtttcctactcatacggctcaaaaatcgattcttttcttacctatgctaactgaattagatttctcataaacctatcccatttttcttgggttaaccagaagaagttaattacataagtttcaaaccctaattttgatcaataatcagaatcagtttgatcttttctcccaccttcagaagaatgaagcataggtatccccacaatatcgttagaattttctgaaaggtaactatctcggtttcatatatggaattcatatagaatctttgaaaaagactttttacataagaaaaaagaacttactatctttgggatctgatgctacaccgctgctcaataccttagtggatcgactctattacataagttgattcctaacttttgcccatatcatggcataagtaagcagttcttaactgtatcgactcaatagctcgctaattgatctttacggtgctttctctatcaatttgatcctttatccatagaatatagtatataggctgcactcattttttttcttcctattttggttctcgtgaagtctctttccttgctacagctgataaaaatcgttgctttggacgatgcatatgtagaaagcctatttttctagtatttactagttgatctttgcttttttccttatttctatagtggagatagtcgcacgttacggtcgttcattctattcaaaaatctccgttccagaaccgtacatgagattttcatctcatacggctcctcccttctgcgcatagtactaagggaataatccatagaataaaaatggaactattctcatctcattatgaactgaaaggaactagtatttttacaagaaatctctagccagccttcccgcaagaggtttttcttaacaccaatcatattagtgttagatataaatggtaactccaacaatttctttgttctcaacgccttctatttccaggaattagtcacttcaacgatctttgatggttatacgggtatccaaagtacaaacgagatggatgtttgttgtcccaaccattcttgttagtcccgataccgataaggaaagggggaatttataacaaagtttttgtgttgttgattcctaggtgtagtgctttttcccttatgccgtctattggtactaatgtagtgtaggattgacccgcaatacagagcccataggtgtaacctttcgctcaatactcaaatcaacaattgaaacatctgaggctgcatcaatcgaggatacacgatagaaggaattgttctatctccaaacttcaccttcaccgagcgtaggtttatttcaagaatttcgttctttctataccgaaccgcgtctctttctcgtaagactgaggtgaggaaaaaaacaagaaaaaagaatcaatgaattggcgatcagaacatatatggatagaacttataagggggtctcgaaaaacaagtaatttttgctgggcctgtatcctttttttaggttcactaggattcttagcggttggaacttccagttatcttggtaggaatctgatatccatatttccatctcagcaaattattttttttccacaaggaatcgtgatgtctttttacgggatcgcaggtctgttcgttagctcctatttgtggtgcactattttgtggaatgtaggtagtggttatgaccgattcgatagaaaagaaggaattgtgtgcatttttcgttggggatttcctggaataaatcgtcgcatcttccttcgcttccttatgagagatatccaatcaatcagaattgaggttaaagagggtctttatcctcgtcgtgtcctttatatggaaatcagaggtcaaggggccattcccttgactcgtactgatgagaattttactccacgagaaattgaacaaaaagctgccgaattggcctatttcttgggcgtaccaattgaagtattttgaattgaaagaataaattctcggcatgggggaaggaacttgctaattccctttttaatataattgaattttggaatgttcatttgaacaaaacatgttagattattctatttcctccttcctttgtcgtggcgactcccatagaataaaccaaaaaggagggcgatatggaataactataataaactataattaagaaaagaataaatttttgtataccaaaagtatttcatatgcgtatgggtcccaactcaattcttttctactagaaaatttctactagtctaataagtagggattcatcaaatatatcgatatttcgtaatacggactcatctttttaggcccaaaagatctttttccttggttgtggctaggcggtgaaacatttcgaaataattaactgggggttctaaatccgatttgttattttagtttcgagtattcatagaaaggaacaaatgaagatgaaattgcaatttgcccaattgagatatctaggaataatattgatttttcatttttatacgaaagggcaaactttctatctagatccaagaactaaactcaattaggttcaataccttgttataaactcgtgcttcagagaaatatcatatagagtcaacgaatgagttcattaacgattcaattcacagatggcgtactcctcctgtttgaatcggagtttgaaaccaaactcaggtgagatccaatgtagatctaactttctattcactcgtgggatccgggcggtccgggggggaccaccacggctcctctcttctcgagaatctatacatcccttatcagtgtatggacagctatctttcgagcacaggtttaggttcggcctcaatgggaaaatggagcacctaacaacgcatcttcacagaccaagaactacgagatcgcccctttcattctggggtgacggagggatcgtaccattcgagcctttttttcatgcttttcccggaggtctggagaaagcagcaatcaatagctcacttcttggtcttcgaccccctcagtcactacgagcgcccccccgatcagtgcaatgggatgtgtctatttatctatctcttgactcgaaatgggagcagaaaaaggatcttagagtgtctagggttgggccaggagggtctcttaacgccttcttttttcttcccatcggagttatttcacaaagacttgccacggtaagggagaagggggaacaagcacacttgaagagcgcagtacaacggagagttgtatgctgcgttcgggaaggatgaatcgctcccgaaaaggaatctattgattctctcccaattggttggatcgtaggtgcgatgatttacttcacttctcctcaggaggataggtggggcgat

8113173 atggaaaaatggtggttcaattcgatgttgtctaacgagagaacataggtatggtttaagtaaatcaatggaaagtcttgatgctattggccataccagtggaagtgatgaaccccttctaaatgatacggagaaaaattggagtgatagtttcagtaatgttgattatttatttggtatcagggatatttggagtttgatctctgatgacacttttttagttagggatagtaatggtgacagttattccgtatattttgatattgaaaatcatagttttgagattgacattatcattacatgtatgatactcaatctagttggactaatcaattaatagttccatttcaggtggtactgacaatttctgaaagttttagtataagaactcgtaataatggcagtgatttcaatataagaggaagatctaatgatttcgatataaataaaaaatacagacatttatgggttcaatgcgaaaattgttatggattaaattataaaaaacttcttaggtcaaaaatgaatatttgtgaacagtgtggatatcatttgaaaatgagtagttcagatagaatcgaactttcgattgattcgggcacttgggatcctatggatgaagatatggtttctatggaccccattcaatttcattcagaagaggaaccttataaagatcgtatcgattcttatcaaagaaagacaggtttaactgaagctgttcaaacaggcataggtcaactaaacggtattcccacagcaattggggttatggattttcagttcatgggaggtagtatgggatctgtagtaggtgagaaaatcactcgtttgattgagtatgctactaatcgatctctacctgtcattattgtgtgtgcttctggaggagcacgcatgcaagaaggaagtttgagcttgatgcaaatggctaaaatatcttctgcttcatatgattaccaatccactaaaaagttattctatgtaccagtccttacatctcctacaaccggtggagtaacagccagttttggtatgttgggagatatcattattgctgaacctaatgcgtacattgcatttgcgggtaaaagagtaattgaacaaacattgaataagacagtacccgatggttcacaagtggctgagtatttattccataaaggcttattcgacccaatcgtaccacgtaatcctttaaaaggtgttctaagtgagttatttcagctccatggtttctttcccttgaatcaaaattcaATATTATTTTTATATATACTTAGTAGTTAATATTACTTATAATAGATATACTTATCATAAGATATCTTTCTAATATACAAATATTAAATCGAGGCACCCATTCTttatggttgttcctgaagtagaaattgtttgatctgttcctgaatagcttccttcaaaacggcttctgcttgctcggtgaatgtcttggtagaagatataatttcttggaattgaggtttattcttttttaagtaagtacgtaactgaacaagaaatttctttacctgtccaatttctaacggatcaagatatccattcgctccggtataaatagtagctatctgctcttccaccgtgagagggtctgattgggattgtttaagcaactcacgtaatcgttgacctcttgccaattgattctgagtagctttatcgagatcagaagcaaattgtgcaaaggcttctaactctgcgaattgcgctagttccaattttgatttgccagctacctgtttcatggctttaatttgagctgcggatcctactctggaaacagaaatacccacattaatagcaggtcggattccagcattgaatagatcggcggataagaatatttgtccatctgtaatggaaattacattagtaggaatataagctgaaacgtctccagattgagtctcaactattggtaaagcagtcatacttccttcacctaaactagaacttgatttagcggctctttccaaaaggcgtgaatgcaaataaaaaacatctcctggataagcttcacgaccgggaggtcttcttaatagaagagacatttggcgataagcttgtgcctgtttggagagatcatcataaattattaaagtatgtcgttcacggtacataaaatattcagccaaagtcgctcccgtataaggagcgaggtattgtaatgtagcaggtgaatccgccgtttcagctacgacaatagtgtattccatggcccctcgctcctggaaagtagtcactacctgagccacagaagatgctttttgaccgatagctacataaacacatattacattttgccctttttgattgagaatcgtatctgtggctactgctgttttgccggtctgtctgtccccaataattaattctcgttgaccgcgtcctataggaatcatcgaatcaatagcaataagccctgtttgaaggggctcatatacagaacgtctcaaaataataccaggggcaggggattcaattaaccgagattcataagctgaaatttcccctctcccatcaataggtgtagccaaagcatttataacacgacccaaataagcctcactcacaggtatctgagcaattcttcctgttgcttttacagaacttccctcttgtatcatcaaaccatcacccattaatacaacgccaacattattggattccaagttcagagcaatgcctattgtaccctcttcaaattctactaattcacctgccattacttcatcaagaccatgaatacgagcaatgccgtcgcctacttgaagtacggtaccggtattcacaatctttacttctatattatattgttcaatacgttcacggataatattactaatttcgtcggctcgaagggttaccattagtgtttctttattctttttcgaaagcaaaggtaaaaataatgcctaaattgtaaactaaagtaaaagtgtcatttcttcaaattgctctccacttctaagttcatagctttcgcggtagcttcatcgatgttacccaccaaataaaaagcctgctcgggcagaccatctaattctccggaaaggatcagttgaaaccccctaattgtttctgcaagaccaacatattttcctggagaaccagtaaatacttctgccacgaagaagggttgtgataagaaacgctcaatttttcgtgctcttgctacagttaaacgatcctcctcggataattcatccaacccaagaatagctataatgtcctgaagttctttgtaacgttgtgaagtttgcttaactctttgcgcagtttcataatgttcctcaccaacgatccgaggttgtaacatagttgacgttgaatctaaaggatctactgctggataaatacccttggcagctaatcctcttgatagtacggtagtagcatctaaatgtgcaaatgtagtggcaggagcagggtctgtcaaatcgtccgcaggtacataaactgcttggatcgaagttatagatccctctttggtagaagtaattctttcttgcaaagaacccatttctgtactaagggtaggttgataacccactgcagaaggcattctccctaataatgcggatacttctgatcctgcttggacaaaacgaaagatattgtcgatgaatagaagcacatcttgttcattaacatcccggaaatattctgccatagttagggcagtcaaaccaactctcatacgagctcccggcggttcattcatttgaccatagactaaagctacttttgattctgcaagatttttttcattaattactccggattctttcatttccatgtaaagatcatttccttcacgagtacgttctcctactccgccaaatacggatacgcctccatgagctttggcaatgttgttgatcaattccatgatgagtactgttttacctactccagctcccccaaatagtccgatttttcctccacggcgataaggagctaaaagatctaccaccttaatacctgtttcaaagattgataatttcgtatctaactgtataaaggcgggcgcagatctatgaataggagatgttgtgcgagtatctacaggacctaaattatcaacaggctctccaagaacgttgaagattcgaccgagagtagctccgccgactggaacacttagaggagctcccgtgtcaatcacttccattcctctcatcagcccatctgtagcactcatagctacagctctaactcgattatttcctaataattgttgtacctcgcaagtcacattaatttgttgaccgactcgacccttaactaccaaagcgttataaatattaggcattttgcccgggggaaaaacgacatccagtactgggccaataatttgagcgatacgccctagttttttttcttcaagtgtggaaaccgcagggctagaagtagtaggattgattctcataattataataaagtgaaatatgtcgaaatctttttggaataataccaaatcaaaataaatgtccgatagcaagttgatcagttaattcaataagagataaatgggagatagctttttcatatagatttccgtctttatattataccctttcgtagatgaattatgcttattttcacatctaggatttacatatacaacatatattactgtcaagagggaattttctcagtatttagattcaaaataagaaagggttcaattataaacccgcaaaagattaggattgggttgcgctatatatatcaaagagtatacaataatgatgtatttggtgaatcaaatacatggtctaataacaaaccattttaacataacattttgttgataatattaattgaatattttttgaaagatttttgttaaaggtttcattcatgcctaatccatatcgagtagaccttgttgttgtgagaattcttaattcctagtaggaaataacattgacagcctctactcgtgtcctagctcgtctgagagctagattcgcttcaatcacttgtctcttaccctcagctctactcaagttagcttcagctattttaagagtttgttgagcttcttgcggatcaatgtcagtactcatctccgcatcatttcctaaaatggtgatctcattattccctattctagcaaaaccacccatcagagccaccgttaaccattggtcgttgaggcgtattctcaaaagacctatatctacagccgtggcaataggggcgtggtttggtaatacaccaatttggccactatttgtagataaaatgatttctttcacttctgaatcccaaataattcgattaggagtcagtacacaaagatttaaggtcatctaatcagttatttcttccatggccccaagaatgccaatattagcacggatcgtacggaaatgtaactcggtattcaaacaactattcagagttcctagagctccttgtaaggcttgttggaaaactcgttgtcggacttgatttatcgctctttgttgttcaaaatgaagggtttcatttttgaaattttctaatcgttccaaactatcacaagtagctttaatcaaattttctttttctcgttctatctcagagtatccattcattcgatactcatctgcttccagttcgactttctgtaagcgaacccgggctttttcgagctgctcaatggcccttctacgtaattcttccgaatttcgaatagtactcaagatcctctgttttcgattatctaataaatcatttaacactccctttccaaaaaaaatcaatacaccaatcactacacttagatttattggatttgttgctaaaatatcggtattaaacccaaaacccccggcggatggccaatggcgtgagaaaacgaaagaatcggttacatttttcatatgctttcctcttatagataggactgacaaagaacaaaatttttttctattacttcgctcttttttgatcaatttatttttaattgaatttcccctttttatgggaatagattaaatctagtaatttcatttaggttaggtcttaggtctcatttcaattgtgaaatatatcgtttgtggaaacgtttcctaaaaggaaaaagtttccattgtactaagctaaggacgggaaggaagaaagcgagtgatctggtaattcctcatcctcaaagcagtccttcctgtagtctcaacaaataagtaattataggagtaattgatataattcgaagaagcaaacgattcaagttaataaaaaggtacttttttacattttgaaagtagattatcttaccattaatttcacaactttcatgatctcttcccgaaccaaacatgaatctttcgattcatttggctctcacgctcaattatttattttattttttgttatgggtattcccatattttttatgtaatgagcctgccctctcttttctgtttgtattcaaagatatctaaactgatacaagaccagaataaatattaggaggactcttccgaccagataaaaatcaataattgtcagcaaagttgtttctttatttgtatccaaaaattcctcttttttatacataggtcgtcgattcggcattggataaaaaaggcagagtgccttttctttctagtaaatggttcaaatccttttatcgatatgagtgttctatatcagataaattaccaactattcattttgaaaacatttcagtactaatgtagtcgtagaaagagtaccatgttttgcctggacttcaaacagtttagctttaaccatggtaatggtctcacattattggttgatagagaatcaaagttgatttaccaataagtcacgaaatgctatggttcttacatatgatttttgaatttattcagaagtaattcgtcgagatcgtgcaccttttttcctatttatcctaataactataaataaagtaaagtgcagccggatggatccaacctattcttgaaatacacaacccgcacttaaacaaaaggattcgcaaataaaagcgctaatgccacaaccagtccgtaaattgttaaagcttccataaaagctagactaagcaataaagtacctcgtattttaccctctgcttctggttgtctcgcaataccctctacagcttggcctgcagcagtaccttgaccaactccgggtccaatagaagcaagtcctacagccaatccagcagcaataacggaagcggcagaaatcagtggattcatctcacaaaaagaaatggttaatgatacaatcaaccaatgaattattacttaattttatcattaagatctattgggtcggagtaactaaaaattaatgataatattactgaatcgtcagaactacttcgatatctcattttttgtttctacccatgatgaagtttttgtaaatccatatcggctctagttattgcatttctttccaaccattctttcattccatccttcgttctttactcttctatatccttgagttcatctacacaatcacaaatgaaacagaagaaaggacttgacttatcttgtaatccatctaatctaaatgcagtaaactgcaatcaaatcaatatatgcatatgatcgatatctatatatagataggactatataactagtgaatatctattacatttctttcttccataacgtcacattttatattgaatcggattataaatcattcctcgaaacccacacaaaaagtggctggacttatagacattacatacatctagtgtgacctccccaacctttttttaattccgtaatatcgttcatcttctctcctacgactctaggtcatatattcatatttatatctattatgttctccaaccaagcagattatcttgaaccatgctgggataagctaaaaaaaagactatttagaaaactagtcaatgatgaccctccatggattcgcctatataagccgcggctaacgttgcaaaaataagagcttgaataccacttgtaaataatccaagaaacatgacaggtataggaactactgaagggactaaagaaacaagaacaacaactactaattcatcagccaatatattcccgaaaagtcgaaaactaagagataagggttttgtgaaatcttctaggatgttaattggtaaaagtattggagttggtttgatgtatttctcgaaataacccaacccttttttggtaagacctgcataaaaatatgccactgacgtgggtaaagctaaagcaacagtagtatttatatcattcgtgggcgcagctaactccccatgaggtaactgtatgattttccaaggtaaaagggcacctgaccaattagaaacaaaaataaataggaacatagttccaataaagggaacccaaggtccatattcttctccaatctgggttttgctcaagtctcgaataaattcaaggacatattcaaagaaattctgaccgtcggtcggaatggtttgtggattccgaacagctatgatggctgaacctaacaagatagcaattacgacccaagaagtgataagtacttgggcatggatttgtaaacctcctatttgccaatagaaatgttggcctacttctacacccgatatatcgtataaccccttgagtgttttaatgtaacatggtataacattcatattgtcctctgatagaaattgaacttcaaaaaaggaattagtttgattcaaccttctcaactcaccaacttgaatcatttatttaggataccaagaaatcacataacatcataatatatatcaatatccccagttttttttatcaaaaaaagtaaccgatccaaaattaacattaactaattttattattcttaaatgatatttgcgactttagaacatatattaactcatatctctttttcgatcatttcaattgtgattatgattcatttgatgaccttattagtccacaaaattgtaggattacgcgattcatcagaaaaagggatgatagctacctttttctctataacaggattattagtttctcgttggatttcttcgggacattttccattaagtaatttatacgagtcattaatcttcctttcgtgtagtttcttcattattcatatgattcccaagatacgtaacgatttaagcacaataattgcaccaagtaccatttttactcaaggctttgccatgtcgggtctttcaactgaaatgcatcaatccgcaatattagtacctgctctacaatctcagtggttaatgatgcacgtaagtatgatgttattgagctatgcagctcttttatgcggatcattattatcagtcgctcttctagtcattacatttcgaaaaaacatcaaaatttttttaaaaagcaataattttttaattaagtcatttttcattgaatatttgaatgaaaaaagaagtgtttttaaaaacacttcatttcgaaattattacaaatatcaattaactgagcgtttggattattggagttatcgtgtcattagtctagggtttacctttttaaccataggtattctttgtggagcagtatgggctaatgaggcatgggggtcctattggaattgggaccccaaggaaacttgggcatttattacttggaccatattcgcgatttatttacatactagaacaaatataaattggaaaggtacgaattcggcacttgtagcttctataggatttattataatttggatatgttattttgggatcaatctattaggaataggtctacatagttatggttcattcacataaacatctaattgaataaactacatgaagaatacataagataaaaaatcatcccatatataacgaaagtttttatgagtttttgagaaccatttaaattaatgattccttgaaatggttctcaaaaactcgagatgtatctaattacaattattattcattttatttcttttttcattatacagtacagcaaacgattttccaaatattaaattcaaagaattatatttccttccgtttcattaatgaaacaatgaaaaaaaagaaagcattgccttctttcccatatcttgtatctatagtatttttgccctggtgggtttctctttcatttaataaatgtctggaactttgggttacaaattggtggaataccgggcaatccaaaactctcttgaatatcattcaagagaaaaacgttctagaaagattcatagaattagaagaactctttctgttggacgaaatgataaaggagtacccggagacacatatacaaaaacttcgtataggaatacacaaggaaacgatacaattggtcaaaacacacaatgaatatcatctccatatcattttgcatttctcgacaaatataatctctttcgctattctaagtggttattttattttgggtaatgaggaacttgtcattcttaattcttgggttcaggaattcctctataacttaagtgacacaataaaagctttttcgattcttttagttactgatttatggattggatttcactcgactcatggttgggaactaataattggttcgttctacaatgattttggattggctcataacgatcaaattatatctggtcttgtttccacctttccagttattctagatacaattgtgaaatattggattttccattatttaaatcgtgtatctccttcgcttgtagtcatttatcattcaatgaatgaatgaagaactcatttgatctcctgatatcaatcaaatcagaatctttctttataaaccattttgaatcttacttaattctttatatttctaccagttcaaggtattcgtcctatattacagtacaactattccagtacaatgacagactcgtgcatagggaactttactagttctctatctaatttattgtagaaattccgagatccatgattggacttcgaccgctacaagatcaacaatgccatgagcttgggcttctgttgctgacataaaaacatccctttccatatcctcggatacaacccataaagggttgcccgttctttgtacataaacctttgttatggtttcgcgaagtttcagtagttcttccgcttccaggataaattcccctgcttgtgcctcataaaaagaactagcaggttggtgaatcataaccctagcgtgagggaatgctagacgtttggtaatttctcctccgaccagaataaaagatcccattgacgcggctaatcccatgcatatcgtatgcacattaggtgacacaaattgcatagtatcataaatggctattcctggtattacccatccgccgggagagtttataaacaaataaagatccctagtaccatcttctatactgagatataccatgagaccaacaagttgattcgagatctcgctatcaaccacttgccctaaaaaaagtaatctttctcgataaagtcggtttacgtcaatccaaaccgcatcttcctctccaggactccgaaaaggtacttttggaacaccaatgggcattaaattaaataaaaattgagtactatacttcactttaatatggaaacgtaacaatcaatggtttattgtcttcatcccttttttatctgattggaaagtttatagagtaagacagagaaatcgatcgttcgaatgataaacaagtatctatccattcgtttcccaaaaatgggaccaatcctcccattgcgtattggtacttatcgggtatagaatagatctgcttctctttgttcttacgaacaaaattgttctttcaatggaatggaataaatattaatcctttctgatacggaatctactgaaattaggtacatggtatatatagtcttttccaatgcgataaaataaagcggcatagtgtctatttttctttgataaagaggtatttccgatgatattgatataacggttcttctatctcgcatgattgggctaaactaaagtaggggataaaaaataacaagaaaaaaaatagaattgaataaccgtacaggcatcttttgttcattgcatacggctccgcaatggaattgactttttcttctcttctattctatcgaagaaagaaatagaatccatctaatccagatcgttgaatgatccatttaccacccttcctttcatagaagtaaaaaagaatactatgatggttctgttactttatatatttatctcgtctgtgatttagcaatcccaaagtttctttttgatacgatcaaataagatttttcgtactctttctttcatagcataagtatcagaaagagacttctggtgtggaagaaaaatggtttgtgacactgaaatgtactcccgacacataagtcaaatcggaaataacctttatttcatactactatctcgatacaaaatctcatgttatgaaaaacaataatggtttgttcatatcgaacccgaagtgccatgctattattacttataattttcttttataatcaatgtggcgaaggcatagtctttttttcaaataaaaactcattggcgccagaaccgtacgtgcacctttggatacatacggttcaaaaaagaaaaagaatcaatgtgtcgattccagttttattttnnnnnnnnnnnnnnnnnnnnnnnnnnnnnnnaaaaaaaagagattactgaacttattaaactaacctatcattgatgtattgtttcatcgatattaaaatcacgatgtcattgtcttgttcctgaatgggccctttcaattcttttaggttcatggtctaccccgggaaaagatatgtccgaattctatttgcacatataggacaaatggtctcagtaccatatcactacagtaatagtaggtataagaatcatttatgatacaagtggtaatcatacatatcaatttgttatcgatttggtattttctgaacggagcctggatactttattttatcagtccaagtaaaccataaattcttctaaattgataatattgatcccaaataaattgatctaattgcacttcacgctccgaatgattgattgatgcctcactcaatacaatatctcttgggcgaaacagaggatatctcgatcaggggagagaacgggtaaatcccatatgacccaatatgtatgacaagtcgcacctaatcgttcgaatctttgttgcgaagtcgataaattatacgtcccctggttgaatcataacgacttacttcaatttttactctatctcccggtagtatccgtataaaactgcggcggatcctccctgaaacatatcctagaattagatcttcattatctaaacggacccggaacataccgttgggaagtgattcagtaattaaaccctcatgaatcaatttttgttctttcattccaggtaacctccttgaagtatcaactaatggaggaagagttatataactcacttttcctctctattttacaaataggaagttagaaaattcggataccagaggtggaagatcaatgattcaccaggttattgatacggataatatccaaataccaaatacgttctcgacgtgatccatgtaaagaaaaaggggttggtttggggaagatcaaagaaagaacttgttcttcttccgtaaaaaattcttctaataataccgaacctaatctctgcaaaaaagagcgtaccgtactcttatgtttacgggccaaagttctagcacacgaaagtctaagtatatactttactcgatacaaactccgtttttcggaggatccactatgataatgagaaagatttctacatatccgaccaaatcgatcaataatatcacaatctgataaatcggtccagattggcttactaataggatgtcccgatacggtacaaaatttagctttagacaatgatccaatgagaggaataactgggactatggtatcgaatttcttaataccagtttctattagaaatgaattctccaacatttgattccttaccaccaaaggattttttagtacacttgaaagataacccagaaaatagaaggaatagtttgataattggtttatatgaatcctgtgtggttgagaccaaaaattaaaataatattgccatagattgacaaggtaacatttccacttcttcatcaaaagatgagtcccttttgaagccagaattgcttttccttgatatcgaacataatgcatgaaaggatccgtaaaaaaccataaagttttctgaaaatagttatggtacactactataagatgttccatttttccatagaactgtattcgctcaagaaaggttccagaagatgttaatcgtaaataagaagattgtttacgaagaaaaacgaataaaaattcgcattcagatacataagaattatataggaattgaaatagtcttttattttcttttgaaaaaacataaattgatttcttcggagtaatgagactattccaattatgatattcgtggagaaagaatcgcaataaatgcaaagatggaacatcttcgatccgacattgaaggatttgaaccaagatttccaaatggataggatagggtattagtatatctgacacataatttaaatgtaataatttgtcctctaaaaagggaaatattgaatgaatagatcgtaaattatgacattttggtatttctttttcttcgggaaaaggtactaatcgcagcgagaatggaatttccacaatgaccgcaaaaccttctgatatcatctgagaaaaaaaatgagaataaaaataattgttgtgcccaacgaatcgattttggttagaataattaaccgaataaattaaaaaattctgttgatacattcgaataattaaacgtttcacaagtactgaactagatttattgtcataaccaaaaatttccacgggttcgtaaaaaatcgaatcgtttaaaccatgatcatgagcaaacgcataaatatactcctgaaaaagaaacggatataggaagtgttgttgccgagatctatctttttctaaatatccttgtaattcttccatttacatttctacttgagccagaggcagtaggttttcttggtttatcaaatgatacatagtgcaatatggccagaacaggtaaagaaaaaatatataccccggaaaagaaagagggagtccaatcaacagatctttttaccttttctatccaattggtttatgttcgttataattacaacaggaaaaatcctttatttttgcaacccaatcgctcttttgactttggaataaattctctttatcaatatactgtttcttctacacatctgtctacaatccataataaagaataattaggattctggttcactcacaggagaacccactctttcccgcattaggcactaattcatttttaacatctaattagatcgggtaatcattccaattaagaacataagctcgttgctttttattttaccagaattggagccatagagctctatccatttattcactagacccaactgtaaatgtgaatttattttgttcccttccaaaaatcataattgattttattacgacatgctgttttttccattcattacccttgaggatcagtcgtggtcttatagactataccaatagtctggacgaattcgttgcttcatccaaatgtgtaaaagatcatagtcgcacttataatgaaagaagttgggaagaagttgttaataacagattacctagagaaataggtaaaagaaatttccatccaagatttaataactggtccattctcattctaggtaaagtccatcttgttgtgatagaaatgaagagaaacaaataagctttagttaatgtaataaggatacccattgtcattccaaaaatgctggcgattttttttattccgaaaagctcagaaatggatatatacggaataggtaaattccacccacctaagtaaagaactgttacaaataatgaagaaactaataaatttaggtaagaagcaagataaaataaaccatatttgatacccgaatactcggtttgataacctgctactaattcctcctccgcttctggtaaatcaaagggcaatctctcacattcggctagagaagaaattagaaaaacaataaatcctataggctgacgccacagattccacccccaaaaaccatattttgactgtgcttcaactatatcaactgtacttgaactgttagataatagagatattgataacacacatagagttaatggtatttcataactaatagattgagcagcagctcgtagaccacctaaaaaggaatatttattattcgatccatatcctgacataagaagcccaataggagcaatactagaaatggcgatccataaaaaaacacctatactgagatcggctaaaacaagacgatacccaaaaggaattactaaataacttagtagaattgatataaccgctatagacggtcccacactaaacaaacgaatatctcctcgagatgggaggaggtcctctttaaaaagtagtttagtcccatccgctatagcttgaagaattcccaacgggccagcatattcaggcccaatacgttgttgtatcgctgcagatatttctctttctaaccacacaattactagtacccccattgttattcccaatataagggtcaaaatgggtacaatccatattagtccatacacttcttttaaaaattccgatgtataaaaagaattgatagtttgtacttctgtcgtatcaattatcattcatagtcgataataacatcactgttcccatcgctatttcaaaaccgtacgtgagacctcagcttcatacggctcctcaatggccacaaaaataaggatgggttcggtattatcaccatctttggatggatagaataaagatacatcggaaagtcccaaattagaccaaggaattctgtctgctagaataagaaaaaagtgcttccgaattgatctcatcctttacaataaaaatttctctttgttcggtaataacttaatatttcaataaaatactccttatatcaatcaatacaaaataaaaagaattagtcattagttcatgaatcgtgataagaattcaatatgtatgaatatggatagagaaaagaataaataaggatccctttttttattattcattcaattactacattccatttcttttttcctgttcttctgtctcagaggaggatactaaaataaagaattaattcgttcttgatagtcatttctttaaccagtgaataggagcatactctagatcggaatcgtagggaagtactacttgatcatttctaccaatttaaagtccttattatgattcgttttatgaagaaatacctcttttttgataccctacattatctccattactaatcctttgtgtaccttggtgttcctaaccgtccactggcttttgattgatccccggtatagtaatacatatgagacagtagtagaaactccatacagttgatcttttgtttgcgcccgcttcaagatatgatgactaatcaaaaaatcttaatcttggggtaagcagtttacactgcttatttttacttcaactttattcttgtacatagggaatgagattgtttcttcttactacaaattgggaagctgtttagtttcactcatataactatctggtttaactcatcaacccgaatgctgaataaaaaagaaaacaatacattgaacctctttcttttttcttttatttctagaagagttcatattccaacgaatcacacctaaaagagggtatcctgagcaattgcaagaattgggttcattgatattcctggtatagtagatgctatcacacatacagtcatactcaattcgatggaattgtttgatcttaaaggggatcttctataatttcgcacgtaaggggttatttcttggtttcgtccagtcattaataacttgattatttttagataatagtagatagaaacaacgctcgtaaggagtcctattgaaaccaagaaatataggcctgcctgccatccacaccagaatagatagagttttccgaaaaaacctgctagtggaggaagacctcctagggataagagacatagggctgaagagagagccaaaaaaggatctttcgtgtataatcctgcataatctcgaatgttatcagttccggtacgtagaccaaataatacaatgcgagcaaaagttcctagattcatggagatatagaacagcatataagttatcatgcttgcatatccatcatttgagtctccaacaattattccaataattacatatccgatttgaccgatggacgaatatgcaagcatacgtttcatgcttgtttgagtaatagcaatgagattccccaatatcatgctaagaatagctaggatttccagaagaagatgccattcgtttgatgagaaataaaaaggaatatcgaaaattcgcgtggctgaagctgaagcagctacttttgaagtaacagaaagaaaagcaacgactggagtgggagatccttcgtatacgtcaggagtccattgatgaaaaggggctggggaaagcttgaacccaattcctacagtgatgaatataagcgcaattgaaattcctggggagttatacatttgtgtattgataagaccattcactatttcttgaagctcgatctcccccccagatgaaccatatagccaagagaaaccatgaaccagaatagaagagcttgccccacccataagtaaatatttcatagtagcctcattagaccgtacatctctcttggtatatccggataataggtaggaacataaactgaaacattctggagctacaaagatagttattaaatcgttagcaccacataaaaacattcctcctagagtagctgttaatacgaataacagaaactctgttatagccatttctgtacattcaatgtactctacggatagaggaatacatagagttgaacatagtaaaataagaaattgaaagatttcgttgaaattgttcgtttggaaatttcccgaaaagctaattataggttcttctctccatcggaacaatagggccgttatgcttattactaaacttgttgaagagatgaaatagaaccaaggtctatctttttgatcagaggttgaatcgatcatcagaagaagaattagaccaaaaattaggatacattctgggaaaatgaaacttccatggaagagaagcaaatgaaacgctttcataaaaattctcgtagaatcgagaatgaagttttcattctgtacatgccagatcatgaattagtaactgcatccaatctccgaaaaagtcccaattgtttcgaactttctatttttggaatgggatatttacggaatcccatgaataggatcaaaccttattccatgatatttccataagattcctctttcttattcttaagcaagcccagggcttagttgatcatgatttatgttttatctttcttttcctttttgtttgtttcgagaatcgtccgattctttttcttgtgtctacatagatcctgttcatggagtcagagtcgaaaagaggattcctcacttctttctctcattcaaaaccgtgcatgagactttcatctcgcacggctcctaagtgataaaagaaagaagaactcatcttctttcttttttgattaccttcctcgcgtatgtataagaccgaatccattcgatttctcgattactaatccttaacttttcgaggaatccttcatcagtggttgtgaatgactgactttttcaatcctttcgaccttggttccgtaggagcaagtcagaaagattgagaaatagaaccatctgatttgattcgttctcaatagccatgagatgatcatcttagggtgatccttttgtcaacggatgctcctattacactcgtagtctctgaaggatgagaaccaactatgtagcatctacatcgataattcaagcattgtatacgtcattagtccgatcctttgtaggaactacccgtaataacgaacttgtaaaatggatctgtttatcataaagagattcgttgttcctgaccctgcttcaccttaattgttatttgaacaaaaagatcaccttttggtaaaagttatgtcttggtccgagtggggatagcatttctcttctgcatgtctatggagttttgaaaaatccaaacatctcagagatagatatagaggtaggaatttgtcgaacgaaccgcacttaagaccattccaacgctccctttcgccatgcataaactgaacccacaattgggataagcacgaaaattaaagcttcgataaatacagatacacccaatacatcgaaactcattgcccatggataaagaaagaccgtttcaacatcaaaaacaacaaaaactagagcaaacatgtaatagcggattcggaattgtaaccaagcgtcccccataggttctatgcccgattcataactagagagcttctctggtcctttactaaccggggctaaaactcctgaaattacaaatgccaagataggaataacgcttgatattattagaaatgcccataaaatatcatattcgtgaagcagaaacataaatgtactcctattaatgttggcattgtcaattcatccagaacttgttttcctaggtgaaacaagaatttattttaatcaaagtgtatagttcagagtttgtttgctgcgtggcatgtcttgtttagagattcatccaaggaatctcgattcctttttgatttcgattctatttagatatggtgtagaggcgttcttacacctctattctctattttctttttcttgtcctagatttgacacatactgatctgattagatccattggaatgaattattgtttttattttcactatgtatttacatgaatatgtggtaatgctcatttctatgaaaaaccaatggtctgtccagtctataaacaagtactaatgaggaaatgaaaactatactaaaaatagaatgtctctatctatgataataattggataagatagcgtgtaccttgtcaactgataacgagagaacaaaatcgggataaataccaatacttattacgggtagaaagatacagattgaaagaaatagttctcgtagtccagaatcccaaaaattagagtttggaatattaaataacttgtatccataaaacatctgacgtaacatagataataaataaataggagttaatatcattccaattgccattacaaaagtaattagcatttttgacattaaaagatattttgtactagtaattagtccaaaaaatactacaaattccgcaacaaaaccactcattcctggcaatgcaagagaagccattgagaagctactgaacatggtaaatgtttttggcattgggatagatatccctcccatttcttcgagataaacaagacgtgttctatcacaactcgttcccgccaagaaaaaaagtgcagcaccaataaatccatgagagagcatttgtaaaatagctccattgagtcccatgtcggttatagaaccaattcctataattgtgaaacccatgtgagatacagaggaataggctattctcttttttaaattacgttgaccaagagaagttgaagctgcatagattatttgcattgttcctattatcaccaaccaaggagaaaatatagaatgagcgtggggtagtaattccatattgatccgaatcaatccatatgcgcccatttttaataggattccagctaaaagcatacatgtactgtaatgtgcttctccatgggtatcaggtaaccatgtatgtaggggtataatcggcaatttgacagcataagcaataaggaagccaaaatagaatattatttccaatgccgcaggatatgattgattaattaatctttcaaaatctaatgttggttcattggaaccatataaacccatacctagaactcctattaagaaaaaaatggaaccccctgcagtgtacaaaataaactttgtagccgagtagagacgtttctttcccccccacatggataaaagtaagtaaacaggaattaattctaactcccacatgatgaaaaaaagtaaaaagtctcgagaggaaaataaccctatttgaccgctgtacattgctagcatcaggaaatagaacaaccgcgaatttcgagtaattggccaagctgctaaagttgctaaagtagtgataaatcctgtcagtaaaatgggtcctatggaaagtccatcgattcctagtctccagtggaaatcaaaaacatctatccatttagaatcttccttcaattgcattaatggatcatccaattggaaatgataacagaatgcataagtcgttagaaggagttctaataagcatatacacatagtataccacctaaacatcttattccctctatgagggaaaaagaaaattgaggaacccgcgaatatcggtaaaacaacaagtattgttaaccaaggaaaataactcgtgataaagactagatacataaagcccgtcctcaaaataatatattttgtcgagcacgggcttttgtcggtaaagaggaatcacaaacgattcaagtggatttttgtaacgtactaattattcaataaattcgattgattaatacgagttgattttctgttacgatggatcgacgaaacaatagcaagtccaatagctgcttcagcagctgcaatggctataccaaagattgagaaaatgtctccctttaattggcgactatcaaatatatcagaaaatgttacaagattgatattaaccgaatttagtataagctcaagacacataagcgctctaaccatgtttcgacttgtgatcaatccatagataccgatagaaaataaataaacactcaaaaaaaggacatgctcaaacatcattgactaactccttatcaatctcgattcatttcaatatgaacaacaattcaaccgattcaattgattagaatagaacaattacacaacaaaagaagatattgacggtagatagatttaacttagaatttagttagaattctaagaattgggaatcattataagttaagtatttttattgcttttattagaaataaaaaaataaataaaaatattgatacataagataaatacaagaatagataagacgatattcgtccacctcccatatatttaatcccttcccctataaaaaaacttgcaacaccaacaccatttgtaattccatcaattatacgtctatcaaaaaactgagttagtttggttaatcctcttatccccacggtaaaaactctagtataaaaaatatctatgtaaccacgattatatgaccaattgtatatcacattttttattcggtccacaagaattcttttaggacccctctttacaaatgaattgattaaatccaaattctggaaaaatgaataagcagatccatataaaatatatgctatgaatagtccgaaaattgctatacttaccgaaaaaatggcatttgtaaaaaattcattcaaatttttagaacttgaatgtaaaagatttgttgatggggttaaccattttgataatatatcaaattgatcaaaagaaattcctattgatccaaccaacaaagtaaatagtactaatacaagaagaggaaatagcatagtattgtccgattcgtgaggatacatggaagtgtatttatttccaaagtaagtactaaagtatcgtatcctatttcttacattattatcaattttcgatctttcttttgcaaaaaaagaaacctttttattcattgttgataaaagtaaatttggattgactcctcttggagttccttttccccatagagatatggaatagaacgaaccatttttcgtgttactgtaatttttaaaatgaacgcggaaatacccatcaaaggtaagtaaatatacccgaaacatataaaatgcggttaatcctgctgtgaaataagctattactgcgaaaattggtgaatacaaccaactatcattaagaatgtcatctttggaccaaaaacaagcaagaggtggaataccacaaagagaaagtgtacccaataaaaaagtagttcttgtaattggaacatatcttgttaaaccacccataagaaccatattctgacttttatctggtgaatatccaacaataggttccattgaatgaataatagatccggatcccaaaaacaataaagcttttgaataggcatgagtgatcaaatggaataaagcagctcgataagaacctatacctagagctaacataatataacccaattgagacattgtagaataggctaagctttttttaatgtctctttgagcaagggccaaagtagcccctaataatagcgttattacacctattaaagaaatgaaattcattatataaggtatgactatgaaaagaggaagaagccgagctacaagaaaaattcctgctgctaccatagtagcagcgtgtataagagccgaaataggagtgggtccttccatagcatcaggtaaccatacgtgaagggggaattgtgcggatttagcaactgcaccgacgaataataaagaagcacacaaggtagcaaataaagaattgacctcattattctggattaagttattagttatttcaagcaaataccgaaattctaaactacctgttatccaataaaaacctaagattcctaacaataaaccaaaatcccctacacgattagttacaaaagctttttgacaagcacttgctgcaattggtcgtgtgaaccaaaaacctattaataaataagaacacattcccacaagttcccaaaaaatataaatttgtatcaaatttgaactagtaactaatcccagcatagaagtattaaaaaaactcatataagcaaaaaatctcaaatatccttgatcgtgatacatatacttgtcactataaataagaaccatgattccaacagtagtaattagtattgacataatagaagtaagtggatcgatcaagtatccaaactctaaggaaaaatcattattgatggtccaagaccatagatattgatagataaaacttccatttatttgttgaatagacagattggctgaaaacaccatagctatacttaagagtaaaacaataggaaaagcccacatgcgacgaatattttttgttgctgtcggaataagtagaagtccaaatcctattgacatagtaactggaagtggaagaaaaggtattatccacgcatattgatatgtatgttccataagaaaagaacctttttttcttataattacaaattgttatcgattcaccttagaaaagatacaaaaaaagaacgatttgaagaattattgtcgagccatagtaattgcacctatcaaggaaactaaaagaattattgaaatgagttcgaatggaagataaaaatctgttgataaatgaatcccaatttgttgaacgttatttattaggtcctgttccataatctggtttgaccttgcagtccaaataattccataccatgacgtatctgggatagtagtaattagtgaaaaaagaatagttgtacaaaccatcaaagtgaccccatctccaatggtccaaaaattggaattattggaatattctgaaccattcatgaacattacagcaaatatgatcaagatatttatggctcccacataaataaggagctgtgcggcagctacaaaataggagttcgatgaaatatagaataaggatatacaaacaagaaccaatcccaatgaaaaggcagaataaattggattggtaaataatactacccccagactccctaatacaagaattgaccccagaaatacaacaagaatatcatgtattggtccaggtaaatccattatgtataaaatacaaaataaataactttttcatgaccttaactttaaatggtccaggaaaggaaaaggggttaccctatttttgttttcttgtatataatattgtatatgatacatttataattgaatatggattaatgtagatatagataaaattatcgggccaaccttaaaaaagtagttgaaatttgctatttcgaaatcatcactaaaaatatatttttagtttaaatcaaagagtgattctcaacaatcattagtttttatttgtagttactgactacccaaaataaatggatcctttatatcaaaacaaaatctcaacgatcaacttctcccataatgatatctatactacccaatatcgtcatgatatcagccaatttcattcttttaactagctgaggaagaatttgcaaattgataaaaccaggtggacgaattttccatctccaggggaaaacactattatctcctatcaaataaattcccaattctccttttggggcttccactctcacataaagttcttgtttcgacaattctaaattgggtgaaggttttttactaataaatctatattcaaaatcattccattcggaattctttgctctatcaaagcgtcgtacttctaaattttcataaggtcctccaggaattccttctagagcttgttgaataatttttatggattccttcatttcaccgattcgtactaaataacgagctaatgaatctccttctttttgccattggacttcccaatcgaattcattgtaacactcataatgatcaactttacgaagatcccattggattccagaagctcgtaacatcggtcctgataaaccccaatttacagcttcttctccaccaatgatgcccactccttcaactcgttccaaaaaaatgggattccacgtaataagtttttgatattcaacaactcctgttaaagaataatcgcagaaatccaaacatttatctatccatccataaggtagatcagcagctactcctccaatacggaaataattatgcatcattcgcatacctgtggcggcttcgaatagatcatatatcaattccctttctctgaaaatatagaaaaagggagtctgtgcaccgatatccgccataaaaggtccaagccataacaaatgagaagctatacggctcaattccagcataattactctgatatagctagctctttgaggtacttgaacattttccaattgttctggcgcatttacggttattgcctctgtgaacatagtagctaaataatcccatcgtgttacataaggtagatattgtataattgttcgattttccgctatcttttccattcctctgtgtaaatagcccaatatgggctcacagtcaataacatcttcaccatcgagagtaacgattagtcggagaacaccatgcattgatgggtggtgaggccccatattgactatcatgagatcttttcttgtaaccggtactgtcatatcttttcttccttaattcattattccataaaaagagactcatcaaaacgaaaaattgaatactaccgattaaattagtaatcggtaattgttcttgaatcaaagggtttatctttgtctatttttatttgagtcgaattcataactgtttgaattgtataatctccaattattgagattggtaatcgacccaaagcaatttgattataattcaattcgtgacgatcataagtagaaagttcatattcttcagtcattgataaacaatttgttggacaatactcgacacagttaccacaaaatatacaaaccccgaaatctatactataattaagtaattgtttctttttaatatctctttcaaatctccaatcaacaacgggtagatctatcgggcatacacgaacacatacttcacaagcaatacatttatcaaattcaaagtggattctaccccggaaacgctctgatgtgatcgatttttcataaggatattgaatagttacaggtaaacgatttgtgtgggataaggtaattatgaaactttgaccaatgtaccttgcagctcgtattgtttgttgaccataattgatggacccaattaccatagggaacatattgtagatatacatgaaaaatttgacgtttctttctcttgtttgatagagattatgaatctaaaatagtgttatcgtattctcttattcaatgagcatcttgtatttcatagaaatttggggtaatatagtccttacgtaagggccagcctatccaactttcaggcattaagatacgtttaagacgtggatgattattataagagattcccaacatatcataagattcgcgttcttgaaaatcagcacttctccaaatccagaaaacagacgggattctaggattatttcttggagcaaatacttttatacatacctcttctggtttatctataccatactgtattcttgtaagatgatacacactagctaaaaatccgcctggtgctacatcataggcacactgagagcgtaaataattgtaaccatatacatatgaaatgacagcaatggagtcccaatcctcggtttttatttgtaaagtctctattcctcggtaatcgaagcccaaagatctatgaaccagctcatgcttgactagccaatcagataagcgattctgatgcattgtcttgatctctcccacatttgtataagtatttcacatttacaatgaaatttttaaagattgacctgctcttttgaaaaatgtttcagaagatatctctgaagtagatggtgattgatagagcaatccttgatcgtaatttccagtatgagtactgtgtcgaacagaaaatttgtgattggtagtaaaatatcgatttttctgttgagatacagctctatcttcaaatatttctcgagatatcttcttacgaagttttgttatagcatctataactgcctctggtttgggtgggcagcccggcaaatagacatccacaggaattagcttatcgactccccgaacagtactataagaatcagtactgaacatccctcctgtaatagtacaggctcccatagcaatgacgtattttggttcaggcatttgctcatataatctcactaaagagggagccattttcattgtgactgtgccggctgttaaaattaggtccgcttgcctaggacttgatcttggtaccaacccataacgatcaaagtcgaatcgcgagcctattaatgaagcaaattcaatgaaacagcaactggtaccatatagaagcggccataaactggagagtcttgaccaattcgaaagatcattcgatgtagttgaaataactgaattgggggttgtttggtcaagtaacggaaactcaatcaaattcataactgtctcaatgtaattttttccttccttttttttgtctgaatactcagatgcaaaatagaaatactttttcttgggtaaaggaacagatgactcgatccatttctgtatcgatcatgatatatgtaataactcgagcatccatttcaaatgcatatcccatttttgcgcagcagggttatgaaaacccacgagaagcaactggacgaattgtatgtgctaattgccatttagctaataagcccgtggatattgaagttccgcaagctgtgcttcctgatactgtatttgaagcagttgttcaaattccttatgatatgcaactgaaacaagttcttgctaatggtaaaaaagggggtttgaatgtgggtgctgttcttattttacccgagggattcgaattagccccccccgatcgtatttctcctgagttgaaagaaaagataggaaatctgtcttttcagagttatcgtcccaataaaaaaaatattcttgtgataggtcctgttccgggtcagaaatatagtgaaatcgtctttcccattctttcccccgaccctgctacaaagaaagacgttcacttcttaaaatatcccatatatgtgggtgggaacagaggaaggggtcagatttatcctgatggtagcaagagtaacaatacagtctataatgctacatcagcaggtatagtaagcaaaatagtacgtaaagaaaagggaggatatgaaataaccatagttgatgcatcggatggacgtcaagcggttgatattatacctccaggaccagaacttcttgtttcagagggtgaatccattaagcttgatcaaccattaacaagcaatcccaatgtaggagggtttggtcagggagatgcagaaatagtgcttcaagatccattacgcgtccaaggccttttgttcttcttcgcatctgttattttggcacaagtttttttggttcttaaaaagaaacagtttgaaaaagttcaattgtacgaaatgaatttttaggtccagagattccttaacatttggtaaaaagtgccgattttttgtccatcgatacaattatgtatgatcaaaaaattctgtaaatccttttgcttgctttgtttatactctttttgttttgcaggacgcctggaattcattacttgtattcctagaggcatacaaacaaatacaaaaaaagcaaggatggtaaaaatgaaaaattttaggaaggattactagtcttcctaatcttctattcgacaggaattttcacccgttttcttgtgtcgtcttgtatcaaaataataattatttttttcctgttcatcaaagattactattcctttccttcttttccgggtctatcggaactcctttgtttagattcataagaagtagtggacaaacaaaggaaaaaaggattatgggcgaagaaaatagataagttaaacttctaacttagagaaattattcaaaaaaaagactgttccggttgaaaggcggatttgattaaggttctattagtttagtctagaaatgatttgcaggatgtctcatccctagaaatcaatataatttatattggattatagataaaatcgattgattcgttctttcttcttgcttccagagagcaaaggatcctttttgtcatttctacaaatataatatctggataactaaccaatttgtaggttatggaatagatcaaagctcattataagaccgcgggccctttccgctctaatcagacaaaggagggtaaggacccgctaagttcttactttttcatgtctacaacccagcatgagtaaagtatatgattggtttgaggaacgtcttgaaattcaggcgattgcagatgatataactagtaaatatgttcctcctcatgtcaacatattttattgtttaggggggatcacacttacttgttttctagtacaagtagctacgggttttgctatgactttttactatcgtccaaccgttacagaggctttttcctctgttcaatacatcatgactgaggccaactttggttggttaatccgatcagttcatcgatggtcagcaagtatgatggttctcatgatgatcctgcatgtatttcgtgtgtatctcacaggtggatttaaaaaacctcgcgaattaacttgggttacgggtgtggttttggctgtattgactgcatcttttggtgtaactggttattccttacctcgggaccaaattggttattgggcagtaaaaattgtgacaggcgtacctgaagctattcctgtaataggatcgcctttagtagagttattacgtggaagtgctagtgtgggccaatccactttaactcgtttttatagtttacacacttttgtattgcctctccttactgccgtatttatgttaatgcactttccaatgatacgtaagcaaggtatttccggtcctttatagagaagacatatctgtaattgatcatatatcggggaggacaatagtatttcattgctacaaatatggattattgaaaaaataagacatgtttttggatacttctcttcaatattgtattccttatttgatacgaatagttgaagtgaattttctgaagagaggatggattgtgtgacttgttataattgatcctattgataatacatagaatggacctgttatctctatcaagatgattctacctcgtcggatatttattctagtatctggggcacggaatatatagaatagatcaagaaaagaaatatttgaactatgattcatacctactattcagacctcgcaaccggactcaaaaaaattcaaataggtatttcctaaatcaaacgaattttattccttcagatttattttgaccgaaggataactctttctctagattttgttgagtcattacatccattcattcaataagtgatcatcaaaggttcttactcgagaacctttgagtttagcttgaggctaaatcatcgtggttctagtatgaatctgaggtttcaattgattcatagggtctcaacaagagaattcctatcaatagtaaaagtaaatccgcaaaaatagggaagagaagattcaagaggcctgtaacgatcaacataaagaaagacagatgagccaacttgatattttttggcattatcatcacaaagaagagattccggatttttttcgtatcttcgaggcaaatcaagtggttaatgaagtttttaactttctattagatatccgttgaaatcagtatttgtgtgtttccgcttgagccgtacgagatgaaattctcatatacggttctcagagggggagttccattgggttacctatctcaatatgggaataacaaagaaacctgacttgaatgatcctgtattaagagcaaaattggctaaagggatgggacataattattatggggaacccgcgtggcccaatgatcttttatatatttttccagtagtaattctaggtactattgcatgtaatgtaggtttagcggttctagaaccgtcaatgattggtgaaccagcagatccatttgcaactcctttggaaatattacccgaatggtacttctttcccgtatttcaaatactccgcacagtacccaataagttattgggcgttcttttaatggtttcagtaccaatgggattattcacagtaccttttttggagaatgtcaataaattccaaaatccatttcgtcgaccagtagctacaacagtttttttgatcggtaccgcagtagctctttggttaggtattggagcaacattacctattgataaatccctaactttaggtctttttcaaattgattcaaccgcgaagtacggaatagttacttcaaagtgaatcttccctagatacattaatttgattatgatctattccgcgaaaatacggatcgtgccaaagatgaaaaatttttttctttagaattagaaaaagaagatgaaaaaattacaatggatgtgtgacttgaactattgatttagccatgcagatatatgaatttgccacgttggaattcacaaccaaacgtgtctccgcatccaaccaaaacgtaagccccctacgtagcagaggataggccggttcgcttgaggagaatattttctatgatcataccgaatcatgttattcacgaataggctccgtaagatccgtagaatagaataagtgatgtggcatgatccaatgttctattccacttatttattatagtgtggaaatgcattcattccctttgcatcgattccgatctatgatactatcggagtgaaagaagggatctaaggaagaacagaggctagactttattagtaacaagtaaatactttttacgtaagaaactcgagatatggtgggtataaacaccaatcaaaagacatgagacaatccaaaaagcacttgatcatgatcaaatttttaagcctacttggatattgagcatttgcctataagaacagaattctttgcaatgaatagtttgcaattctggaaaattgaatgtgataaatctttcttacatagagttattaactatgttattgtatgaaataacgattttttattttatatgggtttatttctgttattcctttgattcttgctcgagccggatgatgaaaaattatcggatccataagaattcacctatcccaatgattgaagtttttctatttggaatcgtcttaggtctaattcctattactttggcaggattatttgtgactgcatatttacaatacagacgtggtgatcagttggacctttgattgagtaacatttctttttttgattgacctcctacgggaggaggtcaaattccagttgcaattcaactttgttaagttattttattgtgattcgacatacataagatagacagaatatgcttactataactagttattttggttttctactggctgctttaactataaccccagctatatttattggtttgaacaagatacgacttatttgaaaatgaattgaataaataattcagaaaaatatttctcgggaattccagatattctatggttctttcccgcaccaattgccaatttttttcttggtcattgagattcacggataattcagattaatatttagggatagatcttacccctttttatcccctcaaacaaaccgaaatggatatagtaagtctcgcttgggctgctttaatggtagtctttacattttccctttcactcgtagtatggggaaggagtggactctaattatattaataattgattgagtaatcgaattgtataaattgtttatttttcgaagcttttttttaaattctttcaaaattatactggaaagacaataatgaataataaccattcgatcaaacaacgaatgattactttagttgtatttcaaaacttacgcatgataggaaattttttacaaccgaattcctcctaaatattctgtttggataaacctgttcttaccagaattttggatattacaatgagaaaaaaccaatccctagttttttctttatttgtttctctctttctttactttcactgttctaagaacaaatcgttctgctattagattacgacgatacttactttatactggaagtgactagtggttgaggttctacacataaaaaaattggatctttcttccgctgagtgatccaccacttagactcctagagatttttttatgcttttttgacatgtcatgtttaagcatgaaaaatggtccgagtcccctttactaattcctttcaaaatctgaagaagttaccatagaacttcgtaaatgatctgttaatggctcaatcaatgacttcttgggatgggaaatcaaaaaaattccttgttttgctatagtatattcctatcttcctctattgattcttttgatggataccggaacctatatataaattataagaaacctaggaattagaagaattggccttcgattattttgggttgatcgaaatcgagtggatgtagcgaaaaaaaatcgaattagactgctatttcatctgtatacaatacaactttatatgatcattgtatacaagataatataaaatactctaaaatgtggtagaaaggactatatatagtcctttctaccacattttatgattccaaccagatcatttcatttaagacttggaattttcttttaatcccttcttcaatcattgaaaaaggattgataagaactatcagattatcctactgcaataattcttgctaagaagaacgcccatgtagtggcaattccacccagaaggtaatgggttactcctacagcacgtccttgtacaatgctcaaggctctaggctgagtagcaggagcaacttttaatttgttatgagcccaaacgatggattcaatgagttcttgccaataaccacggccactgaatagaaacattaaactgaaagcccagacaaaatgagcacctaagaaaaaaagaccatatgcagataatgaagaaccataagactgaataacctgagatgcctgtgcccataagaaatctcggagccacccattaatagtaatggaactttgtgcaaagtttcctcctgtgatatgagttaccaccccttgatcacttatagtaccccaaacatccgactgcattttccaactgaaatggaaaattactacagaaattgcattgtacatccagaatagacctaagaagacatgatcccaggcggatacttgacatgtccccccccgccctggtccatcacaagggaaacgaaaaccaagatttgctttatcaggaatcaaacgggaactgcgagcaaatagaacacctttcagtagtatcaatacagtcacatggatcgtaaatgcatgaatgtgatggactaaaaaatctgcggttcctaatggaataggtaacaaagctactttgccgcctactgctactaactcaccacctccccaggttaagctggtactcgttgttgcaccaggagctgttacgccaggtgctaaagcatgggtattttgtatccattgagcaaagatgggttgtaattgtatagcggtatctgaaaacatatcttggggacgtcctaaagcactcatggtatcattatgaatatacaagccaaaactgtgaaaacctagaaatatgcatacccagttaagatgtgatatgattgcatcgcggtgcctaaggacgcgatctaatagatcgttgtatcgagtagttggatcgtagtctcttaccataaaaatggctgcatgcgcagcagcaccaactatgagaaatccaccaatccacatgtgatgtgtgaacaacgaaagttgtgtaccatagtcagtagctaggtatggataggggggcatggaatacatatgatgagctacaacaatggttaaagagcctaacatagccaggttaagagataattgagcatgccatgacgttgttaggatttcatataggcccttatggccctggcctgtaaatggacctttatgagcctctaaaatgtccttaaggccatgaccaatgccccagttggtcctatacatatgaccagcgatcaggaaaagaatagcaatagctaaatgatggtgtgcaatatcgctcaaccacagaccccctgttattggatctaatcctccacgaaaacttagaaattccgcgtattttgaccaattcaaggtgaaaaagggggttgctccctcggcaaaactgggataaagttgagccaaaaggtcccgattcaagataaattcatgaggaagtggtatctctttaggatcaactccagcgtcgagaaattggttaatcggtaaagatacatggatttggtgtcccgcccaagaaagagacccaagtcctagtaaccccgctaagtggtgattcaacatggattccacatcttggaaccaagccaattttggggcggctttgtgataatggaaccaaccggcaaaaagcattaacgctgcaaagaccaatgcaccaattgcggtacaatagagttgtaattcactagttattccagatgctcgccaaatctgaaaaaaaccggaggttatttgtattcctcggaaacccccgcccacatcaccattcaatatttcttgacctactattggccaaactacctgggcactgggtccaatgtgagtaggatcacttagccatgcttcataattggaaaaacgggcaccatggaagtacatgccactcagccaaagaaagataatggagagttgaccgaaatgagcactaaatacttttcgggagatctcctccaaatcactggtatgactatcaaaatcgtgagcatcagcatgtagattccagatccaagtggtagtatcagggcccttagctattgttcttgagaaatggccgggtctggcccattcctcgaaagacgtttttataggatccctatccacaacaattttcacttctggttccggcgaacgaataatcattaagtcctcctctttccggacaacacatacaaagagacccgccaacagtcaagtttttagtgaacctctgaaagatagatattttatttagttattcactagagcaattatgatattgaagtcgatccgaggcaagtgttcggatctattatgacataatgattaggtgcccaacggacctttaccttggaaaatcctttccggcgtgacaaaaagcaacctagtgtatttatatctgaatgaaggtataagtacctatatggatctacttacatggaacataatatcttttacaaatcacaagatcattcattagaaaagataaaatcattctgatatcttaacaaaatataatgcagaaggaagagatataataaaattcttgattagatcttctcataggaacgatctttttatttgattgatggatccaacaaccaaacctaaattcattaaaaaagagagtggttttaaccaaattttcctgatgtagaggcaatcaagaaagctgcataagtaaatatataacccacggaaaagtgggctaatccaaccaatcttgcttgtacaatggaaagagccaccggcttatctctccatcgaatcaaattagccaaaggtgtgcgttcatgagcccatgctaaagtttcaatcaattcctgccaatatccgcgccatgaaattaagaacataaatccagtagcccaaacaagatgtccaaataagaacatccacgcccataccgataaactattcataccaaaaggattatatccattgataagttgtgaagagtttaaccatagataatctcttaaccatcccatcaaataagtggaggattcattaaattgtgaaacattaccctgccataatgtgatgtgtttccaatgccaataaaaagtaacccatccaatggtatttaacatccagaaaactgccaaataaaatgcgtcccaagcagaaatatcacaagtaccgccgcgtcctgggccgtcacaaggaaaactataaccgaaatcctttttatccggcattaatttagaaccgcgtgcatctaaagcaccttttactaaaatcaatgtagttgtatgcaaacctagagcaatagcatgatgaaccaagaaatccccagggcctattgttaagaaaagtgaattactattctcattaatagcattcaaccatcccggtaaccatatgcttcgacctgcattgaaggctgggccattcgttgaagataagagtacatcgaacccatatgaagtcttaccatgagcagattgtatccattgggcaaatatgggttcgatcaagatttgtttttctggagtaccaaaagcgagcatgacgtcattatgaacataaaggcccaaagtatggaaccccagaaacagactggcccaacttaaatgagatatgatagcttctttatggtctaacattcttgccaatacgttatcctcattctgctccggattgtaatctctaatgaaaaatatagctccatgagcaaaggctcctgtcatgatgaaccctgcaatgtattggtgatgagtatataaggcagcttgagtagtaaagtcttgtgctatgaatgcataagcaggtaaggagtacatatgttgagctactaaggaagtaataacccctaaagaggctagagcaagacctaattgaaaatgaatcgaattattgattgtgtcataaagacccttatgtccacgtcctaatcgaccccccggaggagtatgtgcttctaaaagatctttgatactgtgcccaataccgaagttagttctatacatatgaccagcaatgagaaaaataaatgcaatagctaaatgatgatgagcaatatcggtcagccataaactttgcgtttgtggatggaatcccccaagaagggttagaatggcagttcccgccccttgggcggtcccaaataaatgattactcgaatcagggttttgggcataaagattccactgacccgtaagaaggggtcccaacccttggggatacggtaatacgtctaagaaattattccatctgacgtactctcccctggatccgggaatagcgacatgaactaaatgtcctgtccaagccaaggaacttactccgaaaagtcctgacaaatgatgattgagacgagattcagcatttttgaaccacgaaacgcttggtttccatttgggttgtaggtgtaaccaacccgctattaaagatatagcagaaagaaataatagaaaaagagctcctgtataaagatcttcattggtgcgtaatccgattgtataccaccactgataaacaccggaataagcgatattcactgggccggtagcacctcctcgagtaaaggcttctacagctggttgaccaaaatgaggatcccaaattgcatgagcaattggtcttacatgtaaagggtcctgtatccatgactcaaaatttccttgccaagccacatgaaacagatttcctgacgtccacagaaaaattattgctaactgtccaaagtgagaagcaaaaatgttctgataaagacgttcttcagtaatatcatcatgactctcgaagtcatgtgcggtagcaataccaaaccaaatacgacgagtagtggggtcctgagctaagtcttggctaaacctcggaaatcttaattccataatgcctttcaaatcctcctagccatcaataagatagagccatgctgcgagttgtttcaggccctaaataaacacggacactcaaaaaatctgttgggcaggcagattcacatctcttacaacctacacagtcctcggttcttggcgcggaagctatttgcttggctttacatccgtcccaaggtatcatttccaatacatctgtggggcaagctcgtacacattgagtacaccctatacatgtatcataaatttttactgaatgtgacattggatctataaagttttgaattttgatctggtattatatattgtaatattgtagacaccagacgaaacagtggtttattaaaaacaatcaatatatttcttaaatcaatctgtttatgagaaaaggtcaagacactttgattttgtgtcatcattatttcaatataaataatagtatttcaattctatgatgattatcaatgacagatctcaacttaccctctatttttgtgcctttagtgggcctagtatttccggcaattgcaatggcttctttatctcttcatgtccaaaaaaataagattgtctagatccaatgggaccaaatcctatcaatttatttcaacactgtatcataatacagatattttttagtgcggtacgatatgtggatctttccacacacaaatgaaagaactgttatgtatgcggatacatgctatctgcataaatgtagctggttaaaaacggatcagtaaatatttttaaaagtcaatgtataaaggtaaagtcaaatttgggttattctctcaattccaatcgaatgcaactggatctagtatagtatgcgagatctaaaaacatatctctccgtggcacctgtgttaactactctatggtttgggtctttagcgggtctattgatagaaattaatcgtttattcccagatgccctgtcattcccctttttttaattattgatatgcgaagaaatgaagaaatataattacacatgacgtaactaaaacctcgcctctccctttcaattctttagaatagtaaggaaaaagtgtattgaacctcataaaaaatccggtagatccaagatcgaatttagggcgggctctacgaaatcatagcatagaaagaattaaatgaaatattgggatttaggatagaaataattgatagttagaaagaaattgtattacttaatttctatatatatactatattaatacataattaattagataaaattccaactatttattttttcttcttcttcggttcgaaaatataagacttaagttaagtcgatacaaaatctaaaggaggttcttatccatttgtagatggagcttcaacaacagctaggtctagggggaagttgtgagcattacgttcatgcattacttccataccaaggttagcacgattgatgatatcagcccaagtgttaataacacgaccctgactgtcaactacggattggttgaaattgaaaccatttaggttgaaagccatagtactaatacctaaagcagtgaaccagatacctactacaggccaagcagccaagaagaaatgtaaagaacgagaattgttgaaactagcatattggaagattaatcggccaaaataaccatgagcagctacaatattataagtttcttcctcttgaccgaatctgtaaccttcattagcagactcgttttcagtggtttccctgatcaaactagaggttaccaaggaaccatgcatagcactgaatagggagccgccgaatacaccagctacacctaacatgtgaaatggatgcataaggatgttgtgctctgcctggaatacaatcataaagttgaaagtaccggagattcctaaaggcataccatcagagaaacttccttgaccaataggatagatcaagaaaacagcagtagcagctgcaacaggagctgaatatgcaacagcaatccaaggacgcatacccagacggaaactaagttcccactcacgacccatgtaacaagctacaccaagtaagaagtgtagaacaattagctcataaggaccaccattgtataaccattcatcaacagatgctgcttcccagattgggtaaaaatgcaaacctatagctgcagaagtcggaataatggcaccagagataatattgtttccataaagtaaagaaccagaaacaggttcacgaataccatcaatatctactggaggagcagcaatgaaggcgataataaatacagaagttgcggtcaataaggtagggatcatcaaaacaccgaaccatccaatgtaaagacgattttcagtgctggttacccagttgcagaagcgaccccataggcttgtactttcgcgtctctctaaaattgcagtcatggtaagatcttggtttattcaattttcaaggactcccaagcacacgtattaactatagatgtgatagataatagaaggcttgttatttaacagtataacatggcttatacaccaatgtcaaccaatcttaacaaatgattaaatccacccaaagaatttgtaaatgaaatgagttcaaatattttattttccgtaatgggtttaccttggtatcgtgttcatactgtcgtattgaatgatcccggtcgattgctttctgttcatataatgcatacagctctagtttctggttgggctggttcaatggctttatatgaattagcggtttttgatccctctgatcccgttcttgatccgatgtggagacaaggtatgttcgttatacccttcatgactcgtttaggaataaccaattcgtggggtggttggagtatttcaggaggaactataacaaatccgggtatttggagttatgaaggtgtggcaggggcacacatagtgttttccggtttgtgcttcttggcagctatctggcattgggtatattgggacctagaaatattctgtgatgaacgtacgggaaaaccttctttggatttgcccaagatctttggaattcatttatttctctcaggggtagcttgctttggctttggcgcatttcatgtaacaggtttgtatggtcctggaatatgggtgtccgatccttatggactaactggaaaagtacaatctgtaaatccagcgtggggcgcggaaggttttgatccttttgttccgggaggaatagcctctcatcatattgcagcgggtacattgggcatattagcgggcctattccatcttagtgttcgtccgcctcaacgtctatacaaaggattacgtatgggcaatattgaaactgtactttccagtagtatcgctgctgttttttttgcagcttttgttgttgctggaactatgtggtatggttcagcaactaccccaatcgaattatttggtcctactcgttatcagtgggatcagggatactttcagcaagaaatatatcgaagagttagtgtcgggctagccgaaaatcttagtttatcggaagcttggtctaaaattcccgaaaaattagctttttatgattatattggtaataatccggcaaaggggggattattcagagcaggctcaatggacaatggggatggaattgctgttggatggttaggacaccccgtctttagagataaagacgggcgcgagctttttgtacgtcgtatgcctactttttttgaaacatttccggtagttttggtagatgaagacggaattgtgagagctgatgttccttttagaagggcagaatcaaagtatagtgttgaacaagtaggtgttactgttgagttctatggtggcgaacttaatggagtaagttattctgatcctgctactgtgaaaaaatatgctagacgtgcccaattaggtgaaatttttgaattagatcgggctactttgaaatccgatggtgtttttcgtagcagtccaaggggttggttcacttttgggcatgctacgtttgctttgctcttctttttcggacacatttggcatggcgctagaaccttgttcagagatgtttttgctggtattgatccagatttggatgctcaagtggaatttggagcattccaaaaacttggagatccaactacaaggagacaagtagtctgatacgacattgttgtggtatctttcgcctctatttttttgacattgggtatcagagaaatcttgacttgaatcaccttctttgactttttttctttatatgatatggtaaatgatcccaaatgaataggtgtggaagctataattgtaaaccacgatcgaatccatgaaaaccttatattccctgaggaggttctaccacgtggaaacgctctttaatggaactttagctttagctggtcgtgaccaagaaaccaccggtttcgcttggtgggccggtaatgccagacttatcaatttgtccggtaaactacttggagctcacgtagcccatgccggattaatcgtattctgggccggagcaatgaacctttttgaagtggctcatttcgtaccagagaaacccatgtatgaacaagggttgattttacttccgcacctagcaaccctaggttggggggtaggtccggggggggaagttatagacacctttccatactttgtatctggagtacttcacttaatttcctctgcagtcttaggctttggcggtatttatcatgcgcttctcggacctgagactcttgaagaatcttttccattcttcggttatgtatggaaagatagaaataaaatgactacaattttgggtattcacttaattttgttaggtataggtgcttttcttctagtactcaaggctctttattttgggggcgtatacgatacctgggccccaggggggggagatgtaagaaaaattaccaacttgacccttagccccagtgttatatttggttatttactaaaatctccttttgggggagaaggctggattgttagtgtggacgatttagaagatataattgggggacatgtatggttaggttccatttgtatacttggtggaatttggcatatcttaaccaaacccttcgcatgggctcgacgtgcatttgtatggtctggggaggcttacttgtcttatagtttaggtgctttatctgtctttggtttcatcgcttgttgtttcgtctggttcaataataccgcctatcctagtgagttttacggacccaccgggccagaagcttctcaagctcaagcatttacttttctagttagagaccaacgtcttggagctaatgtgggatccgcccaaggacctactggtttaggtaaatatctaatgcgttccccgactggagaggttatttttggaggagaaactatgcgtttttgggatcttcgtgctccctggttagaacctctaaggggtcccaatggtttggacttgagtagactgaaaaaagacatacaaccttggcaagaacgacgttcagcagaatatatgactcatgctcctttaggttctttaaattccgtgggtggcgtagctactgagatcaatgcggtcaattatgtctctcctagaagttggttagcaacctctcattttgttctaggattcttcttttttgtggggcatttgtggcatgcgggaagggcccgcgcagctgcagcagggtttgaaaaaggaatcgatcgtgatttggaacctgttctttccatgacccctcttaactgagattttcttatttatatctattctattgctcttttatgttctggctcggctaggtggaatagccgagccattcctttttatgaaggataaggggccaggccaaacaaataaagaaaaaatttattcaataaacaannnnnnnnnnnnnnnnnnnnnnnnnnnnnnnnnnnnnnnnnnnnnnnnnnnnnnnnnnnnnnnnnnnnnnnnnnnnnnnnnnnnnnnnnnnnnnnnnnnnnnnnnnnnnnnnnnnnnnnnnnnnnnnnnnnnnnnnnnnnnnnnnnnnnnnnnnnnnnnnnnnnnnnnnnnnnnnnnnnnnnnnnnnnnnnnnnnnnnnnnnnnnnnnnnnnnnnnnnnnnnnnnnnnnnnnnnnnnnnnnnnnnnnnnnnnnnnnnnnnnnnnnnnnnnnnnnnnnnnnnnnnnnnnnnnnnnnnnnnnnnnnnnnnnnnnnnnnnnnnnnnnnnnnnnnnnnnnnnnnnnnnnnnnnnnnnnnnnnnnnnnnnnnnnnnnnnnnnnnnnnnnnnnnnnnnnnnnnnnnnnnnnnnnnnnnnnnnnnnnnnnnnnnnnnnnnnnnnnnnnnnnnnnnnnnnnnnnnnnnnnnnnnnnnnnnnnnnnnnnnnnnnnnnnnnnnnnnnnnnnnnnnnnnnnnnnnnnnnnnnnnnnnnnnnnnnnnnnnnnnnnnnnnnnnnnnnnnnnnnnnnnnnnnnnnnnnnnnnnnnnnnnnnnnnnnnnnnnnnnnnnnnnnnnnnnnnnnnnnnnnnnnnnnnnnnnnnnnnnnnnnnnnnnnnnnnnnnnnnnnnnnnnnnnnnnnnnnnnnnnnnnnnnnnnnnnnnnnnnnnnnnnnnnnnnnnnnnnnnnnnnnnnnnnnnnnnnnnnnnnnnnnnnnnnnnnnnnnnnnnnnnnnnnnnnnnnnnnnnnnnnnnnnnnnnnnnnnnnnnnnnnnnnnnnnnnnnnnnnnnnnnnnnnnnnnnnnnnnnnnnnnnnnnnnnnnnnnnnnnnnnnnnnnnnnnnnnnnnnnnnnnnnnnnnnnnnnnnnnnnnnnnnnnnnnnnnnnnnnnnnnnnnnnnnnnnnnnnnnnnnnnnnnnnnnnnnnnnnnnnnnnnnnnnnnnnnnnnnnnnnnnnnnnnnnnnnnnnnnnnnnnnnnnnnnnnnnnnnnnnnnnnnnnnnnnnnnnnnnnnnnnnnnnnnnnnnnnnnnnnnctaaaaggatctactaaattcatcgagttgttccaaagaatcgaaacggccggttatcaatggaatcccttgtcggctttccgtgaaatactcatttggccgaggacttccaaacacgtcgtaagctaaacccgtactgacgaataaccaacccgcaatgaatagggaaggtatagtaatgctatgaataacccagtatcgaatactggtaataatatcagcaaaagaacgttctcccgtgcttccagacatgctgagctccacaaatttttgtacattcaaaagggggaatcgattccgtgaaagatgggatcagtaaatagaaaactactgatattgcatctttgtgagatcgtcaattttgtaccaaaggtgtatttcgagtataccgaatcagtatagctatccttcctctggcacagcaacgcagccttgatcggtaccgaaatgctacacaattcttcttttttgctccttatctatggataccttatgttatgctattcaatagatcaacctccaatttattataaggtttcctggcttcataaaagtaaggatcttgggaaaatgtaagtcaatgatcaatgggttctaataattcatgaaagatattattgtattgacacaattcaattatatgtgaaatctaacctggttaaattatttgaatcctttcatttcaagtaattggttggatatagtatctactatatcatatttaatgaaagaaagaaaacatagttgtaacaatcaatattcgcgatgcaatcattgttggattaggtccaagacaaagattctttcttgaccaaactacaagtatggaactccatgatatggaaagacagaatatagaacctaaaaggataattgaggcgactcgtcttcgaatcgactttgtacccgaaaaagagaacaataaagtcaaagtttttgtttctcaatagatcctttcgatggatggtggaacacctatgttatgtcactctatctttttttagtattatctataatgaccaatgaatcatgaattttccattggaactaaactaattctcttaattggtttttattaccctcatatctggatggaaacttaggtaagtgttttatcaacatatgtagaaaaaaacatatctaataaagccctttttatcgttggatgaactgcattgctgatattgaccccaagaaagaaacggtaggtacagctagtccgtgaacagccaaccagcgcactgtaaaaattggataagttcgatctatggtcatatggctacacaaaccgttgaggatagttctagatctgggccaagacgaactactgtaggggatttattgaaaccattgaattcggaatatggtaaagtagctccgggatgggggactacaccacttatgggggtcgcaatggctctatttgcgatattcctatctattattttggaaatttataattcttccgttttactggatggaatttcaatgagttaggtttataagaactatgaagtcctagtctttcaatcaaagaaaaattactttagacttggatttctagaccattctattctggtagttcgaccgtggaatttatttgttttggtatttccggaatatgcttactctcaaactctttgtttacacagtagtgatattctttgtttccctttttatctttggattcttatctaatgatccaggacgcaatcctggacgtgaggaataatttatattttgctttttaaaaaattaattcttaataatcttaggatgagattcgaattcaaaagtgatcagagaaagctagagagatgaacccaacccagaatatgaaccgtaaaagaaaacacctattaaaccgatcacaagaataccagttacagtacctatcagccaaagaggaatccttccagtagtatcggccatttcatcaagtggtcatgctagagacaaaaacagtcatggataattatgaggatggtatctatccgaatgggataagagaattactactattctctttctttctcatgcttaatatctttagtttaatcgatatctgtcttaattctgtcctttattcgagtagttttttcttcgccaaattgcccgaagcttatgccattttcaatccaatcgtagatgttatgcctgtcatacctgtactcttttttctcttagcctttgtttggcaagccgctgtaagttttcgatgaaatctttaatactctgctaagatgtatttgataaaaaattctaaaaattgatcagataagtcttacattacgaaccctcgattcaaaaatagaaattcttgtatattgaatgaataaccgcagcgatgaatttggatcagccttttccccgttctgaccttccggtgagtatggactattaggtactccacctaattattgatatgacaagaaatctcgggtaacgaatgaatcaaaatcttattacaaataaatttttatggggtgtcaacaaaaatggtatatgtggtaaaaataggcaatctattccccttaaaaaaaatgatcttggagattgtgtatcaattgaagaaataattggaaaataaaacagcgagtacaaaaatgagtaataaaccccagtatagactggtacgattcaattcaacattttgttcattcgggtttgattgtgtcatagttctctaatttgaattaggtttaatcattttgactgactgtttttacgtagataataagtaaaaaagcagtaggaactagaatgaacagtgcagtagcaataaatgcgagaatattgacttccataatttcattattttttttcttcgcaataactcgggatgtaatcccatagagatgataaatttaactcctgtaaattcattgggatgaattgatcctgatgatactgaataggatcaatattatgaataacaatatctgatctatcaaatcgattcatcgtcgagaattgaatagtataacatgggaagatcctttatccatactaattacgaaatgggattttttattggatcaggaatcccattggatttttcatcctcttacactttcttttctataacctactgtcttccttatcttatctccttcctgtgtattatacttacaattatgaggtattatatgaccggtattctatgggtcacacacagacccaaacgaggtgagatgaaaaatcaaatattccaacaaatttactgaaaagggtccttgctcggtcttttattagtatcctctttcttgtatttatttgtactggaatgcatacatcaaaacgatgtctgcaatttgaatgagatagattgtttacaattagtcattgaggatacacaaactcagaactagaaaaggtgtggtttaacctgaaatctgtcgaggtaattatggtaagttcattgtccatcgtacaataaacgaataccatttttgtatgtactcccggtaaaataggatcactctactccgatcaagaacaatctaaaaagtaagacgaggatgtcttaaaatactgaatatagtaataccaccgattgtactaatgtacatatgatatgtctctcctttatcaatcgggagtagtggaaagatttgaaattcccatatccgaaataaaaaacaaaagaaggatccccactggggattagatcttgcttcctgtccccttttccgtgaaaagagagagatgaattgagaaattcaccggatcctagtctagtccccgtgttcttcgaatggatctcttagttgttgagagggttgcccaaaagcggtatataaggcgtacccagtaaagcttacaagtaaaccagatatggagatggcgactagggttgctgtttccatttttagtcaagatcacaatggatctacgataagatcgtttatttacaactacaacggaatagtatacaaagtcaacagatctcaaccaatcattaaataggatttatggaagcattggtttatacattccttttagtctcgactttagggataatttttttcgctatcttttttcgagaaccacctaaggttccgactaaaaaaatgctttcgaaataatttaattgaagtaatgagcctcccaatatgggaggctcattacttcaaatgactattgctttccaattagctgtttttgcattaattgcgacttcatcagtcttactgattagtgtacccgttgtatttgcttcttctgatggttggtcaagtaacaagaatgttgtattttccggtacatcattatggattggattagtctttttggtagctattcttaattctctcatttcttaaacttatttggtatttccccgatccaaaaacaaaaatgtcactccttaagattcaatctgagttctgaaaaaattctatttcaatgttattaattggatctaataaataagattatcctaatctatcttaattggaatcttactttattctggccctgcacaaatatgatccagccgcatatatgatatatgtcatatatgtgtggacatatacgtgcgtatcaggaagtaagaaaatatgtcaccacaaacagagactaaagcaagtgctggatttaaagctggtgttaaagattacaaattgacttattatactcctgactacgaaaccaaagatactgatatcttggcagcattccgagtaactcctcaacccggggttccgcctgaagaagcaggggctgcggtagcagccgagtcttctactggtacatggacaactgtttggactgatggacttactagtcttgatcgttacaaaggaagatgctaccacatcgagcccgttgtcggggaggaaaatcaatatattgcttatgtagcttatcctttagacctctttgaagaaggttctgttactaacatgtttacttccattgtaggtaatgtatttggtttcaaagccctacgagctctacgtctggaggatctgcgaattcccccttcttattcaaaaactttccaaggcccgcctcatggcatccaagttgaaagagataagttgaacaagtatggtcgtcctctattgggatgtactattaaaccaaaattgggattatccgcaaagaactacggtagagcggtttatgaatgtctacgcggtgggcttgattttaccaaagatgatgaaaacgtgaactcacaaccatttatgcgttggagagaccgtttcgtattttgtgccgaagcaatttataaagcgcaggcggaaacgggtgaaatcaaaggacattacttaaatgctactgcgggtacatgtgaagaaatgatcaaaagggccgtatttgccagagaattgggagttcctatcgtaatgcatgactacttaactgggggattcactgcaaatactagtttgtctcattattgccgcgacaatggcctacttcttcacatccatcgcgcaatgcatgcagttattgatagacagaaaaatcatggtatgcattttcgtgtactagctaaagcattacgtatgtctggtggagatcatattcacgctggtacagtagtaggtaaactggaaggggaacgtgagatgactttgggttttgttgatttattacgtgatgattttattgaaaaagaccgtagtcgtggtatctttttcactcaagattgggtctctatgccaggtgttatacctgtggcttcagggggtattcatgtttggcatatgcctgctcttaccgaaatctttggagatgattccgtactacagtttggtggaggaactttagggcacccttggggaaatgcacctggtgcagtagctaatcgggtggctttagaagcgtgtgtacaagctcgtaatgaaggacgtgatcttgcccgtgaaggtgctgaaattatccgtgaagcttgcaaatggagccctgagctagccgctgcttgtgaagtatggaaagagattaaattcgacttcgaaccggtagataagctagataaagcgaaatagaaagataaaaaaaagcgtgtagaatttagtaattcctctttgttctcctaattgattgcgattaaactcggcccaatcttttactaaaaaaaggattgagccgaataaagaatgagcttcctaacatactatatatttgcatatatctttcatatcatatgtacagacctatatatatatacaagatctaaatagaacgaagacgaaacaactcgatatttctatttttttttggatccaatccataattaatcctcaaagctaagggaagggctccctctatccaatctactcatcctgtatattgtctttttcgttccatgttgcaatatttattatttgattatacgagaacgaattccttatttataaactattttcgatgagaatttgtttttaattaaaaaaagagatctttctcacttattattagttaacaatcctaatcctcatatgcttaattctgataggaaataaaatagtaaaataattattcatcgaatgactattcatctattgtattttcatcaaatagggggcagaagattatttataatacttcgggagctaatgaaactattttagtaaaatttaactgtctcaattctcgagcgatcgcaccaaaaactcgagttccttttggatttccttcttgatcaatgacaactgcagcattgtcgtcatatcgtattatcataccgttttcacgtttgagttctttacatgtacgtacaattacagctctaattacttctgatctttctagaggcatattgggaactgcttctttgattacagcaacaataacatcaccaatatgagcatatcggtgattaccagctcctatgattcgaatacacatcaattttcgagctccgctgttatccgctacattcaaaagggtctgaggttgaatcatatcatttttatttcaatctgttatttcaatgcaaaagtatgaaagaaaaagaaatattgtccgtccagaaataaataaacctgcgttttttcatccccaataccctttttgtttagttctatcctgaaataataaattgagttcgtataggcattttgcgcgcagctattgcgatagctgctctagctacagtttctgatactccacccatttcataaagtattcgaccccgtttaacaacggatacccaatattcaggggatcccttccccgaacccatacgtgtttccgcgggtcttactgtaacaggtttgtcgggaaatatacgtacccatatttttccaccacgacgcacatatcgtgtcattgctcttcgccctgcttctatttgtctagctgtaatccaagcaggttcaagtgcctgaagagcgtatctgccgaaacaaatatgattgcctcgacaagatattcctttcattcttcctctatgctgtttacgaaatctggttcttttgggactaagcatagcaattataccaaatgatcaatcgaatttttatttaaccttttagaattgttcttttttttaactgaaagagtttgtcattttttgttttatcactggacagaatgggaagacaaggttgaggttgtttcttaattccatctctactgcagaaccggacatgagagtttcttctcatccagctcctcgcgaatgaaaggattctaattcaaacacattaatagataatacaccttttattgatattttcttacaaaggaagatgtagatacaagatatacaatacagctagacgtgtgtgtacgtttatgtatctttaatgtaatgtttcttttatttttataacgaatcctttcctttttacctctatcgaataaggattgtaatccaataaatagaggtttcgcgggcgaatatttactctttcctgtttcattcgaaggttcaattcataacctctcagaataaatcaatttttcttggtccgttccgccatcccacccaatgaattattaggatttgttttcaatagaagcctatgcagtcacaggttctgtcgttcccatagcttctccattaatggttaggtccgaactttgcaatggagcttccaacaaaattcgttcccaagtcaatttcctcagtttttattaacctgaaggctctttattattttattctatttttgctttatcacactgccttttatgacgtgattcatagaccatacatattggaatcatatatcattgatatttttgttctttctttctatcatccttccatttatccacatccttttgctttacaacccataatcagatctattttttgttgaaagaatttcagttgctacaaccatatgatagatccactcattcatatagtgactgtttcttgggatctcgacaatacgaagcaataagttggttattagtttattttatataattacaaagtttatagcggggtcagtttttaatcccaacttgaaactataaagaaaaaactaacgagtcacacttatttgataattttattggaaatcatgtaaagacaattcttatttgatatagctatttgcgcaagtattttacgattaagaagcaattgcttcttgtacagattgtgcattaatatactataactatagaataccttattctcacgagttactgcgtttatccgagtgatccacaaacgacgaaaatccctcttttgtctgcctctatctcgatgagaggaaaccaaagctctcattttctgttgagtaatcgttcgagtaagtcttgaatgagcccctctaaaggttgatgcaaataaacgaatttttgttcgacgtctccgagctgtatatcctcgtttaactctggtcattgaatcagattaaagcttaatgaataactaattgatttctcttctttcagtcatccttttccccttccccggtcattaataacaaaacggattattccaatatataaaatatcaattccaatggcttttgctactatgaccttcccaaccacgattttgtattctattccttccatttatttcactggaaataataaattagaatgatactatagtgggttccatcgtttctatggttacctcttaaacggtgaggtcctctctatacaccggagcctcttctttcatttaatcaaatttattgttaacttgtatagttcacactctttggctctacctatctacagtaatagctcttttcacaaaaagagttatccatacagtgacggcatttaattatgaaagttggctaggtagctgaccctgttagtccgtcaagaaaaggagcataacctttttttatgataccatttcctccgcttaatggataaccctttgctaccaatggggaattgcttcttatttcaaatctagatgattggatttgcaccaatggaaaccataaattccatatacagggtatatgatagatcttctctatctatcctagatactgaaaaaattgtctcatttgttcgaacttatgatctgaacgagtcgcacctaaatcttagattgatctaaaaatgatttagatttatcttttaatacaattgttatatgacatgtcggtctttttatcagataactacgtcctcgagcccgcggtcttaactttttcacaatagtactcctattggcttcggctttactaatgaatgaatcagcttccttcaaacccatattatgattagcatttgccgctgcagaataaaccaatttgagaatgggataagatgctcgataaggcatgagttccagtatcataagtgtttcctcataggaacgcccgcgaatctgatcaattactcttcgtgcttttaaaacagacatacatatatgttgagctaaaacttttacttcacctgaacttgagttctttatcatgtttgattcacatctatttttctatttaagttcttttctctataagaggtggaatagaataacccggttgaagcgtaatgatcatacgtctgtaatgcattgtatgtcccattcttctaccctttccgggtagtcgatgactattcatagctattaccttgacaccaaagaagagttcgacccaatgctttatttctgtcctagttgatccggattcgacattagaagtatattgattgttccccaataaccgaatacttttttctgtaaatactgcatatttgattccatccataaatccattttcttccctatgagttccagtatcgataagaattctagttcttactgttcatatgttatggtatgaatataccataccaattcgttatgtatggatgatgagattccattgatacagagtcaattccaatagacttattgaacgttcccattggcgctatttacggcgacgaagaataaaactatcactatattttttccttttcctacttcttcttccaagcgcaggataaccccaaggggttgtgggtttttttctcccaattggggctctcccttcaccgcccccatggggatggtctacagggttcataactacccctcttactacaggacgcttacctagccaacacttagatccggctctacccaaacttttttggttcaccccaacattacccacttgtccgactgttgctaagcagtttttggatatcaaacggacctccccagatggtaatcttaatgtggccgatttaccctcttttgcaatcagtttcgctacagcacctgctgctctagctaattgtccaccctttccacgtgtgatttctatgttatgtatggccgtgcctaagggcatatcggtcaaaggtagggcatttcccattgatataggaacttctgtaccagaaacaatggtatctccaattatagcccctctgggatgtaaaatatatctcttctcaccatccccatagtgtatgagacaaatgtatgcatttcgattagggtcgtattctatggttacgattctaccagatatgtctttttgattccgtcgaaaatcgattttacggtatagacgcttatgacctccccctctatgccttgcggtaatgattcctctggcattacgacctttaccacaacgatgccgtccatagatcaaattatttcgtggattggatttcacttgactgtctacggctccattgcgtgttctcggggtagaagttttgtataaatgtattttgcttgaagtagattcttcttttttatcaataaaaacctcttcccaaactgtacaagcttcttccaaagcatacggctttctagatgtatatgatgatatctagacagatggatcttatatgaatcgtatgatgaagtaccacatgagtggatatataggaatccaaatctgccgaatcactcatgttatgatcttctacatcctaggtctccccgttccgtcatctggcttatgttcttcatgtagcattcagaccgaatgactctatgaaattacgtcgatacttccacatattacgggtaacgtaggagacatctctatttttcccccgggggatcttaattaccactgcttagctttcaattcgcctctgaccatcaaattaaatgtgaataacccgccctcctctctttgaaatttgaaacaaggggcgcttccggttctgtgcgtgcttcaaacaattttgtcttctccatattaccatatctctagagtcaataattttctatgaggaactactgaactcaatcacttgctgccgttactcaacagttttctgttgaggtctatcccgtagaggtactcaaattggatcagtgatcgatttctaggtttcgtcgtaaacctaattggttacttccaattacgtaaatcaatagttcaaaccgcatggcagttccaaaaaaacgtacttctatgtcaaaaaaacgtattcgtagaaatatttggaagaaaaagggatatttagttgcagtaaaaactttttctttagcgaaatcggtttccaccgggcattctaaaagttttttgtgcgacaaacaaataataaatctcaattgatatagcctaaagaacctcaattttgtaagatgaatgttaactaatgtatttttctgtattgaatttctcagatatatagaataagagtgggttctaagtattattttttccctatcgcaattgtacttttcaaaagtacaattgtgatagagattgaaactcttttgttatatgcctatcccaaactaattggttttgtaaatggtaaattatatgcgcaataaagaatattaatactaattatttgtatttaatgatgaaattgtgatagatatgaaatcctagttattttttaaaaaatagagaaaaaactatggagttccaactgtttggggaaaacttagtttctagtacatgaaagttgattgttaaaaaacccacgctaggtaggaggattcaaatatttaaaccacaaaccagtctttattcattgattctaattaatgtttcctaaactatattgaatccttgaatctcgacgattcaacatgaattgactattattatttcaagtaaatggccaagggtaaagatgtcagagtcagagttattttggaatgtaccagttgtgtccgaaatagtgtcaataaggaatcgcggggcatttctagatatattactcaaaagaatcgccacaatacacccagtcgattagaattgcaaaaattttgtcgctattgtcataagcatacgattcatggggaaatcaagaaatagatcgaaggaaacatcatgtgttcgatctttccaaagaacaggacaggaagagtaataacttatataatatacataatatatacaaatcaaacccgattttatgtagatattctttcatgtgtatagatatataatgaaataatatatgaatcaatatttcggttggatccgaaatgaataaataatttagagataaggaataaaccttacccttgtctctgtttatgtttcggattggaacaaatcactataattcgtccacgcctgcgaatcagtcggcatttttcacaaattttacgaacggaagcccttattttcatattttttattccttaattctgaatccacttcttggaagagaataagtctcttgaattttttttaacttcaaattgtatacccatggttaaaaaaataacttaaaatgagaacttattcttgggtaaatcaattgcgaaatgcttatgtagagtgtccaatatttgttttacatcttccatgcgaaaatattcaattctcataagatcttctttactgttactcaaaaggtccaataatgtatgtatattggaccttttgagacaattataggtcctggaaggcaattctaattggtcaataaaaatacaattcaatggaattccttttttgtttttctttagattagttaatctatcttgaaaggttaaaaagggtagagtaaacctgtttttattttcttcgaaattaatgtcctcttcctccgcatatagaaaaggaataaataaatcaatcaaattacgagaagcctcataaagtgcttccttaggagttaaacttccatttgtccatatttctataaaaagtatctcttgtttttcattcccataagaatgaatactatgattcgcatttcgaacaggcatgaatacagcatctatagggtaacttccatcttgagagtcatttatgggttccatacgatatccgcgatctctcttgatttgtaatccaatacacaaatcaattggttccgtcaggttagctatatgttgtgtcgtgtcaactatttctacggaaggtggtgagataatatcttgagcggttacgtatctaggaccccttacgcaaattgacgcgtctctaactccatagagattacttctcaatacaatttctttcaaattttgtaagatttcatgtactgattcctcaatacctactatcgtagaatattcatgtggcaccttcttagattttgcacgtgtgatacatgttccttctatttctccaagtaaggcccttcgcatggcaataccgatggtatcagcttgacctttcataagtggtgacagaatgaaacgaccataataaagacgcttactgtctactcttgattcaacacacttccactgtagtgttcgagtggatcctgctacttcctctcgaaccatactatactagtattattatttgatcatttatttctcttgaaatcggtttaattcttattttcaagcttccttcctattaatctggaagttcttctcagatacaaggaaatgattcagttctagagccaaagatcgtagttctcgaatgagcaatcgaaaagattcgggagcatcctcggggttaggtactcttcctccaataatcgtagcaccaagtacttcttgacgagctctaatatgatcagatttataagtaagcatctcttgtaaaatatgagcaacaccaaatccctctagagcccaaacttccatttctcctactcgttgtcccccctgcttggcccttcctctaaggggttgttgtgtaacaagtgcgtaatgtccactagaacgtccatggattttatcatcaacttgatgaattaattttaggatataggactttcctatttgaacaggttgttcaaacggatctcctgttcttccatcaaatattctgctttttccaggatactcgggttcaaatacccatggatttcttgtttgcttactggcttcatataattcggaaaacactagttttctcgaagcctcttgctcatatctctcatcaaagggtgctattctataatgtctctttagcagattccctgctaacccgagcgagcattcaaatatctgtcccacattcattcgtgagggtactcctaaggggttgaagaccatatcaacgggtgttccatcttgcaaatagggcatatcttgtctaggcaaaattttggaaatgatacctttattcccatgtcttccagctactttatcacctactttgatttcacgtttctgtaaaatatatacacaaatcatttctgaattataactggaaccaccctttttctggatccatctcacatcaataacgcgaccccttccacctataggtagttttagagaagtttcttttgcagtggatacctgcataccaagtatggctcgtaataatctatcctccggggaatacgaggattcgctcgctgtctgaggcgttaatttacctactaaaatatcacctgtttctacccaagatcccagcatcacaattccatttctgtccaaattgaggagtaaatgagcctccagatgcggtatttccttagtaattctttcagggccttggcttgtcacatgagtctgaatttcatattttcggatgtgaaaagaagtataaatatcttcatataccaggcgttcgctaattagtactgcatcttcagaattgtaaccctcccatggcatataagctactaatacgttttttcctaaagcgagttccccaccaactgtagccgcaccgtccgctaaaatttgtccttttttaatgcatttaccccgccgaacccgaggtttttgatgcatacaagtatttttgttggaacgttgatacataactaatggaatacttatagtgttcccattacttgataacatgatcttgtgagtatcagtataaatgacctttccttcgtgttcggctataacagaaacccccgaatctagagccgtttggcgttccagcccagttcctacaatgcacttctcggaccgagaaagcggaactgcttggcgctgcatattagaactcattaaagcccgattcgcatcattatgctcgataaaaggaatgagggaagctccaatagaaaaatattggaaaggaaaaatgcttctaagatgaatctgttcccatgcaatagtcaggaattcttgacggtatcgagctggaacaacctgttcttcctgaataccccgattcaaggccaaagaatttcctgctgctaacatataatattcatctctatttggtgataaataaaccatctgtggctctttttctttttgtttctcagatatttcataaaacggactctctatagatccccaatgaccaatcctcacatgaatagctaaggatccaataagtccaacattgattccttcggacgtgtcaattggacaaatacgtccatagtggctaggatggatatctcgtatacgaaaactagcagttcgccccgtcaatcctccaggacccaaataactcaattttcgtccatgaacaatttgtgtcaatggattagttcgatccaaaacttgagataaagggtgtaggccaaaaaacgattcataagtagttgttaatgaagttgaagttaccaaattttgaggagtcggtatcaatttatgcctaattgctccacatatagttcctctaaccgcattttctaaacgaacaagagccaatccgaattgatcctgtaacagatctgctacagaacgaatacgtttatttttcaagtgattcatatcgtcaaatgtacccattccaaatttcattccgatcaaatgatccgcagcaaccaatacatctcgtggtaacaaaaatgtattgttctgaggtatatcaagattcagtctccggttcatatttcgtcgaccgatccttcctaattcacatctttgttgaaaaaatttcttttgtaattccttacataaagactcagaaaataccggatccccacctacacaagcaaattgttgataaaactccaaaatagcattttcttttgacccaatcttttttttctctttatcattcgggaaagacaagaaaatctcagggtaacaaacattatctagaatttctcttagatttgaacccatagctgatgatagaactagaatagatattttttgtttcctactcacacgggcccatatccttgcttttctatcaatttcgaattccaatcttcctccccaatctgatattatagtgctggtatagacagaaattccgttatggtccaattccgaacggtagtaaatacctggactttgcaatatttgattgatcacaattcggtatattccatttactatagaggttcctagggaattcattagaggaatgtttccaataaaaacggtttgttcttgcatatctttactggttttccaaattaatcccgcgggtacatataattccgaaaaatatgtgagtgattcatacacagcatctctttcttttatcaagggttctaccaattgatatctttccacaaataatttaaattcaatttcttgatctgtatcttcaatttttggaaacttatgaaattcttccgtcaagccttgattaatgaacctacaaaatccctcaaattggatctgactaaatccaggtattgtggacattccctcatttccattccggagcatcttaatcttatttaatcccattattgactcactattcatcgacccgtatggatcgatctagcaatgatggaatgtatattctgtttactgaatcacatgaaattttagtgaactccatatatgtatttcatacgtatgaatggaaatgaattgataaaacattcctgaaacaaattctgtcacttacacttatgaagtcttgtatagatacaatataaaaatggatccaatttccacctataataatgatattacgatcagattgggtaccaaggattcacgattaaatctgctttctatgaatgagataaagacagaataatcaggagcactatccagttttactttactttatttagatttagcacacttaatttaatgaaaattcaaagattttttgatagtagtctaatttatagactaggattgaattgcataatcataaaaggagtagtattttttattcaatatattcaatacaatgaaagattaaagcacggtgattctctatagggtacataagagagagacccgactcggtatctgtgtaccaatttctgttctggggtttacatataatatatattttcttaaaagttaatggttccaatttgccccaaattttgcagtctgtgactggaaatccatttttttttcaatttgaaatagtagaaaagggaagtttttaagcggtgtgtgttttgagatacaatcaatcgaagagactaaactagatcccataagaaacaagaattcatttttggaaagagattgaaaagggataagcacaagagattcaagatcaaaaaaaaataaaaaagaaaaattaatattttcatcgattttggatcggatttttagatagtgtatgagtaggcccgacaaaacccttgtatggcttcttctatttctcgataaaaagaaatatgaccaagagtggttcgaatatatatacaacggatttctttttttacacttcctactattagatagtgcccataaatctcatgataagtacccaaggattcatattgaacttcgatgggaacttctcttgacccaatgacacgttgatctagtctccatcggagccacaaaggactatctaaactgattcgtttccgccgataagctccaagtgcgtcataggaactacaaaaatacggttctttctctttcgtatacttataattattattgtcaactgtttgatatggattatacctatttgcacaaatacctcgacgattcccgatcgttaatacatagagtccgataagtatatcttgggttggtacggaaatgggatctccaatagctggagacaagagattcatatgagaaaacataagtaaacgggcctccgcttgagcttccaaagataaaggtacatgaacagccatttgatccccatcaaagtctgcattgaagcccttacaaactaatgggtgtaaacaaatagcacgtccctccactaaaatgggttggaacgcctgtatgcctaatctatgcagggtgggtgctctattcaacaatacaggatgcccctgcataacttcttgcagtatttcccatacaatcggttctttttcccgaattttacttttagcaatccctatgttagaaacaccatgttgcctgattagaccacgaattacaaatgtttggaaaagttctattgctatttctccaggtaatccgcattgatgtaatgaaagcgaaggacccacaacaatgacggaacgccccgaataatcgacccgtttaccaagcagagtctcacgaaaccttccctctttcccttcaattacatccgaaaatgacttgtaaactttattatgaccatccctcattggttgcccgcggatcccattatcaagaagtgtatccacggcttcttgtactaatttctcctgacacattactaattcccctggtgtagatctacttgttgctaatagatcggtaagagtattgttccgatagataactcgtctatagagttcattaatatccgaactcattagtttccctccatctatctgaatgattggtctcaattcaggaggaagaactggtaataagcacaaaaccatccgttccggttctacttttgttcgaataaaatgtttagctaattccatgcgtctaaccaaaaaatcctttcttcttctaattttgctatcttcccattcatttccagtagacccttcgtctcctaattctttccattctgccaatgaattatctataataattcgaaaatccgaatcagctaattgttctctgatagcacctgctcctgtagagatttctcgatttcgaaatatctcgaagcctgaggtagtaaaaaaaagtgggatactgtatttccgggattggatttcatattcgaatgaacctcgtaatcgtaagaaagtaggttttttagctatgggcctagcaaaagagaaatcgcagtatactaggccttctaattgcttaaggggtttatctaaaagattcgcgatataactaggaagacgtttcaaataccacacatgagtcactggacatgcgagtttgatgtatcccatttgatatcttcgtatccgagagtcaacaaattctaccccgcattgttcacaaaatttagggtcttctttttcagctctgatcgctcgataatttccacaagcacaaattccactttttatgggtccagagattctttcgcaaaacaatccatctttttctggtttattggttttataatgaaaagtatagggttttgtcacctctccaactatctctccattaggtaggattttgttggcccaagcctttatttgttgaggagaaactggtccaattcgaagttgttgatgtttatactggtcgatcatagaatagaaatgatgattcattcagatataggatcggattccccccctttaaaatcggacgtgaaggtttcctctcatccggctcaagtagttacaccaaataaaaaaggaggtgaaactttgttcaaaaccctacaaagagctactccttactcaagttcccagtaagaaccaacctttcattgattcattcttcttttatgacaaaatggaaatgtgaaattattgagtagtctattccccgatgaattttttaaaagaaatactttggaattcgtaagggatttatttgtctatatatcgttccattcgatcttttaggtctctactcacctcgatggttatgccatgatgtcccttgaagcatatatgcgatagatagactcctgtaaccatgctatatttgcttgcttaaacagaatttctctctaaaagaaatggaatggctaattccacgaaaaaatcttttttcacgaggtataactggctattttatacggaatcgaccatggatcaattcccctttcatttggaagtattgaatacacccataattctattctgagtttcatgttattttttccaaaacacatgtcagagtcgggggcatcccaatcggattgaatgggatgacagtttatcaatccgaatctgtaaaatgaaaatcttgatcaaatcaaaattgtttgtcgcgtaagaacaaaataattctctatggtgtaacaaaatatctctcatttccaactcgaatagattttttctttttatttccaaataaatgtttttgtcttgccttgaacggtgcacaaatttttggaatccggtaccaacaggtataatcccccccagaacaacgttctctttcaggcctttcaaccaatcaatacgacctcgtagagcagcttttgctaaaactcgagcggtttcttgaaaacttgcttcggatatgaaactttgagtattcagagacgctcttgttattcccaatgagattgcccgataacagatcgattcgtccaaagcgcgccctgctcgttccgctcgcaacaatccgattaattctccaggcgaaaaaacattagacattccatcttctgaaaccaacacttttgatgttacttgacgtacaataatctctatatgtctattatggatctgtaccccctgggatcgataaaccttttggatcttattaaccaaagagatacgactttgggctatggttagctcagctccaatcaaaaacccccaggggatcccaagaattcttggtatacgctcgttccaaccttcaactctcctttcgaggttcatcgatattgaatcaatcgaacgcacttctaagatttgttctacttttggaagaccttgcgttatgtcaccagatctcgatttttcatatataaatgtaactaatgtatctccttcgtaaaggattttcccataatgaccatgaacagttgctccaggagtagccaaataagacttagccgatcttataactaaaaagtcgacattaacaattaaaatttgaccagatttttttacgtgtgattcgtatttaaatagacatacattttcacaaataaattgtccaaggctaattttgatcgatgtctcttcacaataatcatgatggagaaagcaccaattcaaatggaatgggttcaaaatgatgttactgcatagatcgggattataaatccttctattttcatctattaaacagtatttaagtacttgaagtacttggaaaatctgtttcaaattgtcaagtagcaaatatttctttaacacgatctgattatgagttattaaatggtaagatgaataaaaatttgatattttaggtacaatagcgcctaagggacctaaataatccctaattggaattaggggatccgattcttttgtcacattgttatatttcgaactattgaatggaccaattcgagaacaattggatgatgacaaaattagcaaagattggcattccttatttcgattcaacaacataccaatagttccttgatgttggctaagtgattgaatcttcgccttggaataaaaaggattgatattggtgcaatctaatccattatcgggaatcaatccggaacttgccctatcatacctttttccggtatacgaaatagtggactcaattcttatgaaatcgcgaattagatcatttgcccttacctcaacaaaggaagcatgaacctcttctatagaaccattttgttcttggtcccaattcaatactaagcaagtccgaactaattgaatacttgtgtgataaattcctcgaattgacttgccatttccataaaggatataattgacaactctaaattggacattatccttttcctgcaagatatcacgagggaaaagtgttgctaaatttatcccatcggatatttcatatgtgactacgggtcgaaccaaaacaaaatactttttcttggtaggtgcgatccgttgaacatagatccaatttttccatttttttgattctttagaattttttttttccgtttctggtggtataaaaatgccgctgtgcctggatatcttatctgtctctccaggaaaatgaatatctccagaaaagattttaagttcaatatattttttttttctctccactcgtactaatccgcctactcggcttcttgtatttaaagccagtcgtgtatatactccaatgatactattgttccggaccattattaatgaggatcccggtaagatatgcacttcttcgagaatgaaaaaaaaccgatctactttcatttggtatttcggactaaattcttttgctcctcgatactcaatcaaatcttctttttttatgattgaatccacctctatggtcccatatttagtaattccggaactgcttcttctgtatcgtggatcatcaaaataagcaagaatactatttccacgtaaaataccatttatgggtatttcaatcgaaataccaaaacagggtattagttcttgatcatattgtaatgggatgatgaatctatttcttcgcttttttgccaagaaatcaggattctcttggagaatagaaggatatattaaattccaatgatcattggatatgattcgatcaagtcttgaatagtcaagaatttccctatcttttttaccagaagtatctaacaatttatgtcttacttgatcattagtcattgaggggttatagatatatctttcttcaacagaaagagaataaacattcatttgatcttgatccttgtggagcgaaaaagacactatactggatctgcacgggcctcctgctaatatccataaatggcttgtttttggtaatagatgaacattaccatatgtatattcaggtgcatggtagacatcggtactccagtgcatttctccctctgattcagaataaatatgttttcgtaccctctctttaaaatgaaaagtggacgttccagcacgaatctcagcaatcacttgttctgattctacatattgatcattttgaactaaaatcaaactttttggcggaatagtcacattatatataatatcccgactctcaatagttacatacaagtctatagaacatagaaaagcaggatacccatgacgggtacgtgtgggatgaaccaaatcctcattgaattttatttttccattagaaggagctcgtacatgtttggcagtaccgcctgtgaatactccgccggtatgaaaagttcttaatgttagttgagtccccggttcgccaattgattgacccgcaataatacctacggcttctcccaattcgaccaaatcgccatgagtgggactacgaccataacataattgacagatccaagatgtactcctgcaagtaaagggggttctaatatatattggttgtgctcgaaaggttatgaatcgattgacaagtccaatcccaatatcttgatttcgagtggcaatgcatcgtagaccgatatatatatcgtctgctaatacacgaccaattagtgtttggacaaaaattttttccgtcatctcattttgaggactcacggagatacctcggatagtaccacaatctcttctacgtacaataatgtgttgaactacttcaacaagtctacgtgtaagatatccagcatctgatgttcgtatagcagtatctacaactcctttgcgggctccgtagcaggaaattatatattctgtcaaagaaagtccctcgcgtaaattgctttgaatgggtaaatcaatcatttgtccttggggatctgacattaatcctctcatacctactaattggtgtatctgagatgcatttcccctagctcccgaaaaagacattagatagactggattagagggatcagtcatccgaaaattaggattcatttcttgtctcaaatattcacttgtagcataccatatctcaatagattgacgtaatttttctaccgcatgtacattcccataatgatggtgtttctccaaaataaaactttgttgttcagcgtcttggactaaccatcccttagaaggtattgttaaaagatcatcaattcctaatgaaatagatgtaacagtggcttgatggaaacccaaagtctttacttgatccaggatatgtgatgtatatcccattccgaaatgatctattaatctgctaataagtcgtttcatagcagttccatttatcactttattgtgaaagaccagatcggcccgttctgccataagtacctctatattctgctgagtaggattcgacaatggacctgagtcagtgattcgaaatttcctcaatttttattcacgcagaaagaaaattggaaaacccgatttcttagtctacacacgtctttttttaggaggtcgacatccattatgtggcataggtgttacatcacgtacgaagcttaataatataccacttctacgaatggctcgtaatgctgcatctcttccgagaccaggaccctttatcataacttctgctcgttgcataccctgatcaaccactgtacgaatagcatttaccgctgtggcttgagcagcataaggtgttcctcttcttgtgcctttgaatccagaagtaccggcggaggcccaagaaactacccgacctcgtacatctgtaacagttataatggtattgttgaaactcgcttgaacatgaataacgccttttggtattctacgtccattcttacgtgaaccaatacgcccattcctacgtgaaccaattcttggtatagcttttgtcatattttatcatctcatatttatgagtcagaaatatacaaaaaaaaagatacggggatacccatttcatgttaaaacggatcctttaccttatttttacattttggaaagttctttttagaagaaatacaccctagtacatgttcctcgacgctgaggacatcctctaagagcgggagattttttaacatttcttattggctgtcttgtgtttctaataagttgtttaatagttggcatttattttggctttttaaccccatatttagaacgcccttgttgacgatcctttactccgacagcatctagggttcctcgaacaatgtgatatctcacaccgggtaaatccttaacccttcctcctcttactaatactacagaatgttcttgtaaattatggccaataccaggtatataagcagtgatttcaaatccagaggttaatcgtactctggcaactttacgtaaggcagagtttggttttttgggggtgatagtgtcgtatgtatatatatgtatatatagaataaaatgggttggtttagatcgatcttaacctgatgattgatcatcatgaattatttctattcaatatcaaatcacatttaattatggtttgaaataaaatggatggaaaagttgacagataagtcccccttactgtcactctacagaaccgtacatgagattttcacctcatacggctcctcgttcaattctttcgaagtaattggatccttttcttcgttcgagaatctcctcccttcctccactcaagggtaactaagaccaattcagtcacgttttcatgttccaattgaacactttccatttatgatcaaaggagatctttttaccaaacatatgcggatcaaatcacgatcttataattctaataagaacaagaaatctttctcgatatcaatccctttgcccctcattcttcgagaatcagaagttttcgagtttcaatttgttcattttgaatctgggctcttctatcttctacttatttttgatattgacttattttgctttattctttatttcatttcgattttttcctcttcctctatccctatcccataggtatagcgtttgaatcaatagagaaccttttcttctgtatgaatcgatattattacattccaatttcttcccgatacctcccgaaattgacgggttagtgtgagcttatccatgcggttatgcactcttcaaataggaatcaatttgatgaaagatcctggctttcgtgctttggtgagtcgtccgagatcctttcgatgacctatgttgaagggatatctatatgatccgatcgattgcgtaaggcccgcggtagcaatagaaccggggaaagtatacagaaaagacagttctcttctattatattagtattagttagtgatcccggctctgtgagtcctttcttccgtgatgaactgttggcaccagtcctacattttttctctgtggaccgaggagaaggggggctcagcgggaagagggttgtaccatgagagaagcaaggaggtcaacctgcttcaaatatacaacatggattctggcaatgcaatggagttggaccctcatgtcgatccgaatgaatcagtctttctacagaggtaaatctttgcctgctaggcaagaggatagcaagttacaaattctgtctcggtaggacatgtatttctattactatgaaattcatatttcttgtatgtgttcctaagaaaaggaatttgtccatttcatttttcggggtctcaaaaaaagggcgtggaaacacataggaactcttgaatggaaattgaaaagaaatgtagctccagttccttcggaaatggtaagatctttggcgcaagaagaaggggtgatccatatcatcttgacttggttctgcttcccctctttttttaaccgagtcgggttcttctcctaccagtatcgaatagaacatgctgaacaaaatcttcttcatgtaaaacctgctcgatttagatcgggaaaatcgtacggattttatgaaaccatgtgctatggctcgaatccgtagtcaatcctatttccgataggagcagttgacaattgaatcccattattttactatccataatagtgcgaaaagaaggcccggctccaagttgttcaggaatagtggcgttgagtttctcgaccctttgccttaggatttgttaattctatttctcgatgggatggggatagggtggatctcgaaagatatgaaagatctccctccaagccgtacatacgactttcatcgaatacggctttccacagaattctatatgtatctatgagatcgagtatggaattctgtttactcactttaaattgagtatccgtttccctccttttcctgctaggattggaaatcctgtattttacatatccatacgatcgagtccttaggtttccgaaatagtgtaatgtaaaaagaagtgcttcgaatcattgctatttgactcggacctgttcttaaaaagtcgaggtatttcgaattgtttgttgacacggacaaagtaagggaaaacctctgaattcaatattggaccttggacatataatagttccgaatcgaatctctttagaaagaagatcttttgtctcatggtagcctgctccagtccccttacgaaactttcgttattgggttagccatacacttcacatgtttctagcgattcacatggcatcatcaaatgatacaagtcttggataagaatctacaacgcacttaccaacttgatcttgttgcgcccggtaacaaacatgcataaaccatttctcgaagtatgtgtccggatagaccaaagtctcgatagttagctctaggtcttccagtcaaaaaacaacgtcgatgaagacgtataggtgcactattacgtggtggggattgcaattttccatgaatttcccatttttcactcaacgatgaaactttgcttattttttcttttgaggatcggcgaatcaaatgatatttctgttccaaatttttccgcttgttctctctctgaatcaaactttttcttgccataatgttcagttcctattattatcacagttcggatcctccctctccatcgaaacaaatgaaattgttgctgatacagtacataaaaaaacaataaataactaaattaacgagtttttggctctcgaatatccaactgaccaattaatttcttataacgtactctatttttctttgacaaataagccagcaaccgttgacgttttcctagaatttttcgtagacctctttgtgataaaaaatcttttttgtgcaattccaaatgtgaagtaagtctccgtatcttattggtgaaactgaatacttgaaattcaacagaacccttgttttcttctttttcttcttgtggaataatggaaatgaatgaatttttgaccataaaaaatttttctatccttttttttctttttcatggatttttacgatcaggaattatgccggttattttaatctagtacacacacaaatttggatttattcatatttctttattttatccaaatcaaattttcaatttgatttggatagaaagggataatatgtttccacattaacgaaagaaaagaatttatttctatctaaaaggattcccctaatttatttattcctatatccattcaatctgtcatacatctttcggctttcatataccgaaaatgtcacggaatatagatatgatataggaaatatactattaaactcaaagttctttctttaaagaattctgccttctttaaaatatcataaacagttcctgtaggttgagcacctttttcaaggaaatatagaatagcaggaacatttaaataagtttgattctttatcggatcgtaaaaacctacttttcgaagatctcttccttctcttcggaatcgaacatcaattgcaacgattcgatagatggcacgttgctttctaccacatcgtttcaaacgaagttttaccataacattcctctaattgaaattgcaaagcggtatggaattgattcaatataaaatcatgaatagtcattggttcaaccggtatataatatttctatctacggataaataagtatttatccggataagggtcagcaaggatcaaatttatttaatttaaccccatatcatatgaattgaatgaaaagatattcaattttacccacatttgacacttgattccgtttgtgagaaaccaaacgaattctcagatatgattcttagaaccattctgaaaattaataagatttttcccttaacaaatttttgtttcatgtggaatgcagtgtgatcccatctttcgtttcatgaaaaacgatattgggatagatgtaaataaacaatgccccccctagaaacgtataggaggttttttcctcatacggctcgagaaaaatgattccaatttctgtatataatagtggatccataaaataatgaattttactataatttgaattgagtacttttttttcttacttacagaaaaaaagaaatctcattcatactcataactcaagttgggtaattctgacaagaaaatccttagacatttattgagccgtctctaaactcttttgtttgtctcatttcgaatctatttttattcctcagcctgatccaattgttgagacaattgaaaatagtgtttccttgtttcggaatcctttatccttgctttgtgaaatcattgggtttagacattacctcggggatccttattcctttaaaaatggcagcaacataccttttttgttatttctttctatataaatagaaaatgattgattcccttgtgatacacttttcatcgaaatagttttaccaaaaatttgacttttctctgaatttgaacctttcgatttagaatttatatatacttacagagttggtctaacttattgattttcactaaccctagattctttcccttgaaaaatgaatcaatcctttcttctcgagcttcatcatgtactatttacttataacccaacacaaattaggttccgggcagaacaaactatgtcgagccaagagcatcttcattactatagaagatggcagatgtaaaaatccacagccgaccatgtccttcaagtcgcatggataaatccaagcaaccttttcgtaaatacaagcgatcttttcgtaggcgtttgcccccaattggatcgggggatcgaatcgattatagaaacatgagtttaattagtcgatttattagtgaacaaggaaaaatattatctagacgagtgaatagattgaccttgaaacaacaacgattaattactattgctataaaacaagctcgtattttatctttgttaccttttcttaataatgagaaacaatttgagagagctgagtcgatcccaagaactactggtcctagaaccagaaataaataggcatactcctcaattgactcaaaaatccaattggaactcaagctcagattgatttttgttcgaaaaggcctagaatcctgatttgattcttgtgttataatataagaaacaaaaatgggggagaagaagaaatatttttttattgaaacatgttcgttcatttatttatttttattctatatcttcccggagttcattctccggggaattccctttcaatcattcctgtatattacttttgaattccctttaacgacgagatttattatcgtttcttgcatgtctcgcgaaagtcagagtaggcgcgaattctcccaatttgtgacctaccatacgatctgttatataaataggtaaatgttcctttccattatgaatagcgattgtatggccaatcattgtgggtataatggtagatgcccgagaccaagttactattatttctttctcctccctcatgttgagtttttccatttttcccgctaaatgattagctacaaaaggatttttttttagtgaacgtgtcacagctgattactccttttttttacattttaaagattggcattctatgtccaatatctcgatctaagtatggaggtcagaataaatacagatgaatggaaaaaagagaaatttagctagataagatcaacgatttcttatatagctagaacgaccttcacaaattgcggatactaatttgttaagaatcaatcgaattgaagctatagcgtcatcgttggctggaatcgaaatatctgcaagatctgggtcacaatttgtatcgattaaacaaatcgttggaatccccaaaatggcacattctcgaagagccgtatattcttcttgctgatcaacgatgatcacaatatcaggcaatcccgtcatatatttgatcccaccgagatatgtttgcaaggtagataattttctcttcaacattgctgcatctcttttggggagacggttgagtttccccatcttttcttctgctcttaagtccctgaacttataaagtctcgtttccgtagtggaccaattcgttaacataccaccgagccatttttgattaataaaatgagaccgagcccttattgcagctgatgctactgaatccgatgctttatttttggtaccgacgattaagaagttttttcccctacttgctgcatcaaaaactaaatcacaggcttctgataaaaaacgagcagttctagcgagatttgtaatatgaatacctttacgctttgcagaaatgtaaggggccattctaggattccatttcttagtaccatgaccaaaatgaactcctgcttccatcatctcttccaaattgatgttccaatatcttcttgtcattttttcccacacttcctttttgttttttctttttcgtattattaacaaagagacgaggtaccttgaaataaaagattgttccgatggaaccttctaccggggattgaccattgatacacggcgcaaaccataaatttttttaattacttattttatttattaccaaatcaataatcagaccagtatagttaaaagatagttaaagtgaaaatgaatctgctcttattcctcaaatatccaaatttttatacctaatactccataaatagttcgaattgtataggaacaatgatcaattttagcgcgaattgtttgtaggggaactctaccctctctgatccattcaacacgtgcaatttcttttccgtcgatacggcctgctatttgcacttgaattccttttgtatccgtttgttcagttaattcaatagcttttttcattgcttttcgaaacgaaactctattttttaattgtaaagctatatattctgcaagaatattaggttgtccataaggtttttcaactcttgtgatagcaatgttgagtctccggtttacagaattaaactccttttgtacattcatctgtaattcttcgattcctcgtgtttgcccctctattaataaatttgggaatccaatatagattctgacttggatcaaatcgatttttttttgaattgttatacgtgcaattccttcgaaacctgaggatattttcctatttttttgtacatagttcttgatacaattccgtatttttgcatcttcctgtaggtccccggaataattttttggttgtgcgaaccaaaaggaatgatgactttgagttgtaccaagtctgaaaccaagtggatttattttttgtcccatatttttctattcgattttttcatccatatattttatatgaattcaagtctgacgagaataatattctacgactaacaactcatttatttgcaaaccgatccatttactatctattatttgatttactaatcctttatattggaatgagtcaatagtcaaatgttttggcaattcctcatgggcggatgaagcaatataattttgaatcagaccttttgatctttggttatccttcgcagtaataatatctcggggtttgcaacgataacttggtatatccactatacgaccattaactaaaatatgtctatggttaaccaattgcctggctccggggatggtcgaagccatacccaatcgaaagaggatgttatccaaacgcatttcaagtagttgtagtaaaacctgacccgttgaccctttggcttttccagcgatatgtacatatctaagtaattgtcgctctgtcagaccataatgaaaacgcaatttctgtttttcttctaaacgaatacgatattgcgattttttcccagaacgcaattggtttttaagatcacttccggatctaggtcttttactagttagtcctggtaaagctcccagacggcgtatttttttaaaacgaggtcctcggtaacgagacataaagactccttttttattttattgaaatttttacacaataaatttaaactgaactaaaggataaacaaagcaaaatcgaaaaatgaattgtatcaacatctagattttatatatatagtgaggctccgtttgatgatttgttctgtagagatctaattgctctaatcacgacataatagatcgctgatccagcattttatttgaagaaaaggagagattatcaatctcggaaaaatagatagagaaattaacgaaaatgtgcaaaagctctatttgcctctgccattctatgagtctcttcctttttgcgtatggcatcgccactccctttggcagcatctactaattcggaacttaatttgaaagccatatttcgacccggacgctttcgggatgctcctaataaccaacgaatggcaagtgcttttccttgtgtagatcctatttcaataggaacttgatgagtcgatccgcctacacgtcttgcttttactgctatatcgggagttactccacgtattgcttgacgtaaaacagatagtggatttgtttctgtcttttgttgaatctttttcacggctcgatagataatttgataagccaatgatttttttccgtgtttcataatacggttaaccaacatgttaactaatcgattacgataaattggatcggattttggagttttttcttctgcagtacctcgacgtgacatgagcgtgaaagaggttcaagaatcagttttctttttataagggctaaaaacgaatcacttaccatatataacacaaaatttctcctccaattctttctagtcgagcttctcgatctgttattatacctcgagaagtagaaagaattacaattcccattccacctaaaatcttaggaattcgttgatagttggaataaattcgtaagccgggccggctgatacgctttaaaatgattctagttttatatattccttttctggtttttctatgtcgcagagttgaaaccaagaaatatttgttactctcctgatgtttccgaacgttttcaataaaaccttctcgtagaagtattttaacaatgttttcggtgatatttgtagatgctattcgaacccttccttttttatccgtgtcagcatttcttatagaagttattagatcggcaatagtgtccctacccatgacgaactagaattataggttcccccaaattttgatataatcaacatgtttcctataaagtatatacgtgagacacaatctactacctactatatcatagtctcatctactactattctcatggagagttcgatcctggctcaggatgaacgctggcggcatgcttaacacatgcaagtcggacgggaagtggtgtttccagtggcggacgggtgagtaacgcgtaagaacctgcccttgggaggggaacaacaactggaaacggttgctaataccccgtaggctgaggagcaaaaggaggaatccgcccgaggaggggctcgcgtctgattagctagttggtgaggcaatagcttaccaaggcgatgatcagtagctggtccgagaggatgatcagccacactgggactgagacacggcccagactcctacgggaggcagcagtggggaattttccgcaatgggcgaaagcctgacggagcaatgccgcgtggaggtagaaggcccacgggtcgtgaacttcttttctcggagaagaagcaatgacggtatctgaggaataagcatcggctaactctgtgccagcagccgcggtaagacagaggatgcaagcgttatccggaatgattgggcgtaaagcgtctgtaggtggcttttcaagtccgccgtcaaatcccagggctcaaccctggacaggcggtggaaactaccaagctggagtacggtaggggcagagggaatttccggtggagcggtgaaatgcgtagagatcggaaagaacaccaacggcgaaagcactctgctgggccgacactgacactgagagacgaaagctaggggagcaaatgggattagataccccagtagtcctagccgtaaacgatggatactaggcgctgtgcgtatcgacccgtgcagtgctgtagctaacgcgttaagtatcccgcctggggagtacgttcgcaagaatgaaactcaaaggaattgacgggggcccgcacaagcggtggagcatgtggtttaattcgatgcaaagcgaagaaccttaccagggcttgacatgccgtgaatcctcttgaaagagaggggtgccttcgggaacgcggacacaggtggtgcatggctgtcgtcagctcgtgccgtaaggtgttgggttaagtcccgcaacgagcgcaaccctcgtgtttagttgccaccattgagtttggaaccctgaacagactgccggtgataagccggaggaaggtgaggatgacgtcaagtcatcatgccccttatgccctgggcgacacacgtgctacaatggacgggacaaagggtcgcgatcccgcgagggtgagctaactccaaaaacccgtcctcagttcggattgcaggctgcaactcgcctgcatgaagccggaatcgctagtaatcgccggtcagccatacggcggtgaattcgttcccgggccttgtacacaccgcccgtcacactatgggagctggccatgcccgaagtcgttaccttaaccgcaaggagggggatgccgaaggcggggctagtgactggagtgaagtcgtaacaaggtagccgtactggaaggtgcggctggatcacctccttttcagggagagctaatgcttatgcttgttgggtattttggtttgacactgcttcacacccaaaaagaagcgagctacgtctgagctaagcttggatattgaagtcttctttcgtttctcgacggtgaagtaagaccaagctcatgagcttattatcctaggtcggaacaagttagttgataggatccccttttttacgtccccatgtcgccacacgggagggacatggggacgtaaaaaggaaagagagggatggggtttttctcgcttttggcatagcaggcctcccattgggaggcccacacgactcaaacgaggaaaggcttacggtggatacctaggcacccagagacgaggaagggcgtagcaagcgacgaaatgcttcggggagttgaaaataagcatagatccggagattcccaaataggtcaacctttcgaactgctgctgaatccatgggcaggcaagagacaacctggcgaactgaaacatcttagtagccagaggaaaagaaagcaaaagcgattcccgtagtagcggcgagcgaaatgggagcagcctaaaccgtgaaaacggggttgtgggagagcaatacaagcgtcgtgctgctaggcgaagcggtggagtgctgcaccctagatggctagagtccagtagccgaaagcatcactagcttacgctctgacccgagtagcatggggcacgtggaatcccgtgtgaatcagcaaggaccaccttgcaaggctaaatactcctgggtgaccgatagcgaagtagtaccgtgagggaaaggtgaaaagaacccccatcggggagtgaaatagaacatgaaaccgtgagctcccaagcagtgggaggagaaagtgatctctgaccgcgtgcctgttgaagaatgagccggcgactcataggcagtggcttggttaagggaacccaccggagccgtagcgaaagcgagtcttcatagggcgattgtcactgcttatggacccgaacctgggtgatctatccatgaccaggatgaagcttgggtgaaactaagtggaggtccgaaccgactgatgttgaagaatcagcggatgagttgtggttaggggtgaaatgccactcgaacccagagctagctggttctccccgaaatgcgttgaggcgcagcagttgactggacatctaggggtaaagcactgtttcggtgcgggccgcgagagcggtaccaaatcgaggcaaactctgaatactagatatgacccaaaaataacaggggtcaaggtcagccagtgagacgatgggggataagcttcatcgtcgagagggaaacagcccggatcaccagctaaggcccctaaatgaccgctcagtgataaaggaggtaggggtgcagagacagccaggaggtttgcctagaagcagccacccttgaaagagtgcgtaatagctcactgatcgagcgctcttgcgccgaagatgaacggggctaagcgatctgccgaagctgtgggatgtcaaaatgcatcggtaggggagcgttccgccttagagggaagcaaccgcgaaagcgggggtcgacgaagcggaagcgagaatgtcggcttgagtaacgcaaacattggtgagaatccaatgccccgaaaacctaagggttcctccgcaaggttcgtccacggagggtgagtcagggcctaagatcaggccgaaaggcgtagtcgatggacaacaggtgaatattcctgtactaccccttgttggtcccgagggacggaggaggctaggttagccgaaagatggttatcggttcaaggacgcaaggtgaccttgctttttcagggtaagaaggggtagagaaaatgcctcgagccgatgtccgagtaccaggcgctacggcgctgaagtaacccatgccatactcccaggaaaagctcgaacgaccttcaacaaaagggtacctgtacccgaaaccgacacaggtgggtaggtagagaatacctaggggcgcgagacaactctctctaaggaactcggcaaaatagccccgtaacttcgggagaaggggtgcctcctcacaaagggggtcgcagtgaccaggcccgggcgactgtttaccaaaaacacaggtctccgcaaagtcgtaagaccatgtatgggggctgacgcctgcccagtgccggaaggtcaaggaagttggtgacctgatgacaggggagccggcgaccgaagccccggtgaacggcggccgtaactataacggtcctaaggtagcgaaattccttgtcgggtaagttccgacccgcacgaaaggcgtaacgatctgggcactgtctcggagagaggctcggtgaaatagacatgtctgtgaagatgcggactacctgcacctggacagaaagaccctatgaagctttactgttccctgggattggctttgggcctttcctgcgcagcttaggtggaaggcgaagaaggcccccttccgggggggcccgagccatcagtgagataccactctggaagagctagaattctaaccttgtgtcaggacctacgggccaagggacagtctcaggtagacagtttctatggggcgtaggcctcccaaaaggtaacggaggcgtgcaaaggtttcctcgggccggacggagattggccctcgagtgcaaaggcagaagggagcttgactgcaagactcacccgtcgagcagggacgaaagtcggccttagtgatccgacggtgccgagtggaagggccgtcgctcaacggataaaagttactctagggataacaggctgatcttccccaagagttcacatcgacgggaaggtttggcacctcgatgtcggctcttcgccacctggggctgtagtatgttccaagggttgggctgttcgcccattaaagcggtacgtgagctgggttcagaacgtcgtgagacagttcggtccatatccggtgcgggcgttagagcattgagaggacctttccctagtacgagaggaccgggaaggacgcacctctggtgtaccagttatcgtgcccacggtaaacgctgggtagccaagtgcggagcggataactgctgaaagcatctaagtagtaagcccaccccaagatgagtgctctcctattccgacttccccagagcctccggtagcacagccgagaagcgacgggttctctgcccctgcggggatggagcgacagaagtattgagaatccaagataaggtcacggcgagacgagccgtttatcattacgataggtgtcaagtggaagtgcagtgatgtatgcagctgaggcatcctaacagaccgagagatttgaaccttgttcctacatgacctgatcaattcgatcaggcactcgccatctattttcattgttcaactgtttgacaacatgaaaaaaccaaaagctctgctctccctctctatctatccaagggatggaagggcagaggcctttggtgtcccttccagtcaagaattggggcctcacaatcactagccaatatgcttttctctcatgcctttcttcgttcatggttcgatattctggtgtcctaggcgtagaggaaccacaccaatccatcccgaacttggtggttaaactctactgcggtgacgatactgtaggggaggtcctgcggaaaaatagctcgacgccagaatgataaaaagcttaacacctcttatttgactttgaaaatatttttcaaaaagataaaaatccaaaatgcaaaggtcgtcttattcaaaacctcaattatgcttctctcccacttcacacctcgaaacgcactgttcttgagaaggcgctttcacatcttcttaacccgaatgaggagaggaaaggttcctttttgagggtactcccgggaacagatccagtggagacggggtgggggatatagctcagttggtagagctccgctcttgcaaggcggatgtcagcggttcgagtccgcttatctccagcccgtgaacttagctgatactatgatagcaccgaatttttccaattcggcagttcgatctatgatttctcattcatggacgttgataagatccttccatttagcagcaccttaggatggcatagccttaacgttaatggcgaggttgggtcgttgcgattacgggttggatgtctaattgtccaggcggtaatgatagtatcttgtacctgaaccggtggctcactttttctaagtaatggggaagaggaccgaaacatgccactgaaagactctactgagacaaagggctgtcaagaacgtagaggaggtaggatgggcagttggtcagatctagtatggatcgtacatggacgatagttggagtcggcggctctcctaggattccctcatctgggatccctggggaagaggatcaagtttgcccttgcgaatagcttgatgcactatctcccttcaaccctttgagcgaaatgtggcaaaaggaaggaaaatccatggaccgaccccatcgtctccaccccgtaggaactacgagatcaccccaaggacgccttcggcatccaggggtcactgaccgaccatagaccctgttcaataagtggaacacattagctgtccgctctccggttgggcagtaagggtcggagaagggcaatcactcgttcttaaaaccagcattcttaagaccaaagagtcgggcggaaaaaaggggagagctccccgttcctggttctcctgtagctggattctccggaaccacaagaatccttagaatgggattccaactcagcaccttttgagattttgagaagagttgctctttggagagcacagtacgatgaaagttgtaagctgtgttcgggggggagttattgtctatcgttggcctctatggtagaatcagtcggggaggcctgagaggcggtggtttaccctgggcggcatggccaagcggtaaggcaggggactgcaaatcctttatccccagttcaaatctgggtgtcgcctatgactcgacaaattcttgccctgcataagcaaaggcaaaggacggggcttgtcgatacttgatttatagaatacatagttctataagttgttttctcaaaatctggctcttttgggttcggcccaaacagatataccaatagggatgaggtaaggcttacttattaattccagaactacgaaagtaagaggtcagaaatcttggtatccaaaggttcctaaaattccatttttgctcctaggattgaagaaaagattatacgaaagactatcaagacttcctaattatcttacactacctacactaagggtctactgactctgtctggtggaaaggactgaatttgtatccatacttacgatccgacaaacattcaatcaggatggttggtcggcccttatcttatagaataacagagtaaaaaaaaaaggattcctttgcaatttgcggtgtgtatcatatttttacttaatacttcccaatcagaaatcctataaagaatctgttacgagtggattcattgaattggttcatcaatagccttaagtcagaatcctattttgactctgcgccattgattctactatgattattgatcaataatggaataattccttcatagaaataggagatataattcaccgggactgacggggctcgaacccgcagcttccgccttgacagggcggtgctctgaccaattgaactacaatcccgcgaggtgtatggcacatattcttaatgtaatcataaattctagtcctagtcgtcgtattgtaagaaagacaggaatgatatactgatatcatatccacatataatatgggctatagcgagagtgacacggattactagtaaccaataattattttacggcaaaaaattgatcctttctctttatttctacggattaggcatttccgtttatggaagacaaattggttatattcatggagcggtgaattatacccccaggggaagtcgaatccccgctgcctccttgaaagagagatgtcctgaaccactagacgataggggcatatacgcccgaccatcatcatactatatagtatgagcagttttttggaattgtcaatatagtctaatggtatgaaatttattgttcctgaatgaactcctatgcatatacgtatatattattgcagtagactcataataaaaacggctaattcatgaattgaataaaacggtcgggatagctcagttggtagagcagaggactgaaaatcctcgtgtcaccagttcaaatctggttcctgacaagaaaaaatctattggatagatattcaaattaatagagaaggatcgggatacatattcgttaatagtctagagtatgatacatatttattcatctaggtatatagatatatacatccatttttataggtgggtaaaggtaaaaaagagatatatgtatggtaaagaacaaagtgagattacgttccctttcttcatttttgcttacgttactttccggtccatctaagtgatgtgcgcggtacaaagttcatggtgcaaaactcttttgattcatcctattttttctactcatacgaaagaaattaatatgatattttccaaattgaaattggggaatctcaatgaagtccttttttgcttaagctctccataataaaaatttgagtccagtagttattctgtttcatctagaactctggatataagtaaacatgagtttcttatcatttgcggagacaggatttgaacccgtgacctcaaggttatgagccttgtgagctaccaaactgctctactccgcgctaaagaactaggaactaatggacgaagaaagattggatacgcccctctaccatatctatacaaatagaatagtccatttatacagaatggtaaagaggcccttctatgatcatagatcatagagatctatacaaatatgaaacgatatttttatccgcggatatggtcgaatggtaaaatttctctttgccaaggagaagatgcgggttcgattcccgctatccgcccataattaagtaatgtactatgataaaaaaattaattgactactatactatactatatagttagtatagtagttctatcttacccctttctttgcctaacatccaaaaacaaaagcgggtatagtttagtggtaaaaccctagccttccaagctaacgatgcgggttcgattcccgctacccgctccatattctttattatacatgcgtcatcaatttggatatgcatcctttttcccgaaaagatatattttctgtataatcttatttgagcaagagttaagaatacgaaagaagaaaaatgaaaagtgtgattcgttctattaacaacttaaatagttaaggggtccatcggtttcaaactccgatcaaaaactttatttcttaaaagggtttaatccttttcttctcaatagcatatttgaggaaaaatatacgttctctcgatttgtatccaaaggccaattagaaattgcatcaaaaagttggattatggagtcgcgaagcataatttttgaattggattaactattccataagtataggtaaaggatctatggatgaagatacaaaagtatatttccaatcgtaactggatcttccatttttgtgttgtaaaaggaaatttaagccaaatagctaaaaaagatagttttggtttactagaaccatcagcatattgtttcagctcggtggaaaccaaattcttttcctgaggatctaggagtgaaaatagggaacgaagtaactagactagatagatttggtataatctctctcctctagagggatcatctagaaagcggtttagatgcattcatacagaaaagctgacatagatattatggatctcattttttctctggaaatacatggaccttccataaaggagccgaatgaaaccaaaatttcatgttcggttttgaattagagacgttaaaaatgatcaaccaacgtcgactataacgcggatgtagccaagtggatcaaggcagtggattgtgaatccaccatgcgcgggttcaattcccgtcgttcgccatcacattatttcaaattccaaaaatttgattttccatattcgggctattagctcagtggtagagcgcgcccctgataagggcgaggtctctggttcaagtccaggatggcccgctgcgccagggaaaagaatagaagaagcatctgactctttcatgcatactccacttggctcggtcgttgtgcctgggctgtgagggctctcagccacatggatagttcaatgtgctcatcagcgcctgacccggagatgtggatcatccaaggcacattagctgggttgcccgggactcgaacccggaactagtcggttaaaagccgagtactctaccgttgagttagcaacccggataaataggatatgtagatatgatcgaaatataaaaatcaattgagttgcactgcacgaccctatcaaaacattgaactagcaacacatcgaaaagaaggattttatgatcaattagaaacacaaaataccaaaattatgacagaccacccattttttatcaaattcttttttgattttccctaagaaaatacatcttgtatgagagtaaatgcagcaaggcaaacctttgagtgaaggaaacaaaaaaaccaatatgggtggggataaacagccctatttatctacgatcgaattatatttgttcgatacaccattgtcaatataaatgttgagaaagtaaatcaagtaaataaaatcttgtgttggattggcacaactaagaaattggaatgaaaaattaaggtaaataaaaaatcccggtttattcaatcaataaaagtaagcaagcttaaccccttgtttgttgttaaattcaattcccccaccaaaatatgaataaagcaagaaaaaggaaaatggtgtgtaaatagaaataattacacaaggatagatactagttacccttccctactttattttatatggagtagataatttttccttgttgcaatagaggaaaaagatccctccccaaaccgtgcttgcatttttcattgcacacgactttccctatgtatacatatcaaatttacatcccgagaagaaagtctacgaagactactcagttgcttcaaccactacatgagcatttcagaatataaatttatgaatatatttagagatctattctctatatgggtctcatgaccaattatgccttgaagaggactcgaacctccacgctctttagcacgagattttgagtctcgcgtgtctaccatttcaccatcaaggcatcttgaaagtgaatcgtattccatgaatatgatatctatctaatgtgatatatggaatatatgacaaaggtggagtgttggagtatttctatcgatcggtcatataggcctgagtcagacatcaaattgcttcgatttgaattatccggaggataccttatatatatcaaaaagatgtacaatcaaacctatttctcgattcaatagaagcccaaagaagtgaatatggtacccaaataacgataggatagatatgtcaaaagcgggtctgattacgcctattcctaatcctaaatagaatgtaaggacgtagggatccatatgtaaacatagtctctatttacatacgcttgaatgaccccttctcataataagaatgtacataaccctattccggtctggtccggtatggaatgaacttataatctgatgatcgagtcgattccatgattataagttcataaccccattttgggcggaacagatctactaattcttttattccagttagtaagagggatcttgaactaagaaatagacctagaagggggatatggcgaaattggtagacgctacggacttaaaatccgtcgactttataaatcgtgagggttcaagtccctctatccccaataaaaagccattttactttctaactatttgttttttcatcaatgaaaacaattcactatctttctcattcattctactctttcacaaatggatccaacagaaatctttggatcttatacaaatgaagatatataggcaatctctattattaaataattcacaatccatatatttactaggtcaaattttttgttttagtccctaaagtactctactaggatgatgcacaaaaaatggattggattgagccttggtatggaaacctgctaagtggtaacttccaaattcagagaaaccctggaattaaaaatgggcaatcctgagccaaatcttttgggaaaacaagggtataaaactagaataaaaaaggataggtgcagagactcaatggaagccgttctaacgaatagagttgactacgttgcgtcggtagctggagtccctctatcgaaattagagaaaggccatatatacctgtactatacatactgacatatcaaacgattaatcacgacacgtataatatatttatattatttatgaaatcatgaaattcggaaaaattcagagttattgtgaatccattccaatcgaacaaaaatcgactattcagtaataaaatcattcattccagagtttgatagatcttttgaaaaattgattaatcggacgagaataaagagagagtcccattctacatgtcaataccgacaacaatgaaatttttagtaagagggccgccgtggtgaaatcggtagacacgctgctcttaggaagcagtgctaaagcatctcggttcgagtccgagtggcggcatcttctaaaagagatacaatagatcctaaaatgtattcaattcgcgatttccaattctgtaatgggacccctttctccccaagtaggattcgaacctacgaccagtcagttaacagccaaccgctctaccactgagctactgaggaacaacgggattcgacctcatagagttcaactcccgttctcaacccatgaacaatatgagtccgaagcttccttcgtaactcccggaacttcttcgtagtggctctgttccatgcctcatttcatagggaaccccaaagtggctctatttcattatattccatctatatcccaattacattcatttaatatccctttggtcttattgacataagagatgtcatttatagtctatctctttctatatatggaaagttaagaaatcatcatataataatcgagaaattgcaatagaaaagaaaaagggaggtttgtgtagggatgacaggatttgaacccgtgacattttgtacccaaaacaaacgcgctaccaagctgcgctacatcccttttaaaaattgttgtacagtgtcattgtacaaaatacctgtcttgttttccacatctttattttctcctctatctatatagaactttcttgtcatttcttgtttttggtttcatataataaattatatacatctgaaacccacctaacatataaaaaagaatgaatatttatccgtaatgctcaggtcatctttttcttttttagtgacaggaaaatctcatctattggttcattgtacatatccttgttaggaaatccgcgtaaaaaaaaaatgatcttctgggacggaaggattcgaacctccgaataacgggaccaaaacccgctgccttaccgcttggccacgccccattttgatttctattcgatattaatcaatactaatattggtattggttattcgtcaatcccaacccaaatacacaaaaacatatgggtattttgttgctaggattcaagacatgtagatatagaatcaaaaaaattcattgatcattacataaaattcaattaagatattgtatgaaagttgaattccttatattctcattttagaatgataatgggaggttatttgggaaagtccgaaccgaaaaaaagaaggatttcttgatctgcctttttcatttttccttataaaaataactcaaaattatctaatccacaagaacgaaatgcttgttgggcctgtagctcagaggattagagcacgtggctacgaaccacggtgtcgggggttcgaatccctcctcgcccacagccttcccaaaagggaaggccccttactttacctctgggggtaggaaaatcatgatcgggatagcggacgcaaagctattgaacttgggtcttttgtcgaaatggaatggtcttactttttatttatcgtgaatgattcgatcattacatatagtaaccgaggccggaatcagcatatttgtactccccgtaactcttcctcagccaggcttgggcagaatagcagagcaagcacaagtattattagtagcatagcaaaaatgcgttcctcgtcattaatatgtttgctcgcgtgtggcctatcgggagaatcgatgactgcatcaaagatgcactgctagtacatcatctgagaattcttaattggctatttacaattgacggcgattctcaaatatcgtagaacagaatgtgatacgatgagatagaatgcaatagaaacaaagatagggaacgggttacctactcctaacggtcaaagcgagccctttaattcaattcttcattctttaattaagaattaatcaaatgcgtccattgtctaatggataggacagaggtcttctaaacctttggtataggttcaaatcctattggacgcaatttttttccatctattttgttaaatgtctatactaagaaaccctttttgaatgattcaaatcagaaatttttctcaatgattactctcatgctaagaataagtatgacggaaagagagggattcgaaccctcggtacaaataatttgtacaacggattagcaatccgccgctttagtccactcagccatctctcctaattccaaatttaaaatttgttatgtgatgtaacacgtgaaataaagattgataaaaatccaccttcttcatttattttttcattatcttatttaacaaagcaattgatcaggaattcaatatagataatgactcaaaacggatctcctcaatagaaattctcccggcctgttaagtactggccgggacatttcttctttttctcagtttattacttaatttcttactgttgtcaagtaaggaataaaaaaagacatatgataactaaacaatattaaattacatttaacaattggaaatatttctattctacaagagacaaaatttgaacagttgagattcaattgacccgaattcataagatctgacaccggcagttgaaaagaaggtgaagggggtcgtgtatacagaatttatcatttttatagtctagcttcaatcaccccttcaggcgggaactattagtttagtaatgacttcccagcaaaagcttaacttttatttaaataagctttacatcgcttagccggcgagactaaatacgtgtcaacgctagaattttcatgattccactgctatcttgattttgtctcaccctcttttgttcgacaaatggtccattcatatacaataatgaatatgtaggagagatggccgagtggttcaaggcgtagcattggaactgctatgtaggcttttgtttaccgagggttcgaatccctctctttccgtttctgttaattcaccaatgttatcgaccacaatgtatcaaatcaaataacaatggaaaccattattcccgcaataagacccttatttgatagaaattctctattcctaagcggatggattaaggcccttagatctatttagttcggcgaaaagggggcaaaattctatgaacctttcttaattaggagagagagggattcgaacccccgatagttctttgtttagaactataccggttttcaagaccggagctatcaaccactcggccatctctcccaggggtaatctctattttattcctacgaatagaacatgaccatatgacacactaactatctgtagaaacatcccagatgcaaatccatatttcgatgtatctatgtatactgtatacatagatacatgatctagtatgtctgcttgtaaaataaagactaaactccccccgagttcatgtccgaataaaataaagtggtaataagttctaaagaatcaattgattcatgattaaatccctccatgatggattttattacaattttgactaagtgagggatcaaatgtatagttcatttgttggtagctcggaggattagaaacgcccttttaactcagtggtagagtaacgccatggtaaggcgtaagtcatcggttcaaatccgataaagggcttttttccactaaactcaagttttagccttcgtttttcagccattataacgaattcagattattctgactttaagttaggaagttgaacatttattgattagcaaataattgcacgtattaggagtagtccacctgtagtgacatagtagtcctcctcatgtctcattattcaaaaattgtcctgggacataacagaaatattctataatactctatatatacaattcctattattaatcgacaaaccaaggtgttcttatttctccaatgttcttattctaaataaagaaaaagtaagtggacctgacccattgaatgatgactatatcagctattctgatatttaaattcgatatagattaaattgtataagcggatttttttatttccttagaccgcgcaaagcaagaatttctcaatatttatggtttaatcttcttgttactggatgctccataggaataaatcgctattttttcttctacaaagtttatttcaaaatttttcaattccagagaacgaatgcgagaaaaggactttcattttcagtctaccattatttaatattcaattttgtggcaggaaaaagtaggaaattttattttggttaaaagatatactctgaaatatgagtcataggacaattcaggattcaaatggttatcaaatggttatatagtataagggctaatcaaatcgagctcatggatttacctaggttagtttgtggcccaatagaaaaagagtatattcgaaacccattgtaaaggggcattgaacgagaaattgtctatagataatcgaactatcgtatgccttggaaatgatatgaggtgttcggaaatggttgaagtaattgaataggaggatcactagccggctatcggagtcgaaccgatgaccatcgcattacaaatgcgatgctctaacctctgagctaagcgggctcgcataagaaaaaattgcgtaacaaatagaaatattgtataggaattccggaaaatgtcggtcttagatatgaactaaactaattaatagagttctgtagctaaagttctaagctaagttcttttataaatgaatgaatatcgaacgttacagtattacaaatcacactgtaaaaatgaaagggagagataaaatagatatgggatatatctattcatcttgaattgaaaatacatcaatgatagaattatttctgattgaaataaacaaggtttatccaatagaaatgaactgatataggatggaaaaaaagaaataaatgaaagagatggatgaaattataaatgtatcttgcaaagaaaagggaaaagggatataactcagcggtagagtgtcaccttgacgtggtggaagtcatcagttcgagcctgattatccctaaacccaatgtgagtttttctattttgacttgttcccccgccgtgatcgaacgagaatggataagaggcttgtgggattgacgtgatagggtagggatggctatattgctgggagcgaactccaggctaatatgaagcgcatggatacaaacaagttatgccttggaatgaaagacaattccgatttgtctacgaacaaggaagctataagtaatgcaactatgaatagggctatacggactcgaaccgtagaccttctcggtgtaaacgaggtgctctacctaactgagctatagcccttgtcatagatatcttaacatatagataatttcttgtcaagatggatattccctaatctcacatgataactctttgatccgcttactgttaacagattggtattgcttagaaataatattctatctataatccccgaggtgatgggtcttcttttgcggtgataaattgtaaaacagatcaaactaattattatcgaaatgattcgaactgtttcaaagacccaacatgcattttgttgcattgggctctttcatcaactgatgtaaagatcagttagtccaccatattttttctttacaagaagataatgagctggctccatgtgctctgattcattatttgtattcagatctagtagcaataccaaagtgtttcaaagaagggttaccttgacttaggtctgccttcggcttagatccacctaagttaaatggagtctctatcgttccgctgcaagagtcgaatatgagacttcatacaccttaaagttcataggatgaaaggaggttttttgaagcccttatactcattatgcctagcattgaatgggctgagtatttaccttatcaactatcaaatcaatgatgggttctatttgatttggcacctaaattcgcaccaaaacggaccaaaccaaatatttgtcaggctattgttctctcgaatctatggagtaagacttcaactatgttcattgcataataagctcccttgaaaagcattggcgcaccacgctctgtaggatttgaacctacgacatcgggttttggagacccgcgttctaccgaactgaactaagagcgctttcaaagaatttttttccataacacatctcacataaatatagtatccaaaaaagattatgccccgtcgatcccaattaatctttcgttactgcccataggagaagtaataggtgggccgagctggatttgaaccagcgtagacatattgccaacgaatttacagtccgtccccattaaccactcgggcatcgacccaggaagaattcattctagacttatctaatctatgatcaacttcctttcatagtaccccgtggaaacacttgtttattccatatgtatggatgatatgaactgcctaaacaagaacggcgaacaaccagagtactcactacatcaaacaatttccattaatgaaaccatgtaaatccatcggataatcaaagcatgtctgatgaaatggttgttgttatctgcttcaataacgaatcattggtttaactgaataacaaagaaaatagatagacccttctctttctcttcgtctcaggtcgatggatcttctcaacgggaagatctcccatatgcataatacacattccagttgaccgagcctcattctaattgttttgttccgaagcaaagatatccacggaggccggtttgtcctattcagatattcacgaccaggaggtactggattctctttcggataggccctgaaaggagaagaaaggctggaatgccaacagacgtctgtctattctctaattcacccgacccgatagtacccattttggtaacgtccagtgccaaagtcactgaatgggtaagtcgccaatccctaaaacggactactcattcattagatagagaagatcgccaagatttcgtgatccgctgccgaacctattccaattccaacagctcagactcggatcgtggggatcaccggaatacttcgtatcaacagataagatactcgatcaatattgattagatccgaaatccgttattgaattgctcattcaatgagcattctcaatattatctaaatcgtggatacatatgtatccttgacatactgaaacgactgccattattggtatcaaaccaatagcgattcatacaagctaaatcttctaatcggtaattgggccaaagaacaaatttgcatttaatgaatttatttgtatccgtattaagatgcttgtcctcatccaaaaattttacagagtttcttatgttattttcattgcaaaataatggatttctatccgtaacattccaattatgggaattgaaacaaattaacattctcaattctctgcgacgtctaggagatagaatattttcgggaacaaggaaatcataataatttgtgtccccattcacaagcatattcccatatcgtacaatgggtttatccaaattcctcttatcaatatatcttttttttatgcattttctattagtttggtgtttattgttctcgaccaatgaaatacttatagtttgatatataataaattttccatccctttttatagatagatgaattggttcgataattaatattcccttttttatcaattctgtaagagctagatctttctgaattagcattacatccagatgcatctctcctctttgaatcgaggatatagcaatttcttttggatttatcagtctaagtaagagacaatataccttgatattattgatcattctttggttcaaggagtcatcccatcttaattgaaaaaggaaatattttttcaggaagaaatctagttctgcttccttgtttttcttggattgttttttcttcttaccttttttaatggtaatgtctgatctcgcataattctcttcaatatctttttttttttttcgtagatctgatacaagatttacttggtcctgttgctcatttttttgttggttggaattttctaaaagaagtaatttgattggtataatccatggtttaatcttatatgcatcaaaaagtaagacaaattctgggaagaaccaagattctagatttgatatggtatgataaactctttcttgattcattcccatccaatttaaaaaaatacttttttgattggatgggttgatttcttgatgaatcataagagaaaaaatatctttttttttcaatttgttgttgatttttttttcagtcttagtatttttataaattttggtaccgatatgtgtattggtccaggtctcaatattgatattttttctaagacaaaaggggagaatttgacaatcaaaatattttctatctagattttgatgcgtatcaataatatatccttcttctagataatcactaatagctgtacttaccaatacataaaatgattcggattttggtttattgaaattatatggaattttgtgaaccccatttacttgtaatcttgacccataaatataagaatcctccttatccccatagtttatatatttatgtgataaaagatcatatctgtagtgttttttccgatgaatccgctttaattgagtccttattttgaatcctataacgttgattgattctatttcgccatttttgtggtactaaccgcgaccatttggtttgagataaattgtattgataatgcccctttaaccagtcaatcattccagacttatgaattttcttatgtcttgaattgtaatcaaatattcctcgtgtcatacaataattcttgattttatccttaataaaagggtaagtcccctgatattgaagtagagatttcaattgatacttgttaagtaattgggtttgtgataatttgtaaaatacatatgcttgggacaaggaggataagtcataatacatttttgtactattactattcgaaaaggacttttttatagtgtaaaaaaagttcattgtattttgatctctttcatcaattccttcctgatttgtttgatcgttgtaaacggatttattgattattttttttgttgattcaaagaaaagttggaaatagatcctgtgaatggtaatcatacatagcaagatatctatatatatattttcaatcagagatttcataaaataatgtgatttacgtaggaatatctgccaaatatgtttcttcgatcttttatcatcacaagttagaattattcttttcttgtcttttgtgattcgttctatttgattcctgattgtgattgttctatcagaaagatctttcatctttttttctatgagtgaataatttgtccaattcatggattggatttgaatggttgatttgtgaataatcttattatttatttcggaatcttttccattttcagccgtttcatatactttcaccctgctcaattcaaataagaatattgggtttactttttgaatttccttcattcttgttttttcttttgaaacttttagaagtagaaataaatcctttttaacttttctaacttttttttggagttccttataaatgggttcaaaaaaggaaggtcgttttcggggattcccaaaagggaattccgcttccattccccaaactgttaaaaaacaaaaatttaatttgatccctaggatgagatcgtattttagatcttcgccaaggtttcaaacagaaaggaaatagaatctttatctgaataccgtctgttaaccaatctttgggaaattctgtttctgataattgaacaccattataagtgcatttaacatgcatttctctattccactccttcaaatcctcataccattcggggaattggaataataacatacggccaatatttttagcaattatcaatgaaggcaatacaatgtgttttctaagaaaagattgggttactaacatcaaacctcttattgcttgaacaaatataatgctatcccaagtttctgatattactatacgttcattttcctcttttttttcttcgttttttttctctttttcccttgtctcttcctcctcaaaatcaaaatgtgaaatttctggttctctccccaacgaattcctaaaaatgagattcatcatttcagagatataaaaagaagaaaaaaaaaatgttttgtctattcgatccaaaaaaagcggagaatgcacatttgcttgaaacatttcccaaataaccgttttacgtctttgagcacgcatggatcctttgattatatcccgacgaaaatccgattgttgcgagtaacgtatcaaagccacctcttctgcttgatcactattattagtattatttgtagttataatagggttctgattgttatcagtataaattactacgcgtttagcttttcttgaacgaatttcatgatcttccttagattcttcctcctgttcttccaaatcatcagttaatttgtatgaccaccggggaacccttttacggatttcttctattccaatagatttatttctaattattgtttgatcgtttggatcagttgtaattacatcgaatacccattttaaagcttttgtttgattttctaaatcaattcttgtttgctctctcaatgcaggctcaaaaggaaattctttgattgaggttaaggaattaccaatataattcagtaatgattccctatcaggcggattcttttgatgttccaattttctggaataattagaattattaggaagtagcccataaatcttatttatccaattgatttctatggaatcctccgtatctgtagaagtgattaaatcattcatgatcatatgtgaatagaatttttttattgttccacgatatggtcccctcaaaaaggggtcatacgttttgggcaagtatttttgttcgttttcatcattgcaaaatctggtccttttttcgagcatatctagagcaagaaatcccttttctttttctagagcttttgttcgacttattaattcattgttcaagttgtactttttttgctcattggtataaacccaataatgatactgatccacatgggataatttttctgtcgtgtacaaagacatttttcgttctatcatttccgaaaaagtcgataaactaggtggatatgtaaaagatattatttgttttccatcacttggacatgtataaaaaaaatattgtgccatttcatttcgtacagcattttcaaatcgattattttttatataccgacatgggcgattccatcgtttataatcgaaATGATTTTAAAATCTTTTATACTAGGTAATCTATTATCCTTATGCATGAAGATAATAAATTCGGTCGTTGCGGTCGGACTCTATTATGGATTTCTGACCACATTCTCCATAGGGCCCTCTTATCTCTTCCTTCTCCGAGCTCGGGTTATGGAAGAAGGAACCGAGAAGGAGGTATCAGCAACAACTGGTTTTATTACGGGACAGCTatcaagaattctcactatttcttagattcatggatcaaattcgattcagtgggatctttcactcacatttttttccaccaagaacgttttatgaaactctttgaccccctaatttggagtatcctactttcacgtgattcacagggttcaacaagcaatcgatatttcacgatcaaaggtgtagtactgcttgtagtagcggtccttatatctcgtattaacaatcgaaagatggtcgaaagaaaaaatctctatttgatggggcttcctcctatacctatgaattccattggacccagaaatgagacattggaagaatctttttggtcttccaatatcaataggttgattgtttcgctcctgtatcttccaaaagggaaaaagatttctgagagttgtttcatggatccgcaagagattacttgggttctcccaataaataaaaagtgtatcatgcggagttcgcgatggtggaggaaccggatcggaaaaaagagggattttagttgtaagatatctaatgaaaccgtagctggaattgagatctcattcaaagagaaagatagcaaatatctggagtttctttttttatcctatatggatgatccgatccgcaaggaccatgattgggaattgtttgatcgtctttctccgaggaagaagcgaaacataatcaacttgaattcgggacagctattcgaaatcttagggaaagacttgatttgttatctcatgtctgcttttcgtgaaaaaagaccaattgaagggaagggtttcttcaaacagcaaggagctgaggcaactattcaatcaaatgatattgagcatgtttcccatctcttctcgagaaacaagtggggtatttctttgcaaaattgtgctcaatttcatatgtggcaattccgccaagatctcttcgttagttgggggaagaatcagcacgaattggatttgaacgtatcgagagagaatttgatttggttagacaatgtgtggttgggaaggttttttagcaaggtacggaatgtattgtcaaatattcaatatgattccattagaaatgaggattcagaatatcacacattgatcgatcaaacagagattcagcaactaaaagaaagatcgattctttgggatccttcttttcttcaaacggaacgaacagagatagaatcagatcgattcccgaaatgcctttttggatcttcctcaatgtcccggctattcacggaacgtgagaagcagatgaataatcatctgcttccggaagaaatcgaagaatttcttgggaatcctacaagtcgttcttttttctctgacagatggtcagaacttcatctgggttcgaatcctactgagaggtccactagagatcagagattttggaagaaaaaacaagatgtttcttttgtcccttccaggcgatcggaaaatgttgatatattcaagataattacgtatttacaaaaaaccgtctcaattcatcctatttcatcagatccgggatgtgatatggttccgaaggatgaatcggatatggacagttccaataagatttcattcttgaacaagaatccattttttgatttatttcatctattccatgaccggaacaaagggggatacacgttacaccacgattttgaatcagaagagagatttcaagaaatggcagatctattcactctatcaataaccgagccggatctggtgtatcataggggatttgccttttctattgattcctacgggttggatcaaaaaaaattcttgaatgaggtattcaactccagagatgaatcgaaaaataaatctttattggttctacctcctcttttttatgaagagaatgaatctttttatcgaaggatcagaaaaaaatcggtccggatccactgcgggaatgatttggaagatccaaaactaaaaacagcggtatttgctagcaacaacataatggaggcagtcaatcaatatagattgatccgaaatctgattcaaatccaatatagcacctacgggtacataagaaatgtatcgaatcgattctttttaatgaatagatccgatcgcaacttcgaatatggaattcaaagggatcaaataggaaatgatactctgaatcatataactgtaatgaaatatacgatcaaccaacatttatcgaatttgaaaaagagtcagaagaaatggtttgatcctcttatttctcgaaccgagagatccatgaatcgggatcctgatgcatatagatacaaatggtccaatgggagcaagaatttccaggaacatttggaagatttcgtttctgaacagaagaagcgttttcaagtagtgttcgatcgattccgtattaatcaatattcgattgattggtccgaggctatcgacaaacaagatttgtctaagtcactttgtttctttttgtccaagtcacttctctttttgtccaagtcacttccctttttgtccaagtcacttccccttttctttgtgagtatcgggaatatccccattcataggtccgagatccacatctatgaattgaagggtccgaatgatcaactctgcaatcagttgttagaatcaataggtgttcaaatcgttcatttgaataaattgaaacccttcttattggatgatcatgatacttcccaaagaccgaaattcttgatcaacggaggaacaatattaccatttttgttcaaaaggataccaaagtggatgattgactcattccatactagaaataatcgcaggaaatcctttgataacacggattcctatttctcaatgatatcccatgatcgagacaattggctgaatcccgtgaaaccatttcatagaagttcattgatatcttctttttataaagcaaatcgacttcgattcttgaatgatccacggttctattgtaacaaaagattccctttttatgtggaaaagacccgtatcaataattatgatcttacatatggacaattcctccgcaacaaaatattttctttgtgcgtcggtaaaaaaaaacatatttttttggagagagagactatttcaccaatcgagtcacaggtatctgacatattcatacctaacgattttccacaaagtggtgacgaaacgtataacttgtacaaatctttccattttccaattcgatccgatccattcgttcgtagagctatttactcgatcgcagacatttctgcaacacctctaacagaggaacaaatagtcaatttggaaagaacttattgtcagcctctttcagatatgaatctatctgattcagaagggaagaacttgcatcagtatctcagtttcaattcaaacatgggtttgattcacactccatgttctgagaaatatttaccatccggaaagaggaaaaaacggagtctttgtctaaagaaatgcgttgagaaagggcagatgtatagaacctttcaacgagatagtgctttttcaaatctctcaaaatggaatctgttccaaacatatatgccatggttccttacttcgacagggtgcaaatatctaaatttcacccttttagatactttttcagacccattgccgatactaagtcaaaaatttgtatccatttttcatgatattatgcatggatcagatatatcatggccaattcctcagaggattcttccacaacggactctgataagtgagattttgagtaagtgtttacagaatcttcttctgtccgaagaaatgattcatcgaaataatgagtcaccccttccattgatatggacacatctgagatcaacaaatgctcgggagttcctctattcaatccttttccttcttcttgttgctggatatctcgttcgtatacatcttctcttcgtttcccgagcctctagtgagttacagacagagttagaaaagatcaaatctttgatgattccatcatacatgattgagttgcgaaaacttctggataggtatcctacatctgaactgaattctttctggttaaagaatctctttctagttgctctggaacaattaggagattctctggaagaaatacggggttctgcttctggtggcaacatgctattgggtggtggtcccgcttatggggtcaaatcaatacgttctaagaagaaatatttgaatatcaatctcatcgatctcataagtattataccaaatcccatcaatcgaatcactttttcgagaaatacgagacatctaagtcgtacaagtaaagagatctattcattgataagaaaaagaaaaaacgtgaacggtgattggattgatgataaaatagaatcctgggtcgcgaacagtgattcgattgatgatgaagaaagagaattcttggttcagttctccaccttaacgacagaaaaaaggattgatcaaattctattgagtctgagtcataatgatcatttatcaaagaatgactctggttatcaaatgattgaacaaccgggatcaatttacttacgatacttagttgacattcataaaaagtatctaatgaattatgagttcaatagatcctgtttagcagaaagacggatattccttgctcattatcagacaatcacttattcacaaacctcgtgtggggctaatagttttcatttcccatctcatggaaaacccttttcgctccgcttagccctatccccttctaggggtattttagtgataggttctataggaactggacgatcctatttggtcaaatacctagcgacaaactcctatgttcctttcattacggtatttccgaacaagttcctggatgacaagcctaaaggttatcttattgatgatatcgatgatgatagtgacgatatcgatattgatcttgatacggagctgctaactatgacgaatgtgctaactatgtatatgacgccgaaaatagaccgatttgatatcacccttcaattcgaattagcaaaagcaatgtcttcttgcataatatggattccaaacattcatgatctgtatgtgaatgagtcgaattacttatccctcggtctattagagaactatctctccagggattgtgaaagatgttccactagaaatattcttgttattgcttcgactcatattccccaaaaagtggatcccgctctaatagctccgaataaattaaatacgtgcattaagatacgaaggcttcttattccacaacaacgaaagcactttttcattctttcatatactaggggatttcacttggaaaagaaaatgttccatactaacggattcgggtccataaccatgggttccaatgcacgagatcttgtagcacttatcaatgaggccctatcaattagtattacacagaagaaatcaattatagaaactaatacaattagatcggctcttcatagacaaacttgggatttgcgatcccaggtaagatcggttcaggatcatgggatccttttctatcagataggaagggctgttgcacaaaatgtacttctaagtaattgccccatggatcctatatctatctatatgaagaagaaatcatgtaaggaagggtattcttatttgtacaaatggtacttcgaacttggaacgagcatgaagaaattaacgatacttctttatcttttgagttgttctgccggatcggtcgctcaagatctttggtcttcacccggacccgatgaaaaaaattggatcacttcttatggattcgttgagaatgattctgatctagttcatggcctattagaagtagaaggcgctccggtgggatcctcacggacagagaaagattgcagtcagtttgataataatcgagtgacattacttcttcggtccgaaccaaggaatcagttagatatgatgcaaaatggatcttgttctatcgttgatcagagatttctatatgaaaaatacgaatcggagtttgaagaaggggaaggagccgtcgacccgcaacagatagaggaggatttattcaatcacatagtttgggctcctagaatatggcgcctttgtggcaatctatttgattgtatcgaaaggtccactgaattgggatttccctattgggccgggtcatttcggggcaagcggatcatttatcataaagaggatgagcttcaagagaatgattcggagttcttgcagagtggaaccatgcagtaccagacacgagatagatcttccaaagaacaaggcttttttcgaataagccaattcatttgggaccctgcggatccattctttttcctattcaaggatcagccctttgtctctgtgttttcacgtcgagaattctttgcagatgaagagatgtcaaaggggcttattacttcccaaacaaatcctcctacatctatatataaacgctggttcatcaagaatacgcaagaaaagcacttcgaattgttgattcatcgccagagatggcttagaaccaatagttcattatctaatggatctttccgttctaatactctatccgagagttatcagtatttatcaaatctgtttctatctaagggaacgctattggatcaaatgacaaagacattgttgagaaagagatggcttttcccggatgaaatgaaacatttgattcatgtaacaggagaaagatttcccattccttagccgtaaagatatgtggccatgaaaaagggaagggattaagtggaacaggattggccgggtggtagagtttattcgaagcgcttcgtgattttcaaccaattatgtgcttcaatataattacctggagtaagcgctatagcttgtttccaatactcagcagcttgatcggaccaagcttccgcaatttcagaatcaccctgtagaatggcctgttctccccggtaatgacagatcacggccatattattaaaagcttgtggtaagaatgggtttcgttctagtgcccggaaataatattccaaagcctttgtatgctctccattgcttgtgtgtataaggcctatgttatagagtatataacttcgatcatagggatcaatttctggtcgcgtagcttcataataattctgtaaagcttccgcataatttccttcggattgagccaacataccatctctgtaataggtaaatgcctttttttctcctgaagttgtcggaattattcgtaataagatattggctacaattgaagaggtcttatcaataaaatttccatttatatgagatctaggcataattagcaatccattctagaattcttttcattacccggggaaaatgatcccacaaacaaaggaattatacagtacgaaataacataaaaaactttattctaaaatagatatgggctttccacttaaattgtccccttttgtttggaaagatatgagatattggaaattgatttcattcccatttttgtagtataccaatgagcggaactattactatttcatctaagttaaataaccaaggacttttttactacagattctaataactcgagaagttttgatttgattatgatccaaagagaaaaaagaatggaataatcattccatgaaaaaatagagtagagtaaccataccttctgtttgcataagtgtatacaccacgccatacaatcgaaatataaaaatccatgggacgatcataaatttggaatagatccacggggtagctgatgaatgagagaagtttttgttgagaaatattaaatgggaaaggaattcttcctatgtaactagtgatcggtcgtacctgtactgcagtaatatgaataactcgctattcactcagtttctggtcaataataagttatgtacggaataggtggttccttcccttagaaccgtacttgagagtttcctactcatacggctcaaaaatcgattcttttcttacctatgctaactgaattagatttctcataaacctatcccatttttcttgggttaaccagaagaagttaattacataagtttcaaaccctaattttgatcaataatcagaatcagtttgatcttttctcccaccttcagaagaatgaagcataggtatccccacaatatcgttagaattttctgaaaggtaactatctcggtttcatatatggaattcatatagaatctttgaaaaagactttttacataagaaaaaagaacttactatctttgggatctgatgctacaccgctgctcaataccttagtggatcgactctattacataagttgattcctaacttttgcccatatcatggcataagtaagcagttcttaactgtatcgactcaatagctcgctaattgatctttacggtgctttctctatcaatttgatcctttatccatagaatatagtatataggctgcactcattttttttcttcctattttggttctcgtgaagtctctttccttgctacagctgataaaaatcgttgctttggacgatgcatatgtagaaagcctatttttctagtatttactagttgatctttgcttttttccttatttctatagtggagatagtcgcacgttacggtcgttcattctattcaaaaatctccgttccagaaccgtacatgagattttcatctcatacggctcctcccttctgcgcatagtactaagggaataatccatagaataaaaatggaactattctcatctcattatgaactgaaaggaactagtatttttacaagaaatctctagccagccttcccgcaagaggtttttcttaacaccaatcatattagtgttagatataaatggtaactccaacaatttctttgttctcaacgccttctatttccaggaattagtcacttcaacgatctttgatggttatacgggtatccaaagtacaaacgagatggatgtttgttgtcccaaccattcttgttagtcccgataccgataaggaaagggggaatttataacaaagtttttgtgttgttgattcctaggtgtagtgctttttcccttatgccgtctattggtactaatgtagtgtaggattgacccgcaatacagagcccataggtgtaacctttcgctcaatactcaaatcaacaattgaaacatctgaggctgcatcaatcgaggatacacgatagaaggaattgttctatctccaaacttcaccttcaccgagcgtaggtttatttcaagaatttcgttctttctataccgaaccgcgtctctttctcgtaagactgaggtgaggaaaaaaacaagaaaaaagaatcaatgaattggcgatcagaacatatatggatagaacttataagggggtctcgaaaaacaagtaatttttgctgggcctgtatcctttttttaggttcactaggattcttagcggttggaacttccagttatcttggtaggaatctgatatccatatttccatctcagcaaattattttttttccacaaggaatcgtgatgtctttttacgggatcgcaggtctgttcgttagctcctatttgtggtgcactattttgtggaatgtaggtagtggttatgaccgattcgatagaaaagaaggaattgtgtgcatttttcgttggggatttcctggaataaatcgtcgcatcttccttcgcttccttatgagagatatccaatcaatcagaattgaggttaaagagggtctttatcctcgtcgtgtcctttatatggaaatcagaggtcaaggggccattcccttgactcgtactgatgagaattttactccacgagaaattgaacaaaaagctgccgaattggcctatttcttgggcgtaccaattgaagtattttgaattgaaagaataaattctcggcatgggggaaggaacttgctaattccctttttaatataattgaattttggaatgttcatttgaacaaaacatgttagattattctatttcctccttcctttgtcgtggcgactcccatagaataaaccaaaaaggagggcgatatggaataactataataaactataattaagaaaagaataaatttttgtataccaaaagtatttcatatgcgtatgggtcccaactcaattcttttctactagaaaatttctactagtctaataagtagggattcatcaaatatatcgatatttcgtaatacggactcatctttttaggcccaaaagatctttttccttggttgtggctaggcggtgaaacatttcgaaataattaactgggggttctaaatccgatttgttattttagtttcgagtattcatagaaaggaacaaatgaagatgaaattgcaatttgcccaattgagatatctaggaataatattgatttttcatttttatacgaaagggcaaactttctatctagatccaagaactaaactcaattaggttcaataccttgttataaactcgtgcttcagagaaatatcatatagagtcaacgaatgagttcattaacgattcaattcacagatggcgtactcctcctgtttgaatcggagtttgaaaccaaactcaggtgagatccaatgtagatctaactttctattcactcgtgggatccgggcggtccgggggggaccaccacggctcctctcttctcgagaatctatacatcccttatcagtgtatggacagctatctttcgagcacaggtttaggttcggcctcaatgggaaaatggagcacctaacaacgcatcttcacagaccaagaactacgagatcgcccctttcattctggggtgacggagggatcgtaccattcgagcctttttttcatgcttttcccggaggtctggagaaagcagcaatcaatagctcacttcttggtcttcgaccccctcagtcactacgagcgcccccccgatcagtgcaatgggatgtgtctatttatctatctcttgactcgaaatgggagcagaaaaaggatcttagagtgtctagggttgggccaggagggtctcttaacgccttcttttttcttcccatcggagttatttcacaaagacttgccacggtaagggagaagggggaacaagcacacttgaagagcgcagtacaacggagagttgtatgctgcgttcgggaaggatgaatcgctcccgaaaaggaatctattgattctctcccaattggttggatcgtaggtgcgatgatttacttcacttctcctcaggaggataggtggggcgat

BK061352 atggaaaaatggtggttcaattcgatgttgtctaacgagagaacataggtatggtttaagtaaatcaatggaaagtcttgatgctattggccataccagtggaagtgatgaaccccttctaaatgatacggagaaaaattggagtgatagtttcagtaatgttgattatttatttggtatcagggatatttggagtttgatctctgatgacacttttttagttagggatagtaatggtgacagttattccgtatattttgatattgaaaatcatagttttgagattgacattatcattacatgtatgatactcaatctagttggactaatcaattaatagttccatttcaggtggtactgacaatttctgaaagttttagtataagaactcgtaataatggcagtgatttcaatataagaggaagatctaatgatttcgatataaataaaaaatacagacatttatgggttcaatgccaaaattgttatgaattaaattataaaaaacttcttaggtcaaaaatgaatatttgtgaacagtgtggatatcatttgaaaatgagtagttcagatagaatcgaactttcgattgattcgggcacttgggatcctatggatgaaaatatggttcctatggaccccattcaatttcattcagaagaggaaccttataaagatcgtatcgattcttatcaaagaaagacaggtttaactgaagctgttcaaacaggcataggtcaactaaacggtattcccgcagcaattggggttatggattttaagttcatgggaggtagtatgggatctgtagtaggtgagaaaatcactcgtttgattgagtatgctactaatcaatctctacctgtcattattgtgtgtgcttctggaggagcacgcatgcaagaaggaagtttgagcttgatgcaaatggctaaaatatcttctgcttcatatgattaccaatccactaaaaagttattctatgtaccagtccttacatctcctacaaccggtggagtaacagccagttttggtatgttgggagatatcattattgctgaacctaatgcgtacattgcatttgcgggtaaaagagtaattgaacaaacattgaataagagagtacccgatggttcacaagcggctgagtatttattccataaaggcttattcgacccaattgtaccacgtaatcctttaaaaggtgttctaagtgagttatttcagctccatggtttctttcccttgaatcaaaattccATATTATTTTTATATATACTTAGTAGTTAATATTACTTATAATAGATATACTTATCATAAGATATCTTTCTAATATACAAATATTAAATCGAGGCACCCATTCTttatggttgttcctgaagtagaaattgttcgatctgttcctgaatagcttccttcaaaacggcttctgcttgctcggtgaatgtcttggtagaagatataatttcttggaattgaggtttattcttttttaagtaagtacgtaactgaacaagaaatttctttacctgtccaatttctaacggatcaagatatccattcgctccggtataaatagtagctatctgctcttccaccgtgagagggtctgattgggattgtttaagcaactcacgtaatcgttgacctcttgccaattgattctgagtagctttatcgagatcagaagcaaattgtgcaaaggcttctaactctgcgaattgcgctagttccaattttgatttgccagctacctgtttcatggctttaatttgagctgcggatcctactctggaaacagaaatacccacattaatagcaggtcggattccagcattgaatagatcggcggataagaatatttgtccatctgtaatggaaattacattagtaggaatataagctgaaacgtctccagattgagtctcaactattggtaaagcagtcatacttccttcacctaaactagaacttgatttagcggctctttccaaaaggcgtgaatgcaaataaaaaacatctcctggataagcttcacgaccgggaggtcttcttaatagaagagacatttggcgataagcttgtgcctgtttggagagatcatcataaattattaaagtatgtcgttcacggtacataaaatattcagccaaagtcgctcccgtataaggagcgaggtattgtaatgtagcaggtgaatccgccgtttcggctacgacaatagtgtattccatggcccctcgctcctggaaagtagtcactacctgagccacagaagatgctttttgaccgatagctacataaacacatattacattttgccctttttgattgagaatcgtatctgtggctactgctgttttgccggtctgtctgtccccaataattaattctcgttgaccgcgtcctataggaatcatcgaatcaatagcaataagccctgtttgaaggggctcatatacagaacgtctcaaaataataccaggggcaggggattcaattaaccgagattcagaagctgaaatttcccctctcccatcaataggtgtagccaaagcatttataacacgacccaaataagcctcactcacaggtatctgagcaattcttcctgttgcttttacagaacttccctcttgtatcatcaaaccatcacccattaatacaacgccaacattattggattccaagttcagagcaatgcctattgtaccctcttcaaattctactaattcacctgccattacttcatcaagaccatgaatacgagcaatgccgtcgcctacttgaagtacggtaccggtattcacaatctttacttctctattatattgttcaatacgttcacggataatattactaatttcgtcggctcgaagggttaccattagtgtttctttattctttttcggaagcaaaaggaaaaataatgcctaaattgtaaactaaagtaaaagtgtcatttcttcaaattgctctccacttctaagttcatagctttcgcggtagcttcatcgatgttacccaccaaataaaaagcctgctcgggcagaccatctaattctccggaaaggatcagttgaaaccccctaattgtttctgcaagaccaacatattttcctggagaaccagtaaatacttctgccacgaagaagggttgtgataagaaacgctcaatttttcgtgctcttgctacagttaaacgatcctcctcggataattcatccaacccaagaatagctataatgtcctgaagttctttgtaacgttgtgaagtttgcttaactctttgcgcagtttcataatgttcctcgccaacgatccggggttgtaacatagttgacgttgaatctaaaggatctactgctggataaatacccttggcagctaatcctcttgatagtacggtagtagcatctaaatgtgcaaatgtagtggcaggagcagggtcggtcaaatcgtccgcaggtacataaactgcttggatcgaagttatagatccctctttggtagaagtaattctttcttgtaaagaacccatttctgtactaagggtaggttgataacccactgcagaaggcattctccctaataatgcggatacttctgatcctgcttggacaaaacgaaagatattgtcgatgaatagaagcacatcttgttcattaacatcccggaaatattctgccatagttagggcagtcaaaccaactctcatacgagctcccggcggttcattcatttgaccatagactaaagctacttttgattctgcaagatttttttcattaattactccggattctttcatttccatgtaaagatcatttccttcacgagtacgttctcctactccgccaaatacggatacgcctccatgagctttggcaatgttgttgatcaattccatgatgagtactgttttacccactccagctcccccaaatagtccgatttttcctccacggcgataaggagctaaaagatctaccaccttaatacctgtttcaaagattgataatttcgtatctaactgtataaaggcaggcgcagatctatgaataggagatgttgtgcgagtatctacaggacctaaattatcaacaggctctccaagaacgttgaagattcgcccgagagtagctccgccgactggaacacttagaggagctcccgtgtcaatcacttccattcctctcatcagcccatctgtagcactcatagctacagctctaactcgattatttcctaataattgttgtacctcgcaagtcacattaatttgctgaccgactcgacccttaactaccaaagcgttataaatattaggcattttgcccgggggaaaaacgacatccagtactgggccaataatttgagcgatacgccctagttttttttcttcaagtgtggaaaccgcagggctagaagtagtaggattgattctcataattataataaagtgaaatatgtcgaaatctttttggaataataccaaatcaaaataaatgtccgatagcaagttgatcagttaattcaataagagataaatgggagatagctttttcatatagatttccgtctttatattataccctttcgtagatgaattatgcttattttcacatctaggatttacatatacaacatatattactgtcaagagggaattttctcagtatttagattcaaaataagaaagggttcaattataaacctgcaaaagattaggattgggttgcgctatatatatcaaagagtatacaataatgatgtatttggtgaatcaaatacatggtctaataacaaaccattttaacataacattttgttgataatattaattgaatattttttgaaagatttttgttaaaggtttcattcatgcctaatccatatcgagtagaccttgttgttgtgagaattcttaattcctagtaggaaataacattgacagcctctactcgtgtcctagctcgtctgagagctagattcgcttcaatcacttgtctcttaccctcagctctactcaagttagcttcagctattttaagagcttgttgagcttcttgcggatcaatgtcagtactcatctccgcatcatttcctaaaatggtgatctcattattccctattctagcaaaaccacccatcagagccaccgttaaccattggtcgttgaggcgtattctcaaaagacctatatctacagccgtggcaataggggcgtggtttggtaatacaccaatttggccactatttgtagataaaatgatttctttcacttctgaatcccaaataattcgattaggagtcagtacacaaagatttaaggtcatctaatcagttatttcttccatggccccaagaatgccaatattagcacggatcgtacggaaatgtaactcggtattcaaacaactattcagagttcctagagctccttgtaaggcttgttggaaaactcgttgtcggacttgatttatcgctctttgttgttcaaaatgaagggtttcatttttgtaattttctaatcgttccaaactatcacaagtagctttaatcaaattttctttttctcgttctatctcagagtatccgttcattcgatactcatctgcttccagttcgactttctgtaagcgaacccgggctttttcgagctgctcaatggcccctctacgtaattcttccgaatttcgaatagtactcaagatcctctgttttcgattatctaataaatcatttaacactccctttccaaaaaaaatcaatacaccaatcactacacttagatttattggatttgttgctaaaatatcggtattaaacccgaaacccccggcggatggccaatggcgtgagaaaacgaaagaatcggttacatttttcatatgctttcctcttatagataggactgacaaagaacaaagtttttttctattacttcgctcttttttgatcaatttatttttaattgaatttcccctttttatgggaatagattaaatctagtaatttcgtttaggttaggtcttaggtctcatttcaattgcaaaatatatcgtttgtggaaacgtttcctaaaaggaaaaagtttccattgtactaagctaaggacgggaaggaagaaagcgagtgatctggtaattcctcatcctcgaagcagtccttcctggagtctcaacaaataagtaattataggagtaattgatataattcgaagaagcaaacgattcaagttaataaaaaggtacttttttacattttgaaagtagattatcttaccattaatttcacaactttcatgatctcttcccgaaccaaacatgaatctttcgattcatttggctctcacgctcaattatttatttgattttttgttatgggtattcccatattttttatgtaatgagcctgccctctcttttctgtttgtattcaaagatatctaaactgatacaagaccagaataaatattaggaggactcttccgaccagataaaaatcgataattgtcagcaaagttgtttctttatttgtatccaaaaattcctcttttttatacataggtcgtcgattcggcattggataaaaaaggcagagtgccttttctttctagtaaatggttcaaatccttttatcgatatgagtgttctatatcagataaattaccaactattcattttgaaaacatttcagtactaatgtagtcgtagaaagagtaccatgttttgcctggacttcaaacagtttagctttaaccatgttaatggtctcacattattggttgatagagaatcaaagttgatttaccaataagttacgaaatgctatggttcttacatatgatttttaaatttattcagaagtaattcgtcgagatcgtgcaccttttttcctatttatcctaataactataaataaagtaaaatgcagccggatggatccaacctattcttgaaatacacaacccgcacttaaacaaaaggattcgcaaataaaagcgccaatgccacaaccagtccgtaaattgttaaagcttccataaaagctagactaagcaataaagtacctcgtattttaccctctgcttctggttgtctcgcaataccctctacagcttggcctgcagcagtaccttgaccaactccgggtccaatagaagcaagtcctacagccaatccagcagcaataacggaagcggcagaaatcagtggattcatctcgcaaaaagaaatggttaatgatacaatcaaccaatgaattattacttaattttatcattaagatctattgggtcggagtaactaaaaactaatgataatattactgaatcgtcagaactacttcgatatctcgttttttgtttctacccatgatgaagtttttgtaaatccatatcggctctagttattgcatttctttccaaccattctttcattccatctttcgttctttactcttctatatccttgagttcatctccacaatcacaaatgaaacagaagaaaggacttgacttatcttgtaatccatctaatctaaatgcagtaaactgcaaccaaatcaatatatgcatatgatcgatatctatatatagataggactatataactagtgaatatctattacatttctttcttccataatgtcacattttatattgaatcggattataaatcattcctcgaaacccacacaaaaagtggctggacttatagacattacatacatctagtgtgacctccccaacctttttttaattccgtaatatcgttcatcttctctcctacgactctaggtcatatattcatacttatatctattatgttctccaaccaagcagattatcttgaaccatgctgggataagctaaaaaaaagactatttcgaaaactagttaatgatgaccctccatggattcgcctatataagccgcggctaacgttgcaaaaataagagcttgaataccacttgtaaataatccaagaaacatgacaggtataggaactactgaagggactaaagaaacaagaacaacaactactaattcatcagccaatatattcccgaaaagtcgaaaactaagagataaaggttttgtgaaatcttctaggatgttaattggtaaaagtattggagttggtttgatgtatttctcgaaataacccaacccttttttggtaagacctgcataaaaatatgccactgacgtgggtaaagctaaagcaacagtagtatttatatcattcgtgggcgcagctaactccccatgaggtaactgtatgattttccaaggtaaaagagcacctgaccaattagaaacaaaaataaataggaacatagttccaataaagggaacccaaggtccatattcttctccaatctgggttttgctcaagtctcgaataaattcaaggacatattcaaagaaattctgaccgtcggtcggaatggtttgtggattccgaacagctatgatggctgaacctaacaagatagcaattacgacccaagaagtgataagtacttgggcatggatttgtaaacctcctatttgccaatagaaatgttggcctacttctacacccgatatatcgtataaccccttgagtgttttaatgtaacatggtataacattcatattgtcctctgatagaaattgaacttcaaaaaaggaattagtttgattcaaccttctcaactcaccaacttgaatcatttatttaggataccaagaaatcacataacatcataatatatatcaatatccccagttttttttatcaaaaaaagtaaccgatccaaaattaacattaactaattttattattcttaaatgatatttgcgactttagaacatatattaactcatatctctttttcgatcatttcaattgtgattacgattcatttgatgaccttattagttcacaaaattgtaggattacgtgattcgtcagaaaaagggatgatagctacctttttctctataacaggattattagtttctcgttggatttcttcgggacattttccattaagtaatttatacgagtcattaatcttcctttcgtgtagtttcttcattattcatatgattcccaagatacgtaacgatttaagcacaataattgcaccaagtaccatttttactcaaggctttgccatgtcgggtctttcaactgaaatgcatcaatctgcaatattagtacctgctctacaatctcagtggttaatgatgcacgtaagtatgatgttattgagctatgcagctcttttatgcggatcattattatcagtcgctcttctagtcattacatttcgaaaaaacatcaaaattttttgtaaaagcaataatttattaattaagtcatttttcattgaatatttgaatgaaaaaagaagtgtttttaaaaacacttcatttcgaaattattacaaatatcaattaactgagcgtttggattattggagttatcgtgtcattagtctagggtttacctttttaaccataggtattctttgtggagcagtatgggccaatgaggcatggggatcctattggaattgggaccccaaggaaacttgggcatttattacttggaccatattcgcgatttatttacatactagaacaaataaaaattggaaaggtacgaattcggcacttgtagcttctataggatttcttataatttggatatgctattttgggatcaatctattaggaataggtctacatagttatggttcattcacataaacatctaattgaataaactacatgaagaatacataagataaaaaatcatcccatatataacgaaagtttttatgagtttttgagaaccatttaaattaatgattccttgaaatggttctcaaaaactcgagatgtatctaattacaattcttattcattttatttcttttttcattatacagtacagcaaacaattttttaaatattaaattcaaagaattatatttccttccgtttcattaatgaaacaatgaaaaaaaagaaagcattgccttctttcccatatcttgtatctatagtatttttgccctggtgggtctctctctcatttaataaatgtctggaactttgggttacaaattggtggaataccgggcaatccaaaactctcttgaatatcattcaagagaaaaacgttctagaaagattcatagaattagaagaactctttctgttggacgaaatgataaaggagtacccggagacacatatacaaaaacttcgtataggaatacacaaggaaacgatacaattggttaaaacacacaatgaatatcatctccatatcattttgcatttctcgacaaatataatctctttcgctattctaagtggttattttattttgggtaatgaggaacttgtcattcttaattcttgggttcaggaattcctctataacttaagtgacacaataaaagctttttcgattcttttagttactgatttatggattggatttcactcgactcatggttgggaactaataattggttcgttctacaacgattttggattggctcataatgatcaaattatatctggtcttgtttccacttttccagttattctagatacaattgtgaaatattggattttccattatttaaatcgtgtatctccttcgcttgtagtcatttatcattcaatgaatgaatgaagaactcatttgatctcctgatatcaatcaaatcagaatctttctttataaaccattttgaatcttacttaattctttatatttctaccagttcaaggtattcgtcctatattacagtacaactattccagtacaatgacagactcgtgcatagggaactttactagttccctatctaatttattgtagaaattccgagatccatgattggacttcgaccgctacaagatcaacaatgccatgagcttgggcttctgttgctgacataaaaacatccctttccatatcctcggatacaacccataaggggttgcccgttctttgtacataaacctttgttagggtttcgcgaagtttcagtagttcttccgcttccaggataaattcccctgcttgcgcctcataaaaagaactagcaggttggtgaatcataaccctagcgtgagggaatgctagacgtttggtaatttctcctccgaccagaataaaagatcccattgacgcggctaatcccatgcatatcgtatgcacatcaggtgacacaaattgcatagtatcataaatggctattcctggtattacccatccgccgggagagtttataaacaaataaagatccttagtaccatcttctatactgagatataccatgagaccaacaagttgattcgagatctcgctatcaacctcttggcctaaaaaaagtaatctttctcgataaagtcggtttacgtcaacccaaaccgcatcttcctctccaggactccgaaaaggtacttttggaacaccaatgggcattaaattaaagaaaaatttagtactatacttcactttaatatggaaacgtaacaatcaatggtttattgtcttcatcccttttttctctgattggaaagtttatagagtaagacagagaaatcgatcgttcgaatgataaacaagtatctatccattcgttccccaaaaatgggaccaatcctcccattgcgtattggtacttatcgggtatagaatagatctgcttctctttgttcttacgaacaaaattgttctttcaatggaatggaataaatattaatcctttctgatacggaatctactgaaattaggtatatagtatatatagtcttttccaatgcgataaaataaagtgacatagtgtctatttttctttgataaagaggtatttccgatgatattgatataacgattcttctatctcgcatgattgggctaaactaaagtaggggataaaaaataacaagaaaaaaaatagaattgaataaccgtacaggcatcttttgttcattgcatacggctctgcaatggaattgactttttcttctcttctattctatcgaagaaagaaatagaatccatctaatccagatcgttgaatgatccatttaccacccttcctttcatagaagtaaaaaagaatactatgatggttctgttactttatatatttatctcgtctgtgatttagcaatcccaaagtttctttttgatatgatcaaataagatttttcatactctttctttcatagcataagtatcagaaagagacttctggtgtggaagaaaaatggtttgtgacgctgaaatgtactcccgacacataagtcaaatcggaaataatctttatttcatactactatctcgatacaaaatctcatgttatgaaaaacaataatggtttgttcatatcgaacccgaagtgccatgctattattacttataattttcttttataatcaatgtggcgaaggcatagtctttttttcaaataaaaactcattggcgccagaaccgtacgtgcacctttggatacatacggttcaaaaaagaaaaagaatcaatgtgtcgattccagttttattttaacaggttttcttaatgaaaggtcttctattaaaaaaaagagattactgaacttattaaactaacctatcattgatgtattgtttcatcgatattaaaatcacgatgtcattgtcttgtttctgaatgggccctttcaattcttttaggttcatggtctaccccgggaaaagatctgtccgaattctatttgcacatataggacaaatggtctcagtaccatatcactacagtaatagtaggtataagaatcatttatgatacaagtggtaatcatacatatcaatttgttatcgatttggtattttctgaacggagcctggatactttattttatcagtccaagtaaaccataaattcttctaaattgataatattgatcccaaataaattgatctaattgcacttcacgctccgaatgattgattgatgcctcactcaatacaatatctcttgggcgaaacagaggatatctcgatcaggggagagaacgggtaaatcccatatgacccaatatgtctgacaagtcgcacctaatcgttcgaatctttgttgcgaagtcgataaattatacgtcccctggttgaatcataacgacttacttcaatttttactctatctcccggtagtatccgtataaaactgcggcggatcctccctgaaacatatcctagaattagatcttcattatctaaacggacccggaacataccgttgggaagtgattcagtaattaaaccctcatgaatcaatttttgttctttcattccaggtaacctccttgaagtatcaactaatggaggaagagttatataactcacttttcctctctattttacaaataggaagttagaaaattcggataccagaggtggaagatcaatgattcaccaggttattgatacggataatatccaaataccaaatacgttctcgacgtgatccatgtaaagaaaaaggggttggtttggggaagatcaaagaaagaacttgttcttcttccgtaaaaaattcttctaataatatcgaacctaatctctgcaaaaaagagcgtactgtactcttatgtttacgagccaaagttctagcacacgaaagtctaagtatatactttattcgatacaaactccgtttttcggaggatccactatgataatgagaaagatttctacatatccgaccaaatcgatcaataatatcacaatctgataaatcggtccagatcggcttactaataggatgtcccgatacggtacaaaatttcgctttagacaatgatccaatgagaggaataactgggactatggtatcgaatttattaatacctgtttctattagaaatgaattctccagcatttgattccttaccaccaaaggatttcttagtacacttgaaagataacccaaaaaatagaaggaatagtttgataattggtttatatgaatcctgtgtggttgagaccaaaaatgaaaataatattgccagagattgacaaggtaacatttccacttcttcatcaaaagatgagtcccttttgaagccagaattgcttttccttgatatcgaacataatgcatgaaaggatccgtaaaaaaccataaagttttctgaaaatggttatggtgcactactataagatgttctatttttccatagaactgtattcgctcaagaaaggttccagaagatgttaatcgtaaataagaagattgtttacgaagaaaaacgaataaaaattcgcattcagatacataagaattatataggaacttaaatagtcttttattttcttttgaaaaaacataaattgatttcttcggagtaatgagactattccaattatgatattcgtggagaaagaatcgcaataaatgcaaagatggaacatcttcgatccgacattgaaggatttgaaccaagatttccaaatggataggatagggtattagtatatctgacacataatttaaatgtaataatttgtcctctaaaaagggaaatattgaatgaatagatcgtaaattatgacattttggtatttctttttcttcgggaaaagatactaatcgcagcgagaatggaatttccacaatgaccgcaaaaccttctgatatcatctgagaaaaaaaatgagaataaaaataattgttgtgcccaacgaatcgattttggttagaataattaaccgaattaatcaaataattctgttgatacattcgaataattaaacgtttcacaagtactgaactagatttattgtcataaccaaaaatttccacgggttcgtaaaaaatagaatcgtttaaaccatgatcatgagcaaacgcataaatatactcctgaaaaagaaacggatataggaagtgttgttgccgagatctatctttttctaaatatccttgtaattcttccatttacatttctacttgagccagaggcaggaggttttcttggtttatcaaatgatacatagtgcaatatggtcagaacaggtaaagaaaaaatatataccccggaaaagaaagagggagtccaatcaacagatctttttaccttttctatccaattggtttatgttcgttataattacaacaggaaaaatcctttatttttgcaacccaatcgctcttttgactttggaataaattctctttatcaatatactgtttcttctacacatccgtctacaatccataataaagaataattaggattctggttcactcacagaagaacccactctttcccgcattaggcactaattcatttttaacgtctaattagatcgggtaatcattccaattaagaacataagctcgttgctttttattttaccagaattggagccatagagctctatccatttattcactagacccaactgtaaatgtgaattttttttgtccccttccaaaaatcataattgattttattacgacatgctgctttttccattcattacccttgaggatcagtcgtggtcttatagactataccaatagtctggacgaattcgttgcttcatccaaatgtgtaaaagatcatagtcgcacttataatgaaacaagttgggaagaagttgttaataacagattacctagagaaataggtaaaagaaatttccatccaagatttaataactggtccattctcattctaggtaaagtccatcttgttgtgatagaaatgaagagaaacaaataagctttagttaatgtaataaggatacccattgtcattccaaaaatgctggtgattttatttattccgaaaagctcagaaatggatatatacggaatagataaattccacccacctaagtaaagaactgttacaaataatgaagaaactaataaatttaggtaagaagcaagataaaataaaccgtatttgatacccgaatactcggtttgataacctgctactaattcctcctccgcttctggtaaatcaaagggcaatctctcacattcggctagagaagaaattagaaaaacaataaatcctataggctgacgccacagattccacccccaaaaaccatattttgactgtgcttcaactatatcaactgtacttgaactgttagataatagagatattgataacacacatagagttaatggtatttcataactaatagattgagcagcagctcgtagaccacctaaaaaggaatatttattattcgatccatatcctgacataagaagcccaataggagcaatactagaaatggcgatccataaaaaaacacctatactgagatcggctaaaacaagatgatacccaaaaggaattactaaataacttagtagaattgatataaccgctatagacggtcccacactaaacaaacgaatatctcctcgagatgggaggaggtcctctttaaaaagtagtttagtcccatccgctatagcttgaagaattcccaacgggccagcatattcgggcccaatacgttgttgtatcgctgcagatatttctctttctaaccacacaattactagtacccccattgttattcccaatataagggtcaaaatgggtacaatccatattagtccatacacttcttttaaaaattccgatgtagaaaaggaattgatagtttgtacttctgtcgtatcaattatcattcatagtcgataataacatcactgttcccatcgctatttcaaaaccgtacgtgagacctcagcttcatacggctcctcgatggccacaaaaataaggacgggtttggtattatcgccatctttggatagatagaataaagatacgtcggaaagtcccaaattagaccaaggaattctgtctgctagaataagaaaaaagtgcttccgaattgatctcatcctttacaataaaaatttatctttgttcggtaataacttaatatttcaataaaatactccttataccaatcaatacaaaataaaaagaattagtcattagttcatgaatcgtgataagaattcgatatgtatgaatatggatagagaaaagaataaataaggatccccttttttattattcattcaattactgcattccatttcttttttcctgttcttctgtctcagaggaggatactaaaataaaggattaattcgttcttgatagtcatttctttaaccagtgaataggagcatactctagatcggaatcgtagggaagtactacttgatcatttctaccaatttcaagtccttattatgattcgttttatgaagaaatacctcttttttgataccttacattatctccattactaatcctttgtgtaccttggtgttcctaaccgtccactgacttttgattgatccccggtatagtaatacatacgagacagtagtagaaactccatacagttgatcttttgtttgcgcccgcttcaagatatgatgactaataaaaaaatcttaatcttggggtaagcagtttacactgcttatttttacttcaactttattcttgtacatagggaatgagattgtttcttcttactacaaatttggaagctgtttagtttcactcatataaccatctggtttaactcatcaaccccaatgctgaataaaaaagaaaacaatacattgaacctctttcctttttcttttatttctagaagagttcatattccaacgaatcacacctaaaagagggtatcctgagcaattgcaagaattgggttcattgatattcctggtatagtagatgctatcacacatacagtcatactcaattcgatggaattgtttgatcttaaaggggatcttctataatttcgcacgtaaggggttatttcttggtttcgtccagtcattaataacttgattatttttagataatagtagatagaaacaacgctcgtaaggagtcctattgaaaccaagaaatataggcctgcctgccatccacaccagaatagatagagttttccgaaaaaacctgctagtggaggaagacctcctagggataagagacatagggctgaagagagagccaaaaaaggatctttcgtgtataatcctgcataatctcgaatgttatcagttccggtacgtagaccaaataatacaatgcgagcaaaagttcctagattcatggagatatagaacagcatataagttatcatgcttgcatatccatcatttgagtctccaacaattattccaataattacatatccgatttgaccgatggacgaatatgcaagcatacgtttcatgcttgtttgagtaatagcaatgagattccccaatatcatgctaagaatagctaggatttccagaagaagatgccattcgtttgatgagaaataaaaaggaatatcgaaaattcgcgtggctgaagctgaagcagctacttttgaagtaacagaaagaaaagcaacgactggagtgggagatccttcgtatacgtcaggagtccattgatgaaaaggggctggggaaagcttgaacccaattcctacagtgatgaatataagcgcaattgaaattcctggggagttatacatttgtgtattgataagaccattcactatttcttgaagctcgatctcccccccagatgaaccatatagccaagagaaaccatgaaccagaatagaagagcttgccccacccatgagtaaatatttcatagtagcctcattagaccgtacatctctcttggtatatccggataataggtaggaacataaactgaaacattctggagctacaaagatagttattaaatcgttagcaccacataaaaacattcctcctagagtagctgttaatacgaataacagaaactctgttatagccatttctgtacattcaatgtactctacggatagaggaatacatagagttgaacatagtaaaataagaaattgaaagatttcgttgaaattgttcgtttggaaatttcccgaaaagctaattataggttcttctctccatcggaacaatagggccgttatgcttattactaaacttgttgaagagatgaaatagaaccaaggtctatctttttgatcagaggttgaatcgatcatcagaagaagaattagaccaaaaattaggatacattctgggaaaatgaaacttccatggaagagaagcaaatgaaacgctttcataaaaattctcgtagaatcgagaatgaagttttcattctgtacatgccagatcatgaattagtaactgcatccaatctccgaaaaagtcccaattgtttcgaactttctatttttggaatgggatatttacggaatcccatgaataggatcaaaccttattccatgatatttccataagattcctctttcttattcttaagcaagcccagggcttagttgatcatgatttatgttttatctttcttttcctttttgtttgtttcgagaatcgtccgattctttttcttgtgtctacatagatcctgttcatggagtcagagtcgaaaagaggattcctcacttctttctctcattcaaaaccgtgcatgagactttcatctcgcacggctcctaagtgataaaagaaagaagaactcatcttctttcttttttgattaccttcctcgcgtatgtataagaccgaatccattcgatttctcgattactaatccttaacttttcgaggaatccttcatcagtggttgtgaatgactgactttttcaatcctttcgaccttggttccgtaggagcaagtcagaaagattgagaaatagaaccatctgatttgattcgttctcaatagccatgagatgatcatcttagggtgatccttttgtcaacggatgctcctattacactcgtagtctctgaaggatgagaaccaactatgtagcatctacatcgataattcaagcattgtatacgtcattagtccgatcctttgtaggaactacccgtaataacgaacttgtaaaatggatctgtttatcataaagagattcgttgttcctgaccctgcttcaccttaattgttatttgaacaaaaagatcaccttttggtaaaagttatgtcttggtccgagtggggatagcatttctcttctgcatgtctatggagttttgaaaaatccaaacatctcagagatagatatagaggtaggaatttgtcgaacgaaccgcacttaagaccattccaacgctccctttcgccatgcataaactgaacccacaattgggataagcacgaaaattaaagcttcgataaatacagatacacccaatacatcgaaactcattgcccatggataaagaaagaccgtttcaacatcaaaaacaacaaaaactagagcaaacatgtaatagcggattcggaattgtaaccaagcgtcccccataggttctatgcccgattcataactagagagcttctctggtcctttactaaccggggctaaaactcctgaaattacaaatgccaagataggaataacgcttgatattattagaaatgcccataaaatatcatattcgtgaagcagaaacataaatgtactcctattaatgttggcattgtcaattcatccagaacttattttcctaggtgaaacaagaatttattttaatcaaagtgcatagttcagagtttgtttgctgcgcggcatgtcttgtttagagattcatccaaggaatcgggattcctttttgatttcgattctatttagatatggtgtagaggcattcttacacctctattctctattttctttttcttgtcctagatttgacacatactgatctgattagatccattggaatgaattattgtttttattttcactatgtatttacatgaatatgtggtaatgctcatttctatgaaaaaccaatggtctgtccagtctataaacaagtactaatgaggaaatgcaaactatactaaaaatggaatgtctctatctatgataataattggataagatagcgtgtaccttgtcaactgataacgagagaacaaaatcgggataaataccaatacttattacgggtagaaagatacagattgaaagaaatagttctcgtagtccagaatcccaaaaattagagtttggaatattaaatagcttgtatccataaaacatctgacgtaacatagataataaataaataggagttaatatcattccaattgccattacaaaagtaattagtatttttggcattaaaagatattttgtactagtaattagtccaaaaaatactacaaattccgcaacaaaaccactcattcctggcaatgcaagagaagccatcgagaagctactgaacatggtaaatgtttttggcattgggatagatatccctcccatttcttcgagataaacaagacgtgttctatcacaactcgttcccgccaagaaaaaaagtgcagcaccaataaatccatgagagagcatttgtaaaatagctccattgagtcccatgtcggttatagaaccaattcctataattgtgaaacccatgtgagatacagaggaataggctattctcttttttaaattacgttgaccaagagaagttgaagctgcatagattatttgcattgttcctattatcaccaaccaaggagaaaatatagaatgggcgtggggtagtaattccatattgatccgaatcaatccatatgcgcccatttttaataggattccagctaaaagcatacatgtactgtaatgtgcttctccatgggtatcaggtaaccatgtatgtaggggtataatcggcaatttgacagcataagcaataaggaagccaaaatagaatattatttccaatgccacaggatatgattgattaattaatctttcaaaatctaatgttggttcattggaaccatataaacccatacctagaactcctattaagaaaaaaatggaaccccctgcagtgtacaaaataaactttgtagccgagtagagacgtttctttcccccccacatggataaaagtaagtaaacaggaattaattctaactcccacatgatgaaaaaaagtaaaaagtctcgagaggaaaataatcctatttgaccgctgtacattgctagcatcaggaaatagaacaaccgcgaatttcgagtaattggccaagctgctaaagttgctaaagtagtgataaatcctgtcagtaaaatgggtcctatggaaagtccatcgattcctagtctccagtggaaatcaaaaacatctatccatttagaatcttccttcaattgcattaatggatcatccaattggaaatgataacagaatgcataagtcgttagaaggagttctaataagcatatacataaagtataccacctaaacatcttattccctctatgagggaaaaagaaaattgaggaacccgcgaatatcggtaaaacaacaagtattgttaaccaaggaaaataactcgtgataaagacaagatacataaagcccgccctcaaaataatatattttgtcgagcacgggcttttgtcggtaaagaggaatcacaaatgattcaagtggatttttgtaacgtactaattattcaacaaattcgattgattaatacgagttgattttctgttacgatggatcgacgaaacaatagcaagtccaatagctgcttcagcagctgcaatggctataacaaagattgagaaaatgtctccttttaattggcgactatcaaatatatcagaaaatgttacaagattgatattaaccgaatttagtataagctcaagacacataagcgctctaaccatgtttcgacttgtgatcaatccatagataccgatagaaaataaataaacactcaaaaaaaggacatgctcaaacatcattgactaactccttatcaatctcgattcatttcaatatgaacaacaattcaaccgattcaattgattagaatagaacaattacacaacaaaagaagatattgatggtagatagatttaacttagaatttagttagaattctaagaattgggaatcattataagttaagtatttttattgctttttttataaataaaaaaataaataaaaatattgatacataagataaatacaagaatagataagacgagattcgtccacctcccatatatttaaccccttcccctataaaaaaacttgcaacaccaacaccatttgtaattccatcaattatacgtctatcaaaaaactgagttagtttggttaatcctcttatccccacggtaaaaactctagtataaaaaatatctatgtaaccacgattatatgaccaattgtatatcacattttttattcggtccacaagaattcttttaggacccccttttacaaatgaattgattaaatccaaattctggaaaaatgaataagcggatccatataaaatatatgctatgaatagtccgaaaattgctatacttactgaaaaaattgcatttgtaaaaaattcatccaaatttttagaacttgaatgtaaaagatttgttgatggggttaaccatttcgataatatatcaaattgatcaaaagaaattcctattgatccaaccaacaaagtaaatagtactaatacaagaagaggaaatagcatagtattgtccgattcgtgaggatacatggaagtgtatttatttccaaagtaagtactaaagtatcgtatcctatttcttacattattatcaattttcgatctttcttttgcaaaaaaagaaacctttttattcattgttgataaaagtaaatttggattgactcctcttggagttccttttccccatagagatatggaatagaacgaaccatttttcgtgctactgtaatttttaaaatgaacgcgtaaatacccatcaaaggtaagtaaatatacccgaaacatataaaatgcggttaatcctgctgtgaaataagctattactgcgaaaattggtgaatacaaccaactatcattaagaatgtcatctttggaccaaaaacaagcaagaggtggaataccacaaagagaaagtgtacccaataaaaaagtagttcttgtaattggaacatatcttgttaaaccacccataagaaccatattctgacttttatctggtgaatatccaacaataggttccattgaatgaataatagatccggatcccaaaaacaataaagcttttgaataggcatgagtgatcaaatggaataaagcagctcgataagaacctatacctagagctaacataatataacccaattgagacattgtagaataggctaagctttttttaatgtctctttgagcaagggccaaagtagcccctaataatagtgttattacacctattaaagaaatgaaattcattatataaggtatgactatgaaaagaggaagaagccgagctacaagaaaaattcctgctgctaccatagtagcagcgtgtataagagccgaaataggagtgggtccttccatagcatcaggtaaccatacgtgaagggggaattgtgcggatttagcaaccgcaccgacgaataataaagaagcacacaaagtagcaaataaagaattgaccccattattctggattaagttattagttatttcgagcaaataccgaaattctaaactacctgttatccaataaaaacctaagattcctaacaataaaccaaaatcccctacacgattagttacaaaagctttttgacaagcacttgctgcaattggtcgtgtgaaccaaaatcctattaataaataagaacacattcccacaagttcccaaaaaatataaatttgtatcaaatttgaactagtaactaatcccagcatagaagtattaaaaaaactcatataagcaaaaaatctcaaatatccttgatcgtgatacatatacttgtcactataaataagaaccatgattccaacagtagtaattagtattgacataatagaagtaagtggatcgatcaagtatccaaactctaaggaaaaatcattattgatggtccaagaccatagatattgatagataaaacttccatttatttgttgaatagacagattggctgaaaacaccatagctatacttaagagtaaaacactaggaaaagcccacatgcgacgaatattttttgttgctgtcggaataagtagaagtccaaatcctattgacatagtaactggaagtggaagaaaaggtattatccacgcatattgatatgtatgttccataagaaaagaaactttttttcttataattacaaattgttctcgattcaccttagaaaagatacaaaaaaagaatgatttgaagaattattgtcgagccatagtaattgcacctatcaaggaaactaaaagaattattgaaatgagttcaaacggaagataaaaatctgttgataaatgaatcccaatttgttgaacgttatttattaggtcctgttccataatctggtttgatcttgcagtccaaataattccgtaccatgacgtatctgggatagtagtaattagtgaaaaaagaatagttgtacaaaccatcgaagtgaccccatctccaatggtccaaaaataggaattattggaatattctgaaccattcatgaacattacagcaaatatgatcaagacatttatggctcccacataaataaggagctgtgcggcagctacaaaataggagtttgataaaatatagaataaggatatacaaacaagaaccaatcccaatgaaaaggcagaataaattggattggtaaataatactacccccagaccccctaatacaagaattgaccccagaaatacaacaagaatatcatgtattggtccaggtaaatccattatgtataaaatacaaaataaataactttttcatgaccttaactttaaatggtccaggaaaggaaaaggggttaccccatttttgttttcttgtatataatattgtatatgatacatttataattgaatatggattaatgtagatatagataaaattattgggccaaccttaaaaaagtagttgaaatttgctatttcgaaatcatcacgaaaaatatattttaagtttaaatcaaagagtgattctcaacaatcattagtttttatttgtagttactgactacccaaaataaatggatcctttatatcaaaacaaaatctcaacgatcaacttctcccataatgatatctatactacccaatatcgtcatgatatcagccaatttcattcttttaactagctgaggaagaatttgcaaattgataaaaccgggtggacgaattttccatctccaggggaaaacactattatctcctatcaaataaattcccaattctccttttggggcttccactctcacataaagttcttgtttcgacaattcaaaattgggtgaaggttttttactaataaatctatattcaaaatcattccattcggaattctttgctctatcaaagcgtcgtacttctaaattttcataaggtcctccaggaattccttctagagcctgttgaataatttttatggattccttcatttcaccgattcgtactaaataacgagctaatgaatctccttctttttgccattggacttcccaatcgaattcattgtaacactcataatgatcaactttacgaagatcccattggattccagaagctcgtaacatcggtcctgataaaccccaatttacagcttcttctccaccaataatgcccactccttcaactcgttccaaaaaaatgggattccacgtaataagtttttgatattcaacaactcctgttaaagaataatcgcagaaatccaaacatttatctatccatccataaggtagatcagcagctactcctccaatacggaaataattatgcatcattcgcatacctgtggcggcttcgaatagatcatatatcaattccctttctcttaaaatatagaaaaagggagtctgtgcaccgatatccgccataaaaggtccaagccataacaaatgagaagctatacggctcaattccagcataattactctgatatagctagctctttgaggtacttgaacattttccaattgttctggcgcatttacggttattgcctctgtgaacatagtagctaaataatcccatcgtgttacataaggtagatattgtataattgttcgattttccgctatcttttccattcctctgtgtaaatagcccaatatgggctcacagtcaataacatcttcaccatcgagagtaacgattagtcggagaacaccatgcattgatgggtggtgaggccccatattgactatcatgagatcttttcttgtaaccggtactgtcatatcttttcttccttaattcattattccataaaacgagactcatcaaaacaaaaaattgaatactaccgattaaattagtaatcggtaattgttcttgaatcaaagggttcatctttgtctatttttatttgagtcgaattcataactgtttgaattgtgtaatctccaattattgagattggtaatcgacccaaagcaatttgattataattcaattcgtgacgatcataagtagaaagttcatattcttcagtcattgataaacaatttgttggacaatactcgacacagttaccacaaaatatacaaaccccaaaatctatactataattaagtaattgtttctttttaatatctctttcaaatctccaatcaacaacgggtagatctatcgggcatacacgaacacatacttcacaagcaatacatttatcaaattcaaagtggattcgaccccggaaacgctctgatgtgatcgatttttcataaggatattgaatagttacaggtaaacgatttgtgtgggataaggtaattatgaaactttgaccaatgtaccttgcagctcgtattgtttgttgaccataattcatggacccaattaccatagggaacatattgtagatatacatgaaaaatttgacgtttctttctcttgtttgatagagattatgaatctaaaatagtgttatcgtattctcttattcaatgagcatcttgtatttcatagaaatttggggtaatatagtccttacgtaagggccagcctatccaactttcgggcattaagatacgtttaagacgtggatgattattataagagattcccaacatatcataagattcgcgttcttgaaaatcagcacttctccaaatccagaaaacagacgggattctaggattatttcttggagcaaatacttttatacatacctcttctggtttatctataccatactgtattcttgtaagatgatacacactagctaaaaatccgcctggtgctacatcataggcacactgagagcgtaaataattgtaaccatatacatatgaaatgacagcaatggagtcccaatcctcggtttttatttgtaaagtctctattcctcggtaatcgaagcccaaagatctatgaactagctcatgcttgactagccaatcagataagcgattctgatgcattgtcttgatctctcccacatttgtataagtatttcgcatttacaatgaaatttcaaaagattgacctgctcttttgaaaaatgtttcagaagatatctctgaagtagatggtgattgatagagcaatccttgatcgtaatttccagtatgagtactgtgtcgaacagaaaatttgtgattggtagtaaaatatcgatttttctgttgagatacagttctatcttcaaatatttctcgagatatcttcttacgaagttttgttatagcatctataactgcctctggtttgggtgggcagcccggcaaatagacatccacaggaattagcttatcgactccccgaacagtactataagaatcagtactgaacatccctcctgtaatagtacaggctcccatagcaatgacgtattttggttcgggcatttgctcatataatctcactaaagagggagccattttcattgtgactgtgccggctgttaaaattaggtccgcttgcctaggacttgatcttggtaccaacccataacgatcaaagtcgaatcgcgagcctattaatgaagcaaattcaatgaaacagcaactggtaccatatagaagcggccataaactggagagtcttgaccaattcgaaagatcattcgatgtagttgaaataactgaattgggggttgtttggtcaagtaacggaaactcaatcaaattcataactgtctcaatgtaattttttccttccctttttttgtctgaatactcagatgcaaaatagaaatactttttcttgggtaaaggaacagatgactcgatccatttctgtatcgatcatgatatatgtaataactcgagcatccatttcaaatgcatatcccatttttgcgcagcagggttatgaaaacccacgagaagcaactggacgaattgtatgtgctaattgccatttagctaataagcccgtggatattgaagttccgcaagctgtgcttcctgatactgtatttgaagcagttgttcaaattccttatgatatgcaactgaaacaagttcttgctaatggtaaaaaagggggtttgaatgtgggtgctgttcttattttacccgagggattcgaattagccccccccgatcgtatttctcctgagttgaaagaaaagataggaaatctgtcttttcagagttatcgtcccaataaaaaaaatattcttgtgataggtcctgttccgggtcagaaatatagtgaaatcgtctttcccattctttcccccgaccctgctacgaagaaagacgttcacttcttaaaatatcccatatatgtgggtgggaacagaggaaggggtcagatttatcctgatggtagcaagagtaacaatacagtctataatgctacatcagcaggtatagtaagcaaaatagtacgtaaagaaaagggaggatatgaaataaccatagttgatgcatcggatggacgtcaagtggttgatattatacctccagggccagaacttcttgtttcagagggtgaatccatcaagcttgatcaaccattaacaagcaatcccaatgtaggagggtttggtcagggagatgcagaaatagtgcttcaagatccattacgcgtccaaggccttttgttcttcttcgcatctgttattttggcacaagtttttttggttcttaaaaagaaacagtttgaaaaagttcaattgtacgaaatgaatttttaggtccagagattccttaacatttagtaaaaagtgccgattttttgtccatcgatacaattatgtatgatcaaaaaattctgtaaatccttttgcttgctttgtttatactctttttgttttgcaggacgcctggaattcattacttgtattcctagaggcatacaaacaaatacaaaagaagcaaggatggtaaaaatgaaaaattttaggaaggattactagtcttcctaatcttctattcgacaggaattttcacccgttttcttgtgtcgtcttgtatcaaaataataattatttttttcctgttcatcaaagattactattcctttccttcttttccgggtctatcggaactcctttgtttagattcataagaagtagtggacaaacaaaggaaaaaaggattatgggtgaagcaaatagataagttaaacttctaacttagagaaattattcaaaaaaaagactgttctggttgaagggcggatttgattaaggttctattagtttagtctagaaatgatttgcaggatgtctcatccctagaaatcaatataatttatattggattatagataaaattgattgattcgttctttcttcttgcttccagagagcaaaggatcctttttgtcatttctacaaatataatatctggataactaaccaatttgtaggttatggaatagatcaaagctcattataagaccgcgggccctttccgctctaatcagacaaaggagggtaaggacccgctaagttcttacttttgcatgtctacaatccagcatgagtaaagtatatgattggtttgaggaacgtcttgagattcaggcgattgcagatgatataactagtaaatatgttcctcctcatgtcaacatattttattgtttaggggggatcacacttacttgttttctagtacaagtagctacgggttttgctatgactttttactatcgtccaaccgttacagaggctttttcctctgttcaatacatcatgactgaggccaactttggttggttaatccgatcagttcatcgatggtcagcaagtatgatggttctcatgatgatcctgcacgtatttcgtgtgtatctcacaggtggatttaaaaaacctcgcgaattaacttgggttacaggtgtggttttggctgtattgactgcatcttttggtgtaactggttattccttacctcgggaccaaattggttattgggcagtaaaaatcgtgacaggcgtacctgaagctattcctgtaataggatcgcctttagtagagttattacgtggaagtgctagtgtgggccaatccactttaactcgtttttatagtttacacacttttgtattgcctctccttactgccgtatttatgttaatgcactttccaatgatacgtaagcaaggtatttccggtcctttatagagaagacatatctgtaattgatcatatattggggaggacaatagtatttcattgctacaaatatggattattgaaaaaataagacatgtttttggatacttctcttcaatattgtattccttatttgatatgaatagttgaagtgaattttccgaagagaggatggattgtgtgacttgttataattgatcctattgataatacatagaatggacctgttatctctatcaagatgattctacctcgtcggatatttattctagtatctggagcacggaatatatagaatagatcaagaaaagaaatatttgaactatgattcatacctactattcagaccccgcaaccggactcaaaaaaattaaaataggtatttcctaaatcaaacgaattttattccttcagatttattttgaccgaaggataactctttctctagattttgttgagtcattacatccattcattcaataagtgatcatcaaaggttcttactcgagaacctttgagtttagcttgaggctaaatcatcgtggttctagtatgaatctgaggtttcaattgattcatagggtctcaacaagagaattcctatcaatagtaaaagtaaatctgcaaaaatagggaagagaagattcaagaggcctgtaacgatcaacataaagaaagacagatgagccaacttgatattttttggcattatcatcacaaagaagagattccggatttttttcgtatcttcgaggcaaatcaagcggctaatgaagtttttaactttctattatatatccgttgaaatcagtatttgtgtgttttcgcttgagccgtacgagatgaaattctcatatacggttctcagagggggagttctattgggttacctatctcaatatgggaataacaaagaaacctgacttgaatgatcctgtattaagagcaaagttggctaaagggatgggacataattattatggagaacccgcgtggcccaatgatcttttatatatttttccagtagtaattctaggtactattgcatgtaatgtaggtttagcggttctagaaccgtcaatgattggtgaaccagcagatccatttgcaactcctttggaaatattacccgaatggtacttctttcccgtatttcaaatactccgcacagtacccaataagttattgggcgttcttttaatggtttcagtaccaatgggattattcacagtaccttttttggagaatgtcaataaattccaaaatccatttcgtcgaccagtagctacaacagtttttttgatcggtaccacagtagctctttggttaggtattggagcaacattacctattgataaatccctaactttaggtctttttcaaattgattcaaccgcgaagtacggaatagttacttcaaagtgaatcttccctagatacattaatttgattatgatctattccgcgaaaatacggatcgtgccaaagatgaaaaattgttttctttagaattagaaaaagaagatgaaaaaattacaatggatgtgtgacttgaactattgatttagccatgcagatatatgaatttgccacgttggaattcacaaccaaatgtgtctccgcatccaaccaaaatgtaagtcccctacgtagcagaggataggccggttcgcttgaggagaatattttctatgatcataccgaatcatgttatccacgaacaggctccgtaagatccgtagaatagaataagtgatgtggcatgatccaatgttctattccacttatttattatagtgtgaaaatgcattcatttcctttgcatagattccgatctatgatactatcggagtgaaagaagggatctaaggaagaacagaggctagactttattagtaacaagtaaatactttttacgtaagaaactcgagatatggtggggataaacaccaatcaaaagacatgagacaatccaaaaagcacttgatcatgatcaaatttgtaagcctacttggatattgagcatttgcctataagaacagaattctttgcaatgaatagtttgcaattctggaaaattgaatgtgataaatctttcttacatagagttattaactatgttattgtatgaaataacgattttatattttatatgggtttttttctgttattcctttgattcttgctcgagccggatgatgaaaaattatcggatccataagaattcacctatcccaatgattgaagtttttctatttggaatcgtcttaggtctaattcctattactttggcaggattatttgtgactgcatatttacaatacagacgtggtgatcagttggacctttgattgagtaacatttctttttttgattgacctcctacgggaggaggtcaaattccagttgcaattcaactttgttaagttattttattgtgattcgacatacataagatagacggaatatgcttactataactagttatttcggttttctactggctgctttaactataaccccagctctatttattggtttgaacaagatacgacttatttgaaaatgaattgaataaataattcagaaaaatatttctcgggaattccagatattctatggttctttcccgcaccaattgccaatttttttcttggtcattgagattcacggataattcagattaatatttagggatagatcttacccttttttatcccctcaaacaaaccgaaatggatatagtaagtctcgcttgggctgctttaatggtagtctttacattttccctttcactcgtagtatggggaaggagtggactctaggtatattaataattgattgagtaatcgaattgtatcaattgtttatttttcgaagcttttttttaaactctttcaaaattatactggaaagacaataatgaataataaccattcgatcaaacaatgaatgattactttagttgtatttcaaaacctacgcatgataggaaattttttccaaccgaattccttctaaatattctgtttggataaacctgttcttaccagaattatggatattacaatgagaaaaaaccaatccctagttttttctttatttgtttctctctttctttactttcactgttctaagaacaaatcgttctgctattagattacgacgatacttactttctactggaagtgactagtggttgaggttctacacataataaaattagatctttcttccgctgagcgatccaccacttagactcctagagatttttttatgcttttttgacatgtcatgtttaagcatgaaaaatggtccgagtcccctttactaattcctttcaaaatctgaagaagttaccatagaacttcgtaaatgatctgttaatggctcaatcaatgacttcttgggatgggaaatcaaaaaaattccttgttttgctatagtatattcccatcttcctctattgattcttttgatggatcccggaacctatatataaattataagaaacctagaaattagaagaattggccttcgattattttgggttgatcgaaatcgagtggatgtagcgaaaaaaaatagaattagactgctatttcatctgtatacaatacaactttatatgatcattgtatacaagataataaaaaatactctaaagtgtggtagaaaggactatatatagtcctttctaccacactttatgattccaaccagatcatttcatttaagacttggaattttcttttaatcccttcttcaatcattgaaaaaggattgataagaactatcagattatcctactgcaataattcttgctaagaagaacgcccatgtagtggcaattccacccagaaggtaatgggttactcctacagcacgtccttgtacaatgctcaaggctctaggctgagtagcaggagcaacttttaatttgttatgagcccaaacgatggattcaatgagttcttgccaataaccacggccactgaatagaaacattaaactgaaagcccagacaaaatgagcacctaagaaaagaagaccatatgcagataatgaagaaccataagactgaataacctgagatgcctgtgcccataagaaatctcggagccacccattaatagtaatggaactttgtgcaaagtttcctcctgtgatatgagttaccaccccttgatcacttatagtaccccaaacatccgactgcattttccaactgaaatggaaaattactaccgaaattgcattgtacatccagaatagacctaagaagacatgatcccaggcggatacttgacatgtcccccctcgccctggtccatcacaagggaaacgaaaaccaagatttgctttatcaggaatcaaacgggaactgcgagcaaatagaacacctttcagtagtatcaatacagtcacatggatcgtaaatgcatgaatgtgatggactaaaaaatctgcggttcctaatggaataggtaacaaagctactttgccacctactgctactaactcaccacctccccaggttaagctggtactcgttgttgcaccaggagctgttacgccaggtgctaaagcatgggtattttgtatccattgagcaaagatgggttgtaattgtatagcggtatctgaaaacatatcttggggacgtcctaaagcactcatggtatcattatgaatatacaagccaaaactgtgaaaacctagaaatatacatacccagttaagatgtgatatgattgcatcgcggtgcctaaggacgcgatctaatagatcgttgtatcgagtagttggatcgtagtctcttaccataaaaatggctgcatgcgcagcagcaccaactatgagaaatccaccaatccacatgtgatgtgtgaacaacgaaagttgtgtaccatagtcagtagctaggtatggataggggggcatggaatacatatgatgagctacaacaatggttaaagagcctaacatagccaggttaagagataattgagcatgccatgacgttgttaggatttcatataggcccttatggccctggcctgtaaatggacctttatgagcctctaaaatgtccttaaggccatgaccaatgccccagttggtcctatacatatgaccagcgatcaggaaaagaatagcaatagctaaatgatggtgtgcaatatcgctcaaccacagaccccctgttattggatctaatcctccacgaaaacttagaaattccgcgtattttgaccaattcaaggtgaaaaagggggttgctccctcggcaaaactgggataaagttgagccaaaaggtcccgattcaagataaattcatgaggaagtggtatctctttaggatcaactccagcgtcgagaaattggttaatcggtaaagatacatggatttggtgtcccgcccaagaaagagacccaagtcctagtaaccccgctaagtggtgattcaacatggattccacatcttggaaccaagccaattttggggcggctttgtgataatggaaccaaccggcaaaaagcattaacgatgcaaagaccaatgcaccaattgcggtacaatagagttgtaattcactagttattccagatgctcgccaaatctgaaaaaaaccggaggttatttgtattcctcggaaacccccgcccacatcaccattcaatatttcttgacctactattggccaaactacctgggcactgggtccaatgtgagtaggatcgcttagccatgcttcataattggaaaaacgggcaccatggaagtacatgccactcagccaaagaaagataatggagagttgaccgaaatgagcactaaatacttttcgggagatctcctccaaatcactggtatgactatcgaaatcgtgagcatcagcatgtagattccagatccaagtggtagtatcagggcccttagctattgttcttgagaaatggccgggtctggcccattcctcgaaagacgtttttataggatccctatccacaacaattttcacttctggttccggcgaacgaataatcattaagtcctcctctttccggacaacacatacaaagagacccgccaacagtcaagtttttagtgaacctctgaaagatagatattttatttagttattcactagagcaattatgatattgaagtcgatccgaggcaagtgttcggatctattatgacataacgattaggtgcccaacggacctttatcttggaaaatcctttccggcgtgacaaaaagcaacctagtgtatttatatctgaatgaaggtataagtacctatatggatctacttacatggaacataatatcttttacaaatcacaagatcattcattagaaaagataaaatcattctgatatcttaacaaaatataatgcagaaggaagagatataatgaaattcttaattagatcttctcataggaacgatctttttatttgattgatgaatccaacaaccaaacctaaatgaattaaaaaagagagtggttttaaccaaattttcctgatgtagaggcaatcaagaaagctgcataagtaaatatataacccacggaaaagtgggctaatccaaccaatcttgcttgtacaatggaaagagccaccggcttatctctccatcgaatcaaattagccaaaggtgtgcgttcatgagcccatgctaaagtttcaatcaattcctgccaatacccgcgccatgaaattaagaacataaatccagtagcccaaacaagatgtccaaataagaacatccacgcccataccgataaactattcataccaaaaggattatatccattgataagttgtgaagagtttaaccatagataatctcttaaccatcccatcaaataagtggaggattcattaaattgtgaaacattaccctgccataatgtgatgtgtttccaatgccaataaaaagtaacccatccaatggtatttaacatccagaaaactgccaaataaaatgcgtcccaagcagaaatatcacaagtaccgccgcgtcctgggccgtcacaaggaaaactataaccgaaatcctttttatccggcattaatttagaaccacgtgcatctaaagcaccttttactaaaatcaatgtagttgtatgcaaacctagagcaatagcatgatgaaccaagaaatccccagggcctattgttaagaaaagtgaattactattctcattaatagcatttaaccatcccggtaaccatatgcttcgacctgcattgaaggctgggccattcgttgaagataagagtacatcgaacccatatgaagtcttaccatgagcagattgtatccattgggcaaatataggttcgatcaagatttgtttttctggagtaccaaaagcgagcatgacgtcgttatgaacataaaggcccaaagtatggaaccccagaaacaggctggcccaacttaaatgagatatgatagcttctttatggtctaacattcttgccaatacgttatcctcattctgctccggattgtaatctctaatgaaaaatatagctccatgagcaaaggctcctgtcatgatgaaccctgcaatgtattggtgatgagtatataaggcagcttgagtagtaaagtcttgtgctatgaatgcataagcaggtaaggagtacatatgttgagctactaaggaagtaataacccctaaagaggctagagcaagacctaattgaaaatgaatcgaattattgattgtgtcataaagacccttatgcccacgtcctaatcgaccccccggaggaatatgtgcttctaaaagatctttgatactgtgcccaatcccgaagttagttctatacatatgaccagcaatgagaaaaataaatgcaatagctaaatgatgatgagcaatatcggtcagccataaactttgcgtttgtggatggaatcccccaagaagggttagaatggcagttcccgccccttgggcggtcccaaataaatgactactcgaatcagggttttgggcataaagattccactgacccgtaagaaggggtcccaacccttggggatatggtaatacgtctaagaaattattccatctgacgtactctcccctggatccgggaatagcgacatgaactaaatgtcctgtccaagccaaggaacttactccgaaaagtcctgacaaatgatgattgagacgagattcagcatttttgaaccacgaaacgcttggtttccatttgggttgtaggtgtaaccaacccgctattaaagatatagcagaaagaaataatagaaaaagagctcctgtataaagatcttcattggtgcgtaatccgattgtataccaccactgataaacaccggaataagcgatattcactgggccggtagcacctcctcgagtaaaggcttctacagctggttgaccaaaatgaggatcccaaattgcatgagcaattggtcttacatgtaaagggtcctgtatccatgactcaaaatttccttgccaagccacatgaaacagatttcctgacgtccacagaaagattattgctaactgtccaaagtgagaagcaaaaatgttctgataaagacgttcttcagtaatatcatcatgactctcgaagtcatgtgcggtagcaataccaaaccaaatacgacgagtagtggggtcctgagctaagtcttggctaaacctcggaaatcttaattccataatgcctttcaaatcctcctagccatcaataagatagagccatgctgcgagttgtttcaggccctaaataaacacggacactcaaaaaatctgttgggcaggcagattcacatctcttacaacctacacagtcctcggttcttggcgcggaagctatttgcttggctttacatccgtcccaaggtatcatttccaatacatctgtggggcaagctcgtacacattgagtacaccctatacatgtatcataaatttttactgaatgtgacattggatctataaagttttgaattttgatctggtattatatattgtaatattgtagacaccagacgaaacagtggtttattaaaaacaatcaatatatttcttaaattaatctgtttatgagaaaaggtcaaaacactttgattttgtgtcatcattatttcaatataaataatagtatttcaattctatgatgattatcaatgacagatctcaacttaccctctatttttgtgcctttagtgggcctagtatttccggcaattgcaatggcttctttatctcttcatgtccaaaaaaataagattgtctagatccaatgggaccaaatcttatcaatttatttcaacactgtatcataatacagatattttttagtgcggtacgatatgtggctctttccacacacaaatgaaagaactgttatgtatgcggatacatgatatctgcataaatgtagctggttaaaaacggatcagtaaatatttttaaaagtcaatgtataaaggtaaagtcaaatttgggttattctctcaattccaatcgaatgcaactggatctagtatagtatgcgagatctaaaaacatatctctccgtggcacctgtgttaactactctatggtttgggtctttagcgggtctattgatagaaattaatcgtttattcccagatgccctgtcattcccctttttttaattattgatatgcgaagaaatgaagaaatataattccacatgacgtaactaaaacctcgcctctccctttcaattctttagaatagtaaggaaaaagtgtattgaacctcataaaaaatccggtagatccaagatcaaatttagggcaggctctaagaaatcatagcatagaaagaattaaatgaaatattgggatttaggatagaaataattgatagttagaaagaaattgtattacttaatttctatatatatactatattaatacataattaattagataaaattacaactattgattttttcttcttcttcggttcgaaaatataagacttaagttaaatcgatacaaaatctaaaggaggttcttatccatttgtagatggagcttcaacaacagctaagtctagggggaagttgtgagcattacgttcatgcattacttccataccaaggttagcacgattgatgatatcagcccaagtgttaataacacgaccctgactgtcaactactgattggttgaaattgaaaccatttaagttgaaagccatagtactaatacctaaagcagtgaaccagatacctactacaggccaagcagccaagaagaaatgtaaagaacgagaattgttgaaactagcatattggaagattaatcggccaaaataaccatgagcagctacaatattataagtttcttcctcttgaccgaatctgtaaccttcattagcagactcgttttcagtggtttccctgatcaaactagaggttaccaaggaaccatgcatagcactgaatagggagccgccgaatacaccagctacacctaacatgtgaaatggatgcataaggatgttgtgctctgcctggaatacaatcataaagttgaaagtaccagagattcctaaaggcataccatcagagaaacttccttgaccaatagggtagatcaagaaaacagcagtagcagctgcaacaggagctgaatatgcaacagcaatccaaggacgcatacccagacggaaactaagttcccactcacgacccatgtaacaagctacaccaagtaagaagtgtagaacaattagctcataaggaccaccattgtataaccactcatcaacagatgctgcttcccagattgggtaaaaatgcaaacctatagctgcagaagtcggaataatggcaccagagataatattgtttccataaagtaaagaaccagaaacaggttcacgaataccatcaatatctactggaggagcagcaatgaaggcgataataaatacagaagttgcggtcaataaggtagggatcatcaaaacaccgaaccatccaatgtaaagacgattttcagtgctggttatccagttgcagaagcgaccccataggcttgtactttcgcgtctctctaaaattgcagtcatggtaagatcttggtttattcaattttcaaggactcccaagcacacgtattaactatagatgcgatagataatagaaggcttgttatttaacagtataacatggcttatacaccaatgtcaaccaaccttaacaaatgattaaatccacccaaagaatttgtaaatgaaatgagttcaaatattttattttccgtaatgggtttaccttggtatcgtgttcatactgtcgtattgaatgatcccggtcgattgctttctgttcatataatgcatacagctctagtttctggttgggctggttcgatggctttatatgaattagcggtttttgatccctctgatcccgttcttgatccaatgtggagacaaggtatgtttgttatacccttcatgactcgtttaggaataaccaattcgtggggtggttggagtatttcaggaggaactataacaaatccgggtatttggagttatgaaggtgtggcaggggcacatatagtgttttccggtttgtgcttcttggcagctatctggcattgggtatattgggacctagaaatattctgtgatgaacgtacgggaaaaccttctttggatttgcccaagatctttggaattcatttatttctctcaggggtagcttgctttggctttggcgcatttcatgtaacaggtttgtatggtcctggaatatgggtgtccgatccttatggactaactggaaaagtacaatctgtaaatccagcgtggggcgcggaaggttttgatccttttgttccgggaggaatagcctctcatcatattgcagcgggtacattgggcatattagcaggcctattccatcttagtgtccgtccgcctcaacgtctatacaaaggattacgtatgggcaatattgaaactgtactttccagtagtatcgctgctgttttttttgcagcttttgttgttgctggaactatgtggtatggttcagcaactaccccaatcgaattatttggtcctactcgttatcagtgggatcagggatactttcagcaagaaatatatcgaagagttagtgccgggctagccgaaaatcttagtttatcggaggcttggtctaaaattcccgaaaaattagctttttatgattatattggtaataatccggcaaaggggggattattcagagcaggctcaatggacaatggggatggaattgctgttggatggttaggacaccccgtctttagagataaagaagggcgcgagctttttgtacgtcgtatgcctactttttttgaaacatttccggtagttttggtagatgaagacggaattgtgagagctgatgttccttttagaagggcagaatcaaagtatagtgttgaacaagtaggtgttactgttgagttctatggtggcgaacttaatggagtaagttattctgatcctgctactgtgaaaaaatatgctagacgtgcccaattaggtgaaatttttgaattagatcgggctactttgaaatccgatggtgtttttcgtagcagtccaaggggttggttcacttttgggcatgctacgtttgctttgctcttctttttcggacacatttggcatggcgctagaaccttgttcagagatgtttttgctggtattgatccagatttggatgctcaagtggaatttggaacattccaaaaacttggagatccaactacaaggagacaagtagtctgatacgacattgttgtggtatctttcacctctatttttttgacattgggtatcagagaaatcttgacttgaatcaccttctttgactttttttctttatatgatatggtaaatgatcccaaatgaataggtgtggaagctataattgtaaaccacgatcgaatccatgaaaaccttatattccctgaggaggttctaccacgtggaaacgctctttaatggaactttagctttagctggtcgtgaccaagaaaccaccggtttcgcttggtgggccggtaatgccagacttatcaatttgtccggtaaactacttggagctcacgtagcccatgccggattaatcgtattctgggccggagcaatgaacctttttgaagtggctcatttcgtaccagagaaacccatgtatgaacaagggttgattttacttccgcacctagcaaccctaggttggggggtaggtccggggggggaagttatagacacctttccatactttgtatctggagtacttcacttaatttcctctgcagtcttaggctttggcggtatttatcatgcgcttctcggacctgagactcttgaagaatcttttccattcttcggttatgtatggaaagatagaaataaaatgactacaattttgggtattcacttaattttgttaggtataggtgcttttcttctagtactcaaggctctttattttgggggcgtatatgatacctgggcccccggggggggagatgtaagaaaaattaccaacttgacccttagccccagtgttatatttggttatttactaaaatctccttttgggggagaaggatggattgttagtgtggacgatttagaagatataattgggggacatgtatggttaggttccatttgtatacttggtggaatttggcatatcttaaccaaacccttcgcatgggctcgacgtgcatttgtatggtctggggaggcttacttgtcttatagtttaggtgctttatctgtctttggtttcatcgcttgttgtttcgtctggttcaataataccgcctatcctagtgagttttacggacccaccgggccagaagcttctcaagctcaagcatttacttttctagttagagaccaacgtcttggagctaatgtgggatccgcccaaggacctactggtttaggtaaatatctaatgcgttccccgactggagaggttatttttggaggagaaactatgcgtttttgggatcttcgtgctccctggttagaacctctaaggggtcccaatggtttggacctgagtagactgaaaaaagacatacaaccttggcaagaacgacgttcagcagaatatatgactcatgctcctttaggttctttaaattccgtgggtggcgtagctaccgagatcaatgcagtcaattatgtctctcctagaagttggttatcgacctctcattttgttctaggattcttcttttttgtggggcatttgtggcatgcgggaagggcccgcgcagctgcagcagggtttgaaaaaggaatcgatcgtgatttggaacctgttctttccatgaccccccttaactgagattttcttatttatatctattctattgttcttttctgttctggctcggctaggtggaatagccgagccattcctttttatgaaagataaggggccaggccaaacaaataaagaaaaaatttattcaataaacaaatgactatagcccttggtagatttaccaaagaagaaaatgatctatttgatattatggatgactggttacggagggaccgtttcgtttttgtaggctggtccggcctattgctctttccttgtgcttatttcgctttaggaggttggtttacaggtacaacttttgtaacttcatggtatacccatggattggccagttcctatttagaaggttgcaatttcttaaccgctgcagtttccactcctgcaaatagtttagcacattctttgttgctactatggggccctgaggcacaaggagattttactcgttggtgtcaattaggcggtctatggacttttgtcgctctccatggtgctttcggactaataggtttcatgttacgtcaattcgaacttgctcgatctgttcaattgcgaccttataatgcaatcgcattctctgctccaatcgctgtttttgtttccgtattcctgatttatccactgggtcaatctggttggttctttgcgcccagttttggcgtagcagctatatttcgattcatcctcttcttccaaggatttcataattggacgttgaacccatttcatatgatgggagttgccggagtattaggtgcagctctgctatgcgctattcatggtgctaccgtggaaaacactttattcgaagatggtgacggtgcaaatacattccgtgcttttaatccaactcaagccgaagagacttattcaatggtcactgctaaccgcttttggtcccaaatctttggggttgctttttccaataaacgttggttacatttctttatgttatttgtaccagtaaccggtttatggatgagtgctattggggtagtcggtctggctctgaacctacgtgcctatgacttcgtttcccaagaaatccgtgcagcggaagatcctgaatttgagactttctacaccaaaaatattcttttaaacgaaggtattcgtgcttggatggcggctcaggatcagcctcatgaaaaccttatattccctgaggaggttctaccacgtggaaacgctctttaactaaaaggatctactaaattcatcgagttgttccaaagaatcaaaacggccggttatcaatggaatcccttgtcggctttccgtgaaatactcatttggccgaggacttccaaacacgtcgtaagctaaacccgtactgacgaataaccaacccgcaatgaatagggaaggtatagtaatgctatgaataacccagtatcgaatactggtaataatatcagcaaaagaacgttctcccgtgcttccagacatgctgagctccacaaatttttgtacattcaaaaaggggaatcgattccgtgaaagatgggatcagtaaatagaaaactactgatattgcatctttgtgagatcgtcaattttgtaccaaaggtgtatttcgagtataccgaatcagtatagctatccttcctctggcacagcaacgcagtcttgatcggtaccgaaatgctacacaattccttttttttgttccttatctatggataccttatgttatgctattcaatagatcaacctcaaatttcttataaggtttcctggcttcataaaagtaaggatcttgggaaaatgtaagtcaatgatcaatgggttctaataattcatgaaagatattattgtattgacacaattcaattatatgcgaaatctaacctggttaaattatttgaatcctttcatttcaagtaattggttggatatagtatctactatatcatatttaatgaaagaaagaaaacatagttgtaacaatcaatattcgcgatgcaatcattgttggattaggtccaagacaaagattctttcttgaccaaactacaagtatggaactccatgatatggaaagacagaatatagaacctaaaaggataattgaggcgactcgtcttcgaatcgactttggatccgaaaaagagaacaataaagtcaaagtttttgtttctcgatagatcctttcgatggatggtggaacacctatgttatgtcactctatctttttttagtattatctataatgaccaatgaatcatgaattttccattggaactaaactaattctcttaattggtttttattaccctcgtatctggatggaaacttaggtaagtgttttatcaacatatgtagaaaaaaacatatctaataaagccctttttatcgttggatgaactgcattgctgatattgaccccaagaaagaaacggtaggtacagctagtccgtgaacagccaaccagcgcactgtaaaaataggataggttcgatctatggtcatatggctacacaaaccgttgaggatagttctagatctgggccaagacgaactactgtaggggatttattgaaaccattgaattcggaatatggtaaagtagctccgggatgggggactacaccgcttatgggggtcgcaatggctctatttgcgatattcctatctattattttggaaatttataattcttccgttttactggatggaatttcaatgagttaggtttataagaactatgaagtcctagtctttcaatcaaagaaaaattactttagacttgtatttctagaccattctattctggtagttcgaccgtggaatttatttgtttcggtatttccggaatatgcttactctcaaactctttgtttacacagtagtgatattctttgtttccctttttatctttggattcttatctaatgatccaggacgcaatcctggacgtgaggaataatttctattttgcttttttcaaaattaattcttaataatcttaggatgagattcgaattcgaaagtgatcagagaaagctagagagatgaacccaacccagaatatgaaccgtaaaagaaaacacctattaaaccgatcacaagaataccagttacagtacctatcagccaaagaggaatccttccagtagtatcggccatttcatcaagtggtcatgctagagacaaaaacagtcatggataattatgaggatggtatctatccgaatgggataagagaattactactattctctttctttctcatgcttaatatctttagtttaatcggtatctgtcttaattctgtcctttattcgagtagttttttcttcgccaaattgcccgaagcttatgccattttcaatccaatcgtagatgttatgcctgtcatacctgtactcttttttctcttagcctttgtttggcaagccgctgtaagttttcgatgaaatctttaatactctgctaagatgtattcgataaaaaattctaaaaattgatcaaataagtcttacattacgaaccctcgattccaaaatagaaattcttgtatattgaatgaataaccgcagcgatgaatttggatcagccttttccccgttctgaccttccagtgagtatggactattaggtactccacctaattattgatatgacaagaaatttcgggtaacgaatgaatcaaaatcttattacaaaaaaatttttatggggtgtcaacaaaaatggtatatgtggtaaaaatgggcaatctattccccttaaaaaaaatgatcttggagattgtgtatcaattgaagaaataattggaaaataaaacagcgagtacaaaaatgagtaataaaccccagtatagactggtacgattcaattcaacattttgttcattcgggtttgattgtgtcatagttctataatttgaattaggtttaatcattttgactgactgtttttacgtagataataagtaaaaaagcggtaggaactagaatgaacagtgcagtagcaataaatgcgagaatattgacttccataatttcattattttttttcttcgcaataactcgggatgtaatcccatagagatgagaaatttaactcctgtaaattcattgggatgaattgatcctgatgatactgaataggatcaatattatgaataacaatatctgatctatcaaatcgattcatcgtcgagaattgaatagtataacatgggaagatcctttatccatactaattacgaaatgggattttttattggatcaggaatcccattggatttttcatccccttccacttttttttctataacctactgtcttccttatcttatccccttcctgtgtattatacttacaattatgaggtattatatgaccggtattctatgggtcacacacagacccaaacgaggtgagatgaaaaatcaaatattccaacaaatttactgaaaagggtccttgcttggtctttttttagtatcctctttcttgtatttatttgtactggaatgcatacatcaaaactatgtctgcaatttgaatgagatagattgtttacaattagtcattgaggatacacaaactcagaactagaaaaggtgtggtttaacctgaaatctgtcgaggtaattatgttaagttcattgtccatcgtacaataaacgaatacaatttttgtatgtactcccggtaaaataggatcactctactccgatcaagaacaatcgaaaaagtaagacgaggatgtcttaaaatactgaatatagtaatatcaccgattgtactaatgtacatatgatatgtctctcctttatcaatcgggagtagtggaaagatttgaaattcccgtatccgaaataaaaaacaaaagaaggatccccactggggattagatcttgcttcctgtccccttttccgtgaaaagagagagatgcattgataaattcaccggatcctagtctagtccccgtgttcttcgaatggatctcttagttgttgagagggttgcccaaaagcggtatataaggcgtacccagtaaagcttacaagtaaaccagatatggagatggcgactagggttgctgtttccatttttagtcaagatcacaatggatctacgataagatcgtttatttacaactacaacggaatagtatacaaagtcaacagatctcaaccaatcgttaaataggatttatggaagcattggtttatacattccttttagtctcgactttagggataatttttttcgctatcttttttcgagaaccgcctaaggttccgactaaaaaaatgctttcgaaataatttaattgaagtaatgagcctcccatattgggaggctcattacttcaaatgactattgctttccaattagctgtttttgcattaattgcgacttcatcagtcttactgattagtgtacccgttgtatttgcttcttctgatggttggtcaagtaacaagaatgttgtattttccggtacatcattatggattggattagtctttttggtagctattcttaattctctcatttcttaaacttatttggtatttccccgatccaaaaacaaaaatgtcactccttaagattcaatctgagttctgaaaaaattctatttcaatgttattaattggatctaataaataagattatcctaatctatcttaattggaatcttactttactctggccctgcacaaatatgatccagccgcatatatgatatatgtcatatatgtgtggacatatacgtgcgtatcaggaaggaagaaaatatgtcaccacaaacagagactaaagcaagtgttggatttaaagctggtgttaaagattacaaattgacttattatactcctgactacgaaaccaaagatactgatatcttggcagcattccgagtaactcctcaacccggagttccgcctgaagaagcaggggctgcggtagcagccgagtcttctactggtacatggacaactgtttggactgatggacttaccagccttgatcgttacaaaggaagatgctaccacatcgagcccgttgtcggggaggaaaatcaatatattgcttatgtagcttatcctttagacctctttgaagaaggttctgttactaacatgtttacttccattgtaggtaatgtatttggtttcaaagccctacgagctctacgtctggaggatctgcgaattcccacttcttattcaaaaactttccaaggcccgcctcatggcatccaagttgaaagagataagttgaacaagtatggtcgtcctctattgggatgtactattaaaccaaaattgggattatccgcaaagaactacggtagagcggtttatgaatgtctacgcggtgggcttgattttaccaaagatgatgaaaacgtgaactcacaaccatttatgcgttggagagaccgtttcgtattttgtgccgaagcaatttataaagcgcaggcggaaacgggtgaaatcaaaggacattacttaaatgctactgcgggtacatgtgaagaaatgatcaaaagggccgtatttgccagagaattgggagttcctatcgtaatgcatgactacttaactgggggattcactgcaaatactagtttggctcattattgccgcgacaatggcctacttcttcacatccatcgcgcaatgcatgcagttattgatagacagaaaaatcatggtatgcattttcgtgtactagctaaagcattacgtatgtctggtggagatcatattcacgctggtacagtagtaggtaaactggaaggggaacgtgagatgactttgggttttgttgatttattacgtgatgattttattgaaaaagaccgtagtcgtggtatctttttcactcaagattgggtctctatgccaggtgttatacctgtggcttcagggggtattcatgtttggcatatgcctgctcttaccgaaatctttggagatgattccgtactacagtttggtggaggaactttagggcacccttggggaaatgcacctggtgcagtagctaatcgggtggctttagaagcgtgtgtacaagctcgtaatgaaggacgtgatcttgctcgtgaaggtgctgaaattatccgtgaagctggcaaatggagccctgagctagccgctgcttgtgaagtatggaaagagattaaattcgagttcgaaccggtagataagctagataaagagaaatagaaagataaaaaaaagcgtgtagaatttagtaattcctctttgttctcctaattgattgcgattaaactcggcccaatcttttcctaaaaaaaggattgagccgaataaagaatgagcttcctaacatactatatatttgcatatatctttcatatcatatgtacagacctatatatatatacaagatctaaatagaacgaagacgaaacaactcgatatttctatttttttttggatccaatccataattaatcctcaaagctaagggaagggctccctctatccaatctactcatcctgtatattgtctttttcgttccatgttgcaatatttattatttgattatacgagaacgaattccttatttataaactattttcaatgagaatttgtttttaattaaaaaaagagatctttctcacttattattagttaacaatcctaatcctcatatgcttaattctgataggaaataaaatagtaaaataattattcatcgaatgactattcatctattgtattttcatcaaatagggggcagaagattctttataatacttcgggagctaatgaaactattttagtaaaatttaactgtctcaattctcgagcgatcgcaccaaaaactcgagttccttttggatttccttcttgatcaatgacaaccgcagcattgtcgtcatatcgtattatcataccgttttcacgtttgagttctttacatgtacgtacaattacagctctaattacttctgatctttctagaggcatattgggaactgcttctttgattacagcaacaataacatcaccaatatgagcatatcggtgattaccagctcctatgattcgaatacacatcaattttcgagctccgctgttatccgctacattcaaaagggtctgaggttgaatcatatcatttttatttcaatctgttatttcaatgcaaaagtatgaaagaaaaagaaatattgtccgtccagaaataaataaacctgcgttttttcatccccaataccctttttgtttagttctatcctgaaataagaaattgagttcgtataggcattttgcgcgcagctattgagatagctgctctagctacagtttctgatactccacccatttcataaagtattcgaccccgtttaacaacggatacccaatattcaggggatcccttccccgaacccatacgtgtttccgcgggtcttactgtaacaggtttgtcgggaaatatacgtacccatatttttccaccacgacgcgcatatcgtgtcattgctcttcgccctgcttctatttgtctagctgtaatccaagcaggttcaagtgcctgaagagcgtatctgccgaaacaaatatgattgcctcgacaagatattcctttcattcttcctctatgctgtttacgaaatctggttcttttgggactaagcatagcaattataccaaatgatcaatcgaatttttatttaaccttttagaattgttcttatttttaactgaaagagtttgtcattttttgttttatcactggacagaatgggaagacaaggttgaggttgtttcttaattccatctctactgcagaaccggacatgagagtttcttctcatccagctcctcgcgaatgaaaggattcaaattcaaacacattaatagataatacaccttttattgatattttcttacaaaggaagatgtagatacaagatatacaatacagctagacgtgtgtgtacatttatgtatctttaatgtaatgtttcttttatttttataacgaatcctttcctttttacctctatcaaataaagattgtaatccaataaatagaggtttcgcgggcgaatatttactctttcctgtttcattcgaaggttcaattcatgacctctcagaataaatcaatttttcttggtccgttccgccatcccatccaatgaattattaggattcgttttcaatagaagcctatgcagtcacaggttctgtcgttcccatagcttctccattaatggttaggtccgaactttgcaatggagcttccaacaaaattcgttcccgagtcaatttcctcagtttttattaacctgaaggctctttattattttattctatttatgctttatcacactgccttttatgacgtgattcatagaccatacatattggaatcatatatcattgatatttttgttctttctttctatcatccttccatttatccacatccttttgctttacaacccataatcagatttattttttcttgaaagaatttcagttgctacaaccatatgatagatccattcattcatatagtgactgtttcttgggatctcgacaatacgaagcaataagttggttattagtttattttatataattacaaagtttatagcggggtcagtttttaatcccaacttgaaactataaagaaaaaactaacgagtcacacttatttgataattttattggaaatcatgtaaagacaattcttatttgatatagctatttgcgcaagtattttacgattaagaagcaattgcttcttgcacagattgtgtattaatatactataactatagaataccttattctcacgagttactgcgtttatccgagtgatccacaaacgacgaaaatccctcttttgtctgcctctatctcgatgagaggaaaccaaagctctcattttctgttgagtaatcgttcgagtaagtcttgaatgagcccctctaaaggttgatgcaaataaacgaatttttgttcgacgtctccgagctgtatatcctcgtttaactctggtcattgaatcagattaaagcttaatgaataactaattgatttctcttctttcagtcatccttttccccttccccggtcattaataacaaaacggattattccgatatataaaatatcaattccaatggcttttgctactatgaccttcccaaccacgattttgtattctattccttccagttatttcactggaaataagaaattcgaacgatactatagtgggttccatcgtttctatggttacctcttaaacggtgaggtcctctctatacaccggagcttcttctttcatttaatcaaatttattgttaacttgtatagttcacaccctttggctctacctatctacagtaatagctcttttcacaaaaagagttatccatacagtgacggcatttaattatgaaagttggctaggtagctgaccctgttagtccgtcaagaaaaggagcacaacctttttttatgataccatttcctccgcttaatggataaccatttgctaccaatggggaattgcttcttatttcaaatctagatgattggatttgcaccaatggaaaccataaattccatatacagggtatatgatagatcttctctatctatcctagatactgaaaaaattgtctcatttgttcaaacttatgatctgaacgagtcgcacctaaatcttagattgatctaaaaatgatttagatttatcttttaatacaattgttatatgacatgtcggtctttttatcagataactacgtcctcgagcccgcggtcttaactttttcacaatagtactcctattggcttcgactttactaatgaatgaatcagcttccttcaaacccatattatgattagcatttgccgctgcagaataaaccaatttgagaatgggataagatgctcgataaggcatgagttccagtatcataagtgtttcctcataggaacgcccgcgaatctgatcaattactcttcgtgcttttaaaacagacatacatatatgttgagctaaaacttttacttcacctgaacttgagttctttatcatgtttgattcacatctatttttctatttaagttcttttctctataagaggtggaatagaataacccggttgaagcgtaatgatcatacgtctgtaatgcattgtatgtcccattcttctaccctttccgggtagtcgatgactattcatagctattaccttgacaccaaagaagagttcgacccaatgctttatttctgtcctagttgatccggattcgacattagaagtatattgattgttccccaataaccgaatacttttttctgtaaatactgcatatttgattccatccataaatccattttcttccctatgagttccagtatcgataagaattctagttcttactgttcatatgttatggtatgaatataccataccaattcgttatgtatggatgatgagattccattgatacagagccaattccaatagacttattgaacgttcccattggcgctatttacggcgacgaagaataaaactatcactatattttttccttttcctacttcttcttccaagcgcaggataaccccaaggggttgtgggtttttttctcccaattggggctctcccttcaccgcccccatggggatggtctacagggttcataactacccctcttactacaggacgcttacctagccaacacttagatccggctctacccaaacttttttggttcaccccaacattacccacttgtccgactgttgctaagcagtttttggatatcaaacggacctccccagatggtaatcttaatgtggccgatttaccctcttttgcaatcagtttcgctgcagcacctgctgctctagctaattgtccaccctttccacgtgtgatttctatgttatgtatggccgtgcctaagggcatatcggtcaaaggtagggcatttcccattgatataggaacttctgtaccagaaacaatggtatctccaattatagcccctctgggatgtaaaatatatctcttctcaccatccccatagtgtatgagacaaatgtatgcatttcgattagggtcgtattctatggttacgattctaccagatatgtctttttgattccgtcgaaaatcgattttacggtatagacgcttatgacctccccctctatgccttgcggtaatgattcctctggcattacgacctttaccacaacgatgccgtccatagatcaaattatttcgtggattggatttcacttgactgtctacggctccattgcgtgttctcggggtagaagttttgtataaatgtattttgcttgaagtagattcttcttttttatcaataaaaaccccttcccaaactgtacaagcttcttccaaagcatacggctttctagatgtatatgatgatatctagacagatggatcttatatgaatcgtatgatgaagtaccacatgagtggatatataggaatccaaatctgccgaatcactcatgttatgatcttctacatcctaggtctccccgttccgtcatctggcttatgttcttcatgtagcattcagaccgaatgactctatgaaattacgtcgatacttccacatattacgggtaacgtaggagacatctctatttttcccccgggggatcttaattaccactgcttagctttcaattcgcctctgaccatcaaattaaatgtgaataacccgccctcctctctttgaaatttgaaacaaggggcgcttccggttctgtgcgtgcttcaaacaattttgtcttctccatattaccatatctctagagtcaataattttctatgaggaactactgaactcaatcacttgctgccgttactcaacagttttctgttgaggtctatcccgtagaggtactcaaattggatcagtgatcgatttctaggtttcgtcgtaaacctaattggttacttccaattacgtaaatcaatagttcaaaccgcatggcagttccaaaaaaacgtacttctatgtcaaaaaaacgtattcgtagaaatatttggaagaaaaaaggatatttagttgcagtaaaaactttttctttagcgaaatcggtttccaccgggcattcaaaaagttttttgtgcgacaaacaaataataaatctcaattgatatagcctaaagaacctcaattttgtaagatgaatgttaactaatgtatttttctgtattgaatttctcagatatatagaataagagtgggttctaagtattattttttccctatcacaattgtacttttcaaaagtacaattgtgatagagattgaaactcttttgttatatgcctatcccaaactaattggttttgtaaacggtaaattatatgcacaataaagaatattaatactaattttttgtatttaatgatgaaattgtgatagatatgaaatcctagttatttgttaaaaaatagagaaaaaactatggagttccaactgtttggagagaacttagtttctagtacgtggaagttgattgttaaaaaacccacgctaggtaggaagattccaatatttaaaccacaaaccagtctttattcattgattctaattaatgtttcctaaactatattgattccttgaatctcgacgattcaacatgaattgactattattatttcaagtaaatggccaagggtaaagatgtcagagtcagggttattttggaatgtaccagttgtgtccgaaatagtgtcaataaggaatcgcgaggcatttctagatatattactcaaaagaatcgccacaatacacccagtcgattagaattgcaaaaattttgtcgctattgtcataagcatacgattcatggggaaatcaagaaatagatcgaagggaacatcatgtgttcgatctttccaaagaacaggacaggaagagtaagaacttatataatatacataatatatacaaatcaaacccgattttatgtagatattatttcatgtgtatagatatataatgaaataatatatgaatcaatattttggttggatccgaaatgaataaataatttagagataaggaataaaccttacccttgtctctgtttatgtttcggattggaacaaatcactataattcgtccacgcctgcgaatcagtcgacatttttcacaaattttacgaacagaagcccttattttcatattttttattccttaattctgaatccacttcttggaagagaataagtctcttgaattttttttaacttcaaattgtatacccatggttaaaaaaataacttaaaatgagaacttattcttgggtaaatcaattgcgaaatgcttctgtagagtgtccaatatttgttttacatcttccatgcgaaaatattcaattctcataagatcttcttgactgttactcaaaaggtccaataatgtatgtatattggaccttttgagacaattataggtcctggaaggcaattctaattggtcaataaaaatacaattcaatggaattccttttttgtttttctttagattagttaatctatcttgaaaggttaaaaagggtagagtaaacctgtttttattttcttcgaaattaatgtcctcttcctccgcatatagaaaaggaataaataaatcaatcaaattacgagaagcctcataaagtgcttccttaggagttaaacttccatttgtccatatttctagaaaaagtatctcttgtttttcattcccataagaatgaatactatgattcgcatttcgaacaggcatgaatacagcatctatagggtaacttccatcttgagagtcatttatgggttccatacgatatccgcgatctctcttgatttgtaatccaatacacaaatcaattggttccgtcaggttagctatatgttgtgtcgtgtcaactatttctacggaaggtggtgagatgatatcttgagcggttacgtatctaggaccccttacgcaaatcgacgcgtctctaactccatagagattacttctcaatacaatttctttcaaattttgtaagatttcatgtactgattcctcaatacctactatcgtagaatattcatgtggcaccttcttagattttgcacgtgtgatacatgttccttctatttctccaagtaaggcccttcgcatggcaataccgatggtatcagcttgacctttcataagtggtgacagaatgaaacgaccataataaagacgcttactgtctactcttgattcaacacacttccactgtagtgttcgagtggatcctgctacttcctctcgaaccatactatactagtattattatttgatcatttatttctcttgaaattggtttaattcttattttcaagcttccttcctattaatctggaagttcttctcagatacaaggaaatgattcagttctagagccaaagatcgtagttctcgaatgagcaatcgaaaagattcgggagcaccctcggggttaggtactcttcctccaataatcgtagcaccaagtacttcttgacgagctctaatatgatcagatttataagtaagcatctcttgtaaaatatgagcaacaccaaatccctctagagcccaaacttccatttctcctactcgttgtcccccctgcttggcccttcctctaaggggttgttgtgtaacaagtgcgtaatgtccactagaacgtccatggattttatcatcaacttgatgaattaattttaggatataggactttcctatttgaacaggttgttcaaacggatctcctgttcttccatcaaatattctgctttttccaggatactcgggttcaaatacccatggatttcttgtttgcttactggcttcatataattcggaaaacactagttttctcgaagcctcttgctcatatctctcatcaaagggtgctattctataatgtctctttagcagattccctgctaacccgagcgagcattcaaatatctgtcccacattcattcgtgagggtactcctaaggggttgaagaccatatcaacgggtgttccatcttgcaaatagggcatatcttgtctaggcaaaattttggaaatgatacctttattcccatgtcttccagctactttatcacctactttgatttcacgtttctgtaaaatatatacacaaatcatttctgaattataactggaaccatcctttttctggatccatctcacatcaataacgcgaccccttccacctataggtagttttagagaagtttcttttgcagtggatacctgcataccaagtatggctcgtaataatctatcctccggggaatacgaggattcgctcgctgtctgaggcgttaatttacctactaaaatatcacctgtttctacccaagatcccagcatcacaattccatttctgtccaaattgcggagtaaatgagcctccagatgcggtatttccttagtaattctttcagggccttggcttgtcacatgagtctgaatttcatattttcggatgtgaaaagaagtataaatatcttcatataccaggcgttcgctaattagtactgcatcttcagaattgtaaccctcccatggcatataagctactaatacgttttttcctaaagcgagttccccaccaactgtagccgcaccgtccgctaaaatttgtccttttttaatgcatttaccccgccgaacccgaggtttttgatgcatacaagtatttttgttggaacgttgatacataactaatggaatacttatagtgttcccattacttgataacatgatcttgtgagtatcagtataaatgacctttccttcgtgttcggctataatggaaacccccgaatctagagccgtttggcgttccagcccagttcctacaatgcacttctcggaccgagaaagcggaactgcttggcgctgcatattagaactcattaaagcccgattcgcatcattatgctcgataaaaggaatgagggaagctccaatagaaaaatattggaaaggaaaaatgcttctaagatgaatctgttcccatgcaatagtcaggaattcttgacggtatcgagctggaacaacctgttcttcctgaataccccgattcaaggccaaagaatttcctgctgctaacatataatattcatctctatttggtgataaataaaccatctgtggctctttttctttttgtttctcagatatttcataaaacggactctctatagatccccaatgaccaatcctcacatgaatagctaaggatccaataagtccaacattgattccttcggacgtgtcaattggacaaatacgtccatagtggctaggatggatatctcgtatccgaaaactagcagttcgccccgtcaatcctccaggacccaaataactcaattttcgtccatgaacaatttgtgtcaatggattagttcgatccaaaacttgagataaagggtgtaggccaaaaaacgattcataagtagttgttaatgaagttgaagttaccaaattttgaggagtcggtatcaatttatgcctaattgctccacatatagttcctctaaccgcattttctaaacgaacaagagccaatccgaattgatcctgtaacagatctgctacagaacgaatacgtttatttttcaagtgattcatatcgtcaaatgtacccattccaaatttcattccgatcaaatgatccgcagcaaccaatacatctcgtggtaacaaaaatgtattgttctgaggtatatcaagattcagtctccggttcatatttcgtcgaccgatccttcctaattcacatctttgttgaaaaaatttcttttgtaattccttacataaagactcagaaaataccggatccccacctacacaagcaaattgttgataaaactccaaaatagcattttcttttgacccaatcttttttttctctttatcattcgggaaagacaagaaaatctcagggtaacaaacattatctagaatttctcttagatttgaacccatagctgataatagaactagaatagatattttttgtttcctactcacacgggcccatatccttgcttttctatcaatttcgaattccgatcttcctccccaatctgatattatagtgctggtatagacagaaattccgttatggtccaattccgaacggtagtaaatacctggactttgcaatatttgattgatcacaattcggtatattccatttactatagaggttcctagggaattcattagaggaatgtttccaataaaaacggtttgttcttgcatatctctactggttttccaaattaatcccgcgggtacatataattccgaaaaatatgtgagtgattcatacacagcatctctttcttttatcaagggttctaccaattgatatctttccacaaataatttaaattcaatttcttgatctgtatcttcaatttttggaaacttatgaaattcttccgtcaagccttgattaatgaacctacaaaatccctcaaattggatctgactaaatccaggtattgtggacattccctcatttccattccggagcatcttaatcttatttaatcccattattgactcactattcattgacccatacggatcgatctagcaatgatggaatgtatattctgtttactgaatcacatgaaattttagtgaactccatatatgtatttcatacgtatgaatggaaatgaattgataaaacattcctgaaacaaattctgtcacttacacttatgaagtcttgtatagatacaatataaaaattgatccaatttctacctataattatgatattacgatcagattgggtaccaaggattcacgattaaatctgctttctatgaatgagataaagacagaataatcaggagcactatccagttttactttactttatttagatttagcacacttaatttaatgaaaatgcaaagattttttgatagtagtctaatttatagactaggattgaattgcataatcataaaaggagtagtattttttattcaatatattcaatacaatgaaagattaaagcacggtgattctctatagggtacataagagagagacccgactcggtatctgtgtaccaatttatgttctggggtttacatataatatatattttcttaaaagttaatggttccaatttgccccaaattttgcagtctgtgactggaaatccatttttttttcaatttgaaacagtagaaaagggaagtttttaagtggtgtgtgttttgagatacaatcaatcgaagagactaaactagatcccataagaaacaagaattcatttttggaaagagattgaaaagggataagcacaagagattcaagatcaaaaaaaaataaaaaagaaaaattaatattttaatcgattttggatcggatttttagatagtgtatgagtaggcccgacaaaacccttgtatggcttcttctatttctcgataaaaagaaatatgaccaagagtggttcgaatgtatatacaacggatttctttttttacacttcctactattagatagtgcccataaatctcatgataagtacccaaagattcatattgaacttcgatgggaacttctcttgacccaatgacacgttgatctagtctccatcggagccacaaaggactatctaaactgattcgtttccgccgataagctccaagtgcgtcataggaactacaaaaatgcggttctttctctttcgtatacttataattattattgtcaactgtttgatatggattatacctatttgcacaaatacctcgacgattcccgatcgttaatacatagagtccaataagtatatcttgggttggtacggaaatgggatctccaatagctggagacaagagattcatatgagaaaacataagtaaacgggcctccgcttgagcttccaaagataaaggtacatgaacagccatttgatccccatcaaagtctgcattgaagcccttacaaactaatgggtgtaaacaaatagcacgtccctccactaaaatgggttggaacgcctgtatgcctaatctatgcagggtgggtgctctattcaacaatacaggatgcccctgcataacttcttgcagtatttcccatacaatcggttctttttcccgaattttacttttagcaatccctatgttagaaacaccatgttgcccgattagaccacgaattacaaatgtttggaaaagttctattgctatttctccaggtaatccgcattgatgtaatgaaagcgaaggacccacaacaatgacggaacgccccgaataatcgacccgtttaccaagcagagtctcacgaaatcttccctctttcccttcaattacatccgaaaatgacttgtaaactttattatgaccatccctcattggttgcccgcggatcccattatcaagaagtgtatccacggcttcttgtactaatttctcctgacacattactaattcccctggcgtagatctacttgttgctaatagatcggtaagagtattgttccgatagataactcttctatagagttcattaatatccgaactcattagtttacctccatctatctgaatgattggtctcaattcaggaggaagaactggtaataagcacaaaaccatccgttccggttctacctttgttcgaataaaatgtttagctaattccatacgtctaaccaaaaaatcctttcttcttctaattttgctatcttcccattcatttccagtaaacccttcgtctcctaattctttccattctgccaatgaattatctataataattcgaaaatccgaatcagctaattgttctctgatagcacctgctcctgtagagatttctcgatttcgaaatatctcgaagcctgaggtagtaaaaaaaagtgggatactgtatttccgggattggatttcatattcgaatgaacctcgtaatcgtaagaaagtaggttttttagctatgggcctagcaaaagagaaatcgcagtatactaggccttctaattgcttaaggggtttatctaaaagattcgcgatataactaggaagacgtttcaaataccacacatgagtcactggacatgcgagtttgatgtatcccatttgatatcttcgtatccgagagtcaacaaattctaccccgcattgttcgcaaaatttcgggtcttctttttcagctctgatcgctcgataatttccacaagcacaaattccactttttatgggtccagagattctttcgcaaaacaatccatctttttctggtttattggttttataatgaaaagtatagggttttgtcacctctccaactatctctccattaggtaggattttgttggcccaagcctttatttgttgaggagaaaccggtccaattcgaagttgttgatgtttataccggtcaatcatagaatagaaatgatgattcattcagatataggatcggattccccccctttaaaatcggacgtgaaggtttcctctcatccggctcaagtagttacaccaaataaaaaaggaagtgaaactttgttcaaaaccctacaaagagctactccttactcaagttcccagtaagaaccaacctttcattgattccttcttcttttatgacaaaatggaaatgtgaaattattgagtagtctattccctgatgaatttttaaaaagaaatactttggaattcgtaagggatttatttgtctatatatcgttccattcgatcttttaggtctctactcacctcgatggttatgccatgatgtcccttgaagcatatatgcgatagatagactcctgtaaccatgctatatttgcttgcttaaacagaatttctctctaaaagaaatggaatggctaattccacgaaaaaatcttttttcacgaggtataactggctattttatacggaatcgaccatggatcaattcccctttcatttggaagtattgaatacacccataattctattctgagtttcatgttatttcttccaaaacacatgtcagagttgggggcatcccaatcggattgaatgggatgacagtttctcaatccgaatctgtaaaatgaaaatcttgatcaaatcaaaattgtttgtcgcgtaagaacaaaataattctctatggtgtaacaaaatatctcccatttccaactcgaatagattttttcttttgatttccaaataaatgtttttgtcttgccttgaacggtgcacaaatttttggaatccggtaccaacaggtataatcccccccagaacaacgttctctttcaggcctttcaaccaatcaatacgacctcgtagagcagcttttgctaaaactcgagcggtttcttgaaaacttgcttcggatatgaaactttgagtattcagagacgctcttgttattcccaatgagattgcccgataacagatcgattcgtccaaagcgcgccctgctcgttccgctcgcaacaatccgattaattctccaggcgaaaaaacattagacattccatcttctgaaaccaacacttttgatgttacttgacgtacaataatctctatatgtctattatggatctgtaccccctgggatcgataaaccttttggatcttattaaccaaagagatacgactttgggctatggttagctcagctccaatcaaaaacccccaggggatcccaagaattcttggtatacactcgttccaaccttcaactctcctttcgaggttcatcgatattgaatcaatcgaacgcacttctaagatttgttctacttttggaagaccttgcgttatgtcaccagatctcgatttttcatatataaatgtaactaatgtatctccttcgtaaaggattttcccataatgaccatgaacagttgctccaggagtagccaaataaggcttagccgatcttataactaaaaagtcgacattaacaattaaaatttgaccagatttttttacgtgtggttcgtatttaaatagacatacattttcacaaataaattgtccaaggctaattttgattgatgtctcttcacaataatcatgatggagaaagcaccaattcaaatggaatgggttcaaaatgatgttactgcatagatcgggattataaatcctcctattttcatctattaaacagtatttaagtacttgaagtacttggaaaatctgtttcaaattgtcaagtagcaaatatttctttaacacgatctgattatgagttattaaatggtaagatgaataaaaattcgatattttaggtacaatagcgcctaagggacctaaataattcctaattggaatttggggatccgattcttttgtcacattgttatatttcgaactattgaatggaccaattcgagaacaattggatgatgacaaaattatcaaagattggcattccttatttcgattcaacaacataccaatagttccttgatgttggctaagtgattgaatcttcaccttggaataaaaaggattgatattggtgcaatctaatccattatcgggaatcaatccggaacttgccctatcatacctttttccagtatacaaaatagtggactcaattcttatgaaatcgcgaattagatcatttgcccttacctcaacaaaggaagcatgaacctcttctatagaaccattttgttcttggtcccaattcaatactaagcaagtccgaactaattgaatacttgtgtgataaattcctcgaattgacttgccatttccataaaggatataattgacaactctaaattggacattatccttttcctgcaagatatcacgagggaaaagtgttgctaaatttatcccatcggatatttcatatgtgactacgggtcgaaccaaaacaaaatactttttcttggtaggtgtgatccgttgaacatagatccaatttttccatttttttgattctttagaattttttttttccgtttctggtggtataaaaatgccgctgtgcctggatatcttatctgtctctccaggaaaatgaatatctccagaaaagattttaagttcaatatattttttttttctctccactcggactaatccgcctactcggcttcttgtatttaaagccagtcgtgtatctactccaatgatactattgttccggaccattattaatgaggatcccggtaagatatgcacttcttcgagaatgaaaaaaaaccgatctactttcatttggtatttcggactaaattcttttgctcctcgatactcaatcaaatcttctttttttatgattgaatccacctctatggtcccatatttagtaattccggaactgcttcttctgtatcgtggatcatcaaaataagcaagaatactatttccacgtaaaataccatttatgggtatttcaatcgaaataccaaaacagggtattagttcttgatcatattgtaatgggatgatgaatctatttcttcgctttttcgccaagaaatcaggattctcttggagaatagaaggatatattaaattccaatgatcattggatatgattcgatcaagtcttgaatagtcaagaatttccctatcttttttaccagaagtatccaacaatttatgtcttacttgatcattagtcattgaggggttagagatatatctttcttcaacagaaagagaataaacattcatttgatcttgatccttgtggagcgaaaaagacactatactggatctgcacggacctcctgctaatatccacaaatggcttgtttttggtaatagatgaacattaccatatgtatattcaggtgcatggtagacatcggtactccagtgcatttctccctctgattcagaataaatatgttttcgtaccctctctttaaaatgaaaagtggacgttccagcacgaatctcagcaatcacttgttctgattctacatattgatcattttgaactaaaatcaaactttttggcggaatagtcacattatatataatatcccgactctcaatagttacatacaagtctatagaacatagaaaagcaggatacccatgacgggtacgtgtgggatgaaccaaatcctcattgaatttgatttttccattagaaggagctcgtacatgttcggcagtaccacctgtgaatactccgccggtatgaaaagttcttaatgttagttgagtccccggttcgccaattgattgacccgcaataatacctacggcttctcccaattcgaccagatcgccatgagtgggactacgaccataacataattgacagatccaagatgtactcctgcaagtaaagggggttcgaatatatattggttgtgctcgaaaggttatgaatcgattgacaagtccaatcccaatatcttgatttcgagtggcaatacatcgtagaccaatatatatatcgtctgctaatacacgaccaattagtgtttggacaaaaattttttccgtcatctcattttgaggactcacggaaatacctcggatagtaccacaatctcttctacgtacaataatgtgttgaactacttcaacaagtctacgtgtaagatatccagcatctgatgttcgtatagcagtatctacaactcctttgcgggctccgtagcaggaaattatatattctgtcaaagaaagtccctcgcgtaaattgctttgaatgggtaaatcaatcatttgtccttggggatctgacattaatcctctcatacctactaattggtgtatctgagatgcatttcccctagctcccgaaaaagacattagatagactggattagagggatcagtcatccgaaaattaggattcatttcttgtctcaaatattcacttgtagcataccatatctcaatagattgacgtaatttttctaccgcatgtacattcccataatgatggtgtttctccaaaataaaactttgttgttcagcgtcttggactaaccatcccttagaaggtattgttaaaagatcatcaattcctaatgaaatagatgtaacagtggcttgatggaaacccaaagtctttacttgatccaggatatgtgatgtatatcccattccgaaatgatctattaatctgctaataagtcgtttcatagcagttccatttatcactttattgtgaaagaccagatcggcccgttctgccataagtacctctatattctgctgagtaggattcgacaatggacctgagtcagtgattcgaaatttcctcaattttgattcacgcagaaagaaaattggaaaacccgatttcttagtctacacacgtctttttttaggaggtcgacatccattatgtggcataggtgttacatcacgtacgaagcttaataatataccacttctacgaatggctcgtaatgctgcatctcttccgagaccaggaccctttatcataacttctgctcgttgcataccctgatcaaccactgtacgaatagcatttaccgctgtggcttgagcagcataaggtgttcctcttcttgtgcctttgaatccagaagtaccagcggaggcccaagaaactacccgacctcgtacatctgtaacagttataatggtattgttgaaactcgcttgaacatgaataacgccttttggtattctacgtccattcttacgtgaaccaatacgcccattcctacgtgaaccaattcttggtatagcttttgtcatattttatcatctcatatttatgagtcagaaatatacaaaaaaaaagatacggagatatccatttcatgttaaaacagatcctttaccttatttttacattttggaaagttctttttagaagaaatacaccctagtacatgttcctcgacgctgaggacatcctctaagagcgggagattttgtaacatttcttattggctgtcttgtgtttctaataagttgtttaatagttggcatttattttggctttttaaccccatatttagaacgcccttgttgacgatcctttactccgacagcatctagggttcctcgaacaatgtgatatctcacaccgggtaaatccttaacccttcctcctcttactaatactacagaatgttcttgtaaattatggccaataccaggtatataagcagtgatttcaaatccagaggttaatcgtactctggcaactttacgtaaggcagagtttggttttttgggggtgatagtgtcgtatgtatatatatgtatatatagaataaaatgggttggtttagatcgatcttaacctgatgattgatcatcatgaattatttctattcaatatcaaatcacattgaattatggtttgaaataaaatagatggaaaagttgacagataagtcacccttactgtcactctacagaaccgtacatgagattttcacctcatacggctcctcgttcaattctttcgaagtaattggatccttttcttcgttcgagaatctcctcccttcctccactcaagagtaactaagaccaattcagtcacgttttcatgttccaattgaacactttccatttatgatcaaaggagatctttttaccaaacatatgcggatcaaatcacgatcttataattctaataagaacaagaaatctttctcgatatcaatccctttgcccctcattcttcgagaatcagaagttttcgagtttcaatttgttcattttgaatctgggctcttctatcttctacttatttttgatattgacttattttgctttattctttatttcatttcgattttttcctcttcctctatccctatcccataggtatagcgtttgaatcaatagagaaccttttcttctgtatgaatcgatattattacattccaatttcttcccgatacctcccgaaattgacgggttagtgtgagcttatccatgcggttatgcactcttcaaataggaatcaatttgatgaaagatcctggctttcgtgctttggtgagtcgtccgagatcctttcgatgacctatgttgaagggatatctatatgatccgatcgattgcgtaaggcccgcggtagcaatagaaccggggaaagtatacagaaaagacagttctcttctattatattagtattagttagtgatcccggctctgtgagtcctttcttccgtgatgaactgttggcaccagtcctacattttttctctgtggaccgaggagaaggggggctcagcgggaagagggttgtaccatgagagaagcaaggaggtcaacctgcttcaaatatacaacatggattctggcaatgcaatggagttggaccctcatgtcgatccgaatgaatcagtctttctacagaggtaaatctttgcctgctaggcaagaggatagcaagttacaaattctgtctcggtaggacatgtatttctattactatgaaattcatatttcttgtatgtgttcctaagaaaaggaatttgtccatttcatttttcggggtctcaaaaaaagggcgtggaaacacataggaactcttgaatggaaattgaaaagaaatgtagctccagttccttcggaaatggtaagatctttggcgcaagaagaaggggtgatccatatcatcttgacttggttctgcttcccctctttttttaaccgagtcgggttcttctcctaccagtatcgaatagaacatgctgaacaaaatcttcttcatgtaaaacctgctcgatttagatcgggaaaatcgtacggattttatgaaaccatgtgctatggctcgaatccgtagtcaatcctatttccgataggagcagttgacaattgaatcccattattttactatccataatagtgcgaaaagaaggcccggctccaagttgttcaggaatagtggcgttgagtttctcgaccctttgccttaggatttgttaattctatttctcgatgggatggggatagggtggatctcgaaagatatgaaagatctccctccaagccgtacatacgactttcatcgaatacggctttccacagaattctatatgtatctatgagatcgagtatggaattctgtttactcactttaaattgagtatccgtttccctccttttcctgctaggattggaaatcctgtattttacatatccatacgatcgagtccttaggtttccgaaatagtgtaatgtaaaaagaagtgcttcgaatcattgctatttgactcggacctgttctgaaaaagtcgaggtatttcgaattgtttgttgacacggacaaagtaagggaaaacctctgaattcaatattggaccttggacatataatagttccgaatcgaatctctttagaaagaagatcttttgtctcatggtagcctgctccagtccccttacgaaactttcgttattgggttagccatacacttcacatgtttctagcgattcacatggcatcatcaaatgatacaagtcttggataagaatctacaacgcacttaccaacttgatcttgttgcgcccggtaacaaacatgcataaaccatttctcgaagtatgtgtccggataggccaaagtctcgatagttagctctaggtcttccagtcaaaaaacaacgtcgatgaagacgtataggtgcactattacgtggtggggattgcaattttccatgaatttcccatttttcactcaacgatgaaactttgcttattttttcttttgaggatcggcgaatcaaatgatatttctgttccaaatttttccgcttgttctctctctgaatcaaactttttcttgccataatgttcagttcctattattatcacagttcggatcctccctctccatcgaaacaaatgaaattgttgctgatatagtacataaaaaaacaataaataactaaattaacgagtttttggctctcgaatatccaactgaccaattaatttcttataacgtactctatttttctttgacaaataagccagcaaccgttgacgttttcctagaatttttcgtagacccctttgcgataaaaaatcttttttgtgcaattccaaatgtgaagtaagtctccgtatcttattggtgaaactgaatacttgaaattcaacagaacctttgttttcttctttttcttcttgtggaataatggaaatgaatgaatttttgaccataaaaaatttttctatccttttttttctttttcatggatttttccgatcaggaattatgccagttattttaatctagtacacacaaaaatttggatttattcatatttctttattttatccaaatcaaatttgcaatttgatttggatagaaagggataatatgtttccgcattaacgaaagaaaagaatttatttctatctaaaaggattcccctaatttatttattcctatatccattcaatctgtcatacatctttcggctttcatataccgaaaatgtcacggaatatagatatgatataggaaatatactattaaactcgaagttctttctttaaagaattctgccttccttaaaatatcataaacagttcctgtaggttgagcacctttttcaaggaaatatagaatagcaggaacatttaaataagtttgattctttatcggatcataaaaacctacttttcgaagatctcttccttctcttcggaatcgaacatcaattgcaacgattcgatagatggcacgttgctttctaccacatcgtttcaaacgaagttttaccataacattcctctaattgaaatttcaaagcggtatggaattgattcaatataaaatcatgaatagtcattggttcaaccggtatataatatttctatctacggataaataagtatttatccggataagggtcagcaaggatcgaatttatttaatttaaccccatatcatatgaattgaatgaaaagatattcaattttacccacatttgacacttgattccgtttgtgagaaaccaaacgaattctcagatatgattcttggaaccattctgaaaattaataagatttttcccttaacaaattattgtttcatgtggaatgcagtgtgatcccatctttcgtttcatgaaaaacgatattgggatagatgtaaataaacaatgccccccctagaaacgtataggaggttttttcctcatacggctcgagaaaaatgattctaatttctgtatataatagtggatccataaaatcatgaattttactatgatttgaattgagtacttttttttcttccttacagaaaagaagaaatctcattcatactcataactcaagttgggtaattctgacaagaaaatccttagacatttattgagccgtctctaaactcttttgtttgtctcatttcgaatctatttttattcctcagtctgatccaattgttgagacaattgaaaatagtgtttccttgtttcggaatcctttatccttgctttgtgaaatcattgggtttagacattacctcggggatccttattcctttcaaaatggcagcaacataccttttttgttatttctttctatataaatagaaaatgattgattcccttgtgatacacttttcatcgaaatagttttaccaaaaatttgacttttctctgaatttgaacctttcgatttagaatttatatatacttacagagttggtctaacttattgattttcactaaccctagattctttcccttgaaaaatgaatcaatcccttcttctcgagcttcatcatgtactatttacttataacccaacacaaattaggttccgggcagaacaaactatgtcgagccaagagcatcttcattactatagaagatggcggatgtaaaaatccacagccgaccatgtccttcaagtcgcatggataaatccaagcaaccttttcgtaaatacaagcgatcttttcgtaggcgtttgcccccaattggatcgggggatcgaatcgattatagaaacatgagtttaattagtcgatttattagtgaacaaggaaaaatattatctagacgagtgaatagattgaccttgaaacaacaacgattaattactattgctataaaacaagctcgtattttatctttgttaccttttcttaataatgagaaacaatttgagagagctgagtcgatcccaagaactactggtcctagaaccagaaataaataggcatactcctcaattgactcaaaaatccaattggaactcaagctcagattgatttttgttcgaaaaggcctagaatcctgatttgattcttgtgttataatataagaaacaaaaatgggggagaagaagaaatatttttttattgaaacatgttcattcatttatttatttttattctatatcttcccggagttcattctccggggaattccatttcaatcattcctgtatattacttttgaatctcctttaacgacgagatttattatcgtttcttgcatgtctcgcgaaagtcagagtaggcgcgaattctcccaatttgtgacctaccatacgatctgttatataaataggtaaatgttcctttccattatgaatagcgattgtatggccaatcattgtgggtataatggtagatgcccgagaccaagttactattatttctttctcctccctcatgttgagtttttcaatttttcccgctaaatgattagctacaaaaggatttttttttagtgaacgtgtcacagctgattactccttttttttacatttttaagattggcattctatgtccaatatctcgatctaagtatggaggtcagaataaatacagatgaatggaaaaaagagaaatttagctagataagatcaacgatttcttatatagctagaacgaccttcacaaattgcggatactaatttgttaagaatcaatcgaattgaagctatagcgtcatcgttggctggaatcgaaatatctgcaagatctgggtcacaatttgtatcgattaaacaaatcgtcggaatccccaaaatggcacattctcgaagagccgtatattcttcttgctgatcaacgatgatcacaatatcaggcaatcccgtcatatatttgatcccaccgagatatgtttgcaaggtagataattttctcttcaacattgctgcatctcttttggggagacggttgagtttccccatcttttcttctgctcttaagtccctgaacttataaagtctcgtttccgtagtggaccaattcgttaacataccaccgagccatttttgattaataaaatgagaccgagcccttattgcagctgatgctactgaatccgatgctttatttttggtaccgacgattaagaagttttttcccctacttgctgcatcaaaaactaaatcacaggcttctgataaaaaacgagcagttctagcgagatttgtaatatgaatacctttacgctttgcagaaatgtaaggggccattctaggattccatttcttagtaccatgaccaaaatgaactcctgcttccatcatctcttccaaattgatgttccaatatcttcttgtcattttttcccacacttcctttttgttttttctttttcgtattattaacaaagagacgaggtaccttgaaataaataattgttccgatggaaccttctaccggggattgaccattgatacacggcacaaaccataaatttttttaattacttattttatttattaccaaatcaataatcagaccagtatagttaaaagatagttaaagtgaaaatgaatccgctcttattcctcaaatatccaaatttttatacctaatactccataaatagttcgagttgtataggaacaatgatcaattttagcgcgaattgtttgtaggggaactctaccctctctgatccattcgacacgtgcaatttcttttccgtcgatacggcctgctatttgcacttgaattccttttgtatccgtttgttcagttaattcaatagcttttttcattgcttttcgaaacgaaactctattttttaattgtaaagctatatattctgcaagaatattaggttgtccataaggtttttcaactcttgtgatagcaatgttgagtctccggtttacagaatgaaactccttttgtacattcatctgtaattcttcgattcctcgtgtttgcccctctattaataaatttgggaatccaatatagattatgacttggatcaaatcaatttttttttgaattgttatacgtgcaattccttcgaaacctgaggatattttcctatttttttgtacatagttcttaatacaattccgtattttttcatcttcctgtaggcccccggaataattttttggttgtgcaaaccaaaaggaatgatgactttgagttgtaccaagtctgaaaccaagtggatttattttttgtcccatattttgctattctattttttcatccatattttttatatgaattcaagtctgacgagaataatattctacgactaacaactcatttatttgtaaaccgatccatttactatctattatttgatttactaatcctttatattggaatgagtcaatagtcaaatgttttggcaattcctcatgggcggatgaagcaatataattttgaatcagaccttttgatctttggttatccttcgcagtaataatatctcggggtttgcaacgataacttggtatatccactatacgaccattaactagaatatgtctatggttaactaattgcctggctccggggatggtcgaagccatacccaatcgaaagaggatgttatccaaacgcatttcaagtagttgtagtaaaacctgacccgttgaccctttggcttttccagcgatatgtacatatctaagtaattgtcgctctgtcagaccataatgaaaacgcaatttctgtttttcttctaaacgaatacgatattgcgattttttcccagaacgcaattggtttttaagatcacttccggatctaggtcttttactagttagtcctggtaaagctcccagacggcgtatttttttaaaacgaggtcctcggtaacgagacataaagactccttttttattttattgaaatttttacacaataaatttaaactgaactaaaggataaacaaagcaaaatcgaaaaatgaattgtatcaacatctagattttatatatatagtgaggctccgtttgatgatttgttctgtagagatctaattgctctaatcacgacataatagatcgccgatccagcattttttttgaagaaaaggagagattatcaatctcggaaaaatagatagagaaattaacgaaaatgtgcaaaagctctatttgcctctgccattctatgagtctcttcctttttgcgtatggcatcgccactccctttggcagcatctactaattcggaacttaatttgaaagccatatttcgacccggacgctttcgggatgctcctaataaccaacgaatggcaagtgcttttccttgtgtagatcctatttcaataggaacttgatgagtcgatccgcctacacgtcttgcttttactgctatatcgggagttactccacgtattgcttgacgtaaaacagatagtggatttgtttctgtcttttgttgaatctttttcacggctcgatagataatttgataagccaatgatttttttccgtgtttcataatacggttaaccaacatgttaactaatcgattacgataaattggatcggattttggagttttttcttctgcagtacctcgacgtgacatgagcgtgaaagaggttcaagaatcagttttctttttataagggctaaaaacgaatcacttaccatatataacacaaaatttctcctccaattctttctagtcgagcttctcgatctgttattatacctcgagaagtagaaagaattacaattcccattccacctaaaatcttaggaattcgttgatagttggaataaattcgtaagccgggtcggctgatacgctttaaaatggttctagttttatatattccttttctggtttttctatgtcgcagagttgaaaccaagaaatatttgttactctcctgatgtttccgaacgttttcaataaaaccttctcgtagaagtattttaacaatgttttcggtgatatttgtagatgctattcgaacccttccttttttatccgtgtcagcatttcttatagaagttattagatcggcaatagtgtccctacccatgacgaactagaattataggttcctcctaattttgatataatcaacatgtttcctataaagtatatacgtgagacacaatctactacctactatatcatagtctcatctactactattctcatggagagttcgatcctggctcaggatgaacgctggcggcatgcttaacacatgcaagtcggacgggaagtggtgtttccagtggcggacgggtgagtaacgcgtaagaacctgcccttgggaggggaacaacaactggaaacggttgctaataccccgtaggctgaggagcaaaaggaggaatccgcccgaggaggggctcgcgtctgattagctagttggtgaggcaatagcttaccaaggcgatgatcagtagctggtccgagaggatgatcagccacactgggactgagacacggcccagactcctacgggaggcagcagtggggaattttccgcaatgggcgaaagcctgacggagcaatgccgcgtggaggtagaaggcccacgggtcgtgaacttcttttctcggagaagaagcaatgacggtatctgaggaataagcatcggctaactctgtgccagcagccgcggtaagacagaggatgcaagcgttatccggaatgattgggcgtaaagcgtctgtaggtggcttttcaagtccgccgtcaaatcccagggctcaaccctggacaggcggtggaaactaccaagctggagtacggtaggggcagagggaatttccggtggagcggtgaaatgcgtagagatcggaaagaacaccaacggcgaaagcactctgctgggccgacactgacactgagagacgaaagctaggggagcaaatgggattagataccccagtagtcctagccgtaaacgatggatactaggcgctgtgcgtatcgacccgtgcagtgctgtagctaacgcgttaagtatcccgcctggggagtacgttcgcaagaatgaaactcaaaggaattgacgggggcccgcacaagcggtggagcatgtggtttaattcgatgcaaagcgaagaaccttaccagggcttgacatgccgtgaatcctcttgaaagagaggggtgccttcgggaacgcggacacaggtggtgcatggctgtcgtcagctcgtgccgtaaggtgttgggttaagtcccgcaacgagcgcaaccctcgtgtttagttgccaccattgagtttggaaccctgaacagactgccggtgataagccggaggaaggtgaggatgacgtcaagtcatcatgccccttatgccctgggcgacacacgtgctacaatggacgggacaaagggtcgcgatcccgcgagggtgagctaactccaaaaacccgtcctcagttcggattgcaggctgcaactcgcctgcatgaagccggaatcgctagtaatcgccggtcagccatacggcggtgaattcgttcccgggccttgtacacaccgcccgtcacactatgggagctggccatgcccgaagtcgttaccttaaccgcaaggagggggatgccgaaggcggggctagtgactggagtgaagtcgtaacaaggtagccgtactggaaggtgcggctggatcacctccttttcagggagagctaatgcttatgcttgttgggtattttggtttgacactgcttcacacccaaaaagaagcgagctacgtctgagctaagcttggatattgaagtcttctttcgtttctcgacggtgaagtaagaccaagctcatgagcttattatcctaggtcggaacaagttagttgataggatccccttttttacgtccccatgtccctcccgtgtggcgacatggggacgtaaaaaggaaagagagggatggggtttttctcgcttttggcatagcaggcctcccattgggaggcccacacgactcaaacgaggaaaggcttacggtggatacctaggcacccagagacgaggaagggcgtagcaagcgacgaaatgcttcggggagttgaaaataagcatagatccggagattcccaaataggtcaacctttcgaactgctgctgaatccatgggcaggcaagagacaacctggcgaactgaaacatcttagtagccagaggaaaagaaagcaaaagcgattcccgtagtagcggcgagcgaaatgggagcagcctaaaccgtgaaaacggggttgtgggagagcaatacaagcgtcgtgctgctaggcgaagcggtggagtgctgcaccctagatggctagagtccagtagccgaaagcatcactagcttacgctctgacccgagtagcatggggcacgtggaatcccgtgtgaatcagcaaggaccaccttgcaaggctaaatactcctgggtgaccgatagcgaagtagtaccgtgagggaaaggtgaaaagaacccccatcggggagtgaaatagaacatgaaaccgtgagctcccaagcagtgggaggagaaagtgatctctgaccgcgtgcctgttgaagaatgagccggcgactcataggcagtggcttggttaagggaacccaccggagccgtagcgaaagcgagtcttcatagggcgattgtcactgcttatggacccgaacctgggtgatctatccatgaccaggatgaagcttgggtgaaactaagtggaggtccgaaccgactgatgttgaagaatcagcggatgagttgtggttaggggtgaaatgccactcgaacccagagctagctggttctccccgaaatgcgttgaggcgcagcagttgactggacatctaggggtaaagcactgtttcggtgcgggccgcgagagcggtaccaaatcgaggcaaactctgaatactagatatgacccaaaaataacaggggtcaaggtcagccagtgagacgatgggggataagcttcatcgtcgagagggaaacagcccggatcaccagctaaggcccctaaatgaccgctcagtgataaaggaggtaggggtgcagagacagccaggaggtttgcctagaagcagccacccttgaaagagtgcgtaatagctcactgatcgagcgctcttgcgccgaagatgaacggggctaagcgatctgccgaagctgtgggatgtcaaaatgcatcggtaggggagcgttccgccttagagggaagcaaccgcgaaagcgggggtcgacgaagcggaagcgagaatgtcggcttgagtaacgcaaacattggtgagaatccaatgccccgaaaacctaagggttcctccgcaaggttcgtccacggagggtgagtcagggcctaagatcaggccgaaaggcgtagtcgatggacaacaggtgaatattcctgtactaccccttgttggtcccgagggacggaggaggctaggttagccgaaagatggttatcggttcaaggacgcaaggtgaccttgctttttcagggtaagaaggggtagagaaaatgcctcgagccgatgtccgagtaccaggcgctacggcgctgaagtaacccatgccatactcccaggaaaagctcgaacgaccttcaacaaaagggtacctgtacccgaaaccgacacaggtgggtaggtagagaatacctaggggcgcgagacaactctctctaaggaactcggcaaaatagccccgtaacttcgggagaaggggtgcctcctcacaaagggggtcgcagtgaccaggcccgggcgactgtttaccaaaaacacaggtctccgcaaagtcgtaagaccatgtatgggggctgacgcctgcccagtgccggaaggtcaaggaagttggtgacctgatgacaggggagccggcgaccgaagccccggtgaacggcggccgtaactataacggtcctaaggtagcgaaattccttgtcgggtaagttccgacccgcacgaaaggcgtaacgatctgggcactgtctcggagagaggctcggtgaaatagacatgtctgtgaagatgcggactacctgcacctggacagaaagaccctatgaagctttactgttccctgggattggctttgggcctttcctgcgcagcttaggtggaaggcgaagaaggcccccttccgggggggcccgagccatcagtgagataccactctggaagagctagaattctaaccttgtgtcaggacctacgggccaagggacagtctcaggtagacagtttctatggggcgtaggcctcccaaaaggtaacggaggcgtgcaaaggtttcctcgggccggacggagattggccctcgagtgcaaaggcagaagggagcttgactgcaagactcacccgtcgagcagggacgaaagtcggccttagtgatccgacggtgccgagtggaagggccgtcgctcaacggataaaagttactctagggataacaggctgatcttccccaagagttcacatcgacgggaaggtttggcacctcgatgtcggctcttcgccacctggggctgtagtatgttccaagggttgggctgttcgcccattaaagcggtacgtgagctgggttcagaacgtcgtgagacagttcggtccatatccggtgcgggcgttagagcattgagaggacctttccctagtacgagaggaccgggaaggacgcacctctggtgtaccagttatcgtgcccacggtaaacgctgggtagccaagtgcggagcggataactgctgaaagcatctaagtagtaagcccaccccaagatgagtgctctcctattccgacttccccagagcctccggtagcacagccgagaagcgacgggttctctgcccctgcggggatggagcgacagaagtattgagaatccaagataaggtcacggcgagacgagccgtttatcattacgataggtgtcaagtggaagtgcagtgatgtatgcagctgaggcatcctaacagaccgagagatttgaaccttgttcctacatgacctgatcaattcgatcaggtactcgccatctattttcattgttcaactgtttgacaacatgaaaaaaccaaaagctctgctctccctctctatctatccaagggatggaagggcagaggcctttggtgtcccttccagtcaagaattggggcctcacaatcactagccaatatgcttttctctcatgcctttcttcgttcatggtttgatattctggtgtcctaggcgtagaggaaccacaccaatccatcccgaacttggtggttaaactctactgcggtgacgatactgtaggggaggtcctgcggaaaaatagctcgacgccagaatgataaaaagcttaacacctcttatttgactttgaaaatatttttcaaaaagataaaaatccaaaatgcaaaggtcgtcttattcaaaacctcaattatgcttctctcccacttcacacctcgaaacgcactgttcttgagaaggcgctttcacatcttcttaacccgaatgaggagaggaaaggttcctttttgagggtactcccgggaacagatccagtggagacggggtgggggatatagctcagttggtagagctccgctcttgcaaggcggatgtcagcggttcgagtccgcttatctccagcccgtgaacttagctgatactatgatagcaccgaatttttccaattcggcagttcgatctatgatttctcattcatggacgttgataagatccttccatttagcagcaccttaggatggcatagccttaacgttaatggcgaggttgggtcgttgcgattacgggttggatgtctaattgtccaggcggtaatgatagtatcttgtacctgaaccggtggctcactttttctaagtaatggggaagaggaccgaaacatgccactgaaagactctactgagacaaagggctgtcaagaacgtagaggaggtaggatgggcagttggtcagatctagtatggatcgtacatggacgatagttggagtcggcggctctcctaggattccctcatctgggatccctggggaagaggatcaagtttgcccttgcgaatagcttgatgcactatctcccttcaaccctttgagcgaaatgtggcaaaaggaaggaaaatccatggaccgaccccatcgtctccaccccgtaggaactacgagatcaccccaaggacgccttcggcatccaggggtcactgaccgaccatagaccctgttcaataagtggaacacattagctgtccgctctccggttgggcagtaagggtcggagaagggcaatcactcgttcttaaaaccagcattcttaagaccaaagagtcgggcggaaaaaaggggagagctctccgttcctggttctcctgtagctggattctccggaaccacaagaatccttagaatgggattccaactcagcaccttttgagattttgagaagagttgctctttggagagcacagtacgatgaaagttgtaagctgtgttcgggggggagttattgtctatcgttggcctctatggtagaatcagtcggggaggcctgagaggcggtggtttaccctgggcggcatggccaagcggtaaggcaggggactgcaaatcctttatccccagttcaaatctgggtgtcgcctatgactcgacaaattcttgccctgcataagcaaaggcaaaggacggggcttgtcgatacttgatttatagaatacatagttctataagttgttttctcaaaatctggctcttttgggttcggcccaaacagatataccaatagggatgaggtaaggcttacttattaattccagaactacgaaagtaagaggtcagaaatcttggtatccaaaggttcctaaaattccatttttgctcctaggattgaagaaaagattatatgaaagactgtcaagtcttcctaattatcttacactacctacactaagggtctactgactctgtctggtggaaaggattgaatttgtatccatacttacgatccgacaaacattcaatcagggtggttggtcggctcttatcttatagaataacatagtaaaaaaaaaaggattcctttgcaattttcgttgtgtatcatatttttacttaatactccccaatcagaaatcctataaagaatctgttacgagtggattcattgaattggttcatcaatagccttaagtcagaatcctattttgactctgcgccattgattctactatgattattgatcaataatggaataattccttcatagaaataggagatataattcaccgggactgacggggctcgaacccgcagcttccgccttgacagggcggtgctctgaccaattgaactacaatcccgcgaggtgtatggcacatattcttaatgtaatcataaattctagtcctagtcgtcgtattgtaagaaagacaggaatgatatactgatatcatatccacatataatatgggctatagtgagagtgacacggattgctagtaaccaataattattttacggcaaaaaattgaccctttctctttatttctacagattaggcatttccgtttatggaagacaaattggttatattcatggagcggtgaattatacccccaggggaagtcgaatccccgctgcctccttgaaagagagatgtcctgaaccactagacgataggggcatatacgcccgaccatcatcatactatatagtatgagcagttttttggaattgtcaatatagtctaatggtatgaaatctattgttcctgaatgaactcctatgcatatacgtatatattattgcagtagactcataataaaaatggctaattcatgaattgaataaaacggtcgggatagctcagttggtagagcagaggactgaaaatcctcgtgtcaccagttcaaatctggttcctgacaagaaaaaatctattggatagatattcaaattaatagagaagaatcgggatacatattcgttaatagtctagagtatgatacatatttattaatctaggtatatagatatatacatccatttttataggtgggtaaaggtaaaaaagagatatatgtatggtaaagaacaaagtgagattacgttccctttcttcatttttgcttacgttactttccggtccatctaagtgatgtgcgcgatacaaagttcatggtgcagaactcttttgattcatcctattttttctactcatacgaaagaaatgaatatgatattttccaaattgaaattggggaatctcaatgaagtcctttttagcttaagctgtccataatacaaatttgagtccagtagttattctgtttcatctagaactctggatataagtaaacatgagtttcttatcatttgcggagacaggatttgaacccgtgacctcaaggttatgagccttgtgagctaccaaactgctctactccgcgctaaaaaactaggaactaatggacgaagaaagattggatacgcccctctaccatatctatacaaatagaatagtccatttatacagaatggtaaagaggcccttctatgatcatagatcatagagatctatacaaatatgaaacgatatttttatccgcggatatggtcgaatggtaaaatttctctttgccaaggagaagatgcgggttcgattcccgctatccgcccataattaagtaatgtactatgataaaaaaattaattgactactatactatactatatagttagtatagtagttctatcttacccctttctttgcctaacatccaaaaacaaaagcgggtatagtttagtggtaaaaccctagccttccaagctaacgatgcgggttcgattcccgctacccgctccatattctttattatacatgcgtcatcaatttggatatgcatcctttttcccgaaaagatatattttctgtataatcttatttgagcaagagttaagaatacgaaagaagaaaaatgaaaggtgcgattcgttctattaacaacttaaatagttaaggggtccatcggtttcaaactccgatcaaaaactttatttcttaaaaaggtttaatccttttcttctcaatagcatatttgaggaaaaatatacgttctcacgatttgtatccaaaggccaattagaaattgcatcaaaaagttggattatggagtcgcgaagcataatttttgaattggattaactattccataagtataggtaaaggatctatggatgaagatacaaaagtatatttccaatcgtaactggatcttccatttttgtgttgtaaaaggaaattgaagccaaatagctaaaaaagatagttttggtttactagaaccatcagcatattgtttcagctcggtggaaaccaaattcttttcctgaggatctaggagtgaaaatagggaacgaagtaactagactagatagatttggtataatctctctcctctagagggatcatctagaaagcggtttagatgcattcatacagaaaagctgacatagatattatggatctctttttttctctggaaatacatggaccttccataaaggagccgaatgaaaccaaaatttcatgttcggttttgaattagagacgttaaaaatgatcaaccaacgtcgactataacgcggatgtagccaagtggatcaaggcagtggattgtgaatccaccatgcgcgggttcaattcccgtcgttcgccatcacattatttcaaattccaaaaattagattttccatattcgggctattagctcagtggtagagcgcgcccctgataagggcgaggtctctggttcaagtccaggatggcccgctgcgccagggaaaagaatagaagaagcatctgactctttcatgcatactccacttggctcggtcgttgtgcctgggctgtgagggctctcagccacatggatagttcaatgtgctcatcagcgcctgacccggagatgtggatcatccaaggcacattagctgggttgcccgggactcgaacccggaactagtcggttaaaagccgagtactctaccgttgagttagcaacccnnnnnnnnnnnnnnnnnnnnnnnnnnnnnnnnnnnnnnnnnnnnnnnnnnnnnnnnnnnnnnnnnnnnnnnnnnnnnnnnnnnnnnnnnnnnnnnnnnnnnnnnnnnnnnnnnnnnnnnnnnnnnnnnnnnnnnnnnnnnnnnnnnnnnnnnnnnnnnnnnnnnnnnnnnnnnnnnnnnnnnnnnnnnnnnnnnnnnnnnnnnnnnnnnnnnnnnnnnnnnnnnnnnnnnnnnnnnnnnnnnnnnnnnnnnnnnnnnnnnnnnnnnnnnnnnnnnnnnnnnnnnnnnnnnnnnnnnnnnnnnnnnnnnnnnnnnnnnnnnnnnnnnnnnnnnnnnnnnnnnnnnnnnnnnnnnnnnnnnnnnnnnnnnnnnnnnnnnnnnnnnnnnnnnnnnnnnnnnnnnnnnnnnnnnnnnnnnnnnnnnnnnnnnnnnnnnnnnnnnnnnnnnnnnnnnnnnnnnnnnnnnnnnnnnnnnnnnnnnnnnnnnnnnnnnnnnnnnnnnnnnnnnnnnnnnnnnnnnnnnnnnnnnnnnnnnnnnnnnnnnnnnnnnnnnnnnnnnnnnnnnnnnnnnnnnnnnnnnnnnnnnnatggagtagataatttttccttgttgcaatagaggaaaaagatccctccccaaaccgtgcttgcatttttcattgcacacgactttccctatgtatacatatcaaatttacatcccgagaagaaagtctacaaagactactcagttgcttcaaccactacatgagcatttcagaatagaaatttatgaatatatttagagatctattctctatataggtctcatgaccaattatgccttgaagaggactcgaacctccacgctctttagcacgagattttgagtctcgcgtgtctaccatttcaccatcaaggcatcttgaaagtgaatcgtattccatgaatatgatatctatctaatgtgatatatggaatatatgacaaaagtggagtgttggagtatttctatcgatcggtcatataggtctgagtcagacatcaaattgcttcgatttgaattatccggaggataccttatatatatcaaaaagatgtacaatcaaacctatttctcgattcaatagaagcccaaagaagtgaatatggtacccaaataacgataggatagatatgtcaaaagcgggtctgattacgcctattcctaatcctaaatagaatgtaaggacgtagggatccatatgtaaacatagtctctatttacatacgcttgaatgaccccttctcataataagaatgtacataaccctattccggtctggtccggtatggaatgaacttataatctgatgatcgagtcgattccatgattataagttcataaccccattttgggcggaacagatctactaattcttttattccagttagtaagagggatcttgaactaagaaatagacctagaagggggatatggcgaaattggtagacgctacggacttaaaatccgtcgactttataaatcgtgagggttcaagtccctctatccccaataaagagccattttactttctaactatttgttttttcattaatgaaaacaattcactatctttctcattcattctactctttcacaaacggatccaacagaaatctttggatcttatacaaatgaagatatataggcaatctctattattaaataattcacaatccatatatttactaggtcaaattttttgttttagtccctaaagtactctactaggatgatgcacaaaaaaaggattggattgagccttggtatggaaacctgctaagtggtaacttccaaattcagagaaaccctggaattaaaaatgggcaatcctgagccaaatcttttgggaaaacaaggatataaaactagaataaaaaaggataggtgcagagactcaatggaagccgttctaacgaatagagttgactacgttgcgtcggtagctggaatccctctatcaaaattagagaaaggccatatatacctgtactatacatactgacatatcaaacgattaatcacgacacgtataatatatttatattatttatgaaatcatgaaattcggaaaaattcagagttattgtgaatccattccaatcgaacaaaaattgactattcagtaataaaatcattcattccagagtttgatagatcttttgaaaaattgattaatcggacgagaataaagagagagtcccattctacatgtcaataccgacaacaatgaaatttatagtaagagggccgccgtggtgaaatcggtagacacgctgctcttaggaagcagtgctaaagcatctcggttcgagtccgagcggcggcatcttctaaaagagatacaatagatcctaaaatgtattcaattcgcgattttcaattctgtaatgggacccctttctccccaagtaggattcgaacctacgaccagtcagttaacagccaaccgctctaccactgagctactgaggaacaacgggattcgacctcatagagttcaactcccgttctcaacccatgaacaatatgagtccgaagcttccttcgtaactcccggaacttcttcgtagtggctctgttccatgcctcatttcatagggaaccccaaagtggctctatttcattatattccatctatatcccaattacattcatttaatatccctttggtcttattgacataagagatgtcatttatagtctatctctttctatatatggaaagttaagaaatcatcatataataatcgagaaattgcaatagaaaagaaaaagggaggtttgtgtagggatgacaggatttgaacccgtgacattttgtacccaaaacaaacgcgctaccaagctgcgctacatcccttttaaaaattgttgtacagtgtcattgtacaaaatacctgtctttttttccacatctttattttctcctctatctatatagaactttcttgtcatttcttgtttttggtttcatataataaattatatacatctgaaacccacctaacatataaaaaagaatgaatatttatccgtaatgctcaggtcatctttttcttttttagtgacaggaaaatctcatctattggttcattgtacatatccttgttaggaaatccgcgtataaaaaaaatgatcttctgggacggaaggattcgaacctccgaataacgggaccaaaacccgctgccttaccgcttggccacgccccattttgatttctattcgatattaatcaacactaatattggtattggttattcgtcaatcccaacccaaatacacaaaaacatatgggtattttgttgctaggattcaagacatgtagatatagaatcaaaaaaattcattgatcattacatagaattcaattaagatattgtatgaaagttgaattccttatattctcattttagaatgataatggggggttatttggaaaagtccgaaccgaaaaaaagaaggatttcttgatctgcctttttcatttttccttataaaaataactccaaattatctaatccacaagaacaaaatgcttgttgggcctgtagctcagaggattagagcacgtggctacgaaccacggtgtcgggggttcgaatccctcctcgcccacagccttcccaaaagggaaggccccttactttacctctgggggtaggaaaatcatgatcgggatagcggacgcaaagctattgaacttgggtcttttgtcgaaatggaatggtcttactttttatttatcgtgaatgattcgatcattacatatagtaaccgaggccggaatcagcatatttgtactccccgtaactcttcctcagccaggcttgggcagaatagcagagcaagcacaagtattattagtagcatagcaaaaatgcgttcctcgtcattaatatgtttgctcgcgtgtggcctatcgggagaatcgatgactgcatcaaagatgcactgctagtacatcatctgagaattcttaattggctatttacaattgacggcgattctcaaatatcgtagaacagaatgtgatacgatgagatagaatgcaatagaaacaaagatagggaacgggttacctactcctaacggtcaaagcgagccctttaattcaattcttcattctttaattaagaattaatcaaatgcgtccattgtctaatggataggacagaggtcttctaaacctttggtataggttcaaatcctattggacgcaatttttttccatctattttgttaaatgtctatactaagaaaccctttttgaatgattcaaatcagaaatttttctcaatgattactctcatgctaagaataagtatgacggaaagagagggattcgaaccctcggtacaaataattcgtacaacggattagcaatccgccgctttagtccactcagccatctctcctaattccaaattgaaaatttgttatgtgatgtaacacgtgaaataaagattgataaaaatccaccttcttcattttttttttcattatcttatttaacaaagcaattgatcaggaattcaatatagataatgactcaaaactaatctcctcaatagaaattctcccggcctgttaagtactggccgggacatttcttctttttctcagtttattacttaatttcttactgttgtcaagtaagcaataaaaaaagacatatgataactaaacaatattaaattacatttaacaatttgaaatatttctattctacaagagacaaaatttgaacagttgagattcaattgaccctaattcataagatctgacactggcagttgaaaagaaggtgaagggggtcatgtatacagaatttataatttttatagtctagcttcaatcaccccttcaggcgggaactattagtttagtaatgacttcccagcaaaagcttaacttttatttaaataagctttacatcgcttagccggcgagactaaatacgtgtcaacgctagaattttcatgattccgctgctatcttgattttgtctcacccttttttgttcgacaaatggtccattcatatacaataatgaatatgtaggagagatggccgagtggttcaaggcgtagcattggaactgctatgtaggcttttgtttaccgagggttcgaatccctctctttccgtttctgttaattcaccaacgttatcgaccacaatgtatcaaatcaaataacaattgaaaccattattccagcaataggacccttatttgatagaaattatctattcctaagcggatggattaaggcccttagatctatttagttcggcgaaaagggttccaaattctatgaacctttcttaattaggagagagagggattcgaaccctcgatagttctttgtttagaactataccggttttcaagaccggagctatcaaccactcggccatctctcccaggggtaatctctattttattcctacgaatagaacatgaccatatgatacactaactatctgtagaaacatcccagatgcaaatccatatttcgatgtatctatgtatactgtatacatagatacatgatctagtatgtctgcttgtaaaataaagactaaactccccccgagttcatgtccgaataaaataaagtggtaataagttctaaagaatcaattgattcatgattaaatccctccatgatggattttattacaatttttactaagtgagggatcaaatgtatagttcatttgttggtagctcggaggattagaaacgcccttttaactcagtggtagagtaacgccatggtaaggcgtaagtcatcggttcaaatccgataaagggcttttttccactaaactcaagttttagccttcgtttttcagccattataacgaattctgattattctgactttaagttaggaagttgaacatttattgattagcaaataattgcacgtattaggagtagtccacctgtagtgacatagtagtcctcctcatgtctcattattcacaaattgtcctgggacataacagaaatattctataatactctatatatacaattcctattattaatcaacaaaccaaggtgttcttatttctccaatgttcttattataaataaataaaaagtaagtggacctgacccattgaatgatgactatatcagctattctgatatttaaattcgatatagattaaattgtacaagcagatttttttatttccttagaccacgcaaagcaagaatttgtcaatatttatgatttaatcttcttgttactggatgctccataggaataaatcactattttttcttctacaaagtttatttccaaatttttcaattccagagaacgaatgcgagaaaaggactttcattttcagtctaccattatttaatattcaattttgtggcaggaaaaagtaggaaattttattttggttaaaagatatactctgaaatatgagtcataggacaattcaggattcaaatggttatcaaatggttatatagtataaggactaatcgaatcgagctcatggatttacctaggttagtttgtggcccaatagaaaaagagtatattcgaaacccattgtaaaggggcattgaacgagaaatcgtctatagataatcgaactatcgtatgccttggaaatgatatgaggtgttcggaaatggttgaagtaattgaataggaggatcactagccggctatcggagtcgaaccgatgaccatcgcattacaaatgcgatgctctaacctctgagctaagcgggctcgcataagaaaaaattgcgtaacaaatagaaatgttgtataggaattccggaaaatgtcggtcttagatatgaactaaactaattaatagagttttatagctaaagttctaagctaagttcttttagaaatgaatgaatatcgaacgttacagtattacaaatcacactgtaaaaatgaaagggagaaataaaatagatatgggatatatctattcatcttgaattgaaaatacatcaatgatagaattatttctgattgaaataaacaaggtttacccaatagaaatgaactgatagaggatggaaaaaaagaaataaatgaaagagatggatgaaattataaatgtatcttgcaaagaaaagggaaaagggatataactcagcggtagagtgtcaccttgacgtggtggaagtcatcagttcgagcctgattatccctaaacccaatgtgagtttttctattttgacttgctcccccgccgtgatcgaacgagaatggataagaggcttgtgggattgacgtgatagggtagggatggctatattgctgggagcgaactccaggctaatatgaagcgcatggatacaaacaagttatgccttggaatgaaagacaattccgatttgtctacgaacaaggaagctataagtaatgcaactatgaatagggctatacggactcgaaccgtagaccttctcggtgtaaacgaggtgctctacctaactgagctatagcccttgtcatagatatcttaacatatagataatttcttgtcaagatggatattccctaatcccacatgataactctttgatccgtttactgttaacaggttggtattgcttagaaataatattctatctataatccccgaggtgatgggtcttcttttgcggtgataaattgtaaaacagatcaaacttattattatcgaaatgattcgaactgtttcaaagacccaacatgcattttgttgcattgggctctttcatcaactgatgtaaagatcagttagtctaccatattttttctttacaggaagataataagctggctccatgtgctctgattcattatttgtattcagatctagtagcaataccaaagtgtttcaaagaagggttaccttgacttaggtctgccttcggcttagatcaacctaagttaaatggagtctctatcgttccgctgcaagagtcgaatatgagacttcatacaccttaaagttcataggatgaaaggaggttttttgaagcccttatactcattatgcctagcattgaatgggctgggtatttaccttatcaactatcaaatcaatgatgggttctatttgatttggcacctaaatttgcaccaaaacggaccgaaccaaatatttgtcaggctattgttctctcgaatctatggagtaagacttcaaccatgttcattgcataataagctcccttgaaaagcattggcgcaccacgctctgtaggatttgaacctacgacatcaggttttggagacccgcgttctaccgaactgaactaagagcgctttcaaagaattttttttcataacacatctcgcataaatatagtatccaaaaaagattatgccccgtcgatctcaattaatctctcgttactgcccataggagaagtaataggtgggccgagctggatttgaaccagcgtagacatattgccaacgaatttacagtccgtccccattaaccgctcgggcatcgacccaggaagaattcattctaggcttatttaatctacgatcaacttcctttcatagtaccccgtggaaacacttgtttattccatatgtatggatgatatgaactgcctaaacaagaacggcgaacaaccagagtactcactacatcaaacaatttccattaatgaaaccatgtaaatccatcggataatcaaagcatgtctgatgaaatggttgttgttatctgcttcaataacgaatcattggtttaactgaataacaaagaaaatagatagacccttctctttctcttcgtctcaggtcgatggatcttctcaatgggaagatctcccatatgcataatacacattccagttgaccgagcctcattctaattgttttgttccgaagcaaagatatccacggaggccggtttgtcctattcagatattcacgaccaggaggtactggattctctttcggataggccctgaaaggagaagaaaggctggaatgccaacagacgtctgtctattctctaattcacccgacccgatagtacccattttggtaacgtccagtgccaaagtcactgaatgggtaagtcgccaatccctaaaacggactactcattcattagatagagaagatcgccaagatttcgtgatccgctgccgaacctattccaattccaacagctcagactcggatcgtggggatcaccggaatacttcgtatcaacagataagatactcgatcaatattgattagatccgaaatccgttattgaattgctcattcaatgagcattctcaatattatctaaatcgtggatacatatgtatccttgacatactgaaacgactgccattattggtatcaaaccaatagcgatttatacaagctaaatcttctaatcggtaattgggccaaagaacaaatttgcatttaattaatttatttgtatctgtattaagatgcttgtcctcatccaaaaattttccagagtttcttatgttgttttcattgcaaaataatggatttctatccgtaacattccaattatgggaattgaaacaaattaacattctcaattctctgcgacgtctaggagatagaatattttcaggaacaaggaaatcataataatttgtgtccccattcacaagcatattcccatatcgtacaatgggtttatccaaattcctcttatcaatatatcttttttttatgcattttctattagtttggtgtttattgttctcgaccaatgaaatacttatagtttgatatataatcaattttccatccctttttatagatagacgaattggttcgataattaatattcctttttttatcaattctgtaagagctagatctttctgaattagcattacatccagatgtatctctcctctttgaatcgaggatatagcaatttcttttggatttatcagtctaagtaagagacaatataccttgatattattgatcattctttggttcaaggagtcatcccatcttaattgaaaaaggaaatattttttcaagaagaaatctagttctgcttccttgtttttcttggattgttttttcttcttaccttttttaatggtaatgtctgatctcgcataattctcttcaatatctttttttttttttcgtagatctgatacaagatttacttggtcctcttgctcatttttttgttggttggaattttctaaaagaagtaatttgattggtataatccatggtttaatcttatatgcatcaaaaagtaagacaaattctgggaagaaccaagattctagatttgatatggtatgataaactctttcttgattcattcccatccaattaaaaaaaatacttttttgattggatgggttgatttcttgatgaatcataagagaaaaaatatccttttttttcaatttgttgttgatttttttttcagtcttagtatttttatcaattttggtaccgatatgtgtattggtccaggtctcaatatcgatattttttctaagacaaaagtggagaattcgacaatcaaaatattttctatctagatttttatgcgtatcaataatatatccttcttctagataatcactaatagctgtacttaccaatacataaaatgattcggattttggtttattgaaattatatggaattttgtgaaccccgtttacttgtaatcttgacccataaatataaaaatcctccttatccccatagttcatatatttatgtgataaaagatcatatctgtagtgttttttccaatgaatccgctttaattgagtccttattttgaatcctataacgttgattgattctatttcgccacttttgcggtactaaccgcgaccatttggtttgagataaattgtattgataatgcccctttaaccagtcaatcattccagacttatgaattttcttatgtcttgaattgtaatcaaatattcctcgtgtcatacaataatccttgattttatccttaataaaaggataagtcccctgatattgaagtagagatttcaattgatacttgttaagtaattgggtttgtgataatttgtaaaatacatatgcttgggacaaggaggataagtcataatacattttattactattactattcgaaaaggacttttttatagtggaaaaaaagttcattgtattttgatctctttcatcaattccttcctgatttgtttgatcgttgtaaacggatttattgattattttttttgttgattcaaagaaaagttggaaatagatcctgtgaatggtaatcatacatagcaagatatctatatatattttttcaatcagagatttcataaaataatgtgatttacgtaggaatatctgccaaatatgtttcttcgatcttttatcatcacaagttagaattatttttttcttgtcttttgtgattcgttctatttgattcctgattgtgattgttctatcagaaagatctttcatctttttttctatgagtgaataatttgtccaattcatggattggatttgaatggttgatttgtgaataatcttattatttattttggaatcttttccattttcagccgtttcatatactttcgccttgctcaattcaaataagaatattgggtttactttttgaatttccttcattcttgttttttcttttgaaacttttagaagtagaaagaaatcctttttcacttttctaacttttttttggagttctttataaatgggttcaaaaaaggaaggtcgttttcggggattcccaaaagggaattccgcttccattccccaaactgttaaaaaacaaaaattttatttgatccctaggatgagatcgtattttagatcttcgccaaggtttcaaacagaaaggaaatagaatctttatctgaataccgtccgttaaccaatctttgggaaattctgtttctgataattgaacaccattataagtgcatttaacatgcatttctctattccactccttcaaatcctcataccattcggggaattggaataataacatacggccaatatttttagcaattatcaatgaaggcaatacaatgtattttctaagaaaagattgggttactaacatcaaacctcttattgcttgagcaaatataatgctatcccaagtttctgatattactatacgttcattttcctcttttttttcttcgttttttttctctttttcccttgtctcttcctcctcaaaattcaaatgtgaaatttctggttctctccccaccgaattcctaaaaatgagattcatcatttcagagatataaaaagaagaaaaaaaaaatgttttgtctattcgatccaaaaaaagtggggaatgcacatttgcttgaaacatttcccaaataaccgttttacgtctttgagcacgcatggatcctttgattatatcccgacgaaaatccgattgttgcgagtaacgtatcaaagccacctcttccgcttgatcactattattagtattatttgtagttgtaatagggttctgatcgttatcagtataaattactacgcgtttggcttttcttgaacgaatttcatgatcttccttagattcttcctcctgttcttccaaatcatcagttaatttgtatgaccatcgaggaacccttttacggatttcttctattccaatagatttatttctaattattgtttgatcgtttggatcagttgtaattacatcgaatacccattttaaagcttttgtttgactttctaaatcaattcttgtttgctctctcaatgtaggctcaaaaggaaattctttgattgaggttaaggaattatcaatataattcagtaatgattccctatcaggcggattcttttgatgttcaaattttctggaataattagaattattaggaagtagcccataaatcttatttatccaattgatttctatggaatcctccgtatctgtagaagtgattaaatcattcatgatcatatgtgaatagaatttttttattgttccacgatacggtccccccaaaaaggggtcatacgttttgggcaagtatttttgttcattttcatcattgcataatctggtccttttttcgagcatatctagagcaagaaaccccttttctttttctagagcttttgttcgacttattaattcattgttcaagttgtactttttttgctcattggtataaacccaataatgatactgatccacatgggataatttttctgtcgtgtacaaagacatttttcgttctatcatttccgaaaaagtcgataaactaggtggatatgtaaaagatattatttgttttccatcacttggacatgtataaaaaaaatattgtgccatttcatttcgtacagcattttcaaatcgattattttttatataccgacatgggcgattccatcgtttataatcgaaATGATTTTGAAATCTTTTCTACTAGGTAATCTATTATCCTTATGCATGAAGATAATAAATTCGGTCGTTGCGGTCGGACTCTATTATGGATTTCTGACCACATTCTCCATAGGACCCTCTTATCTCTTCCTTCTCCGAGCCCGGGTTATGGAAGAAGGAACCGAGAAGGAGGTATCAGCAACAACTGGTTTTATTACGGGACAGCTatcaagaattctcactatttcttagattcatggatcaaattcgattcagtgggatctttcactcacatttttttccaccaagaacgttttatgaaactctttgacccccgaatttggagtatcctactttcacgtgattcacagggttcaacaagcaatcgatatttcacgatcaaaggtgtagtactgcttgtagtagcggtccttatatctcgtattaacaatcgaaagatggtcgaaagaaaaaatctctatttgatggggcttcctcctatacctatgaattccattggacccagaaatgagacattggaagaatctttttggtcttccaatatcaataggttgattgtttcgctcctgtatcttccaaaagggaaaaagatttctgagagttgtttcatggatccgcaagagattacttgggttctcccaataaataaaaagtgtatcatgcggagttcgcgatggtggaggaaccggatcggaaaaaagagggattttagttgtaagatatctaatgaaaccgtagctggaattgagatctcattcaaagagaaagatagcaaatatctggagtttctttttttatcctatatggatgatccgatccgcaaggaccatgattgggaattgtttgatcgtctttctccgaggaagaagcgaaacataatcaacttgaattcgggacagctattcgaaatcttagggaaagacttgatttgttatctcatgtctgcttttcgtgaaaaaagaccaattgaagggaagggtttcttcaaacagcaaggagctgaggcaactattcaatcaaatgatattgagcatgtttcccatctcttctcgagaaacaagtggggtatttctttgcaaaattgtgctcaatttcatatgtggcaattccgccaagatctcttccttagttgggggaagaatcagcacgaattggatttgaacgtatcgagagagaatttgatttggttagacaatgtgtggttgggaaggttttttagcaaggtacggaatgtattgtcaaatattcaatatgattccattagaaatgaggattcagaatatcacacattgatcgatcaaacagagattcagcaactaaaagaaagattgattctttgggatccttcctttcttcaaacggaacgaacagagatagaatcagatcgattcccgaaatgcctttttggatcttcctcaatgtcccggctattcacggaacgtgagaagcagatgaataatcatctgcttccggaagaaatcgaagaatttcttgggaatcctacaagtcgttcttttttctctgacagatggtcagaacttcatctgggttcgaatcctactgagaggtccactagagatcagagattttggaagaaaaaacaagatgtttcttttgtcccttccaggcgatcggaaaatgttgatatattcaagataattacgtatttacaaaaaaccgtctcaattcatcctatttcatcagatccgggatgtgatatggttccgaaggatgaatcggatatggacagttccaataagatttcattcttgaacaagaatccattttttgatttatttcatctattccatgaccggaacaaagggggatacacgttacaccacgattttgaatcagaagagagatttcaagaaatggcagatctattcactctatcaataaccgagccggatctggtgtatcataggggatttgccttttctattgattcttacgggttggatcaaaaaaaattcttgaatgaggtattcaactccagagatgaatcgaaaaagaaatctttattggttctacctcctcttttttatgaagagaatgaatctttttatcgaaggatcagaaaaaaatcggtccggatccactgcgggaatgatttggaagatccaaaactaaaaacagcggtatttgctagcaacaacataatggaggcagtcaatcaatatagattgatccgaaatctgattcaaatccaatatagcacctacgggtacataagaaatgtatcgaatcgattctttttaatgaatagatccgatcgcaacttcgaatatggaattcaaagggatcaaataggaaatgatactctgaatcatataactgtaatgaaatatacgatcaaccaacatttatcgaatttgaaaaagagtcagaagaaatggtttgatcctcttatttctcgaaccgagagatccatgaatcgggatcctgatgcatatagatacaaatggtccaatgggagcaagaatttccaggaacatttggaagatttcgtttctgaacagaagaagcgttttcaagtagtgttcgatcgattccgtattaatcaatattcgattgattggtccgaggctatcgacaaacaagatttgtctaagtcactttgtttctttttgtccaagtcacttctctttttgtccaagtcacttccctttttgtccaagtcacttccccttttctttgtgagtatcgggaatatccccattcataggtccgagatccacatctatgaattgaagggtccgaatgatcaactctgcaatcagttgttagaatcaataggtgttcaaatcgttcatttgaataaattgaaacccttcttattggatgatcatgatacttcccaaagaccgaaattcttgatcaacggaggaacaatattaccatttttgttcaaaaggataccaaagtggatgattgactcattccatactagaaataatcgcaggaaatcctttgataacacggattcctatttctcaatgatatcccatgatcgagacaattggctgaatcccgtgaaaccatttcatagaagttcattgatatcttctttttataaagcaaatcgacttcgattcttgaatgatccacggttctattgtaacaaaagattccctttttatgtggaaaagacccgtatcaataattatgatcttacatatggacaattcctccgcaacaaaatattttctttgtgcgtcggtaaaaaaaaacatatttttttggagagagagactatttcaccaatcgagtcacaggtatctgacatattcatacctaacgattttccacaaagtggtgacgaaacgtataacttgtacaaatctttccattttccaattcgatccgatccattcgttcgtagagctatttactcgatcgcagacatttctgcaacacctctaacagaggaacaaatagtcaatttggaaagaacttattgtcagcctctttcagatatgaatctatctgattcagaagggaagaacttgcatcagtatctcagtttcaattcaaacatgggtttgattcacactccatgttctgagaaatatttaccatccggaaagaggaaaaaacggagtctttgtctaaagaaatgcgttgagaaagggcagatgtatagaacctttcaacgagatagtgctttttcaaatctctcaaaatggaatctgttccaaacatatatgccatggttccttacttcgacagggtgcaaatatctaaatttcacccttttagatactttttcagacccattgccgatactaagtcaaaaatttgtatccatttttcatgatattatgcatggatcagatatatcatggccaattcctcagaggattcttccacaacggactctgataagtgagattttgagtaagtgtttacagaatcttcttctgtccgaagaaatgattcatcgaaataatgagtcaccccttccattgatatggacacatctgagatcaacaaatgctcgggagttcctctattcaacccttttccttcttcttgttgctggatatctcgttcgtatacatcttctcttcgtttcccgagcctctagtgagttacagacagagttagaaaagatcaaatctttgatgattccatcatacatgattgagttgcgaaaacttctggataggtatcctacatctgaactgaattctttctggttaaagaatctctttctagttgctctggaacaattaggagattctctggaagaaatacggggttctgcttctggtggcaacatgctattgggtggtggtcccgcttatggggtcaaatcaatacgttctaagaagaaatatttgaatatcaatctcatcgatctcataagtattataccaaatcccatcaatcgaatcactttttcgagaaatacgagacatctaagtcgtacaagtaaagagatctattcattgataagaaaaagaaaaaacgtgaacggtgattggattgatgataaaatagaatcctgggtcgcgaacagtgattcgattgatgatgaagaaagagaattcttggttcagttctccaccttaacgacagaaaaaaggattgatcaaattctattgagtctgagtcataatgatcatttatcaaagaatgactctggttatcaaatgattgaacaaccgggatcaatttacttacgatacttagttgacattcataaaaagtatctaatgaattatgagttcaatagatcctgtttagcagaaagacggatattccttgctcattatcagacaatcacttattcacaaacctcgtgtggggctaatagttttcatttcccatctcatggaaaacccttttcgctccgcttagccctatccccttctaggggtattttagtgataggttctataggaactggacgatcctatttggtcaaatacctagcgacaaactcctatgttcctttcattacggtatttccgaacaagttcctggatgacaagcctaaaggttatcttattgatgatatcgatgatgatagtgacgatatcgatattgatcttgatacggagctgctaactatgacgaatgtgctaactatgtatatgacgccgaaaatagaccaatttgatatcacccttcaattcgaattagcaaaagcaatgtcttcttgcataatatggattccaaacattcatgatctgtatgtgaatgagtcgaattacttatccctcggtctattagagaactatctctccagggattgtgaaagatgttccactagaaatattcttgttattgcttcgactcatattccccaaaaagtggatcccgctctaatagctccgaataaattaaatacatgcattaagatacgaaggcttcttattccacaacaacgaaagcactttttcattctttcatatactaggggatttcacttggaaaagaaaatgttccatactaacggattcgggtccataaccatgggttccaatgcacgagatcttgtagcacttatcaatgaggccctatcaattagtattacacagaagaaatcaattatagaaactaatacaattagattggctcttcatagacaaacttgggatttgcgatcccaggtaagatcggttcaggatcatgggatccttttctatcagataggaagggctgttgcacaaaatgtacttctaagcaattgccccatggatcctatatctatctatatgaagaagaaatcatgtaaggaagggtattcttatttgtacaaatggtacttcgaacttggaacgagcatgaagaaattaacgatacttctttatcttttgagttgttctgccggatcggtcgctcaagatctttggtcttcacccggacccgatgaaaaaaattggatcacttcttatggattcgttgagaatgattctgatctagttcatggcctattagaagtagaaggcgctccggtgggatcctcacggacagagaaagattgcagtcagtttgataataatcgagtgacattacttcttcggtccgaaccaaggaatcagttagatatgatgcaaaatggatcttgttctatcgttgatcagagatttctatatgaaaaatacgaatcggagtttgaagaaggggaaggagccgtcgacccgcaacagatagaggaggatttattcaatcacatagtttgggctcctagaatatggcgcctttgtggcaatctatttgattgtatcgaaaggcccactgaattgggatttccctattgggccgggtcatttcggggcaagcggatcatttatcataaagaggatgagcttcaagagaatgattcggagttcttgcagagtggaaccatgcagtaccagacacgagatagatcttccaaagaacaaggcttttttcgaataagccaattcatttgggaccctgcggatccattctttttcctattcaaggatcagccctttgtctctgtgttttcacgtcgagaattctttgcagatgaagagatgtcaaaggggcttattacttcccaaaaaaatcctcctacatctatatataaacgctggttcatcaagaatacgcaagaaaagcacttcgaattgttgattcatcgccagagatggcttagaaccaatagttcattatctaatggatctttccgttctaatactctatccgagagttatcagtatttatcaaatctctttctatctaagggaacgctattggatcaaatgacaaagacattgttgagaaagagatggcttttcccggatgaaatgaaacatttgattcatgtaacaggagaaagatttcccattccttagccgtaaagatatgtggccatgaaaaagggaagggattaagtggaacaggattggccgggtggtagagtttattcgaagcgcttcgtgattttcaaccaattatgtgcttcaatataattacctggagtaagcgctatagcttgtttccaatactcagcagcttgatcggaccaagcttccgcaatttcagaatcaccctgtagaatggcctgttctccccggtaatgacagatcacggccatattattaaaagcttgtggtaagaatgggtttcgttctagtgcccggaaataatattccaaagcctttgtatgctctccattgcttgtgtgtataaggcctatgttatagagtatataacttcgatcatagggatcaatttctggtcgcgtagcttcataataattctgtaaagcttccgcataatttccttcggattgagccaacataccatctctgtaataggtaaatgcccttttttctcctgaagttgtcggaattattcgtaataagatattggctacaattgaagaggtcttatcaataaaatttccatttatatgagatctaggcataattagcaatccattctagaattcttttcattacccggggaaaatgatcccacaaacaaaggaattatacagtacgaaataacataaaaaactttattctaaaatagatacgggctttccgcttaaattgtccccttttgtttggaaagatatgagatattggaaattgatttcattcccatttttgtagtataccaatgagcggaactattactatttcatctaagttaaataaccaaggacttttttattacagattctgataactcgagaagttttgatttggttatgatccaaagagaaaaaagaatggaataatcattccatgaaaaaatagagtagagtaaccataccttctgtttgcataagtgtatacaccacgccatacaatcgaaatatcaaaatccatgggacgatcataaatttggaatagatctgtggggtagctgatgaatgagagaagtttttgttgagaaatattaaatgggaaaggaattcttcctatggaactagtgatcggtcgtacctgtactgcagtaatatgaataattcgctattcactcagtttctggtcaataataagttatgtacggaataggtggttccttcccttagaaccgtacttgagagtttcctactcatacggctcaaaaatcgattcttttcttacctatgctaactgaattagatttctcataaacctatcccatttttcttgggttaaccagaagaagttaattacataagttttaaaccctaattttgatcaataatcagaatcagtttgatcttttctcccaccttcagaagaatgaagcataggtatccccacaatatcgttagaattttctgaaaggtaactatctcggtttcatatatggaattcatatagaatctttgaaaaagactttttccataagaaaaaagaacttactatctttgggatctgatgctacaccgctgctcaataccttagtggatcaactctattacataagttgattcctaacttttgcccatatcatgacataagtaagcagttcttaactgtatcgactcaatagctcgctaattgatctttacggtgctttctctatcaatttgatcctttatccatagaatatagtatataggctgcactcattttttttcttcctattttggttctcgtgaagtctctttccttgctacagctgataaaaatcgttgctttggacgatgcatatgtagaaagcctatttttctagtatttactagttgatctttgcttttttccttatttctatagtggagatagtcgcacgttacggtcgttcattctattcaaaaatctccgttccagaaccgtacatgagattttcatctcatacggctcctcccttctgcgcatagtactaagggaataatccatagaataaaaattgaactattctcatctcattatgaactgaaaggaactagtatttttacaagaaatctctagccagccttcccgcaagaggtttttcttaacaccaatcatattagtgttagatataaatggtaactccaacaatttctttgttctcaacgccttctatttccaggaattagtcacttcaacgatctttgatggttatacgggtatccaaagtacaaacgagatggatgtttgttgtcccaaccattcttgttagtcccgataccgataaggaaagggggaatttataacaaagtttttgtgttgttgattcctaggtgtagtgctttttcccttatgccgtctattggtactaatgtagtgtaggattgacccgcaatacagaacccataggtgtaacctttcgctcaatactcaaatcaacaattgaaacatctgaggctgcatcaatcgaggatacacgatagaaggaattgttctatctccaaacttcaccttcaccgagcgtaggtttatttcaagaatttagttctttctataccgaaccgcgtctctttctcgtaagactgaggtgaggaaaaaaacaagaaaaaagaatcaatgaattggcgatcagaacatatatggatagaacttataagggggtctcgaaaaacaagtaatttttgctgggcctgtattctttttttaggttcactaggattcttagcggttggaacttccagttatcttggtaggaatctgatatccatatttccatctcagcaaattattttttttccgcaaggaatcgtgatgtctttttacgggatcgcaggtctattcgttagctcctatttgtggtgcacaattttgtggaatgtaggtagtggttatgaccgattcgatagaaaagaaggaattgtgtgcatttttcgttggggatttcctggaataaatcgtcgcatcttccttcgcttccttatgagagatatccaatcaatcagaattgaggttaaagagggtctttatactcgtcgtgtcctttatatggaaatcagaggtcaaggggccattcccttgactcgtactgatgagaattttactccacgagaaattgaacaaaaagctgccgaattggcctatttcttgggcgtaccaattgaagtattttgaattgaaagaataaattctcggcatgggggaaggaacttgctaattccttttttaatacaattgaattttggaatgttcattcgaacaaaacatgttagattattctatttcctccttcctttgtcgtggcgactcccatagaataaaacaaaaaggagggcaatatggaataactataataaactataattaagaaaagaagaaatttttgtataccaaaagtatttcgtatgcgtatggatcccaactcaattcttttctactagaaaatttctactagtctaataagtagggattcatcaaatatatcgatatttcgtaatacggactcatctttttaggcccaaaagatttttttccttggttgtggctagacggtgaaacatttcgaaataattaactgggggttctaaatccgattcgttattttagtttcgagtattcatagaaaggaacaaatgaagatgaaattgcaatttgcccaattgagatatctaggaataatattgatttttcatttttatccgaaagggcgaactttatatctagatccaagaactaaactcaattaggttcaataccttgttataaactcgtgcttcagagaaatatcatatagagtcaacgaatgagttcattaacgattcaattcacagatggcgtactcctcctgtttgaatcggagtttgaaaccaaactcaggtgagatccaatgtagatctaactttctattcactcgtgggatccgggcggtccgggggggaccaccacggctcctctcttctcgagaatctatacatcccttatcagtgtatggacagctatctttcgagcacaggtttaggttcggcctcaatgggaaaatggagcacctaacaacgcatcttcacagaccaagaactacgagatcgcccctttcattctggggtgacggagggatcgtaccattcgagcctttttttcatgcttttcccggaggtctggagaaagcagcaatcaatagctcacttcttggtcttcgaccccctcagtcactacgagcgcccccccgatcagtgcaatgggatgtgtctatttatctatctcttgactcgaaatgggagcagaaaaaggatcttagagtgtctagggttgggccaggagggtctcttaacgccttcttttttcttcccatcggagttatttcacaaagacttgccacggtaagggagaagggggaacaagcacacttgaagagcgcagtacaacggagagttgtatgctgcgttcgggaaggatgaatcgctcccgaaaaggaatctattgattctctcccaattggttggatcgtaggtgcgatgatttacttcacttctcctcaggaggataggtggggcgat
[truncated: 32,270,642 more chars]
