# Supplementary material for: The different routes of parallel evolution in epiarenic growth in a hyperarid desert environment
Source: Front Plant Sci. 2026 Jul 7;17:1822909. doi: 10.3389/fpls.2026.1822909 (PMC13392990; doi:10.3389/fpls.2026.1822909)
Supplement: Supplementary file 4 [file SupplementaryFile4.docx]

**Supplementary Material 004:**

**This Table summarizes the chosen substitution models in ML analysis to study plastome pyhlogentics. The respective nexus-file is also provided and correspond to the alignment provided with Suupl. Material 003.**

**Evolutionary model: genetic regions**

1) K3Pu+F+R3: ccD_atpB_intergenic_infA_intergenic_ndhI_ndhK_petB_intron_psbJ_intergenic_psbN_intergenic_rpl14_rpl20_intergenic_rpl32_rpl33_rpl36_rpoA_rpoC2_rpoC2_intergenic_rps11_rps16_rps7_intergenic_rps8_trnfM_CAU_intergenic_trnV_UAC_intron_ycf3_intron1_ycf3_intron2

2) K3Pu+F+R3: accD_intergenic_atpF_intergenic_ndhI_intergenic_petN_intergenic_rpl32_intergenic_rpoB_intergenic_rps16_intergenic_rps2_intergenic_trnG_UCC_intron_trnK_UUU_intron_trnL_UAA_intron_trnS_UGA_intergenic

3) TVM+F+I+R3: atpA_atpE_petB_petD_petG_petN_psaJ_psbB_psbC_psbD_psbH_psbI_rpl20_trnR_UCU_trnS_GGA_trnT_GGU

4) TVM+F+I+R3: atpA_intergenic_ndhF_intergenic_ndhG_intergenic_psaC_intergenic_psaI_intergenic_psaJ_intergenic_rpl33_intergenic_trnF_GAA_intergenic_trnK_UUU_intergenic_trnL_UAA_intergenic_trnS_GCU_intergenic_trnT_GGU_intergenic_trnT_UGU_intergenic_trnW_CCA_intergenic_trnY_GUA_intergenic_ycf4_intergenic

5) GTR+F+I+R2: atpB_atpI_infA_ndhH_ndhJ_petA_psbL_rpoB_rpoC1_rps18_rps2_rps4

6) K3Pu+F+R3: atpF_atpF_intron_ccsA_cemA_clpP_intron1_clpP_intron2_petB_intergenic_petD_intron_psbB_intergenic_psbH_intergenic_psbM_psbT_rps12_intergenic1_rps19_intergenic_rps3_ycf4

7) TN+F+I+R2: atpH_psaA_psaB_psbA_psbE_psbJ_psbN_trnD_GUC_trnE_UUC_trnK_UUU_trnL_UAG_trnW_CCA

8) TVM+F+R4: atpH_intergenic_cemA_intergenic_clpP_intergenic_matK_intergenic_ndhA_intron_ndhC_intergenic_ndhD_intergenic_ndhE_intergenic_petL_intergenic_psbA_intergenic_psbE_intergenic_psbM_intergenic_psbZ_intergenic_rbcL_intergenic_rpl14_intergenic_rpl22_rpoA_intergenic_rps14_intergenic_rps15_rps16_intron_rps18_intergenic_rps4_intergenic_trnC_GCA_intergenic_trnD_GUC_intergenic_trnE_UUC_intergenic_trnG_UCC_intergenic_trnL_UAG_intergenic_trnQ_UUG_intergenic_trnS_GGA_intergenic_trnV_UAC_intergenic_ycf1_ycf3_intergenic

9) TIM+F+R3: atpI_intergenic_petG_intergenic_psbL_intergenic_rps3_intergenic_trnG_GCC_intergenic_trnH_GUG_intergenic_trnR_UCU_intergenic

10) TIM+F+I+R3: ccsA_intergenic_petA_intergenic_psaA_intergenic_psbK_intergenic_rpl22_intergenic_rpl2_intergenic_rpoC1_intergenic_rps15_intergenic

11) K3Pu+F+I+R3: clpP_psaC_psbF_psbK_rbcL_rrn5S_intergenic_trnR_ACG_intergenic_ycf1_pseudo

12) TVM+F+I+R3: matK_ndhA_ndhC_ndhD_ndhE_ndhF_ndhG_rpoC1_intron

13) K3Pu+F+R2: ndhB_ndhB_intergenic_petL_psaI_psbZ_rps12_intergenic2_rps14_rps19_trnA_UGC_intergenic_trnL_CAA_intergenic_trnN_GUU_intergenic_trnV_GAC_intergenic_ycf15_ycf15_intergenic_ycf2_ycf3

14) HKY+F: ndhB_intron_psaB_intergenic_rpl23_rpl23_intergenic_rpl2_rpl2_intron_rps12_rps12_intron_rps7_rrn4_5S_intergenic_trnF_GAA_trnI_GAU_intergenic_trnP_UGG_trnS_GCU_ycf68_ycf68_intergeni,

15) TVM+F+I+R3: ndhH_intergenic_petD_intergenic_psbI_intergenic_rpl16_intergenic_rpl16_intron_rpl36_intergenic_rps11_intergenic_rps8_intergenic

16) TVM+F+I+R3: ndhJ_intergenic_psbT_intergenic_trnC_GCA_trnP_UGG_intergenic

17) GTR+F+I+R5: psbC_intergenic_rrn16S_intergenic_rrn23S_intergenic_ycf2_intergenic,

18) K3P+I+R2: rpl16_ycf68_intron

19) HKY+F+R2: rrn16S_rrn23S_rrn4_5S_rrn5S_trnA_UGC_trnA_UGC_intron_trnfM_CAU_trnG_GCC_trnG_UCC_trnH_GUG_trnI_GAU_trnI_GAU_intron_trnL_CAA_trnL_UAA_trnN_GUU_trnQ_UUG_trnR_ACG_trnS_UGA_trnT_UGU_trnV_GAC_trnV_UAC_trnY_GUA

**NOTE:**

In the following you will find the respective NEXUS-file:

# partitioning file for plastome ML analysis

#nexus

begin sets;

charset accD_atpB_intergenic_infA_intergenic_ndhI_ndhK_petB_intron_psbJ_intergenic_psbN_intergenic_rpl14_rpl20_intergenic_rpl32_rpl33_rpl36_rpoA_rpoC2_rpoC2_intergenic_rps11_rps16_rps7_intergenic_rps8_trnfM_CAU_intergenic_trnV_UAC_intron_ycf3_intron1_ycf3_intron2 = 1-1260 4451-4934 13361-13472 27488-28030 28674-29359 31929-32650 49310-49413 51247-51351 53930-54298 56202-56864 59197-59357 59884-60084 60291-60404 60504-61517 68361-72430 72431-72544 72545-72961 76719-76966 82010-82067 82068-82472 90863-91019 100180-100742 113961-114673 114674-115390;

charset accD_intergenic_atpF_intergenic_ndhI_intergenic_petN_intergenic_rpl32_intergenic_rpoB_intergenic_rps16_intergenic_rps2_intergenic_trnG_UCC_intron_trnK_UUU_intron_trnL_UAA_intron_trnS_UGA_intergenic = 1261-1364 5895-6269 28031-28118 34576-35551 59358-59883 64797-65653 76967-77322 79774-79995 91398-92056 93064-93296 94248-94747 97929-98252;

charset atpA_atpE_petB_petD_petG_petN_psaJ_psbB_psbC_psbD_psbH_psbI_rpl20_trnR_UCU_trnS_GGA_trnT_GGU = 1365-2888 4935-5339 31112-31759 32651-33154 33988-34101 34486-34575 41316-41444 43069-44595 44759-46180 46316-47377 48653-48874 48999-49109 55848-56201 96652-96723 97565-97651 98253-98324;

charset atpA_intergenic_ndhF_intergenic_ndhG_intergenic_psaC_intergenic_psaI_intergenic_psaJ_intergenic_rpl33_intergenic_trnF_GAA_intergenic_trnK_UUU_intergenic_trnL_UAA_intergenic_trnS_GCU_intergenic_trnT_GGU_intergenic_trnT_UGU_intergenic_trnW_CCA_intergenic_trnY_GUA_intergenic_ycf4_intergenic = 2889-2959 25297-25380 25912-26231 40751-40949 41061-41315 41445-41811 60085-60290 90341-90788 92479-93063 94011-94247 96920-97564 98325-99198 99272-99641 100817-100929 101014-101070 115946-116601;

charset atpB_atpI_infA_ndhH_ndhJ_petA_psbL_rpoB_rpoC1_rps18_rps2_rps4 = 2960-4450 7904-8647 13127-13360 26232-27413 28119-28598 29360-30322 49941-50057 61578-64796 65654-67681 78106-78411 79063-79773 80689-81294;

charset atpF_atpF_intron_ccsA_cemA_clpP_intron1_clpP_intron2_petB_intergenic_petD_intron_psbB_intergenic_psbH_intergenic_psbM_psbT_rps12_intergenic1_rps19_intergenic_rps3_ycf4 = 5340-5894 6270-6980 8843-9769 10020-10709 11901-12518 12519-13126 31760-31928 33295-33987 44596-44758 48875-48998 50080-50184 51352-51453 73457-73590 78940-79062 79996-80646 115391-115945;

charset atpH_psaA_psaB_psbA_psbE_psbJ_psbN_trnD_GUC_trnE_UUC_trnK_UUU_trnL_UAG_trnW_CCA = 6981-7226 35552-37804 38275-40479 41812-42873 47378-47629 49187-49309 51115-51246 89700-89773 90025-90096 92407-92478 94748-94827 100743-100816;

charset atpH_intergenic_cemA_intergenic_clpP_intergenic_matK_intergenic_ndhA_intron_ndhC_intergenic_ndhD_intergenic_ndhE_intergenic_petL_intergenic_psbA_intergenic_psbE_intergenic_psbM_intergenic_psbZ_intergenic_rbcL_intergenic_rpl14_intergenic_rpl22_rpoA_intergenic_rps14_intergenic_rps15_rps16_intron_rps18_intergenic_rps4_intergenic_trnC_GCA_intergenic_trnD_GUC_intergenic_trnE_UUC_intergenic_trnG_UCC_intergenic_trnL_UAG_intergenic_trnQ_UUG_intergenic_trnS_GGA_intergenic_trnV_UAC_intergenic_ycf1_ycf3_intergenic = 7227-7903 10710-10922 11508-11900 15009-15647 16737-17755 20600-21007 22529-22621 22928-23106 34314-34485 42874-43068 47630-48532 50185-51114 51703-51939 53380-53929 54299-54416 56865-57248 61518-61577 76023-76125 76126-76398 77323-78105 78412-78660 81295-81541 89057-89699 89774-90024 90097-90267 91276-91397 94828-94901 95714-96045 97652-97835 100013-100179 101809-105959 113371-113960;

charset atpI_intergenic_petG_intergenic_psbL_intergenic_rps3_intergenic_trnG_GCC_intergenic_trnH_GUG_intergenic_trnR_UCU_intergenic = 8648-8842 34102-34217 50058-50079 80647-80688 91091-91204 92131-92172 96724-96831;

charset ccsA_intergenic_petA_intergenic_psaA_intergenic_psbK_intergenic_rpl22_intergenic_rpl2_intergenic_rpoC1_intergenic_rps15_intergenic = 9770-10019 30323-31111 37805-38274 49600-49940 57249-57273 58525-58530 67682-67707 76399-76718;

charset clpP_psaC_psbF_psbK_rbcL_rrn5S_intergenic_trnR_ACG_intergenic_ycf1_pseudo = 10923-11507 40505-40750 48533-48652 49414-49599 51940-53379 87741-87972 96120-96651 105960-106165;

charset matK_ndhA_ndhC_ndhD_ndhE_ndhF_ndhG_rpoC1_intron = 13473-15008 15648-16736 20237-20599 21008-22528 22622-22927 23107-25296 25381-25911 67708-68360;

charset ndhB_ndhB_intergenic_petL_psaI_psbZ_rps12_intergenic2_rps14_rps19_trnA_UGC_intergenic_trnL_CAA_intergenic_trnN_GUU_intergenic_trnV_GAC_intergenic_ycf15_ycf15_intergenic_ycf2_ycf3 = 17756-19288 19289-19547 34218-34313 40950-41060 51514-51702 73591-75184 75720-76022 78661-78939 88046-88190 93378-93925 94974-95287 99714-99938 101071-101240 101241-101808 106166-112795 112864-113370;

charset ndhB_intron_psaB_intergenic_rpl23_rpl23_intergenic_rpl2_rpl2_intron_rps12_rps12_intron_rps7_rrn4_5S_intergenic_trnF_GAA_trnI_GAU_intergenic_trnP_UGG_trnS_GCU_ycf68_ycf68_intergenic = 19548-20236 40480-40504 57274-57543 57544-57708 57709-58524 58531-59196 73085-73456 75185-75719 81542-82009 87396-87619 90268-90340 92244-92307 95288-95361 96832-96919 116602-116948 116949-117296;

charset ndhH_intergenic_petD_intergenic_psbI_intergenic_rpl16_intergenic_rpl16_intron_rpl36_intergenic_rps11_intergenic_rps8_intergenic = 27414-27487 33155-33294 49110-49186 54825-54946 54947-55847 60405-60503 72962-73084 82473-82588;

charset ndhJ_intergenic_psbT_intergenic_trnC_GCA_trnP_UGG_intergenic = 28599-28673 51454-51513 88986-89056 95362-95641;

charset psbC_intergenic_rrn16S_intergenic_rrn23S_intergenic_ycf2_intergenic = 46181-46315 84080-84385 87196-87292 112796-112863;

charset rpl16_ycf68_intron = 54417-54824 117297-117324;

charset rrn16S_rrn23S_rrn4_5S_rrn5S_trnA_UGC_trnA_UGC_intron_trnfM_CAU_trnG_GCC_trnG_UCC_trnH_GUG_trnI_GAU_trnI_GAU_intron_trnL_CAA_trnL_UAA_trnN_GUU_trnQ_UUG_trnR_ACG_trnS_UGA_trnT_UGU_trnV_GAC_trnV_UAC_trnY_GUA = 82589-84079 84386-87195 87293-87395 87620-87740 87973-88045 88191-88985 90789-90862 91020-91090 91205-91275 92057-92130 92173-92243 92308-92406 93297-93377 93926-94010 94902-94973 95642-95713 96046-96119 97836-97928 99199-99271 99642-99713 99939-100012 100930-101013;

charpartition mymodels =

K3Pu+F+R3: accD_atpB_intergenic_infA_intergenic_ndhI_ndhK_petB_intron_psbJ_intergenic_psbN_intergenic_rpl14_rpl20_intergenic_rpl32_rpl33_rpl36_rpoA_rpoC2_rpoC2_intergenic_rps11_rps16_rps7_intergenic_rps8_trnfM_CAU_intergenic_trnV_UAC_intron_ycf3_intron1_ycf3_intron2,

K3Pu+F+R3: accD_intergenic_atpF_intergenic_ndhI_intergenic_petN_intergenic_rpl32_intergenic_rpoB_intergenic_rps16_intergenic_rps2_intergenic_trnG_UCC_intron_trnK_UUU_intron_trnL_UAA_intron_trnS_UGA_intergenic,

TVM+F+I+R3: atpA_atpE_petB_petD_petG_petN_psaJ_psbB_psbC_psbD_psbH_psbI_rpl20_trnR_UCU_trnS_GGA_trnT_GGU,

TVM+F+I+R3: atpA_intergenic_ndhF_intergenic_ndhG_intergenic_psaC_intergenic_psaI_intergenic_psaJ_intergenic_rpl33_intergenic_trnF_GAA_intergenic_trnK_UUU_intergenic_trnL_UAA_intergenic_trnS_GCU_intergenic_trnT_GGU_intergenic_trnT_UGU_intergenic_trnW_CCA_intergenic_trnY_GUA_intergenic_ycf4_intergenic,

GTR+F+I+R2: atpB_atpI_infA_ndhH_ndhJ_petA_psbL_rpoB_rpoC1_rps18_rps2_rps4,

K3Pu+F+R3: atpF_atpF_intron_ccsA_cemA_clpP_intron1_clpP_intron2_petB_intergenic_petD_intron_psbB_intergenic_psbH_intergenic_psbM_psbT_rps12_intergenic1_rps19_intergenic_rps3_ycf4,

TN+F+I+R2: atpH_psaA_psaB_psbA_psbE_psbJ_psbN_trnD_GUC_trnE_UUC_trnK_UUU_trnL_UAG_trnW_CCA,

TVM+F+R4: atpH_intergenic_cemA_intergenic_clpP_intergenic_matK_intergenic_ndhA_intron_ndhC_intergenic_ndhD_intergenic_ndhE_intergenic_petL_intergenic_psbA_intergenic_psbE_intergenic_psbM_intergenic_psbZ_intergenic_rbcL_intergenic_rpl14_intergenic_rpl22_rpoA_intergenic_rps14_intergenic_rps15_rps16_intron_rps18_intergenic_rps4_intergenic_trnC_GCA_intergenic_trnD_GUC_intergenic_trnE_UUC_intergenic_trnG_UCC_intergenic_trnL_UAG_intergenic_trnQ_UUG_intergenic_trnS_GGA_intergenic_trnV_UAC_intergenic_ycf1_ycf3_intergenic,

TIM+F+R3: atpI_intergenic_petG_intergenic_psbL_intergenic_rps3_intergenic_trnG_GCC_intergenic_trnH_GUG_intergenic_trnR_UCU_intergenic,

TIM+F+I+R3: ccsA_intergenic_petA_intergenic_psaA_intergenic_psbK_intergenic_rpl22_intergenic_rpl2_intergenic_rpoC1_intergenic_rps15_intergenic,

K3Pu+F+I+R3: clpP_psaC_psbF_psbK_rbcL_rrn5S_intergenic_trnR_ACG_intergenic_ycf1_pseudo,

TVM+F+I+R3: matK_ndhA_ndhC_ndhD_ndhE_ndhF_ndhG_rpoC1_intron,

K3Pu+F+R2: ndhB_ndhB_intergenic_petL_psaI_psbZ_rps12_intergenic2_rps14_rps19_trnA_UGC_intergenic_trnL_CAA_intergenic_trnN_GUU_intergenic_trnV_GAC_intergenic_ycf15_ycf15_intergenic_ycf2_ycf3,

HKY+F: ndhB_intron_psaB_intergenic_rpl23_rpl23_intergenic_rpl2_rpl2_intron_rps12_rps12_intron_rps7_rrn4_5S_intergenic_trnF_GAA_trnI_GAU_intergenic_trnP_UGG_trnS_GCU_ycf68_ycf68_intergenic,

TVM+F+I+R3: ndhH_intergenic_petD_intergenic_psbI_intergenic_rpl16_intergenic_rpl16_intron_rpl36_intergenic_rps11_intergenic_rps8_intergenic,

TVM+F+I+R3: ndhJ_intergenic_psbT_intergenic_trnC_GCA_trnP_UGG_intergenic,

GTR+F+I+R5: psbC_intergenic_rrn16S_intergenic_rrn23S_intergenic_ycf2_intergenic,

K3P+I+R2: rpl16_ycf68_intron,

HKY+F+R2: rrn16S_rrn23S_rrn4_5S_rrn5S_trnA_UGC_trnA_UGC_intron_trnfM_CAU_trnG_GCC_trnG_UCC_trnH_GUG_trnI_GAU_trnI_GAU_intron_trnL_CAA_trnL_UAA_trnN_GUU_trnQ_UUG_trnR_ACG_trnS_UGA_trnT_UGU_trnV_GAC_trnV_UAC_trnY_GUA;

end;
