# Supplementary material for: The different routes of parallel evolution in epiarenic growth in a hyperarid desert environment
Source: Front Plant Sci. 2026 Jul 7;17:1822909. doi: 10.3389/fpls.2026.1822909 (PMC13392990; doi:10.3389/fpls.2026.1822909)
Supplement: Supplementary file 5 [file SupplementaryFile5.docx]

**Supplementary Material 005:**

**FASTA file of the *Agt1* alignment used for the reconstruction of an allelic phylogenetic tree. Alignment length including gaps is 3821 base pairs.**

>HEID811243_MT047538_Tillandsia_virescens_forma_La_Isla_Clone_1

TGCAAGATCATTTTACGCCAA---------------TCATACAATTATAAATGGCAACTAATTACAT-------------------------------------------------------------------------------------------------TCATCTTA----CATATTCCTATATAT--------GAATAGATAATGCAATAC---TGT-----------------------------------------------------------------------------------------------GGATAGA-------TTTTTGCATACTTACCTCGTGGCCTTTCCTAAACGACGATGCCTTTCGATGACATTTTCGAGGCCCTCCTCAAAGATGAGATCCAAAGCTGCTCTTAGCCCGTAAAGTAGTTGGATGGAAGGCGTATACGGCCAGTAAGTTCCAAGCTTGTAGAACTTCAAGTAGTCGTTCCAGTCGAAGAAAACTCTGACCGACTTGGCATTTTTGGAAGCTTCCAGAGCTTTCGGGCTCGCGCAAACAATGCCCATTCCAGTAGGCAGGGAAA-GTGCTTTCTGCGA

>HEID811243_MT047540_Tillandsia_virescens_forma_La_Isla_Clone_3

TGCAAGATCATTTTACGCCAATTATGTTACAAGAGGTCATACAAATATAAATGGCAACTAATTACAT-------------------------------------------------------------------------------------------------TCATCTTA----CATATTCCTATATAT--------GAATAGATAATGCAATAC---TGT-----------------------------------------------------------------------------------------------GGATAGA-------TTTTTGCATACTTACCTCGTGGCCTTTCCTAAACGACGATGTCTTTCGATGACATTTTCGAGGCCCTCCTCAAAGATGAGATCCAAAGCTGCTCTTAGCCCGTAAAGTAGTTGGATGGAAGGCGTATACGGCCAGTAAGTTCCAAGCTTGTAGAACTTCAAGTAGTCGTTCCAGTCGAAGAAAACTCTGACCGACTTGGCATTTTTGGAAGCTTCCAGAGCTTTCGGGCTCGCGCAAACAATGCCCATTCCAGTAGGCAGGGAAA-GTGCTTTCTGCGA

>HEID811243_MT047539_Tillandsia_virescens_forma_La_Isla_Clone_2

TGCAAGATCATTTTACGCCAATTATGTTACAAGAGGTCATACAAATATAAATGGCAACTAATTACAT-------------------------------------------------------------------------------------------------TCATCTTA----CATATTCCTATATAT--------GAATAGATAATGCAATAC---TGT-----------------------------------------------------------------------------------------------GGATAGA-------TTTTTGCATACTTACCTCGTGGCCTTTCCTAAACGACGATGTCTTTCGATGACATTTTCGAGGCCCTCCTCAAAGATGAGATCCAAAGCTGCTCTTAGCCCGTAAAGTAGTTGGATGGAAGGCGTATACGGCCAGTAAGTTCCAAGCTTGTAGAACTTCAAGTAGTCGTTCCAGTCGAAGAAAACTCTGACCGACTTGGCATTTTTGGAAGCTTCCAGAGCTTTCGGGCTCGCGCAAACAATGCCCATTCCAGTAGGCAGGGAAA-GTGCTTTCTGCGA

>HEID132303_MT038826_Tillandsia_rectangula_BRC241

nnnnnnnnnnnnnnACGCCAATTATGTTACAAGAGGTCATACAATTATAAATGGCAATTAATTACAT-------------------------------------------------------------------------------------------------CCACCTTA----TATATATAT--------------GAATAGATAATGCAATAC---TGTAAATTT-----AGAAAAAA------------GCTAAAAGAAA-ACAATCGGCTATTCAKTAAAAGCCAGCTTAATTTTTACAGTACACTCGAT--CGATAGA-------TTTTTGCATACTTACCTCGTGGCCTTTCCTAAACGACGATGCCTTTCGATGACATTTTCGAGGCCCTCCTCAAAGATGAGATCCAAAGCTGCTCTTAGCCCGTAAAGTAGTTGGATGGAAGGCGTATACGGCCAGTAAGTTCCAAGCTTGTAGAACTTCAAGTAGTCATTCCAGTCGAAGAAAACTCTAACCGACTTGGCATTTTTGGAAGCTTCCAGAGCTTTCGGGCTCGCGCAAACAATGCCCATTCCAGTAGGCAGGGAAA-GTGCTTTCTGCGA

>HEID132290_MT047462_1_Tillandsia_xiphioides_BRC456

TGCAAAATCATTTTACGCCAA---------------TCATACAATTATAAATGGCAACTAATTACAT-------------------------------------------------------------------------------------------------CCATCTTA----CATATTCCTATATAT--------GAATAGATAATGCAATAC---TGTAAATTT-----AGAAAACA------------GCTAAAAGAAACACAATCGGCTATTCAGTAAAAGCCAGCTTAATTTTTACAGTATACTCGAT--CGATAGA------TTTTTTGCATACTTACCTCGTGGCCTTTCCTAAACGACGATGCCTTTCGATGACATTTTCGAGCCCCTCCTCAAAGATGAGATCCAAAGCTGCTCTTAGCCCGTAAAGTAGTTGGATGGAAGGCGTATACGGCCAGTAAGTTCCAAGCTTGTAGAACTTCAAGTAGTCATTCCAGTCGAAGAAAACTCTAACCGACTTGGCATTTTTGGAAGCTTCCAGAGCTTTCGGGCTCGCGCAAACAATGCCCATTCCAGTAGGCAGGGAAA-GTGCTTTCTGCGA

>HEID108216_MT047579_1_Tillandsia_geissei

TGCAAGATCATTTTACGCCAA---------------TCATACAATTATAAATGGCAACTAATCATAT-------------------------------------------------------------------------------------------------CCATCTTA----CATATTCCTATATAT--------GAATAGATAATGCAATAC---TGTAAATTT-----AGAAAAAA------------GCTAAAAGAAA-ACAATCGGCTATTCAGTAAAAGCCAGC----TTTTTACAGTA--CTCGAT--CGATAGAAG-------TTTGCATACTTACCTCGTGGCCTTTCCTAAACGACGATGCCTTTCGATGACATTTTCGAGCCCCTCCTCAAAGATGAGATCCAAAGCTGCTCTTAGCCCGTAAAGTAGTTGGATGGAAGGCGTATACGGCCAGTAAGTTCCAAGCTTGTAGAACTTCAAGTAGTCATTCCAGTCGAAGAAAACTCTGACCGACTTGGCATTTTTGGAAGCTTCTAGAGCTTTCGGGCTCGCGCAAACAATGCCCATTCCAGTAGGCAGGGAAA-GTGCTTTCTGCGA

>HEID104565_MT047578_1_Tillandsia_werdermannii

TGCAAGATCATTTTACGCCAA---------------TCATACAATTATAAATGGCAACTAATTACAT-------------------------------------------------------------------------------------------------CCATCTTA----CATATTCCTATATAT--------GAATAGATAATGCAATAC---TGTAAATTT-----AGAAAACA------------GCTAAAAGAAA-ACAATCGGCTATTCAGTAAAAGCCAGC----TTTTTACAGTATACTCGAT--CGATAGAAG-------TTTGCATACTTACCTCGTGGCCTTTCCTAAACGACGATGCCTTTCGATGACATTTTCGAGGCCCTCCTCAAAGATGAGATCCAAAGCTGCTCTTAGCCCGTAAAGTAGTTGGATGGAAGGCGTATACGGCCAGTAAGTTCCAAGCTTGTAGAACTTCAAGTAGTCATTCCAGTCGAAGAAAACTCTGACCGACTTGGCATTTTTGGAAGCTTGCAGAGCTTTCGGGCTAGCGCAAACAATGCCCATTCCAGTAGGCAGGGAAA-GTGCTTTCTGCGA

>HEID131338_MT047555_Tillandsia_purpurea_Clone_1

TGCAAGATCATTTTACGCCAA---------------TCATACAATTATAAATGCCAACTAATTATAT-------------------------------------------------------------------------------------------------CCATCTTA----CATATTCCTATATATAT------GAATAGATAATGCAATAC---TGTAAATTT-----AGGAGAAA------------GCTAAAAGAAA-ACAATCGGCTATTCAGTAAAAGCCAGC----TTTTTACAGTA--CTCGAT--AGATAGAAG-------TTTGCATACTTACCTCGTGGCTTTTCCTAAACGATGATGCCTTTCAATGACATTTTCGAGCCCCTCCTCAAAGATGAGATCCAAAGCTGCTCTTAGCCCGTAAAGTAGTTGGATGGAAGGCGTATACGGCCAGTAAGTTCCGAGCTTGTAGAACTTCAAGTAGTCATTCCAGTCGAAGAAAACTCTGACCGACTTGGCATTTTTTGAAGCTTCTAGAGCTTTCGGGCTCGCGCAAACAATGCCCATTCCAGTAGGCAGGGAAA-GTGCTTTCTGCGA

>HEID131338_MT047554_Tillandsia_purpurea_Clone_2

TGCAAGATCATTTTACGCCAA---------------TCATACAATTATAAATGCCAACTAATTATAT-------------------------------------------------------------------------------------------------CCATCTTA----CATATTCCTATATATAT------GAATAGATAATGCAATAC---TGTAAATTT-----AGGAGAAA------------GCTAAAAGAAA-ACAATCGGCTATTCAGTAAAAGCCAGC----TTTTTACAGTA--CTCGAT--AGATAGAAG-------TTTGCATACTTACCTCGTGGCTTTTCCTAAACGATGATGCCTTTCAATGACATTTTCGAGCCCCTCCTCAAAGATGAGATCCAAAGCTGCTCTTAGCCCGTAAAGTAGTTGGATGGAAGGCGTATACGGCCAGTAAGTTCCAAGCTTGTAGAACTTCAAGTAGTCATTCCAGTCGAAGAAAACTCTGACCGACTTGGCATTTTTTGAAGCTTCTAGAGCTTTCGGGCTCGCGCAAACAATGCCCATTCCAGTAGGCAGGGAAA-GTGCTTTCTGCGA

>HEID131338_MT047553_Tillandsia_purpurea_Clone_3

TGCAAGATCATTTTACGCCAA---------------TCATACAATTATAAATGCCAACTAATTATAT-------------------------------------------------------------------------------------------------CCATCTTA----CATATTCCTATATATAT------GAATAGATAATGCAATAC---TGTAAATTT-----AGGAGAAA------------GCTAAAAGAAA-ACAATCGGCTATTCAGTAAAAGCCAGC----TTTTTACAGTA--CTCGAT--AGATAGAAG-------TTTGCATACTTACCTCGTGGCTTTTCCTAAACGATGATGCCTTTCAATGACATTTTCGAGCCCCTCCTCAAAGATGAGATCCAAAGCTGCTCTTAGCCCGTAAAGTAGTTGGATGGAAGGCGTATACGGCCAGTAAGTTCCAAGCTTGTAGAACTTCAAGTAGTCATTCCAGTCGAAGAAAACTCTGACCGACTTGGCATTTTTTGAAGCTTCTAGAGCTTTCGGGCTCGCGCAAACAATGCCCATTCCAGTAGGCAGGGAAA-GTGCTTTCTGCGA

>HEID104854_MT047551_Tillandsia_purpurea_Clone_3

TGCAAGATCATTTTACGCCAA---------------TCATACAATTATAAATGCCAACTAATTATAT-------------------------------------------------------------------------------------------------CCATCTTA----CATATTCCTATATATAT------GAATAGATAATGCAATAC---TGTAAATTT-----AGGAAAAA------------GCTAAAAGAAA-ACAATCGGCTATTCAGTAAAAGCCAGC----GTTTTACAGTA--CTCGAT--CGATAGAAG-------TTTGCATACTTACCTCGTGGCCTTTCCTAAACGATGATGCCTTTCGATGACATTTTCGAGCCCCTCCTCAAAGATGAGATCCAAAGCTGCTCTTAGCCCGTAAAGTAGTTGGATGGAAGGCGTATACGGCCAGTAAGTTCCGAGCTTGTAGAACTTCAAGTAGTCATTCCAGTCGAAGAAAACTCTGACCGACTTGGCATTTTTGGAAGCTTCTAGAGCTTTCGGGCTCGCGCAAACAATGCCCATTCCAGTAGGCAGGGAAA-GTGCTTTCTGCGA

>HEID104854_MT047550_Tillandsia_purpurea_Clone_2

TGCAAGATCATTTTACGCCAA---------------TCATACAATTATAAATGCCAACTAATTATAT-------------------------------------------------------------------------------------------------CCATCTTA----CATATTCCTATATATAT------GAATAGATAATGCAATAC---TGTAAATTT-----AGGAAAAA------------GCTAAAAGAAA-ACAATCGGCTATTCAGTAAAAGCCAGC----GTTTTACAGTA--CTCGAT--CGATAGAAG-------TTTGCATACTTACCTCGTGGCCTTTCCTAAACGATGATGCCTTTCGATGACATTTTCGAGCCCCTCCTCAAAGATGAGATCCAAAGCTGCTCTTAGCCCGTAAAGTAGTTGGATGGAAGGCGTATACGGCCAGTAAGTTCCGAGCTTGTAGAACTTCAAGTAGTCATTCCAGTCGAAGAAAACTCTGACCGACTTGGCATTTTTGGAAGCTTCTAGAGCTTTCGGGCTCGCGCAAACAATGCCCATTCCAGTAGGCAGGGAAA-GTGCTTTCTGCGA

>HEID104854_MT047549_Tillandsia_purpurea_Clone_1

TGCAAGATCATTTTACGCCAA---------------TCATACAATTATAAATGCCAACTAATTATAT-------------------------------------------------------------------------------------------------CCATCTTA----CATATTCCTATATATAT------GAATAGATAATGCAATAC---TGTAAATTT-----AGGAAAAA------------GCTAAAAGAAA-ACAATCGGCTATTCAGTAAAAGGCAGC----GTTTTACAGTA--CTCGAT--CGATAGAAG-------TTTGCATACTTACCTCGTGGCCTTTCCTAAACGATGATGCCTTTCGATGACATTTTCGAGCCCCTCCTCAAAGATGAGATCCAAAGCTGCTCTTAGCCCGTAAAGTAGTTGGATGGAAGGCGTATACGGCCAGTAAGTTCCGAGCTTGTAGAACTTCAAGTAGTCATTCCAGTCGAAGAAAACTCTGACCGACTTGGCATTTTTGGAAGCTTCTAGAGCTTTCGGGCTCGCGCAAACAATGCCCATTCCAGTAGGCAGGGAAA-GTGCTTTCTGCGA

>HEID131405_MT047546_Tillandsia_marconae_Clone_1

TGCAAGATCATTTTATGCCAA---------------TCATACAATTATAAATGCCAACTAATTATAT-------------------------------------------------------------------------------------------------CCATCTTA----CATATTCCTATATAT--------GAATAGATAATGCAATAT---TGTAAATTT-----AGGAAAAAA-----------GCTAAAAGAAA-ACAATCGGCTATTCAGTAAAAG----------------------------------------------TTTGCATACTTACCTCGTGGCCTTTCCTAAACGATGATGCCTTTCGATGACATTTTCGAGCCCCTCCTCAAAGATGAGATCCAAAGCTGCTCTTAGCCCGTAAAGTAGTTGGATGGAAGGCGTATACGGCCAGTAAGTTCCAAGCTTGTAGAACTTCAAGTAGTCATTCCAGTCGAAGAAAACTCTGACCGACTTGGCATTTTTGGAAGCTTCTAGAGCTTTCGGGCTCGCGCAAACAATGCCCATTCCAGTAGGCAGGGAAA-GTGCTTTCTGCGA

>HEID103591_MT047545_Tillandsia_marconae_Clone_1

TGCAAGATCATTTTATGCCAA---------------TCATACAATTATAAATGCCAACTAATTATAT-------------------------------------------------------------------------------------------------CCATCTTA----CATATTCCTATATAT--------GAATAGATAATGCAATAT---TGTAAATTT-----AGGAAAAAA-----------GCTAAAAGAAA-ACAATCGGCTATTCAGTAAAAG----------------------------------------------TTTGCATACTTACCTCGTGGCCTTTCCTAAACGATGATGCCTTTCGATGACATTTTCGAGCCCCTCCTCAAAGATGAGATCCAAAGCTGCTCTTAGCCCGTAAAGTAGTTGGATGGAAGGCGTATACGGCCAGTAAGTTCCAAGCTTGTAGAACTTCAAGTAGTCATTCCAGTCGAAGAAAACTCTGACCGACTTGGCATTTTTGGAAGCTTCTAGAGCTTTCGGGCTCGCGCAAACAATGCCCATTCCAGTAGGCAGGGAAA-GTGCTTTCTGCGA

>HEID104553_MT047473_1_Tillandsia_incarnata_BRC239

TGCAAGATCATTTTACGCCAA-----------------------TTATAAATGGCAACTAATTATAT-------------------------------------------------------------------------------------------------CCATCTTA----CATATTCCTATATAT--------GAATAGATAATGCAATAC---TGTAAATTT-----AGGAAAAA------------GCTAAAAAAAA-ACAATCGGCTATT--------------------------------TCGAT--CGATAGA-AG------TTTGCATACTTACCTCGTGGCCTTTCCTAAACGATGATGCCTTTCGATGACATTTTCGAGCCCCTCCTCAAAGATGAGATCCAAAGCTGCTCTTAGCCCGTAAAGTAGTTGGATGGAAGGCGTATACGGCCAGTAAGTTCCAAGCTTGTAGAACTTCAAGTAGTCATTCCAGTCGAAGAAAACTCTGACCGACTTGGCATTTTTTGAAGCTTCTAGAGCTTTCGGGCTCGCGCAAACAATGCCCATTCCAGTAGGCnnnnnnn-nnnnnnnnnnnnn

>HEID113098_MT038714_Tillandsia_landbeckii_BRC381

TGCA---TCATTTTACGCCAATTATGTTACAAGAGGTCATACAATTATAAATGGCAACTAATTACAT-------------------------------------------------------------------------------------------------TCATCTTA----CATATTCCTATATAT--------GAATAGATAATGCAATAC---TGTAAATTT-----AGAAAAAA------------GCTAAAAGAAA-ACAATCGGCTATTCAGTAAAAGCCAGCTTAATTTTTACAGTATACTCGAT--CGATAGATAGA---TTTTTGCATACTTACCTCGTGGCCTTTCCTAAACGACGATGCCTTTCGATGACATTTTCGAGGCCCTCCTCAAAGATGAGATCCAAAGCTGCTCTTAGCCCGTAAAGTAGTTGGATGGAAGGCGTATACGGCCAGTAAGTTCCAAGCTTGTAGAACTTCAAGTAGTCATTCCAGTCGAAGAAAACTCTGACCGACTTGGCATTTTTGGAAGCTTCCAGAGCTTTCGGGCTCGCGCAAACAATGCCCATTCCAGTAGGCAGGGAAA-GTGCTTTCTGCGA

>HEID113099_MT047537_Tillandsia_landbeckii_subsp_andina_Clone113099_1

TGCAAGATCATTTTACGCCAA---------------TCATACAATTATAAATGGCAACTAATTACAT-------------------------------------------------------------------------------------------------TCATCTTA----CATATTCCTATATAT--------GAATAGATAATGCAATAC---TGTAAATTT-----AGAAAAAA------------GCTAAAAGAAA-ACAATTGGCTATTCAGTAAAAGCCAGCTTAATTTTTACAGTATACTCGAT--CGATAGATAGA---TTTTTGCATACTTACCTCGTGGCCTTTCCTAAACGACGATGCCTTTCGATGACATTTTCGAGGCCCTCCTCAAAGATGAGATCCAAAGCTGCTCTTAGCCCGTAAAGTAGTTGGATGGAAGGCGTATACGGCCAGTAAGTTCCAAGCTTGTAGAACTTCAAGTAGTCATTCCAGTCGAAGAAAACTCGGACCGACTTGGCATTTTTGGAAGCTTCCAGAGCTTTCGGGCTCGCGCAAACAATGCCCATTCCAGTAGGCAGGGAAA-GTGCTTTCTGCGA

>HEID113099_MT047536_Tillandsia_landbeckii_subsp_andina_Clone_3

TGCAAGATCATTTTACGCCAA---------------TCATACAATTATAAATGGCAACTAATTACAT-------------------------------------------------------------------------------------------------TCATCTTATATGCATATTCCTATATAT--------GAATAGATAATGCAATAC---TGTAAATTT-----AGAAAAAAA------------CTAAAAGAAA-ACAATTGGCTATTCAGTAAAAGCCAGCTTAATTTTTACAGTATACTCGAT--CGATAGATAGA---TTTTTGCATACTTACCTCGTGGCCTTTCCTAAACGACGATGCCTTTCGATGACATTTTCGAGGCCCTCCTCAAAGATGAGATCCAAAGCTGCTCTTAGCCCGTAAAGTAGTTGGATGGAAGGCGTATACGGCCAGTAAGTTCCGAGCTTGTAGAACTTCAAGTAGTCATTCCAGTCGAAGAAAACTCTGACCGACTTGGCATTTTTGGAAGCTTCCAGAGCTTTCGGGCTCGCGCAAACAATGCCCATTCCAGTAGGCAGGGAAA-GTGCTTTCTGCGA

>HEID131787_MT047535_Tillandsia_virescens_Clone_2

TGCAAGATCATTTTACGCCAA---------------TCATACAATTATAAATGGCAACTAATTGCAT-------------------------------------------------------------------------------------------------TCATCTTATATGCATATTCCTATATAT--------GAATAGATAATGCAATAC---TGTAAATTT-----AGAAAAAAA------------CTAAAAGAAA-ACAATTGGCTATTCAGTAAAAGCCAGCTTAATTTTTACAGTATACTCGAT--CGATAGATAGA---TTTTCGCATACTTACCTCGTGGCCTTTCCTAAACGACGATGCCTTTCGATGACATTTTCGAGGCCCTCCTCAAAGATGAGATCCAAAGCTGCTCTTAGCCCGTAAAGTAGTTGGATGGAAGGCGTATACGGCCAGTAAGTTCCGAGCTTGTAGAACTTCAAGTAGTCATTCCAGTCGAAGAAAACTCTGACCGACTTGGCATTTTTGGAAGCTTCCAGAGCTTTCGGGCTCGCGCAAACAATGCCCATTCCAGTAGGCAGGGAAA-GTGCTTTCTGCGA

>HEID131590_MT047568_Tillandsia_recurvata_Clone_3

TGCAAGATCATTTTACGCCAA---------------TCATACAATTATAAATGGCAACTAATTATAT-------------------------------------------------------------------------------------------------TCATCTTA----CATATTCCTATA----------------------------C---TGTAAATTT-----AAAAAAAAAAAAAAAAA----CTAAAAGAAA-ATAATCGTCTATTCAGTAAAAGCCAGCTTAATTTTTACAGTATACTCGAT--CGATAGA-------TTTTTGCATACTTACCTCGTGGCCTTTCCTAAACGACGATGCCTTTCGATGACATTTTCGAGGCCCTCCTCAAAGATGAGATCCAAAGCTGCTCTTAGCCCGTAAAGTAGTTGGATGGAAGGCGTATACGGCCAGTAAGTTCCAAGATTGTAGAACTTCAAGTAGTCATTCCAGTCGAAGAAAACTCTGACCGACTTGGCATTTTTGGAAGCTTCCAGAGCTTTCGGGCTCGCGCAAACAATTCCCATTCCAGTAGGCAGGGAAA-GTGCTTTCTGCGA

>HEID131590_MT047567_Tillandsia_recurvata_Clone_2

TGCAAGATCATTTTACGCCAA---------------TCATACAATTATAAATGGCAACTAATTATAT-------------------------------------------------------------------------------------------------TCATCTTA----CATATTCCTATA----------------------------C---TGTAAATTT-----AAAAAAAAAAAAAAAAAA---CTAAAAGAAA-ATAATCGTCTATTCAGTAAAAGCCAGCTTAATTTTTACAGTATACTCGAT--CGATAGA-------TTTTTGCATACTTACCTCGTGGCCTTTCCTAAACGACGATGCCTTTCGATGACATTTTCGAGGCCCTCCTCAAAGATGAGATCCAAAGCTGCTCTTAGCCCGTAAAGTAGTTGGATGGAAGGCGTATACGGCCAGTAAGTTCCAAGATTGTAGAACTTCAAGTAGTCATTCCAGTCGAAGAAAACTCTGACCGACTTGGCATTTTTGGAAGCTTCCAGAGCTTTCGGGCTCGCGCAAACAATTCCCATTCCAGTAGGCAGGGAAA-GTGCTTTCTGCGA

>HEID131590_MT047566_Tillandsia_recurvata_Clone_1

TGCAAGATCATTTTACGCCAA---------------TCATACAATTATAAATGGCAACTAATTATAT-------------------------------------------------------------------------------------------------TCATCTTA----CATATTCCTATA----------------------------C---TGTAAATTT-----AAAAAAAAAAAAAAAA-----CTAAAAGAAA-ATAATCGTCTATTCAGTAAAAGCCAGCTTAATTTTTACAGTATACTCGAT--CGATAGA-------TTTTTGCATACTTACCTCGTGGCCTTTCCTAAACGACGATGCCTTTCGATGACATTTTCGAGGCCCTCCTCAAAGATGAGATCCAAAGCTGCTCTTAGCCCGTAAAGTAGTTGGATGGAAGGCGTATACGGCCAGTAAGTTCCAAGATTGTAGAACTTCAAGTAGTCATTCCAGTCGAAGAAAACTCTGACCGACTTGGCATTTTTGGAAGCTTCCAGAGCTTTCGGGCTCGCGCAAACAATTCCCATTCCAGTAGGCAGGGAAA-GTGCTTTCTGCGA

>HEID105087_MT047562_Tillandsia_recurvata_Clone_3

TGCAAGATCATTTTACGCCAA---------------TCATACAATTATAAATGGCAACTAATTATAT-------------------------------------------------------------------------------------------------TCATCTTA----CATATTCCTATA----------------------------C---TGTAAATTT-----AAAAAAAAAAAAAAAAA----TTAAAAGAAA-ATAATCGTCTATTCAGTAAAAGCCAGCTTAATTTTTACAGTATACTCGAT--CGATAGA-------TTTTTGCATACTTACCTCGTGGCCTTTCCTAAACGACGATGCCTTTCGATGACATTTTCGAGCCCCTCCTCAAAGATGAGATCCAAAGCTGCTCTTAGCCCGTAAAGTAGTTGGATGGAAGGCGTATACGGCCAGTAAGTTCCAAGCTTGTAGAACTTCAAATAGTCATTCCAGTCGAAGAAAACTCTGACCGACTTGGCATTTTTGGAAGCTTCCAGAGCTTTCGGGCTCGCGCAAATAATGCCCATTCCAGTAGGCAGGGAAA-GTGCTTTCTGCGA

>HEID105087_MT047561_Tillandsia_recurvata_Clone_2

TGCAAGATCATTTTATGCCAA---------------TCATACAATTATAAATGGCAACTAATTATAT-------------------------------------------------------------------------------------------------TCATCTTA----CATATTCCTATA----------------------------C---TGTAAATTT-----AAAAAAAAAAAAAAAAAAA--TTAAAAGAAA-ATAATCGTCTATTCAGTAAAAGCCAGCTTAATTTTTACAGTATACTCGAT--CGATAGA-------TTTTTGCATACTTACCTCGTGGCCTTTCCTAAACGACGATGCCTTTCGATGACATTTTCGAGCCCCTCCTCAAAGATGAGATCCAAAGCTGCTCTTAGCCCGTAAAGTAGTTGGATGGAAGGCGTATACGGCCAGTAAGTTCCAAGCTTGTAGAACTTCAAATAGTCATTCCAGTCGAAGAAAACTCTGACCGACTTGGCATTTTTGGAAGCTTCCAGAGCTTTCGGGCTCGCGCAAATAATGCCCATTCCAGTAGGCAGGGAAA-GTGCTTTCTGCGA

>HEID105087_MT047560_Tillandsia_recurvata_Clone_1

TGCAAGATCATTTTACGCCAA---------------TCATACAATTATAAATGGCAACTAATTATAT-------------------------------------------------------------------------------------------------TCATCTTA----CATATTCCTATA----------------------------C---TGTAAATTT-----AAAAAAAAAAAAAAAA-----TTAAAAGAAA-ATAATCGTCTATTCAGTAAAAGCCAGCTTAATTTTTACAGTATACTCGAT--CGATAGA-------TTTTTGCATACTTACCTCGTGGCCTTTCCTAAACGACGATGCCTTTCGATGACATTTTCGAGCCCCTCCTCAAAGATGAGATCCAAAGCTGCTCTTAGCCCGTAAAGTAGTTGGATGGAAGGCGTATACGGCCAGTAAGTTCCAAGCTTGTAGAACTTCAAATAGTCATTCCAGTCGAAGAAAACTCTGACCGACTTGGCATTTTTGGAAGCTTCCAGAGCTTTCGGGCTCGCGCAAATAATGCCCATTCCAGTAGGCAGGGAAA-GTGCTTTCTGCGA

>HEID131780_MT047559_Tillandsia_purpurea

TGTAAGATCATTTTACACCAA---------------TCATACAATTATAAATGCCAACTAATTATAT-------------------------------------------------------------------------------------------------ACATCTTA----CATATTCCTATATAT--------GAATAGATAATGCAATAC---TGTAAATTT-----AGGAAAAAA-----------GCTAAAAGAAA-ACAATCGGCTATTCAGTAAAAA----------------------------------------------TTTGCATACTTACCTCGTGGCCTTTCCTAAACGATGATGCCTTTCGATGACATTTTCGAGCCCCTCCTCAAAGATGAGATCCAAAGCTGCTCTTAGCCCGTAAAGTAGTTGGATGGAAGGCGTATACGGCCAGTAAGTTCCAAGCTTGTAGAACTTCAAGTAGTCATTCCAGTCGAAGAAAACTCTGACCGACTTGGCATTTTTGGAAGCTTCTAGAGCTTTCGGGCTCGCGCAAACAATGCCCATTCCAGTAGGCAGGGAAA-GTGCTTTCTGCGA

>HEID131780_MT047558_Tillandsia_purpurea_Clone_3

TGTAAGATCATTTTACACCAA---------------TCATACAATTATAAATGCCAACTAATTATAT-------------------------------------------------------------------------------------------------ACATCTTA----CATATTCCTATATAT--------GAATAGATAATGCAATAC---TGTAAATTT-----AGGAAAAAA-----------GCTAAAAGAAA-ACAATCGGCTATTCAGTAAAAA----------------------------------------------TTTGCATACTTACCTCGTGGCCTTTCCTAAACGATGATGCCTTTCGATGACATTTTCGAGCCCCTCCTCAAAGATGAGATCCAAAGCTGCTCTTAGCCCGTAAAGTAGTTGGATGGAAGGCGTATACGGCCAGTAAGTTCCAAGCTTGTAGAACTTCAAGTAGTCATTCCAGTCGAAGAAAACTCTGACCGACTTGGCATTTTTGGAAGCTTCTAGAGCTTTCGGGCTCGCGCAAACAATGCCCATTCCAGTAGGCAGGGAAA-GTGCTTTCTGCGA

>HEID131780_MT047557_Tillandsia_purpurea_Clone_2

TGTAAGATCATTTTACACCAA---------------TCATACAATTATAAATGCCAACTAATTATAT-------------------------------------------------------------------------------------------------ACATCTTA----CATATTCCTATATAT--------GAATAGATAATGCAATAC---TGTAAATTT-----AGGAAAAAA-----------GCTAAAAGAAA-ACAATCGGCTATTCAGTAAAAA----------------------------------------------TTTGCATACTTACCTCGTGGCCTTTCCTAAACGATGATGCCTTTCGATGACATTTTCGAGCCCCTCCTCAAAGATGAGATCCAAAGCTGCTCTTAGCCCGTAAAGTAGTTGGATGGAAGGCGTATACGGCCAGTAAGTTCCAAGCTTGTAGAACTTCAAGTAGTCATTCCAGTCGAAGAAAACTCTGACCGACTTGGCATTTTTGGAAGCTTCTAGAGCTTTCGGGCTCGCGCAAACAATGCCCATTCCAGTAGGCAGGGAAA-GTGCTTTCTGCGA

>HEID131780_MT047556_Tillandsia_purpurea_Clone_1

TGTAAGATCATTTTACACCAA---------------TCATACAATTATAAATGCCAACTAATTATAT-------------------------------------------------------------------------------------------------ACATCTTA----CATATTCCTATATAT--------GAATAGATAATGCAATAC---TGTAAATTT-----AGGAAAAAA-----------GCTAAAAGAAA-ACAATCGGCTATTCAGTAAAAA----------------------------------------------TTTGCATACTTACCTCGTGGCCTTTCCTAAACGATGATGCCTTTCGATGACATTTTCGAGCCCCTCCTCAAAGATGAGATCCAAAGCTGCTCTTAGCCCGTAAAGTAGTTGGATGGAAGGCGTATACGGCCAGTAAGTTCCAAGCTTGTAGAACTTCAAGTAGTCATTCCAGTCGAAGAAAACTCTGACCGACTTGGCATTTTTGGAAGCTTCTAGAGCTTTCGGGCTCGCGCAAACAATGCCCATTCCAGTAGGCAGGGAAA-GTGCTTTCTGCGA

>HEID100442_MT038713_Tillandsia_landbeckii_BRC380

TGCAAGATCATTTTACGCCAATTATGTTACAAGAGGTCATACAATTATAAATGGCAACTAATTACAT-------------------------------------------------------------------------------------------------TCATCTTA----CATATTCCTATATAT--------GAATAGATAATGCAATAC---TGTAAATTT-----AGAAAAAA------------GCTAAAAGAAA-ACAATCGGCTATTCAGTAAAAGCCAGCTTAATTTTTACAGTATACTCGAT--CGATAGATAGA---TTTTTGCATACTTACCTCGTGGCCTTTCCTAAACGACGATGCCTTTCGATGACATTTTCGAGGCCCTCCTCAAAGATGAGATCCAAAGCTGCTCTTAGCCCGTAAAGTAGTTGGATGGAAGGCGTATACGGCCAGTAAGTTCCAAGCTTGTAGAACTTCAAGTAGTCATTCCAGTCGAAGAAAACTCTGACCGACTTGGCATTTTTGGAAGCTTCCAGAGCTTTCGGGCTCGCGCAAACAATGCCCATTCCAGTAGGCAGGGAAA-GTGCTTTCTGCGA

>HEID113097_MT038719_Tillandsia_landbeckii_BRC386

TGCA---TCATTTTACGCCAATTATGTTACAAGAGGTCATACAATTATAAATGGCAACTAATTACAT-------------------------------------------------------------------------------------------------TCATCTTA----CATATTCCTATATAT--------GAATAGATAATGCAATAC---TGTAAATTT-----AGAAAAAA------------GCTAAAAGAAA-ACAATCGGCTATTCAGTAAAAGCCAGCTTAATTTTTACAGTATACTCGAT--CGATAGATAGA---TTTTTGCATACTTACCTCGTGGCCTTTCCTAAACGACGATGCCTTTCGATGACATTTTCGAGGCCCTCCTCAAAGATGAGATCCAAAGCTGCTCTTAGCCCGTAAAGTAGTTGGATGGAAGGCGTATACGGCCAGTAAGTTCCAAGCTTGTAGAACTTCAAGTAGTCATTCCAGTCGAAGAAAACTCTGACCGACTTGGCATTTTTGGAAGCTTCCAGAGCTTTCGGGCTCGCGCAAACAATGCCCATTCCAGTAGGCAGGGAAA-GTGCTTTCTGCGA

>HEID112451_MT038718_Tillandsia_landbeckii_BRC385

TGCAAGATCATTTTACGCCAATTATGTTACAAGAGGTCATACAATTATAAATGGCAACTAATTACAT-------------------------------------------------------------------------------------------------TCATCTTA----CATATTCCTATATAT--------GAATAGATAATGCAATAC---TGTAAATTT-----AGAAAAAA------------GCTAAAAGAAA-ACAATCGGCTATTCAGTAAAAGCCAGCTTAATTTTTACAGTATACTCGAT--CGATAGATAGA---TTTTTGCATACTTACCTCGTGGCCTTTCCTAAACGACGATGCCTTTCGATGACATTTTCGAGGCCCTCCTCAAAGATGAGATCCAAAGCTGCTCTTAGCCCGTAAAGTAGTTGGATGGAAGGCGTATACGGCCAGTAAGTTCCAAGCTTGTAGAACTTCAAGTAGTCATTCCAGTCGAAGAAAACTCTGACCGACTTGGCATTTTTGGAAGCTTCCAGAGCTTTCGGGCTCGCGCAAACAATGCCCATTCCAGTAGGCAGGGAAA-GTGCTTTCTGCGA

>WU_0001965_MT038717_Tillandsia_landbeckii_BRC384

TGCAAGATCATTTTACGCCAATTATGTTACAAGAGGTCATACAATTATAAATGGCAACTAATTACAT-------------------------------------------------------------------------------------------------TCATCTTA----CATATTCCTATATAT--------GAATAGATAATGCAATAC---TGTAAATTT-----AGAAAAAA------------GCTAAAAGAAA-ACAATCGGCTATTCAGTAAAAGCCAGCTTAATTTTTACAGTATACTCGAT--CGATAGATAGA---TTTTTGCATACTTACCTCGTGGCCTTTCCTAAACGACGATGCCTTTCGATGACATTTTCGAGGCCCTCCTCAAAGATGAGATCCAAAGCTGCTCTTAGCCCGTAAAGTAGTTGGATGGAAGGCGTATACGGCCAGTAAGTTCCAAGCTTGTAGAACTTCAAGTAGTCATTCCAGTCGAAGAAAACTCTGACCGACTTGGCATTTTTGGAAGCTTCCAGAGCTTTCGGGCTCGCGCAAACAATGCCCATTCCAGTAGGCAGGGAAA-GTGCTTTCTGCGA

>HEID112534_MT038716_Tillandsia_landbeckii_BRC383

TGCAAGATCATTTTACGCCAATTATGTTACAAGAGGTCATACAATTATAAATGGCAACTAATTACAT-------------------------------------------------------------------------------------------------TCATCTTA----CATATTCCTATATAT--------GAATAGATAATGCAATAC---TGTAAATTT-----AGAAAAAA------------GCTAAAAGAAA-ACAATCGGCTATTCAGTAAAAGCCAGCTTAATTTTTACAGTATACTCGAT--CGATAGATAGA---TTTTTGCATACTTACCTCGTGGCCTTTCCTAAACGACGATGCCTTTCGATGACATTTTCGAGGCCCTCCTCAAAGATGAGATCCAAAGCTGCTCTTAGCCCGTAAAGTAGTTGGATGGAAGGCGTATACGGCCAGTAAGTTCCAAGCTTGTAGAACTTCAAGTAGTCATTCCAGTCGAAGAAAACTCTGACCGACTTGGCATTTTTGGAAGCTTCCAGAGCTTTCGGGCTCGCGCAAACAATGCCCATTCCAGTAGGCAGGGAAA-GTGCTTTCTGCGA

>HEID100441_MT038715_Tillandsia_landbeckii_BRC382

TGCAAGATCATTTTACGCCAATTATGTTACAAGAGGTCATACAATTATAAATGGCAACTAATTACAT-------------------------------------------------------------------------------------------------TCATCTTA----CATATTCCTATATAT--------GAATAGATAATGCAATAC---TGTAAATTT-----AGAAAAAA------------GCTAAAAGAAA-ACAATCGGCTATTCAGTAAAAGCCAGCTTAATTTTTACAGTATACTCGAT--CGATAGATAGA---TTTTTGCATACTTACCTCGTGGCCTTTCCTAAACGACGATGCCTTTCGATGACATTTTCGAGGCCCTCCTCAAAGATGAGATCCAAAGCTGCTCTTAGCCCGTAAAGTAGTTGGATGGAAGGCGTATACGGCCAGTAAGTTCCAAGCTTGTAGAACTTCAAGTAGTCATTCCAGTCGAAGAAAACTCTGACCGACTTGGCATTTTTGGAAGCTTCCAGAGCTTTCGGGCTCGCGCAAACAATGCCCATTCCAGTAGGCAGGGAAA-GTGCTTTCTGCGA

>HEID100441_MT047577_1_Tillandsia_landbeckii_subsp_landbeckii

TGCAAGATCATTTTACGCCAATTATGTTACAAGAGGTCATACAATTATAAATGGCAACTAATTACAT-------------------------------------------------------------------------------------------------TCATCTTA----CATATTCCTATATAT--------GAATAGATAATGCAATAC---TGTAAATTT-----AGAAAAAA------------GCTAAAAGAAA-ACAATCGGCTATTCAGTAAAAGCCAGCTTAATTTTTACAGTATACTCGAT--CGATAGATAGA---TTTTTGCATACTTACCTCGTGGCCTTTCCTAAACGACGATGCCTTTCGATGACATTTTCGAGGCCCTCCTCAAAGATGAGATCCAAAGCTGCTCTTAGCCCGTAAAGTAGTTGGATGGAAGGCGTATACGGCCAGTAAGTTCCAAGCTTGTAGAACTTCAAGTAGTCATTCCAGTCGAAGAAAACTCTGACCGACTTGGCATTTTTGGAAGCTTCCAGAGCTTTCGGGCTCGCGCAAACAATGCCCATTCCAGTAGGCAGGGAAA-GTGCTTTCTGCGA

>HEID112451_MT047576_Tillandsia_landbeckii

TGCAAGATCATTTTACGCCAATTATGTTACAAGAGGTCATACAATTATAAATGGCAACTAATTACAT-------------------------------------------------------------------------------------------------TCATCTTA----CATATTCCTATATAT--------GAATAGATAATGCAATAC---TGTAAATTT-----AGAAAAAA------------GCTAAAAGAAA-ACAATCGGCTATTCAGTAAAAGCCAGCTTAATTTTTACAGTATACTCGAT--CGATAGATAGA---TTTTTGCATACTTACCTCGTGGCCTTTCCTAAACGACGATGCCTTTCGATGACATTTTCGAGGCCCTCCTCAAAGATGAGATCCAAAGCTGCTCTTAGCCCGTAAAGTAGTTGGATGGAAGGCGTATACGGCCAGTAAGTTCCAAGCTTGTAGAACTTCAAGTAGTCATTCCAGTCGAAGAAAACTCTGACCGACTTGGCATTTTTGGAAGCTTCCAGAGCTTTCGGGCTCGCGCAAACAATGCCCATTCCAGTAGGCAGGGAAA-GTGCTTTCTGCGA

>HEID112534_MT047575_Tillandsia_landbeckii

TGCAAGATCATTTTACGCCAATTATGTTACAAGAGGTCATACAATTATAAATGGCAACTAATTACAT-------------------------------------------------------------------------------------------------TCATCTTA----CATATTCCTATATAT--------GAATAGATAATGCAATAC---TGTAAATTT-----AGAAAAAA------------GCTAAAAGAAA-ACAATCGGCTATTCAGTAAAAGCCAGCTTAATTTTTACAGTATACTCGAT--CGATAGATAGA---TTTTTGCATACTTACCTCGTGGCCTTTCCTAAACGACGATGCCTTTCGATGACATTTTCGAGGCCCTCCTCAAAGATGAGATCCAAAGCTGCTCTTAGCCCGTAAAGTAGTTGGATGGAAGGCGTATACGGCCAGTAAGTTCCAAGCTTGTAGAACTTCAAGTAGTCATTCCAGTCGAAGAAAACTCTGACCGACTTGGCATTTTTGGAAGCTTCCAGAGCTTTCGGGCTCGCGCAAACAATGCCCATTCCAGTAGGCAGGGAAA-GTGCTTTCTGCGA

>HEID113094_MT047574_Tillandsia_landbeckii_var_rigidor

TGCAAGATCATTTTACGCCAATTATGTTACAAGAGGTCATACAATTATAAATGGCAACTAATTACAT-------------------------------------------------------------------------------------------------TCATCTTA----CATATTCCTATATAT--------GAATAGATAATGCAATAC---TGTAAATTT-----AGAAAAAA------------GCTAAAAGAAA-ACAATCGGCTATTCAGTAAAAGCCAGCTTAATTTTTACAGTATACTCGAT--CGATAGATAGA---TTTTTGCATACTTACCTCGTGGCCTTTCCTAAACGACGATGCCTTTCGATGACATTTTCGAGGCCCTCCTCAAAGATGAGATCCAAAGCTGCTCTTAGCCCGTAAAGTAGTTGGATGGAAGGCGTATACGGCCAGTAAGTTCCAAGCTTGTAGAACTTCAAGTAGTCATTCCAGTCGAAGAAAACTCTGACCGACTTGGCATTTTTGGAAGCTTCCAGAGCTTTCGGGCTCGCGCAAACAATGCCCATTCCAGTAGGCAGGGAAA-GTGCTTTCTGCGA

>HEID100442_MT047573_Tillandsia_landbeckii_subsp_andina

TGCAAGATCATTTTACGCCAATTATGTTACAAGAGGTCATACAATTATAAATGGCAACTAATTACAT-------------------------------------------------------------------------------------------------TCATCTTA----CATATTCCTATATAT--------GAATAGATAATGCAATAC---TGTAAATTT-----AGAAAAAA------------GCTAAAAGAAA-ACAATCGGCTATTCAGTAAAAGCCAGCTTAATTTTTACAGTATACTCGAT--CGATAGATAGA---TTTTTGCATACTTACCTCGTGGCCTTTCCTAAACGACGATGCCTTTCGATGACATTTTCGAGGCCCTCCTCAAAGATGAGATCCAAAGCTGCTCTTAGCCCGTAAAGTAGTTGGATGGAAGGCGTATACGGCCAGTAAGTTCCAAGCTTGTAGAACTTCAAGTAGTCATTCCAGTCGAAGAAAACTCTGACCGACTTGGCATTTTTGGAAGCTTCCAGAGCTTTCGGGCTCGCGCAAACAATGCCCATTCCAGTAGGCAGGGAAA-GTGCTTTCTGCGA

>HEID113095_MT047572_Tillandsia_landbeckii_subsp_andina

TGCA---TCATTTTACGCCAATTATGTTACAAGAGGTCATACAATTATAAATGGCAACTAATTACAT-------------------------------------------------------------------------------------------------TCATCTTA----CATATTCCTATATAT--------GAATAGATAATGCAATAC---TGTAAATTT-----AGAAAAAA------------GCTAAAAGAAA-ACAATCGGCTATTCAGTAAAAGCCAGCTTAATTTTTACAGTATACTCGAT--CGATAGATAGA---TTTTTGCATACTTACCTCGTGGCCTTTCCTAAACGACGATGCCTTTCGATGACATTTTCGAGGCCCTCCTCAAAGATGAGATCCAAAGCTGCTCTTAGCCCGTAAAGTAGTTGGATGGAAGGCGTATACGGCCAGTAAGTTCCAAGCTTGTAGAACTTCAAGTAGTCATTCCAGTCGAAGAAAACTCTGACCGACTTGGCATTTTTGGAAGCTTCCAGAGCTTTCGGGCTCGCGCAAACAATGCCCATTCCAGTAGGCAGGGAAA-GTGCTTTCTGCGA

>HEID113096_MT047571_Tillandsia_landbeckii_subsp_andina

TGCA---TCATTTTACGCCAATTATGTTACAAGAGGTCATACAATTATAAATGGCAACTAATTACAT-------------------------------------------------------------------------------------------------TCATCTTA----CATATTCCTATATAT--------GAATAGATAATGCAATAC---TGTAAATTT-----AGAAAAAA------------GCTAAAAGAAA-ACAATCGGCTATTCAGTAAAAGCCAGCTTAATTTTTACAGTATACTCGAT--CGATAGATAGA---TTTTTGCATACTTACCTCGTGGCCTTTCCTAAACGACGATGCCTTTCGATGACATTTTCGAGGCCCTCCTCAAAGATGAGATCCAAAGCTGCTCTTAGCCCGTAAAGTAGTTGGATGGAAGGCGTATACGGCCAGTAAGTTCCAAGCTTGTAGAACTTCAAGTAGTCATTCCAGTCGAAGAAAACTCTGACCGACTTGGCATTTTTGGAAGCTTCCAGAGCTTTCGGGCTCGCGCAAACAATGCCCATTCCAGTAGGCAGGGAAA-GTGCTTTCTGCGA

>HEID113097_MT047570_Tillandsia_landbeckii_subsp_andina

TGCA---TCATTTTACGCCAATTATGTTACAAGAGGTCATACAATTATAAATGGCAACTAATTACAT-------------------------------------------------------------------------------------------------TCATCTTA----CATATTCCTATATAT--------GAATAGATAATGCAATAC---TGTAAATTT-----AGAAAAAA------------GCTAAAAGAAA-ACAATCGGCTATTCAGTAAAAGCCAGCTTAATTTTTACAGTATACTCGAT--CGATAGATAGA---TTTTTGCATACTTACCTCGTGGCCTTTCCTAAACGACGATGCCTTTCGATGACATTTTCGAGGCCCTCCTCAAAGATGAGATCCAAAGCTGCTCTTAGCCCGTAAAGTAGTTGGATGGAAGGCGTATACGGCCAGTAAGTTCCAAGCTTGTAGAACTTCAAGTAGTCATTCCAGTCGAAGAAAACTCTGACCGACTTGGCATTTTTGGAAGCTTCCAGAGCTTTCGGGCTCGCGCAAACAATGCCCATTCCAGTAGGCAGGGAAA-GTGCTTTCTGCGA

>HEID113098_MT047569_Tillandsia_landbeckii_subsp_andina

TGCA---TCATTTTACGCCAATTATGTTACAAGAGGTCATACAATTATAAATGGCAACTAATTACAT-------------------------------------------------------------------------------------------------TCATCTTA----CATATTCCTATATAT--------GAATAGATAATGCAATAC---TGTAAATTT-----AGAAAAAA------------GCTAAAAGAAA-ACAATCGGCTATTCAGTAAAAGCCAGCTTAATTTTTACAGTATACTCGAT--CGATAGATAGA---TTTTTGCATACTTACCTCGTGGCCTTTCCTAAACGACGATGCCTTTCGATGACATTTTCGAGGCCCTCCTCAAAGATGAGATCCAAAGCTGCTCTTAGCCCGTAAAGTAGTTGGATGGAAGGCGTATACGGCCAGTAAGTTCCAAGCTTGTAGAACTTCAAGTAGTCATTCCAGTCGAAGAAAACTCTGACCGACTTGGCATTTTTGGAAGCTTCCAGAGCTTTCGGGCTCGCGCAAACAATGCCCATTCCAGTAGGCAGGGAAA-GTGCTTTCTGCGA

>HEID131923_MT047565_Tillandsia_recurvata_Clone_3

TGCAAGATCATTTTACGCCAATTATGTTACAAGAGGTCATACAATTATAAATGGCAACTAATTACAT-------------------------------------------------------------------------------------------------TCATCTTATATGCATATTCCTATATAT--------GAATAGATAATGCAATAC---TGTAAATTT-----AGAAAAAAA------------CTAAAAGAAA-ACAATTGACTATTCAGTAAAAGCCAGCTTAATTTTTACAGTATACTCGAT--CGATAGATAGA---TTTTTGCATACTTACCTCGTGGCCTTTCCTAAACGACGATGCCTTTCGATGACATTTTCGAGGCCCTCCTCAAAGATGAGATCCAAAGCTGCTCTTAGCCCGTAAAGTAGTTGGATGGAAGGCGTATACGGCCAGTAAGTTCCAAGCTTGTAGAACTTCAAGTAGTCATTCCAGTCGAAGAAAACTCGGACCGACTTGGCATTTTTGGAAGCTTCCAGAGCTTTTGGGCTCGCGCAAACAATGCCCATTCCAGTAGGCAGGGAAA-GTGCTTTCTGCGA

>HEID131923_MT047564_Tillandsia_recurvata_Clone_2

TGCAAGATCATTTTACGCCAATTATGTTACAAGAGGTCATACAATTATAAATGGCAACTAATTACAT-------------------------------------------------------------------------------------------------TCATCTTATATGCATATTCCTATATAT--------GAATAGATAATGCAATAC---TGTAAATTT-----AGAAAAAA------------GCTAAAAGAAA-ACAATTGGCTATTCAGTAAAAGTCAGCTTAATTTTTACAGTATACTCGAT--CGATAGA-------TTTTTGCATACTTACCTCGTGGCCTTTCCTAAACGACGATGCCTTTCGATGACATTTTCGAGGCCCTCCTCAAAGATGAGATCCAAAGCTGCTCTTAGCCCGTAAAGTAGTTGGATGGAAGGCGTATACGGCCAGTAAGTTCCGAGCTTGTAGAACTTCAAGTAGTCATTCCAGTCGAAGAAAACTCTGACCGACTTGGCATTTTTGGAAGCTTCCAGAGCTTTCGGGCTCGCGCAAACAATGCCCATTCCAGTAGGCAGGGAAA-GTGCTTTCTGCGA

>HEID131923_MT047563_Tillandsia_recurvata_Clone_1

TGCAAGATCATTTTACGCCAATTATGTTACAAGAGGTCATACAATTATAAATGGCAACTAATTACAT-------------------------------------------------------------------------------------------------TCATCTTATATGCATATTCCTATATAT--------GAATAGATAATGCAATAC---TGTAAATTT-----AGAAAAAA------------GCTAAAAGAAA-ACAATTGGCTATTCAGTAAAAGTCAGCTTAATTTTTACAGTATACTCGAT--CGATAGA-------TTTTTGCATACTTACCTCGTGGCCTTTCCTAAACGACGATGCCTTTCGATGACATTTTCGAGGCCCTCCTCAAAGATGAGATCCAAAGCTGCTCTTAGCCCGTAAAGTAGTTGGATGGAAGGCGTATACGGCCAGTAAGTTCCGAGCTTGTAGAACTTCAAGTAGTCATTCCAGTCGAAGAAAACTCTGACCGACTTGGCATTTTTGGAAGCTTCCAGAGCTTTCGGGCTCGCGCAAACAATGCCCATTCCAGTAGGCAGGGAAA-GTGCTTTCTGCGA

>HEID131405_MT047548_Tillandsia_marconae_Clone_3

TGCAAGATCATTTTACGCCAATTATGTTACAAGAGGTCATACAATTATAAATGGCAACTAATTACAT-------------------------------------------------------------------------------------------------TCATCTTA----CATATTCCTATATAT--------GAATAGATAATGCAATAC---TGTAAATTT-----AGAAAAAA------------GCTAAAAGAAA-ACAATCGGCTATTCAGTAAAAGCCAGCTTAATTTTTACAGTATACTCGAT--CGATAGATAGA---TTTTTGCATACTTACCTCGTGGCCTTTCCTAAACGACGATGCCTTTCGATGACATTTTCGAGGCCCTCCTCAAAGATGAGATCCAAAGCTGCTCTTAGCCCGTAAAGTAGTTGGATGGAAGGCGTATACGGCCAGTAAGTTCCAAGCTTGTAGAACTTCAAGTAGTCATTCCAGTCGAAGAAAACTCTGACCGACTTGGCATTTTTGGAAGCTTCCAGAGCTTTCGGGCTCGCGCAAACAATGCCCATTCCAGTAGGCAGGGAAA-GTGCTTTCTGTGA

>HEID131405_MT047547_Tillandsia_marconae_Clone_2

TGCAAGATCATTTTACGCCAATTATGTTACAAGAGGTCATACAATTATAAATGGCAACTAATTACAT-------------------------------------------------------------------------------------------------TCATCTTA----CATATTCCTATATAT--------GAATAGATAATGCAATAC---TGTAAATTT-----AGAAAAAA------------GCTAAAAGAAA-ACAATCGGCTATTCAGTAAAAGCCAGCTTAATTTTTACAGTATACTCGAT--CGATAGATAGA---TTTTTGCATACTTACCTCGTGGCCTTTCCTAAACGACGATGCCTTTCGATGACATTTTCGAGGCCCTCCTCAAAGATGAGATCCAAAGCTGCTCTTAGCCCGTAAAGTAGTTGGATGGAAGGCGTATACGGCCAGTAAGTTCCAAGCTTGTAGAACTTCAAGTAGTCATTCCAGTCGAAGAAAACTCTGACCGACTTGGCATTTTTGGAAGCTTCCAGAGCTTTCGGGCTCGCGCAAACAATGCCCATTCCAGTAGGCAGGGAAA-GTGCTTTCTGCGA

>HEID103591_MT047544_Tillandsia_marconae_Clone_3

TGCAAGATCATTTTACGCCAATTATGTTACAAGAGGTCATACAATTATAAATGGCAACTAATTACAT-------------------------------------------------------------------------------------------------TCATCTTA----CATATTCCTATATAT--------GAATAGATAATGCAATAC---TGTAAATTT-----AGAAAAAA------------GCTAAAAGAAA-ACAATCGGCTATTCAGTAAAAGCCAGCTTAATTTTTACAGTATACTCGAT--CGATAGATAGA---TTTTTGCATACTTACCTCGTGGCCTTTCCTAAACGACGATGCCTTTCGATGACATTTTCGAGGCCCTCCTCAAAGATGAGATCCAAAGCTGCTCTTAGCCCGTAAAGTAGTTGGATGGAAGGCGTATACGGCCAGTAAGTTCCAAGCTTGTAGAACTTCAAGTAGTCATTCCAGTCGAAGAAAACTCTGACCGACTTGGCATTTTTGGAAGCTTCCAGAGCTTTCGGGCTCGCGCAAACAATGCCCATTCCAGTAGGCAGGGAAA-GTGCTTTCTGCGA

>HEID103591_MT047543_Tillandsia_marconae_Clone_2

TGCAAGATCATTTTACGCCAATTATGTTACAAGAGGTCATACAATTATAAATGGCAACTAATTACAT-------------------------------------------------------------------------------------------------TCATCTTA----CATATTCCTATATAT--------GAATAGATAATGCAATAC---TGTAAATTT-----AGAAAAAA------------GCTAAAAGAAA-ACAATCGGCTATTCAGTAAAAGCCAGCTTAATTTTTACAGTATACTCGAT--CGATAGATAGA---TTTTTGCATACTTACCTCGTGGCCTTTCCTAAACGACGATGCCTTTCGATGACATTTTCGAGGCCCTCCTCAAAGATGAGATCCAAAGCTGCTCTTAGCCCGTAAAGTAGTTGGATGGAAGGCGTATACGGCCAGTAAGTTCCAAGCTTGTAGAACTTCAAGTAGTCATTCCAGTCGAAGAAAACTCTGACCGACTTGGCATTTTTGGAAGCTTCCAGAGCTTTCGGGCTCGCGCAAACAATGCCCATTCCAGTAGGCAGGGAAA-GTGCTTTCTGCGA

>HEID131739_MT047541_Tillandsia_virescens

TGCAAGATCATTTTACGCCAATTATGTTACAAGAGGTCATACAATTATAAATGGCAACTAATTACAT-------------------------------------------------------------------------------------------------TCATCTTATATGCATATTCCTATATAT--------GAATAGATAATGCAATAC---TGTGAATTT-----AGAAAAAA------------GCTAAAAGAAA-ACAATCGGCTATTCAGTAAAAGCCAGCTTAATTTTTACAGTATACTCGAT--CGATAGATAGA---TTTTTGCATACTTACCTCGTGGCCTTTCCTAAACGACGATGCCTTTCGATGACATTTTCGAGGCCCTCCTCAAAGATGAGATCCAAAGCTGCTCTTAGCCCGTAAAGTAGTTGGATGGAAGGCGTATACGGCCAGTAAGTTCCAAGCTTGTAGAACTTCAAGTAGTCATTCCAGTCGAAGAAAACTCTGACCGACTTGGCATTTTTGGAAGCTTCCAGAGCTTTCGGGCTCGCGCAAACAATGCCCATTCCAGTAGGCAGGGAAA-GTGCTTTCTGCGA

>HEID131787_MT047534_Tillandsia_virescens_Clone_3

TGCAAGATCATTTTACGGCAATTATATTACAAGAGGTCATACAATTATAAATGGCAACTAATTGCAT-------------------------------------------------------------------------------------------------TCATTTTATATGCATATTCCTATATAT--------GAATAGATAATGCAATAC---TGTAAATTT-----AGAAAAAA------------GCTAAAAGAAA-ACAATCGGCTATTCAGTAAAAGCCAGTTTAATTTTTACAGTATACTCGAT--CGATAGATAGA---TTTTTGCATACTTACCTCGTGGCCTTTCCTAAACGACGATGCCTTTCGATGACATTTTCGAGGCCCTCCTCAAAGATGAGATCCAAAGCTGCTCTTAGCCCGTAAAGTAGTTGGATGGAAGGCGTATACGGCCAGTAAGTTCCAAGTTTGTAGAACTTCAAGTAGTCATTCCAGTCGAAGAAAACTCTGACCGACTTGGCATTCTTGGAAGCTTCCAGAGCTTTCGGGCTCGCGCAAACAATGCCCATTCCAGTAGGCAGGGAAA-GTGCTTTCTGCGA

>HEID131787_MT047533_Tillandsia_virescens_Clone_1

TGCAAGATCATTTTACGGCAATTATATTACAAGAGGTCATACAATTATAAATGGCAACTAATTGCAT-------------------------------------------------------------------------------------------------TCATCTTATATGCATATTCCTATATAT--------GAATAGATAATGCAATAC---TGTAAATTT-----AGAAAAAAA------------CTAAAAGAAA-ACAATTGGCTATTCAGTAAAAGCCAGCTTAATTTTTACAGTATACTCGAT--CGATAGATAGA---TTTTCGCATACTTACCTCGTGGCCTTTCCTAAACGACGATGCCTTTCGATGACATTTTCGAGGCCCTCCTCAAAGATGAGATCCAAAGCTGCTCTTAGCCCGTAAAGTAGTTGGATGGAAGGCGTATACGGCCAGTAAGTTCCGAGCTTGTAGAACTTCAAGTAGTCATTCCAGTCGAAGAAAACTCTGACCGACTTGGCATTTTTGGAAGCTTCCAGAGCTTTCGGGCTCGCGCAAACAATGCCCATTCCAGTAGGCAGGGAAA-GTGCTTTCTGCGA

>MHJB_B1050_Tillandsia_duratii_Clone1

TGCGAGATCATTTTATGCCAATTATGTTACAAGAGGTCATACAATTATAAATGGCAACTAATTACAT-------------------------------------------------------------------------------------------------CCCTCTTA----CATATTCC------------------TAGATAATGCAATAC---TGTAAATTT-----AGAAAAAA------------GCTAAAAGAAA-ACAATCGGCTATTCAGTAAAAGACAGCTTAATTTTTACAGTATACTCGAT--CGATAGA-------TTTTTGCATACTTACCTCGTGGCCTTTCCTAAACGACGATGCCTTTCGATGACATTTTCGAGGCCCTCCTCAAAGATGAGATCCAAAGCTGCTCTTAGCCCGTAAAGTAGTTGGATGGAAGGCGTATACGGCCAGTAAGTTCCAAGCTTGTAGAACTTCAAGTAGTCATTCCAGTCGAAGAAAACTCTGACTGACTTGGCATTTTTGGAAGCTTCCAGAGCTTTCGGGCTCGCGCAAACAATGCCCATTCCAGTAGGCAGGGAAA-GTGCTTTCTGCGA

>HBV-WU0024707_Tillandsia_krahnii

TGCAAGAACATTTTACGCCAATTATGTTACAAGAGGTCATACAATTATAAATGCCAACTAATTATAT-------------------------------------------------------------------------------------------------CCATCTTA----CATACTCCTATGTAT--------GAATAGATAATGCAATAC---TGTAAATTT-----AGGAAAAA------------GCTAAAAGAAA-ACAATCGGCTATT--------------------------------TCGAT--CGATAGA-AG------TTTGCATACTTACCTCGTGGCCTTTCCTAAACGACGATGCCTTTCGATGACATTTTCGAGCCCCTCCTCAAAGATGAGATCCAAAGCTGCTCTTAGCCCGTAAAGTAGTTGGATGGAAGGCGTATACGGCCAGTAAGTTCCAAGCTTGTAGAACTTCAAGTAGTCATTCCAGTCGAAGAAAACTCTGACCGACTTGGCATTTTTTGAAGCTTCTAGAGCTTTCGGGCTCGCGCAAACAATGCCCATTCCAGTAGGCAGGGAAA-GTGCTTTCTGCGA

>HBV-WU0025293_Tillandsia_kuehhasii

TGCAAGATCATTTTACGCCAATTATGTTACAAGAGGTCATACAATTATAAATGGCAACTAATTACAT-------------------------------------------------------------------------------------------------TCATCTTA----CATATTCCTATATAT--------GAATAGATAATGCAATAC---TGTAAATTT-----AGAAAAAA------------GCTAAAAGAAA-ACAATCGACTATTCAGTAAAAGCCAGCTTAATTTTTACAGTATACTCGAT--GGATAGATAGA---TTTTTGCATACTTACCTCGTGGCCTTTCCTAAACGACGATGCCTTTCGATGACATTTTCGAGGCCCTCCTCAAAGATGAGATCCAAAGCTGCTCTTAGCCCGTAAAGTAGTTGGATGGAAGGCGTATACGGCCAGTAAGTTCCAAGCTTGTAGAACTTCAAGTAGTCGTTCCAGTCGAAGAAAACTCTGACCGACTTGGCATTTTTGGAAGCTTCCAGAGCTTTCGGGCTCGCGCAAACAATGCCCATTCCAGTAGGCAGGGAAA-GTGCTTTCTGCGA

>HBV-WU0025293_Tillandsia_kuehhasii_Clone1

TGCAAGATCATTTTACGCCAATTATGTTACAAGAGGTCATACAATTATAAATGGCAACTAATTACAT-------------------------------------------------------------------------------------------------TCATCTTA----CATATTCCTATATAT--------GAATAGATAATGCAATAC---TGTAAATTT-----AGAAAAAA------------GCTAAAAGAAA-ACAATCGACTATTCAGTAAAAGCCAGCTTAATTTTTACAGTATACTCGAT--GGATAGATAGA---TTTTTGCATACTTACCTCGTGGCCTTTGCTAAACGACGATGCCTTTCGATGACATTTTCGAGGCCCTCCTCAAAGATGAGATCCAAAGCTGCTCTTAGCCCGTAAAGTAGTTGGATGGAAGGCGTATACGGCCAGTAAGTTCCAAGCTTGTAGAACTTCAAGTAGTCGTTCCAGTCGAAGAAAACTCTGACCGACTTGGCATTTTTGGAAGCTTCCAGAGCTTTCGGGCTCGCGCAAACAATGCCCATTCCAGTAGGCAGGGAAA-GTGCTTTCTGCGA

>HBV-WU0025293_Tillandsia_kuehhasii_Clone2

TGCAAGATCATTTTACGCCAATTATGTTACAAGAGGTCATACAATTATAAATGGCAACTAATTACAT-------------------------------------------------------------------------------------------------TCATCTTA----CATATTCCTATATAT--------GAATAGATAATGCAATAC---TGTAAATTT-----AGAAAAAA------------GCTAAAAGAAA-ACAATCGACTATTCAGTAAAAGCCAGCTTAATTTTTACAGTATACTCGAT--GGATAGATAGA---TTTTTGCATACTTACCTCGTGGCCTTTCCTAAACGACGATGCCTTTCGATGACATTTTCGAGGCCCTCCTCAAAGATGAGATCCAAAGCTGCTCTTAGCCCGTAAAGTAGTTGGATGGAAGGCGTATACGGCCAGTAAGTTCCAAGCTTGTAGAACTTCAAGTAGTCGTTCCAGTCGAAGAAAACTCTGACCGACTTGGCATTTTTGGAAGCTTCCAGAGCTTTCGGGCTCGCGCAAACAATGCCCATTCCAGTAGGCAGGGAAA-GTGCTTTCTGCGA

>HBV-WU0025293_Tillandsia_kuehhasii_Clone3

TGCAAGATCATTTTACGCCAATTATGTTACAAGAGGTCATACAATTATAAATGGCAACTAATTACAT-------------------------------------------------------------------------------------------------TCATCTTA----CATATTCCTATATAT--------GAATAGATAATGCAATAC---TGTAAATTT-----AGAAAAAA------------GCTAAAAGAAA-ACAATCGACTATTCAGTAAAAGCCAGCTTAATTTTTACAGTATACTCGAT--GGATAGATAGA---TTTTTGCATACTTACCTCGTGGCCTTTGCTAAACGACGATGCCTTTCGATGACATTTTCGAGGCCCTCCTCAAAGATGAGATCCAAAGCTGCTCTTAGCCCGTAAAGTAGTTGGATGGAAGGCGTATACGGCCAGTAAGTTCCAAGCTTGTAGAACTTCAAGTAGTCGTTCCAGTCGAAGAAAACTCTGACCGACTTGGCATTTTTGGAAGCTTCCAGAGCTTTCGGGCTCGCGCAAACAATGCCCATTCCAGTAGGCAGGGAAA-GTGCTTTCTGCGA

>HBV-WU0029532_Tillandsia_lotteae

TGCAAGATCATTTTACGCCAA---------------TCATACAATTATAAATGGCAACTAATTACAT-------------------------------------------------------------------------------------------------CCATCTTA----CATATTCCTATATAT--------GAATAGATAATGCAATAC---TGTAAATTT-----AGAAAACA------------GCTAAAAGAAACACAATCGGCTATTCAGTAAAAGCCAGCTTAATTTTTACAGTATACTCGAT--CGATAGA------TTTTTTGCATACTTACCTCGTGGCCTTTCCTAAACGACGATGCCTTTCGATGACATTTTCGAGCCCCTCCTCAAAGATGAGATCCAAAGCTGCTCTTAGCCCGTAAAGTAGTTGGATGGAAGGCGTATACGGCCAGTAAGTTCCAAGCTTGTAGAACTTCAAGTAGTCATTCCAGTCGAAGAAAACTCTAACCGACTTGGCATTTTTGGAAGCTTCCAGAGCTTTCGGGCTCGCGCAAACAATGCCCATTCCAGTAGGCAGGGAAA-GTGCTTTCTGCGA

>HBV-WU0035551_Tillandsia_lotteae_Clone1

TGCAAGATCATTTTACGCCAATTATGTTACAAGAGGTCATACAATTATAAATGGCAACTAATTACAT-------------------------------------------------------------------------------------------------TCATCTTA----CATATTCCTATATAT--------GAATAGATAATGCAATAC---TGTAAATTT-----AGAAAAAA------------GCTAAAAGAAA-ACAATCGACTATTCAGTAAAAGCCAGCTTAATTTTTACAGTATACTCGAT--GGATAGATAGA---TTTTTGCATACTTACCTCGTGGCCTTTCCTAAACGACGATGCCTTTCGATGACATTTTCGAGGCCCTCCTCAAAGATGAGATCCAAAGCTGCTCTTAGCCCGTAAAGTAGTTGGATGGAAGGCGTATACGGCCAGTAAGTTCCAAGCTTGTAGAACTTCAAGTAGTCGTTCCAGTCGAAGAAAACTCTGACCGACTTGGCATTTTTGGAAGCTTCCAGAGCTTTCGGGCTCGCGCAAACAATGCCCATTCCAGTAGGCAGGGAAA-GTGCTTTCTGCGA

>HBV-WU0035551_Tillandsia_lotteae_Clone2

TGCAAGATCATTTTACGCCAATTATGTTACAAGAGGTCATACAATTATAAATGGCAACTAATTACAT-------------------------------------------------------------------------------------------------TCATCTTA----CATATTCCTATATAT--------GAATAGATAATGCAATAC---TGTAAATTT-----AGAAAAAA------------GCTAAAAGAAA-ACAATCGACTATTCAGTAAAAGCCAGCTTAATTTTTACAGTATACTCGAT--GGATAGATAGA---TTTTTGCATACTTACCTCGTGGCCTTTCCTAAACGACGATGCCTTTCGATGACATTTTCGAGGCCCTCCTCAAAGATGAGATCCAAAGCTGCTCTTAGCCCGTAAAGTAGTTGGATGGAAGGCGTATACGGCCAGTAAGTTCCAAGCTTGTAGAACTTCAAGTAGTCGTTCCAGTCGAAGAAAACTGTGACCGACTTGGCATTTTTGGAAGCTTCCAGAGCTTTCGGGCTCGCGCAAACAATGCCCATTCCAGTAGGCAGGGAAA-GTGCTTTCTGCGA

>HBV-WU0035551_Tillandsia_lotteae_Clone3

TGCAAGATCATTTTACGCCAATTATGTTACAAGAGGTCATACAATTATAAATGGCAACTAATTACAT-------------------------------------------------------------------------------------------------TCATCTTA----CATATTCCTATATAT--------GAATAGATAATGCAATAC---TGTAAATTT-----AGAAAAAA------------GCTAAAAGAAA-ACAATCGACTATTCAGTAAAAGCCAGCTTAATTTTTACAGTATACTCGAT--GGATAGATAGA---TTTTTGCATACTTACCTCGTGGCCTTTCCTAAACGACGATGCCTTTCGATGACATTTTCGAGGCCCTCCTCAAAGATGAGATCCAAAGCTGCTCTTAGCCCGTAAAGTAGTTGGATGGAAGGCGTATACGGCCAGTAAGTTCCAAGCTTGTAGAACTTCAAGTAGTCGTTCCAGTCGAAGAAAACTCTGACCGACTTGGCATTTTTGGAAGCTTCCAGAGCTTTCGGGCTCGCGCAAACAATGCCCATTCCAGTAGGCAGGGAAA-GTGCTTTCTGCGA

>HEID100441_Tillandsia_landbeckii_subsp__landbeckii

TGCAAGATCATTTTACGCCAATTATGTTACAAGAGGTCATACAATTATAAATGGCAACTAATTACAT-------------------------------------------------------------------------------------------------TCATCTTA----CATATTCCTATATAT--------GAATAGATAATGCAATAC---TGTAAATTT-----AGAAAAAA------------GCTAAAAGAAA-ACAATCGGCTATTCAGTAAAAGCCAGCTTAATTTTTACAGTATACTCGAT--CGATAGATAGA---TTTTTGCATACTTACCTCGTGGCCTTTCCTAAACGACGATGCCTTTCGATGACATTTTCGAGGCCCTCCTCAAAGATGAGATCCAAAGCTGCTCTTAGCCCGTAAAGTAGTTGGATGGAAGGCGTATACGGCCAGTAAGTTCCAAGCTTGTAGAACTTCAAGTAGTCATTCCAGTCGAAGAAAACTCTGACCGACTTGGCATTTTTGGAAGCTTCCAGAGCTTTCGGGCTCGCGCAAACAATGCCCATTCCAGTAGGCAGGGAAA-GTGCTTTCTGCGA

>HEID100442_Tillandsia_landbeckii_subsp_andina

TGCAAGATCATTTTACGCCAATTATGTTACAAGAGGTCATACAATTATAAATGGCAACTAATTACAT-------------------------------------------------------------------------------------------------TCATCTTA----CATATTCCTATATAT--------GAATAGATAATGCAATAC---TGTAAATTT-----AGAAAAAA------------GCTAAAAGAAA-ACAATCGGCTATTCAGTAAAAGCCAGCTTAATTTTTACAGTATACTCGAT--CGATAGATAGA---TTTTTGCATACTTACCTCGTGGCCTTTCCTAAACGACGATGCCTTTCGATGACATTTTCGAGGCCCTCCTCAAAGATGAGATCCAAAGCTGCTCTTAGCCCGTAAAGTAGTTGGATGGAAGGCGTATACGGCCAGTAAGTTCCAAGCTTGTAGAACTTCAAGTAGTCATTCCAGTCGAAGAAAACTCTGACCGACTTGGCATTTTTGGAAGCTTCCAGAGCTTTCGGGCTCGCGCAAACAATGCCCATTCCAGTAGGCAGGGAAA-GTGCTTTCTGCGA

>HEID103591_Tillandsia_marconae_Clone1

TGCAAGATCATTTTATGCCAA---------------TCATACAATTATAAATGCCAACTAATTATAT-------------------------------------------------------------------------------------------------CCATCTTA----CATATTCCTATATAT--------GAATAGATAATGCAATAT---TGTAAATTT-----AGGAAAAAA-----------GCTAAAAGAAA-ACAATCGGCTATTCAGTAAAAG----------------------------------------------TTTGCATACTTACCTCGTGGCCTTTCCTAAACGATGATGCCTTTCGATGACATTTTCGAGCCCCTCCTCAAAGATGAGATCCAAAGCTGCTCTTAGCCCGTAAAGTAGTTGGATGGAAGGCGTATACGGCCAGTAAGTTCCAAGCTTGTAGAACTTCAAGTAGTCATTCCAGTCGAAGAAAACTCTGACCGACTTGGCATTTTTGGAAGCTTCTAGAGCTTTCGGGCTCGCGCAAACAATGCCCATTCCAGTAGGCAGGGAAA-GTGCTTTCTGCGA

>HEID103591_Tillandsia_marconae_Clone2

TGCAAGATCATTTTACGCCAATTATGTTACAAGAGGTCATACAATTATAAATGGCAACTAATTACAT-------------------------------------------------------------------------------------------------TCATCTTA----CATATTCCTATATAT--------GAATAGATAATGCAATAC---TGTAAATTT-----AGAAAAAA------------GCTAAAAGAAA-ACAATCGGCTATTCAGTAAAAGCCAGCTTAATTTTTACAGTATACTCGAT--CGATAGATAGA---TTTTTGCATACTTACCTCGTGGCCTTTCCTAAACGACGATGCCTTTCGATGACATTTTCGAGGCCCTCCTCAAAGATGAGATCCAAAGCTGCTCTTAGCCCGTAAAGTAGTTGGATGGAAGGCGTATACGGCCAGTAAGTTCCAAGCTTGTAGAACTTCAAGTAGTCATTCCAGTCGAAGAAAACTCTGACCGACTTGGCATTTTTGGAAGCTTCCAGAGCTTTCGGGCTCGCGCAAACAATGCCCATTCCAGTAGGCAGGGAAA-GTGCTTTCTGCGA

>HEID103591_Tillandsia_marconae_Clone3

TGCAAGATCATTTTACGCCAATTATGTTACAAGAGGTCATACAATTATAAATGGCAACTAATTACAT-------------------------------------------------------------------------------------------------TCATCTTA----CATATTCCTATATAT--------GAATAGATAATGCAATAC---TGTAAATTT-----AGAAAAAA------------GCTAAAAGAAA-ACAATCGGCTATTCAGTAAAAGCCAGCTTAATTTTTACAGTATACTCGAT--CGATAGATAGA---TTTTTGCATACTTACCTCGTGGCCTTTCCTAAACGACGATGCCTTTCGATGACATTTTCGAGGCCCTCCTCAAAGATGAGATCCAAAGCTGCTCTTAGCCCGTAAAGTAGTTGGATGGAAGGCGTATACGGCCAGTAAGTTCCAAGCTTGTAGAACTTCAAGTAGTCATTCCAGTCGAAGAAAACTCTGACCGACTTGGCATTTTTGGAAGCTTCCAGAGCTTTCGGGCTCGCGCAAACAATGCCCATTCCAGTAGGCAGGGAAA-GTGCTTTCTGCGA

>HEID104553_Tillandsia_incarnata_Clone1

TGCAAGATCATTTTACACCAA-----------------------TTATAAATGGCAACTAATTATAT-------------------------------------------------------------------------------------------------CCATCTTA----CATATTCCTATATAT--------GAATAGATAATGCAATAC---TGTAAATTT-----AGGAAAAA------------GCTAAAAAAAA-ACAATCGGCTATT--------------------------------TCGAT--CGATAGA-AG------TTTGCATACTTACCTCGTGGCCTTTCCTAAACGACGATGCCTTTCGATGACATTTTCGAGGCCCTCCTCAAAGATGAGATCCAAAGCTGCTCTTAGCCCGTAAAGTAGTTGGATGGAAGGCGTATACGGCCAGTAAGTTCCAAGCTTGTAGAACTTCAAGTAGTCATTCCAGTCGAAGAAAACTCTGACCGACTTGGCATTTTTGGAAGCTTCCAGAGCTTTCGGGCTCGCGCAAACAATGCCCATTCCAGTAGGCAGGGAAA-GTGCTTTCTGCGA

>HEID104553_Tillandsia_incarnata_Clone2

TGCAAGATCATTTTACGCCAA---------------TCATACAATTATAAATGCCAACTAATTATAT-------------------------------------------------------------------------------------------------CCATCTTA----CATATTCCTATATATAT------GAATAGATAATGCAATAC---TGTAAATTT-----AGGAAAAA------------GCTAAAAGAAA-ACAATCGGCTATTCAGTAAAAGCCAGC----TTTTTACAGTA--CTCGAT--GGATAGA-AG------TTTGCATACTTACCTCGTGGCCTTTCCTAAACGATGATGCCTTTCGATGACATTTTCGAGCCCCTCCTCAAAGATGAGATCCAAAGCTGCTCTTAGCCCGTAAAGTAGTTGGATGGAAGGCGTATACGGCCAGTAAGTTCCAAGCTTGTAGAACTTCAAGTAGTCATTCCAGTCGAAGAAAACTCTGACCGACTTGGCATTTTTTGAAGCTTCCAGAGCTTTCGGGCTCGCGCAAACAATGCCCATTCCAGTAGGCAGGGAAA-GTGCTTTCTGCGA

>HEID104553_Tillandsia_incarnata_Clone3

TGCAAGATCATTTTACGCCAA-----------------------TTATAAATGGCAACTAATTATAT-------------------------------------------------------------------------------------------------CCATCTTA----CATATTCCTATATAT--------GAATAGATAATGCAATAC---TGTAAATTT-----AGGAAAAA------------GCTAAAAAAAA-ACAATCGGCTATT--------------------------------TCGAT--CGATAGA-AG------TTTGCATACTTACCTCGTGGCCTTTCCTAAACGATGATGCCTTTCGATGACATTTTCGAGCCCCTCCTCAAAGATGAGATCCAAAGCTGCTCTTAGCCCGTAAAGTAGTTGGATGGAAGGCGTATACGGCCAGTAAGTTCCAAGCTTGTAGAACTTCAAGTAGTCATTCCAGTCGAAGAAAACTCTGACCGACTTGGCATTTTTTGAAGCTTCTAGAGCTTTCGGGCTCGCGCAAACAATGCCCATTCCAGTAGGCAGGGAAA-GTGCTTTCTGCGA

>HEID104565_Tillandsia_werdermanii

TGCAAGATCATTTTACGCCAA---------------TCATACAATTATAAATGGCAACTAATTACAT-------------------------------------------------------------------------------------------------CCATCTTA----CATATTCCTATATAT--------GAATAGATAATGCAATAC---TGTAAATTT-----AGAAAACA------------GCTAAAAGAAA-ACAATCGGCTATTCAGTAAAAGCCAGC----TTTTTACAGTATACTCGAT--CGATAGA-AG------TTTGCATACTTACCTCGTGGCCTTTCCTAAACGACGATGCCTTTCGATGACATTTTCGAGGCCCTCCTCAAAGATGAGATCCAAAGCTGCTCTTAGCCCGTAAAGTAGTTGGATGGAAGGCGTATACGGCCAGTAAGTTCCAAGCTTGTAGAACTTCAAGTAGTCATTCCAGTCGAAGAAAACTCTGACCGACTTGGCATTTTTGGAAGCTTGCAGAGCTTTCGGGCTAGCGCAAACAATGCCCATTCCAGTAGGCAGGGAAAAGTGCTTTCTGCGA

>HEID104848_Tillandsia_capillaris_f_virescens

TGCAAGATCATTTTACGCCAATTATGTTACAAGAGGTCATACAATTATAAATGGCAACTAATTACAT-------------------------------------------------------------------------------------------------TCATCTTATATGCATATTCCTATATAT--------GAATAGATAATGCAATAC---TGTAAATTT-----AGAAAAAA------------GCTAAAAGAAA-ACAATCGGCTATTCAGTAAAAGCCAGCTTAATTTTTACAGTATACTCGAT--GGATAGATAGA---TTTTTGCATACTTACCTCGTGGCCTTTCCTAAACGACGATGCCTTTCGATGACATTTTCGAGGCCCTCCTCAAAGATGAGATCCAAAGCTGCTCTTAGCCCGTAAAGTAGTTGGATGGAAGGCGTATACGGCCAGTAAGTTCCAAGCTTGTAGAACTTCAAGTAGTCATTCCAGTCGAAGAAAACTCTGACCGACTTGGCATTTTTGGAAGCTTCCAGAGCTTTCGGGCTCGCGCAAACAATGCCCATTCCAGTAGGCAGGGAAA-GTGCTTTCTGCGA

>HEID104854_Tillandsia_purpurea_Clone1

TGCAAGATCATTTTACGCCAA---------------TCATACAATTATAAATGCCAACTAATTATAT-------------------------------------------------------------------------------------------------CCATCTTA----CATATTCCTATATATAT------GAATAGATAATGCAATAC---TGTAAATTT-----AGGAAAAA------------GCTAAAAGAAA-ACAATCGGCTATTCAGTAAAAGGCAGC----GTTTTACAGTA--CTCGAT--CGATAGA-AG------TTTGCATACTTACCTCGTGGCCTTTCCTAAACGATGATGCCTTTCGATGACATTTTCGAGCCCCTCCTCAAAGATGAGATCCAAAGCTGCTCTTAGCCCGTAAAGTAGTTGGATGGAAGGCGTATACGGCCAGTAAGTTCCGAGCTTGTAGAACTTCAAGTAGTCATTCCAGTCGAAGAAAACTCTGACCGACTTGGCATTTTTGGAAGCTTCTAGAGCTTTCGGGCTCGCGCAAACAATGCCCATTCCAGTAGGCAGGGAAA-GTGCTTTCTGCGA

>HEID104854_Tillandsia_purpurea_Clone2

TGCAAGATCATTTTACGCCAA---------------TCATACAATTATAAATGCCAACTAATTATAT-------------------------------------------------------------------------------------------------CCATCTTA----CATATTCCTATATATAT------GAATAGATAATGCAATAC---TGTAAATTT-----AGGAAAAA------------GCTAAAAGAAA-ACAATCGGCTATTCAGTAAAAGCCAGC----GTTTTACAGTA--CTCGAT--CGATAGA-AG------TTTGCATACTTACCTCGTGGCCTTTCCTAAACGATGATGCCTTTCGATGACATTTTCGAGCCCCTCCTCAAAGATGAGATCCAAAGCTGCTCTTAGCCCGTAAAGTAGTTGGATGGAAGGCGTATACGGCCAGTAAGTTCCGAGCTTGTAGAACTTCAAGTAGTCATTCCAGTCGAAGAAAACTCTGACCGACTTGGCATTTTTGGAAGCTTCTAGAGCTTTCGGGCTCGCGCAAACAATGCCCATTCCAGTAGGCAGGGAAA-GTGCTTTCTGCGA

>HEID104854_Tillandsia_purpurea_Clone3

TGCAAGATCATTTTACGCCAA---------------TCATACAATTATAAATGCCAACTAATTATAT-------------------------------------------------------------------------------------------------CCATCTTA----CATATTCCTATATATAT------GAATAGATAATGCAATAC---TGTAAATTT-----AGGAAAAA------------GCTAAAAGAAA-ACAATCGGCTATTCAGTAAAAGCCAGC----GTTTTACAGTA--CTCGAT--CGATAGA-AG------TTTGCATACTTACCTCGTGGCCTTTCCTAAACGATGATGCCTTTCGATGACATTTTCGAGCCCCTCCTCAAAGATGAGATCCAAAGCTGCTCTTAGCCCGTAAAGTAGTTGGATGGAAGGCGTATACGGCCAGTAAGTTCCGAGCTTGTAGAACTTCAAGTAGTCATTCCAGTCGAAGAAAACTCTGACCGACTTGGCATTTTTGGAAGCTTCTAGAGCTTTCGGGCTCGCGCAAACAATGCCCATTCCAGTAGGCAGGGAAA-GTGCTTTCTGCGA

>HEID104860_Tillandsia_yuncharaensis_Clone1

TGCAAGATCATTTTACGCCAA---------------TCATACAATTATAAATGGCAACTAATTACAT-------------------------------------------------------------------------------------------------CCATCTTA----CATATTCCTATATAT--------GAATAGATAATGCAATAC---TGTAAATTT-----AGAAAACA------------GCTAAAAGAAA-ACAATCGGCTATTCAGTAAAAGCCAGCTTAATTTTTACAGTATACTCGAT--CGATAGA-AG------TTTGCATACTTACCTCGTGGCCTTTCCTAAACGACGATGCCTTTCGATGACATTTTCGAGCCCCTCCTCAAAGATGAGATCCAAAGCTGCTCTTAGCCCGTAAAGTAGTTGGATGGAAGGTGTATACGGCCAGTAAGTTCCAAGCTTGTAGAACTTCAAGTAGTCATTCCAGTCGAAGAAAACTCTGACCGACTTGGCATTTTTGGAAGCTTCCAGAGCTTTCGGGCTTGCGCAAACAATGCCCATTCCAGTAGGCAGGGAAA-GTGCTTTCTGCGA

>HEID104860_Tillandsia_yuncharaensis_Clone2

TGCAAGATCATTTTACGCCAA---------------TCATACAATTATAAATGGCAACTAATTACAT-------------------------------------------------------------------------------------------------CCATCTTA----CATATTCCTATATAT--------GAATAGATAATGCAATAC---TGTAAATTT-----AGAAAAAA------------GCTAAAAGAAA-ACAATCGGCTATTCAGTAAAAGCCAGCTTAATTTTTACAGTATACTCGAT--CGATAGA-AG------TTTGCATACTTACCTCGTGGCCTTTCCTAAACGACGATGCCTTTCGATGACATTTTCGAGCCCCTCCTCAAAGATGAGATCCAAAGCTGCTCTTAGCCCGTAAAGTAGTTGGATGGAAGGCGTATACGGCCAGTAAGTTCCAAGCTTGTAGAACTTCAAGTAGTCATTCCAGTCGAAGAAAACTCTGACCGACTTGGCATTTTTGGAAGCTTCCAGAGCTTTCGGGCTCGCGCAAACAATGCCCATTCCAGTAGGCAGGGAAA-GTGCTTTCTGCGA

>HEID104860_Tillandsia_yuncharaensis_Clone3

TGCAAGATCATTTTACGCCAA---------------TCATACAATTATAAATGGCAACTAATTACAT-------------------------------------------------------------------------------------------------CCATCTTA----CATATTCCTATATAT--------GAATAGATAATGCAATAC---TGTAAATTT-----AGAAAAAA------------GCTAAAAGAAA-ACAATCGGCTATTCAGTAAAAGCCAGCTTAATTTTTACAGTATACTCGAT--CGATAGA-AG------TTTGCATACTTACCTCGTGGCCTTTCCTAAACGACGATGCCTTTCGATGACATTTTCGAGCCCCTCCTCAAAGATGAGATCCAAAGCTGCTCTTAGCCCGTAAAGTAGTTGGATGGAAGGCGTATACGGCCAGTAAGTTCCAAGCTTGTAGAACTTCAAGTAGTCATTCCAGTCGAAGAAAACTCTGACCGACTTGGCATTTTTGGAAGCTTCCAGAGCTTTCGGGCTCGCGCAAACAATGCCCATTCCAGTAGGCAGGGAAA-GTGCTTTCTGCGA

>HEID105087_Tillandsia_recurvata_Clone1

TGCAAGATCATTTTACGCCAA---------------TCATACAATTATAAATGGCAACTAATTATAT-------------------------------------------------------------------------------------------------TCATCTTA----CATATTCCTATA----------------------------C---TGTAAATTT-----AAAAAAAAAAAAAAAA-----TTAAAAGAAA-ATAATCGTCTATTCAGTAAAAGCCAGCTTAATTTTTACAGTATACTCGAT--CGATAGA-------TTTTTGCATACTTACCTCGTGGCCTTTCCTAAACGACGATGCCTTTCGATGACATTTTCGAGCCCCTCCTCAAAGATGAGATCCAAAGCTGCTCTTAGCCCGTAAAGTAGTTGGATGGAAGGCGTATACGGCCAGTAAGTTCCAAGCTTGTAGAACTTCAAATAGTCATTCCAGTCGAAGAAAACTCTGACCGACTTGGCATTTTTGGAAGCTTCCAGAGCTTTCGGGCTCGCGCAAATAATGCCCATTCCAGTAGGCAGGGAAA-GTGCTTTCTGCGA

>HEID105087_Tillandsia_recurvata_Clone2

TGCAAGATCATTTTATGCCAA---------------TCATACAATTATAAATGGCAACTAATTATAT-------------------------------------------------------------------------------------------------TCATCTTA----CATATTCCTATA----------------------------C---TGTAAATTT-----AAAAAAAAAAAAAAAAAAA--TTAAAAGAAA-ATAATCGTCTATTCAGTAAAAGCCAGCTTAATTTTTACAGTATACTCGAT--CGATAGA-------TTTTTGCATACTTACCTCGTGGCCTTTCCTAAACGACGATGCCTTTCGATGACATTTTCGAGCCCCTCCTCAAAGATGAGATCCAAAGCTGCTCTTAGCCCGTAAAGTAGTTGGATGGAAGGCGTATACGGCCAGTAAGTTCCAAGCTTGTAGAACTTCAAATAGTCATTCCAGTCGAAGAAAACTCTGACCGACTTGGCATTTTTGGAAGCTTCCAGAGCTTTCGGGCTCGCGCAAATAATGCCCATTCCAGTAGGCAGGGAAA-GTGCTTTCTGCGA

>HEID105087_Tillandsia_recurvata_Clone3

TGCAAGATCATTTTACGCCAA---------------TCATACAATTATAAATGGCAACTAATTATAT-------------------------------------------------------------------------------------------------TCATCTTA----CATATTCCTATA----------------------------C---TGTAAATTT-----AAAAAAAAAAAAAAAAA----TTAAAAGAAA-ATAATCGTCTATTCAGTAAAAGCCAGCTTAATTTTTACAGTATACTCGAT--CGATAGA-------TTTTTGCATACTTACCTCGTGGCCTTTCCTAAACGACGATGCCTTTCGATGACATTTTCGAGCCCCTCCTCAAAGATGAGATCCAAAGCTGCTCTTAGCCCGTAAAGTAGTTGGATGGAAGGCGTATACGGCCAGTAAGTTCCAAGCTTGTAGAACTTCAAATAGTCATTCCAGTCGAAGAAAACTCTGACCGACTTGGCATTTTTGGAAGCTTCCAGAGCTTTCGGGCTCGCGCAAATAATGCCCATTCCAGTAGGCAGGGAAA-GTGCTTTCTGCGA

>HEID105263_Tillandsia_kuehhasii

TGCAAGATCATTTTACGCCAATTATGTTACAAGAGGTCATACAATTATAAATGGCAACNAATTACAT-------------------------------------------------------------------------------------------------TCATCTTA----CATATTCCTATATAT--------GAATAGATAATNCAATNC---TGT-----------------------------------------------------------------------------------------------GGATAGA-------TTTTTGCATACTTACCTCGTGGCCTTTCCTAAACGACGATGCCTTTCGATGACATTTTCGAGGCCCTCCTCAAAGATGAGATCCAAAGCTGCTCTTAGCCCGTAAAGTAGTTGGATGGAAGGCGTATACGGCCAGTAAGTTCCAAGCTTGTAGAACTTCAAGTAGTCGTTCCAGTCGAAGAAAACTCTGACCGACTTGGCATTTTTGGAAGCTTCCAGAGCTTTCGGGCTCGCGCAAACAATGCCCATTCCAGTAGGCAGGGAAA-NTGCTTTCTGCGA

>HEID108216_Tillandsia_geissei

TGCAAGATCATTTTACGCCAA---------------TCATACAATTATAAATGGCAACTAATCATAT-------------------------------------------------------------------------------------------------CCATCTTA----CATATTCCTATATAT--------GAATAGATAATGCAATAC---TGTAAATTT-----AGAAAAAA------------GCTAAAAGAAA-ACAATCGGCTATTCAGTAAAAGCCAGC----TTTTTACAGTA--CTCGAT--CGATAGA-AG------TTTGCATACTTACCTCGTGGCCTTTCCTAAACGACGATGCCTTTCGATGACATTTTCGAGCCCCTCCTCAAAGATGAGATCCAAAGCTGCTCTTAGCCCGTAAAGTAGTTGGATGGAAGGCGTATACGGCCAGTAAGTTCCAAGCTTGTAGAACTTCAAGTAGTCATTCCAGTCGAAGAAAACTCTGACCGACTTGGCATTTTTGGAAGCTTCTAGAGCTTTCGGGCTCGCGCAAACAATGCCCATTCCAGTAGGCAGGGAAA-GTGCTTTCTGCGA

>HEID112451_Tillandsia_landbeckii

TGCAAGATCATTTTACGCCAATTATGTTACAAGAGGTCATACAATTATAAATGGCAACTAATTACAT-------------------------------------------------------------------------------------------------TCATCTTA----CATATTCCTATATAT--------GAATAGATAATGCAATAC---TGTAAATTT-----AGAAAAAA------------GCTAAAAGAAA-ACAATCGGCTATTCAGTAAAAGCCAGCTTAATTTTTACAGTATACTCGAT--CGATAGATAGA---TTTTTGCATACTTACCTCGTGGCCTTTCCTAAACGACGATGCCTTTCGATGACATTTTCGAGGCCCTCCTCAAAGATGAGATCCAAAGCTGCTCTTAGCCCGTAAAGTAGTTGGATGGAAGGCGTATACGGCCAGTAAGTTCCAAGCTTGTAGAACTTCAAGTAGTCATTCCAGTCGAAGAAAACTCTGACCGACTTGGCATTTTTGGAAGCTTCCAGAGCTTTCGGGCTCGCGCAAACAATGCCCATTCCAGTAGGCAGGGAAA-GTGCTTTCTGCGA

>HEID112534_Tillandsia_landbeckii

TGCAAGATCATTTTACGCCAATTATGTTACAAGAGGTCATACAATTATAAATGGCAACTAATTACAT-------------------------------------------------------------------------------------------------TCATCTTA----CATATTCCTATATAT--------GAATAGATAATGCAATAC---TGTAAATTT-----AGAAAAAA------------GCTAAAAGAAA-ACAATCGGCTATTCAGTAAAAGCCAGCTTAATTTTTACAGTATACTCGAT--CGATAGATAGA---TTTTTGCATACTTACCTCGTGGCCTTTCCTAAACGACGATGCCTTTCGATGACATTTTCGAGGCCCTCCTCAAAGATGAGATCCAAAGCTGCTCTTAGCCCGTAAAGTAGTTGGATGGAAGGCGTATACGGCCAGTAAGTTCCAAGCTTGTAGAACTTCAAGTAGTCATTCCAGTCGAAGAAAACTCTGACCGACTTGGCATTTTTGGAAGCTTCCAGAGCTTTCGGGCTCGCGCAAACAATGCCCATTCCAGTAGGCAGGGAAA-GTGCTTTCTGCGA

>HEID113094_Tillandsia_landbeckii_var_rigidor

TGCAAGATCATTTTACGCCAATTATGTTACAAGAGGTCATACAATTATAAATGGCAACTAATTACAT-------------------------------------------------------------------------------------------------TCATCTTA----CATATTCCTATATAT--------GAATAGATAATGCAATAC---TGTAAATTT-----AGAAAAAA------------GCTAAAAGAAA-ACAATCGGCTATTCAGTAAAAGCCAGCTTAATTTTTACAGTATACTCGAT--CGATAGATAGA---TTTTTGCATACTTACCTCGTGGCCTTTCCTAAACGACGATGCCTTTCGATGACATTTTCGAGGCCCTCCTCAAAGATGAGATCCAAAGCTGCTCTTAGCCCGTAAAGTAGTTGGATGGAAGGCGTATACGGCCAGTAAGTTCCAAGCTTGTAGAACTTCAAGTAGTCATTCCAGTCGAAGAAAACTCTGACCGACTTGGCATTTTTGGAAGCTTCCAGAGCTTTCGGGCTCGCGCAAACAATGCCCATTCCAGTAGGCAGGGAAA-GTGCTTTCTGCGA

>HEID113095_Tillandsia_landbeckii_subsp_andina_W_Till

TGCA---TCATTTTACGCCAATTATGTTACAAGAGGTCATACAATTATAAATGGCAACTAATTACAT-------------------------------------------------------------------------------------------------TCATCTTA----CATATTCCTATATAT--------GAATAGATAATGCAATAC---TGTAAATTT-----AGAAAAAA------------GCTAAAAGAAA-ACAATCGGCTATTCAGTAAAAGCCAGCTTAATTTTTACAGTATACTCGAT--CGATAGATAGA---TTTTTGCATACTTACCTCGTGGCCTTTCCTAAACGACGATGCCTTTCGATGACATTTTCGAGGCCCTCCTCAAAGATGAGATCCAAAGCTGCTCTTAGCCCGTAAAGTAGTTGGATGGAAGGCGTATACGGCCAGTAAGTTCCAAGCTTGTAGAACTTCAAGTAGTCATTCCAGTCGAAGAAAACTCTGACCGACTTGGCATTTTTGGAAGCTTCCAGAGCTTTCGGGCTCGCGCAAACAATGCCCATTCCAGTAGGCAGGGAAA-GTGCTTTCTGCGA

>HEID113096_Tillandsia_landbeckii_subsp_andina

TGCA---TCATTTTACGCCAATTATGTTACAAGAGGTCATACAATTATAAATGGCAACTAATTACAT-------------------------------------------------------------------------------------------------TCATCTTA----CATATTCCTATATAT--------GAATAGATAATGCAATAC---TGTAAATTT-----AGAAAAAA------------GCTAAAAGAAA-ACAATCGGCTATTCAGTAAAAGCCAGCTTAATTTTTACAGTATACTCGAT--CGATAGATAGA---TTTTTGCATACTTACCTCGTGGCCTTTCCTAAACGACGATGCCTTTCGATGACATTTTCGAGGCCCTCCTCAAAGATGAGATCCAAAGCTGCTCTTAGCCCGTAAAGTAGTTGGATGGAAGGCGTATACGGCCAGTAAGTTCCAAGCTTGTAGAACTTCAAGTAGTCATTCCAGTCGAAGAAAACTCTGACCGACTTGGCATTTTTGGAAGCTTCCAGAGCTTTCGGGCTCGCGCAAACAATGCCCATTCCAGTAGGCAGGGAAA-GTGCTTTCTGCGA

>HEID113097_Tillandsia_landbeckii_subsp_andina

TGCA---TCATTTTACGCCAATTATGTTACAAGAGGTCATACAATTATAAATGGCAACTAATTACAT-------------------------------------------------------------------------------------------------TCATCTTA----CATATTCCTATATAT--------GAATAGATAATGCAATAC---TGTAAATTT-----AGAAAAAA------------GCTAAAAGAAA-ACAATCGGCTATTCAGTAAAAGCCAGCTTAATTTTTACAGTATACTCGAT--CGATAGATAGA---TTTTTGCATACTTACCTCGTGGCCTTTCCTAAACGACGATGCCTTTCGATGACATTTTCGAGGCCCTCCTCAAAGATGAGATCCAAAGCTGCTCTTAGCCCGTAAAGTAGTTGGATGGAAGGCGTATACGGCCAGTAAGTTCCAAGCTTGTAGAACTTCAAGTAGTCATTCCAGTCGAAGAAAACTCTGACCGACTTGGCATTTTTGGAAGCTTCCAGAGCTTTCGGGCTCGCGCAAACAATGCCCATTCCAGTAGGCAGGGAAA-GTGCTTTCTGCGA

>HEID113098_Tillandsia_landbeckii_subsp_andina

TGCA---TCATTTTACGCCAATTATGTTACAAGAGGTCATACAATTATAAATGGCAACTAATTACAT-------------------------------------------------------------------------------------------------TCATCTTA----CATATTCCTATATAT--------GAATAGATAATGCAATAC---TGTAAATTT-----AGAAAAAA------------GCTAAAAGAAA-ACAATCGGCTATTCAGTAAAAGCCAGCTTAATTTTTACAGTATACTCGAT--CGATAGATAGA---TTTTTGCATACTTACCTCGTGGCCTTTCCTAAACGACGATGCCTTTCGATGACATTTTCGAGGCCCTCCTCAAAGATGAGATCCAAAGCTGCTCTTAGCCCGTAAAGTAGTTGGATGGAAGGCGTATACGGCCAGTAAGTTCCAAGCTTGTAGAACTTCAAGTAGTCATTCCAGTCGAAGAAAACTCTGACCGACTTGGCATTTTTGGAAGCTTCCAGAGCTTTCGGGCTCGCGCAAACAATGCCCATTCCAGTAGGCAGGGAAA-GTGCTTTCTGCGA

>HEID113099_Tillandsia_landbeckii_subsp_andina_Clone1

TGCAAGATCATTTTACGCCAA---------------TCATACAATTATAAATGGCAACTAATTACAT-------------------------------------------------------------------------------------------------TCATCTTA----CATATTCCTATATAT--------GAATAGATAATGCAATAC---TGTAAATTT-----AGAAAAAA------------GCTAAAAGAAA-ACAATTGGCTATTCAGTAAAAGCCAGCTTAATTTTTACAGTATACTCGAT--CGATAGATAGA---TTTTTGCATACTTACCTCGTGGCCTTTCCTAAACGACGATGCCTTTCGATGACATTTTCGAGGCCCTCCTCAAAGATGAGATCCAAAGCTGCTCTTAGCCCGTAAAGTAGTTGGATGGAAGGCGTATACGGCCAGTAAGTTCCAAGCTTGTAGAACTTCAAGTAGTCATTCCAGTCGAAGAAAACTCGGACCGACTTGGCATTTTTGGAAGCTTCCAGAGCTTTCGGGCTCGCGCAAACAATGCCCATTCCAGTAGGCAGGGAAA-GTGCTTTCTGCGA

>HEID113099_Tillandsia_landbeckii_subsp_andina_Clone3

TGCAAGATCATTTTACGCCAA---------------TCATACAATTATAAATGGCAACTAATTACAT-------------------------------------------------------------------------------------------------TCATCTTATATGCATATTCCTATATAT--------GAATAGATAATGCAATAC---TGTAAATTT-----AGAAAAAAA------------CTAAAAGAAA-ACAATTGGCTATTCAGTAAAAGCCAGCTTAATTTTTACAGTATACTCGAT--CGATAGATAGA---TTTTTGCATACTTACCTCGTGGCCTTTCCTAAACGACGATGCCTTTCGATGACATTTTCGAGGCCCTCCTCAAAGATGAGATCCAAAGCTGCTCTTAGCCCGTAAAGTAGTTGGATGGAAGGCGTATACGGCCAGTAAGTTCCGAGCTTGTAGAACTTCAAGTAGTCATTCCAGTCGAAGAAAACTCTGACCGACTTGGCATTTTTGGAAGCTTCCAGAGCTTTCGGGCTCGCGCAAACAATGCCCATTCCAGTAGGCAGGGAAA-GTGCTTTCTGCGA

>HEID131337_Tillandsia_cacticola

TGCAAGATCATTTTACGCCAA---------------TCATACAATTATAAATGCCAACTAATTATAT-------------------------------------------------------------------------------------------------CCATCTTA----CATATTCCTATATAT--------GAATAGATAATGCAATACTACTGTAAATTT-----AGAAAAAA------------GCTAAAAGAAA-ACAATCGGCTATTCAGTAAAAA----------------------------------------------TTTGCATACTTACCTCGTGGCCTTTCCTAAACGATGATGCCTTTCGATGACATTTTCGAGCCCCTCCTCAAAGATGAGATCCAAAGCTGCTCTAAGCCCATAAAGTAGTTGGATGGAAGGCGTATACGGCCAGTAAGTTCCAAGCTTGTAGAACTTCAAGTAGTCATTCCAGTCGAAGAAAACTCTGACCGACTTGGCATTTTTGGAAGCTTCTAGAGCTTTCGGGCTCGCGCAAACAATGCCCATTCCAGTAGGCAGGGAAAAGTGCTTTCTGCGA

>HEID131338_Tillandsia_purpurea_Clone1

TGCAAGATCATTTTACGCCAA---------------TCATACAATTATAAATGCCAACTAATTATAT-------------------------------------------------------------------------------------------------CCATCTTA----CATATTCCTATATATAT------GAATAGATAATGCAATAC---TGTAAATTT-----AGGAGAAA------------GCTAAAAGAAA-ACAATCGGCTATTCAGTAAAAGCCAGC----TTTTTACAGTA--CTCGAT--AGATAGA-AG------TTTGCATACTTACCTCGTGGCTTTTCCTAAACGATGATGCCTTTCAATGACATTTTCGAGCCCCTCCTCAAAGATGAGATCCAAAGCTGCTCTTAGCCCGTAAAGTAGTTGGATGGAAGGCGTATACGGCCAGTAAGTTCCGAGCTTGTAGAACTTCAAGTAGTCATTCCAGTCGAAGAAAACTCTGACCGACTTGGCATTTTTTGAAGCTTCTAGAGCTTTCGGGCTCGCGCAAACAATGCCCATTCCAGTAGGCAGGGAAA-GTGCTTTCTGCGA

>HEID131338_Tillandsia_purpurea_Clone2

TGCAAGATCATTTTACGCCAA---------------TCATACAATTATAAATGCCAACTAATTATAT-------------------------------------------------------------------------------------------------CCATCTTA----CATATTCCTATATATAT------GAATAGATAATGCAATAC---TGTAAATTT-----AGGAGAAA------------GCTAAAAGAAA-ACAATCGGCTATTCAGTAAAAGCCAGC----TTTTTACAGTA--CTCGAT--AGATAGA-AG------TTTGCATACTTACCTCGTGGCTTTTCCTAAACGATGATGCCTTTCAATGACATTTTCGAGCCCCTCCTCAAAGATGAGATCCAAAGCTGCTCTTAGCCCGTAAAGTAGTTGGATGGAAGGCGTATACGGCCAGTAAGTTCCAAGCTTGTAGAACTTCAAGTAGTCATTCCAGTCGAAGAAAACTCTGACCGACTTGGCATTTTTTGAAGCTTCTAGAGCTTTCGGGCTCGCGCAAACAATGCCCATTCCAGTAGGCAGGGAAA-GTGCTTTCTGCGA

>HEID131338_Tillandsia_purpurea_Clone3

TGCAAGATCATTTTACGCCAA---------------TCATACAATTATAAATGCCAACTAATTATAT-------------------------------------------------------------------------------------------------CCATCTTA----CATATTCCTATATATAT------GAATAGATAATGCAATAC---TGTAAATTT-----AGGAGAAA------------GCTAAAAGAAA-ACAATCGGCTATTCAGTAAAAGCCAGC----TTTTTACAGTA--CTCGAT--AGATAGA-AG------TTTGCATACTTACCTCGTGGCTTTTCCTAAACGATGATGCCTTTCAATGACATTTTCGAGCCCCTCCTCAAAGATGAGATCCAAAGCTGCTCTTAGCCCGTAAAGTAGTTGGATGGAAGGCGTATACGGCCAGTAAGTTCCAAGCTTGTAGAACTTCAAGTAGTCATTCCAGTCGAAGAAAACTCTGACCGACTTGGCATTTTTTGAAGCTTCTAGAGCTTTCGGGCTCGCGCAAACAATGCCCATTCCAGTAGGCAGGGAAA-GTGCTTTCTGCGA

>HEID131403_Tillandsia_zecheri_Clone2

TGCAAGATCATTTTACGTCAA---------------TCATACAATTATAAATGACAACTAATTATAT-------------------------------------------------------------------------------------------------TCATCTTA----CATATTTCTATA----------------------------C---TGTAA-TTTTTTTTAAAAAAAAAA-----------CTAAAAGAAA-ATAATCGTCTATTCAGTAAAAGCCAGCTTAATTTTTACAGTATACTCGAT--GGATAGA-------TTTTTGCATACTTACCTCGTGGCCTTTCCTAAACGACGATGCCTTTCGATGACATTTTCGAGCCCCTCCTCAAAGATGAGATCCAAAGCTGCTCTTAGCCCGTAAAGTAGTTGGATGGAAGGCGTATACGGCCAGTAAGTTCCAAGCTTGTAGAACTTCAAATAGTCATTCCAGTCGAAGAAAACTCTGACCGACTTGGCATTTTTGGAAGCTTCCAGAGCTTTCGGGCTCGCGCAAACAATGCCCATTCCAGTAGGCAGGGAAA-GTGCTTTCTGCGA

>HEID131403_Tillandsia_zecheri_Clone3

TGCAAGATCATTTTACGCCAA---------------TCATACAATTATAAATGGCAACTAATTACAT-------------------------------------------------------------------------------------------------CCATCTTA----CATATTCCTATATAT--------GAATAGATAATGCAATAC---TGTAAATTT-----AGAAAAAA------------GCTAAAAGAAA-ACAATCGGCTATTCAGTAAAAGCCAGCTTAATTTTTACAGTATACTCGAT--CGATAGA------TTTTTTGCATACTTACCTCGTGGCCTTTCCTAAACGACGATGCCTTTCGATGACATTTTCGAGCCCCTCCTCAAAGATGAGATCCAAAGCTGCTCTTAGCCCGTAAAGTAGTTGGATGGAAGGTGTATACGGCCAGTAAGTTCCAAGCTTGTAGAACTTCAAGTAGTCATTCCAGTCGAAGAAAACTCTGACCGACTTGGCATTTTTGGAAGCTTCCAGAGCTTTCGGGCTCGCGCAAACAATGCCCATTCCAGTAGGCAGGGAAA-GTGCTTTCTGCGA

>HEID131403_Tillandsia_zecheri_Clone4

TGCAAGATCATTTTACGCCAATTATGTTACAAGAGGTCATACAATTATAAATGGCAACTAATTACAT-------------------------------------------------------------------------------------------------CC-TCTTA----CATATTCC------------------TAGATAATGCAATAC---TGTAAATTT-----AGAAAAAA------------GCTAAAAGAAA-ACAATCGGCTATTCAGTAAAAGCCAGCTTAATTTTTACAGTATACTCGAT--CGATAGA-------TTTTTGCATACTTACCTCGTGGCCTTTCCTAAACGACGATGCCTTTCGATGACATTTTCGAGGCCCTCCTCAAAGATGAGATCCAAAGCTGCTCTTAGCCCGTAAAGTAGTTGGATGGAAGGCGTATACGGCCAGTAAGTTCCAAGCTTGTAGAACTTCAAGTAGTCATTCCAGTCGAAGAAAACTCTGACCGACTTGGCATTTTTGGAAGCTTCCAGAGCTTTCGGGCTCGCGCAAACAATGCCCATTCCAGTAGGCAGGGAAA-GTGCTTTCTGCGA

>HEID131405_Tillandsia_marconae_Clone1

TGCAAGATCATTTTATGCCAA---------------TCATACAATTATAAATGCCAACTAATTATAT-------------------------------------------------------------------------------------------------CCATCTTA----CATATTCCTATATAT--------GAATAGATAATGCAATAT---TGTAAATTT-----AGGAAAAAA-----------GCTAAAAGAAA-ACAATCGGCTATTCAGTAAAAG----------------------------------------------TTTGCATACTTACCTCGTGGCCTTTCCTAAACGATGATGCCTTTCGATGACATTTTCGAGCCCCTCCTCAAAGATGAGATCCAAAGCTGCTCTTAGCCCGTAAAGTAGTTGGATGGAAGGCGTATACGGCCAGTAAGTTCCAAGCTTGTAGAACTTCAAGTAGTCATTCCAGTCGAAGAAAACTCTGACCGACTTGGCATTTTTGGAAGCTTCTAGAGCTTTCGGGCTCGCGCAAACAATGCCCATTCCAGTAGGCAGGGAAA-GTGCTTTCTGCGA

>HEID131405_Tillandsia_marconae_Clone2

TGCAAGATCATTTTACGCCAATTATGTTACAAGAGGTCATACAATTATAAATGGCAACTAATTACAT-------------------------------------------------------------------------------------------------TCATCTTA----CATATTCCTATATAT--------GAATAGATAATGCAATAC---TGTAAATTT-----AGAAAAAA------------GCTAAAAGAAA-ACAATCGGCTATTCAGTAAAAGCCAGCTTAATTTTTACAGTATACTCGAT--CGATAGATAGA---TTTTTGCATACTTACCTCGTGGCCTTTCCTAAACGACGATGCCTTTCGATGACATTTTCGAGGCCCTCCTCAAAGATGAGATCCAAAGCTGCTCTTAGCCCGTAAAGTAGTTGGATGGAAGGCGTATACGGCCAGTAAGTTCCAAGCTTGTAGAACTTCAAGTAGTCATTCCAGTCGAAGAAAACTCTGACCGACTTGGCATTTTTGGAAGCTTCCAGAGCTTTCGGGCTCGCGCAAACAATGCCCATTCCAGTAGGCAGGGAAA-GTGCTTTCTGCGA

>HEID131405_Tillandsia_marconae_Clone3

TGCAAGATCATTTTACGCCAATTATGTTACAAGAGGTCATACAATTATAAATGGCAACTAATTACAT-------------------------------------------------------------------------------------------------TCATCTTA----CATATTCCTATATAT--------GAATAGATAATGCAATAC---TGTAAATTT-----AGAAAAAA------------GCTAAAAGAAA-ACAATCGGCTATTCAGTAAAAGCCAGCTTAATTTTTACAGTATACTCGAT--CGATAGATAGA---TTTTTGCATACTTACCTCGTGGCCTTTCCTAAACGACGATGCCTTTCGATGACATTTTCGAGGCCCTCCTCAAAGATGAGATCCAAAGCTGCTCTTAGCCCGTAAAGTAGTTGGATGGAAGGCGTATACGGCCAGTAAGTTCCAAGCTTGTAGAACTTCAAGTAGTCATTCCAGTCGAAGAAAACTCTGACCGACTTGGCATTTTTGGAAGCTTCCAGAGCTTTCGGGCTCGCGCAAACAATGCCCATTCCAGTAGGCAGGGAAA-GTGCTTTCTGTGA

>HEID131417_Tillandsia_humillis_Clone1

TGCAAGATCATTTTACGCCAATTATGTTACAAGAGGTCATACAATTATAAATGGCAACTAATTACAT-------------------------------------------------------------------------------------------------CC-TCTTA----CATATTCC------------------TAGATAATGCAATAC---TGTAAATTT-----AGAAAAAA------------GCTAAAAGAAA-ACAATCGGCTATTCAGTAAAAGCCAGCTTAATTTTCACAGTATACTCGAT--CGATAGA-------TTTTTGCATACTTACCTCGTGGCCTTTCCTAAACGACGATGCCTTTCGATGACATTTTCGAGGCCCTCCTCAAAGATGAGATCCAAAGCTGCTCTTAGCCCGTAAAGTAGTTGGATGGAAGGCGTATACGGCCAGTAAGTTCCAAGCTTGTAGAACTTCAAGTAGTCATTCCAGTCGAAGAAAACTCTGACCGACTTGGCATTTTTGGAAGCTTCCAGAGCTTTCGGGCTCGCGCAAACAATGCCCATTCCAGTAGGCAGGGAAA-GTGCTTTCTTCGA

>HEID131417_Tillandsia_humillis_Clone2

TGCAAGATCATTTTACGCCAA---------------TCATACAATTATAAATGCCAACTAATTATATCCAGAGCCTTGTTGTTCTTTTGATGTAGCTGAGTTCATTTTTCATTGAATGAAGCGGCAACATGCTGCCTTTCTCTCGAAAAAAAAACTAATTATATCCATCTTA----CATATTCCTATATAT--------GAATAGATAATGTAATAC---TGTAAATTT-----AGGAAATAA-----------GCTAAAAGAAA-ACAATCGGCTATTCAGTAAAAG----------------------------------------------TTTGCATACTTACCTCGTGGCCTTTCCTAAACGATGATGCCTTTCGATGACATTTTCGAGCCCCTCCTCAAAGATGAGATCCAAAGCTGCTCTTAGCCCGTAAAGTAGTTGGATGGAAGGCGTATACGGCCAGTAAGTTCCAAGCTTGTAGAACTTCAAGTAGTCATTCCAGTCGAAGAAAACTCTGACCGACTTGGCATTTTTGGAAGCTTCTAGAGCTTTCGGGCTCGCGCAAACAATGCCCATTCCAGTAGGCAGGGAAA-GTGCTTTCTGCGA

>HEID131417_Tillandsia_humillis_Clone3

TGCAAGATCATTTTACGCCAATTATGTTGCAAGAGGTCATACAATTATAAATGGCAACTAATTACAT-------------------------------------------------------------------------------------------------CC-TCTTA----CATATTCC------------------TAGATAATGCAATAC---TGTAAATTT-----AGAAAAAA------------GCTAAAAGAAA-ACAATCGGCTATTCAGTAAAAGCCAGCTTAATTTTTACAGTATACTCGAT--CGATAGA-------TTTTTGCATACTTACCTCGTGGCCTTTCCTAAACGACGATGCCTTTCGATGACATTTTCGAGGCCCTCCTCAAAGATGAGATCCAAAGCTGCTCTTAGCCCGTAAAGTAGTTGGATGGAAGGCGTATACGGCCAGTAAGTTCCAAGCTTGTAGAACTTCAAGTAGTCATTCCAGTCGAAGAAAACTCTGACCGACTTGGCATTTTTGGAAGCTTCCAGAGCTTTCGGGCTCGCGCAAACAATGCCCATTCCAGTAGGCAGGGAAA-GTGCTTTCTGCGA

>HEID131590_Tillandsia_recurvata_Clone1

TGCAAGATCATTTTACGCCAA---------------TCATACAATTATAAATGGCAACTAATTATAT-------------------------------------------------------------------------------------------------TCATCTTA----CATATTCCTATA----------------------------C---TGTAAATTT-----AAAAAAAAAAAAAAAA-----CTAAAAGAAA-ATAATCGTCTATTCAGTAAAAGCCAGCTTAATTTTTACAGTATACTCGAT--CGATAGA-------TTTTTGCATACTTACCTCGTGGCCTTTCCTAAACGACGATGCCTTTCGATGACATTTTCGAGGCCCTCCTCAAAGATGAGATCCAAAGCTGCTCTTAGCCCGTAAAGTAGTTGGATGGAAGGCGTATACGGCCAGTAAGTTCCAAGATTGTAGAACTTCAAGTAGTCATTCCAGTCGAAGAAAACTCTGACCGACTTGGCATTTTTGGAAGCTTCCAGAGCTTTCGGGCTCGCGCAAACAATTCCCATTCCAGTAGGCAGGGAAA-GTGCTTTCTGCGA

>HEID131590_Tillandsia_recurvata_Clone2

TGCAAGATCATTTTACGCCAA---------------TCATACAATTATAAATGGCAACTAATTATAT-------------------------------------------------------------------------------------------------TCATCTTA----CATATTCCTATA----------------------------C---TGTAAATTT-----AAAAAAAAAAAAAAAAAA---CTAAAAGAAA-ATAATCGTCTATTCAGTAAAAGCCAGCTTAATTTTTACAGTATACTCGAT--CGATAGA-------TTTTTGCATACTTACCTCGTGGCCTTTCCTAAACGACGATGCCTTTCGATGACATTTTCGAGGCCCTCCTCAAAGATGAGATCCAAAGCTGCTCTTAGCCCGTAAAGTAGTTGGATGGAAGGCGTATACGGCCAGTAAGTTCCAAGATTGTAGAACTTCAAGTAGTCATTCCAGTCGAAGAAAACTCTGACCGACTTGGCATTTTTGGAAGCTTCCAGAGCTTTCGGGCTCGCGCAAACAATTCCCATTCCAGTAGGCAGGGAAA-GTGCTTTCTGCGA

>HEID131590_Tillandsia_recurvata_Clone3

TGCAAGATCATTTTACGCCAA---------------TCATACAATTATAAATGGCAACTAATTATAT-------------------------------------------------------------------------------------------------TCATCTTA----CATATTCCTATA----------------------------C---TGTAAATTT-----AAAAAAAAAAAAAAAAA----CTAAAAGAAA-ATAATCGTCTATTCAGTAAAAGCCAGCTTAATTTTTACAGTATACTCGAT--CGATAGA-------TTTTTGCATACTTACCTCGTGGCCTTTCCTAAACGACGATGCCTTTCGATGACATTTTCGAGGCCCTCCTCAAAGATGAGATCCAAAGCTGCTCTTAGCCCGTAAAGTAGTTGGATGGAAGGCGTATACGGCCAGTAAGTTCCAAGATTGTAGAACTTCAAGTAGTCATTCCAGTCGAAGAAAACTCTGACCGACTTGGCATTTTTGGAAGCTTCCAGAGCTTTCGGGCTCGCGCAAACAATTCCCATTCCAGTAGGCAGGGAAA-GTGCTTTCTGCGA

>HEID131619_Tillandsia_capillaris

TGCAAGATCATTTTACGCCAATTATGTTACAAGAGGTCATACAATTATAAATGGCAACTAATTACAT-------------------------------------------------------------------------------------------------TCATCTTATATGCATATTCCTATATAT--------GAATAGATAATGCAATAC---TGTAAATTT-----AGAAAAAA------------GCTAAAAGAAA-ACAATTGGCTATTCAGTAAAAGCCAGCTTAATTTTTACAGTATACTCGAT--CGATAGATAGA---TTTTCGCATACTTACCTCGTGGCCTTTCCTAAACGACGATGCCTTTCGATGACATTTTCGAGGCCCTCCTCAAAGATGAGATCCAAAGCTGCTCTTAGCCCGTAAAGTAGTTGGATGGAAGGCGTATACGGCCAGTAAGTTCCAAGCTTGTAGAACTTCAAGTAGTCATTCCAGTCGAAGAAAACTCTGACCGACTTGGCATTTTTGGAAGCTTCCAGAGCTTTCGGGCTCGCGCAAACAATGCCCATTCCAGTAGGCAGGGAAAAGTGCTTTNNNNNN

>HEID131668_Tillandsia_retorta_Clone1

TGCATGATCATTTTACGCCAA---------------TCATACAATTATAAAAGGCAACTAATTATAT-------------------------------------------------------------------------------------------------TCATCTTA----CATATTTCTATATAT--------GAATAGATAATGCAATAC---TGTAAATT------AAAAAAAAAAAAAAA------CTAAAAGAAA-AC-----------------------------------------------T--CGATGGATAGA---TTTTTGCATACTTACCTCGTGGCCTTTCCTAAACGACGATGCCTTTCGATGACATTTTCGAGGCCCTCCTCAAAGATGAGATCCAAAGCTGCTCGTAGCCCGTAAAGTAGTTGGATGGAAGGCGTATACGGCCAGTAAGTTCCAAGCTTGTAGAACTTCAAGTAGTCATTCCAGTCGAAGAAAACTCTGACCGACTTGGCATTTTTGGAAGCTTCCATAGCTTTCGGGCTCGCGCAAACAATGCCCATTCCAGTAGGCAGGGAAA-GTGCTTTCTGCGA

>HEID131668_Tillandsia_retorta_Clone2

TGCATGATCATTTTACGCCAA---------------TCATACAATTATAAAAGGCAACTAATTATAT-------------------------------------------------------------------------------------------------TCATCTTA----CATATTTCTATATAT--------GAATAGATAATGCAATAC---TGTAAATT------AAAAAAAAAAAAAAAAA----CTAAAAGAAA-AC-----------------------------------------------T--CGATGGATAGA---TTTTTGCATACTTACCTCGTGGCCTTTCCTAAACGACGATGCCTTTCGATGACATTTTCGAGGCCCTCCTCAAAGATGAGATCCAAAGCTGCTCGTAGCCCGTAAAGTAGTTGGATGGAAGGCGTATACGGCCAGTAAGTTCCAAGCTTGTAGAACTTCAAGTAGTCATTCCAGTCGAAGAAAACTCTGACCGACTTGGCATTTTTGGAAGCTTCCATAGCTTTCGGGCTCGCGCAAACAATGCCCATTCCAGTAGGCAGGGAAA-GTGCTTTCTGCGA

>HEID131668_Tillandsia_retorta_Clone3

TGCATGATCATTTTACGCCAA---------------TCATACAATTATAAAAGGCAACTAATTATAT-------------------------------------------------------------------------------------------------TCATCTTA----CATATTTCTATATAT--------GAATAGATAATGCAATAC---TGTAAATT------AAAAAAAAAAAAAAAAA----CTAAAAGAAA-AC-----------------------------------------------T--CGATGGATAGA---TTTTTGCATACTTACCTCGTGGCCTTTCCTAAACGACGATGCCTTTCGATGACATTTTCGAGGCCCTCCTCAAAGATGAGATCCAAAGCTGCTCTTAGCCCGTAAAGTAGTTGGATGGAAGGCGTATACGGCCAGTAAGTTCCAAGCTTGTAGAACTTCAAGTAGTCATTCCAGTCGAAGAAAACTCTGACCGACTTGGCATTTTTGGAAGCTTCCAGAGCTTTCGGGCTCGCGCAAACAATGCCCATTCCAGTAGGCAGGGAAA-GTGCTTTCTGCGA

>HEID131668_Tillandsia_retorta_Clone4

TGCATGATCATTTTACGCCAA---------------TCATACAATTATAAAAGGCAACTAATTATAT-------------------------------------------------------------------------------------------------TCATCTTA----CATATTTCTATATAT--------GAATAGATAATGCAATAC---TGTAAATT------AAAAAAAAAAAAAAA------CTAAAAGAAA-AC-----------------------------------------------T--CGATGGATAGA---TTTTTGCATACTTACCTCGTGGCCTTTCCTAAACGACGATGCCTTTCGATGACATTTTCGAGGCCCTCCTCAAAGATGAGATCCAAAGCTGCTCGTAGCCCGTAAAGTAGTTGGATGGAAGGCGTATACGGCCAGTAAGTTCCAAGCTTGTAGAACTTCAAGTAGTCATTCCAGTCGAAGAAAACTCTGACCGACTTGGCATTTTTGGAAGCTTCCATAGCTTTCGGGCTCGCGCAAACAATGCCCATTCCAGTAGGCAGGGAAA-GTGCTTTCTGCGA

>HEID131739_Tillandsia_virescens

TGCAAGATCATTTTACGCCAATTATGTTACAAGAGGTCATACAATTATAAATGGCAACTAATTACAT-------------------------------------------------------------------------------------------------TCATCTTATATGCATATTCCTATATAT--------GAATAGATAATGCAATAC---TGTGAATTT-----AGAAAAAA------------GCTAAAAGAAA-ACAATCGGCTATTCAGTAAAAGCCAGCTTAATTTTTACAGTATACTCGAT--CGATAGATAGA---TTTTCGCATACTTACCTCGTGGCCTTTCCTAAACGACGATGCCTTTCGATGACATTTTCGAGGCCCTCCTCAAAGATGAGATCCAAAGCTGCTCTTAGCCCGTAAAGTAGTTGGATGGAAGGCGTATACGGCCAGTAAGTTCCAAGCTTGTAGAACTTCAAGTAGTCATTCCAGTCGAAGAAAACTCTGACCGACTTGGCATTTTTGGAAGCTTCCAGAGCTTTCGGGCTCGCGCAAACAATGCCCATTCCAGTAGGCAGGGAAA-GTGCTTTNNNNNN

>HEID131764_Tillandsia_caliginosa_Clone1

TGCAAGATCATTTTACGCCAA---------------TCATACAATTATAAATGACAACTAATTATAT-------------------------------------------------------------------------------------------------TCATCTTA----CATATTTCTATA----------------------------C---TGTAA-TTTTTTTTAAAAAAAAAA-----------CTAAAAGAAA-ATAATCGTCTATTCAGTAAAAGCCAGCTTAATTTTTACAGTATACTCGAT--GGATAGA-------TTTTTGCATACTTACCTCGTGGCCTTTCCTAAACGACGATGCCTTTCGATGACATTTTCGAGCCCCTCCTCAAAGATGAGATCCAAAGCTGCTCTTAGCCCGTAAAGTAGTTGGATGGAAGGCGTATACGGCCAGTAAGTTCCAAGCTTGTAGAACTTCAAATAGTCATTCCAGTCGAAGAAAACTCTGACCGACTTGGCATTTTTGGAAGCTTCCAGAGCTTTCGGGCTCGCGCAAACAATGCCCATTCCAGTAGGCAGGGAAA-GTGCTTTCTGCGA

>HEID131764_Tillandsia_caliginosa_Clone2

TGCAAGATCATTTTACGCCAATTATGTTACAAGAGGTCATACAATTATAAATGGCAACTAATTACAT-------------------------------------------------------------------------------------------------CC-TCTTA----CATATTCC------------------TAGATAATGCAATAC---TGTAAATTT-----AGAAAAAA------------GCTAAAAGAAA-ACAATCGGCTATTCAGTAAAAGCCAGCTTAATTTTTACAGTATACTCGAT--CGATAGA-------TTTTTGCATACTTACCTCGTGGCCTTTCCTAAACGACGATGCCTTTCGATGACATTTTCGAGGCCCTCCTCAAAGATGAGATCCAAAGCTGCTCTTAGCCCGTAAAGTAGTTGGATGGAAGGCGTATACGGCCAGTAAGTTCCAAGCTTGCAGAACTTCAAGTAGTCATTCCAGTCGAAGAAAACTCTGACCGACTTGGCATTTTTGGAAGCTTCCAGAGCTTTCGGGCTCGCGCAAACAATGCCCATTCCAGTAGGCAGGGAAA-GTGCTTTCTGCGA

>HEID131764_Tillandsia_caliginosa_Clone3

TGCAAGATCATTTTACGCCAA---------------TCATACAATTATAAATGACAACTAATTATAT-------------------------------------------------------------------------------------------------TCATCTTA----CATATTTCTATA----------------------------C---TGTAA-TTTTTTTTAAAAAAAAAA-----------CTAAAAGAAA-ATAATCGTCTATTCAGTAAAAGCCAGCTTAATTTTTACAGTATACTCGAT--GGATAGA-------TTTTTGCATACTTACCTCGTGGCCTTTCCTAAACGACGATGCCTTTCGATGACATTTTCGAGCCCCTCCTCAAAGATGAGATCCAAAGCTGCTCTTAGCCCGTAAAGTAGTTGGATGGAAGGCGTATACGGCCAGTAAGTTCCAAGCTTGTAGAACTTCAAATAGTCATTCCAGTCGAAGAAAACTCTGACCGACTTGGCATTTTTGGAAGCTTCCAGAGCTTTCGGGCTCACGCAAACAATGCCCATTCCAGTAGGCAGGGAAA-GTGCTTTCTGCGA

>HEID131780_Tillandsia_pupurea_Clone1

TGTAAGATCATTTTACACCAA---------------TCATACAATTATAAATGCCAACTAATTATAT-------------------------------------------------------------------------------------------------ACATCTTA----CATATTCCTATATAT--------GAATAGATAATGCAATAC---TGTAAATTT-----AGGAAAAAA-----------GCTAAAAGAAA-ACAATCGGCTATTCAGTAAAAA----------------------------------------------TTTGCATACTTACCTCGTGGCCTTTCCTAAACGATGATGCCTTTCGATGACATTTTCGAGCCCCTCCTCAAAGATGAGATCCAAAGCTGCTCTTAGCCCGTAAAGTAGTTGGATGGAAGGCGTATACGGCCAGTAAGTTCCAAGCTTGTAGAACTTCAAGTAGTCATTCCAGTCGAAGAAAACTCTGACCGACTTGGCATTTTTGGAAGCTTCTAGAGCTTTCGGGCTCGCGCAAACAATGCCCATTCCAGTAGGCAGGGAAA-GTGCTTTCTGCGA

>HEID131780_Tillandsia_pupurea_Clone2

TGTAAGATCATTTTACACCAA---------------TCATACAATTATAAATGCCAACTAATTATAT-------------------------------------------------------------------------------------------------ACATCTTA----CATATTCCTATATAT--------GAATAGATAATGCAATAC---TGTAAATTT-----AGGAAAAAA-----------GCTAAAAGAAA-ACAATCGGCTATTCAGTAAAAA----------------------------------------------TTTGCATACTTACCTCGTGGCCTTTCCTAAACGATGATGCCTTTCGATGACATTTTCGAGCCCCTCCTCAAAGATGAGATCCAAAGCTGCTCTTAGCCCGTAAAGTAGTTGGATGGAAGGCGTATACGGCCAGTAAGTTCCAAGCTTGTAGAACTTCAAGTAGTCATTCCAGTCGAAGAAAACTCTGACCGACTTGGCATTTTTGGAAGCTTCTAGAGCTTTCGGGCTCGCGCAAACAATGCCCATTCCAGTAGGCAGGGAAA-GTGCTTTCTGCGA

>HEID131780_Tillandsia_pupurea_Clone3

TGTAAGATCATTTTACACCAA---------------TCATACAATTATAAATGCCAACTAATTATAT-------------------------------------------------------------------------------------------------ACATCTTA----CATATTCCTATATAT--------GAATAGATAATGCAATAC---TGTAAATTT-----AGGAAAAAA-----------GCTAAAAGAAA-ACAATCGGCTATTCAGTAAAAA----------------------------------------------TTTGCATACTTACCTCGTGGCCTTTCCTAAACGATGATGCCTTTCGATGACATTTTCGAGCCCCTCCTCAAAGATGAGATCCAAAGCTGCTCTTAGCCCGTAAAGTAGTTGGATGGAAGGCGTATACGGCCAGTAAGTTCCAAGCTTGTAGAACTTCAAGTAGTCATTCCAGTCGAAGAAAACTCTGACCGACTTGGCATTTTTGGAAGCTTCTAGAGCTTTCGGGCTCGCGCAAACAATGCCCATTCCAGTAGGCAGGGAAA-GTGCTTTCTGCGA

>HEID131787_Tillandsia_virescens_Clone1

TGCAAGATCATTTTACGGCAATTATATTACAAGAGGTCATACAATTATAAATGGCAACTAATTGCAT-------------------------------------------------------------------------------------------------TCATCTTATATGCATATTCCTATATAT--------GAATAGATAATGCAATAC---TGTAAATTT-----AGAAAAAAA------------CTAAAAGAAA-ACAATTGGCTATTCAGTAAAAGCCAGCTTAATTTTTACAGTATACTCGAT--CGATAGATAGA---TTTTCGCATACTTACCTCGTGGCCTTTCCTAAACGACGATGCCTTTCGATGACATTTTCGAGGCCCTCCTCAAAGATGAGATCCAAAGCTGCTCTTAGCCCGTAAAGTAGTTGGATGGAAGGCGTATACGGCCAGTAAGTTCCGAGCTTGTAGAACTTCAAGTAGTCATTCCAGTCGAAGAAAACTCTGACCGACTTGGCATTTTTGGAAGCTTCCAGAGCTTTCGGGCTCGCGCAAACAATGCCCATTCCAGTAGGCAGGGAAA-GTGCTTTCTGCGA

>HEID131787_Tillandsia_virescens_Clone2

TGCAAGATCATTTTACGCCAA---------------TCATACAATTATAAATGGCAACTAATTGCAT-------------------------------------------------------------------------------------------------TCATCTTATATGCATATTCCTATATAT--------GAATAGATAATGCAATAC---TGTAAATTT-----AGAAAAAAA------------CTAAAAGAAA-ACAATTGGCTATTCAGTAAAAGCCAGCTTAATTTTTACAGTATACTCGAT--CGATAGATAGA---TTTTCGCATACTTACCTCGTGGCCTTTCCTAAACGACGATGCCTTTCGATGACATTTTCGAGGCCCTCCTCAAAGATGAGATCCAAAGCTGCTCTTAGCCCGTAAAGTAGTTGGATGGAAGGCGTATACGGCCAGTAAGTTCCGAGCTTGTAGAACTTCAAGTAGTCATTCCAGTCGAAGAAAACTCTGACCGACTTGGCATTTTTGGAAGCTTCCAGAGCTTTCGGGCTCGCGCAAACAATGCCCATTCCAGTAGGCAGGGAAA-GTGCTTTCTGCGA

>HEID131787_Tillandsia_virescens_Clone3

TGCAAGATCATTTTACGGCAATTATATTACAAGAGGTCATACAATTATAAATGGCAACTAATTGCAT-------------------------------------------------------------------------------------------------TCATTTTATATGCATATTCCTATATAT--------GAATAGATAATGCAATAC---TGTAAATTT-----AGAAAAAA------------GCTAAAAGAAA-ACAATCGGCTATTCAGTAAAAGCCAGTTTAATTTTTACAGTATACTCGAT--CGATAGATAGA---TTTTTGCATACTTACCTCGTGGCCTTTCCTAAACGACGATGCCTTTCGATGACATTTTCGAGGCCCTCCTCAAAGATGAGATCCAAAGCTGCTCTTAGCCCGTAAAGTAGTTGGATGGAAGGCGTATACGGCCAGTAAGTTCCAAGTTTGTAGAACTTCAAGTAGTCATTCCAGTCGAAGAAAACTCTGACCGACTTGGCATTCTTGGAAGCTTCCAGAGCTTTCGGGCTCGCGCAAACAATGCCCATTCCAGTAGGCAGGGAAA-GTGCTTTCTGCGA

>HEID131788_Tillandsia_recurvata_Clone1

TGCAAGATCATTTTACGCCAA---------------TCATACAATTATAAATGGCAACTAATTATAT-------------------------------------------------------------------------------------------------TCATCTTA----CATATTCCTATA----------------------------C---TGTAAATTT-----AAAAAAAAAAAAAAAA-----TTAAAAGAAA-ATAATCGTCTATTCAGTAAAAGCCAGCTTAATTTTTACAGTATACTCGAT--CGATAGA-------TTTTTGCATACTTACCTCGTGGCCTTTCCTAAACGACGATGCCTTTCGATGACATTTTCGAGCCCCTCCTCAAAGATGAGATCCAAAGCTGCTCTTAGCCCGTAAAGTAGTTGGATGGAAGGCGTATACGGCCAGTAAGTTCCAAGCTTGTAGAACTTCAAATAGTCATTCCAGTCGAAGAAAACTCTGACCGACTTGGCATTTTTGGAAGCTTCCAGAGCTTTCGGGCTCGCGCAAATAATGCCCATTCCAGTAGGCAGGGAAA-GTGCTTTCTGCGA

>HEID131788_Tillandsia_recurvata_Clone2

TGCAAGATCATTTTACGCCAA---------------TCATACAATTATAAATGGCAACTAATTATAT-------------------------------------------------------------------------------------------------TCATCTTA----CATATTCCTATA----------------------------C---TGTAAATTT-----AAAAAAAAAAAAAAAAAAA--TTAAAAGAAA-ATAATCGTCTATTCAGTAAAAGCCAGCTTAATTTTTACAGTATACTCGAT--CGATAGA-------TTTTTGCATACTTACCTCGTGGCCTTTCCTAAACGACGATGCCTTTCGATGACATTTTCGAGCCCCTCCTCAAAGATGAGATCCAAAGCTGCTCTTAGCCCGTAAAGTAGTTGGATGGAAGGCGTATACGGCCAGTAAGTTCCAAGCTTGTAGAACTTCAAATAGTCATTCCAGTCGAAGAAAACTCTGACCGACTTGGCATTTTTGGAAGCTTCCAGAGCTTTCGGGCTCGCGCAAATAATGCCCATTCCAGTAGGCAGGGAAA-GTGCTTTCTGCGA

>HEID131788_Tillandsia_recurvata_Clone3

TGCAAGATCATTTTACGCCAA---------------TCATACAATTATAAATGGCAACTAATTATAT-------------------------------------------------------------------------------------------------TCATCTTA----CATATTCCTATA----------------------------C---TGTAAATTT-----AAAAAAAAAAAAAAAAAAAA-TTAAAAGAAA-ATAATCGTCTATTCAGTAAAAGCCAGCTTAATTTTTACAGTATACTCGAT--CGATAGA-------TTTTTGCATACTTACCTCGTGGCCTTTCCTAAACGACGATGCCTTTCGATGACATTTTCGAGCCCCTCCTCAAAGATGAGATCCAAAGCTGCTCTTAGCCCGTAAAGTAGTTGGATGGAAGGCGTATACGGCCAGTAAGTTCCAAGCTTGTAGAACTTCAAATAGTCATTCCAGTCGAAGAAAACTCTGACCGACTTGGCATTTTTGGAAGCTTCCAGAGCTTTCGGGCTCGCGCAAATAATGCCCATTCCAGTAGGCAGGGAAA-GTGCTTTCTGCGA

>HEID131788_Tillandsia_recurvata_Clone4

TGCAAGATCATTTTACGCCAA---------------TCATACAATTATAAATGGCAACTAATTATAT-------------------------------------------------------------------------------------------------TCATCTTA----CATATTCCTATA----------------------------C---TGTAAATTT-----AAAAAAAAAAAAAAAAA----TTAAAAGAAA-ATAATCGTCTATTCAGTAAAAGCCAGCTTAATTTTTACAGTATACTCGAT--CGATAGA-------TTTTTGCATACTTACCTCGTGGCCTTTCCTAAACGACGATGCCTTTCGATGACATTTTCGAGCCCCTCCTCAAAGATGAGATCCAAAGCTGCTCTTAGCCCGTAAAGTAGTTGGATGGAAGGCGTATACGGCCAGTAAGTTCCAAGCTTGTAGAACTTCAAATAGTCATTCCAGTCGAAGAAAACTCTGACCGACTTGGCATTTTTGGAAGCTTCCAGAGCTTTCGGGCTCGCGCAAATAATGCCCATTCCAGTAGGCAGGGAAA-GTGCTTTCTGCGA

>HEID131923_Tillandsia_recurvata_Clone1

TGCAAGATCATTTTACGCCAATTATGTTACAAGAGGTCATACAATTATAAATGGCAACTAATTACAT-------------------------------------------------------------------------------------------------TCATCTTATATGCATATTCCTATATAT--------GAATAGATAATGCAATAC---TGTAAATTT-----AGAAAAAA------------GCTAAAAGAAA-ACAATTGGCTATTCAGTAAAAGTCAGCTTAATTTTTACAGTATACTCGAT--CGATAGA-------TTTTTGCATACTTACCTCGTGGCCTTTCCTAAACGACGATGCCTTTCGATGACATTTTCGAGGCCCTCCTCAAAGATGAGATCCAAAGCTGCTCTTAGCCCGTAAAGTAGTTGGATGGAAGGCGTATACGGCCAGTAAGTTCCGAGCTTGTAGAACTTCAAGTAGTCATTCCAGTCGAAGAAAACTCTGACCGACTTGGCATTTTTGGAAGCTTCCAGAGCTTTCGGGCTCGCGCAAACAATGCCCATTCCAGTAGGCAGGGAAA-GTGCTTTCTGCGA

>HEID131923_Tillandsia_recurvata_Clone2

TGCAAGATCATTTTACGCCAATTATGTTACAAGAGGTCATACAATTATAAATGGCAACTAATTACAT-------------------------------------------------------------------------------------------------TCATCTTATATGCATATTCCTATATAT--------GAATAGATAATGCAATAC---TGTAAATTT-----AGAAAAAA------------GCTAAAAGAAA-ACAATTGGCTATTCAGTAAAAGTCAGCTTAATTTTTACAGTATACTCGAT--CGATAGA-------TTTTTGCATACTTACCTCGTGGCCTTTCCTAAACGACGATGCCTTTCGATGACATTTTCGAGGCCCTCCTCAAAGATGAGATCCAAAGCTGCTCTTAGCCCGTAAAGTAGTTGGATGGAAGGCGTATACGGCCAGTAAGTTCCGAGCTTGTAGAACTTCAAGTAGTCATTCCAGTCGAAGAAAACTCTGACCGACTTGGCATTTTTGGAAGCTTCCAGAGCTTTCGGGCTCGCGCAAACAATGCCCATTCCAGTAGGCAGGGAAA-GTGCTTTCTGCGA

>HEID131923_Tillandsia_recurvata_Clone3

TGCAAGATCATTTTACGCCAATTATGTTACAAGAGGTCATACAATTATAAATGGCAACTAATTACAT-------------------------------------------------------------------------------------------------TCATCTTATATGCATATTCCTATATAT--------GAATAGATAATGCAATAC---TGTAAATTT-----AGAAAAAAA------------CTAAAAGAAA-ACAATTGACTATTCAGTAAAAGCCAGCTTAATTTTTACAGTATACTCGAT--CGATAGATAGA---TTTTTGCATACTTACCTCGTGGCCTTTCCTAAACGACGATGCCTTTCGATGACATTTTCGAGGCCCTCCTCAAAGATGAGATCCAAAGCTGCTCTTAGCCCGTAAAGTAGTTGGATGGAAGGCGTATACGGCCAGTAAGTTCCAAGCTTGTAGAACTTCAAGTAGTCATTCCAGTCGAAGAAAACTCGGACCGACTTGGCATTTTTGGAAGCTTCCAGAGCTTTTGGGCTCGCGCAAACAATGCCCATTCCAGTAGGCAGGGAAA-GTGCTTTCTGCGA

>HEID132151_Tillandsia_latifolia_Clone1

TGCAAGATCATTTTACGCCAA---------------TCATACAATTATAAATGCCAACTAATTATAT-------------------------------------------------------------------------------------------------CCATCTTA----CATATTCCTATATATAT------GAATAGATAATGCAATAC---TGTAAATTT-----AGGAGAAA------------GCTAAAAGAAA-ACAATCGGCTATTCAGTAAAAGCCAGC----TTTTTACAGTA--CTCGAT--AGATAGA-AG------TTTGCATACTTACCTCGTGGCTTTTCCTAAACGATGATGCCTTTCAATGACATTTTCGAGCCCCTCCTCAAAGATGAGATCCAAAGCTGCTCTTAGCCCGTAAAGTAGTTGGATGGAAGGCGTATACGGCCAGTAAGTTCCAAGCTTGTAGAACTTCAAGTAGTCATTCCAGTCGAAGAAAACTCTGACCGACTTGGCATTTTTTGAAGCTTCTAGAGCTTTCGGGCTCGCGCAAACAATGCCCATTCCAGTAGGCAGGGAAA-GTGCTTTCTGCGA

>HEID132151_Tillandsia_latifolia_Clone2

TGCAAGATCATTTTACGCCAA---------------TCATACAATTATAAATGCCAACTAATTATAT-------------------------------------------------------------------------------------------------CCATCTTA----CATATTCCTATATATAT------GAATAGATAATGCAATAC---TGTAAATTT-----AGGAGAAA------------GCTAAAAGAAA-ACAATCGGCTATTCAGTAAAAGCCAGC----TTTTTACAGTA--CTCGAT--AGATAGA-AG------TTTGCATACTTACCTCGTGGCTTTTCCTAAACGATGATGCCTTTCAATGACATTTTCGAGCCCCTCCTCAAAGATGAGATCCAAAGCTGCTCTTAGCCCGTAAAGTAGTTGGATGGAAGGCGTATACGGCCAGTAAGTTCCAAGCTTGTAGAACTTCAAGTAGTCATTCCAGTCGAAGAAAACTCTGACCGACTTGGCATTTTTTGAAGCTTCTAGAGCTTTCGGGCTCGCGCAAACAATGCCCATTCCAGTAGGCAGGGAAA-GTGCTTTCTGCGA

>HEID132151_Tillandsia_latifolia_Clone3

TGCAAGATCATTTTACGCCAA---------------TCATACAATTATAAATGCCAACTAATTATAT-------------------------------------------------------------------------------------------------CCATCTTA----CATATTCCTATATATAT------GAATAGATAATGCAATAC---TGTAAATTT-----AGGAAAAA------------GCTAAAAGAAA-ACAATCGGCTATTCAGTAAAAGCCAGC----TTTTTACAGTA--CTCGAT--CGATCGATAGAAG---TTTGCATACTTACCTCGTGGCCTTTCCTAAACGATGATGCCTTTCGATGACATTTTCGAGCCCCTCCTCAAAGATGAGATCCAAAGCTGCTCTTAGCCCGTAAAGTAGTTGGATGGAAGGCGTATACGGCCAGTAAGTTCCGAGCTTGTAGAACTTCAAGTAGTCATTCCAGTCGAAGAAAACTCTGACCGACTTGGCATTTTTGGAAGCTTCTAGAGCTTTCGGGCTCACGCAAACAATGCCCATTCTAGTAGGCAGGGAAA-GTGCTTTCTGCGA

>HEID132229_Tillandsia_latifolia_var_divaricata

TGCAAGATCATTTTACGCCAATTATGTTACAAGAGGTCATACAATTATAAATGCCAACTAATTATAT-------------------------------------------------------------------------------------------------CCATCTTA----CATACTCCTATATAT--------GAATAGATAATGCAATAC---TGTAAATTT-----AGGAAAAA------------GCTAAAAGAAA-ACAATCAGCTATTCAGTAAAAGCCAGC----TTTTTACACTA--CTCGATCTCGATAGA-AG------TTTGCATACTTACCTCGTGGCCTTTCCTAAACGATGATGCCTTTCGATGACATTTTCGAGCCCCTCCTCAAAGATGAGATCCAAAGCTGCTCTTAGCCCGTAAAGTAGTTGGATGGAAGGCGTATACGGCCAGTAAGTTCCGAGCTTGTAGAACTTCAAGTAGTCATTCCAGTCGAAGAAAACTCTGACCGACTTGGCATTTTTTGAAGCTTCTAGAGCTTTCGGGCTCGCGCAAACAATGCCCATTCCAGTAGGCAGGGAAA-GTGCTTTNNNNNN

>HEID132237_Tillandsia_arequitae_Clone1

TGCAAGATCATTTTACGCCAA---------------TCATACAATTATAAATGGCAACTAATTACAT-------------------------------------------------------------------------------------------------CCATCTTA----CATATTCCTATATAT--------GAATAGATAATGCAATAC---TGTAAATTT-----AGAAAAAA------------GCTAAAAGAAA-ACAATCGCCTATTCAGTAAAAGCCAGCTTAATTTTTACAGTATACTCGAT--CGATAGA-------TTTTTGCATACTTACCTCGTGGCCTTTCCTAAATGACGATGCCTTTCGATGACATTTTCGAGCCCCTCCTCAAAGATGAGATCCAAAGCTGCTTCTAGCCCGTAAAGTAGTTGGATGGAAGGCGTATACGGCCAGTAAGTTCCAAGCTTGTAGAACTTCAAGTAGTCATTCCAGTCGAAGAAAACTCTGACCGNNNNNNNNNNNNNNNNNNNNNNNNNNNNNNNNNNNNNNNNNNNNNNNNNNNNNNNNNNNNNNNNNNNNNNNNNNNNNNNNNNNNNN

>HEID132237_Tillandsia_arequitae_Clone2

TGCAAGATCATTTTACGCCAATTATGTTACAAGAGGTCATACAATTATAAATGGCAACTAATTACAT-------------------------------------------------------------------------------------------------CC-TCTTA----CATATTCC------------------TAGATAATGCAATAC---TGTAAATTT-----AGAAAAAA------------GCTAAAAGAAA-ACAATCGGCTATTAAGTAAAAGCCAGCTTAATTTTTACAGTATACTCGAT--CGATAGA-------TTTTTGCATACTTACCTCGTGGCCTTTCCTAAACGACGATGCCTTTCGATGACATTTTCGAGCCCCTCCTCAAAGATGAGATCCAAAGCTGCTCTTAGCCCGTAAAGTAGTTGGATGGAAGGTGTATACGGCCAGTAAGTTCCAAGCTTGTAGAACTTCAAGTAGTCATTCCAGTCGAAGAAAACTCTGACCGACTTGGCATTTTTGGAAGCTTCCAGAGCTTTCGGGCTCGCGCAAACAATGCCCATTCCAGTAGGCAGGGAAA-GTGCTTTCTGCGA

>HEID132237_Tillandsia_arequitae_Clone3

TGCAAGATCATTTTACGCCAA---------------TCATACAATTATAAATGACAACTAATTATAT-------------------------------------------------------------------------------------------------TCATCTTA----CATATTTCTATA----------------------------C---TGTAA-TTTTTTTTAAAAAAAAAA-----------CTAAAAGAAA-ATAATCGTCTATTCAGTAAAAGCCAGCTTAATTTTTACAGTATACTCGAT--GGATAGA-------TTTTTGCATACTTACCTCGTGGCCTTTCCTAAACGACGATGCCTTTCGATGACATTTTCGAGCCCCTCCTCAAAGATGAGATCCAAAGCTGCTCTTAGCCCGTAAAGTAGTTGGATGGAAGGCGTATACGGCCAGTAAGTTCCAAGCTTGTAGAACTTCAAATAGTCATTCAAGTCGAAGAAAACTCTGACCGACTTGGCATTTTTGGAAGCTTCCAGAGCTTTCGGGCTCGCGCAAACAATGCCCATTCCAGTAGGCAGGGAAA-GTGCTTTCTGCGA

>HEID132280_Tillandsia_cauligera_Clone1

TGCAAGATCATTTTACGCCAATTATGTTACAAGAGGTCATACAATTATAAATGCCAACTAATTATAT-------------------------------------------------------------------------------------------------CCATCTTA----CATATTCCTATATAT--------GAATAGATAATGCAATAC---TGTAAATTT-----AGGAAAAAA------------CTAAAAGAAA-ACAATCGGCTATT--------------------------------TCGAT--GGATAGA-AG------TTTGCATACTTACCTCGTGGCCTTTCCTAAACGACGATGCCTTTCGATGACATTTTCGAGCCCCTCCTCAAAGATGAGATCCAAAGCTGCTCTTAGCCCGTAAAGTAGTTGGATGGAAGGCGTATACGGCCAGTAAGTTCCAAGCTTGTAGAACTTCAAGTAGTCATTCCAGTCGAAGAAAACTCTGACCGACTTGGCATTTTTTGAAGCTTCTAGAGCTTTCGGGCTCGCGCAAACAATGCCCATTCCAGTAGGCAGGGAAA-GTGCTTTCTGCGA

>HEID132280_Tillandsia_cauligera_Clone2

TGCAAGATCATTTTACGCCAATTATGTTACAAGAGGTCATACAATTATAAATGGCAACTAATTACAT-------------------------------------------------------------------------------------------------CC-TCTTA----CATATTCC------------------TAGATAATGCAGTAC---TGTAAATTT-----AGAAAAAAA------------CTAAAAGAAA-ACAATCGGCTATTCNNNNNNNNNNNNNNNNNNNNNNNNNNNNNNNNNNNN--NNNNNNN-------NTTTTGCATACTTACCTCGTGGCCTTTCCTAAACGATGATGCCTTTCGATGACATTTTCGAGCCCCTCCTCAAAGATGAGATCCAAAGCTGCTCTTAGCCCGTAAAGTAGTTGGATGGAAGGCGTATACGGCCAGTAAGTTCCGAGCTTGTAGAACTTCAAGTAGTCATTCCAGTCGAAGAAAACTCTGACCGACTTGGCATTTTTGGAAGCTTCTAGAGCTTTCGGGCTCGCGCAAACAATGCCCATTCCAGTAGGCAGGGAAA-GTGCTTTCTGCGA

>HEID132280_Tillandsia_cauligera_Clone3

TGCAAGATCATTTTACGCCAATTATGTTACAAGAGGTCATACAATTATAAATGGCAACTAATTACAT-------------------------------------------------------------------------------------------------CC-TCTTA----CATATTCC------------------TAGATAATGCAATAC---TGTAAATTT-----AGAAAAAA------------GCTAAAAGAAA-ACAATCGGCTATTCAGTAAAAGCCAGCTTAATTTTTACAGTATACTCGAT--CGATATA-------TTTTTGCATACTTACCTCGTGGCCTTTCCTAAACGACGATGCCTTTCGATGACATTTTCGAGGCCCTCCTCAAAGATGAGATCCAAAGCTGCTCTTAGCCCGTAAAGTAGTTGGATGGAAGGCGTATACGGCCAGTAAGTTCCAAGCTTGTAGAACTTCAAGTAGTCATTCCAGTCGAAGAAAACTCTGACCGACTTGGCATTTTTGGAAGCTTCCAGAGCTTTCGGGCTCGCGCAAACAATGCCCATTCCAGTAGGCAGGGAAA-GTGCTTTCTGCGA

>HEID132283_Tilllandsia_rectangula_Clone1

TGCAAGATCATTTTACGCCAATTATGTTACAAGAGGTCATACAATTATAAATGGCAACTAATTACAT-------------------------------------------------------------------------------------------------CC-TCTTA----CATATTCC------------------TAGATAATGCAATAC---TGTAAATTT-----AGAAAAAA------------GCTAAAAGAAA-ACAATCGGCTATTCAGTAAAAGCCAGCTTAATTTTTACAGTATACTCGAT--CGATAGA-------TTTTTGCATACTTACCTCGTGGCCTTTCCTAAACGACGATGCCTTTCGATGACATTTTCGAGGCCCTCCTCAAAGATGAGATCCAAAGCTGCTCTTAGCCCGTAAAGTAGTTGGATGGAAGGCGTATACGGCCAGTAAGTTCCAAGCTTGTAGAACTTCAAGTAGTCATTCCAGTCGAAGAAAACTCTGACCGACTTGGCATTTTTGGAAGCTTCCAGAGCTTTCGGGCTCGCGCAAACAATGCCCATTCCAGTAGGCAGGGAAA-GTGCTTTCTGCGA

>HEID132283_Tilllandsia_rectangula_Clone2

TGCAAGATCATTTTACGCCAATTATGTTACAAGAGGTCATACAATTATAAATGGCAACTAATTACAT-------------------------------------------------------------------------------------------------CCATCTTATATACATATTCCTAAATATATATAT--GAATAGATAATGCAATAC---TGTAAATTT-----AGAAAAAA------------GCTAAAAGAAA-ACAATCTGCTATTCAGTAAAAGCCAGCTTAATTTTTACAGTATACTCGAT--CGATAGA-------TTTTTGCATACTTACCTCGTGGCCTTTCCTAAACGACGATGCCTTTCGATGACATTTTCGAGGCCCTCCTCAAAGATGAGATCCAAAGCTGCTCTTAGCCCGTAAAGTAGTTGGATGGAAGGCGTATACGGCCAGTAAGTTCCAAGCTTGTAGAACTTCAAGTAGTCATTCCAGTCGAAGAAAACTCTGACCGACTTGGCATTTTTGGAAGCTTCCAGAGCTTTCGGGCTCGCGCAAACAATGCCCATTCCAGTAGGCAGGGAAA-GTGCTTTCTGCGA

>HEID132283_Tilllandsia_rectangula_Clone3

TGCAAGATCATTTTACGCCAATTATGTTACAAGAGGTCATACAATTATAAATGGCAACTAATTACAT-------------------------------------------------------------------------------------------------CC-TCTTA----CATATTCC------------------TAGATAATGCAATAC---TGTAAATTT-----AGAAAAAA------------GCTAAAAGAAA-ACAATCGGCTATTCAGTAAAAGCCAGCTTAATTTTTACAGTATACTCGAT--CGATAGA-------TTTTTGCATACTTACCTCGTGGCCTTTCCTAAACGACGATGCCTTTCGATGACATTTTCGAGGCCCTCCTCAAAGATGAGATCCAAAGCTGCTCTTAGCCCGTAAAGTAGTTGGATGGAAGGCGTATACGGCCAGTAAGTTCCAAGCTTGTAGAACTTCAAGTAGTCATTCCAGTCGAAGAAAACTCTGACCGACTTGGCATTTTTGGAAGCTTCCAGAGCTTTCGGGCTCGCGCAAACAATGCCCATTCCAGTAGGCAGGGAAA-GTGCTTTCTGCGA

>HEID132288_Tillandsia_duratii_Clone1

TGCAAGATCATTTTACGCCAATTATGTTACAAGAGGTCATACAATTATAAATGGCAACTAATTACAT-------------------------------------------------------------------------------------------------CC-TCTTA----CATATTCC------------------TAGATAATGCAATAC---TGTAAATTT-----AGAAAAAA------------GCTAAAAGAAA-ACAATCGGCTATTCAGTAAAAGCCAGCTTAATTTTTACAGTATACTCGAT--CGATAGA-------TTTTTGCATACTTACCTCGTGGCCTTTCCTAAACGACGATGCCTTTCGATGACATTTTCGAGGCCCTCCTCAAAGATGAGATCCAAAGCTGCTCTTAGCCCGTAAAGTAGTTGGATGGAAGGCGTATACGGCCAGTAAGTTCCAAGCTTGTAGAACTTCAAGTAGTCATTCCAGTCGAAGAAAACTCTGACCGACTTGGCATTTTTGGAAGCTTCCAGAGCTTTCGGGCTCGCGCAAACAATGCCCATTCCAGTATGCAGGGAAA-GTGCTTTCTGCGA

>HEID132288_Tillandsia_duratii_Clone2

TGCAAGATCATTTTACGCCAATTATGTTACAAGAGGTCATACAATTATAAATGGCAACTAATTACAT-------------------------------------------------------------------------------------------------CC-TCTTA----CATATTCC------------------TAGATAATGCAATAC---TGTAAATTT-----AGAAAAAA------------GCTAAAAGAAA-ACAATCGGCTATTCAGTAAAAGCCAGCTTAATTTTTACAGTATACTCGAT--CGATAGA-------TTTTTGCATACTTACCTCGTGGCCTTTCCTAAACGACGATGCCTTTCGATGACATTTTCGAGCCCCTCCTCAAAGATGAGATCCAAAGCTGCTCTTAGCCCGTAAAGTAGTTGGATGGAAGGCGTATACGGCCAGTAAGTTCCAAGCTTGTAGAACTTCAAATAGTCATTCCAGTCGAAGAAAACTCTGACCGACTTGGCATTTTTGGAAGCTTCCAGAGCTTTCGGGCTCGCGCAAACAATGCCCATTCCAGTAGGCAGGGAAA-GTGCTTTCTGTGA

>HEID132288_Tillandsia_duratii_Clone3

TGCAAGATCATTTTACGCAAA---------------TCATACAATTATAAATGACAACTAATTATAT-------------------------------------------------------------------------------------------------TCATCTTA----CATATTTCTATA----------------------------C---TGTAA-TTTTTTTTAAAAAAAA-------------CTAAAAGAAA-ATAATCGTCTATTCAGTAAAAGCCAGCTTAATTTTTACAGTATACTCGAT--GGATAGA-------TTTTTGCATACTTACCTCGTGGCCTTTCCTAAACGACGATGCCTTTCGATGACATTTTCGAGCCCCTCCTCAAAGATGAGATCCAAAGCTGCTCTTAGCCCGTAAAGTAGTTGGATGGAAGGCGTATACGGCCAGTAAGTTCCAAGCTTGTAGAACTTCAAATAGTCATTCCAGTCGAAGAAAACTCTGACCGACTTGGCATTTTTGGAAGCTTCCAGAGCTTTCGGGCTCGCGCAAACAATGCCCATTCCAGTAGGCAGGGAAA-GTGCTTTCTGCGA

>HEID132290_Tillandsia_xiphioides_Clone1

TGCAAAATCATTTTACGCCAA---------------TCATACAATTATAAATGGCAACTAATTACAT-------------------------------------------------------------------------------------------------CCATCTTA----CATATTCCTATATAT--------GAATAGATAATGCAATAC---TGTAAATTT-----AGAAAACA------------GCTAAAAGAAACACAATCGGCTATTCAGTAAAAGCCAGCTTAATTTTTACAGTATACTCGAT--CGATAGA------TTTTTTGCATACTTACCTCGTGGCCTTTCCTAAACGACGATGCCTTTCGATGACATTTTCGAGCCCCTCCTCAAAGATGAGATCCAAAGCTGCTCTTAGCCCGTAAAGTAGTTGGATGGAAGGCGTATACGGCCAGTAAGTTCCAAGCTTGTAGAACTTCAAGTAGTCATTCCAGTCGAAGAAAACTCTAACCGACTTGGCATTTTTGGAAGCTTCCAGAGCTTTCGGGCTCGCGCAAACAATGCCCATTCCAGTAGGCAGGGAAA-GTGCTTTCTGCGA

>HEID132290_Tillandsia_xiphioides_Clone2

TGCAAGATCATTTTACGCCAATTATGTTACAAGAGGTCATACAATTATAAATGGCAACTAATTACAT-------------------------------------------------------------------------------------------------CC-TCTTA----CATATTCC------------------TAGATAATGCAATAC---TGTAAATTT-----AGAAAAAA------------GCTAAAAGAAA-ACAATCGGCTATTCAGTAAAAGCCAGCTTAATTTTTACAGTATACTCGAT--CGATAGA-------TTTTTGCATACTTACCTCGTGGCCTTTCCTAAACGACGATGCCTTTCGATGACATTTTCGAGGCCCTCCTCAAAGATGAGATCCAAAGCTGCTCTTAGCCCGTAAAGTAGTTGGATGGAAGGCGTATACGGCCAGTAAGTTCCAAGCTTGTAGAACTTCAAGTAGTCATTCCAGTCGAAGAAAACTCTGACCGACTTGGCATTTTTGGAAGCTTCCAGAGCTTTCGGGCTCACGCAAACAATGCCCATTCCAGTAGGCAGGGAAA-GTGCTTTCTGCGA

>HEID132290_Tillandsia_xiphioides_Clone3

TGCAAGATCATTTTACGCCAATTATGTTACAAGAGGTCATACAATTATAAATGGCAACTAATTACAT-------------------------------------------------------------------------------------------------CC-TCTTA----CATATTCC------------------TAGATAATGCAATAC---TGTAAATTT-----AGAAAAAA------------GCTAAAAGAAA-ACAATCGGCTATTCAGTAAAAGCCAGCTTAATTTTTACAGTATACTCGAT--CGATAGA-------TTTTTGCATACTTACCTCGTGGCCTTTCCTAAACGACGATGCCTTTCGATGACATTTTCGAGGCCCTCCTCAAAGATGAGATCCAAAGCTGCTCTTAGCCCGTAAAGTAGTTGGATGGAAGGCGTATACGGCCAGTAAGTTCCAAGCTTGTAGAACTTCAAGTAGTCATTCCAGTCGAAGAAAACTCTGACCGACTTGGCATTTTTGGAAGCTTCCAGAGCTTTCGGGCTCGCGCAAACAATGCCCATTCCAGTAGGCAGGGAAA-GTGCTTTCTGCGA

>HEID132303_Tillandsia_rectangula_Clone1

TGCAAGATCATTTTACGCCAA---------------TCATACAATTATAAATGACAACTAATTATAT-------------------------------------------------------------------------------------------------TCATCTTA----CATATTTCTATA----------------------------C---TGTAA-TTTTTTTTAAAAAAAAAA-----------CTAAAAGAAA-ATAATCGTCTATTCAGTAAAAGCCAGCTTAATTTTTACAGTATACTCGAT--GGATAGA-------TTTTTGCATACTTACCTCGTGGCCTTTCCTAAACGACGATGCCTTTCGATGACATTTTCGAGCCCCTCCTCAAAGATGAGATCCAAAGCTGCTCTTAGCCCGTAAAGTAGTTGGATGGAAGGCGTATACGGCCAGTAAGTTCCAAGCTTGTAGAACTTCAAATAGTCATTCCAGTCGAAGAAAACTCTGACCGACTTGGCATTTTTGGAAGCTTCCAGAGCTTTCGGGCTCGCGCAAACAATGCCCATTCCAGTAGGCAGGGAAA-GTGCTTTCTGCGA

>HEID132303_Tillandsia_rectangula_Clone2

TGCAAGATCATTTTACGCCAATTATGTTACAAGAGGTCATACAATTATAAATGGCAACTAATTACAT-------------------------------------------------------------------------------------------------CCATCTTATATACATATTCCTATATATATATATATGAATAGATAATGCAATAC---TGTAAATTT-----AGAAAAAA------------GCTAAAAGAAA-ACAATCGGCTATTCAGTAAAAGCCAGCTTAATTTTTACAGTATAATCGAT--CGATAGA-------TTTTTGCATACTTACCTCGTGGCCTTTCCTAAACGACGATGCCTTTCGATGACATTTTCGAGGCCCTCCTCAAAGATGAGATCCAAAGCTGCTCTTAGCCCGTAAAGTAGTTGGATGGAAGGCGTATACGGCCAGTAAGTTCCAAGCTTGTAGAACTTCAAGTAGTCATTCCAGTCGAAGAAAACTCTAACCGACTTGGCATTTTTGGAAGCTTCCAGAGCTTTCGGGCTCGCGCAAACAATGCCCATTCCAGTAGGCAGGGAAA-GTGCTTTCTGCGA

>HEID132303_Tillandsia_rectangula_Clone3

TGCAAGATCATTTTACGCCAA---------------TCATACAATTATAAATGACAACTAATTATAT-------------------------------------------------------------------------------------------------TCATCTTA----CATATTTCTATA----------------------------C---TGTAA-TTTTTTTTAAAAAAAAAA-----------CTAAAAGAAA-ATAATCGTCTATTCAGTAAAAGCCAGCTTAATTTTTACAGTATACTCGAT--GGATAGA-------TTTTTGCATACTTACCTCGTGGCCTTTCCTAAACGACGATGCCTTTCGATGACATTTTCGAGCCCCTCCTCAAAGATGAGATCCAAAGCTGCTCTTAGCCCGTAAAGTAGTTGGATGGAAGGCGTATACGGCCAGTAAGTTCCAAGCTTGTAGAACTTCAAATAGTCATTCCAGTCGAAGAAAACTCTGACCGACTTGGCATTTTTGGAAGCTTCCAGAGCTTTCGGGCTCGCGCAAACAATGCCCATTCCAGTAGGCAGGGAAA-GTGCTTTCTGCGA

>HEID132329_Tillandsia_kirschnekii

TGCAAGATCATTTTACGCCAATTATGTTACAAGAGGTCATACAATTATAAATGGCAACTAATTACAT-------------------------------------------------------------------------------------------------CCCTCTTA----CATATTCC------------------TGGATAATGCAATAC---TGTAAATTT-----AGAAAAAA------------GCTAAAAGAAA-ACAATCGGCTATTCAGTAAAAGCCAGCTTAATTTTTACAGTATACTCGAT--CGATAGA-------TTTTTGCATACTTACCTCGTGGCCTTTCCTAAACGACGATGCCTTTCGATGACATTTTCGAGGCCCTCCTCAAAGATGAGATCCAAAGCTGCTCTTAGCCCGTAAAGTAGTTGGATGGAAGGCGTATACGGCCAGTAAGTTCCAAGCTTGTAGAACTTCAAGTAGTCATTCCAGTCGAAGAAAACTCTGACCGACTTGGCATTTTTGGAAGCTTCCAGAGCTTTCGGGCTCGCGCAAACAATGCCCATTCCAGTAGGCAGGGAAAAGTGCTTTNNNNNN

>HEID132425_Tillandsia_paleacea_Clone1

TGCAAGATCATTTTACGCCAATTATGTTACAAGAGGTCATACAATTATAAATGGCAACTAATTACAT-------------------------------------------------------------------------------------------------CC-TCTTA----CATATTCC------------------TAGATAATGCAATAC---TGTAAATTT-----AGAAAAAA------------GCTAAAAGAAA-ACAATCGGCTATTCAGTAAAAGCCAGCTTAATTTTTACAGTATACTCGAT--CGATAGA-------TTTTTGCATACTTACCTCGTGGCCTTTCCTAAACGACGATGCCTTTCGATGACATTTTCGAGGCCCTCCTCAAAGATGAGATCCAAAGCTGCTCTTAGCCCGTAAAGTAGTTGGATGGAAGGCGTATACGGCCAGTAAGTTCCAAGCTTGTAGAACTTCAAGTAGTCATTCCAGTCGAAGAAAACTCTGACCGACTTGGCATTTTTGGAAGCTTCCAGAGCTTTCGGGCTCGGGCAAACAATGCCCATTCCAGTAGGCAGGGAAA-GTGCTTTCTGCGA

>HEID132425_Tillandsia_paleacea_Clone2

TGCAAGATCATTTTACGCCAA---------------TCATACAATTATAAATGACAACTAATTATAT-------------------------------------------------------------------------------------------------TCATCTTA----CATATTTCTATA----------------------------C---TGTAA-TTTTTTTTAAAAAAAAAA-----------CTAAAAGAAA-ATAATCGTCTATTCAGTAAAAGCCAGCTTAATTTTTACAGTATACTCGAT--GGATAGA-------TTTTTGCATACTTACCTCGTGGCCTTTCCTAAACGACGATGCCTTTCGATGACATTTTCGAGCCCCTCCTCAAAGATGAGATCCAAAGCTGCTCTTAGCCCGTAAAGTAGTTGGATGGAAGGCGTATACGGCCAGTAAGTTCCAAGCTTGTAGAACTTCAAATAGTCATTCCAGTCGAAGAAAACTCTGACCGACTTGGCATTTTTGGAAGCTTCCAGAGCTTTCGGGCTCGCGTAAACAATGCCCATTCCAGTAGGCAGGGAAA-GTGCTTTCTGCGA

>HEID132425_Tillandsia_paleacea_Clone3

TGCAAGATCATTTTACGCCAATTATGTTACAAGAGGTCATACAATTATAAATGGCAACTAATTACAT-------------------------------------------------------------------------------------------------CCCTCTTA----CATATTCC------------------TGGATAATGCAGTAC---TGTAAATTT-----AGAAAAAA------------GCTAAAAGAAA-ACAATCGGCTATTCAGTAAAAGCCAGCTTAATTTTTACAGTATACTCGAT--CGATAGA-------TTTTTGCATACTTACCTCGTGGCCTTTCCTAAACGACGATGCCTTTCGATGACATTTTCGAGGCCCTCCTCAAAGATGAGATCCAAAGCTGCTCTTAGCCCGTAAAGTAGTTGGATGGAAGGCGTATACGGCCAGTAAGTTCCAAGCTTGTAGAACTTCAAGTAGTCATTCCAGTCGAAGAAAACTCTGACCGACTTGGCATTTTTGGAAGCTTCCAGAGCTTTCGGGCTCGGACAAACAATGCCCATTCCAGTAGGCAGGGAAA-GTGCTTTCTGCGA

>HEID132479_Tillandsia_paleacea

TGCAAGATCATTTTACGCCAATTATGTTACAAGAGGTCATACAATTATAAATGGCAACTAATTACAT-------------------------------------------------------------------------------------------------CCCTCTTA----CATATTCC------------------TAGATAATGCAATAC---TGTAAATTT-----AGAAAAAA------------GCTAAAAGAAA-ACAATCGGCTATTCAGTAAAAGCCAGCTTAATTTTTACAGTATACTCGAT--CGATAGA-------TTTTTGCATACTTACCTCGTGGCCTTTCCTAAACGACGATGCCTTTCGATGACATTTTCGAGGCCCTCCTCAAAGATGAGATCCAAAGCTGCTCTTAGCCCGTAAAGTAGTTGGATGGAAGGCGTATACGGCCAGTAAGTTCCAAGCTTGTAGAACTTCAAGTAGTCATTCCAGTCGAAGAAAACTCTGACCGACTTGGCATTTTTGGAAGCTTCCAGAGCTTTCGGGCTCGCGCAAACAATGCCCATTCCAGTAGGCAGGGAAA-GTGCTTNNNNNNN

>HEID8112431_Tillandsia_virescens_Clone1

TGCAAGATCATTTTACGCCAA---------------TCATACAATTATAAATGGCAACTAATTACAT-------------------------------------------------------------------------------------------------TCATCTTA----CATATTCCTATATAT--------GAATAGATAATGCAATAC---TGT-----------------------------------------------------------------------------------------------GGATAGA-------TTTTTGCATACTTACCTCGTGGCCTTTCCTAAACGACGATGCCTTTCGATGACATTTTCGAGGCCCTCCTCAAAGATGAGATCCAAAGCTGCTCTTAGCCCGTAAAGTAGTTGGATGGAAGGCGTATACGGCCAGTAAGTTCCAAGCTTGTAGAACTTCAAGTAGTCGTTCCAGTCGAAGAAAACTCTGACCGACTTGGCATTTTTGGAAGCTTCCAGAGCTTTCGGGCTCGCGCAAACAATGCCCATTCCAGTAGGCAGGGAAA-GTGCTTTCTGCGA

>HEID8112431_Tillandsia_virescens_Clone2

TGCAAGATCATTTTACGCCAATTATGTTACAAGAGGTCATACAAATATAAATGGCAACTAATTACAT-------------------------------------------------------------------------------------------------TCATCTTA----CATATTCCTATATAT--------GAATAGATAATGCAATAC---TGT-----------------------------------------------------------------------------------------------GGATAGA-------TTTTTGCATACTTACCTCGTGGCCTTTCCTAAACGACGATGTCTTTCGATGACATTTTCGAGGCCCTCCTCAAAGATGAGATCCAAAGCTGCTCTTAGCCCGTAAAGTAGTTGGATGGAAGGCGTATACGGCCAGTAAGTTCCAAGCTTGTAGAACTTCAAGTAGTCGTTCCAGTCGAAGAAAACTCTGACCGACTTGGCATTTTTGGAAGCTTCCAGAGCTTTCGGGCTCGCGCAAACAATGCCCATTCCAGTAGGCAGGGAAA-GTGCTTTCTGCGA

>HEID8112431_Tillandsia_virescens_Clone3

TGCAAGATCATTTTACGCCAATTATGTTACAAGAGGTCATACAAATATAAATGGCAACTAATTACAT-------------------------------------------------------------------------------------------------TCATCTTA----CATATTCCTATATAT--------GAATAGATAATGCAATAC---TGT-----------------------------------------------------------------------------------------------GGATAGA-------TTTTTGCATACTTACCTCGTGGCCTTTCCTAAACGACGATGTCTTTCGATGACATTTTCGAGGCCCTCCTCAAAGATGAGATCCAAAGCTGCTCTTAGCCCGTAAAGTAGTTGGATGGAAGGCGTATACGGCCAGTAAGTTCCAAGCTTGTAGAACTTCAAGTAGTCGTTCCAGTCGAAGAAAACTCTGACCGACTTGGCATTTTTGGAAGCTTCCAGAGCTTTCGGGCTCGCGCAAACAATGCCCATTCCAGTAGGCAGGGAAA-GTGCTTTCTGCGA

>MHJB_B1044_Tillandsia_caticola

TGCAAGATCATTTTACGCCAA---------------TCATACAATTATAAATGCCAACTAATTATAT-------------------------------------------------------------------------------------------------CCATCTTA----CATATTCCTATATAT--------GAATAGATAATGCAATACTACTGTAAATTT-----AGAAAAAA------------GCTAAAAGAAA-ACAATCGGCTATTCAGTAAAAG----------------------------------------------TTTGCATACTTACCTCGTGGCCTTTCCTAAACGATGATGCCTTTCGATGACATTTTCGAGCCCCTCCTCAAAGATGAGATCCAAAGCTGCTCTAAGCCCGTAAAGTAGTTGGATGGAAGGCGTATACGGCCAGTAAGTTCCAAGCTTGTAGAACTTCAAGTAGTCATTCCAGTCGAAGAAAACTCTGACCGACTTGGCATTTTTAGAAGCTTCTAGAGCTTTCGGGCTCGCGCAAACAATGCCCATTCCAGTAGGCAGGGAAA-GTGCTTNNNNNNN

>MHJB_B1049_Tillandsia_duratii_Clone1

TGCAAGATCATTTTACGCAAATTATGTTACAAGAGGTCATACAATTATAAATGGCAACTAATTACAT-------------------------------------------------------------------------------------------------CCCTCTTA----CATATTCC------------------TAGATAATGCAATAC---TGTAAATTT-----AGAAAAAA------------GCTAAAAGAAA-ACAATCGGCTATTCAGTAAAAGCCAGCTTAATTTTTACAGTATACTCGAT--CGATAGA-------TTTTTGCATACTTACCTCGTGGCCTTTCCTAAACGACGATGCCTTTCGATGACATTTTCGAGGCCCTCCTCAAAGATGAGATCCAAAGCTGCTCTTAGCCCGTAAAGTAGTTGGATGGAAGGCGTATACGGCCAGTAAGTTCCAAGCTTGTAGAACTTCAAGTAGTCATTCCAGTCGAAGAAAACTCTGACCGACTTGGCATTTTTGGAAGCTTCCAGAGCTTTCGGGCTCGCGCAAACAATGCCCATTCCAGTAGGCAGGGAAA-GTGCTTTCTGCGA

>MHJB_B1049_Tillandsia_duratii_Clone2

TGCAAGATCATTTTACGCCAATTATGTTACAAGAGGTCATACAATTATAAATGGCAACTAATTACAT-------------------------------------------------------------------------------------------------CCCTCTTA----CATATTCC------------------TAGATAATGCAATGC---TGTAAATTT-----AGAAAAAA------------GCTAAAAGAAA-ACAATCGGCTATTCAGTAAAAGCCAGCTTAATTTTT--------------------AGATAGA---TTTTTGCATACTTACCTCGTGGCCTTTCCTAAACGACGATGCCTTTCGATGACATTTTCGAGGCCCTCCTCAAAGATGAGATCCAAAGCTGCTCTTAGCCCGTAAAGTAGTTGGATGGAAGGCGTATACGGCCAGTAAGTTCCAAGCTTGTAGAACTTCAAGTAGTCATTCCAGTCGAAGAAAACTCTAACCGACTTGGCATTTTTGGAAGCTTCCAGAGCTTTCGGGCTCGCGCAAACAATGCCCATTCCAGTAGGCAGGGAAA-GTGCTTTCTACGA

>MHJB_B1049_Tillandsia_duratii_Clone3

TGCAAGATCATTTTACGCAAATTATGTTACAAGAGGTCATACAATTATAAATGGCAACTAATTACAT-------------------------------------------------------------------------------------------------CCCTCTTA----CATATTCC------------------TAGATAATGCAATAC---TGTAAATTT-----AGAAAAAA------------GCTAAAAGAAA-ACAATCGGCTATTCAGTAAAAGCCAGCTTAATTTTTACAGTATACTCGAT--CGATAGA-------TTTTTGCATACTTACCTCGTGGCCTTTCCTAAACGACGATGCCTTTCGATGACATTTTCGAGGCCCTCCTCAAAGATGAGATCCAAAGCTGCTCTTAGCCCGTAAAGTAGTTGGATGGAAGGCGTATACGGCCAGTAAGTTCCAAGCTTGTAGAACTTCAAGTAGTCATTCCAGTCGAAGAAAACTCTGACCGACTTGGCATTTTTGGAAGCTTCCAGAGCTTTCGGGCTCGCGCAAACAATGCCCATTCCAGTAGGCAGGGAAA-GTGCTTTCTGCGA

>MHJB_B1050_Tillandsia_duratii

TGCAAGATCATTTTATGCCAATTATGTTACAAGAGGTCATACAATTATAAATGGCAACTAATTACAT-------------------------------------------------------------------------------------------------CCCTCTTA----CATATTCC------------------TAGATAATGCAATAC---TGTAAATTT-----AGAAAAAA------------GCTAAAAGAAA-ACAATCGGCTATTCAGTAAAAGCCAGCTTAATTTTTACAGTATACTCGAT--CGATAGA-------TTTTTGCATACTTACCTCGTGGCCTTTCCTAAACGACGATGCCTTTCGATGACATTTTCGAGGCCCTCCTCAAAGATGAGATCCAAAGCTGCTCTTAGCCCGTAAAGTAGTTGGATGGAAGGCGTATACGGCCAGTAAGTTCCAAGCTTGTAGAACTTCAAGTAGTCATTCCAGTCGAAGAAAACTCTGACCGACTTGGCATTTTTGGAAGCTTCCAGAGCTTTCGGGCTCGCGCAAACAATGCCCATTCCAGTAGGCAGGGAAA-GTGCTTTNNNNNN

>MHJB_B1050_Tillandsia_duratii_Clone2

TGCGAGATCATTTTATGCCAATTATGTTACAAGAGGTCATACAATTATAAATGGCAACTAATTACAT-------------------------------------------------------------------------------------------------CCCTCTTA----CATATTCC------------------TAGATAATGCAATAC---TGTAAATTT-----AGAAAAAA------------GCTAAAAGAAA-ACAATCGGCTATTCAGTAAAAGACAGCTTAATTTTTACAGTATACTCGAT--CGATAGA-------TTTTTGCATACTTACCTCGTGGCCTTTCCTAAACGACGATGCCTTTCGATGACATTTTCGAGGCCCTCCTCAAAGATGAGATCCAAAGCTGCTCTTAGCCCGTAAAGTAGTTGGATGGAAGGCGTATACGGCCAGTAAGTTCCAAGCTTGTAGAACTTCAAGTAGTCATTCCAGTCGAAGAAAACTCTGACTGACTTGGCATTTTTGGAAGCTTCCAGAGCTTTCGGGCTCGCGCAAACAATGCCCATTCCAGTAGGCAGGGAAA-GTGCTTTCTGCGA

>MHJB_B1050_Tillandsia_duratii_Clone3

TGCAAGATCATTTTACGCCAATTATGTTACAAGAGGTCATACAATTATAAATGGCAACTAATTACAT-------------------------------------------------------------------------------------------------CCCTCTTA----CATATTCC------------------TAGATAATGCAATAC---TGTAAATTT-----AGAAAAAA------------GCTAAAAGAAA-ACAATCGGCTATTCAGTAAAAGCCAGCTTAATTTTTACAGTATACTCGAT--CGATAGA-------TTTTTGCATACTTACCTCGTGGCCTTTCCTAAACGACGATGCCTTTCGATGACATTTTCGAGGCCCTCCTCAAAGATGAGATCCAAAGCTGCTCTTAGCCCGTAAAGTAGTTGGATGGAAGGCGTATACGGCCAGTAAGTTCCAAGCTTGTAGAACTTCAAGTAGTCATTCCAGTCGAAGAAAACTCTAACCGACTTGGCATTTTTGGAAGCTTCCAGAGCTTTCGGGCTCGCGCAAACAATGCCCATTCCAGTAGGCAGGGAAA-GTGCTTTCTGCGA

>MHJB_B1052_Tillandsia_humilis

TGTAAGATCATTTTACGCCAA---------------TCATACAATTATAAATGCCAACTAATTATAT-------------------------------------------------------------------------------------------------CCATCTTA----CATATTCCTATATAT--------GAATAGATAATGCAATAC---TGTCAATTT-----AGAAAAAA------------GCTAAAAGAAA-ACAATCGGCTATTCAGTAAAAA----------------------------------------------TTTGCATACTTACCTCGTGGCCTTTCCTAAACGATGATGCCTTTCGATGACATTTTCGAGCCCCTCCTCAAAGATGAGATCCAACGCTGCTCTTAGCCCGTAAAGTAGTTGGATGGAAGGCGTATACGGCCAGTAAGTTCCAAGCTTGTAGAACTTCAAGTAGTCATTCCAGTCGAAGAAAACTCTGACCGACTTGGCATTTTTGGAAGCTTCTAGAGCTTTCGGGCTCGCGCAAACAATGCCCATTCCAGTAGGCAGGGAAA-G-GCTTTCNNNNN

>MHJB_B1070_Tillandsia_purpurea

TGCAAGATCATTTTACGCCAA---------------TCATACAATTATAAATGCCAACTAATTATAT-------------------------------------------------------------------------------------------------CCATCTTA----CATATTCCTATATAT--------GAATAGATAATGCAATAC---TGTAAATTT-----AGAAAAAA------------GCTAAAAGAAA-ACAATCGGCTATTCAGTAAAAA----------------------------------------------TTTGCATACTTACCTCGTGGCCTTTCCTAAACGATGATGCCTTTCGATGACATTTTCGAGCCCCTCCTCAAAGATGAGATCCAAAGCTGCTCTTAGCCCGTAAAGTAGTTGGATGGAAGGCGTATACGGCCAGTAAGTTCCAAGCTTGTAGAACTTCAAGTAGTCATTCCAGTCGAAGAAAACTCTGACCGACTTGGCATTTTTGGAAGCTTCTAGAGCTTTCGGGCTCGCGCAAACAATGCCCATTCCAGTAGGCAGGGAAAAGTGCTTNNNNNNN

>MHJB_B1073_Tillandsia_streptocarpa

TGCAAGATCATTTTACGCCAATTANGTTACAANAGGTCATACAATTATAAATGGCAACTAATTACAT-------------------------------------------------------------------------------------------------CCCTCTTA----CATATTCN------------------TAGATAATGCAATAC---TGTAAATTT-----AGAAAAAA------------GCTAAAAGAAA-ACAATCGGCTATTCAGTAAAAGCCAGCTTAATTTTTACGGTATACTCGAT--CGATAGA-------TTTTTGCATACTTACCTCGTGGCCTTTCCTAAACGACGATGCCTTTCGATGACATTTTCGAGGCCCTCCTCAAAGATGAGATCCAAAGCTGCTCTTAGCCCGTAAAGTAGTTGGATGGAAGGCGTATACGGCCAGTAAGTTCCAAGCTTGTAGAACTTCAAGTAGTCATTCCAGTCGAAGAAAACTCTGACCGACTTGGCATTTTTGGAANNTTCCAGAGCTTTCGGGCTCGCGCAAACAATGCCCATTCCAGTAGGCANGGAAA-GTCCTTTNNNNNN

>MHJB_B1083_Tillandsia_straminea

TGCAAGATCATTTTATGCCAA---------------TCATACAATTATAAATGCCAACTAATTATAT-------------------------------------------------------------------------------------------------CCATCTTA----CATATTCCTATATAT--------GAATAGATAATGCAATAC---TGTAAATTT-----AGAAAAAAA-----------GCTAAAAGAAA-ACAATCGGCTATTCAGTAAAAA----------------------------------------------TTTGCATACTTACCTCGTGGCCTTTCCTAAACGATGATGCCTTTCGATGACATTTTCGAGCCCCTCCTCAAAGATGAGATCCAAAGCTGCTCTTAGCCCGTAAAGTAGTTGGATGGAAGGCGTATACGGCCAGTAAGTTCCAAGCTTGTAGAACTTCAAGTAGTCATTCCAGTCGAAGAAAACTCTGACCGACTTGGCATTTTTGGAAGCTTCTAGAGCTTTCGGGCTCGCGCAAACAATGCCCATTCCAGTAGGCAGAGAAA-GTGCTTNNNNNNN

>MHJB_B1085_Tillandsia_straminea

TGCAAGATCATTTTACGCCAA---------------TCATACAATTATAAATGCCAACTAATTATAT-------------------------------------------------------------------------------------------------CCATCTTA----CATATTCCTATATAT--------GAATAGATAATGCAATAC---TGTAAATTT-----AGAAAAAA------------GCTAAAAGAAA-ACAATCGGCTATTCAGTAAAAA----------------------------------------------TTTGCATACTTACCTCGTGGCCTTTCCTAAACGATGATGCCTTTCGATGACATTTTCGAGCCCCTCCTCAAAGATGAGATCCAAAGCTGCTCTTAGCCCGTAAAGTAGTTGGATGGAAGGCGTATACGGCCAGTAAGTTCCAAGCTTGTAGAACTTCAAGTAGTCATTCCAGTCGAAGAAAACTCTGACCGACTTGGCATTTTTGGAAGCTTCTAGAGCTTTCGGGCTCGCGCAAACAATGCCCATTCCAGTAGGCAGGGAAA-GTGCTTTNNNNNN

>MHJB_B1933_Tillandsia_werdermanii

TGCAAGATCATTTTACGCCAA---------------TCATACAATTATAAATGGCAACTAATTACAT-------------------------------------------------------------------------------------------------CCATCTTA----CATATTCCTATATAT--------GAATAGATAATGCAATAC---TGTAAATTT-----AGAAAACA------------GCTAAAAGAAA-ACAATCGGCTATTCAGTAAAAGCCAGC----TTTTTACAGTATACTCGAT--CGATAGA-AG------TTTGCATACTTACCTCGTGGCCTTTCCTAAACGACGATGCCTTTCGATGACATTTTCGAGGCCCTCCTCAAAGATGAGATCCAAAGCTGCTCTTAGCCCGTAAAGTAGTTGGATGGAAGGCGTATACGGCCAGTAAGTTCCAAGCTTGTAGAACTTCAAGTAGTCATTCCAGTCGAAGAAAACTCTGACCGACTTGGCATTTTTGGAAGCTTGCAGAGCTTTCGGGCTAGCGCAAACAATGCCCATTCCAGTAGGCAGGGAAA-GTGCTTNNNNNNN

>MHJB_B214_Tillandsia_aff_streptocarpa_Clone1

TGCAAGATCATTTTACGCCAATTATGTTACAAGAGGTCATACAATTATAAATGGCAACTAATTACAT-------------------------------------------------------------------------------------------------CC-TCTTA----CATATTCC------------------TAGATAATGCAATAC---TGTAAATTT-----AGAAAAAA------------GCTAAAAGAAA-ACAATCGGCTATTCAGTAAAAGCCAGCTTAATTTTTACAGTATACTCGAT--CGATAGA-------TTTTTGCATACTTACCTCGTGGCCTTTCCTAAACGACGATGCCTTTCGATGACATTTTCGAGGCCCTCCTCAAAGATGAGATCCAAAGCTGCTCTTAGCCCGTAAAGTAGTTGGATGGAAGGCGTATACGGCCAGTAAGTTCCAAGCTTGTAGAACTTCAAGTAGTCATTCCAGTCGAAGAAAACTCTGACCGACTTGGCATTTTTGGAAGCTTCCAGAGCTTTCGGGCTCGCGCAAACAATGCCCATTCCAGTAGGCAGGGAAA-GTGCTTTCTGCGA

>MHJB_B214_Tillandsia_aff_streptocarpa_Clone2

TGCAAGATCATTTTACGCCAATTATGTTACAAGAGGTCATACAATTATAAATGGCAACTAATTACAT-------------------------------------------------------------------------------------------------CC-TCTTA----CATATTCC------------------TAGATAATGCAATAC---TGTAAATTT-----AGAAAAAA------------GCTAAAAGAAA-ACAATCGGCTATTCAGTAAAAGCCAGCTTAATTTTTACAGTATACTCGAT--CGATAGA-------TTTTTACATACTTACCTCGTGGCCTTTCCTAAACGACGATGCCTTTCGATGACATTTTCGAGGCCCTCCTCAAAGATGAGATCCAAAGCTGCTCTTAGCCCGTAAAGTAGTTGGATGGAAGGCGTATACGGCCAGTAAGTTCCAAGCTTGTAGAACTTCAAGTAGTCATTCCAGTCGAAGAAAACTCTGACCGACTTGGCATTTTTGGAAGCTTCCAGAGCTTTCGGGCTCGCGCAAACAATGCCCATTCCAGTAGGCAGGGAAA-GTGCTTTCTGCGA

>MHJB_B214_Tillandsia_aff_streptocarpa_Clone3

TGCAAGATCATTTTACGCCAATTATGTTACAAGAGGTCATACAATTATAAATGGCAACTAATTACAT-------------------------------------------------------------------------------------------------CC-TCTTA----CATATTCC------------------TAGATAATGCAATAC---TGTAAATTT-----AGAAAAAA------------GCTAAAAGAAA-ACAATCGGCTATTCAGTAAAAGCCAGCTTAATTTTTACAGTATACTCGAT--CGATAGA-------TTTTTGCATACTTACCTCGTGGCCTTTCCTAAACGACGATGCCTTTCGATGACATTTTCGAGGCCCTCCTCAAAGATGAGATCCAAAGCTGCTCTTAGCCCGTAAAGTAGTTGGATGGAAGGCGTATACGGCCAGTAAGTTCCAAGCTTGTAGAACTTCAAGTAGTCATTCCAGTCGAAGAAAACTCTGACCGACTTGGCATTTTTGGAAGCTTCCAGAGCTTTCGGGCTCGCGCAAACAATGCCCATTCCAGTAGGCAGGGAAA-GTGCTTTCTGCGA

>MHJB_B214_Tillandsia_aff_streptocarpa_Clone4

TGCAAGATCATTTTACGCAAATTATGTTACAAGAGGTCATACAATTATAAATGGCAACTAATTACAT-------------------------------------------------------------------------------------------------CCCTCTTA----CATATTCC------------------TAGATAATGCAATAC---TGTAAATTT-----AGAAAAAA------------GCTAAAAGAAA-ACAATCGGCTATTCAGTAAAAGCCAGCTTAATTTTTACAGTATACTCGAT--CGATAGA-------TTTTTGCATACTTACCTCGTGGCCTTTCCTAAACGACGATGCCTTTCGATGACATTTTCGAGGCCCTCCTCAAAGATGAGATCCAAAGCTGCTCTTAGCCCGTAAAGTAGTTGGATGGAAGGCGTATACGGCCAGTAAGTTCCAAGCTTGTAGAACTTCAAGTAGTCATTCCAGTCGAAGAAAACTCTGACCGACTTGGCATTTTTGGAAGCTTCCAGAGCTTTCGGGCTCGCGCAAACAATGCCCATTCCAGTAGGCAGGGAAA-GTGCTTTCTGCGA

>MHJB_B214_Tillandsia_aff_streptocarpa_Clone_5

TGCAAGATCATTTTACGCAAATTATGTTACAAGAGGTCATACAATTATAAATGGCAACTAATTACAT-------------------------------------------------------------------------------------------------CCCTCTTA----CATATTCC------------------TAGATAATGCAATAC---TGTAAATTT-----AGAAAAAA------------GCTAAAAGAAA-ACAATCGGCTATTCAGTAAAAGCCAGCTTAATTTTTACAGTATACTCGAT--CGATAGA-------TTTTTGCATACTTACCTCGTGGCCTTTCCTAAACGACGATGCCTTTCGATGACATTTTCGAGGCCCTCCTCAAAGATGAGATCCAAAGCTGCTCTTAGCCCGTAAAGTAGTTGGATGGAAGGCGTATACGGCCAGTAAGTTCCAAGCTTGTAGAACTTCAAGTAGTCATTCCAGTCGAAGAAAACTCTGACCGACTTGGCATTTTTGGAAGCTTCCAGAGCTTTCGGGCTCGCGCAAACAATGCCCATTCCAGTAGGCAGGGAAA-GTGCTTTCTGCGA

>MHJB_B235_Tillandsia_krahnii

TGCAAGATCATTTTACGCCAATTATGTTACAAGAGGTCATACAATTATAAATGCCAACTAATTATAT-------------------------------------------------------------------------------------------------CCATCTTA----CATACTCCTATGTAT--------GAATAGATAATGCAATAC---TGTAAATTT-----AGGAAAAA------------GCTAAAAGAAA-ACAATCGGCTATT--------------------------------TCGAT--CGATAGA-AG------TTTGCATACTTACCTCGTGGCCTTTCCTAAACGACGATGCCTTTCGATGACATTTTCGAGCCCCTCCTCAAAGATGAGATCCAAAGCTGCTCTTAGCCCGTAAAGTAGTTGGATGGAAGGCGTATACGGCCAGTAAGTTCCAAGCTTGTAGAACTTCAAGTAGTCATTCCAGTCGAAGAAAACTCTGACCGACTTGGCATTTTTTGAAGCTTCTAGAGCTTTCGGGCTCGCGCAAACAATGCCCATTCCAGTAGGCAGGGAAA-GTGCTTTNNNNNN

>MHJB_B418_Tillandsia_prolata

TGCAAGATCATTTTACGCCAA---------------TCATACAATTATAAATGGCAACTAATTACAT-------------------------------------------------------------------------------------------------CCATCTTA----CATATTCCTATATAT--------GAATAGATAATGCAATAC---TGTAAATTT-----AGAAAACA------------GCTAAAAGAAA-ACAATCGGCTATTCAGTAAAAGCCAAC----TTTTTACAGTATACTCGAT--CGATAGA-AG------TTTGCATACTTACCTCGTGGCCTTTCCTAAACGACGATGCCTTTCGATGACATTTTCGAGGCCCTCCTCAAAGATGAGATCCAAAGCTGCTCTTAGCCCGTAAAGTAGTTGGATGGAAGGCGTATACGGCCAGTAAGTTCCAAGCTTGTAGAACTTCAAGTAGTCATTCCAGTCGAAGAAAACTCTGACCGACTTGGCATTTTTGGAAGCTTGCAGAGCTTTCGGGCTAGCGCAAACAATGCCCATTCCAGTAGGCAGGGAAA-GTGCTTTNNNNNN

>HEID131788_MT038709_1_Tillandsia_recurvata_BRC242

nnnnnnnnnnTTTTACGCCAATTATGTTACAAGAGGTCATACAATTATAAATGRCAACTAATTACAT-------------------------------------------------------------------------------------------------CCCTCTTA----CATATTCC------------------TGGATAATGCAATAC---TGTAAATTT-----AGAAAAAA------------GCTAAAAGAAA-ACAATCGGCTATTCAGTAAAAGCCAGCTTAATTTTTACAGTATACTCGAT--CGATAGA-------KTTTTGCATACTTACCTCGTGGCCTTTCCTAAACGACGATGCCTTTCGATGACATTTTCGAGGCCCTCCTCAAAGATGAGATCCAAAGCTGCTCTTAGCCCGTAAAGTAGTTGGATGGAAGGCGTATACGGCCAGTAAGTTCCAAGCTTGTAGAACTTCAAGTAGTCATTCCAGTCGAAGAAAACTCTGACCGACTTGGCATTTTTGGAAGCTTCCAGAGCTTTCRGGCTCGCGCAAACAATGCCCATTCCAGTAGGCnnnnnnn-nnnnnnnnnnnnn

>HEID131619_MT038729_1_Tillandsia_capillaris_BRC420

TGCAAGATCATTTTACGCCAATTATGTTACAAGAGGTCATACAATTATAAATGGCAACTAATTACAT-------------------------------------------------------------------------------------------------TCATCTTATATGCATATTCCTATATAT--------GAATAGATAATGCAATAC---TGTRAATTT-----AGAAAAAA------------GCTAAAAGAAA-ACAATTGGCTATTCAGTAAAAGCCAGCTTAATTTTTACAGTATACTCGAT--CGATAGATAGA---TTTTCGCATACTTACCTCGTGGCCTTTCCTAAACGACGATGCCTTTCGATGACATTTTCGAGGCCCTCCTCAAAGATGAGATCCAAAGCTGCTCTTAGCCCGTAAAGTAGTTGGATGGAAGGCGTATACGGCCAGTAAGTTCCAAGCTTGTAGAACTTCAAGTAGTCATTCCAGTCGAAGAAAACTCTGACCGACTTGGCATTTTTGGAAGCTTCCAGAGCTTTCGGGCTCGCGCAAACAATGCCCATTCCAGTAGGCAGGGAAA-GTGCTTTCTGCGA

>HEID131664_MT038730_1_Tillandsia_capillaris_BRC421

TGCAAGATCATTTTACGCCAATTATGTTACAAGAGGTCATACAATTATAAATGGCAACTAATTACAT-------------------------------------------------------------------------------------------------TCATCTTATATGCATATTCCTATATAT--------GAATAGATAATGCAATAC---WGTAAATTT-----AGAAAAAA------------ACTAAAAGAAA-ACAATCGGCTATTCAGTAAAAGCCAGCTTAATTTTTACAGTATACTCGAT--SGATAGATAGA---TTTTTGCATACTTACCTCGTGGCCTTTCCTAAACGACGATGCCTTTCGATGACATTTTCGAGGCCCTCCTCAAAGATGAGATCCAAAGCTGCTCTTAGCCCGTAAAGTAGTTGGATGGAAGGCGTATACGGCCAGTAAGTTCCGAGCTTGTAGAACTTCAAGTAGTCATTCCAGTCGAAGAAAACTCTGACCGACTTGGCATTTTTGGAAGCTTCCAGAGCTTTCGGGCTCGCGCAAACAATGCCCATTCCAGTAGGCAGGGAAA-nnnnnnnnnnnnn

>HEID103946_MT038742_1_Tillandsia_kirschnekii_BRC246

nnnnnnnnnnnnnnnnnnnnnnnnnnnnnnnnGAGGTCATACAATTATAAATGRCAACTAATTACAT-------------------------------------------------------------------------------------------------CCCTCTTA----CATATTCC------------------TRGATAATGCAATAC---TGTAAATTT-----AGAAAAAA------------GCTAAAAGAAA-ACAATCGKCTATTCAGTAAAAGCCAGCTTAATTTTTACAGTATACTCGAT--CGATAGA-------TTTTTGCATACTTACCTCGTGGCCTTTCCTAAACGACGATGCCTTTCGATGACATTTTCGAGSCCCTCCTCAAAGATGAGATCCAAAGCTGCTCTTAGCCCGTAAAGTAGTTGGATGGAAGGCGTATACGGCCAGTAAGTTCCAAGCTTGTAGAACTTCAARTAGTCATTCCAGTCGAAGAAAACTCTGACCGACTTGGCATTTTTGGAAGCTTCCAGAGCTTTCGGGCTCGCGCAAACAATGCCCATTCCAGTAGGCAGGGAAA-GTGCTTTCTGCGA

>HEID132425_MT038743_1_Tillandsia_paleacea_BRC341

TGCAAGATCATTTTACGCCAATTATGTTACAAGAGGTCATACAATTATAAATGGCAACTAATTACAT-------------------------------------------------------------------------------------------------CCCTCTTA----CATATTCC------------------TRGATAATGCARTAC---TGTAAATTT-----AGAAAAAA------------GCTAAAAGAAA-ACAATCGGCTRTTCARTAAAAGCCAGCTTAATTTTTACAGTATACTCGAT--CGATAGA-------TTTTTGCATACTTACCTCGTGGCCTTTCCTAAACGACGATGCCTTTCGATGACATTTTCGAGGCCCTCCTCAAAGATGAGATCCAAAGCTGCTCTTAGCCCGTAAAGTAGTTGGATGGAAGGCGTATACGGCCAGTAAGTTCCAAGCTTGTAGAACTTCAAGTAGTCATTCCAGTCGAAGAAAACTCTGACCGACTTGGCATTTTTGGAAGCTTCCAGAGCTTTCGGGCTCGSGCAAACAATGCCCATTCCAGTAGGCAGGGAAA-GTGCTTTCTGCGA

>HEID104854_MT038756_1_Tillandsia_purpurea_BRC299

TGCAAGATCATTTTACGCCAA---------------TCATACAATTATAAATGCCAACTAATTATAT-------------------------------------------------------------------------------------------------CCATCTTA----CATATTCCTATATATAT------GAATAGATAATGCAATAC---TGTAAATTT-----AGGAAAAA------------GCTAAAAGAAA-ACAATCGGCTATTCAGTAAAAGSCAGC----GTTTTACAGTA--CTCGAT--CGATAGA-AG------TTTGCATACTTACCTCGTGGCCTTTCCTAAACGATGATGCCTTTCGATGACATTTTCGAGCCCCTCCTCAAAGATGAGATCCAAAGCTGCTCTTAGCCCGTAAAGTAGTTGGATGGAAGGCGTATACGGCCAGTAAGTTCCGAGCTTGTAGAACTTCAAGTAGTCATTCCAGTCGAAGAAAACTCTGACCGACTTGGCATTTTTGGAAGCTTCTAGAGCTTTCGGGCTCGCGCAAACAATGCCCATTCCAGTAGGCAGGGAAA-GTGCTTTCTGCGA

>HEID131417_MT038832_1_Tillandsia_humilis_BRC258

TGCAAGATCATTTTACGCCAA---------------TCATACAATTATAAATGCCAACTAATTATATCCAGAGCCTTGTTGTTCTTTTGATGTAGCTGAGTTCATTTTTCATTGAATGAAGCGGCAACATGCTGCCTTTCTCTCGAAAAAAAAACTAATTATATCCATCTTA----CATATTCCTATATAT--------GAATAGATAATGTAATAC---TGTAAATTT-----AGGAAATAA-----------GCTAAAAGAAA-ACAATCGGCTATTCAGTAAAAG----------------------------------------------TTTGCATACTTACCTCGTGGCCTTTCCTAAACGATGATGCCTTTCGATGACATTTTCGAGCCCCTCCTCAAAGATGAGATCCAAAGCTGCTCTTAGCCCGTAAAGTAGTTGGATGGAAGGCGTATACGGCCAGTAAGTTCCAAGCTTGTAGAACTTCAAGTAGTCATTCCAGTCGAAGAAAACTCTGACCGACTTGGCATTTTTGGAAGCTTCTAGAGCTTTCGGGCTCGCGCAAACAATGCCCATTCCAGTAGGCAGGGAAA-GTGCTTTCTGCGA

>MT038877_1_Tillandsia_paleacea_BRC491

TGCAAGATCATTTTACGCCAATTATGTTACAAGAGGTCATACAATTATAAATGGCAACTAATTACAT-------------------------------------------------------------------------------------------------CCCTCTTA----CATATTCC------------------TAGATAATGCAATAC---TGTAAATTT-----AGAAAAAA------------GCTAAAAGAAA-ACAATCGGCTATTCAGTAAAAGCCAGCTTAATTTTTACAGTATACTCGAT--CGATAGA-------TTTTTGCATACTTACCTCGTGGCCTTTCCTAAACGACGATGCCTTTCGATGACATTTTCGAGGCCCTCCTCAAAGATGAGATCCAAAGCTGCTCTTAGCCCGTAAAGTAGTTGGATGGAAGGCGTATACGGCCAGTAAGTTCCAAGCTTGTAGAACTTCAAGTAGTCATTCCAGTCGAAGAAAACTCTGACCGACTTGGCATTTTTGGAAGCTTCCAGAGCTTTCGGGCTCGCGCAAACAATGCCCATTCCAGTAGGCAGGGAAA-nnnnnnnnnnnnn

>HEID131639_MT047482_1_Tillandsia_streptocarpa_BRC460

TGCAAGATCATTTTACGCCAATTATGTTACAAGAGGTCATACAATTATAAATGGCAACTAATTACAT-------------------------------------------------------------------------------------------------CCCTCTTA----CATATTCC------------------TAGATAATGCAATAC---TGTAAATTT-----AGAAAAAA------------GCTAAAAGAAA-ACAATCGGCTATTCAGTAAAAGCCAGCTTAATTTTTACAGTATACTCGAT--CGATAGA-------TTTTTGCATACTTACCTCGTGGCCTTTCCTAAACGACGATGCCTTTCGATGACATTTTCGAGGCCCTCCTCAAAGATGAGATCCAAAGCTGCTCTTAGCCCGTAAAGTAGTTGGATGGAAGGCGTATACGGCCAGTAAGTTCCAAGCTTGTAGAACTTCAAGTAGTCATTCCAGTCGAAGAAAACTCTGACCGACTTGGCATTTTTGGAAGCTTCCAGAGCTTTCGGGCTCGCGCAAACAATGCCCATTCCAGTAGGCAGGGAAA-GTGCTTTCTGCGA

>TP089_Tillandsia_pupurea_Clone2

TGCAAGATCATTTTATGCCAA---------------TCATACAATTATAAATGCCAACTAATTATAT-------------------------------------------------------------------------------------------------CCATCTTA----CATATTCCTATATAT--------GAATAGATAATGCAATAT---TGTAAATTT-----AGGAAAAAA-----------GCTAAAAGAAA-ACAATCGGCTATTCAGTAAAAG----------------------------------------------TTTGCATACTTACCTCGTGGCCTTTCCTAAACGATGATGCCTTTCGATGACATTTTCGAGCCCCTCCTCAAAGATGAGATCCAAAGCTGCTCTTAGCCCGTAAAGTAGTTGGATGGAAGGCGTATACGGCCAGTAAGTTCCAAGCTTGTAGAACTTCAAGTAGTCATTCCAGTCGAAGAAAACTCTGACCGACTTGGCATTTTTGGAAGCTTCTAGAGCTTTCGGGCTCGCGCAAACAATGCCCATTCCAGTAGGCAGGGAAA-GTGCTTTCTGCGA

>TP089_Tillandsia_pupurea_Clone3

TGCAAGATCATTTTACGCCAA---------------TCATACAATTATAAATGCCAACTAATTATAT-------------------------------------------------------------------------------------------------CCATCTTA----CATATTCCTATATAT--------GAATAGATAATGCAATAC---TGTAAATTT-----AGAAAAAA------------GCTAAAAGAAA-ACAATCGGCTATTCAGTAAAAG----------------------------------------------TTTGCATACTTACCTCGTGGCCTTTCCTAAACGATGATGCCTTTCGATGACATTTTCGAGCCCCTCCTCAAAGATGAGATCCAAAGCTGCTCTTAGCCCGTAAAGTAGTTGGATGGAAGGCGTATACGTCCAGTAAGTTCCAAGCTTGTAGAACTTCAAGTAGTCATTCCAGTCGAAGAAAACTCTGACCGACTTGGCATTTTTGGAAGCTTCTAGAGCTTTCGGGCTCGCGCAAACAATGCCCATTCCAGTAGGCAGGGAAA-GTGCTTTCTGCGA

>TP089_Tillandsia_pupurea_Clone4

TGCAAGATCATTTTACGCCAA---------------TCATACAATTATAAATGCCAACTAATTATAT-------------------------------------------------------------------------------------------------CCATCTTA----CATATTCCTATATAT--------GAATAGATAATGCAATAC---TGTAAATTT-----AGAAAAAA------------GCTAAAAGAAA-ACAATCGGCTATTCAGTAAAAG----------------------------------------------TTTGCATACTTACCTCGTGGCCTTTCCTAAACGATGATGCCTTTCGATGACATTTTCGAGCCCCTCCTCAAAGATGAGATCCAAAGCTGCTCTTAGCCCGTAAAGTAGTTGGATGGAAGGCGTATACGGCCAGTAAGTTCCAAGCTTGTAGAACTTCAAGTAGTCATTCCAGTCGAAGAAAACTCTGACCGACTTGGCATTTTTGGAAGCTTCTAGAGCTTTCGGGCTCGCGCAAACAATGCCCATTCCAGTAGGCAGGGAAA-GTGCTTTCTGCGA
