# Supplementary material for: The different routes of parallel evolution in epiarenic growth in a hyperarid desert environment
Source: Front Plant Sci. 2026 Jul 7;17:1822909. doi: 10.3389/fpls.2026.1822909 (PMC13392990; doi:10.3389/fpls.2026.1822909)

Supplementary Material 007

Maximum-likelihood tree of plastome data. Bootstrap-values are provided with the respective branch and given as %. GenBank accession codes are indicated with the taxon name. The outgroup/root was set with *Brocchinia micrantha*.

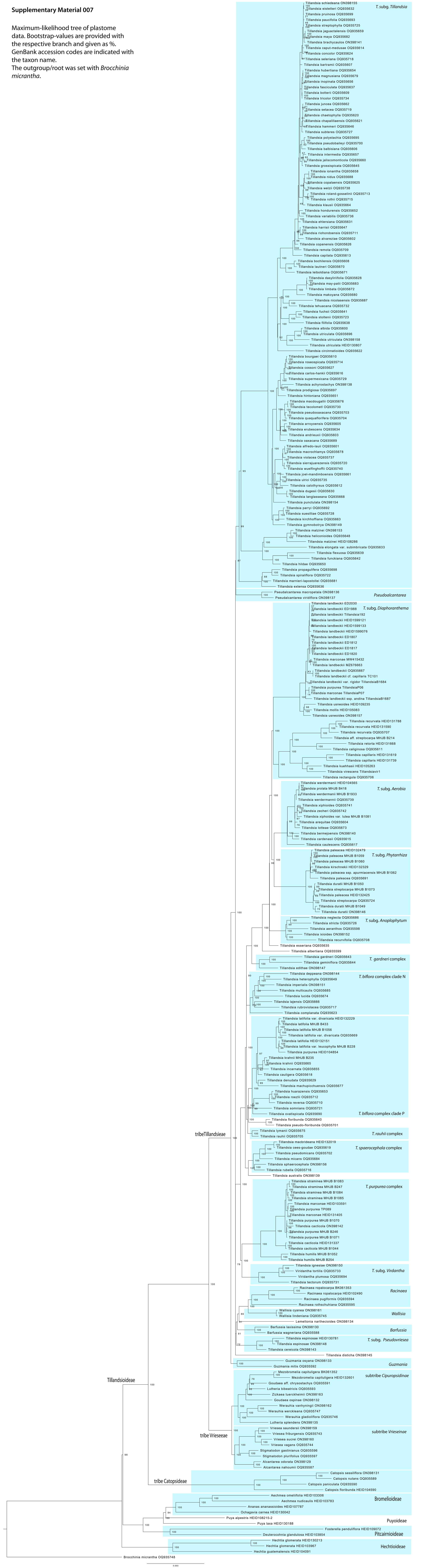

Supplement: Supplementary file 7 [file SupplementaryFile7.pdf]
