# Supplementary material for: The different routes of parallel evolution in epiarenic growth in a hyperarid desert environment
Source: Front Plant Sci. 2026 Jul 7;17:1822909. doi: 10.3389/fpls.2026.1822909 (PMC13392990; doi:10.3389/fpls.2026.1822909)
Supplement: Supplementary file 8 [file SupplementaryFile8.pdf]

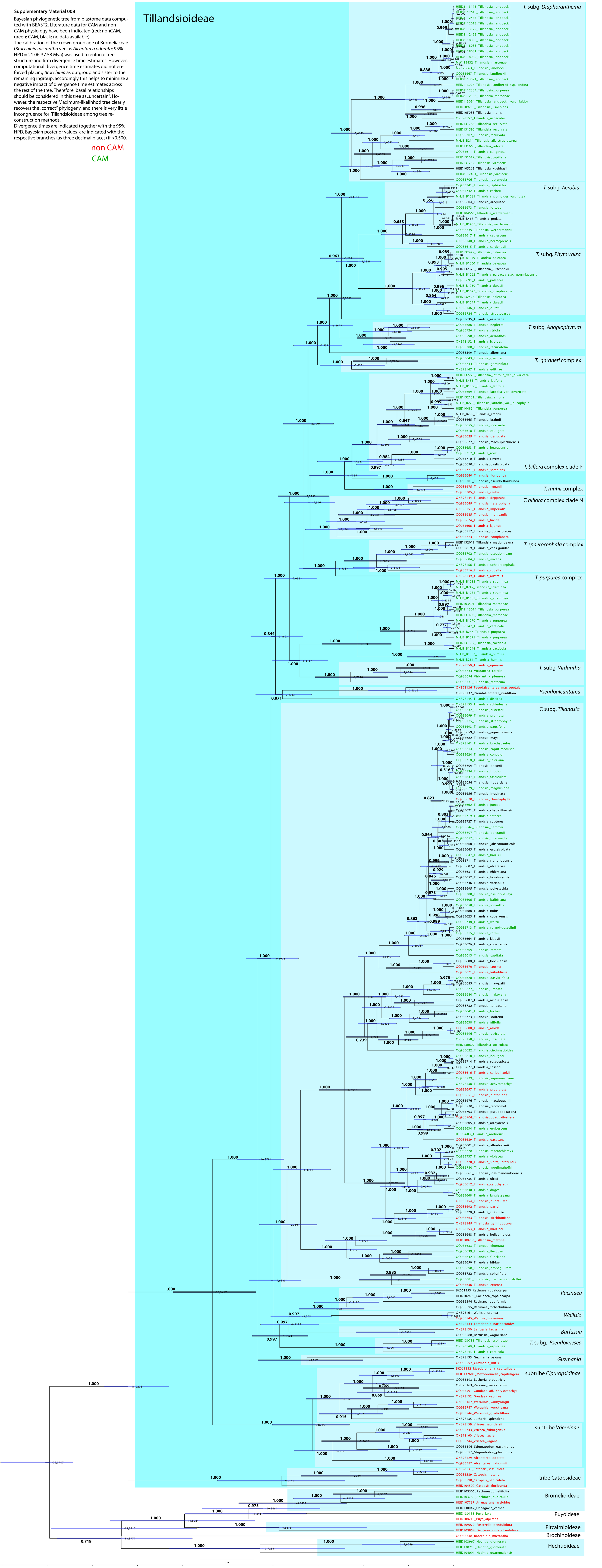

Supplementary Material 008

Bayesian phylogenetic tree from plastome data computed with BEAST2. Literature data for CAM and non CAM physiology have been indicated (red: nonCAM, green: CAM, black: no data available).
The calibration of the crown group age of Bromeliaceae (*Brocchinia micrantha* versus *Alcantarea odorata*; 95% HPD = 21.06-37.58 Mya) was used to enforce tree structure and firm divergence time estimates. However, computational divergence time estimates did not enforced placing *Brocchinia* as outgroup and sister to the remaining ingroup; accordingly this helps to minimize a negative impact of divergence time estimates across the rest of the tree. Therefore, basal relationships should be considered in this tree as „uncertain". However, the respective Maximum-likelihhod tree clearly recovers the „correct" phylogeny, and there is very little incongruence for Tillandsioideae among tree reconstruction methods.
Divergence times are indicated together with the 95% HPD. Bayesian posterior values are indicated with the respective branches (as three decimal places) if >0.500.

non CAM

CAM
