# Supplementary material for: The different routes of parallel evolution in epiarenic growth in a hyperarid desert environment
Source: Front Plant Sci. 2026 Jul 7;17:1822909. doi: 10.3389/fpls.2026.1822909 (PMC13392990; doi:10.3389/fpls.2026.1822909)

Supplementary Material 009

Agt1 allelic diversity and genetic relationships of epiarenic *Tillandsia* species and their closest relatives computed as Maximum-likelihood tree. The five phylogenetic groups, which include epiarenic *Tillandsia* species are underlaid in blue and marked (A-E; see Fig. 2). All *agt1* alleles obtained from cloned PCR products are named accordingly. Bootstrap values are provided along branches in %.

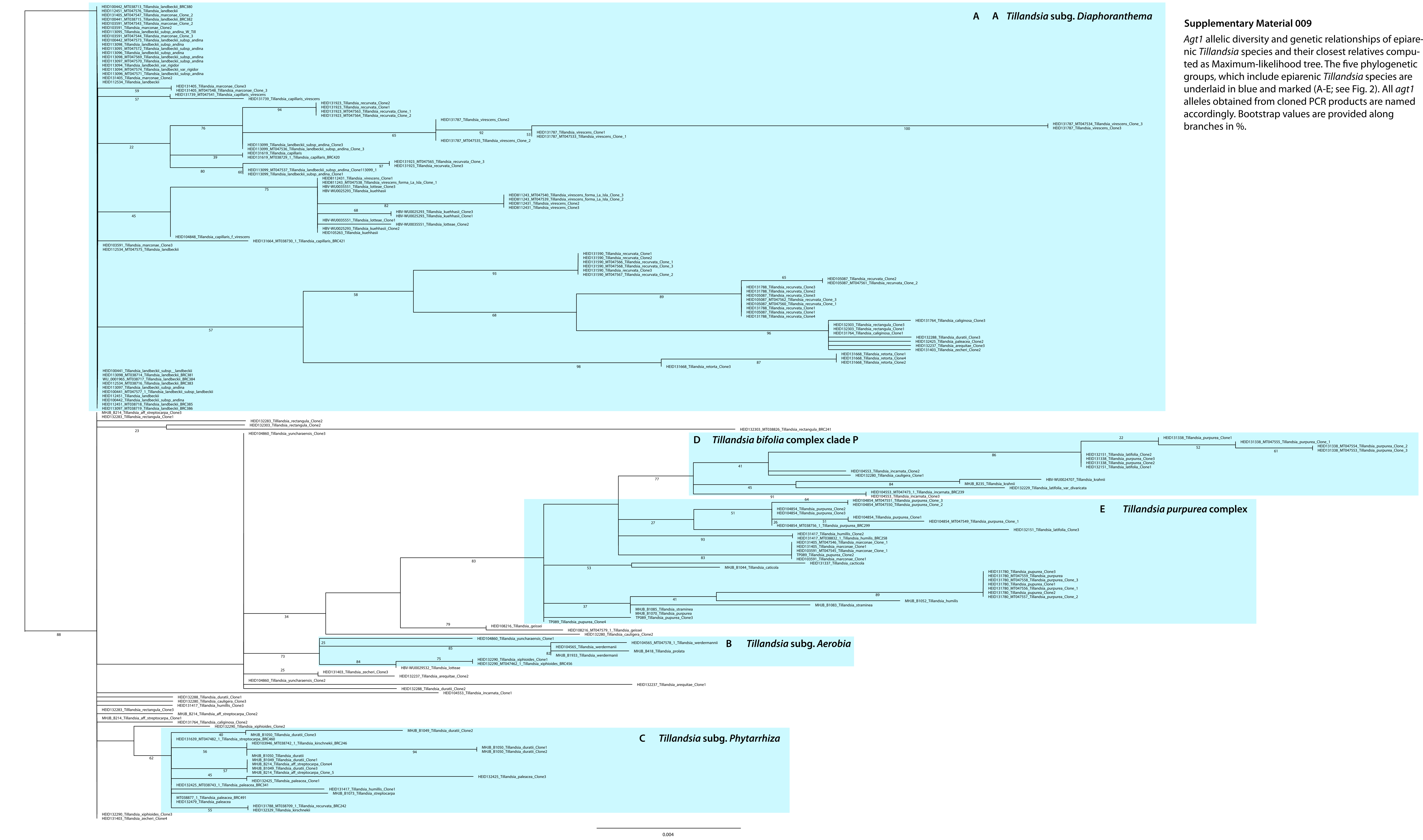

Supplement: Supplementary file 9 [file SupplementaryFile9.pdf]
